# Membrane disruption, but not metabolic rewiring, is the key mechanism of anticancer-action of FASN-inhibitors: a multi-omics analysis in ovarian cancer

**DOI:** 10.1038/s41598-020-71491-z

**Published:** 2020-09-10

**Authors:** Thomas W. Grunt, Astrid Slany, Mariya Semkova, Ramón Colomer, María Luz López-Rodríguez, Michael Wuczkowski, Renate Wagner, Christopher Gerner, Gerald Stübiger

**Affiliations:** 1 Cell Signaling and Metabolism Networks Program, Division of Oncology, Department of Medicine I, Medical University of Vienna, Waehringer Guertel 18-20, 1090 Vienna, Austria; 2 Comprehensive Cancer Center, Vienna, Austria; 3 Ludwig Boltzmann Institute for Hematology and Oncology, Vienna, Austria; 4 Department of Analytical Chemistry, University of Vienna, Vienna, Austria; 5 Department of Medical Oncology, Hospital Universitario La Princesa and Spanish National Cancer Research Centre (CNIO), Clinical Research Program, Madrid, Spain; 6 Departamento de Química Orgánica I, Facultad de Ciencias Químicas, Universidad Complutense de Madrid, Madrid, Spain; 7 Department of Biomedical Imaging and Image-Guided Therapy, Medical University of Vienna, Vienna, Austria

**Keywords:** Lipidomics, Lipids, Metabolomics, Proteomics, Cancer metabolism, Ovarian cancer, Proteomics, Biochemical networks, Cancer metabolism, Targeted therapies, Ovarian cancer, Cancer

## Abstract

Fatty-acid(FA)-synthase(FASN) is a druggable lipogenic oncoprotein whose blockade causes metabolic disruption. Whether drug-induced metabolic perturbation is essential for anticancer drug-action, or is just a secondary—maybe even a defence response—is still unclear. To address this, SKOV3 and OVCAR3 ovarian cancer(OC) cell lines with clear cell and serous histology, two main OC subtypes, were exposed to FASN-inhibitor G28UCM. Growth-inhibition was compared with treatment-induced cell-metabolomes, lipidomes, proteomes and kinomes. SKOV3 and OVCAR3 were equally sensitive to low-dose G28UCM, but SKOV3 was more resistant than OVCAR3 to higher concentrations. Metabolite levels generally decreased upon treatment, but individual acylcarnitines, glycerophospholipids, sphingolipids, amino-acids, biogenic amines, and monosaccharides reacted differently. Drug-induced effects on central-carbon-metabolism and oxidative-phosphorylation (OXPHOS) were essentially different in the two cell lines, since drug-naïve SKOV3 are known to prefer glycolysis, while OVCAR3 favour OXPHOS. Moreover, drug-dependent increase of desaturases and polyunsaturated-fatty-acids (PUFAs) were more pronounced in SKOV3 and appear to correlate with G28UCM-tolerance. In contrast, expression and phosphorylation of proteins that control apoptosis, FA synthesis and membrane-related processes (beta-oxidation, membrane-maintenance, transport, translation, signalling and stress-response) were concordantly affected. Overall, membrane-disruption and second-messenger-silencing were crucial for anticancer drug-action, while metabolic-rewiring was only secondary and may support high-dose-FASN-inhibitor-tolerance. These findings may guide future anti-metabolic cancer intervention.

## Introduction

The rewiring of cell metabolism has been a well-known hallmark of cancer for a long time. Recent evidence suggests that many well-established oncoproteins and tumour suppressors directly control cell metabolism, thereby determining the characteristic cell phenotype of cancer. The metabolic pathways of cancer cells have therefore attracted increasing attention as promising reservoir for novel cancer drug targets^1–3^. One of those onco-metabolic targets is fatty acid synthase (FASN), the terminal enzyme in the de novo synthesis of saturated long-chain fatty acids (FAs). FASN is overexpressed in a wide variety of malignancies including ovarian cancer (OC). It is associated with malignant transformation, progression, drug resistance and poor prognosis^1–3^. Regulation of FASN expression and lipid biosynthesis has been studied in detail in cancer, and a variety of compounds have been developed that directly interfere with FASN enzyme activity and block de novo FA synthesis. Disabling a key metabolic enzyme naturally causes serious disturbance of the metabolic balance and the homeostatic equilibrium of the cells, leading to energy crisis, growth arrest and/or cell death^4,5^. Treatment with FASN inhibitors is therefore necessarily associated with severe metabolic aberrations in the cancer cells. However, it is still unclear whether these metabolic changes are the primary cause or just a secondary consequence of the cytotoxic action of FASN blocking drugs.

To address this, we exposed clear cell (SKOV3) and serous (OVCAR3) ovarian cancer cells, two major histological subtypes of OC, to the FASN selective inhibitor G28UCM^6,7^. We compared the metabolomes of SKOV3 and OVCAR3 cells in the presence or absence of G28UCM with the corresponding proteomes and kinomes. Based on this multi-omics approach and the establishment of ‘SKOV3/OVCAR3 Matching Scores’, we were able to show that the anticancer effect of the FASN inhibitor is mainly due to damage to the lipid bilayer and blockade of lipid signalling, and only secondarily to a deterioration of the central cell metabolism. Thus metabolic disruption in response to FASN blockade is only a distal secondary consequence of the more proximal primary depletion of cellular lipid compartments. Deterioration of lipid membranes appears as the causative primary anticancer event, whereas metabolic perturbation seems to be only a consequence thereof.

## Results

### Both cell lines are equally sensitive to low doses of G28UCM, but differentially sensitive to higher doses

**Figure 1:**
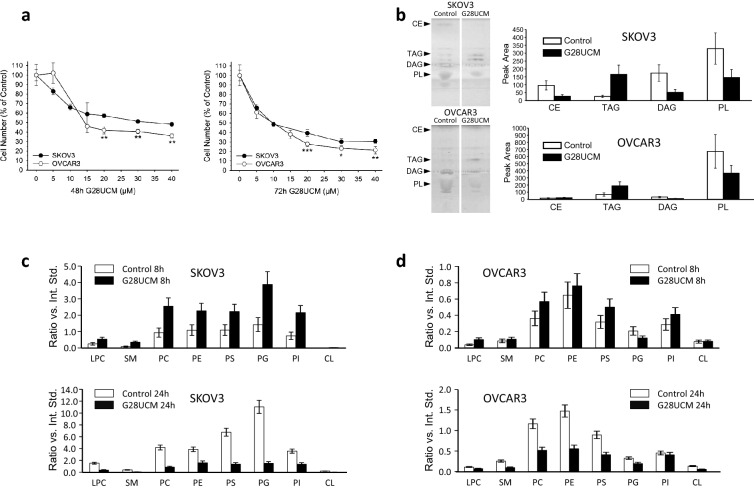
Effects of FASN inhibitor G28UCM on growth and lipid content of SKOV3 and OVCAR3 cells. (**a**) The bipartite pattern of relative growth sensitivity of SKOV3 and OVCAR3 cells against G28UCM. Cells were exposed for 48 h and 72 h to various concentrations of the drug prior to cell growth measurement using a formazan dye assay. At low doses both cell lines are equally sensitive, while at doses ≥ 20 µM, OVCAR3 appears more sensitive than SKOV3. Means ± SD, n = 3. Two-tailed Student's t-test, p < 0.05 (*), p < 0.01 (**) or p < 0.001 (***) between SKOV3 and OVCAR3 treated cells. (**b**) TLC separation and changes of the major cellular lipid classes in SKOV3 and OVCAR3 cells upon G28UCM treatment (20 µM, 72 h). Relative changes of the different phospholipid classes in (**c**) SKOV3 and (**d**) OVCAR3 treated with 0.1% DMSO (Control) or 40 µM G28UCM for 8 h and 24 h. Values are sums of signal intensity of lipid species relative to class specific internal standards added to the samples before analysis (Ratio vs. Int. Std.) (Supplemental Figure S2). *CE* cholesterol esters, *CL* cardiolipin, *DAG* diacylglycerols, *LPC* lysophosphatidylcholine, *PC* phosphatidylcholine, *PE* phosphatidylethanolamine, *PG* phosphatidylglycerol, *PI* phosphatidylinositol, *PL* phospholipids, *PS* phosphatidylserine, *SM* sphingomyelin, *TAG* triacylglycerols.

SKOV3 and OVCAR3 cells were exposed for 48 or 72 h to various concentrations of the FASN inhibitor G28UCM before cell numbers were determined. Figure 1a demonstrates that G28UCM inhibited the growth in both cell lines in a dose-dependent manner. Consistent with our previous studies^8,9^, after 72 h drug exposure the IC_50_ value in each cell line was in the low μM range, demonstrating that both are highly sensitive to FASN inhibition. Nevertheless, at higher concentrations, the drug-resistant cell fraction was significantly larger in SKOV3 than in OVCAR3 cells (Fig. 1a, Supplemental Figures S1a,b).

### G28UCM causes accumulation of storage lipids and depletion of membrane lipids in both cell lines equally

Thin-layer chromatography (TLC) of control and G28UCM-treated cell cultures revealed a typical shift in main cellular lipid classes, with cholesterol esters (CE), diacylglycerols (DAG) and phospholipids (PL) decreasing, while triacylglycerols (TAG) increased (Fig. 1b). This corroborates our previous results^8^ indicating rearrangement from structural membrane lipids (PL) and signalling lipids (DAG) to energy storage lipids (TAG) as a primary consequence of FASN-inhibition apart from general reduction of the total amount of lipids/cell (Supplemental Fig. S1a,b).

For a more detailed analysis of the changes of the individual PL classes the lipid extracts were subjected to MALDI-MS in positive and negative ionization mode using PL class specific internal standards for relative quantification (Supplemental Fig. S2). The protocol follows methods that have already been validated during previous experiments using different types of biological samples including cancer cells^8,9^. Experiments were performed on individual PL-species in order to assign them to the different PL-classes, and signal intensity ratios to the corresponding internal standard were calculated (see Material and Methods). The obtained values were summed up to provide a quantitative measure of each PL class. For testing the reproducibility of lipid analysis by MALDI-MS multiple extracts of the same cell culture were analysed. Results showed a variability in the range of 10–33% in the relative abundance of individual PL classes (Supplemental Fig. S3). This was in good agreement with a cross-validation by liquid chromatography (LC) electrospray ionization (ESI) tandem mass spectrometry (MS/MS) as reference method. Data showed a variability of 6–31% for biological replicates and 4–9% for technical replicates (Supplemental Table S1). As shown in Fig. 1c,d, a typical pattern was observed, which is characterized by an initial increase in lipid species after 8 h and a sharp decrease after 24 h of G28UCM treatment (relative to DMSO), with the changes in SKOV3 being more pronounced than in OVCAR3 cells.

### G28UCM causes accumulation of polyunsaturated fatty acids (PUFAs) in both cell lines equally

**Figure 2:**
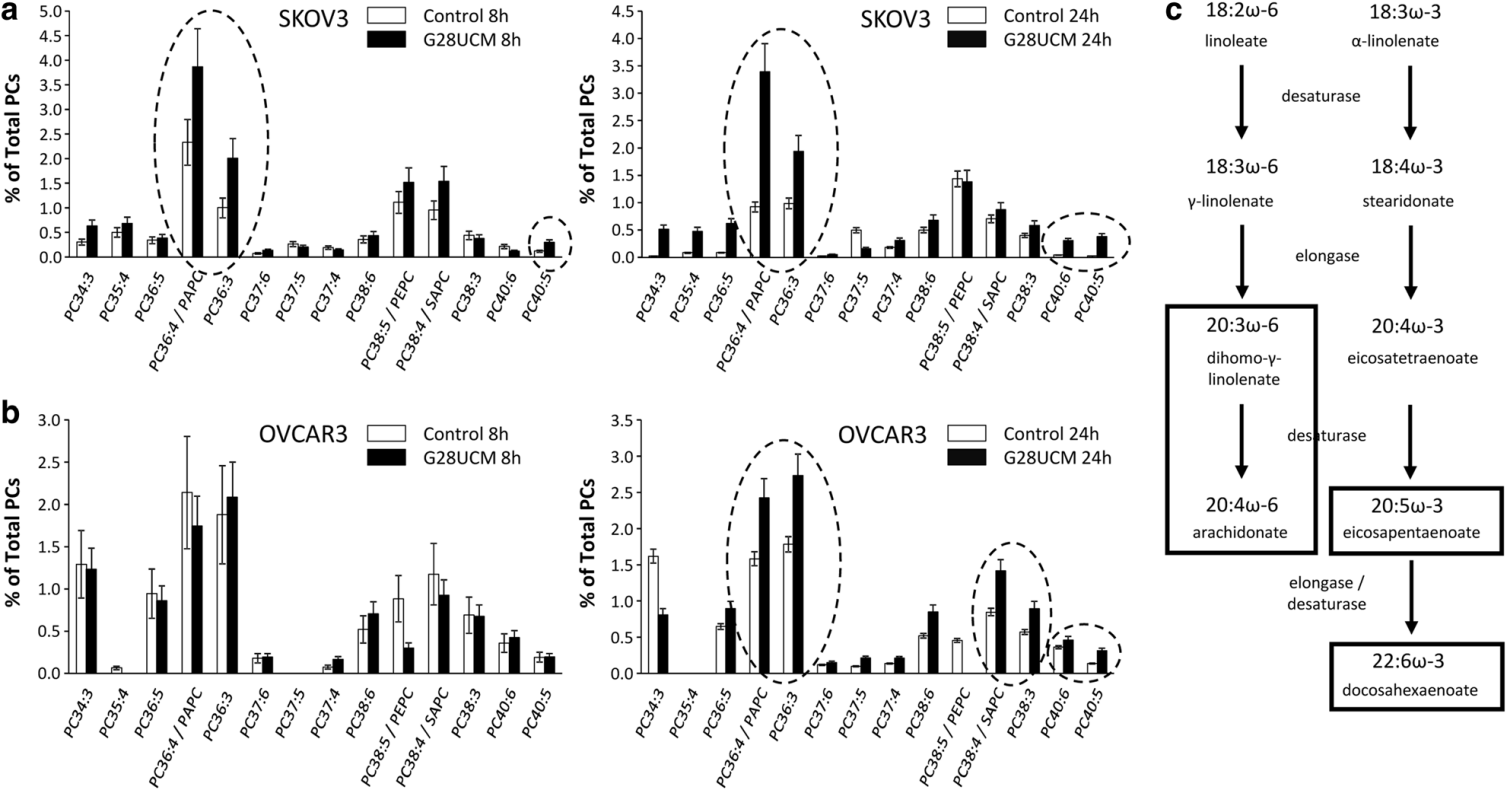
Effects of the FASN inhibitor G28UCM on the phosphatidylcholine (PC) composition of SKOV3 and OVCAR3 cells. Changes in the relative composition of PC species containing PUFAs with > 2 total double bonds (DBs) in (**a**) SKOV3 and (**b**) OVCAR3 cells treated with 0.1% DMSO and 40 µM G28UCM for 8 h and 24 h. Displayed is the relative composition of PC species with > 2 DBs in % of total PC (dashed lines). Values are means ± SD (n = 3). Dashed lines indicate the PC species mostly affected by FASN-inhibition. Letter code of the PUFAs: A, arachidonate (20:4); E, eicosapentaenoate (20:5); P, palmitate (16:0); S, stearate (18:0). (c) Schematic view of the major biosynthesis pathways of very long-chain polyunsaturated FAs (PUFAs) derived from essential ω-3 and ω-6 FAs. Boxes indicate those PUFAs, which were found to be mostly affected by FASN-inhibition.

A MALDI-MS based lipidomics analysis was used to monitor the changes in phosphatidylcholines (PC), which make up the majority of membrane glycerophospholipids. Around 30 individual PC species were detected containing FA residues with 0–6 double bonds (DBs). The composition of PC with 0–2 DBs, which contain palmitate (16:0) and oleate (18:1), were unchanged upon G28UCM treatment (Supplemental Fig. S4). In contrast, marked changes were observed in PC species that are composed of polyunsaturated FA (PUFAs) with > 2 DBs (Fig. 2a,b). In particular, arachidonate (20:4), eicosapentaenoate (20:5) and docosahexaenoate (22:6) were increased in the G28UCM-exposed cells. These very long-chain PUFAs are synthesized from linoleate (18:2) and linolenate (18:3) via the action of desaturases/elongases (Fig. 2c)^10^. Enrichment of PUFAs occurred earlier and was more pronounced in SKOV3 than in OVCAR3 (Fig. 2a,b). Overall, we believe that the rapid quantitative and qualitative changes in membrane lipids in SKOV3 are related to the higher drug resistance of these cells compared to OVCAR3 and could be an adaptive response to the drug effects.

### Striking differences in G28UCM-induced metabolomic patterns between the two cell lines

Using MS with multiple reaction monitoring, we observed that an 8-h exposure to G28UCM did not alter the metabolite levels in SKOV3, but increased approximately half of the glycerophospholipids and almost all sphingolipids in OVCAR3. After 24 h, however, all metabolites in both cell lines were markedly downregulated compared to 8 h of treatment. Remarkably, this decrease between short-term and long-term drug exposure was the only striking analogy in the metabolomic patterns of the two cell lines (Table 1).

**Table 1:**
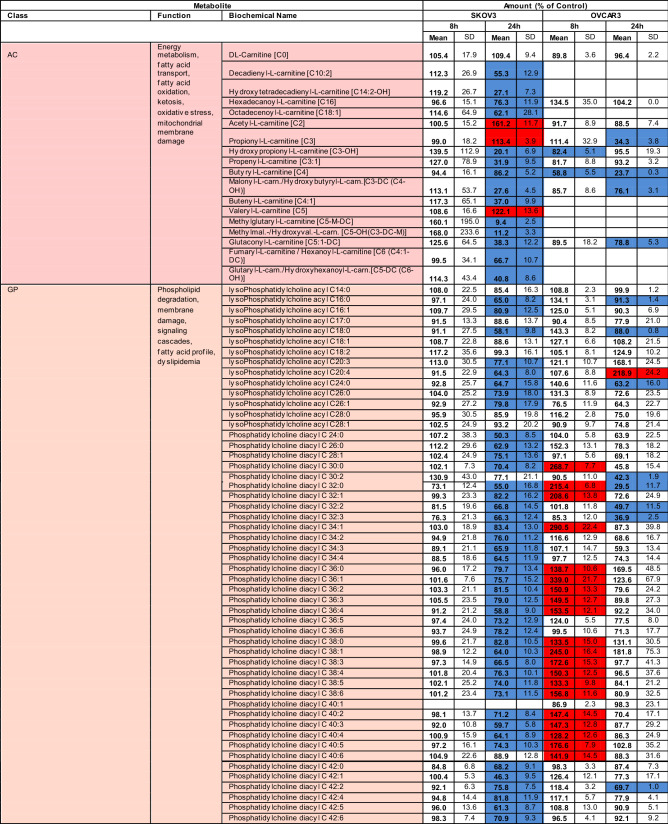

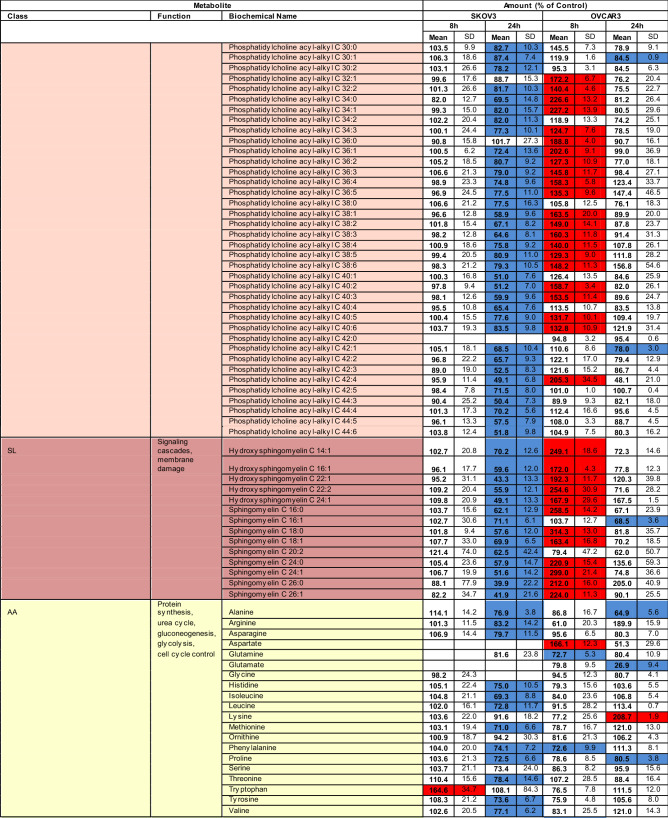

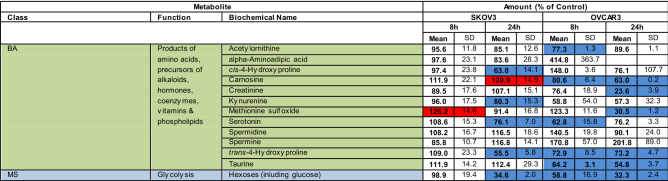
Targeted metabolomic analysis with multiple reaction monitoring mass spectrometry of SKOV3 and OVCAR3 cells exposed for various times to 40 µM G28UCM.

*AC* acylcarnitines, *GP* glycerophospholipids, *SL* sphingolipids (sphingomyelins), *AA* amino acids, *BA* biogenic amines, *MS* monosaccharides.

Blue: significantly (p < 0.05) down-regulated.

Red: significantly (p < 0.05) up-regulated.

In particular, 14 of 17 acylcarnitines were significantly downregulated in SKOV3 after 24 h, whereas in OVCAR3 only 5 were significantly reduced at 8 and/or 24 h. Interestingly, expression of carnitine was unaffected by the drug. This amino acid derivative associates with fatty acyl residues and transfers them to the mitochondria for subsequent beta-oxidation. This suggests that the depletion of acylcarnitines is due to the loss of FAs rather than downregulation of carnitine (Table 1).

Furthermore, after 8 h of drug exposure, 42 of the 90 detectable (glycero)phospholipids and 12 of 14 sphingolipids were significantly upregulated in OVCAR3, but not in SKOV3 (Table 1). Despite this transient lipid upregulation in OVCAR3, a general downregulation of all (glycero)phospholipids and sphingolipids was observed in both cell lines after 24 h of treatment compared to 8 h. For example, in SKOV3, after 8 h of treatment, the overall mean ± SD of all (glycero)phospholipids and sphingolipids was 99 ± 21% of Control and after 24 h 70 ± 12% of Control; while in OVCAR3 the overall mean ± SD was 148 ± 11% of Control after 8 h and 91 ± 22% of Control after 24 h. Thus there was a decline in lipid levels between 8 and 24 h of drug exposure. This is consistent with decreased levels of membrane phospholipids (including LPC, PC and others) and signalling lipids (e.g. DAG) as shown by TLC (Fig. 1b) and MS-based lipidomics (Fig. 1c,d) after > 8 h of treatment—especially in SKOV3 cells. Together, our data expand previous findings from us and others demonstrating membrane remodelling and impairment of signal transduction when FASN is targeted^9,11^.

In addition, metabolomic analysis revealed that FASN inhibitor treatment diminished the content of some proteinogenic amino acids and their products, the biogenic amines, in both cell lines, which complements previous reports demonstrating downregulation of pathways associated with amino acid and protein translation upon blockade of FASN^3,4,9,12,13^. Finally, it was found that the cellular levels of hexoses (including glucose) were reduced to 30–60% after treatment (Table 1).

Overall, apart from the fact that exposure to the FASN inhibitor resulted in a general decrease in cellular FA content, the response pattern of the other metabolites was very different in the two OC cell lines. This was somewhat unexpected, considering the fact that both cell lines were effectively growth-inhibited by G28UCM within 48 h of drug exposure (Fig. 1a).

### Proteomics identifies matching and non-matching effects of G28UCM in the two cell lines

MS/MS shotgun proteomic analysis of OC cells exposed for 8 or 24 h to G28UCM revealed drastic alterations in the proteome. Supplemental Tables S2a,b summarize all significantly (p < 0.05) up- or downregulated proteins in SKOV3 and OVCAR3 cells, respectively.

The UniProt Accession Numbers of these proteins were uploaded to the DAVID platform and were analysed with the BioCarta and KEGG databases using the Functional Annotation Chart Tool. Obtained results are described below and summarized in Table 2 (for all details see Supplemental Table S3). They provide a concise overview on the effects of the FASN inhibitor on major functional processes and their associated sub-processes in the OC cells.

**Table 2:**
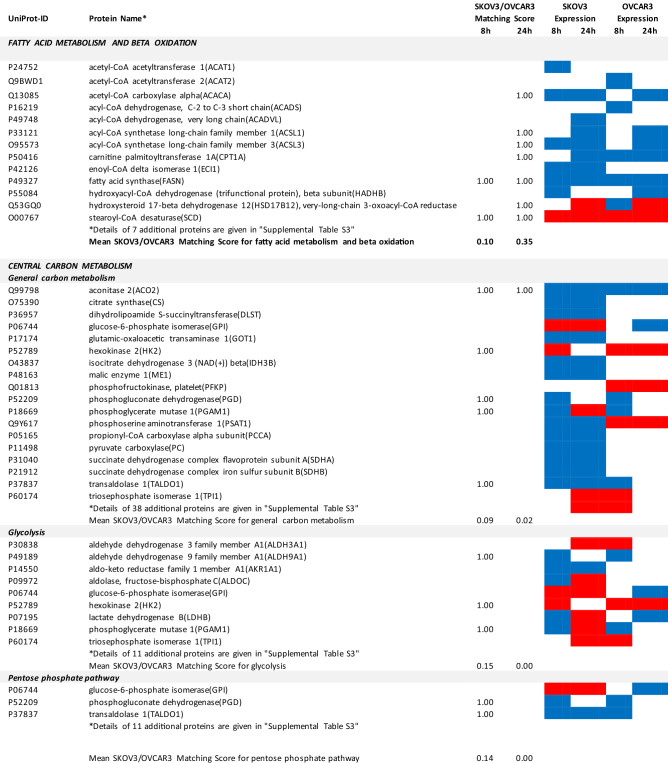

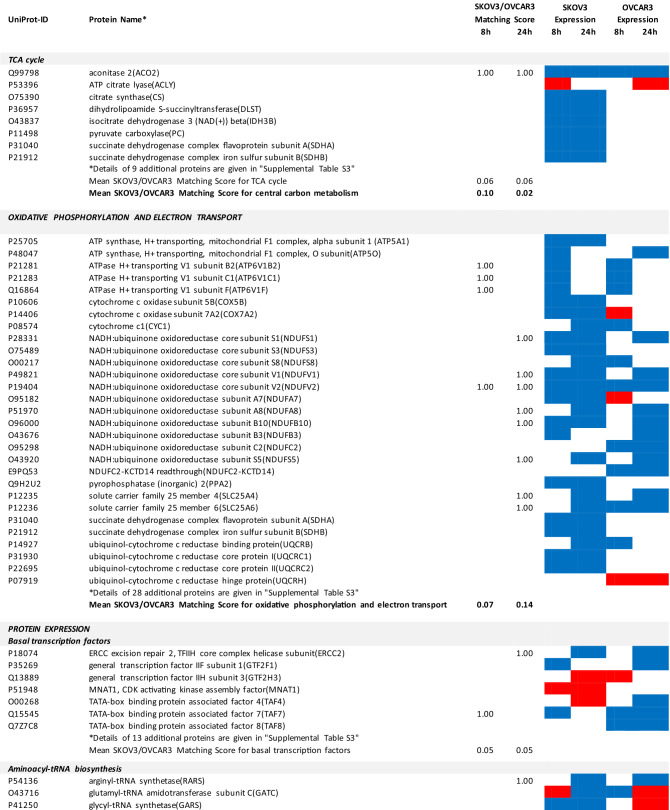

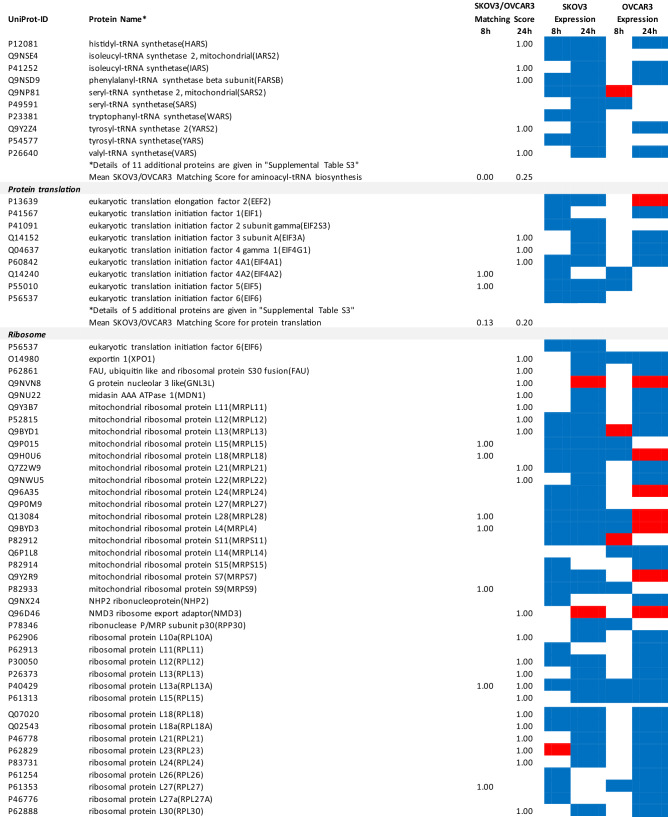

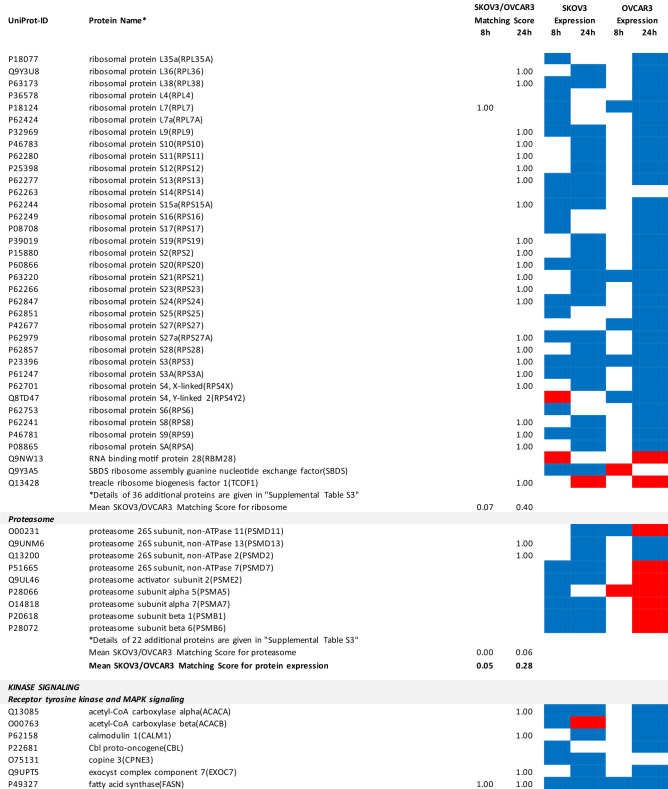

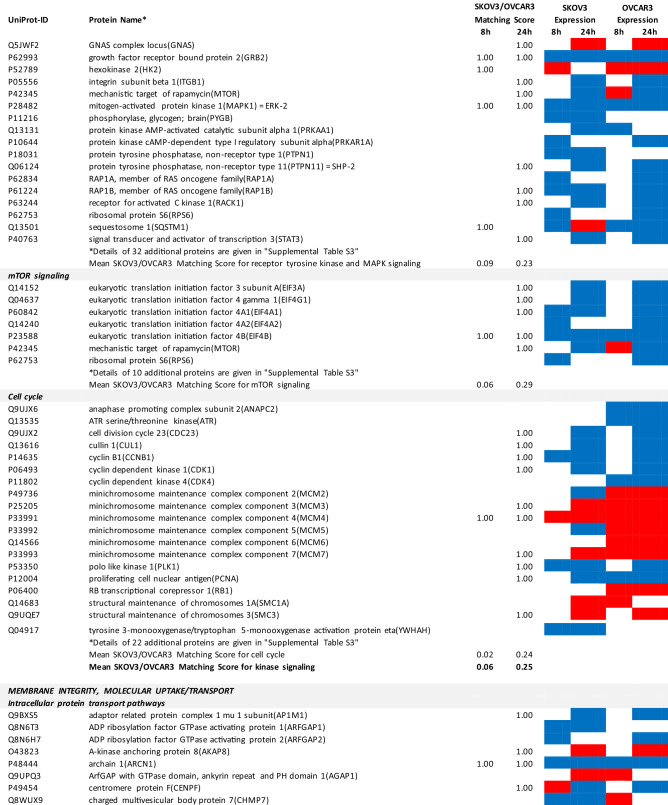

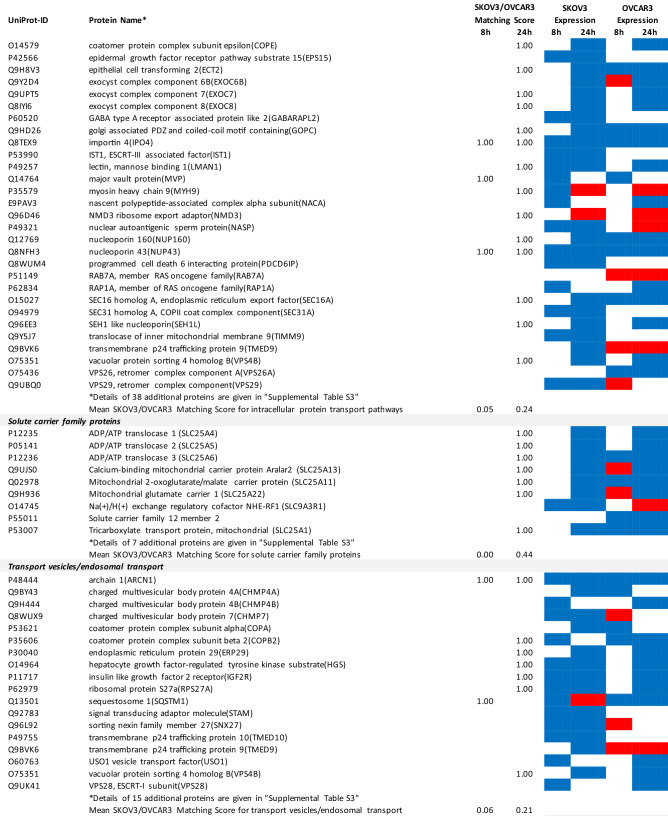

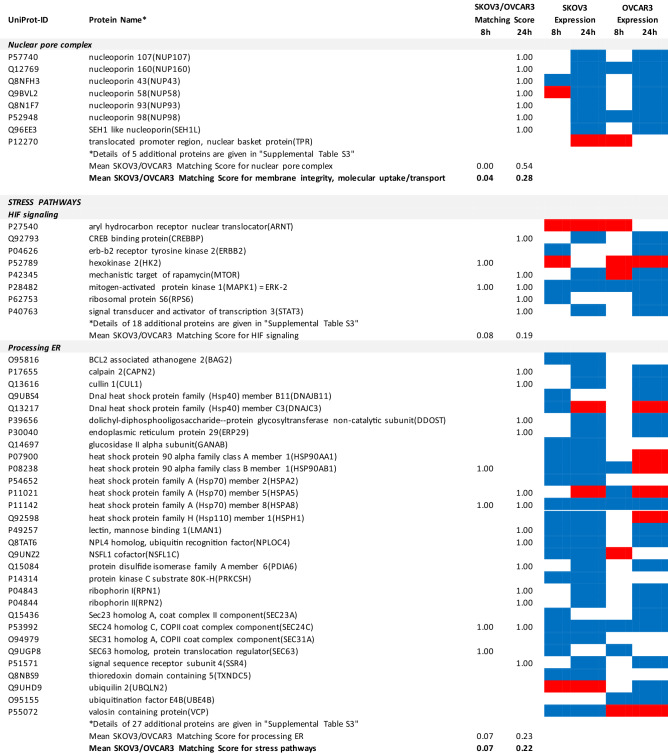

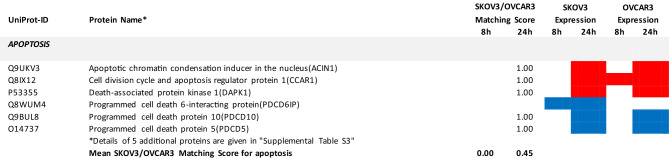
DAVID-assisted shotgun proteomic analysis of key cell processes in SKOV3 and OVCAR3 cells exposed to 40 µM G28UCM using BioCarta and KEGG databases.

For Individual Proteins: SKOV3/OVCAR3 Matching Score: 1,00: uniform regulation at the same time in both cell lines; 0,00: no uniform regulation in both cell lines at the specific time.

For Key Cell Processes or Sub-Processes: Mean SKOV3/OVCAR3 Matching Score: mean of 'Individual Protein SKOV3/OVCAR3 Matching Scores'.

Significantly (p < 0.05) downregulated proteins: Blue cell.

Significantly (p < 0.05) upregulated proteins: Red cell.

#### G28UCM blocks FA synthesis, FA activation and FA degradation, but stimulates FA elongation and FA desaturation in both cell lines equally

In both cell lines, synthesis of FAs and of ketone bodies was impaired due to reduced expression of FASN, acetyl-CoA acetyltransferases (ACAT1, ACAT2) and acetyl-CoA-carboxylase alpha (ACACA). Activation and degradation of FAs was also reduced due to downregulation of acyl-CoA synthetase, carnitine palmitoyltransferase and of several lipolytic enzymes. In contrast, upregulation of very-long-chain 3-oxoacyl-CoA reductase and of stearoyl-CoA desaturase (SCD), the key fatty acid desaturase (Table 2 and Supplemental Table S3), indicates stimulated formation of unsaturated FAs palmitoleate (16:1) and oleate (18:1), which are key building blocks for the PL that are components of cell membranes^14^.

#### G28UCM affects central carbon metabolism, oxidative phosphorylation (OXPHOS) and electron transport more intensely in SKOV3 than in OVCAR3 cells

The enzymes of central carbon metabolism (general carbon metabolism, glycolysis, pentose phosphate pathway, tricarboxylic acid cycle) were more vigorously modulated in SKOV3 than in OVCAR3 cells (Table 2 and Supplemental Table S3).

For example, acetyl-CoA—a central metabolic intermediate that is required for energy production, lipid synthesis and epigenetic gene regulation^3^—is converted from free acetate by the catalytic action of acyl-CoA synthetase short-chain family member 2 (ACSS2)^15^. This enzyme was found to be downregulated and thus less available for synthesis of acetyl-CoA in SKOV3 cells (Supplemental Table S3).

In contrast, the reduced content of L-valine in G28UCM-treated SKOV3 cells as shown in Table 1 was probably in part due to a G28UCM-mediated sevenfold upregulation of hydroxyisobutyryl-CoA hydrolase (HIBCH), which is involved in the degradation of this proteinogenic amino acid^16^. In addition, downregulation of a number of amino acid-producing enzymes was seen (Supplemental Tables S2a and S3), which correlates with drug-mediated depletion of amino acids as shown in Table 1. Together, this suggests that the amino acid turnover was impaired in G28UCM-treated SKOV3 cells.

Some glycolytic enzymes were lowered in SKOV3 cells after 8 h, but then increased after 24 h treatment. Overall, drug-mediated regulation of glycolytic enzymes was quite complex and was more pronounced in SKOV3 than in OVCAR3 cells (Table 2 and Supplemental Table S3).

In addition, many enzymes of the pentose phosphate pathway and the TCA cycle, which are closely linked to glycolysis^17^, were temporarily or stably suppressed during the observation period in SKOV3, but not in OVCAR3 (Table 2 and Supplemental Table S3).

Similarly, OXPHOS and electron transport were severely impaired in SKOV3, while inhibition in OVCAR3 was mainly restricted to Complex I (Table 2 and Supplemental Table S3).

#### G28UCM affects protein synthesis and cell growth signalling in both cell lines similarly

Exposure to G28UCM was found to downregulate key components of the gene expression machineries including transcription factor complexes TFIID, TFIIE, TFIIF and/or TFIIH, many aminoacyl-tRNAs of the canonical amino acids, eukaryotic translation initiation factors (EIF1–EIF6), and ribosomal proteins. In addition, G28UCM reduced expression of the PI3K downstream effectors mTOR, RPS6 and the eukaryotic translation initiation factors EIF3a, EIF4a, EIF4b and EIF4G in both cell lines, and upregulated PI3K/mTOR-inhibitory phosphatase PPP2cA. Thus, FASN inhibition was silencing the PI3K pathway, which is known to modulate cell metabolism, energy balance and ribosomal protein synthesis at the endoplasmic reticulum (ER)^18^. In addition, we observed that G28UCM treatment affected the composition of the proteasomes (Table 2 and Supplemental Table S3). Taken together, our data confirm previous reports, which showed that FASN inhibition interferes with protein synthesis and promotes protein degradation, resulting in lower protein steady state levels^4,9,13^. Moreover, insulin-, epidermal- and hepatocyte-growth factor receptors and their downstream effectors (CBL, CRK, EGFR, ERBB2, GRB2, MAPKs, RAS family members), as well as STAT3, which regulate carbohydrate utilization, growth, migration and invasion^19,20^, were downregulated by G28UCM in SKOV3 and/or OVCAR3 (Table 2 and Supplemental Table S3).

#### G28UCM impedes the molecular transport machinery in both cell lines equally

Furthermore, intracellular transport proteins, solute carrier family proteins, and coatomer protein complexes, which mediate the transport of newly synthesized proteins from the ER, via the Golgi to the trans Golgi network^21,22^, were significantly downregulated by G28UCM. Of note, a number of charged multivesicular bodies (MVB) were also significantly decreased. They are probably involved in sorting of endosomal cargo and enable degradation of membrane proteins and lipids via lysosomes^23^. In addition, components of the nuclear pore complex (nucleoporins) were markedly downregulated indicating impaired nucleocytoplasmic shuttling^24^. Altogether, the proteomic patterns of these processes revealed high congruence between both cell lines and demonstrate that molecular trafficking was heavily impeded in treated SKOV3 and OVCAR3 cells (Table 2 and Supplemental Table S3).

#### G28UCM activates stress pathways and apoptosis in both cell lines equally

We observed that HIF-1 alpha and HIF-1 beta (aryl hydrocarbon receptor nuclear translocator [ARNT]) were upregulated in both cell lines. This indicates drug-induced increase in environmental and/or energetic stress, which may be aggravated by rearrangement of membrane PL and downregulation of ER proteins in both cell lines (Table 2 and Supplemental Table S3) leading to induction of cell death. It is thus not surprising that all these changes strongly activate the apoptotic pathways. Accordingly, many pro-apoptotic effectors were upregulated, while apoptosis antagonists were diminished in both cell lines (Table 2 and Supplemental Table S3).

### Kinomics reveals a high degree of concordance in signalling and stress response in both cell lines

Protein levels determined by MS/MS shotgun proteomics were correlated with protein phosphorylation obtained from antibody microarrays with subsequent analysis in the DAVID platform using the KEGG database. Accordingly, FASN inhibition was found to affect the phosphorylation of proteins involved in functional systems (FS) that control ‘Molecular and Cellular Interaction’ (FS1) and ‘Stress Responses Due to Derangements’ (FS2) in both cell lines (Tables 3 and 4). Specifically, G28UCM strongly impaired intracellular signalling networks in SKOV3 and OVCAR3, including downregulation of phosphorylated variants of CaMK1d, CaMKK1 (Ca^2+^ signalling), CLK1 (RNA splicing), CSF1R, MAPK/ERK, Gab1 and JUN (receptor signalling). Moreover, we observed very strong downregulation (≤ 15% of Control) of phosphorylated forms of B-MYB, CDK5, CFL1 (cytoskeleton), CSK (SRC signalling), ENFB2 (ephrin signalling) and GFAP in drug-exposed SKOV3, and of eIF4b (translation), MEK2 (signalling), MYC, p53 and SMAD1 (transcription) in treated OVCAR3 (for details see Supplemental Table S4a,b).

**Table 3:** DAVID-assisted antibody microarray kinomic analysis of KEGG pathways in SKOV3 cells exposed for 24 h to 40 µM G28UCM.

Functional system (FS)	Major cell functional group	Affected KEGG pathways	Number of Phosphorylated Proteins
FS1 molecular and cellular interaction	Signal transduction and endocrine system	hsa04010:MAPK signaling pathway	18
		hsa04012:ErbB signaling pathway	15
		hsa04014:Ras signaling pathway	12
		hsa04015:Rap1 signaling pathway	14
		*hsa04020:Calcium signaling pathway*	*6*
		***hsa04022:cGMP-PKG signaling pathway***	***5***
		***hsa04024:cAMP signaling pathway***	***8***
		*hsa04064:NF-kappa B signaling pathway*	*7*
		hsa04066:HIF-1 signaling pathway	9
		hsa04068:FoxO signaling pathway	7
		*hsa04070:Phosphatidylinositol signaling system*	*4*
		*hsa04071:Sphingolipid signaling pathway*	*11*
		*hsa04150:mTOR signaling pathway*	*7*
		hsa04151:PI3K-Akt signaling pathway	17
		***hsa04310:Wnt signaling pathway***	***7***
		hsa04370:VEGF signaling pathway	7
		hsa04668:TNF signaling pathway	8
		hsa04910:Insulin signaling pathway	8
		hsa04912:GnRH signaling pathway	7
		***hsa04914:Progesterone-mediated oocyte maturation***	***4***
		hsa04915:Estrogen signaling pathway	7
		hsa04916:Melanogenesis	5
		*hsa04917:Prolactin signaling pathway*	*4*
		hsa04919:Thyroid hormone signaling pathway	9
		*hsa04920:Adipocytokine signaling pathway*	*4*
		hsa04921:Oxytocin signaling pathway	12
		*hsa04925:Aldosterone synthesis and secretion*	*4*
	Cell growth and death	*hsa04110:Cell cycle*	*7*
		*hsa04114:Oocyte meiosis*	*6*
		*hsa04115:p53 signaling pathway*	*5*
	Transport and catabolism Cellular community	***hsa04144:Endocytosis***	***9***
		hsa04510:Focal adhesion	17
		hsa04520:Adherens junction	8
		hsa04540:Gap junction	9
		hsa04810:Regulation of actin cytoskeleton	9
	Circulatory system development	*hsa04270:Vascular smooth muscle contraction*	*7*
		***hsa04320:Dorso-ventral axis formation***	***3***
		***hsa04360:Axon guidance***	***8***
		hsa04380:Osteoclast differentiation	10
	Excretory system	*hsa04960:Aldosterone-regulated sodium reabsorption*	*3*
		*hsa04961:Endocrine and other factor-regulated calcium reabsorption*	*3*
		Sum of affected KEGG pathways: 41	330
FS2 stress response (due to derangement)	Endocrine and metabolic diseases nervous system and neurodegenerative diseases	*hsa04930:Type II diabetes mellitus*	*3*
		*hsa04931:Insulin resistance*	*8*
		*hsa04713:Circadian entrainment*	*4*
		hsa04720:Long-term potentiation	6
		hsa04722:Neurotrophin signaling pathway	13
		*hsa04723:Retrograde endocannabinoid signaling*	*4*
		*hsa04725:Cholinergic synapse*	*5*
		*hsa04726:Serotonergic synapse*	*5*
		*hsa04730:Long-term depression*	*4*
		*hsa04750:Inflammatory mediator regulation of TRP channels*	*5*
		***hsa05010:Alzheimer's disease***	***5***
		***hsa05014:Amyotrophic lateral sclerosis (ALS)***	***3***
		*hsa05020:Prion diseases*	*3*
	Substance dependence	***hsa05030:Cocaine addiction***	***5***
		***hsa05031:Amphetamine addiction***	***5***
		***hsa05034:Alcoholism***	***9***
	Infectious diseases	hsa05100:Bacterial invasion of epithelial cells	11
		hsa05120:Epithelial cell signaling in Helicobacter pylori infection	6
		***hsa05130:Pathogenic Escherichia coli infection***	***5***
		hsa05131:Shigellosis	7
		***hsa05132:Salmonella infection***	***4***
		***hsa05133:Pertussis***	***4***
		hsa05140:Leishmaniasis	6
		*hsa05142:Chagas disease (American trypanosomiasis)*	*5*
		*hsa05146:Amoebiasis*	*4*
		*hsa05160:Hepatitis C*	*6*
		hsa05161:Hepatitis B	12
		*hsa05162:Measles*	*6*
		hsa05164:Influenza A	7
		*hsa05166:HTLV-I infection*	*11*
		*hsa05168:Herpes simplex infection*	*7*
		*hsa05169:Epstein-Barr virus infection*	*8*
		*hsa05416:Viral myocarditis*	*4*
	Cancers	hsa05200:Pathways in cancer	23
		*hsa05202:Transcriptional misregulation in cancer*	*6*
		hsa05203:Viral carcinogenesis	15
		hsa05205:Proteoglycans in cancer	19
		hsa05206:MicroRNAs in cancer	16
		hsa05210:Colorectal cancer	6
		***hsa05211:Renal cell carcinoma***	***5***
		hsa05212:Pancreatic cancer	6
		hsa05213:Endometrial cancer	8
		hsa05214:Glioma	10
		hsa05215:Prostate cancer	11
		***hsa05216:Thyroid cancer***	***4***
		hsa05218:Melanoma	8
		hsa05219:Bladder cancer	7
		hsa05220:Chronic myeloid leukemia	10
		*hsa05221:Acute myeloid leukemia*	*4*
		*hsa05222:Small cell lung cancer*	*6*
		hsa05223:Non-small cell lung cancer	6
		hsa05230:Central carbon metabolism in cancer	6
		hsa05231:Choline metabolism in cancer	9
	Immune system and disease	hsa04062:Chemokine signaling pathway	10
		*hsa04611:Platelet activation*	*6*
		*hsa04620:Toll-like receptor signaling pathway*	*5*
		*hsa04650:Natural killer cell mediated cytotoxicity*	*7*
		hsa04660:T cell receptor signaling pathway	8
		hsa04662:B cell receptor signaling pathway	6
		*hsa04664:Fc epsilon RI signaling pathway*	*5*
		hsa04666:Fc gamma R-mediated phagocytosis	9
		*hsa04670:Leukocyte transendothelial migration*	*7*
		Sum of affected KEGG pathways: 62	448

Italics font: pathways with up-regulated phosphoproteins (> 150% of Control).

Bold italics font: pathways with down-regulated phosphoproteins (< 50% of Control).

Regular font: pathways with up- and down-regulated phosphoproteins.

**Table 4:** DAVID-assisted antibody microarray kinomic analysis of KEGG pathways in OVCAR3 cells exposed for 24 h to 40 µM G28UCM.

Functional system (FS)	Major Cell Functional Group	Affected KEGG Pathways	Number of Phosphorylated Proteins
FS1 molecular/cellular interaction	Signal transduction and endocrine system	hsa04010:MAPK signaling pathway	22
		hsa04012:ErbB signaling pathway	20
		hsa04014:Ras signaling pathway	21
		hsa04015:Rap1 signaling pathway	17
		*hsa04020:Calcium signaling pathway*	*6*
		***hsa04022:cGMP-PKG signaling pathway***	***8***
		***hsa04024:cAMP signaling pathway***	***11***
		hsa04064:NF-kappa B signaling pathway	7
		hsa04066:HIF-1 signaling pathway	14
		hsa04068:FoxO signaling pathway	14
		***hsa04071:Sphingolipid signaling pathway***	***10***
		hsa04150:mTOR signaling pathway	9
		hsa04151:PI3K-Akt signaling pathway	25
		***hsa04152:AMPK signaling pathway***	***7***
		***hsa04310:Wnt signaling pathway***	***7***
		***hsa04350:TGF-beta signaling pathway***	***4***
		hsa04370:VEGF signaling pathway	10
		***hsa04630:Jak-STAT signaling pathway***	***6***
		hsa04668:TNF signaling pathway	11
		hsa04910:Insulin signaling pathway	13
		hsa04912:GnRH signaling pathway	11
		hsa04913:Ovarian steroidogenesis	3
		hsa04914:Progesterone-mediated oocyte maturation	10
		hsa04915:Estrogen signaling pathway	9
		hsa04916:Melanogenesis	6
		hsa04917:Prolactin signaling pathway	11
		hsa04919:Thyroid hormone signaling pathway	12
		***hsa04920:Adipocytokine signaling pathway***	***5***
		***hsa04921:Oxytocin signaling pathway***	***8***
		*hsa04922:Glucagon signaling pathway*	*5*
		***hsa04923:Regulation of lipolysis in adipocytes***	***4***
	Cell growth/death	***hsa04110:Cell cycle***	***5***
		***hsa04114:Oocyte meiosis***	***6***
	Cellular community	hsa04510:Focal adhesion	18
		hsa04520:Adherens junction	9
		***hsa04540:Gap junction***	***6***
		***hsa04550:Signaling pathways regulating pluripotency of stem cells***	***12***
		hsa04810:Regulation of actin cytoskeleton	12
	Circulatory system	hsa04261:Adrenergic signaling in cardiomyocytes	7
		***hsa04270:Vascular smooth muscle contraction***	***7***
	Development	***hsa04320:Dorso-ventral axis formation***	***3***
		***hsa04360:Axon guidance***	***7***
		hsa04380:Osteoclast differentiation	15
	Excretory system	***hsa04960:Aldosterone-regulated sodium reabsorption***	***3***
		Sum of affected KEGG pathways: 44	436
FS2 Stress Response (due to derangement)	Endocrine and metabolic diseases	***hsa04930:Type II diabetes mellitus***	***5***
		***hsa04931:Insulin resistance***	***9***
		***hsa04932:Non-alcoholic fatty liver disease (NAFLD)***	***8***
	Nervous system and neurodegenrative diseases	hsa04720:Long-term potentiation	6
		hsa04722:Neurotrophin signaling pathway	20
		hsa04723:Retrograde endocannabinoid signaling	5
		***hsa04725:Cholinergic synapse***	***6***
		hsa04726:Serotonergic synapse	5
		*hsa04728:Dopaminergic synapse*	*7*
		***hsa04730:Long-term depression***	***5***
		*hsa04750:Inflammatory mediator regulation of TRP channels*	*7*
		hsa05014:Amyotrophic lateral sclerosis (ALS)	3
		***hsa05020:Prion diseases***	***5***
	Substance dependence Infectious diseases	hsa05030:Cocaine addiction	4
		hsa05100:Bacterial invasion of epithelial cells	6
		hsa05120:Epithelial cell signaling in Helicobacter pylori infection	11
		hsa05130:Pathogenic Escherichia coli infection	4
		***hsa05131:Shigellosis***	***7***
		***hsa05132:Salmonella infection***	***7***
		***hsa05133:Pertussis***	***6***
		hsa05140:Leishmaniasis	8
		***hsa05142:Chagas disease (American trypanosomiasis)***	***8***
		***hsa05145:Toxoplasmosis***	***9***
		hsa05152:Tuberculosis	11
		hsa05160:Hepatitis C	12
		hsa05161:Hepatitis B	15
		hsa05162:Measles	10
		hsa05164:Influenza A	15
		hsa05166:HTLV-I infection	15
		hsa05168:Herpes simplex infection	9
		hsa05169:Epstein-Barr virus infection	14
	Cancers	hsa05200:Pathways in cancer	28
		hsa05202:Transcriptional misregulation in cancer	10
		*hsa05203:Viral carcinogenesis*	*10*
		hsa05205:Proteoglycans in cancer	27
		hsa05206:MicroRNAs in cancer	14
		***hsa05210:Colorectal cancer***	***9***
		***hsa05211:Renal cell carcinoma***	***10***
		hsa05212:Pancreatic cancer	11
		hsa05213:Endometrial cancer	9
		hsa05214:Glioma	12
		hsa05215:Prostate cancer	13
		***hsa05216:Thyroid cancer***	***7***
		hsa05218:Melanoma	11
		hsa05219:Bladder cancer	9
		hsa05220:Chronic myeloid leukemia	11
		hsa05221:Acute myeloid leukemia	12
		***hsa05222:Small cell lung cancer***	***6***
		hsa05223:Non-small cell lung cancer	11
		hsa05230:Central carbon metabolism in cancer	11
		***hsa05231:Choline metabolism in cancer***	***12***
	Immune system/disease	hsa04062:Chemokine signaling pathway	13
		hsa04611:Platelet activation	9
		***hsa04620:Toll-like receptor signaling pathway***	***11***
		***hsa04621:NOD-like receptor signaling pathway***	***6***
		***hsa04622:RIG-I-like receptor signaling pathway***	***4***
		hsa04650:Natural killer cell mediated cytotoxicity	11
		***hsa04660:T cell receptor signaling pathway***	***14***
		***hsa04662:B cell receptor signaling pathway***	***8***
		hsa04664:Fc epsilon RI signaling pathway	12
		hsa04666:Fc gamma R-mediated phagocytosis	10
		*hsa04670:Leukocyte transendothelial migration*	*7*
		Sum of affected KEGG pathways: 62	610

Italics font: pathways with up-regulated phosphoproteins (> 150% of Control).

Bold italics font: pathways with down-regulated phosphoproteins (< 50% of Control).

Regular font: pathways with up-/down-regulated phosphoproteins.

### The ‘SKOV3/OVCAR3 matching score’: a useful tool to differentiate causal mechanisms of drug action from reactive secondary drug reactions

SKOV3 and OVCAR3 showed comparable growth inhibition to < 20 µM G28UCM after 48 and 72 h treatment. At higher concentrations, however, the surviving fraction was markedly larger in SKOV3 than in OVCAR3 (Fig. 1). Accordingly, drug-treated SKOV3 and OVCAR3 showed distinctly different response patterns for lipids, amino acids, and biogenic amines. The only similarity in the metabolomes of treated SKOV3 and OVCAR cells was an overall decline of metabolite levels between 8 and 24 h of treatment (Table 1). The data suggest that although growth in both cell lines depends on active fatty acid synthesis, the intracellular metabolic pathways yet respond quite differently to inhibition of FASN in these cell lines.

**Figure 3:**
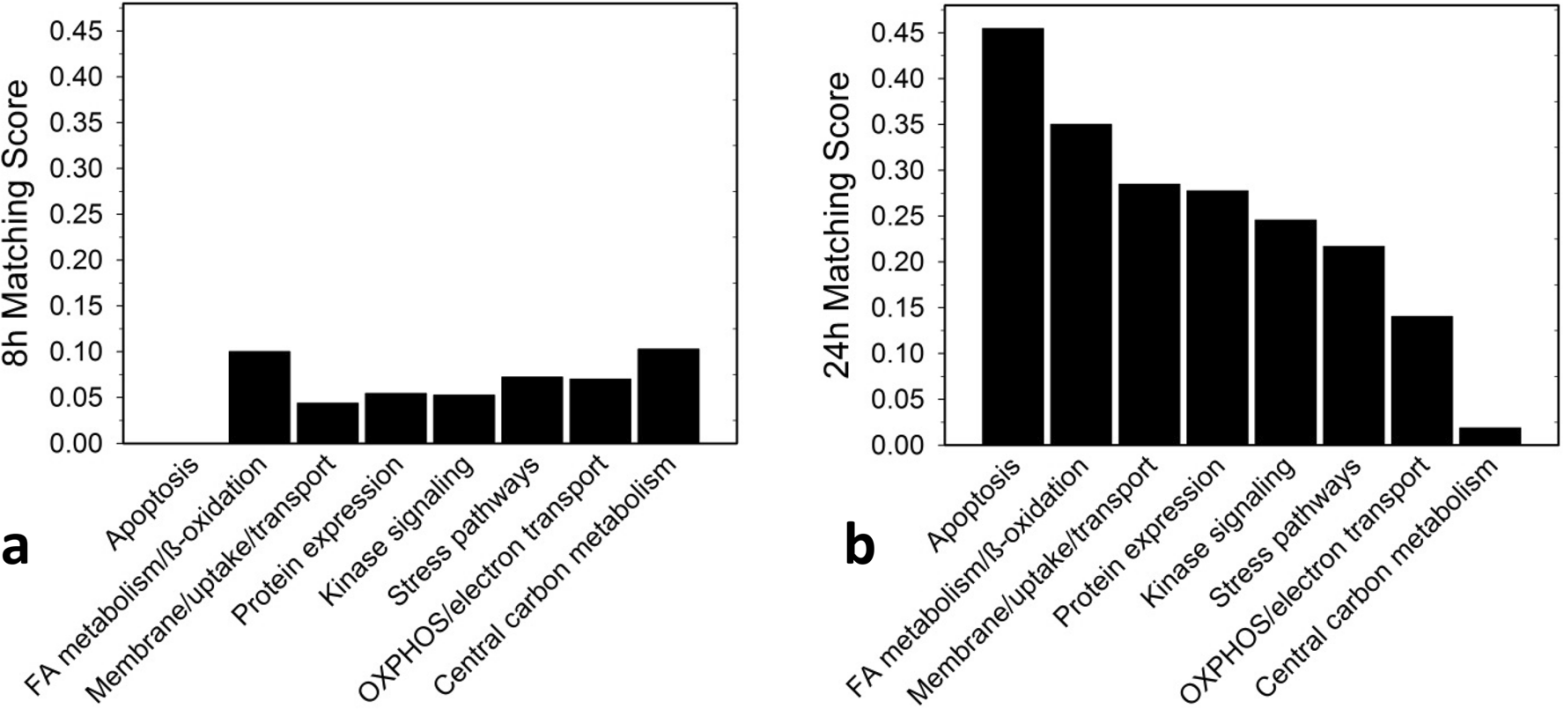
The concordance (‘Matching Score’) of the effects of the FASN inhibitor G28UCM on the stead-state level of proteins that control key cell processes including apoptosis, fatty acid (FA) metabolism/beta-oxidation, membrane maintenance/molecular uptake/transport, protein expression, kinase signalling, stress pathways, oxidative phosphorylation (OXPHOS)/electron transport, and central carbon metabolism after 8 h (**a**) and 24 h (**b**) of drug exposure. For estimation of the SKOV3/OVCAR3 Matching Score see Material and Methods Section and footnotes to Table 2 and Supplemental Table S3.

**Figure 4:**
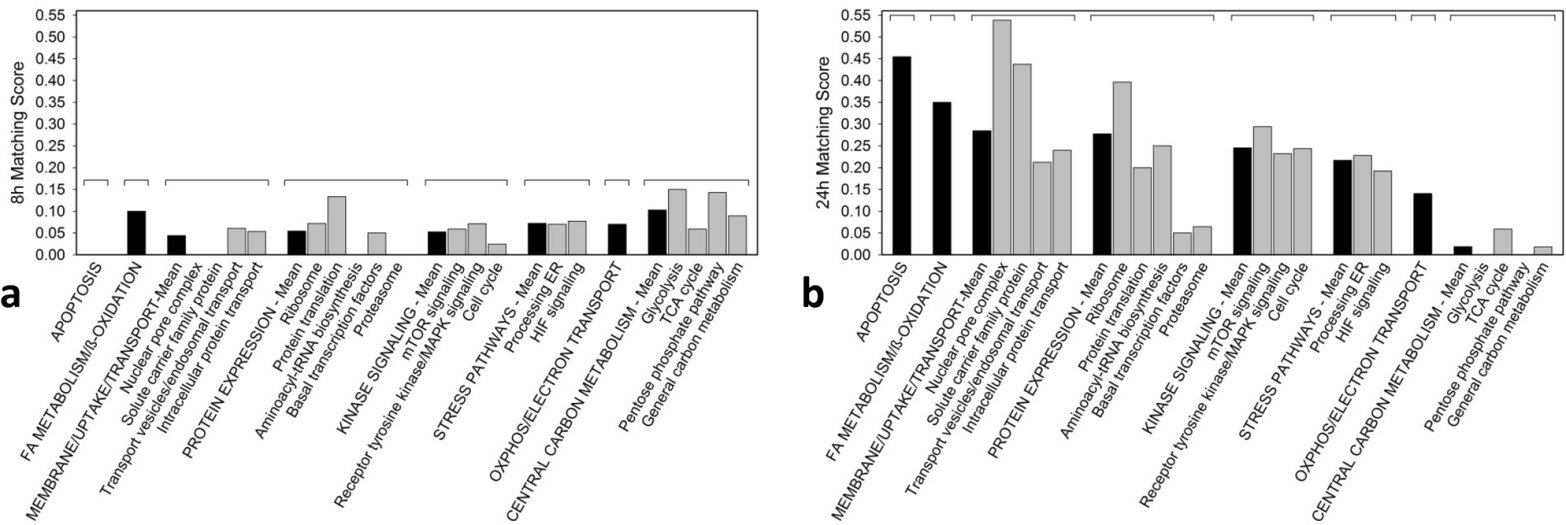
The concordance (‘Matching Score’) of the effects of the FASN inhibitor G28UCM on the stead-state level of proteins that control key cell processes [apoptosis, fatty acid (FA) metabolism/beta-oxidation, membrane maintenance/molecular uptake/transport, protein expression, kinase signalling, stress pathways oxidative phosphorylation (OXPHOS)/electron transport, and central carbon metabolism—black columns] and associated sub-processes (grey columns) after 8 h (**a**) and 24 h (**b**) of drug exposure. For estimation of the SKOV3/OVCAR3 Matching Score see Material and Methods Section and Footnotes to Table 2 and Supplemental Table S3.

Therefore, we wondered if these dichotomous drug sensitivities were reflected in corresponding bipartite response patterns in the proteomes of SKOV3 and OVCAR3 cells. To test this, the drug-mediated modulation of key proteins from crucial intracellular (sub-)processes were compared between SKOV3 and OVCAR3 by estimating to what extent the reactions of each protein and its corresponding (sub-)process match in the two cell lines. For this purpose, the so-called 'SKOV3/OVCAR3 Matching Score' has been introduced and the corresponding results are summarized in Figs. 3 and 4. While after a short-term drug exposure this score is still very low and similar for most processes (Fig. 3a), it increases sharply after 24 h of treatment with the highest matching in apoptosis being followed by FA metabolism/beta-oxidation, membrane integrity/molecular uptake/transport, protein expression, kinase signalling, stress pathways, and OXPHOS/electron transport. Notably, central carbon metabolism had by far the lowest matching score (Fig. 3b). In addition, a more detailed analysis involving a number of secondary sub-processes of the large pathways revealed very little agreement between the two cell lines after 8 h of drug exposure. Interestingly, at this early stage glycolysis and pentose phosphate pathway exhibited the highest scores (Fig. 4a). However, after 24 h, the situation changed dramatically. At this time, the scores in glycolysis, pentose phosphate pathway and general carbon metabolism were close to zero, whereas in apoptosis, nuclear pore complex, solute carrier family proteins and ribosomes highest matching was observed (Fig. 4b). Taken together, after 24 h drug action the central carbon metabolism revealed lowest SKOV3/OVCAR3 Matching Scores. Thus, while both cell lines are sensitive to growth inhibition by the FASN inhibitor, central carbon anabolic and catabolic pathways yet respond essentially different in both cell lines, which argues against a causal role of central carbon metabolism in the mechanism of action of the FASN inhibitor.

Finally, kinomics revealed that G28UCM modulated a total of 92 KEGG pathways concordantly in both cell lines, while only 25 KEGG pathways were independently regulated. This suggests that G28UCM affects many signalling systems consistently in both cell lines (Table 5).

**Table 5:** DAVID-assisted antibody microarray kinomic analysis of KEGG pathways in OC cells exposed for 24 h to 40 µM G28UCM.

SKOV3 (% of Control > 150 or < 50)	OVCAR3 (% of Control > 150 or < 50)	Both	Single
KEGG pathways	KEGG pathways		
hsa04010:MAPK signaling pathway	hsa04010:MAPK signaling pathway	1	
hsa04012:ErbB signaling pathway	hsa04012:ErbB signaling pathway	1	
hsa04014:Ras signaling pathway	hsa04014:Ras signaling pathway	1	
hsa04015:Rap1 signaling pathway	hsa04015:Rap1 signaling pathway	1	
hsa04020:Calcium signaling pathway	hsa04020:Calcium signaling pathway	1	
hsa04022:cGMP-PKG signaling pathway	hsa04022:cGMP-PKG signaling pathway	1	
hsa04024:cAMP signaling pathway	hsa04024:cAMP signaling pathway	1	
hsa04064:NF-kappa B signaling pathway	hsa04064:NF-kappa B signaling pathway	1	
hsa04066:HIF-1 signaling pathway	hsa04066:HIF-1 signaling pathway	1	
hsa04068:FoxO signaling pathway	hsa04068:FoxO signaling pathway	1	
hsa04070:Phosphatidylinositol signaling system			1
hsa04071:Sphingolipid signaling pathway	hsa04071:Sphingolipid signaling pathway	1	
hsa04150:mTOR signaling pathway	hsa04150:mTOR signaling pathway	1	
hsa04151:PI3K-Akt signaling pathway	hsa04151:PI3K-Akt signaling pathway	1	
	hsa04152:AMPK signaling pathway		1
hsa04310:Wnt signaling pathway	hsa04310:Wnt signaling pathway	1	
	hsa04350:TGF-beta signaling pathway		1
hsa04370:VEGF signaling pathway	hsa04370:VEGF signaling pathway	1	
	hsa04630:Jak-STAT signaling pathway		1
hsa04668:TNF signaling pathway	hsa04668:TNF signaling pathway	1	
hsa04910:Insulin signaling pathway	hsa04910:Insulin signaling pathway	1	
hsa04912:GnRH signaling pathway	hsa04912:GnRH signaling pathway	1	
	hsa04913:Ovarian steroidogenesis		1
hsa04914:Progesterone-mediated oocyte maturation	hsa04914:Progesterone-mediated oocyte maturation	1	
hsa04915:Estrogen signaling pathway	hsa04915:Estrogen signaling pathway	1	
hsa04916:Melanogenesis	hsa04916:Melanogenesis	1	
hsa04917:Prolactin signaling pathway	hsa04917:Prolactin signaling pathway	1	
hsa04919:Thyroid hormone signaling pathway	hsa04919:Thyroid hormone signaling pathway	1	
hsa04920:Adipocytokine signaling pathway	hsa04920:Adipocytokine signaling pathway	1	
hsa04921:Oxytocin signaling pathway	hsa04921:Oxytocin signaling pathway	1	
	hsa04922:Glucagon signaling pathway		1
	hsa04923:Regulation of lipolysis in adipocytes		1
hsa04925:Aldosterone synthesis and secretion			1
hsa04110:Cell cycle	hsa04110:Cell cycle	1	
hsa04114:Oocyte meiosis	hsa04114:Oocyte meiosis	1	
hsa04115:p53 signaling pathway			1
hsa04144:Endocytosis			1
hsa04510:Focal adhesion	hsa04510:Focal adhesion	1	
hsa04520:Adherens junction	hsa04520:Adherens junction	1	
hsa04540:Gap junction	hsa04540:Gap junction	1	
	hsa04550:Signaling pathways regulating pluripotency of stem cells		1
hsa04810:Regulation of actin cytoskeleton	hsa04810:Regulation of actin cytoskeleton	1	
	hsa04261:Adrenergic signaling in cardiomyocytes		1
hsa04270:Vascular smooth muscle contraction	hsa04270:Vascular smooth muscle contraction	1	
hsa04320:Dorso-ventral axis formation	hsa04320:Dorso-ventral axis formation	1	
hsa04360:Axon guidance	hsa04360:Axon guidance	1	
hsa04380:Osteoclast differentiation	hsa04380:Osteoclast differentiation	1	
hsa04960:Aldosterone-regulated sodium reabsorption	hsa04960:Aldosterone-regulated sodium reabsorption	1	
hsa04961:Endocrine and other factor-regulated calcium reabsorption			1
hsa04930:Type II diabetes mellitus	hsa04930:Type II diabetes mellitus	1	
hsa04931:Insulin resistance	hsa04931:Insulin resistance	1	
hsa04713:Circadian entrainment			1
	hsa04932:Non-alcoholic fatty liver disease (NAFLD)		1
hsa04720:Long-term potentiation	hsa04720:Long-term potentiation	1	
hsa04722:Neurotrophin signaling pathway	hsa04722:Neurotrophin signaling pathway	1	
hsa04723:Retrograde endocannabinoid signaling	hsa04723:Retrograde endocannabinoid signaling	1	
hsa04725:Cholinergic synapse	hsa04725:Cholinergic synapse	1	
hsa04726:Serotonergic synapse	hsa04726:Serotonergic synapse	1	
	hsa04728:Dopaminergic synapse		1
hsa04730:Long-term depression	hsa04730:Long-term depression	1	
hsa04750:Inflammatory mediator regulation of TRP channels	hsa04750:Inflammatory mediator regulation of TRP channels	1	
hsa05010:Alzheimer's disease			1
hsa05014:Amyotrophic lateral sclerosis (ALS)	hsa05014:Amyotrophic lateral sclerosis (ALS)	1	
hsa05020:Prion diseases	hsa05020:Prion diseases	1	
hsa05030:Cocaine addiction	hsa05030:Cocaine addiction	1	
hsa05031:Amphetamine addiction			1
hsa05034:Alcoholism			1
hsa05100:Bacterial invasion of epithelial cells	hsa05100:Bacterial invasion of epithelial cells	1	
hsa05120:Epithelial cell signaling in Helicobacter pylori infection	hsa05120:Epithelial cell signaling in Helicobacter pylori infection	1	
hsa05130:Pathogenic Escherichia coli infection	hsa05130:Pathogenic Escherichia coli infection	1	
hsa05131:Shigellosis	hsa05131:Shigellosis	1	
hsa05132:Salmonella infection	hsa05132:Salmonella infection	1	
hsa05133:Pertussis	hsa05133:Pertussis	1	
hsa05140:Leishmaniasis	hsa05140:Leishmaniasis	1	
hsa05142:Chagas disease (American trypanosomiasis)	hsa05142:Chagas disease (American trypanosomiasis)	1	
	hsa05145:Toxoplasmosis		1
hsa05146:Amoebiasis			1
	hsa05152:Tuberculosis		1
hsa05160:Hepatitis C	hsa05160:Hepatitis C	1	
hsa05161:Hepatitis B	hsa05161:Hepatitis B	1	
hsa05162:Measles	hsa05162:Measles	1	
hsa05164:Influenza A	hsa05164:Influenza A	1	
hsa05166:HTLV-I infection	hsa05166:HTLV-I infection	1	
hsa05168:Herpes simplex infection	hsa05168:Herpes simplex infection	1	
hsa05169:Epstein-Barr virus infection	hsa05169:Epstein-Barr virus infection	1	
hsa05416:Viral myocarditis			1
hsa05200:Pathways in cancer	hsa05200:Pathways in cancer	1	
hsa05202:Transcriptional misregulation in cancer	hsa05202:Transcriptional misregulation in cancer	1	
hsa05203:Viral carcinogenesis	hsa05203:Viral carcinogenesis	1	
hsa05205:Proteoglycans in cancer	hsa05205:Proteoglycans in cancer	1	
hsa05206:MicroRNAs in cancer	hsa05206:MicroRNAs in cancer	1	
hsa05210:Colorectal cancer	hsa05210:Colorectal cancer	1	
hsa05211:Renal cell carcinoma	hsa05211:Renal cell carcinoma	1	
hsa05212:Pancreatic cancer	hsa05212:Pancreatic cancer	1	
hsa05213:Endometrial cancer	hsa05213:Endometrial cancer	1	
hsa05214:Glioma	hsa05214:Glioma	1	
hsa05215:Prostate cancer	hsa05215:Prostate cancer	1	
hsa05216:Thyroid cancer	hsa05216:Thyroid cancer	1	
hsa05218:Melanoma	hsa05218:Melanoma	1	
hsa05219:Bladder cancer	hsa05219:Bladder cancer	1	
hsa05220:Chronic myeloid leukemia	hsa05220:Chronic myeloid leukemia	1	
hsa05221:Acute myeloid leukemia	hsa05221:Acute myeloid leukemia	1	
hsa05222:Small cell lung cancer	hsa05222:Small cell lung cancer	1	
hsa05223:Non-small cell lung cancer	hsa05223:Non-small cell lung cancer	1	
hsa05230:Central carbon metabolism in cancer	hsa05230:Central carbon metabolism in cancer	1	
hsa05231:Choline metabolism in cancer	hsa05231:Choline metabolism in cancer	1	
hsa04062:Chemokine signaling pathway	hsa04062:Chemokine signaling pathway	1	
hsa04611:Platelet activation	hsa04611:Platelet activation	1	
hsa04620:Toll-like receptor signaling pathway	hsa04620:Toll-like receptor signaling pathway	1	
	hsa04621:NOD-like receptor signaling pathway		1
	hsa04622:RIG-I-like receptor signaling pathway		1
hsa04650:Natural killer cell mediated cytotoxicity	hsa04650:Natural killer cell mediated cytotoxicity	1	
hsa04660:T cell receptor signaling pathway	hsa04660:T cell receptor signaling pathway	1	
hsa04662:B cell receptor signaling pathway	hsa04662:B cell receptor signaling pathway	1	
hsa04664:Fc epsilon RI signaling pathway	hsa04664:Fc epsilon RI signaling pathway	1	
hsa04666:Fc gamma R-mediated phagocytosis	hsa04666:Fc gamma R-mediated phagocytosis	1	
hsa04670:Leukocyte transendothelial migration	hsa04670:Leukocyte transendothelial migration	1	
Sum of affected KEGG pathways: 103	Sum of affected KEGG pathways: 106	92	25

Comparison of signal transduction pathways that are affected in SKOV3 or OVCAR3, or in both cell lines.

## Discussion

It has been known for decades that cancer progression causes extensive metabolic rewiring in the malignant cells. Fatty acid synthase (FASN), the key enzyme that controls the biosynthesis of fatty acids and lipids, for example, is overexpressed in most tumours and correlates with malignant progression^3,14^. Accordingly, FASN has been assigned the function of a metabolic oncogene^1^ and it has been targeted with a variety of novel high-affinity inhibitors with anticancer potency including G28UCM^5,9,25^, which has been used in this study.

As expected, major lipid classes were downregulated in SKOV3 and OVCAR3 OC cells. FASN blockade caused depletion of all membrane lipids including phosphatidylcholines (PC), which are key components of the ER and the outer mitochondrial membrane, and sphingomyelins (SM) that act as building blocks for lipid rafts and organize signalling complexes in the membrane (Fig. 1c,d). Lipid deficiency correlated with low expression of the lipogenic enzymes FASN and acyl-CoA synthetase (Table 2 and Supplemental Table S3). Further in-depth analyses revealed cell line-specific differences. While lipid levels remained unchanged in SKOV3 during the first 8 h of drug exposure, they increased in OVCAR3. With longer treatment, however, the amount of lipids decreased in both cell lines relative to the 8-h time point (Table 1), and a shift from structural PL to storage lipids (TAG) was observed (Fig. 1b).

The pentose phosphate pathway—the main NADPH-regeneration process providing reducing power—has been found to be impaired by the treatment, and membrane saturation has been reduced due to overexpression of desaturases and accumulation of PUFAs. These conditions make cells susceptible to oxidative stress^26^, which increases during the treatment, because NADPH has been consumed during FA desaturation and cannot sufficiently be replenished by the pentose phosphate pathway. Thus blockade of FA synthesis likely impairs antioxidative mechanisms in SKOV3 and OVCAR3 cells. This is to our knowledge the first report demonstrating a direct negative effect of a FASN antagonist on the level of saturated FA in OC cells.

Production of FA is an essential anabolic cell pathway, and its blockade naturally has considerable consequences for the general metabolic balance in the cells. Thus perturbations were not restricted to lipids only, but occurred in all major metabolite classes, albeit they varied markedly between the two cell lines. As a rule, downregulations of most metabolites were more pronounced in SKOV3 than in OVCAR3 (Table 1). Overall, an unexpected divergence in the metabolic sensitivity of these two cell lines to FASN blockade was observed, despite the fact that growth and survival was stalled in both cell lines^8,9^. We have therefore hypothesized that disruption of cell metabolism due to a blockade of endogenous FA biosynthesis does not strictly follow a uniformly ordered pattern of mechanistic processes, but depends on internal cell context and external cell conditions. If this is the case, drug-induced cell metabolism disturbance may not be the main cause of treatment-related growth arrest and cell death. Rather, it could be the other way around—it could be the cause of drug resistance. SKOV3 represent highly invasive, multidrug-resistant cells with hyperactive ERBB2-PI3K/MAPK^27,28^. Signalling via this axis has been suggested to promote glycolysis and the Warburg effect^29,30^, which induces chemoresistance^31^. In contrast, OVCAR3 are less invasive, lack hyperactivation of PI3K or MAPK, exhibit higher levels of OXPHOS and are more sensitive^27–29^. Accordingly, we observed that the surviving cell fraction was smaller in OVCAR3 than in SKOV3 after high dose-G28UCM (Fig. 1a).

Therefore, we sought to develop a multi-omics approach that is capable of reflecting molecular processes related to either sensitivity or resistance to G28UCM. Accordingly, proteomic analysis revealed that metabolic pathway enzymes become downregulated within 8 h of drug exposure. However, the individual metabolic adjustments in glycolysis, TCA cycle and pentose phosphate pathway, OXPHOS and electron transport varied considerably between both cell lines, which may be due to their differential dependence on glycolysis and OXPHOS and may be associated with their differential resistance to high-dose G28UCM. The alterations in fatty acid metabolism and beta-oxidation, on the other hand, were highly concordant and very strong in both cell lines (Table 2 and Supplemental Table S3). This was also the case for apoptosis, membrane integrity/molecular uptake/transport, protein expression, kinase signalling, and stress pathways. In order to relatively quantify the similarity of response, a ‘SKOV3/OVCAR3 matching score’ was established and the basic cell processes were ranked according to this score. We believe that this semi-quantitative matching score can be used to identify those processes that are closely related to the drug effect being characterized by a high score (Figs. 3 and 4). It was of course not surprising to see maximal concordance in apoptosis and FA metabolic pathways, which reflects the toxicity and target selectivity of the inhibitor, while all other metabolic pathways ranked lowest. As expected, all processes in or close to membrane compartments were maximally affected by FASN blockade in both cell lines. This was true for structures of intracellular transport including the nuclear pore complex, which consists of proteins of the nucleoporin family, members of the solute carrier family proteins, lysosomal and multivesicular bodies, and many proteins from the trans-Golgi network such as coatomer complexes. Molecular transport thus completely ceased, corroborating our preliminary data, which suggest that pharmacological inhibition of FA synthesis, unlike previously thought, does not promote elevated uptake of exogenous lipids (data not shown). This aggravates stress at the ER and leads to a shut-down of protein expression due to ribosomal degradation, cessation of amino acid synthesis, and of translation and transcription (Table 2 and Supplemental Table S3). This disastrous situation can obviously have contradictory effects on protein turnover depending on the actual cell and environmental context. While downregulating proteasomal subunits in SKOV3, they accumulated in OVCAR3 (Table 2 and Supplemental Table S3), indicating a higher prevalence of protein degradation in sensitive OVCAR3 compared to resistant SKOV3 cells. The reason for this discordance has yet to be elucidated. Compartments that contain lipids as key elements naturally were exquisitely sensitive to FASN blockade. This also had major effects on cell signalling via lipid rafts and second messengers. Accordingly, major signal transduction systems such as receptor tyrosine kinase-, mTOR- and MAPK-pathways have been shut down. Finally, energy balance, including beta-oxidation, OXPHOS, and electron transport—representing highly ordered processes at or near the mitochondrial membrane—were disrupted.

In conclusion, while G28UCM-induced blockade of FASN strongly affects the metabolism in both OC cell lines, it yet caused quite distinct metabolite patterns in these cells. Data from the metabolome correlated well with the corresponding proteome and kinome in SKOV3 and OVCAR3 cells. Overall, our data suggest that damage to the membrane lipid bilayer and blockade of lipid signalling are the main causes of the anticancer effect of the FASN inhibitor, whereas rewiring of central carbon metabolism is just a secondary consequence of the primary failure in lipid balance that may contribute to FASN inhibitor resistance.

## Material and methods

### Cells, culture conditions and reagents

OC cell lines SKOV3 and OVCAR3 (ATCC, Manassas, VA) were maintained in α-MEM^32^. Media were supplemented with 10% fetal calf serum (FCS), 100 IU(µg)/ml penicillin–streptomycin and 2 mM glutamine (Gibco, Karlsruhe, Germany). Cells were maintained at 37 °C, 5% CO_2_ and 95% humidity and were tested for absence of viral/bacterial/fungal/mycoplasmal infection (Venor GeM, Minerva Biolabs, Berlin, Germany). The species origins were proven by species-PCR, and cell line identities were examined by fluorescent nonaplex-PCR of short tandem repeat markers in the year 2019 (DSMZ, Braunschweig, Germany). FASN inhibitor G28UCM (R. Colomer, M.L. López Rodríguez, Madrid, Spain)^6,33,34^ was dissolved in pure DMSO and stock solutions were diluted 1:1000 in media.

### Cell proliferation

Cells (500–3,000/well, 96-well plate) were grown overnight in medium containing 5% FCS to let them adhere, before media containing 5% FCS ± G28UCM were added. Cell numbers were determined after 72 h using a formazan dye assay (Biomedica, Vienna, Austria) as described^12,13,35^. Means ± SD of triplicate experiments are given. Statistically significant differences between G28UCM treated SKOV3 and OVCAR3 cells were examined using two-tailed Student's t-test at p < 0.05 (*), p < 0.01 (**) or p < 0.001 (***).

### Targeted metabolomics

Biological triplicates each of one million solvent- or G28UCM-treated cells were analysed. Cells were scraped in ice-cold lysis buffer (10 mM phosphate buffer in 85% ethanol) and lysed by three freeze–thaw cycles. Metabolomics experiments were realized using a fully validated method based on the AbsoluteIDQ p180 kit from Biocrates (Biocrates Life Sciences AG, Innsbruck, Austria), as described previously^36^. This kit allows to determine the levels of 186 metabolites including amino acids, biogenic amines, acylcarnitines, sphingolipids, glycerophospholipids and the sum of hexoses. Amino acids and biogenic amines were derivatized with phenyl-isothiocyanate and quantified by applying an LC–MS/MS method and by means of stable isotope-labelled internal standards included in the kit. The other metabolites were determined semi-quantitatively with a flow injection analysis (FIA)–MS/MS method using chemically homologous internal standards also included in the kit. Both, LC–MS/MS and FIA-MS/MS were carried out on a 4,000 QTRAP MS system (AB SCIEX, Framingham, MA) coupled to a 1,200 rapid resolution (RR)-high performance liquid chromatography (HPLC) system (Agilent, Palo Alto, CA), using Analyst 1.6.2 software (AB SCIEX, Redwood City, CA). For data analysis, Biocrates’ proprietary software was applied (MetIDQ, version 5–4–8-DB100-Boron-2607). Raw data of 3 independent measurements were expressed in % relative to vehicle control (control = 100%) and means ± SD were calculated. Statistically significant differences between control and inhibitor treated cells were determined by two-tailed Student's t-test at a level of significance of p < 0.05.

### Lipid extraction

Approximately 5 × 10^6^ cells from at least 2 separate experiments were suspended in PBS and centrifuged (5,000×*g*, 5 min). The supernatant was discarded, the cell pellet was washed 3 × with PBS and centrifuged (5,000×*g*, 5 min). The cells were then resuspended in 100 µL H_2_O, 1.6 mL CHCl_3_:MeOH [70:30(v/v)] was added, sonicated for 1 min and incubated on ice for 30 min. Then, 0.4 mL of 0.7 M aqueous formic acid was added, vortexed and centrifuged (5,000×*g*, 5 min) to separate the lower organic phase containing neutral- and phospholipids from the upper aqueous phase. Lipids from lower phase were vacuum dried, redissolved in 50 µL CHCl_3_:MeOH [70:30(v/v)] and samples from all experiments were stored at ≤ − 20 °C using glass vials before being subjected to the next steps.

### Thin-layer chromatography (TLC)

For TLC separation a previously established method was used^8^. Accordingly, plates (ALUGRAM Nano-SIL-G, Macherey–Nagel, Düren, Germany) were developed full-length (10 cm) with pure hexane, rotated by 90° and redeveloped full-length, then dried on a heating-plate (150 °C, 20 min) and 8–10 µL of lipid extracts were applied. Plates were developed using methyl-acetate:1-propanol:CHCl_3_:methanol:0.25%KCl [25:25:25:10:9(v/v/v/v/v)] until 4.5 cm from origin for separation of phospholipids, dried by hot air (1–2 min) and developed again using hexane:diethyl ether:acetic acid [80:20:1.5(v/v/v)] until 9.5 cm from origin for separation of neutral lipids. Plates were redried, sprayed with 0.05% primuline, photographed under UV-light and evaluated using GelAnalyzer software (https://www.gelanalyzer.com/).

### Matrix-assisted laser desorption/ionization mass spectrometry (MALDI-MS)

Mass spectra were recorded using an AXIMA-Performance (Shimadzu, Manchester, UK) curved-field reflectron time-of-flight (RTOF) mass spectrometer equipped with a 337 nm pulsed nitrogen laser. Measurements were performed using 6-aza-2-thiothymine (ATT) and 9-aminoacridine (9AA) as matrices for detection in the positive and negative mode, respectively^37,38^. The ion acceleration voltage was set to 20 kV and the reflectron analyser was operated at 25 kV. For structural confirmation of the lipid molecules a hybrid quadrupole iontrap (QIT)-TOF tandem-mass spectrometer (AXIMA-Resonance, Shimadzu) was used. Acquisition was performed in the low-mass range (m/z 300–1,000) and high-resolution (R = 1,000) ion selection modes for MS/MS experiments of monoisotopically selected precursor ions using low-energy collision induced dissociation (CID) with argon as the collision gas. The [M−H]^+^/[M−H]^-^ ions of lipid class specific standards (Avanti Polar Lipids, Alabaster, AL, USA) were used for mass spectral calibration and as internal standards for relative quantification of the cell-derived lipid species (Supplemental Fig. S2). Data processing was performed by Launchpad 2.9.3 software (Shimadzu) using the Savitzky-Golay smoothing algorithm. Identification of individual lipid species was performed on LIPID MAPS database search using MS and MS/MS based data (https://www.lipidmaps.org/tools/ms/).

### Proteomics analysis

Two million each of G28UCM- and DMSO-treated cells were subjected to cell lysis, as described previously^39^. In short, cells were lysed in ice-cold lysis buffer containing proteasome inhibitors, by applying mechanical shear stress. After centrifugation, proteins in the supernatant were precipitated overnight by adding ice-cold ethanol. The remaining pellet was dissolved in 500 mM NaCl and subsequently diluted in NP40-buffer. Dissolved proteins were recovered and precipitated overnight with ice-cold ethanol. After centrifugation, proteins were solubilized in sample buffer, and protein concentrations were assessed by applying a Bradford assay (Bio-Rad-Laboratories, Vienna, Austria). Proteins were further processed using a modified version of the FASP (filter-aided sample preparation) protocol^40,41^. In short, 25 µg of proteins were loaded onto a wetted MWCO filter (Pall Austria Filter GmbH, Vienna, Austria) with a pore size of 3 kD, followed by reduction of disulphide bonds with dithiothreitol (DTT), alkylation with iodoacetamide (IAA) and washing steps with 50 mM ammonium bicarbonate buffer. Proteins were digested by applying Trypsin/Lys-C (Mass Spec Grade quality; Promega, Mannheim, Germany) at 37 °C, first overnight, and then a second time for 4 h. Resulting peptides were eluted by centrifugation, followed by clean-up through a C-18 spin column (Pierce, Thermo Fisher Scientific, Austria).

For LC–MS/MS analyses, samples were reconstituted in 5 µl 30% formic acid (FA), supplemented with four synthetic peptide standards for internal quality control, and diluted with 40 µl mobile phase A (97.9% H_2_O, 2% ACN, 0.1% FA). Of this solution 5 µl were loaded on a C-18 Pepmap100 pre-column and eluted over a C-18 separation column at a flow rate of 300 nL/min using a 235 min gradient of 8–40% mobile phase B (79.9% ACN, 2% H_2_O, 0.1% FA). MS scans were performed in the range from m/z 400–1,400 at a resolution of 70,000 (at m/z = 200). MS/MS scans of the six most abundant ions were achieved through HCD fragmentation at 30% normalized collision energy and analysed in the orbitrap at a resolution of 17,500 (at m/z = 200). For each experimental condition three biological and two technical replicates were measured. For data analysis, the MaxQuant software (version 1.5.2.8), including the Andromeda search engine, and the Perseus statistical evaluation tool (version 1.5.2.6) were used^42,43^. Protein identifications were realized by searching against the human UniProt database (version 09/2014 with 20.193 reviewed protein entries) and applying false discovery rates (FDR) of 0.01 both on peptide and protein level. Relative protein quantification, based on label-free quantification (LFQ) values, was achieved by two-tailed t-tests using a p-value < 0.05. Data obtained from both biological and technical replicates were used; the LFQ values from technical replicates were averaged. Missing values were replaced from a normal distribution in order to enable t-testing. Twenty to thirty percent of proteins were found to be concordantly regulated in both LC–MS/MS based proteomic analysis and in antibody microarray based kinomic assays (see below). Thus, cross-validation of both techniques was possible for these proteins.

### Antibody microarray kinomics

SKOV3 and OVCAR3 cells (1.7 × 10^6^/100 mm dish) were treated with solvent or G28UCM for 24 h. Cells were then washed with ice-cold PBS and lysed in 400 μL lysis buffer (20 mM MOPS, pH 7.0, 2 mM EGTA, 5 mM EDTA, 50 mM sodium fluoride, 60 mM β-glycerophosphate, pH 7.2, 25 mM sodium pyrophosphate, 2.5 mM sodium orthovanadate, 50 nM phenylarsine oxide, 1% Triton X-100, 0.05% sodium dodecylsulphate, 0.5 µM aprotinin, 3 mM benzamidine, 1 mM Petabloc, 10 µM leupeptin, 1 mM dithiothreitol) followed by four cycles of sonication for 10 s each with 10 s intervals on ice to rupture the cell membranes and to shear the chromosomal DNA. Proteins were then subjected for 15 min to chemical cleavage at cysteines at 37 °C using 10 mM Tris(2-carboxyethyl)phosphine hydrochloride (added to lysis buffer before sonication) and 100 mM 2-nitro-5-thiocyanatobenzoic acid (added to lysis buffer after sonication). Resulting homogenates were then centrifuged at 90,000×*g* for 30 min at 4 °C and proteins in the supernatants were quantified using the Bradford assay and adjusted to 1 µg/µl. Aliquots of 60 µg protein of the whole cell lysates were then biotinylated, purified in microspin G-25 columns, diluted in sample diluent to a final volume of 400 µl and then incubated on the KAM-1325 antibody microarray following the manufacturer’s instructions (Kinexus Bioinformatics, Vancouver, BC, Canada). Microarray scanning and statistical data analysis was performed at Kinexus. For 20–30% of the proteins displayed on the microarray chips a comparison with the proteomic data for cross-check validation was possible.

### Gene ontology (GO) term enrichment, pathway- and process-analysis

Drug-regulated proteins and phosphoproteins identified by shotgun proteomic analysis and antibody microarray kinomic analysis, respectively, were subjected to functional annotation analysis using the ‘Database for Annotation, Visualization, and Integrated Discovery (DAVID) Resources 6.8′-platform^44^. This bioinformatics tool associates particular genes and/or proteins with biological functions, processes and pathways (‘GO terms’) that are controlled by these genes/proteins. The procedure employs a modified Fisher exact test to yield a specific p-value (‘EASE Score’) that identifies biological processes that are statistically significantly overrepresented (‘enriched’) in the submitted list of differentially expressed genes/proteins relative to the general population background list.

On this platform, a pathway-centered analysis of proteins that were significantly (p < 0.05) regulated by G28UCM in SKOV3 and OVCAR3 cells was performed with the built-in Functional Annotation Chart Tool linked to the Kyoto Encyclopedia of Genes and Genomes (KEGG) and the BioCarta databases. For evaluation of the data from shotgun proteomics we focused on key cell functional processes and associated sub-processes. However, for the evaluation of kinomic antibody microarray data, the application of the DAVID Functional Annotation Clustering Tool linked to KEGG was more informative. In this way, identified pathway clusters were combined to coherent ‘cell functional groups’ and these were integrated in fundamental ‘functional systems’ (FS).

### Establishment of ‘SKOV3/OVCAR3 matching scores’

To compare the trends of drug-mediated modulation (up or down) of key proteins in these (sub-)processes between the two cell lines, ‘SKOV3/OVCAR3 Matching Scores’ were established by estimating the analogy of responses in the two cell lines after 8 or 24 h of drug exposure, respectively. For individual proteins, a ‘SKOV3/OVCAR3 Matching Score’ of 1.00 designates uniform regulation at the same time in both cell lines, while a value of 0.00 indicates no uniform regulation in both cell lines at the specific time. For the entire key cell processes and sub-processes, the mean values of the estimated scores of all proteins associated to a particular (sub-)process were calculated. The resulting ‘SKOV3/OVCAR3 (Sub-)Process Matching Scores’ were used to classify the (sub-)processes according to their causal significance for the anticancer drug response of the cells, which is considered directly associated with the height of the score.

### Statistical analysis

Experimental data are presented as the mean ± standard deviation. Data were analysed by two-tailed Student’s t-test at p < 0.05 (*), p < 0.01 (**), and p < 0.001 (***). During GO term enrichment, pathway- and process-analysis a modified Fisher exact test was used to yield a specific p-value (‘EASE Score’) that identifies biological processes that are statistically significantly overrepresented (‘enriched’) in the submitted list of differentially expressed genes/proteins relative to the general population background list.

## Supplementary information

Supplementary file 1
