# Supplementary material for: Membrane disruption, but not metabolic rewiring, is the key mechanism of anticancer-action of FASN-inhibitors: a multi-omics analysis in ovarian cancer
Source: Sci Rep. 2020 Sep 10;10:14877. doi: 10.1038/s41598-020-71491-z (PMC7483762; doi:10.1038/s41598-020-71491-z)
Supplement: Supplementary file 1 [file 41598_2020_71491_MOESM1_ESM.pdf]

SUPPLEMENTAL INFORMATION

MEMBRANE DISRUPTION, BUT NOT METABOLIC REWIRING, IS THE KEY MECHANISM OF ANTICANCER-ACTION OF FASN-INHIBITORS – A MULTI-OMICS ANALYSIS IN OVARIAN CANCER

Thomas W. Grunt[1,2,3,*], Astrid Slany[4], Mariya Semkova[4], Ramón Colomer[5], María Luz López-Rodríguez[6], Michael Wuczkowski[7], Renate Wagner[1,2], Christopher Gerner[4], Gerald Stübiger[2,7]

[1]Cell Signaling & Metabolism Networks Program, Division of Oncology, Department of Medicine I, Medical University of Vienna, Austria; [2]Comprehensive Cancer Center, Vienna, Austria; [3]Ludwig Boltzmann Institute for Hematology & Oncology, Vienna, Austria; [4]Department of Analytical Chemistry, University of Vienna, Austria; [5]Department of Medical Oncology, Hospital Universitario La Princesa and Spanish National Cancer Research Centre (CNIO), Clinical Research Program, Madrid, Spain; [6]Departamento de Química Orgánica I, Facultad de Ciencias Químicas, Universidad Complutense de Madrid, Spain; [7]Department of Biomedical Imaging & Image-guided Therapy, Medical University of Vienna, Austria

*Corresponding author: Thomas W. Grunt, Division of Oncology, Department of Medicine I, Medical University of Vienna, Waehringer Guertel 18 – 20, A-1090 Vienna, Austria; P: +43 (0)1 40400-54570, F: +43 (0)1 40400-54650, Email: thomas.grunt@meduniwien.ac.at

SUPPLEMENTAL FIGURE LEGENDS

Supplemental Figure S1. Influence of FASN-inhibition on total amount of lipids per cell in (a) SKOV3 and (b) OVCAR3 cells, and (c) scheme of the action of FASN in *de novo* lipogenesis and the synthesis of the major cellular lipid classes. Relative change of total cellular lipids in (a) SKOV3 and (b) OVCAR3 cells upon G28UCM treatment (20µM, 72 hours). A decrease of 60% (SKOV3) to >80% (OVCAR3) in cell number and of about 30% lipid content was observed compared to cells grown in 0.1% DMSO. (c) Palmitate (16:0) as the primary reaction product of FASN is further processed to acyl-CoA via several steps of elongation and desaturation leading to fatty acids with different carbon chain-lengths and number of double bonds. These fatty acids are then esterified to cholesterol or glycerol to generate cholesterol ester (CE), triacylglycerol (TAG), diacylglycerol (DAG) and phospholipids (PL) as the major structural (e.g. membrane), signalling and storage lipid classes.

Supplemental Figure S2. Representative MALDI mass spectra of lipid extracts of untreated and G28UCM treated SKOV3 (a, b) and OVCAR3 (c, d) recorded in positive (a, c) and negative (b, d) ionization mode at 8 hours and 24 hours. Phospholipid class specific internal standards (indicated by asterisks) were added to the samples for semiquantitative analysis of the corresponding lipid species. Abbreviations: LPC, lysophosphatidylcholine; PC, phosphatidylcholine; PE, phosphatidylethanolamine; PG, phosphatidyglycerol; PI, phosphatidylinositol; PS, phosphatidylserine

Supplemental Figure S3. Reproducibility of glycerophospholipid analysis by MALDI-MS. Six individual control cell culture samples were prepared according to the lipid extraction and MS analysis protocols described in Material and Methods. In (a) the data from the independent analysis of the lipid extracts displaying the relative abundances of the sum of individual lipid species relative to class specific internal standards (Ratio vs. Int. Std.) are displayed and in (b) their means ± SD are shown. Numbers above the error bars indicate the coefficient of variation (CV %). Abbreviations: CL, cardiolipin; LPC, lysophosphatidylcholine; PC, phosphatidylcholine; PE, phosphatidylethanolamine; PG, phosphatidylglycerol; PI, phosphatidylinositol; PS, phosphatidylserine; SM, sphingomyelin.

Supplemental Figure S4. Effects of FASN-inhibition on the phosphatidylcholine (PC) composition of SKOV3 and OVCAR3 cells. Changes in the relative composition of PC species containing fatty acid residues with 0-2 total double bonds (DBs) in (a) SKOV3 and (b) OVCAR3 cells treated with 0.1% DMSO and 40µM G28UCM for 8 hours and 24 hours. Displayed is the relative composition of PC

species with 0-2 DBs in % of total PC. Values are means ± SD (n = 3). Letter code of the fatty acid residues: L, linoleate (18:2); O, oleate (18:1); P, palmitate (16:0); S, stearate (18:0).

SUPPLEMENTAL TABLE LEGENDS

Supplemental Table S1: Reproducibility analysis of biological and technical replicates of SKOV3 samples. Glycerophospholipids were quantitatively measured by LC-ESI-MS/MS according to the AbsoluteIDQ p180 kit (see Material and Methods). Samples of three independent cell cultures (biological replica) treated with 0.1% DMSO (control) or 40µM G28UCM for 8 hours and 24 hours were measured two times each (technical replica). From that the standard deviation (SD) and coefficient of variation (CV) for the individual lipid species were calculated.

Supplemental Table S2: Shotgun proteomic analysis of SKOV3 (a) and OVCAR3 (b) cells exposed for 8 hours and 24 hours to 40µM G28UCM. All proteins identified by MS/MS. Colour Code:

8h Up	24h Up	8h Down
24h Down		

Supplemental Table S3: DAVID-assisted shotgun proteomic analysis of key cell processes in SKOV3 and OVCAR3 cells exposed to 40µM G28UCM using BioCarta and KEGG databases.

**Legend**				
Significantly ($p < 0.05$) downregulated proteins: Blue cell				
Significantly ($p < 0.05$) upregulated proteins: Red cell				
**For Individual Proteins**				
SKOV3/OVCAR3 Matching Score:				
1,00: uniform regulation at the same time in both cell lines.				
0,00: no uniform regulation in both cell lines at the specific time.				
**For Key Cell Processes or Sub-Processes**				
Mean SKOV3/OVCAR3 Matching Score:				
Mean of 'Individual Protein SKOV3/OVCAR3 Matching Scores'				

Supplemental Table S4: Summary of all down- (< 50 % of Control) and up-regulated (> 150 % of Control) phosphoproteins as determined by antibody microarray kinomic analysis in SKOV3 (a) and OVCAR3 (b) cells exposed for 24 hours to 40µM G28UCM.

Supplemental Figure S1

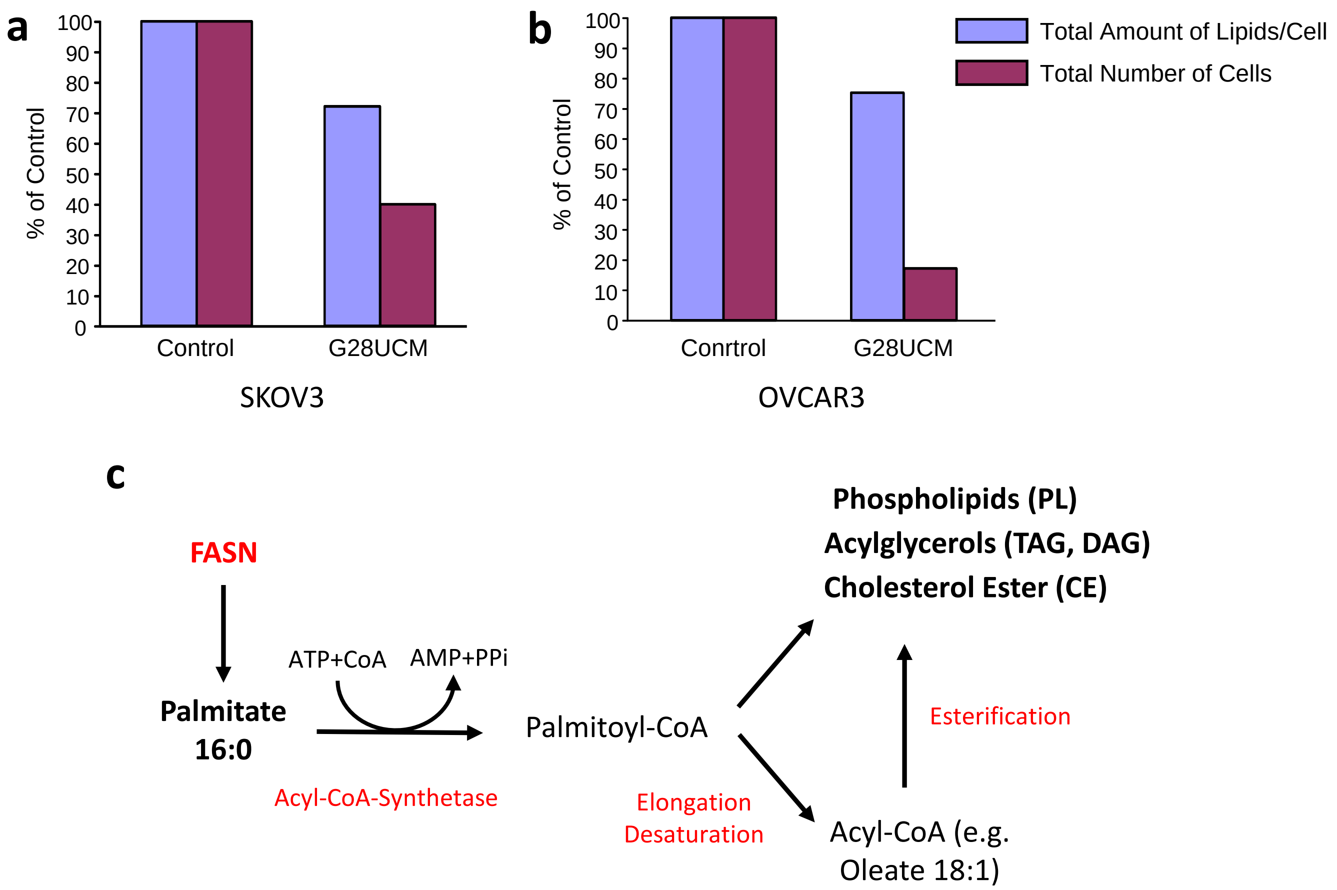

Supplemental Figure S2

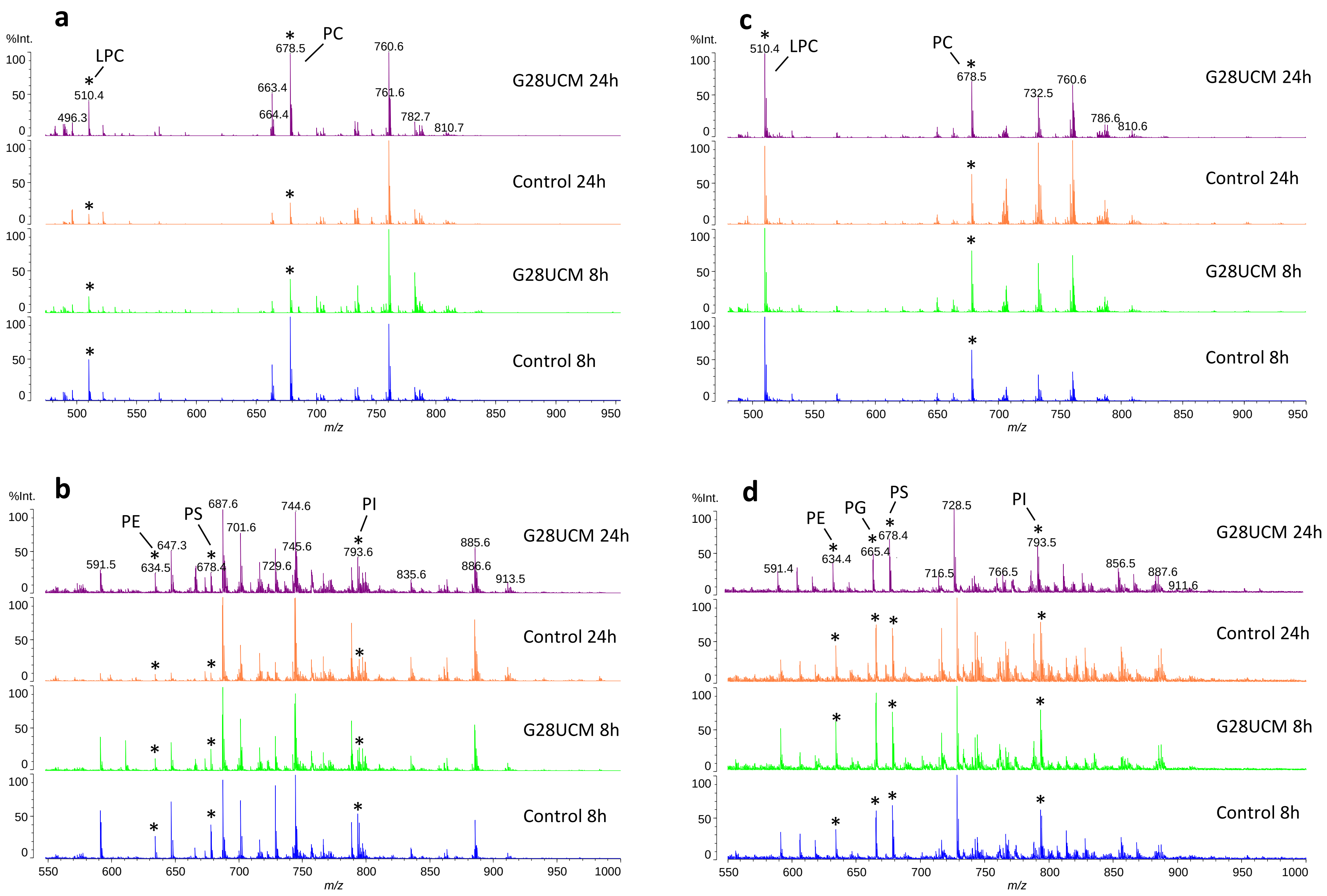

Supplemental Figure S3

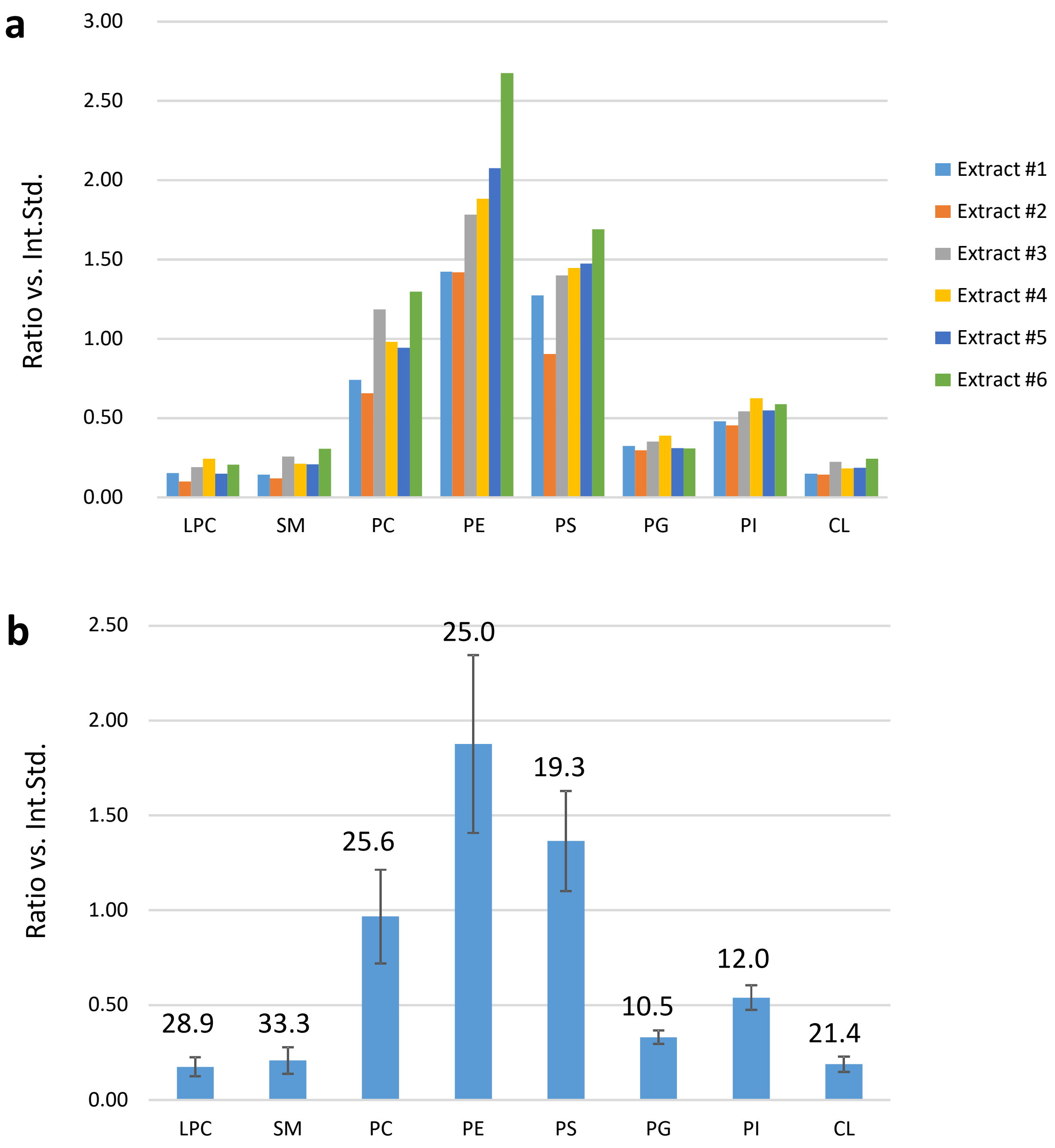

Supplemental Figure S4

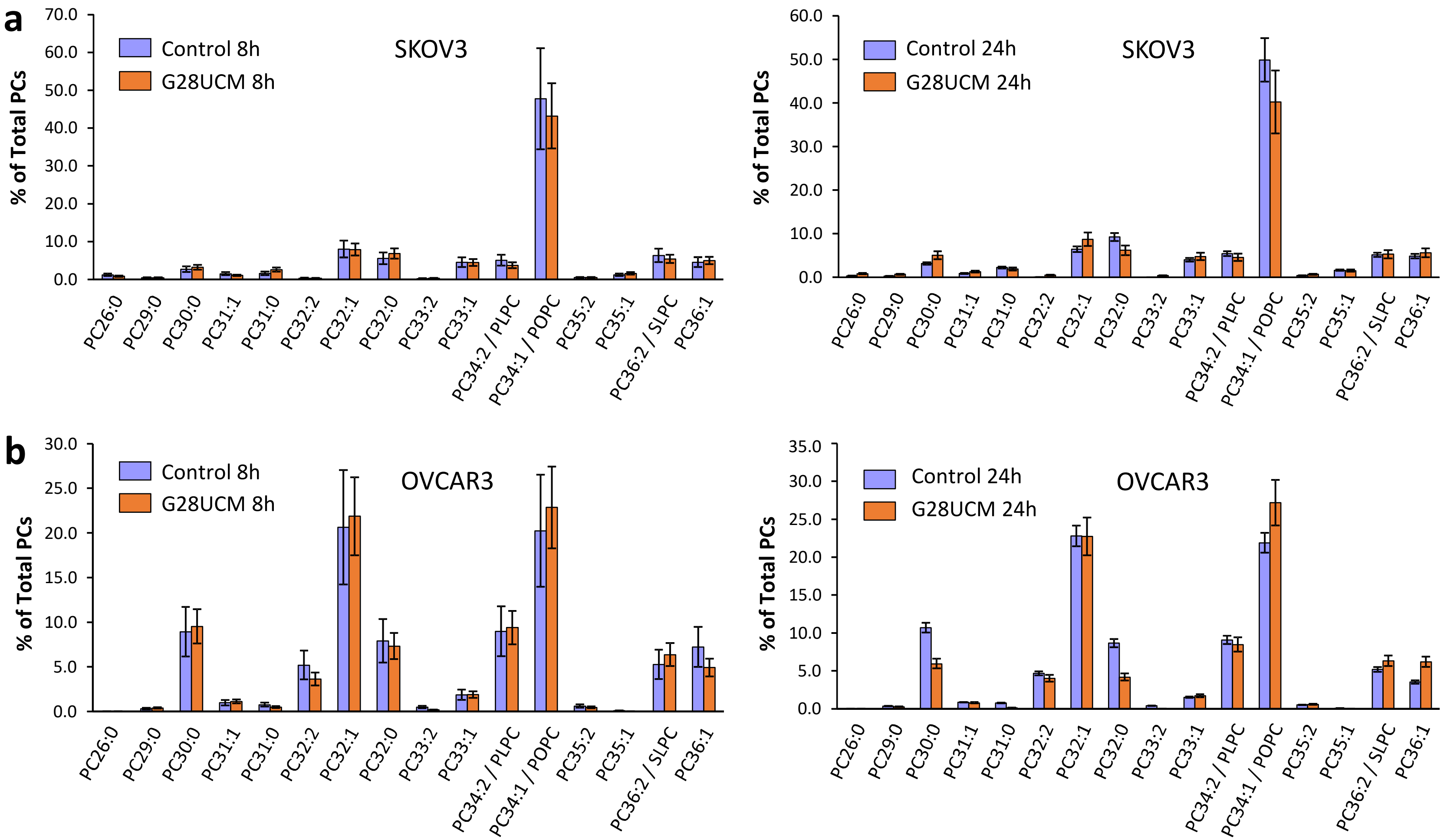

Supplemental Table S1: Reproducibility analysis of biological and technical replicates of SKOV3 samples

8h samples	1 - SKOV con1 8h control	2 - SKOV con2 8h control	3 - SKOV con3 8h control	4 - SKOV FASN1 8h G28UCM	5 - SKOV FASN2 8h G28UCM
lysoPC a C14:0	8,07	9,2	7,75	8,44	11
lysoPC a C16:0	8,33	6,65	9,39	6,52	10,3
lysoPC a C16:1	14,6	19	17,7	15,8	23,6
lysoPC a C17:0	0,622	0,569	0,678	0,503	0,62
lysoPC a C18:0	1,48	0,836	1,71	0,993	1,59
lysoPC a C18:1	50,1	39,3	49,7	43,3	61,6
lysoPC a C18:2	4,7	5,25	5,01	4,45	7,22
lysoPC a C20:3	1,99	1,64	1,97	1,74	2,69
lysoPC a C20:4	4,42	4,87	5,25	3,56	5,07
lysoPC a C24:0	0,852	0,904	1,37	0,689	0,963
lysoPC a C26:0	1,69	1,56	2,83	1,65	2,27
lysoPC a C26:1	0,422	0,373	0,656	0,351	0,418
lysoPC a C28:0	2,22	1,23	2,53	1,31	2,02
lysoPC a C28:1	0,988	0,812	1,37	0,848	1,15
PC aa C24:0	0,269	0,483	0,459	0,309	0,476
PC aa C26:0	1,94	2,36	2,83	2,2	3,24
PC aa C28:1	1,18	1,47	1,69	1,1	1,73
PC aa C30:0	11,9	9,38	13,2	10,9	12,1
PC aa C30:2	0,147	0,164	0,35	0,16	0,359
PC aa C32:0	29,8	16,4	37	23,4	19,4
PC aa C32:1	79,2	45,6	97,5	58,3	88,2
PC aa C32:2	5,71	3,41	7,74	3,82	5,89
PC aa C32:3	0,339	0,296	0,558	0,235	0,362
PC aa C34:1	285	163	343	224	305
PC aa C34:2	47,6	28,9	61,8	34,5	54,6
PC aa C34:3	3,41	2,01	4,39	2,31	3,67
PC aa C34:4	0,452	0,281	0,511	0,274	0,406
PC aa C36:0	2,51	1,57	3,16	2,02	2,69
PC aa C36:1	33,6	16,8	33,9	30,1	28
PC aa C36:2	75,8	46,9	102	65	93,5
PC aa C36:3	14,9	8,83	18,4	11,7	18,5

**PC aa C36:4**	9,44	5,08	10,9	5,89	9,37
**PC aa C36:5**	3,36	1,99	4,02	2,27	3,67
**PC aa C36:6**	0,524	0,37	0,569	0,371	0,494
**PC aa C38:0**	3	2,1	4,2	2,51	3,73
**PC aa C38:1**	1,39	0,71	1,39	1,4	1,12
**PC aa C38:3**	6,49	3,35	7,97	5,17	6,51
**PC aa C38:4**	10,5	6,09	12,4	8,18	11,9
**PC aa C38:5**	13,4	7,93	16,7	10,2	16,5
**PC aa C38:6**	6,14	3,44	7,17	4,52	7,16
**PC aa C40:1**	0,417	0,297	0,523	0,392	0,412
**PC aa C40:2**	0,654	0,437	0,963	0,722	0,689
**PC aa C40:3**	0,648	0,374	0,922	0,635	0,613
**PC aa C40:4**	0,982	0,519	1,19	0,865	1,06
**PC aa C40:5**	3,08	1,7	3,52	2,36	2,97
**PC aa C40:6**	6,33	3,77	7,53	4,97	7,35
**PC aa C42:0**	0,138	0,116	0,201	0,118	0,138
**PC aa C42:1**	0,122	0,082	0,18	0,13	0,127
**PC aa C42:2**	0,249	0,153	0,339	0,22	0,245
**PC aa C42:4**	0,233	0,17	0,301	0,178	0,218
**PC aa C42:5**	0,255	0,147	0,331	0,217	0,285
**PC aa C42:6**	0,501	0,374	0,624	0,44	0,479
**PC ae C30:0**	6,36	5,67	7,72	6,02	7,19
**PC ae C30:1**	2,83	2,57	3,19	2,42	3,22
**PC ae C30:2**	0,29	0,312	0,348	0,234	0,342
**PC ae C32:1**	47,5	27,8	61,6	39,2	53
**PC ae C32:2**	8,32	4,74	10,7	6,25	10,2
**PC ae C34:0**	4,27	1,8	4,12	3,29	2,85
**PC ae C34:1**	67,6	36,9	83,3	53,2	69,7
**PC ae C34:2**	24,7	13,3	28,7	19	27,4
**PC ae C34:3**	2,5	1,46	2,83	1,8	2,9
**PC ae C36:0**	2,17	1,41	3,07	1,87	1,78
**PC ae C36:1**	16,3	8,23	18,8	14,9	14,9
**PC ae C36:2**	16,2	9,5	20,5	14,1	18,9
**PC ae C36:3**	10,3	5,59	12,4	8,63	12,2
**PC ae C36:4**	11,9	7	14,6	8,57	13,5
**PC ae C36:5**	5,64	3,37	6,41	3,94	6,18
**PC ae C38:0**	0,98	0,662	1,14	0,867	1,1

**PC ae C38:1**	3,07	1,54	3,74	3,15	2,8
**PC ae C38:2**	4,52	2,46	6	4,56	5,22
**PC ae C38:3**	3,45	1,77	4,29	2,96	3,74
**PC ae C38:4**	6,6	3,78	8	5,45	7,32
**PC ae C38:5**	12,9	7,75	16,2	10,4	15
**PC ae C38:6**	9,33	5,42	11,2	7,09	10,3
**PC ae C40:1**	0,995	0,667	1,03	0,771	1,04
**PC ae C40:2**	0,945	0,419	1,09	0,845	0,918
**PC ae C40:3**	1,07	0,543	1,41	1,02	1
**PC ae C40:4**	1,53	0,873	1,91	1,28	1,44
**PC ae C40:5**	3,65	1,99	4,35	3,13	3,89
**PC ae C40:6**	4,52	2,52	5,61	3,86	5,5
**PC ae C42:0**	0,838	0,579	0,889	0,748	0,757
**PC ae C42:1**	0,486	0,251	0,523	0,34	0,513
**PC ae C42:2**	0,632	0,419	0,803	0,468	0,687
**PC ae C42:3**	0,746	0,474	0,827	0,456	0,761
**PC ae C42:4**	0,261	0,174	0,367	0,257	0,306
**PC ae C42:5**	0,875	0,544	1,01	0,815	0,893
**PC ae C44:3**	0,248	0,191	0,272	0,15	0,23
**PC ae C44:4**	0,234	0,153	0,318	0,207	0,272
**PC ae C44:5**	0,23	0,144	0,255	0,196	0,242
**PC ae C44:6**	0,153	0,106	0,186	0,166	0,163

**24h samples**	7 - SKOV con1 24h control	8 - SKOV con2 24h control	9 - SKOV con3 24h control	10 - SKOV FASN1 24 G28UCM	11 - SKOV FASN2 24h G28UCM
**lysoPC a C14:0**	10,3	10,8	10,6	12	9,18
**lysoPC a C16:0**	12,1	13,6	12,5	10,2	7,92
**lysoPC a C16:1**	21	23,9	23,2	23,5	21,7
**lysoPC a C17:0**	0,603	0,609	0,608	0,718	0,547
**lysoPC a C18:0**	2,16	1,87	1,71	1,38	0,933
**lysoPC a C18:1**	57,2	59,9	56,8	62	58

**lysoPC a C18:2**	5,03	6,03	5,69	6,79	6,39
**lysoPC a C20:3**	2,32	2,51	2,38	2,09	2,15
**lysoPC a C20:4**	4,84	5,46	5,16	3,99	3,63
**lysoPC a C24:0**	1,87	1,61	1,53	0,93	0,948
**lysoPC a C26:0**	3,57	3,1	3,15	2,07	2,31
**lysoPC a C26:1**	0,745	0,672	0,742	0,533	0,567
**lysoPC a C28:0**	3,08	2,6	2,65	2,42	1,9
**lysoPC a C28:1**	1,63	1,33	1,4	1,26	1,36
**PC aa C24:0**	0,532	0,64	0,715	0,322	0,46
**PC aa C26:0**	3,93	3,68	4,03	2,31	3,67
**PC aa C28:1**	1,97	2	2,11	1,5	2,01
**PC aa C30:0**	17,1	17,8	17,2	14,5	12,4
**PC aa C30:2**	0,428	0,361	0,373	0,359	0,466
**PC aa C32:0**	36,7	39,3	32,9	27,9	16,6
**PC aa C32:1**	96,2	106	95,8	88,1	68,6
**PC aa C32:2**	7,81	8,73	7,52	5,47	5,01
**PC aa C32:3**	0,543	0,556	0,567	0,37	0,326
**PC aa C34:1**	305	329	318	304	228
**PC aa C34:2**	57,6	66,3	59,7	49	44,8
**PC aa C34:3**	4,24	4,83	4,28	2,95	2,79
**PC aa C34:4**	0,466	0,528	0,511	0,331	0,293
**PC aa C36:0**	3,01	3,34	3,01	3,06	2,12
**PC aa C36:1**	29,7	27,3	26	27,5	18,7
**PC aa C36:2**	91,1	98	91,1	82,4	75,8
**PC aa C36:3**	16,6	18,6	16,8	14,9	13
**PC aa C36:4**	9,81	12	10,5	6,97	5,67
**PC aa C36:5**	3,05	3,47	3,13	2,65	2,16
**PC aa C36:6**	0,392	0,487	0,51	0,408	0,379
**PC aa C38:0**	4,44	4,63	4,5	4,26	3,68
**PC aa C38:1**	1,67	1,6	1,55	1,33	1,07
**PC aa C38:3**	7,58	8,29	7,58	5,77	5,22
**PC aa C38:4**	8,8	9,95	8,99	7,6	6,8
**PC aa C38:5**	11,2	11,7	10,9	9,65	7,93
**PC aa C38:6**	5,53	6,23	5,67	4,78	3,85
**PC aa C40:1**	0,578	0,441	0,529	0,473	0,463
**PC aa C40:2**	1,08	0,973	0,96	0,737	0,726
**PC aa C40:3**	1,01	0,989	0,881	0,622	0,614

**PC aa C40:4**	1,14	1,17	1,09	0,875	0,782
**PC aa C40:5**	2,57	2,56	2,31	2,14	1,8
**PC aa C40:6**	4,39	4,46	4,16	4,28	3,86
**PC aa C42:0**	0,192	0,179	0,197	0,149	0,131
**PC aa C42:1**	0,188	0,202	0,185	0,11	0,075
**PC aa C42:2**	0,355	0,336	0,317	0,251	0,237
**PC aa C42:4**	0,326	0,307	0,318	0,294	0,252
**PC aa C42:5**	0,283	0,283	0,288	0,209	0,171
**PC aa C42:6**	0,494	0,582	0,504	0,434	0,391
**PC ae C30:0**	10,3	9,09	9,52	9,63	7,48
**PC ae C30:1**	4,34	3,98	4,08	4,13	3,74
**PC ae C30:2**	0,456	0,473	0,455	0,341	0,464
**PC ae C32:1**	65,6	67,8	62,1	64,2	49,4
**PC ae C32:2**	11,8	13,3	12,6	11,2	9,22
**PC ae C34:0**	4,44	4,29	4,21	3,67	2,59
**PC ae C34:1**	86	90	82,2	82,8	58,1
**PC ae C34:2**	31,3	34,7	32,1	29,9	23,5
**PC ae C34:3**	3,32	3,88	3,51	3,11	2,52
**PC ae C36:0**	2,14	1,7	1,69	2,27	1,59
**PC ae C36:1**	21,2	20,1	19,2	18,5	13,1
**PC ae C36:2**	23,2	24,6	23	21,3	19,2
**PC ae C36:3**	14,9	15,2	14,8	13,3	11,4
**PC ae C36:4**	16,7	17,9	17	14,2	12
**PC ae C36:5**	7,22	7,7	7,46	6,52	5,19
**PC ae C38:0**	0,868	0,836	0,738	0,834	0,614
**PC ae C38:1**	4,8	4,16	4,07	3,13	2,65
**PC ae C38:2**	7,08	6,96	6,51	5,37	4,65
**PC ae C38:3**	5,54	5,79	5,33	4,17	3,33
**PC ae C38:4**	9,7	9,95	9,02	8,2	7,06
**PC ae C38:5**	18,7	19,8	18,7	17,6	14,4
**PC ae C38:6**	12,8	13,6	12,9	11,7	9,65
**PC ae C40:1**	0,912	0,786	0,786	0,467	0,484
**PC ae C40:2**	1,56	1,45	1,36	0,898	0,781
**PC ae C40:3**	1,72	1,5	1,49	1,25	0,945
**PC ae C40:4**	2,22	2,26	2,03	1,69	1,4
**PC ae C40:5**	5,06	5,07	4,66	4,42	3,78
**PC ae C40:6**	6,82	6,96	6,59	6,34	5,42

**PC ae C42:0**	0,684	0,658	0,668	0,674	0,544
**PC ae C42:1**	0,303	0,276	0,288	0,242	0,182
**PC ae C42:2**	0,308	0,308	0,249	0,199	0,216
**PC ae C42:3**	0,397	0,465	0,438	0,254	0,258
**PC ae C42:4**	0,408	0,373	0,372	0,215	0,193
**PC ae C42:5**	1,17	1,05	1,02	0,88	0,782
**PC ae C44:3**	0,136	0,154	0,184	0,091	0,102
**PC ae C44:4**	0,157	0,174	0,142	0,112	0,112
**PC ae C44:5**	0,238	0,201	0,253	0,14	0,142
**PC ae C44:6**	0,254	0,207	0,191	0,169	0,145

6 - SKOV FASN3 8h G28UCM	1 - SKOV con1 8h repl control	2 - SKOV con2 8h repl control	3 - SKOV con3 8h repl control	4 - SKOV FASN1 8h repl G28UCM
12,8	13,3	9,06	6,85	6,92
11,9	12,7	6,79	10,6	6,32
27,4	23,2	20,3	16,5	12,5
0,672	0,728	0,568	0,568	0,444
2,22	2,69	1,15	1,72	1,18
67,7	65,9	41,7	52,9	39,3
9,11	7,31	5,74	4,46	3,95
3,3	2,76	1,95	2,13	1,66
6,44	6,03	5,31	5,02	3,22
1,49	1,24	0,937	1,12	0,798
3,22	2,67	1,79	2,35	1,84
0,738	0,648	0,451	0,638	0,387
2,91	2,43	1,65	2,73	1,38
1,52	1,1	0,939	1,43	0,826
0,778	0,651	0,464	0,305	0,356
4,26	4,42	2,57	2,19	2,21
1,89	1,84	1,49	1,49	1,14
12,1	12,3	8,55	13,2	10,4
0,294	0,197	0,173	0,32	0,203
16,5	21,8	14,7	32,3	18,1
65,8	64,9	48,7	102	49,1
3,86	4,35	3,77	8,48	3,12
0,283	0,339	0,288	0,521	0,171
241	249	168	334	201
41,4	44,4	31,2	64	30,2
2,73	2,8	2,28	4,54	1,91
0,356	0,371	0,295	0,481	0,252
2,08	2,29	1,64	2,88	1,67
24,3	27,1	15,2	33,4	25,8
61,8	68,9	46,8	97,3	58,7
13,2	13,7	9,14	18,5	10,6

7,91	8,43	5,44	11,3	5,09
3,21	3,07	2,13	3,82	1,89
0,411	0,384	0,304	0,565	0,241
2,4	2,96	2,02	3,72	2,3
1,12	1,37	0,719	1,4	1
4,84	6,22	3,38	7,55	4,71
8,5	10,2	5,77	12	7,2
11,3	12,6	8,52	16	8,71
5,61	5,96	3,9	6,91	3,74
0,381	0,412	0,343	0,437	0,408
0,484	0,653	0,368	0,808	0,618
0,479	0,675	0,391	0,83	0,54
0,75	0,985	0,592	1,14	0,753
2,08	2,91	1,55	3,3	2,22
5,07	5,91	3,71	6,92	4,44
0,114	0,156	0,105	0,152	0,117
0,125	0,146	0,082	0,153	0,12
0,215	0,262	0,157	0,276	0,217
0,175	0,18	0,127	0,236	0,162
0,19	0,251	0,116	0,314	0,212
0,495	0,485	0,326	0,524	0,407
7,22	6,46	5,14	7,6	5,83
3,56	3,35	2,41	3,21	2,43
0,407	0,418	0,27	0,329	0,239
40,2	39,4	29,2	61,4	33,3
7,6	7,31	5,41	11,2	5
2,25	3,41	1,75	3,88	2,57
55,1	56,7	36,5	85,2	49,2
21	21,8	13,5	31,5	16,5
2,26	2,43	1,58	3,22	1,55
1,17	1,7	1,06	2,06	1,71
12,9	14	8,36	19,7	13,5
14,2	14,8	9,33	21,7	12,7
8,85	9,63	5,81	12,7	7,39
10,2	11,2	7,56	14,5	7,58
4,85	5,09	3,72	6,94	3,16
0,806	0,858	0,625	1,06	0,695

2,16	3,31	1,38	3,65	2,9
3,41	4,75	2,41	6,12	4,1
2,66	3,28	1,87	4,5	2,8
5,23	6,17	3,62	8,22	4,68
10,3	12,1	8,36	16	9,06
7,6	8,6	5,87	11,2	5,93
0,823	1,03	0,604	1,03	0,701
0,722	1,03	0,459	1,16	0,866
0,803	1,03	0,511	1,38	0,892
1,12	1,53	0,786	1,73	1,21
2,73	3,63	1,86	4,25	2,8
3,74	4,59	2,68	5,7	3,51
0,609	0,718	0,557	0,832	0,591
0,439	0,41	0,262	0,514	0,336
0,506	0,618	0,408	0,708	0,413
0,638	0,726	0,475	0,739	0,428
0,253	0,365	0,124	0,354	0,228
0,705	0,915	0,563	0,947	0,754
0,282	0,278	0,169	0,263	0,135
0,256	0,257	0,148	0,253	0,176
0,197	0,241	0,166	0,23	0,191
0,165	0,171	0,071	0,175	0,135

12 - SKOV FASN3 24h G28UCM	7 - SKOV con1 24h repl control	8 - SKOV con2 24h repl control	9 - SKOV con3 24h repl control	10 - SKOV FASN1 24h rep G28UCM
10,7	11,5	17,2	14	13,4
8,55	14,7	17,7	15,4	11,1
18,4	24,1	31	27,2	23,9
0,479	0,629	0,657	0,62	0,517
1,16	2,44	2,53	2,46	1,57
46,5	66,1	72,5	61,5	66,6

5,44	5,81	7	6,48	7,18
1,67	2,53	2,78	2,57	2,24
3,07	5,59	6,29	5,56	4,06
1,02	1,53	1,6	1,62	1,02
2,31	3,51	3,5	3,23	2,3
0,437	0,601	0,733	0,66	0,496
2,12	3,31	2,49	2,58	2,24
1,18	1,46	1,37	1,3	1,1
0,315	0,849	0,741	0,773	0,379
2,56	4,5	4,79	4,57	2,83
1,47	1,82	2,31	2,08	1,49
10,1	19,8	19,3	17,4	12,7
0,278	0,434	0,408	0,532	0,245
13,7	44,6	39,9	36,2	15,5
72,4	121	108	96,4	73,7
4,42	10,1	8,41	7,55	4,5
0,307	0,641	0,559	0,58	0,351
238	390	368	316	268
41,4	75,4	66,5	62,2	43,8
2,56	5,82	4,77	4,32	2,73
0,269	0,617	0,516	0,494	0,303
2,29	3,71	3,3	3,1	2,29
15,3	37,8	27,9	26	22
66,2	118	102	92	74,5
12,1	22,7	18,9	17,4	13
5,66	13,3	11,7	11	6,14
2,12	4,15	3,33	3,73	2,22
0,297	0,6	0,479	0,519	0,358
3,14	5,43	4,61	4,58	3,62
0,792	2,09	1,79	1,5	1,17
4,41	10	8,49	7,78	5,4
6,15	11,4	9,77	9,42	6,9
7,4	14,9	12,6	12,7	8,31
3,72	7,36	6,2	6,44	4,31
0,277	0,66	0,498	0,508	0,386
0,573	1,29	1,01	0,868	0,828
0,478	1,21	0,994	0,88	0,612

0,567	1,43	1,16	1,07	0,721
1,55	3,29	2,72	2,64	1,98
3,25	5,24	4,62	4,73	3,71
0,104	0,224	0,156	0,21	0,133
0,066	0,231	0,194	0,157	0,084
0,215	0,37	0,294	0,324	0,289
0,196	0,415	0,296	0,269	0,256
0,15	0,441	0,309	0,296	0,201
0,307	0,637	0,592	0,587	0,389
6,83	11,2	10,2	9,41	8,08
3,3	4,68	4,11	3,97	3,74
0,357	0,524	0,458	0,488	0,409
50,1	80,3	70,1	62,6	55
9,12	15,2	13,2	11,8	10,1
2,17	5,57	4,39	4,3	3,05
60,4	110	95,9	82,3	68,5
23,5	40,3	35,6	30,9	27,5
2,48	4,47	3,92	3,48	2,75
1,3	2,5	1,67	1,72	1,61
11,4	26,7	22,3	18,9	14,6
16,2	29,7	25,6	22,5	19,1
10,4	18,8	16,6	14,3	11,9
11,3	21,5	18,8	16,5	12,9
5,11	9,15	8,07	7,27	5,59
0,499	1,14	0,912	0,936	0,621
1,85	5,69	4,25	4,02	2,71
3,79	8,76	7,3	6,72	4,81
3,08	7,06	6,01	5,3	3,75
6,19	12,1	10,4	9,52	7,53
13,1	23,4	20,2	18,3	15
8,98	16,3	14,4	12,9	10,6
0,312	1,03	0,899	0,843	0,474
0,593	1,74	1,44	1,39	0,83
0,757	2,03	1,65	1,48	0,964
1,19	2,7	2,34	2	1,47
3,18	6,1	5,19	4,88	4,04
4,92	8,27	7,27	6,48	5,56

0,547	0,761	0,72	0,677	0,573
0,175	0,37	0,302	0,289	0,189
0,158	0,406	0,31	0,277	0,186
0,171	0,587	0,489	0,459	0,275
0,16	0,491	0,431	0,371	0,206
0,636	1,23	1,07	1,09	0,82
0,08	0,242	0,243	0,169	0,099
0,106	0,209	0,153	0,157	0,113
0,105	0,316	0,242	0,212	0,139
0,099	0,351	0,249	0,247	0,118

		Biological replica:	3 independent cell cultures		
5 - SKOV FASN2 8h repl G28UCM	6 - SKOV FASN3 8h rpl G28UCM		**SKOV cont 8h control**	SKOV cont repl 8h control	**SKOV G28UCM 8h** G28UCM
9,91	9,48	**lysoPC a C14:0**	**8,34**	9,74	**10,75**
9,39	8,44	**lysoPC a C16:0**	**8,12**	10,03	**9,57**
22,9	19,9	**lysoPC a C16:1**	**17,10**	20,00	**22,27**
0,604	0,573	**lysoPC a C17:0**	**0,62**	0,62	**0,60**
1,2	1,55	**lysoPC a C18:0**	**1,34**	1,85	**1,60**
62,2	51,7	**lysoPC a C18:1**	**46,37**	53,50	**57,53**
7,2	6,14	**lysoPC a C18:2**	**4,99**	5,84	**6,93**
2,59	2,08	**lysoPC a C20:3**	**1,87**	2,28	**2,58**
5,23	4,76	**lysoPC a C20:4**	**4,85**	5,45	**5,02**
1,03	0,989	**lysoPC a C24:0**	**1,04**	1,10	**1,05**
2,21	2,21	**lysoPC a C26:0**	**2,03**	2,27	**2,38**
0,58	0,489	**lysoPC a C26:1**	**0,48**	0,58	**0,50**
1,99	2,66	**lysoPC a C28:0**	**1,99**	2,27	**2,08**
1,1	1,36	**lysoPC a C28:1**	**1,06**	1,16	**1,17**
0,507	0,394	**PC aa C24:0**	**0,40**	0,47	**0,52**
3,56	2,83	**PC aa C26:0**	**2,38**	3,06	**3,23**
2	1,52	**PC aa C28:1**	**1,45**	1,61	**1,57**
12,6	11,9	**PC aa C30:0**	**11,49**	11,35	**11,70**
0,414	0,338	**PC aa C30:2**	**0,22**	0,23	**0,27**
14,1	19,6	**PC aa C32:0**	**27,73**	22,93	**19,77**
86,2	87,1	**PC aa C32:1**	**74,10**	71,87	**70,77**
5,17	5,41	**PC aa C32:2**	**5,62**	5,53	**4,52**
0,356	0,38	**PC aa C32:3**	**0,40**	0,38	**0,29**
302	315	**PC aa C34:1**	**263,67**	250,33	**256,67**
52,1	50,8	**PC aa C34:2**	**46,10**	46,53	**43,50**
3,33	3,36	**PC aa C34:3**	**3,27**	3,21	**2,90**
0,431	0,398	**PC aa C34:4**	**0,41**	0,38	**0,35**
2,35	2,68	**PC aa C36:0**	**2,41**	2,27	**2,26**
26,4	27,9	**PC aa C36:1**	**28,10**	25,23	**27,47**
91	82	**PC aa C36:2**	**74,90**	71,00	**73,43**
17	17,1	**PC aa C36:3**	**14,04**	13,78	**14,47**

8,91	8,97	**PC aa C36:4**	**8,47**	8,39	**7,72**
3,26	3,61	**PC aa C36:5**	**3,12**	3,01	**3,05**
0,459	0,569	**PC aa C36:6**	**0,49**	0,42	**0,43**
3,42	3,56	**PC aa C38:0**	**3,10**	2,90	**2,88**
1,21	1,05	**PC aa C38:1**	**1,16**	1,16	**1,21**
6,7	6,07	**PC aa C38:3**	**5,94**	5,72	**5,51**
11,1	11,1	**PC aa C38:4**	**9,66**	9,32	**9,53**
14,4	15,6	**PC aa C38:5**	**12,68**	12,37	**12,67**
6,21	6,68	**PC aa C38:6**	**5,58**	5,59	**5,76**
0,404	0,385	**PC aa C40:1**	**0,41**	0,40	**0,40**
0,699	0,598	**PC aa C40:2**	**0,68**	0,61	**0,63**
0,672	0,593	**PC aa C40:3**	**0,65**	0,63	**0,58**
1,07	0,96	**PC aa C40:4**	**0,90**	0,91	**0,89**
3,08	2,9	**PC aa C40:5**	**2,77**	2,59	**2,47**
6,88	7,15	**PC aa C40:6**	**5,88**	5,51	**5,80**
0,132	0,117	**PC aa C42:0**	**0,15**	0,14	**0,12**
0,14	0,126	**PC aa C42:1**	**0,13**	0,13	**0,13**
0,226	0,199	**PC aa C42:2**	**0,25**	0,23	**0,23**
0,24	0,209	**PC aa C42:4**	**0,23**	0,18	**0,19**
0,22	0,233	**PC aa C42:5**	**0,24**	0,23	**0,23**
0,497	0,469	**PC aa C42:6**	**0,50**	0,45	**0,47**
6,8	7,27	**PC ae C30:0**	**6,58**	6,40	**6,81**
3,54	3,49	**PC ae C30:1**	**2,86**	2,99	**3,07**
0,448	0,358	**PC ae C30:2**	**0,32**	0,34	**0,33**
49,6	50,6	**PC ae C32:1**	**45,63**	43,33	**44,13**
9,63	9,6	**PC ae C32:2**	**7,92**	7,97	**8,02**
2,18	2,62	**PC ae C34:0**	**3,40**	3,01	**2,80**
68,6	68	**PC ae C34:1**	**62,60**	59,47	**59,33**
26,5	26,1	**PC ae C34:2**	**22,23**	22,27	**22,47**
2,66	2,87	**PC ae C34:3**	**2,26**	2,41	**2,32**
1,83	2,06	**PC ae C36:0**	**2,22**	1,61	**1,61**
15	14,6	**PC ae C36:1**	**14,44**	14,02	**14,23**
19,5	17,4	**PC ae C36:2**	**15,40**	15,28	**15,73**
12	11,1	**PC ae C36:3**	**9,43**	9,38	**9,89**
13,4	12,8	**PC ae C36:4**	**11,17**	11,09	**10,76**
5,86	6,21	**PC ae C36:5**	**5,14**	5,25	**4,99**
1,02	1,19	**PC ae C38:0**	**0,93**	0,85	**0,92**

2,72	2,4	**PC ae C38:1**	**2,78**	2,78	**2,70**
5,11	4,32	**PC ae C38:2**	**4,33**	4,43	**4,40**
3,44	3,21	**PC ae C38:3**	**3,17**	3,22	**3,12**
7,15	6,88	**PC ae C38:4**	**6,13**	6,00	**6,00**
14,1	14	**PC ae C38:5**	**12,28**	12,15	**11,90**
9,82	10	**PC ae C38:6**	**8,65**	8,56	**8,33**
0,989	1,05	**PC ae C40:1**	**0,90**	0,89	**0,88**
0,894	0,747	**PC ae C40:2**	**0,82**	0,88	**0,83**
1,17	0,949	**PC ae C40:3**	**1,01**	0,97	**0,94**
1,51	1,42	**PC ae C40:4**	**1,44**	1,35	**1,28**
3,89	3,36	**PC ae C40:5**	**3,33**	3,25	**3,25**
5,02	4,95	**PC ae C40:6**	**4,22**	4,32	**4,37**
0,7	0,755	**PC ae C42:0**	**0,77**	0,70	**0,70**
0,476	0,466	**PC ae C42:1**	**0,42**	0,40	**0,43**
0,67	0,73	**PC ae C42:2**	**0,62**	0,58	**0,55**
0,619	0,646	**PC ae C42:3**	**0,68**	0,65	**0,62**
0,295	0,238	**PC ae C42:4**	**0,27**	0,28	**0,27**
0,794	0,814	**PC ae C42:5**	**0,81**	0,81	**0,80**
0,223	0,264	**PC ae C44:3**	**0,24**	0,24	**0,22**
0,265	0,205	**PC ae C44:4**	**0,24**	0,22	**0,25**
0,163	0,227	**PC ae C44:5**	**0,21**	0,21	**0,21**
0,141	0,125	**PC ae C44:6**	**0,15**	0,14	**0,16**

		**Biological replica:**	**3 independent cell cultures**		
11 - SKOV FASN2 24h re G28UCM	12 - SKOV FASN3 24h rpl G28UCM		**SKOV cont 24h control**	SKOV cont repl 24h control	**SKOV G28UCM 24h** G28UCM
10,6	7,67	**lysoPC a C14:0**	**10,57**	14,23	**10,63**
8,73	9,4	**lysoPC a C16:0**	**12,73**	15,93	**8,89**
17,3	16,9	**lysoPC a C16:1**	**22,70**	27,43	**21,20**
0,515	0,527	**lysoPC a C17:0**	**0,61**	0,64	**0,58**
1,28	1,33	**lysoPC a C18:0**	**1,91**	2,48	**1,16**
49,1	49,3	**lysoPC a C18:1**	**57,97**	66,70	**55,50**

4,73	5,24	**lysoPC a C18:2**	**5,58**	6,43	**6,21**
1,58	1,9	**lysoPC a C20:3**	**2,40**	2,63	**1,97**
3,07	3,35	**lysoPC a C20:4**	**5,15**	5,81	**3,56**
0,841	1,56	**lysoPC a C24:0**	**1,67**	1,58	**0,97**
2,15	3,68	**lysoPC a C26:0**	**3,27**	3,41	**2,23**
0,491	0,789	**lysoPC a C26:1**	**0,72**	0,66	**0,51**
2,21	3,46	**lysoPC a C28:0**	**2,78**	2,79	**2,15**
1,14	1,87	**lysoPC a C28:1**	**1,45**	1,38	**1,27**
0,294	0,367	**PC aa C24:0**	**0,63**	0,79	**0,37**
2,03	2,65	**PC aa C26:0**	**3,88**	4,62	**2,85**
1,15	1,61	**PC aa C28:1**	**2,03**	2,07	**1,66**
13,3	13,5	**PC aa C30:0**	**17,37**	18,83	**12,33**
0,236	0,371	**PC aa C30:2**	**0,39**	0,46	**0,37**
26,4	26,1	**PC aa C32:0**	**36,30**	40,23	**19,40**
98,5	111	**PC aa C32:1**	**99,33**	108,47	**76,37**
6,69	7,38	**PC aa C32:2**	**8,02**	8,69	**4,97**
0,436	0,495	**PC aa C32:3**	**0,56**	0,59	**0,33**
315	337	**PC aa C34:1**	**317,33**	358,00	**256,67**
56,8	58,9	**PC aa C34:2**	**61,20**	68,03	**45,07**
3,75	3,85	**PC aa C34:3**	**4,45**	4,97	**2,77**
0,41	0,415	**PC aa C34:4**	**0,50**	0,54	**0,30**
3,16	2,6	**PC aa C36:0**	**3,12**	3,37	**2,49**
23,8	25	**PC aa C36:1**	**27,67**	30,57	**20,50**
90,8	92,9	**PC aa C36:2**	**93,40**	104,00	**74,80**
17,6	17,1	**PC aa C36:3**	**17,33**	19,67	**13,33**
7,85	7,9	**PC aa C36:4**	**10,77**	12,00	**6,10**
2,95	3,17	**PC aa C36:5**	**3,22**	3,74	**2,31**
0,481	0,413	**PC aa C36:6**	**0,46**	0,53	**0,36**
4,42	4,22	**PC aa C38:0**	**4,52**	4,87	**3,69**
1,06	1,11	**PC aa C38:1**	**1,61**	1,79	**1,06**
5,95	6,29	**PC aa C38:3**	**7,82**	8,76	**5,13**
8,54	8,54	**PC aa C38:4**	**9,25**	10,20	**6,85**
10,6	10,9	**PC aa C38:5**	**11,27**	13,40	**8,33**
5,24	5,46	**PC aa C38:6**	**5,81**	6,67	**4,12**
0,401	0,456	**PC aa C40:1**	**0,52**	0,56	**0,40**
0,768	0,768	**PC aa C40:2**	**1,00**	1,06	**0,68**
0,603	0,631	**PC aa C40:3**	**0,96**	1,03	**0,57**

0,795	0,788	**PC aa C40:4**	**1,13**	1,22	**0,74**
2,25	2,24	**PC aa C40:5**	**2,48**	2,88	**1,83**
4,7	4,73	**PC aa C40:6**	**4,34**	4,86	**3,80**
0,151	0,122	**PC aa C42:0**	**0,19**	0,20	**0,13**
0,09	0,111	**PC aa C42:1**	**0,19**	0,19	**0,08**
0,257	0,263	**PC aa C42:2**	**0,34**	0,33	**0,23**
0,28	0,301	**PC aa C42:4**	**0,32**	0,33	**0,25**
0,215	0,218	**PC aa C42:5**	**0,28**	0,35	**0,18**
0,442	0,446	**PC aa C42:6**	**0,53**	0,61	**0,38**
8,29	9,08	**PC ae C30:0**	**9,64**	10,27	**7,98**
3,33	3,74	**PC ae C30:1**	**4,13**	4,25	**3,72**
0,299	0,363	**PC ae C30:2**	**0,46**	0,49	**0,39**
69,7	74	**PC ae C32:1**	**65,17**	71,00	**54,57**
11,6	12,4	**PC ae C32:2**	**12,57**	13,40	**9,85**
3,88	3,55	**PC ae C34:0**	**4,31**	4,75	**2,81**
86	92,2	**PC ae C34:1**	**86,07**	96,07	**67,10**
30,9	32,7	**PC ae C34:2**	**32,70**	35,60	**25,63**
3,22	3,38	**PC ae C34:3**	**3,57**	3,96	**2,70**
2,68	2,16	**PC ae C36:0**	**1,84**	1,96	**1,72**
17,1	18,2	**PC ae C36:1**	**20,17**	22,63	**14,33**
21,7	22,4	**PC ae C36:2**	**23,60**	25,93	**18,90**
13,5	14,2	**PC ae C36:3**	**14,97**	16,57	**11,70**
15	15,7	**PC ae C36:4**	**17,20**	18,93	**12,50**
6,85	7,05	**PC ae C36:5**	**7,46**	8,16	**5,61**
0,882	0,76	**PC ae C38:0**	**0,81**	1,00	**0,65**
2,88	2,68	**PC ae C38:1**	**4,34**	4,65	**2,54**
5,13	5,33	**PC ae C38:2**	**6,85**	7,59	**4,60**
4,14	4,15	**PC ae C38:3**	**5,55**	6,12	**3,53**
8,6	8,43	**PC ae C38:4**	**9,56**	10,67	**7,15**
18	18,3	**PC ae C38:5**	**19,07**	20,63	**15,03**
12,5	12,3	**PC ae C38:6**	**13,10**	14,53	**10,11**
0,48	0,461	**PC ae C40:1**	**0,83**	0,92	**0,42**
0,719	0,757	**PC ae C40:2**	**1,46**	1,52	**0,76**
1	1	**PC ae C40:3**	**1,57**	1,72	**0,98**
1,56	1,55	**PC ae C40:4**	**2,17**	2,35	**1,43**
4,33	4,27	**PC ae C40:5**	**4,93**	5,39	**3,79**
6,58	6,57	**PC ae C40:6**	**6,79**	7,34	**5,56**

0,692	0,705	**PC ae C42:0**	**0,67**	0,72	**0,59**
0,249	0,215	**PC ae C42:1**	**0,29**	0,32	**0,20**
0,221	0,24	**PC ae C42:2**	**0,29**	0,33	**0,19**
0,276	0,253	**PC ae C42:3**	**0,43**	0,51	**0,23**
0,185	0,241	**PC ae C42:4**	**0,38**	0,43	**0,19**
0,861	0,762	**PC ae C42:5**	**1,08**	1,13	**0,77**
0,081	0,116	**PC ae C44:3**	**0,16**	0,22	**0,09**
0,121	0,132	**PC ae C44:4**	**0,16**	0,17	**0,11**
0,162	0,152	**PC ae C44:5**	**0,23**	0,26	**0,13**
0,119	0,127	**PC ae C44:6**	**0,22**	0,28	**0,14**

Supplemental Table S1

SKOV FASN repl 8h G28UCM	SD SKOV cont 8h control	SKOV G28UCM 8h G28UCM	Biological replica CV (%) SKOV cont 8h control	SKOV G28UCM 8h G28UCM	Technical replica:	2 measurements SKOV cont 8h vs. replica control
8,77	0,7618	2,1910	9,1340	20,3878	**lysoPC a C14:0**	9,04
8,05	1,3816	2,7626	17,0083	28,8576	**lysoPC a C16:0**	9,08
18,43	2,2605	5,9138	13,2195	26,5591	**lysoPC a C16:1**	18,55
0,54	0,0545	0,0866	8,7491	14,4666	**lysoPC a C17:0**	0,62
1,31	0,4530	0,6136	33,7591	38,3244	**lysoPC a C18:0**	1,60
51,07	6,1232	12,6982	13,2060	22,0710	**lysoPC a C18:1**	49,93
5,76	0,2757	2,3438	5,5296	33,8375	**lysoPC a C18:2**	5,41
2,11	0,1966	0,7862	10,5297	30,5104	**lysoPC a C20:3**	2,07
4,40	0,4155	1,4406	8,5727	28,6775	**lysoPC a C20:4**	5,15
0,94	0,2852	0,4071	27,3746	38,8706	**lysoPC a C24:0**	1,07
2,09	0,6987	0,7908	34,4772	33,2252	**lysoPC a C26:0**	2,15
0,49	0,1512	0,2068	31,2700	41,1727	**lysoPC a C26:1**	0,53
2,01	0,6790	0,8017	34,0633	38,5426	**lysoPC a C28:0**	2,13
1,10	0,2853	0,3366	26,9969	28,7015	**lysoPC a C28:1**	1,11
0,42	0,1172	0,2377	29,0439	45,6269	**PC aa C24:0**	0,44
2,87	0,4452	1,0300	18,7335	31,8562	**PC aa C26:0**	2,72
1,55	0,2558	0,4177	17,6820	26,5457	**PC aa C28:1**	1,53
11,63	1,9422	0,6928	16,8985	5,9215	**PC aa C30:0**	11,42
0,32	0,1126	0,1015	51,1116	37,4443	**PC aa C30:2**	0,23
17,27	10,4543	3,4646	37,6960	17,5274	**PC aa C32:0**	25,33
74,13	26,3232	15,5565	35,5239	21,9827	**PC aa C32:1**	72,98
4,57	2,1664	1,1837	38,5481	26,1696	**PC aa C32:2**	5,58
0,30	0,1405	0,0641	35,3330	21,8616	**PC aa C32:3**	0,39
272,67	91,8767	42,7122	34,8458	16,6411	**PC aa C34:1**	257,00
44,37	16,5012	10,2132	35,7944	23,4787	**PC aa C34:2**	46,32
2,87	1,1962	0,6964	36,5798	23,9852	**PC aa C34:3**	3,24
0,36	0,1195	0,0666	28,8083	19,2983	**PC aa C34:4**	0,40
2,23	0,7994	0,3707	33,1241	16,3794	**PC aa C36:0**	2,34
26,70	9,7872	2,9366	34,8300	10,6913	**PC aa C36:1**	26,67
77,23	27,5610	17,4517	36,7971	23,7654	**PC aa C36:2**	72,95
14,90	4,8422	3,5726	34,4802	24,6953	**PC aa C36:3**	13,91

7,66	3,0280	1,7475	35,7359	22,6262	**PC aa C36:4**	8,43
2,92	1,0355	0,7136	33,1533	23,3961	**PC aa C36:5**	3,07
0,42	0,1044	0,0627	21,3992	14,7508	**PC aa C36:6**	0,45
3,09	1,0536	0,7382	33,9860	25,6310	**PC aa C38:0**	3,00
1,09	0,3926	0,1617	33,7477	13,3235	**PC aa C38:1**	1,16
5,83	2,3592	0,8844	39,7391	16,0613	**PC aa C38:3**	5,83
9,80	3,2371	2,0616	33,4991	21,6402	**PC aa C38:4**	9,49
12,90	4,4295	3,3650	34,9423	26,5659	**PC aa C38:5**	12,53
5,54	1,9263	1,3267	34,5009	23,0190	**PC aa C38:6**	5,59
0,40	0,1131	0,0157	27,4225	3,9788	**PC aa C40:1**	0,40
0,64	0,2643	0,1289	38,6082	20,4132	**PC aa C40:2**	0,65
0,60	0,2740	0,0844	42,2840	14,6674	**PC aa C40:3**	0,64
0,93	0,3435	0,1567	38,2922	17,5751	**PC aa C40:4**	0,90
2,73	0,9496	0,4551	34,3228	18,4244	**PC aa C40:5**	2,68
6,16	1,9206	1,3462	32,6810	23,2229	**PC aa C40:6**	5,70
0,12	0,0441	0,0129	29,0883	10,4256	**PC aa C42:0**	0,14
0,13	0,0493	0,0025	38,4959	1,9764	**PC aa C42:1**	0,13
0,21	0,0930	0,0161	37,6584	7,0909	**PC aa C42:2**	0,24
0,20	0,0655	0,0240	27,9187	12,6131	**PC aa C42:4**	0,21
0,22	0,0925	0,0490	37,8428	21,2221	**PC aa C42:5**	0,24
0,46	0,1250	0,0283	25,0177	6,0022	**PC aa C42:6**	0,47
6,63	1,0431	0,6843	15,8444	10,0488	**PC ae C30:0**	6,49
3,15	0,3113	0,5853	10,8734	19,0847	**PC ae C30:1**	2,93
0,35	0,0293	0,0874	9,2464	26,6692	**PC ae C30:2**	0,33
44,50	16,9771	7,6950	37,2034	17,4358	**PC ae C32:1**	44,48
8,08	3,0001	2,0077	37,8796	25,0440	**PC ae C32:2**	7,95
2,46	1,3848	0,5220	40,7690	18,6668	**PC ae C34:0**	3,21
61,93	23,6006	9,0279	37,7007	15,2156	**PC ae C34:1**	61,03
23,03	7,9908	4,3879	35,9408	19,5305	**PC ae C34:2**	22,25
2,36	0,7150	0,5524	31,5908	23,8125	**PC ae C34:3**	2,34
1,87	0,8310	0,3808	37,4880	23,7033	**PC ae C36:0**	1,91
14,37	5,5242	1,1547	38,2473	8,1127	**PC ae C36:1**	14,23
16,53	5,5435	2,7429	35,9965	17,4335	**PC ae C36:2**	15,34
10,16	3,4874	2,0007	36,9816	20,2223	**PC ae C36:3**	9,41
11,26	3,8527	2,5117	34,5018	23,3502	**PC ae C36:4**	11,13
5,08	1,5805	1,1265	30,7485	22,5760	**PC ae C36:5**	5,20
0,97	0,2433	0,1552	26,2379	16,7861	**PC ae C38:0**	0,89

2,67	1,1277	0,5020	40,5150	18,5707	**PC ae C38:1**	2,78
4,51	1,7779	0,9160	41,0917	20,8337	**PC ae C38:2**	4,38
3,15	1,2831	0,5575	40,4770	17,8684	**PC ae C38:3**	3,19
6,24	2,1494	1,1484	35,0835	19,1406	**PC ae C38:4**	6,07
12,39	4,2586	2,6851	34,6699	22,5642	**PC ae C38:5**	12,22
8,58	2,9494	1,7250	34,0970	20,7085	**PC ae C38:6**	8,60
0,91	0,2002	0,1427	22,3151	16,2511	**PC ae C40:1**	0,89
0,84	0,3531	0,0991	43,1623	11,9586	**PC ae C40:2**	0,85
1,00	0,4368	0,1199	43,3524	12,7449	**PC ae C40:3**	0,99
1,38	0,5246	0,1600	36,4917	12,5000	**PC ae C40:4**	1,39
3,35	1,2121	0,5892	36,3996	18,1304	**PC ae C40:5**	3,29
4,49	1,5672	0,9833	37,1662	22,5190	**PC ae C40:6**	4,27
0,68	0,1662	0,0830	21,6249	11,7746	**PC ae C42:0**	0,74
0,43	0,1475	0,0868	35,1245	20,1549	**PC ae C42:1**	0,41
0,60	0,1924	0,1170	31,1298	21,1360	**PC ae C42:2**	0,60
0,56	0,1849	0,1534	27,0999	24,8164	**PC ae C42:3**	0,66
0,25	0,0967	0,0295	36,1555	10,8503	**PC ae C42:4**	0,27
0,79	0,2398	0,0945	29,6136	11,7430	**PC ae C42:5**	0,81
0,21	0,0416	0,0665	17,5550	30,1328	**PC ae C44:3**	0,24
0,22	0,0825	0,0339	35,1083	13,8234	**PC ae C44:4**	0,23
0,19	0,0582	0,0263	27,7710	12,4130	**PC ae C44:5**	0,21
0,13	0,0402	0,0015	27,1036	0,9276	**PC ae C44:6**	0,14
			**30,7824**	**20,4662**	**mean CV (%)**	

SKOV G28UCM repl 24h	**SD**		**Biological replica CV (%)**		**Technical replica:**	**2 measurements (**
	**SKOV cont 24h**	**SKOV G28UCM 24h**	**SKOV cont 24h**	**SKOV G28UCM 24h**		**SKOV cont 24h vs. replica**
G28UCM	**control**	G28UCM	**control**	G28UCM		control
10,56	0,2517	1,4114	2,3817	13,2820	**lysoPC a C14:0**	12,40
9,74	0,7767	1,1774	6,1001	13,2442	**lysoPC a C16:0**	14,33
19,37	1,5133	2,5865	6,6664	12,2005	**lysoPC a C16:1**	25,07
0,52	0,0032	0,1231	0,5299	21,1829	**lysoPC a C17:0**	0,62
1,39	0,2281	0,2235	11,9220	19,3069	**lysoPC a C18:0**	2,20
55,00	1,6862	8,0467	2,9089	14,4986	**lysoPC a C18:1**	62,33

5,72	0,5085	0,6934	9,1068	11,1722	**lysoPC a C18:2**	6,01
1,91	0,0971	0,2615	4,0413	13,2758	**lysoPC a C20:3**	2,52
3,49	0,3101	0,4636	6,0166	13,0105	**lysoPC a C20:4**	5,48
1,14	0,1778	0,0476	10,6445	4,9300	**lysoPC a C24:0**	1,63
2,71	0,2581	0,1386	7,8860	6,2136	**lysoPC a C26:0**	3,34
0,59	0,0413	0,0674	5,7399	13,1592	**lysoPC a C26:1**	0,69
2,64	0,2639	0,2610	9,5035	12,1595	**lysoPC a C28:0**	2,79
1,37	0,1570	0,0902	10,7993	7,1199	**lysoPC a C28:1**	1,42
0,35	0,0920	0,0818	14,6255	22,3619	**PC aa C24:0**	0,71
2,50	0,1803	0,7239	4,6463	25,4298	**PC aa C26:0**	4,25
1,42	0,0737	0,3035	3,6371	18,2819	**PC aa C28:1**	2,05
13,17	0,3786	2,2008	2,1800	17,8440	**PC aa C30:0**	18,10
0,28	0,0357	0,0943	9,2235	25,6480	**PC aa C30:2**	0,42
22,67	3,2187	7,5027	8,8669	38,6735	**PC aa C32:0**	38,27
94,40	5,7770	10,3375	5,8157	13,5366	**PC aa C32:1**	103,90
6,19	0,6317	0,5263	7,8771	10,5974	**PC aa C32:2**	8,35
0,43	0,0120	0,0323	2,1634	9,6658	**PC aa C32:3**	0,57
306,67	12,0139	41,2957	3,7859	16,0892	**PC aa C34:1**	337,67
53,17	4,5398	3,8070	7,4180	8,4475	**PC aa C34:2**	64,62
3,44	0,3297	0,1960	7,4089	7,0859	**PC aa C34:3**	4,71
0,38	0,0320	0,0313	6,3860	10,5025	**PC aa C34:4**	0,52
2,68	0,1905	0,5009	6,1066	20,1164	**PC aa C36:0**	3,25
23,60	1,8771	6,2960	6,7845	30,7123	**PC aa C36:1**	29,12
86,07	3,9837	8,1462	4,2652	10,8906	**PC aa C36:2**	98,70
15,90	1,1015	1,4295	6,3549	10,7209	**PC aa C36:3**	18,50
7,30	1,1197	0,7535	10,3964	12,3518	**PC aa C36:4**	11,39
2,78	0,2230	0,2951	6,9329	12,7761	**PC aa C36:5**	3,48
0,42	0,0626	0,0576	13,5106	15,9327	**PC aa C36:6**	0,50
4,09	0,0971	0,5601	2,1472	15,1657	**PC aa C38:0**	4,70
1,11	0,0603	0,2691	3,7517	25,2867	**PC aa C38:1**	1,70
5,88	0,4099	0,6841	5,2442	13,3272	**PC aa C38:3**	8,29
7,99	0,6165	0,7263	6,6669	10,6028	**PC aa C38:4**	9,72
9,94	0,4041	1,1763	3,5871	14,1267	**PC aa C38:5**	12,33
5,00	0,3704	0,5781	6,3753	14,0436	**PC aa C38:6**	6,24
0,41	0,0694	0,1104	13,4533	27,3010	**PC aa C40:1**	0,54
0,79	0,0659	0,0917	6,5567	13,5081	**PC aa C40:2**	1,03
0,62	0,0692	0,0809	7,2101	14,1648	**PC aa C40:3**	0,99

0,77	0,0404	0,1580	3,5660	21,3097	**PC aa C40:4**	1,18
2,16	0,1473	0,2961	5,9399	16,1826	**PC aa C40:5**	2,68
4,38	0,1570	0,5179	3,6191	13,6412	**PC aa C40:6**	4,60
0,14	0,0093	0,0226	4,9075	17,6949	**PC aa C42:0**	0,19
0,10	0,0091	0,0232	4,7341	27,7830	**PC aa C42:1**	0,19
0,27	0,0190	0,0181	5,6548	7,7443	**PC aa C42:2**	0,33
0,28	0,0095	0,0492	3,0093	19,8786	**PC aa C42:4**	0,32
0,21	0,0029	0,0299	1,0141	16,9276	**PC aa C42:5**	0,32
0,43	0,0482	0,0646	9,1481	17,1184	**PC aa C42:6**	0,57
8,48	0,6134	1,4654	6,3650	18,3638	**PC ae C30:0**	9,95
3,60	0,1858	0,4153	4,4959	11,1527	**PC ae C30:1**	4,19
0,36	0,0101	0,0669	2,1928	17,2656	**PC ae C30:2**	0,48
66,23	2,8746	8,3500	4,4112	15,3025	**PC ae C32:1**	68,08
11,37	0,7506	1,1731	5,9726	11,9135	**PC ae C32:2**	12,98
3,49	0,1168	0,7738	2,7070	27,5381	**PC ae C34:0**	4,53
82,23	3,9004	13,6451	4,5319	20,3355	**PC ae C34:1**	91,07
30,37	1,7776	3,6950	5,4362	14,4150	**PC ae C34:2**	34,15
3,12	0,2848	0,3528	7,9770	13,0487	**PC ae C34:3**	3,76
2,15	0,2570	0,4979	13,9405	28,9474	**PC ae C36:0**	1,90
16,63	1,0017	3,7072	4,9669	25,8642	**PC ae C36:1**	21,40
21,07	0,8718	2,5632	3,6940	13,5619	**PC ae C36:2**	24,77
13,20	0,2082	1,4731	1,3909	12,5905	**PC ae C36:3**	15,77
14,53	0,6245	1,5133	3,6308	12,1062	**PC ae C36:4**	18,07
6,50	0,2400	0,7920	3,2172	14,1257	**PC ae C36:5**	7,81
0,75	0,0677	0,1702	8,3212	26,2281	**PC ae C38:0**	0,91
2,76	0,3980	0,6466	9,1643	25,4246	**PC ae C38:1**	4,50
5,09	0,3005	0,7910	4,3869	17,1839	**PC ae C38:2**	7,22
4,01	0,2303	0,5710	4,1469	16,1907	**PC ae C38:3**	5,84
8,19	0,4813	1,0080	5,0361	14,0982	**PC ae C38:4**	10,12
17,10	0,6351	2,3159	3,3309	15,4050	**PC ae C38:5**	19,85
11,80	0,4359	1,4171	3,3274	14,0173	**PC ae C38:6**	13,82
0,47	0,0727	0,0948	8,7858	22,5128	**PC ae C40:1**	0,88
0,77	0,1002	0,1539	6,8764	20,3175	**PC ae C40:2**	1,49
0,99	0,1300	0,2488	8,2803	25,2849	**PC ae C40:3**	1,65
1,53	0,1229	0,2511	5,6628	17,5980	**PC ae C40:4**	2,26
4,21	0,2339	0,6201	4,7440	16,3473	**PC ae C40:5**	5,16
6,24	0,1868	0,7203	2,7513	12,9546	**PC ae C40:6**	7,07

0,66	0,0131	0,0742	1,9574	12,6127	**PC ae C42:0**	0,69
0,22	0,0135	0,0368	4,6809	18,4450	**PC ae C42:1**	0,30
0,22	0,0341	0,0298	11,8140	15,6105	**PC ae C42:2**	0,31
0,27	0,0342	0,0491	7,9014	21,5734	**PC ae C42:3**	0,47
0,21	0,0205	0,0277	5,3344	14,6212	**PC ae C42:4**	0,41
0,81	0,0794	0,1228	7,3493	16,0293	**PC ae C42:5**	1,11
0,10	0,0242	0,0110	15,3473	12,0879	**PC ae C44:3**	0,19
0,12	0,0160	0,0035	10,1546	3,1492	**PC ae C44:4**	0,17
0,15	0,0268	0,0208	11,6031	16,1307	**PC ae C44:5**	0,24
0,12	0,0327	0,0356	15,0674	25,8389	**PC ae C44:6**	0,25
			**6,3352**	**16,3825**	**mean CV (%)**	

of same sample	SD		Technical replica CV (%)	
SKOV G28UCM 8h vs. replica	SKOV cont 8h	SKOV G28UCM 8h	SKOV cont 8h	SKOVG28UCM 8h
G28UCM	control	G28UCM	control	G28UCM
9,76	0,9876	1,3977	10,9267	14,3233
8,81	1,3482	1,0772	14,8537	12,2242
20,35	2,0506	2,7106	11,0545	13,3198
0,57	0,0012	0,0410	0,1894	7,2035
1,46	0,3616	0,2058	22,6310	14,1373
54,30	5,0440	4,5726	10,1015	8,4210
6,35	0,6010	0,8226	11,1064	12,9646
2,34	0,2923	0,3300	14,0967	14,0818
4,71	0,4290	0,4384	8,3297	9,3014
0,99	0,0403	0,0766	3,7651	7,7130
2,23	0,1721	0,2074	8,0091	9,2874
0,49	0,0674	0,0120	12,6871	2,4342
2,05	0,1956	0,0495	9,1775	2,4204
1,13	0,0705	0,0547	6,3692	4,8221
0,47	0,0493	0,0721	11,2342	15,3457
3,05	0,4832	0,2593	17,7752	8,5007
1,56	0,1131	0,0141	7,4107	0,9046
11,67	0,1014	0,0471	0,8874	0,4041
0,29	0,0068	0,0335	3,0357	11,3585
18,52	3,3941	1,7678	13,3978	9,5469
72,45	1,5792	2,3806	2,1638	3,2858
4,55	0,0613	0,0306	1,0989	0,6742
0,30	0,0106	0,0064	2,7185	2,1368
264,67	9,4281	11,3137	3,6685	4,2747
43,93	0,3064	0,6128	0,6616	1,3949
2,89	0,0448	0,0259	1,3829	0,8987
0,35	0,0229	0,0106	5,7373	3,0061
2,25	0,1014	0,0212	4,3282	0,9435
27,08	2,0270	0,5421	7,6014	2,0017
75,33	2,7577	2,6870	3,7803	3,5668
14,68	0,1862	0,3064	1,3385	2,0868

7,69	0,0589	0,0471	0,6989	0,6130
2,99	0,0825	0,0919	2,6915	3,0795
0,42	0,0495	0,0016	10,9346	0,3890
2,99	0,1414	0,1508	4,7140	5,0508
1,15	0,0002	0,0896	0,0203	7,7884
5,67	0,1556	0,2263	2,6699	3,9931
9,66	0,2404	0,1933	2,5325	2,0001
12,79	0,2145	0,1673	1,7125	1,3089
5,65	0,0047	0,1556	0,0844	2,7517
0,40	0,0106	0,0028	2,6200	0,7125
0,64	0,0530	0,0047	8,1946	0,7424
0,59	0,0113	0,0184	1,7678	3,1231
0,91	0,0061	0,0255	0,6799	2,7984
2,60	0,1273	0,1862	4,7551	7,1571
5,98	0,2569	0,2546	4,5112	4,2592
0,12	0,0099	0,0009	6,8430	0,7686
0,13	0,0007	0,0009	0,5546	0,7366
0,22	0,0108	0,0090	4,5302	4,0651
0,20	0,0379	0,0094	18,2589	4,7858
0,23	0,0123	0,0064	5,2008	2,8138
0,46	0,0387	0,0097	8,1839	2,0805
6,72	0,1296	0,1249	1,9970	1,8585
3,11	0,0896	0,0613	3,0604	1,9705
0,34	0,0158	0,0146	4,8171	4,3235
44,32	1,6263	0,2593	3,6561	0,5850
8,05	0,0377	0,0424	0,4746	0,5273
2,63	0,2711	0,2404	8,4573	9,1529
60,63	2,2156	1,8385	3,6301	3,0321
22,75	0,0236	0,4007	0,1059	1,7613
2,34	0,1037	0,0283	4,4383	1,2087
1,74	0,4313	0,1838	22,5633	10,5862
14,30	0,2993	0,0943	2,1034	0,6593
16,13	0,0872	0,5657	0,5686	3,5063
10,03	0,0354	0,1909	0,3759	1,9038
11,01	0,0566	0,3559	0,5084	3,2331
5,03	0,0778	0,0613	1,4972	1,2175
0,95	0,0563	0,0311	6,3474	3,2877

2,69	0,0024	0,0212	0,0847	0,7891
4,45	0,0707	0,0801	1,6156	1,7995
3,14	0,0330	0,0212	1,0334	0,6767
6,12	0,0872	0,1673	1,4379	2,7352
12,14	0,0919	0,3441	0,7523	2,8339
8,46	0,0660	0,1791	0,7671	2,1183
0,90	0,0066	0,0250	0,7393	2,7895
0,83	0,0460	0,0052	5,4041	0,6233
0,97	0,0240	0,0443	2,4268	4,5573
1,33	0,0629	0,0707	4,5172	5,3166
3,30	0,0589	0,0707	1,7920	2,1427
4,43	0,0754	0,0896	1,7664	2,0218
0,69	0,0469	0,0160	6,3773	2,3117
0,43	0,0174	0,0033	4,2785	0,7704
0,58	0,0283	0,0358	4,7298	6,1877
0,59	0,0252	0,0382	3,7954	6,4572
0,26	0,0097	0,0130	3,5248	4,9323
0,80	0,0009	0,0120	0,1165	1,5105
0,21	0,0002	0,0094	0,0995	4,4056
0,23	0,0111	0,0210	4,8766	9,1140
0,20	0,0019	0,0127	0,8937	6,2802
0,15	0,0066	0,0219	4,5937	14,6952
			**4,9322**	**4,4209 mean CV (%)**

**of same sample**	**SD**		**Technical replica CV (%)**	
**SKOV G28UCM 24h vs. replica**	**SKOV cont 24h**	**SKOV G28UCM 24h**	**SKOV cont 24h**	**SKOV G28UCM 24h**
G28UCM	control	G28UCM	control	G28UCM
10,59	2,5927	0,0495	20,9091	0,4673
9,32	2,2627	0,6034	15,7866	6,4765
20,28	3,3470	1,2964	13,3523	6,3913
0,55	0,0203	0,0436	3,2642	7,9210
1,28	0,3983	0,1666	18,1475	13,0648
55,25	6,1754	0,3536	9,9071	0,6399

5,96	0,5987	0,3465	9,9670	5,8118
1,94	0,1579	0,0448	6,2791	2,3104
3,53	0,4667	0,0495	8,5111	1,4029
1,05	0,0613	0,1233	3,7674	11,7049
2,47	0,0990	0,3394	2,9610	13,7413
0,55	0,0389	0,0563	5,6187	10,2021
2,39	0,0118	0,3465	0,4232	14,4871
1,32	0,0542	0,0731	3,8312	5,5424
0,36	0,1122	0,0134	15,8392	3,7721
2,68	0,5233	0,2428	12,3120	9,0756
1,54	0,0306	0,1721	1,4959	11,1850
12,75	1,0371	0,5893	5,7298	4,6216
0,33	0,0500	0,0592	11,8223	18,1569
21,03	2,7813	2,3099	7,2682	10,9820
85,38	6,4582	12,7515	6,2158	14,9344
5,58	0,4714	0,8650	5,6433	15,5069
0,38	0,0269	0,0658	4,6785	17,2676
281,67	28,7557	35,3553	8,5160	12,5522
49,12	4,8319	5,7276	7,4778	11,6611
3,11	0,3677	0,4785	7,8067	15,4098
0,34	0,0288	0,0554	5,5088	16,4443
2,59	0,1768	0,1367	5,4477	5,2851
22,05	2,0506	2,1920	7,0427	9,9412
80,43	7,4953	7,9667	7,5941	9,9048
14,62	1,6499	1,8149	8,9185	12,4167
6,70	0,8697	0,8462	7,6394	12,6326
2,55	0,3677	0,3323	10,5761	13,0586
0,39	0,0493	0,0396	9,8952	10,1707
3,89	0,2475	0,2781	5,2676	7,1498
1,09	0,1320	0,0349	7,7643	3,2043
5,51	0,6647	0,5280	8,0211	9,5879
7,42	0,6718	0,8085	6,9098	10,8932
9,13	1,5085	1,1384	12,2310	12,4670
4,56	0,6058	0,6270	9,7102	13,7493
0,41	0,0278	0,0071	5,1922	1,7275
0,73	0,0365	0,0773	3,5464	10,5423
0,59	0,0481	0,0311	4,8374	5,2437

0,75	0,0613	0,0189	5,2082	2,4986
1,99	0,2852	0,2310	10,6352	11,5880
4,09	0,3724	0,4125	8,0959	10,0892
0,13	0,0052	0,0052	2,6868	3,9383
0,09	0,0016	0,0080	0,8556	8,9708
0,25	0,0047	0,0250	1,4170	9,9145
0,26	0,0068	0,0224	2,1239	8,5086
0,19	0,0453	0,0245	14,2910	12,6356
0,40	0,0556	0,0342	9,8279	8,5123
8,23	0,4478	0,3559	4,4993	4,3237
3,66	0,0849	0,0849	2,0235	2,3163
0,37	0,0203	0,0214	4,2615	5,7633
60,40	4,1248	8,2496	6,0584	13,6582
10,61	0,5893	1,0748	4,5386	10,1333
3,15	0,3111	0,4832	6,8631	15,3312
74,67	7,0711	10,7009	7,7647	14,3315
28,00	2,0506	3,3470	6,0047	11,9535
2,91	0,2734	0,2923	7,2652	10,0437
1,94	0,0849	0,3041	4,4581	15,7135
15,48	1,7442	1,6263	8,1505	10,5038
19,98	1,6499	1,5321	6,6618	7,6667
12,45	1,1314	1,0607	7,1757	8,5194
13,52	1,2257	1,4378	6,7841	10,6371
6,05	0,4973	0,6293	6,3665	10,3992
0,70	0,1287	0,0745	14,2203	10,6150
2,65	0,2192	0,1508	4,8730	5,6924
4,85	0,5256	0,3441	7,2783	7,1002
3,77	0,4031	0,3441	6,9035	9,1280
7,67	0,7896	0,7330	7,8063	9,5592
16,07	1,1078	1,4614	5,5809	9,0956
10,96	1,0135	1,1950	7,3355	10,9084
0,45	0,0679	0,0358	7,7491	8,0269
0,76	0,0471	0,0080	3,1638	1,0503
0,99	0,1061	0,0028	6,4478	0,2869
1,48	0,1249	0,0707	5,5316	4,7885
4,00	0,3253	0,2970	6,3037	7,4184
5,90	0,3889	0,4785	5,5047	8,1120

0,62	0,0349	0,0483	5,0217	7,7621	
0,21	0,0222	0,0127	7,2722	6,0996	
0,20	0,0302	0,0174	9,7427	8,5780	
0,25	0,0554	0,0285	11,7228	11,5077	
0,20	0,0330	0,0151	8,0944	7,5425	
0,79	0,0354	0,0342	3,1996	4,3253	
0,09	0,0424	0,0054	22,5672	5,7165	
0,12	0,0108	0,0085	6,5578	7,3149	
0,14	0,0184	0,0156	7,5451	11,1117	
0,13	0,0460	0,0115	18,3970	8,9185	
			**7,5163**	**8,9813**	**mean CV (%)**

Supplemental Table S2a: Shotgun proteomic analysis of SKOV3 cells exposed for 8h and 24h to 40µM G28UCM. All proteins identified by MS/MS.

p-Value	x	2^x	% of Control	Protein IDs	Protein Names	Gene names
**8 h Up**						
0,001	3,37	10,36	**1036,11**	Q9UPV0	**Centrosomal protein of 164 kDa**	CEP164
0,024	3,23	9,36	**935,99**	P42338;O00329	**Phosphatidylinositol 4,5-bisphosphate 3-kinase catalytic subunit beta isoform;Phos**	PIK3CB;PIK3CD
0,011	3,19	9,11	**911,37**	P05386	**60S acidic ribosomal protein P1**	RPLP1
0,001	3,04	8,20	**819,72**	Q9NV56	**MRG-binding protein**	MRGBP
0,011	3,02	8,13	**813,46**	Q5UIP0	**Telomere-associated protein RIF1**	RIF1
0,000	2,93	7,61	**761,42**	Q9UER7	**Death domain-associated protein 6**	DAXX
0,009	2,80	6,95	**695,39**	O00148	**ATP-dependent RNA helicase DDX39A**	DDX39A
0,003	2,76	6,77	**676,54**	Q3ZCM7	**Tubulin beta-8 chain**	TUBB8
0,000	2,73	6,65	**665,06**	Q16678	**Cytochrome P450 1B1**	CYP1B1
0,004	2,73	6,62	**661,99**	Q9Y2H5	**Pleckstrin homology domain-containing family A member 6**	PLEKHA6
0,030	2,65	6,27	**627,24**	Q9Y3B8	**Oligoribonuclease, mitochondrial**	REXO2
0,030	2,60	6,04	**604,32**	O43464	**Serine protease HTRA2, mitochondrial**	HTRA2
0,048	2,59	6,00	**600,29**	Q9UGU0	**Transcription factor 20**	TCF20
0,027	2,58	5,98	**597,99**	Q01780	**Exosome component 10**	EXOSC10
0,021	2,28	4,86	**485,94**	Q15021	**Condensin complex subunit 1**	NCAPD2
0,032	2,26	4,81	**480,52**	Q8NCA5	**Protein FAM98A**	FAM98A
0,044	2,25	4,77	**477,24**	Q12873	**Chromodomain-helicase-DNA-binding protein 3**	CHD3
0,016	2,23	4,69	**469,37**	O76027	**Annexin A9**	ANXA9
0,031	2,21	4,63	**463,27**	P51808	**Dynein light chain Tctex-type 3**	DYNLT3
0,002	2,20	4,58	**458,02**	O00411	**DNA-directed RNA polymerase, mitochondrial**	POLRMT
0,033	2,16	4,48	**448,02**	Q9NV88	**Integrator complex subunit 9**	INTS9
0,004	2,16	4,47	**447,08**	Q92575	**UBX domain-containing protein 4**	UBXN4
0,043	2,16	4,47	**446,78**	Q66PJ3	**ADP-ribosylation factor-like protein 6-interacting protein 4**	ARL6IP4
0,024	2,15	4,45	**445,36**	O00442	**RNA 3-terminal phosphate cyclase**	RTCD1
0,026	2,13	4,38	**437,83**	Q16629	**Serine/arginine-rich splicing factor 7**	SRSF7
0,002	2,09	4,26	**426,03**	Q15528	**Mediator of RNA polymerase II transcription subunit 22**	MED22
0,037	2,08	4,23	**422,59**	P15170;Q8IYD1	**Eukaryotic peptide chain release factor GTP-binding subunit ERF3A**	GSPT1

0,017	2,05	4,14	**414,44**	Q9GZR7	**ATP-dependent RNA helicase DDX24**	DDX24
0,001	2,04	4,12	**411,59**	Q15428	**Splicing factor 3A subunit 2**	SF3A2
0,018	2,04	4,11	**411,02**	Q9UGP8	**Translocation protein SEC63 homolog**	SEC63
0,012	2,03	4,09	**409,43**	Q13151	**Heterogeneous nuclear ribonucleoprotein A0**	HNRNPA0
0,017	2,02	4,06	**406,26**	Q05519	**Serine/arginine-rich splicing factor 11**	SRSF11
0,047	1,98	3,94	**394,15**	O43264	**Centromere/kinetochore protein zw10 homolog**	ZW10
0,020	1,96	3,88	**388,24**	Q9UMN6	**Histone-lysine N-methyltransferase MLL4**	WBP7
0,034	1,94	3,83	**383,32**	Q9Y2H1	**Serine/threonine-protein kinase 38-like**	STK38L
0,018	1,93	3,81	**380,71**	O95182	**NADH dehydrogenase [ubiquinone] 1 alpha subcomplex subunit 7**	NDUFA7
0,011	1,92	3,79	**378,87**	Q9GZS1	**DNA-directed RNA polymerase I subunit RPA49**	POLR1E
0,022	1,91	3,77	**377,08**	P62995	**Transformer-2 protein homolog beta**	TRA2B
0,016	1,89	3,71	**371,11**	Q8IY17	**Neuropathy target esterase**	PNPLA6
0,048	1,87	3,66	**365,82**	P04350;A6NNZ2	**Tubulin beta-4A chain**	TUBB4A
0,022	1,86	3,62	**361,93**	Q9NVH0	**Exonuclease 3-5 domain-containing protein 2**	EXD2
0,037	1,83	3,55	**354,67**	Q8IYU8	**EF-hand domain-containing family member A1**	EFHA1
0,023	1,82	3,54	**354,25**	O00562	**Membrane-associated phosphatidylinositol transfer protein 1**	PITPNM1
0,022	1,82	3,54	**353,71**	O95071	**E3 ubiquitin-protein ligase UBR5**	UBR5
0,031	1,82	3,53	**353,39**	P27707	**Deoxycytidine kinase**	DCK
0,014	1,80	3,48	**347,74**	Q9HC78;Q96K62	**Zinc finger and BTB domain-containing protein 20;Zinc finger and BTB domain-conta**	ZBTB20;ZBTB45
0,041	1,80	3,47	**347,18**	Q9BTD8	**RNA-binding protein 42**	RBM42
0,048	1,77	3,42	**341,81**	Q14498;Q86U06	**RNA-binding protein 39**	RBM39
0,020	1,77	3,40	**340,06**	P02649	**Apolipoprotein E**	APOE
0,028	1,76	3,39	**339,13**	O43716	**Glutamyl-tRNA(Gln) amidotransferase subunit C, mitochondrial**	GATC
0,044	1,76	3,38	**338,01**	Q96SU4	**Oxysterol-binding protein-related protein 9**	OSBPL9
0,041	1,75	3,35	**335,22**	Q96FJ2	**Dynein light chain 2, cytoplasmic**	DYNLL2
0,006	1,75	3,35	**335,21**	Q96HY6	**DDRGK domain-containing protein 1**	DDRGK1
0,040	1,73	3,31	**331,05**	O15042	**U2 snRNP-associated SURP motif-containing protein**	U2SURP
0,006	1,72	3,30	**329,81**	P49454	**Centromere protein F**	CENPF
0,016	1,71	3,26	**326,48**	Q9P265	**Disco-interacting protein 2 homolog B**	DIP2B
0,009	1,70	3,26	**325,53**	Q03701	**CCAAT/enhancer-binding protein zeta**	CEBPZ
0,026	1,67	3,19	**319,28**	Q6KC79	**Nipped-B-like protein**	NIPBL
0,021	1,64	3,12	**311,96**	P35250	**Replication factor C subunit 2**	RFC2
0,034	1,63	3,11	**310,56**	Q5VSL9;Q9ULQ0	**Protein FAM40A**	FAM40A

0,023	1,63	3,09	**308,81**	Q9Y282	**Endoplasmic reticulum-Golgi intermediate compartment protein 3**	ERGIC3
0,026	1,59	3,02	**301,88**	Q9H3P2	**Negative elongation factor A**	WHSC2
0,009	1,59	3,00	**300,38**	Q86UV5	**Ubiquitin carboxyl-terminal hydrolase 48**	USP48
0,050	1,59	3,00	**300,27**	P50851	**Lipopolysaccharide-responsive and beige-like anchor protein**	LRBA
0,045	1,58	2,99	**299,05**	Q9Y4F1	**FERM, RhoGEF and pleckstrin domain-containing protein 1**	FARP1
0,045	1,58	2,99	**298,74**	Q99729	**Heterogeneous nuclear ribonucleoprotein A/B**	HNRNPAB
0,044	1,57	2,98	**297,65**	Q76FK4	**Nucleolar protein 8**	NOL8
0,026	1,57	2,97	**296,75**	Q9P1Y5	**Calmodulin-regulated spectrin-associated protein 3**	CAMSAP3
0,039	1,54	2,91	**291,09**	Q9NPJ6	**Mediator of RNA polymerase II transcription subunit 4**	MED4
0,030	1,53	2,90	**289,68**	P16455	**Methylated-DNA--protein-cysteine methyltransferase**	MGMT
0,011	1,49	2,81	**281,28**	Q10713	**Mitochondrial-processing peptidase subunit alpha**	PMPCA
0,000	1,49	2,81	**280,88**	Q12872	**Splicing factor, suppressor of white-apricot homolog**	SFSWAP
0,030	1,48	2,78	**278,27**	Q8TD47	**40S ribosomal protein S4, Y isoform 2**	RPS4Y2
0,045	1,47	2,78	**277,77**	Q96DE0	**U8 snoRNA-decapping enzyme**	NUDT16
0,042	1,47	2,77	**276,95**	Q9UKE5	**TRAF2 and NCK-interacting protein kinase**	TNIK
0,018	1,47	2,77	**276,89**	Q9H299	**SH3 domain-binding glutamic acid-rich-like protein 3**	SH3BGRL3
0,023	1,47	2,76	**276,36**	P54274	**Telomeric repeat-binding factor 1**	TERF1
0,034	1,46	2,76	**275,76**	Q9NVC6	**Mediator of RNA polymerase II transcription subunit 17**	MED17
0,039	1,46	2,75	**275,42**	Q2TB90	**Putative hexokinase HKDC1**	HKDC1
0,000	1,46	2,75	**275,33**	O60563	**Cyclin-T1**	CCNT1
0,024	1,45	2,73	**272,90**	Q9UDT6	**CAP-Gly domain-containing linker protein 2**	CLIP2
0,013	1,44	2,72	**272,21**	O96019;O94805	**Actin-like protein 6A**	ACTL6A
0,018	1,43	2,70	**269,64**	Q7KZ85	**Transcription elongation factor SPT6**	SUPT6H
0,046	1,43	2,69	**268,54**	Q08379	**Golgin subfamily A member 2**	GOLGA2
0,001	1,38	2,59	**259,46**	Q9P2N5	**RNA-binding protein 27**	RBM27
0,015	1,37	2,58	**258,41**	Q92610	**Zinc finger protein 592**	ZNF592
0,047	1,37	2,58	**258,04**	Q13443	**Disintegrin and metalloproteinase domain-containing protein 9**	ADAM9
0,014	1,37	2,58	**257,92**	Q9NRZ9	**Lymphoid-specific helicase**	HELLS
0,019	1,37	2,58	**257,89**	Q9NQV6	**PR domain zinc finger protein 10**	PRDM10
0,000	1,36	2,57	**256,82**	P52294	**Importin subunit alpha-1**	KPNA1
0,002	1,35	2,56	**255,73**	Q96T58	**Msx2-interacting protein**	SPEN
0,000	1,34	2,53	**253,44**	O75934	**Pre-mRNA-splicing factor SPF27**	BCAS2
0,020	1,34	2,53	**253,08**	Q8WV41	**Sorting nexin-33**	SNX33

0,049	1,33	2,52	**252,20**	Q96FV9	**THO complex subunit 1**	THOC1
0,047	1,33	2,51	**251,27**	Q6ZSJ8	**Uncharacterized protein C1orf122**	C1orf122
0,027	1,33	2,51	**250,68**	Q02447	**Transcription factor Sp3**	SP3
0,020	1,30	2,46	**245,77**	Q9NQX3	**Gephyrin;Molybdopterin adenylyltransferase;Molybdopterin molybdenumtransfera**	GPHN
0,043	1,28	2,43	**242,77**	Q7L5D6	**Golgi to ER traffic protein 4 homolog**	GET4
0,009	1,26	2,40	**239,70**	Q15050	**Ribosome biogenesis regulatory protein homolog**	RRS1
0,042	1,26	2,39	**238,74**	Q70E73	**Ras-associated and pleckstrin homology domains-containing protein 1**	RAPH1
0,041	1,25	2,38	**238,03**	Q9H267	**Vacuolar protein sorting-associated protein 33B**	VPS33B
0,013	1,25	2,38	**238,01**	Q13888;Q6P1K8	**General transcription factor IIH subunit 2;General transcription factor IIH subunit 2-**	GTF2H2;GTF2H2C
0,024	1,25	2,38	**237,85**	Q8N3C7	**CAP-Gly domain-containing linker protein 4**	CLIP4
0,022	1,25	2,37	**237,27**	Q96B26	**Exosome complex component RRP43**	EXOSC8
0,028	1,25	2,37	**237,14**	P61962	**DDB1- and CUL4-associated factor 7**	DCAF7
0,002	1,24	2,37	**236,90**	Q9H008	**Phospholysine phosphohistidine inorganic pyrophosphate phosphatase**	LHPP
0,027	1,24	2,36	**235,87**	Q8WYH8;Q9UNL4	**Inhibitor of growth protein 5**	ING5
0,002	1,24	2,36	**235,56**	Q9UKD2	**mRNA turnover protein 4 homolog**	MRTO4
0,023	1,23	2,35	**235,37**	Q8WXF1	**Paraspeckle component 1**	PSPC1
0,007	1,23	2,35	**234,66**	Q9NUI1	**Peroxisomal 2,4-dienoyl-CoA reductase**	DECR2
0,015	1,22	2,32	**232,24**	Q96P48	**Arf-GAP with Rho-GAP domain, ANK repeat and PH domain-containing protein 1**	ARAP1
0,030	1,21	2,31	**231,35**	Q7L2J0	**7SK snRNA methylphosphate capping enzyme**	MEPCE
0,018	1,21	2,31	**230,94**	Q6PK04	**Coiled-coil domain-containing protein 137**	CCDC137
0,044	1,21	2,31	**230,80**	Q0JRZ9	**FCH domain only protein 2**	FCHO2
0,001	1,20	2,30	**230,06**	P09234	**U1 small nuclear ribonucleoprotein C**	SNRPC
0,000	1,19	2,28	**228,23**	Q8IUF8	**MYC-induced nuclear antigen**	MINA
0,025	1,19	2,27	**227,41**	Q96S94	**Cyclin-L2**	CCNL2
0,004	1,18	2,27	**227,35**	Q8N2M8	**CLK4-associating serine/arginine rich protein**	CLASRP
0,002	1,18	2,26	**225,90**	Q9Y3B4	**Pre-mRNA branch site protein p14**	SF3B14
0,038	1,17	2,25	**225,23**	Q8TF74	**WAS/WASL-interacting protein family member 2**	WIPF2
0,027	1,15	2,21	**221,46**	Q8WUA8	**Tsukushin**	TSKU
0,042	1,15	2,21	**221,15**	Q96EY4	**Translation machinery-associated protein 16**	TMA16
0,026	1,13	2,20	**219,55**	Q9NXV6	**CDKN2A-interacting protein**	CDKN2AIP
0,011	1,13	2,19	**219,34**	P62829	**60S ribosomal protein L23**	RPL23
0,044	1,13	2,18	**218,31**	Q92769	**Histone deacetylase 2**	HDAC2
0,021	1,12	2,17	**216,61**	Q92560	**Ubiquitin carboxyl-terminal hydrolase BAP1**	BAP1

0,029	1,10	2,14	**214,35**	Q14676	**Mediator of DNA damage checkpoint protein 1**	MDC1
0,004	1,09	2,13	**213,29**	P32322	**Pyrroline-5-carboxylate reductase 1, mitochondrial**	PYCR1
0,031	1,09	2,13	**213,22**	P17676	**CCAAT/enhancer-binding protein beta**	CEBPB
0,004	1,09	2,13	**212,68**	Q76L83	**Putative Polycomb group protein ASXL2**	ASXL2
0,005	1,07	2,10	**210,37**	P26599	**Polypyrimidine tract-binding protein 1**	PTBP1
0,040	1,07	2,10	**209,64**	Q00013	**55 kDa erythrocyte membrane protein**	MPP1
0,033	1,06	2,08	**208,50**	Q9P253	**Vacuolar protein sorting-associated protein 18 homolog**	VPS18
0,000	1,05	2,07	**207,03**	Q8IXM2	**Chromatin complexes subunit BAP18**	BAP18
0,012	1,04	2,05	**205,42**	P52272	**Heterogeneous nuclear ribonucleoprotein M**	HNRNPM
0,010	1,03	2,04	**204,33**	P60953	**Cell division control protein 42 homolog**	CDC42
0,000	1,01	2,01	**201,20**	P39060	**Collagen alpha-1(XVIII) chain;Endostatin**	COL18A1
0,042	0,99	1,98	**198,34**	O43432	**Eukaryotic translation initiation factor 4 gamma 3**	EIF4G3
0,044	0,98	1,98	**197,82**	Q9BVL2	**Nucleoporin p58/p45**	NUPL1
0,002	0,98	1,98	**197,52**	Q9NVI7;Q5T2N8	**ATPase family AAA domain-containing protein 3A**	ATAD3A
0,020	0,98	1,97	**196,74**	Q9H4I3	**TraB domain-containing protein**	TRABD
0,015	0,97	1,96	**196,08**	Q08211	**ATP-dependent RNA helicase A**	DHX9
0,031	0,97	1,96	**195,99**	Q14451	**Growth factor receptor-bound protein 7**	GRB7
0,032	0,96	1,95	**195,11**	Q9NRW7	**Vacuolar protein sorting-associated protein 45**	VPS45
0,021	0,96	1,95	**194,90**	Q99583	**Max-binding protein MNT**	MNT
0,026	0,95	1,94	**193,51**	Q14644	**Ras GTPase-activating protein 3**	RASA3
0,002	0,95	1,93	**193,41**	Q02880	**DNA topoisomerase 2-beta**	TOP2B
0,000	0,95	1,93	**193,28**	Q9UFF9	**CCR4-NOT transcription complex subunit 8**	CNOT8
0,013	0,94	1,92	**192,27**	O60232	**Sjoegren syndrome/scleroderma autoantigen 1**	SSSCA1
0,007	0,93	1,91	**191,03**	P14902	**Indoleamine 2,3-dioxygenase 1**	IDO1
0,000	0,93	1,91	**190,68**	Q9Y5A9	**YTH domain family protein 2**	YTHDF2
0,033	0,91	1,88	**188,41**	Q9Y3A2	**Probable U3 small nucleolar RNA-associated protein 11**	UTP11L
0,035	0,90	1,87	**186,56**	P51116	**Fragile X mental retardation syndrome-related protein 2**	FXR2
0,038	0,90	1,86	**186,49**	P55795	**Heterogeneous nuclear ribonucleoprotein H2**	HNRNPH2
0,028	0,89	1,85	**185,29**	O14929	**Histone acetyltransferase type B catalytic subunit**	HAT1
0,002	0,89	1,85	**185,09**	Q96EZ8	**Microspherule protein 1**	MCRS1
0,026	0,89	1,85	**184,95**	Q9HCJ3	**Ribonucleoprotein PTB-binding 2**	RAVER2
0,028	0,88	1,84	**184,48**	Q9NPR2	**Semaphorin-4B**	SEMA4B
0,035	0,88	1,84	**184,45**	Q13439	**Golgin subfamily A member 4**	GOLGA4

0,000	0,88	1,83	**183,46**	Q16186	**Proteasomal ubiquitin receptor ADRM1**	ADRM1
0,032	0,88	1,83	**183,44**	Q96FK6	**WD repeat-containing protein 89**	WDR89
0,018	0,86	1,81	**180,88**	Q9NP64	**Nucleolar protein of 40 kDa**	ZCCHC17
0,006	0,85	1,81	**180,85**	Q8WXA9	**Splicing regulatory glutamine/lysine-rich protein 1**	SREK1
0,040	0,85	1,81	**180,84**	Q9P0L0	**Vesicle-associated membrane protein-associated protein A**	VAPA
0,004	0,85	1,80	**180,19**	Q9UHD9	**Ubiquilin-2**	UBQLN2
0,044	0,84	1,79	**179,46**	Q9Y3C4	**TP53RK-binding protein**	TPRKB
0,049	0,83	1,78	**178,09**	Q9ULU4	**Protein kinase C-binding protein 1**	ZMYND8
0,010	0,82	1,77	**177,11**	P33991;Q9UJA3	**DNA replication licensing factor MCM4**	MCM4
0,003	0,82	1,76	**176,47**	P51553	**Isocitrate dehydrogenase [NAD] subunit gamma, mitochondrial**	IDH3G
0,048	0,81	1,75	**175,41**	Q13043	**Serine/threonine-protein kinase 4;Serine/threonine-protein kinase 4 37kDa subunit**	STK4
0,045	0,81	1,75	**175,30**	Q9NUD5	**Zinc finger CCHC domain-containing protein 3**	ZCCHC3
0,015	0,80	1,74	**173,71**	Q8IXK0	**Polyhomeotic-like protein 2**	PHC2
0,045	0,79	1,73	**173,31**	O75175	**CCR4-NOT transcription complex subunit 3**	CNOT3
0,042	0,78	1,72	**171,86**	Q9UPN4	**5-azacytidine-induced protein 1**	AZI1
0,021	0,78	1,72	**171,62**	A9UHW6	**MIF4G domain-containing protein**	MIF4GD
0,040	0,77	1,71	**170,79**	Q9BWF3	**RNA-binding protein 4**	RBM4
0,015	0,77	1,70	**170,46**	Q13595	**Transformer-2 protein homolog alpha**	TRA2A
0,009	0,77	1,70	**170,27**	Q9NVP1	**ATP-dependent RNA helicase DDX18**	DDX18
0,028	0,76	1,69	**169,11**	Q6ZNW5	**GDP-D-glucose phosphorylase C15orf58**	C15orf58
0,043	0,75	1,68	**167,79**	Q00610;P53675	**Clathrin heavy chain 1**	CLTC
0,037	0,74	1,67	**167,47**	Q12906	**Interleukin enhancer-binding factor 3**	ILF3
0,042	0,74	1,67	**166,89**	Q5JTW2	**Centrosomal protein of 78 kDa**	CEP78
0,041	0,74	1,66	**166,44**	P61964	**WD repeat-containing protein 5**	WDR5
0,021	0,73	1,66	**166,30**	Q02750	**Dual specificity mitogen-activated protein kinase kinase 1**	MAP2K1
0,027	0,73	1,66	**165,81**	Q53H96	**Pyrroline-5-carboxylate reductase 3**	PYCRL
0,033	0,72	1,65	**165,00**	Q9BTC8	**Metastasis-associated protein MTA3**	MTA3
0,032	0,72	1,64	**164,36**	O15056	**Synaptojanin-2**	SYNJ2
0,034	0,71	1,64	**163,92**	Q969V3	**Nicalin**	NCLN
0,020	0,71	1,64	**163,57**	Q13123	**Protein Red**	IK
0,016	0,70	1,63	**162,61**	P27540	**Aryl hydrocarbon receptor nuclear translocator**	ARNT
0,023	0,68	1,60	**160,17**	Q4LE39	**AT-rich interactive domain-containing protein 4B**	ARID4B
0,046	0,68	1,60	**160,12**	Q9BZV1	**UBX domain-containing protein 6**	UBXN6

0,011	0,68	1,60	**160,10**	P07737;CON__P02584	**Profilin-1**	PFN1
0,028	0,67	1,59	**159,47**	Q32P44	**Echinoderm microtubule-associated protein-like 3**	EML3
0,040	0,67	1,59	**159,34**	Q15652	**Probable JmjC domain-containing histone demethylation protein 2C**	JMJD1C
0,040	0,66	1,58	**157,62**	P83436	**Conserved oligomeric Golgi complex subunit 7**	COG7
0,007	0,66	1,58	**157,54**	Q8N3X1	**Formin-binding protein 4**	
0,005	0,65	1,57	**156,80**	P52789;P52790	**Hexokinase-2**	HK2
0,040	0,65	1,56	**156,48**	Q15418	**Ribosomal protein S6 kinase alpha-1**	RPS6KA1
0,045	0,63	1,55	**155,00**	Q8WWQ0	**PH-interacting protein**	PHIP
0,012	0,62	1,54	**153,76**	Q08945	**FACT complex subunit SSRP1**	SSRP1
0,048	0,62	1,53	**153,38**	Q9HCG8	**Pre-mRNA-splicing factor CWC22 homolog**	CWC22
0,035	0,61	1,53	**152,79**	Q9ULV4;Q6QEF8	**Coronin-1C**	CORO1C
0,000	0,61	1,53	**152,55**	P53396	**ATP-citrate synthase**	ACLY
0,032	0,61	1,52	**152,23**	Q13155	**Aminoacyl tRNA synthase complex-interacting multifunctional protein 2**	AIMP2
0,035	0,60	1,52	**151,70**	P19338	**Nucleolin**	NCL
0,037	0,60	1,52	**151,70**	P51948	**CDK-activating kinase assembly factor MAT1**	MNAT1
0,008	0,59	1,50	**150,16**	Q92499	**ATP-dependent RNA helicase DDX1**	DDX1
0,019	0,58	1,50	**150,00**	Q9Y388	**RNA-binding motif protein, X-linked 2**	RBMX2
0,013	0,58	1,49	**149,23**	Q96DH6	**RNA-binding protein Musashi homolog 2**	MSI2
0,000	0,56	1,48	**147,74**	O75083	**WD repeat-containing protein 1**	WDR1
0,026	0,55	1,46	**146,26**	P00338;Q6ZMR3;P0786	**L-lactate dehydrogenase A chain**	LDHA
0,024	0,53	1,44	**144,04**	Q9BPX5	**Actin-related protein 2/3 complex subunit 5-like protein**	ARPC5L
0,028	0,52	1,43	**143,14**	Q9P0V9	**Septin-10**	Sep.10
0,030	0,52	1,43	**142,99**	Q9UHB9	**Signal recognition particle 68 kDa protein**	SRP68
0,009	0,51	1,43	**142,65**	P06744	**Glucose-6-phosphate isomerase**	GPI
0,045	0,51	1,42	**142,24**	032;P68133;P62736;P63	**Actin, alpha cardiac muscle 1;Actin, alpha skeletal muscle;Actin, aortic smooth musc**	ACTC1;ACTA1;ACT
0,007	0,50	1,41	**141,06**	O00571;O15523;Q9NQI	**ATP-dependent RNA helicase DDX3X;ATP-dependent RNA helicase DDX3Y**	DDX3X;DDX3Y
0,039	0,49	1,40	**140,42**	Q13330	**Metastasis-associated protein MTA1**	MTA1
0,009	0,47	1,39	**138,68**	P52888	**Thimet oligopeptidase**	THOP1
0,017	0,46	1,37	**137,33**	P46087	**Putative ribosomal RNA methyltransferase NOP2**	NOP2
0,023	0,46	1,37	**137,23**	P62136	**Serine/threonine-protein phosphatase PP1-alpha catalytic subunit**	PPP1CA
0,034	0,45	1,37	**137,03**	Q14974	**Importin subunit beta-1**	KPNB1
0,045	0,45	1,36	**136,24**	Q96CW5	**Gamma-tubulin complex component 3**	TUBGCP3
0,026	0,44	1,36	**136,08**	Q09666	**Neuroblast differentiation-associated protein AHNAK**	AHNAK

0,032	0,41	1,32	**132,44**	P50454	**Serpin H1**	SERPINH1
0,027	0,40	1,32	**131,88**	Q15459	**Splicing factor 3A subunit 1**	SF3A1
0,024	0,39	1,31	**131,38**	Q9NW13	**RNA-binding protein 28**	RBM28
0,037	0,38	1,30	**130,15**	O14979	**Heterogeneous nuclear ribonucleoprotein D-like**	HNRPDL
0,043	0,38	1,30	**130,02**	Q12792	**Twinfilin-1**	TWF1
0,012	0,31	1,24	**124,28**	P52788	**Spermine synthase**	SMS
0,003	0,31	1,24	**123,79**	Q9Y224	**UPF0568 protein C14orf166**	C14orf166
0,001	0,30	1,24	**123,51**	P62140	**Serine/threonine-protein phosphatase PP1-beta catalytic subunit**	PPP1CB
0,020	0,30	1,23	**123,31**	P35249	**Replication factor C subunit 4**	RFC4
0,006	0,28	1,22	**121,74**	Q6NZI2	**Polymerase I and transcript release factor**	PTRF
0,038	0,26	1,20	**119,58**	Q9C0J8	**pre-mRNA 3 end processing protein WDR33**	WDR33
0,013	0,25	1,19	**119,08**	Q14103	**Heterogeneous nuclear ribonucleoprotein D0**	HNRNPD
**24 h Up**						
0,00	5,00	31,99	**3199,09**	Q3ZCM7	**Tubulin beta-8 chain**	TUBB8
0,02	3,96	15,51	**1551,23**	Q9H930	**Nuclear body protein SP140-like protein**	SP140L
0,02	3,92	15,10	**1509,72**	Q8TC07	**TBC1 domain family member 15**	TBC1D15
0,00	3,57	11,89	**1189,13**	P06748	**Nucleophosmin**	NPM1
0,00	3,32	10,01	**1000,55**	Q16678	**Cytochrome P450 1B1**	CYP1B1
0,02	3,25	9,52	**952,43**	P13929	**Beta-enolase**	ENO3
0,01	3,03	8,19	**818,82**	Q9UBB6	**Neurochondrin**	NCDN
0,00	2,89	7,42	**741,89**	Q8NI77	**Kinesin-like protein KIF18A**	KIF18A
0,00	2,88	7,37	**736,58**	Q13547	**Histone deacetylase 1**	HDAC1
0,03	2,86	7,24	**723,96**	P08621	**U1 small nuclear ribonucleoprotein 70 kDa**	SNRNP70
0,00	2,79	6,94	**694,01**	Q6NVY1	**3-hydroxyisobutyryl-CoA hydrolase, mitochondrial**	HIBCH
0,00	2,78	6,88	**687,59**	Q15121	**Astrocytic phosphoprotein PEA-15**	PEA15
0,00	2,76	6,78	**678,22**	P05386	**60S acidic ribosomal protein P1**	RPLP1
0,00	2,75	6,72	**672,02**	Q9NZT1	**Calmodulin-like protein 5**	CALML5
0,00	2,73	6,62	**661,93**	33778;Q16778;Q8N257;	**Histone H2B type 1-J;Histone H2B type 1-O;Histone H2B type 1-B;Histone H2B type**	HIST1H2BJ;HIST1H
0,04	2,71	6,56	**656,32**	Q6P996;Q6P474	**Pyridoxal-dependent decarboxylase domain-containing protein 1**	PDXDC1
0,00	2,69	6,44	**643,66**	Q16512	**Serine/threonine-protein kinase N1**	PKN1
0,00	2,62	6,16	**616,43**	O14791	**Apolipoprotein L1**	APOL1
0,00	2,61	6,11	**611,44**	O76054	**SEC14-like protein 2**	SEC14L2
0,00	2,60	6,07	**606,72**	Q08554	**Desmocollin-1**	DSC1

0,01	2,55	5,87	**586,99**	O14874	**[3-methyl-2-oxobutanoate dehydrogenase [lipoamide]] kinase, mitochondrial**	BCKDK
0,00	2,53	5,79	**578,56**	P43490	**Nicotinamide phosphoribosyltransferase**	NAMPT
0,00	2,50	5,68	**567,50**	P37108	**Signal recognition particle 14 kDa protein**	SRP14
0,00	2,49	5,62	**561,62**	P40306	**Proteasome subunit beta type-10**	PSMB10
0,02	2,47	5,55	**554,86**	Q6WKZ4;Q7L804	**Rab11 family-interacting protein 1**	RAB11FIP1
0,01	2,42	5,36	**536,37**	P17480	**Nucleolar transcription factor 1**	UBTF
0,03	2,42	5,36	**536,00**	P15408	**Fos-related antigen 2**	FOSL2
0,03	2,39	5,26	**525,87**	Q8IWI9	**MAX gene-associated protein**	MGA
0,00	2,39	5,23	**522,90**	Q9Y6X8	**Zinc fingers and homeoboxes protein 2**	ZHX2
0,00	2,37	5,16	**515,58**	Q7LFL8	**CXXC-type zinc finger protein 5**	CXXC5
0,03	2,33	5,04	**504,43**	P06702	**Protein S100-A9**	S100A9
0,01	2,33	5,02	**501,60**	Q9P2R7	**Succinyl-CoA ligase [ADP-forming] subunit beta, mitochondrial**	SUCLA2
0,01	2,32	5,01	**500,69**	Q9NX46	**Poly(ADP-ribose) glycohydrolase ARH3**	ADPRHL2
0,02	2,32	5,00	**500,40**	Q13151	**Heterogeneous nuclear ribonucleoprotein A0**	HNRNPA0
0,03	2,32	4,98	**497,76**	Q9HAB8	**Phosphopantothenate--cysteine ligase**	PPCS
0,01	2,28	4,85	**485,01**	P47736	**Rap1 GTPase-activating protein 1**	RAP1GAP
0,04	2,28	4,85	**484,93**	P28290	**Sperm-specific antigen 2**	SSFA2
0,05	2,26	4,78	**478,04**	Q9UHY1	**Nuclear receptor-binding protein**	NRBP1
0,01	2,21	4,63	**463,39**	P84103	**Serine/arginine-rich splicing factor 3**	SRSF3
0,01	2,16	4,48	**448,44**	Q9Y5V0	**Zinc finger protein 706**	ZNF706
0,00	2,16	4,46	**445,84**	P22102	**Trifunctional purine biosynthetic protein adenosine-3;Phosphoribosylamine--glycine**	GART
0,01	2,15	4,45	**444,96**	P46782	**40S ribosomal protein S5;40S ribosomal protein S5, N-terminally processed**	RPS5
0,03	2,14	4,42	**442,26**	P48200	**Iron-responsive element-binding protein 2**	IREB2
0,05	2,12	4,36	**435,75**	Q4V328	**GRIP1-associated protein 1**	GRIPAP1
0,03	2,12	4,35	**434,67**	Q9Y4C8	**Probable RNA-binding protein 19**	RBM19
0,04	2,08	4,24	**423,87**	P23246	**Splicing factor, proline- and glutamine-rich**	SFPQ
0,01	2,08	4,23	**423,20**	P62993	**Growth factor receptor-bound protein 2**	GRB2
0,00	2,06	4,17	**417,01**	Q99715	**Collagen alpha-1(XII) chain**	COL12A1
0,03	2,05	4,15	**415,06**	Q9H0A0	**N-acetyltransferase 10**	NAT10
0,01	2,02	4,06	**405,55**	Q9H2H8	**Peptidyl-prolyl cis-trans isomerase-like 3**	PPIL3
0,01	2,00	4,00	**400,45**	Q6P4E1	**Protein CASC4**	CASC4
0,02	1,99	3,98	**398,20**	P35226;P35227	**Polycomb complex protein BMI-1**	BMI1
0,00	1,98	3,94	**393,99**	Q9C0J8	**pre-mRNA 3 end processing protein WDR33**	WDR33

0,00	1,95	3,87	**386,85**	P12273	**Prolactin-inducible protein**	PIP
0,03	1,95	3,86	**385,63**	P29218	**Inositol monophosphatase 1**	IMPA1
0,01	1,92	3,77	**377,35**	Q9P013	**Protein CWC15 homolog**	CWC15
0,03	1,91	3,77	**376,92**	Q15287	**RNA-binding protein with serine-rich domain 1**	RNPS1
0,00	1,88	3,67	**367,17**	P39060	**Collagen alpha-1(XVIII) chain;Endostatin**	COL18A1
0,03	1,84	3,57	**357,02**	Q9NSE4	**Isoleucine--tRNA ligase, mitochondrial**	IARS2
0,01	1,83	3,55	**355,15**	Q9H008	**Phospholysine phosphohistidine inorganic pyrophosphate phosphatase**	LHPP
0,03	1,83	3,55	**354,94**	Q9NX62	**Inositol monophosphatase 3**	IMPAD1
0,00	1,80	3,48	**347,76**	Q9H4I2	**Zinc fingers and homeoboxes protein 3**	ZHX3
0,02	1,80	3,47	**347,07**	Q16629	**Serine/arginine-rich splicing factor 7**	SRSF7
0,04	1,78	3,44	**343,67**	P22061	**Protein-L-isoaspartate(D-aspartate) O-methyltransferase**	PCMT1
0,03	1,78	3,43	**342,54**	O15460	**Prolyl 4-hydroxylase subunit alpha-2**	P4HA2
0,01	1,75	3,36	**335,86**	Q9H2U1	**Probable ATP-dependent RNA helicase DHX36**	DHX36
0,02	1,75	3,36	**335,65**	Q5ZPR3	**CD276 antigen**	CD276
0,01	1,74	3,35	**334,55**	Q14512	**Fibroblast growth factor-binding protein 1**	FGFBP1
0,03	1,74	3,34	**334,45**	P16220;P18846	**Cyclic AMP-responsive element-binding protein 1**	CREB1
0,03	1,73	3,32	**332,44**	P27540	**Aryl hydrocarbon receptor nuclear translocator**	ARNT
0,00	1,73	3,32	**332,13**	Q9UPN9	**E3 ubiquitin-protein ligase TRIM33**	TRIM33
0,02	1,73	3,32	**332,05**	Q9Y2R4	**Probable ATP-dependent RNA helicase DDX52**	DDX52
0,00	1,72	3,29	**329,12**	Q9UKY1	**Zinc fingers and homeoboxes protein 1**	ZHX1
0,03	1,71	3,28	**327,53**	P61225	**Ras-related protein Rap-2b**	RAP2B
0,02	1,71	3,27	**327,40**	Q9H7L9	**Sin3 histone deacetylase corepressor complex component SDS3**	SUDS3
0,03	1,71	3,27	**326,68**	Q8NFH5	**Nucleoporin NUP53**	NUP35
0,00	1,71	3,26	**326,07**	P30042	**ES1 protein homolog, mitochondrial**	C21orf33
0,01	1,69	3,23	**322,75**	Q15257	**Serine/threonine-protein phosphatase 2A activator**	PPP2R4
0,04	1,68	3,21	**321,38**	P80217	**Interferon-induced 35 kDa protein**	IFI35
0,01	1,68	3,19	**319,38**	Q9BY42	**UPF0549 protein C20orf43**	C20orf43
0,01	1,67	3,18	**318,43**	Q5JTZ9	**Alanine--tRNA ligase, mitochondrial**	AARS2
0,01	1,67	3,18	**318,24**	O00186	**Syntaxin-binding protein 3**	STXBP3
0,01	1,67	3,18	**317,98**	O00625	**Pirin**	PIR
0,00	1,66	3,16	**315,87**	Q8IVL5	**Prolyl 3-hydroxylase 2**	LEPREL1
0,01	1,66	3,15	**315,01**	Q9ULM3	**YEATS domain-containing protein 2**	YEATS2
0,01	1,66	3,15	**314,97**	P16333	**Cytoplasmic protein NCK1**	NCK1

0,03	1,65	3,15	**314,72**	O15145	**Actin-related protein 2/3 complex subunit 3**	ARPC3
0,00	1,65	3,14	**314,50**	P02751	**Fibronectin;Anastellin;Ugl-Y1;Ugl-Y2;Ugl-Y3**	FN1
0,02	1,65	3,14	**313,86**	O00767	**Acyl-CoA desaturase**	SCD
0,02	1,63	3,09	**308,86**	O43291	**Kunitz-type protease inhibitor 2**	SPINT2
0,03	1,62	3,07	**306,85**	P51948	**CDK-activating kinase assembly factor MAT1**	MNAT1
0,01	1,62	3,07	**306,68**	Q02413	**Desmoglein-1**	DSG1
0,01	1,60	3,03	**303,23**	Q9ULG6	**Cell cycle progression protein 1**	CCPG1
0,02	1,59	3,02	**301,99**	P57081	**tRNA (guanine-N(7)-)-methyltransferase subunit WDR4**	WDR4
0,04	1,59	3,02	**301,88**	Q9NZI7	**Upstream-binding protein 1**	UBP1
0,02	1,59	3,01	**301,06**	O75164	**Lysine-specific demethylase 4A**	KDM4A
0,04	1,59	3,01	**300,86**	P53634	**Dipeptidyl peptidase 1;Dipeptidyl peptidase 1 exclusion domain chain;Dipeptidyl pe**	CTSC
0,02	1,59	3,00	**300,31**	O75683	**Surfeit locus protein 6**	SURF6
0,00	1,59	3,00	**300,17**	P68366;Q9NY65	**Tubulin alpha-4A chain**	TUBA4A
0,01	1,58	2,99	**298,63**	P00918	**Carbonic anhydrase 2**	CA2
0,03	1,56	2,95	**294,60**	Q8WWI1	**LIM domain only protein 7**	LMO7
0,04	1,54	2,90	**289,85**	P31949	**Protein S100-A11**	S100A11
0,01	1,53	2,90	**289,63**	P52926	**High mobility group protein HMGI-C**	HMGA2
0,01	1,52	2,87	**286,59**	Q01081;Q8WU68	**Splicing factor U2AF 35 kDa subunit;Splicing factor U2AF 26 kDa subunit**	U2AF1;U2AF1L4
0,01	1,52	2,86	**286,16**	Q9NVZ3;Q8NC96	**Adaptin ear-binding coat-associated protein 2**	NECAP2
0,04	1,52	2,86	**285,97**	Q9Y226	**Solute carrier family 22 member 13**	SLC22A13
0,00	1,51	2,84	**284,39**	P30838;P43353;P48448	**Aldehyde dehydrogenase, dimeric NADP-preferring**	ALDH3A1
0,00	1,51	2,84	**284,16**	Q13795	**ADP-ribosylation factor-related protein 1**	ARFRP1
0,00	1,50	2,83	**282,56**	Q12962	**Transcription initiation factor TFIID subunit 10**	TAF10
0,03	1,49	2,81	**280,81**	P48506	**Glutamate--cysteine ligase catalytic subunit**	GCLC
0,00	1,47	2,77	**276,89**	P43243	**Matrin-3**	MATR3
0,03	1,47	2,76	**276,19**	Q7Z4V5	**Hepatoma-derived growth factor-related protein 2**	HDGFRP2
0,00	1,46	2,76	**275,58**	Q92766	**Ras-responsive element-binding protein 1**	RREB1
0,02	1,45	2,72	**272,32**	P35240	**Merlin**	NF2
0,02	1,44	2,72	**272,06**	Q9UKJ3	**G patch domain-containing protein 8**	GPATCH8
0,00	1,43	2,70	**269,92**	Q96PU8	**Protein quaking**	QKI
0,00	1,43	2,69	**269,35**	Q16254	**Transcription factor E2F4**	E2F4
0,04	1,42	2,67	**266,81**	P61163	**Alpha-centractin**	ACTR1A
0,00	1,42	2,67	**266,74**	P49903	**Selenide, water dikinase 1**	SEPHS1

0,01	1,41	2,66	**266,07**	Q5QJE6	**Deoxynucleotidyltransferase terminal-interacting protein 2**	DNTTIP2
0,04	1,41	2,66	**265,75**	P19447	**TFIIH basal transcription factor complex helicase XPB subunit**	ERCC3
0,01	1,41	2,66	**265,71**	Q9H8M2	**Bromodomain-containing protein 9**	BRD9
0,00	1,41	2,66	**265,60**	P09917	**Arachidonate 5-lipoxygenase**	ALOX5
0,03	1,41	2,66	**265,56**	Q96F63	**Coiled-coil domain-containing protein 97**	CCDC97
0,00	1,40	2,65	**264,78**	Q5W0V3;Q86V87	**Protein FAM160B1**	FAM160B1
0,04	1,40	2,65	**264,70**	P24468;P10589	**COUP transcription factor 2**	NR2F2
0,02	1,40	2,64	**263,82**	O75607	**Nucleoplasmin-3**	NPM3
0,03	1,38	2,60	**259,56**	Q9NX58	**Cell growth-regulating nucleolar protein**	LYAR
0,04	1,37	2,59	**258,57**	Q8N3F8	**MICAL-like protein 1**	MICALL1
0,01	1,36	2,57	**257,16**	Q9H6R0	**Putative ATP-dependent RNA helicase DHX33**	DHX33
0,00	1,36	2,57	**256,74**	Q8IYU8	**EF-hand domain-containing family member A1**	EFHA1
0,00	1,35	2,56	**255,77**	P53801	**Pituitary tumor-transforming gene 1 protein-interacting protein**	PTTG1IP
0,02	1,35	2,55	**255,14**	O00763	**Acetyl-CoA carboxylase 2;Biotin carboxylase**	ACACB
0,02	1,35	2,55	**255,01**	Q9Y3E5	**Peptidyl-tRNA hydrolase 2, mitochondrial**	PTRH2
0,00	1,34	2,54	**253,92**	P54727	**UV excision repair protein RAD23 homolog B**	RAD23B
0,05	1,34	2,53	**253,07**	Q13740	**CD166 antigen**	ALCAM
0,01	1,34	2,52	**252,50**	Q8WUB8	**PHD finger protein 10**	PHF10
0,02	1,33	2,52	**252,06**	P17936	**Insulin-like growth factor-binding protein 3**	IGFBP3
0,00	1,33	2,51	**251,39**	Q7L4I2	**Arginine/serine-rich coiled-coil protein 2**	RSRC2
0,00	1,33	2,51	**250,99**	Q9BQ61	**Uncharacterized protein C19orf43**	C19orf43
0,00	1,33	2,51	**250,83**	Q96LT9	**RNA-binding protein 40**	RNPC3
0,00	1,33	2,51	**250,75**	Q6P587	**Acylpyruvase FAHD1, mitochondrial**	FAHD1
0,02	1,31	2,47	**247,49**	Q92878	**DNA repair protein RAD50**	RAD50
0,02	1,31	2,47	**247,35**	Q5JRA6	**Melanoma inhibitory activity protein 3**	MIA3
0,04	1,30	2,47	**246,96**	P35659	**Protein DEK**	DEK
0,05	1,29	2,45	**245,28**	Q6PJ69	**Tripartite motif-containing protein 65**	TRIM65
0,03	1,29	2,44	**244,17**	P38398	**Breast cancer type 1 susceptibility protein**	BRCA1
0,03	1,28	2,43	**242,99**	Q86V81	**THO complex subunit 4**	ALYREF
0,03	1,27	2,42	**241,93**	Q8IZ69	**tRNA (uracil-5-)-methyltransferase homolog A**	TRMT2A
0,02	1,27	2,41	**241,26**	P50579	**Methionine aminopeptidase 2**	METAP2
0,03	1,26	2,40	**239,62**	Q8NEJ9	**Neuroguidin**	NGDN
0,02	1,25	2,38	**238,09**	Q96RL1	**BRCA1-A complex subunit RAP80**	UIMC1

0,01	1,24	2,37	**236,93**	Q3YEC7	**Rab-like protein 1**	PARF
0,00	1,24	2,36	**236,48**	P09972	**Fructose-bisphosphate aldolase C**	ALDOC
0,00	1,24	2,36	**236,25**	Q2TAK8	**PWWP domain-containing protein MUM1**	MUM1
0,00	1,24	2,36	**235,95**	Q9NYH9	**U3 small nucleolar RNA-associated protein 6 homolog**	UTP6
0,01	1,24	2,36	**235,62**	Q8IYB3	**Serine/arginine repetitive matrix protein 1**	SRRM1
0,03	1,22	2,33	**233,48**	Q9H0A8	**COMM domain-containing protein 4**	COMMD4
0,00	1,22	2,33	**233,46**	P07195	**L-lactate dehydrogenase B chain**	LDHB
0,00	1,21	2,31	**231,43**	Q8IYH5	**ZZ-type zinc finger-containing protein 3**	ZZZ3
0,00	1,21	2,31	**231,42**	Q04828;P52895	**Aldo-keto reductase family 1 member C1;Aldo-keto reductase family 1 member C2**	AKR1C1;AKR1C2
0,01	1,21	2,31	**231,11**	Q8N2M8	**CLK4-associating serine/arginine rich protein**	CLASRP
0,00	1,21	2,31	**230,89**	Q14686	**Nuclear receptor coactivator 6**	NCOA6
0,00	1,21	2,31	**230,76**	O60701	**UDP-glucose 6-dehydrogenase**	UGDH
0,00	1,20	2,30	**230,07**	Q96EZ8	**Microspherule protein 1**	MCRS1
0,01	1,20	2,30	**229,78**	P11277	**Spectrin beta chain, erythrocyte**	SPTB
0,04	1,19	2,29	**228,69**	P16435	**NADPH--cytochrome P450 reductase**	POR
0,03	1,19	2,28	**228,22**	Q13428	**Treacle protein**	TCOF1
0,00	1,18	2,27	**226,76**	P80188	**Neutrophil gelatinase-associated lipocalin**	LCN2
0,00	1,18	2,26	**226,18**	P53355	**Death-associated protein kinase 1**	DAPK1
0,03	1,17	2,25	**225,24**	P27361	**Mitogen-activated protein kinase 3**	MAPK3
0,02	1,17	2,25	**224,60**	Q8WUA8	**Tsukushin**	TSKU
0,00	1,16	2,23	**223,35**	O15031	**Plexin-B2**	PLXNB2
0,00	1,16	2,23	**223,15**	Q6I9Y2	**THO complex subunit 7 homolog**	THOC7
0,01	1,15	2,21	**221,46**	Q96BD5	**PHD finger protein 21A**	PHF21A
0,01	1,14	2,21	**220,63**	O95503	**Chromobox protein homolog 6**	CBX6
0,00	1,14	2,20	**220,48**	O00468	**Agrin**	AGRN
0,00	1,12	2,18	**217,72**	Q15427	**Splicing factor 3B subunit 4**	SF3B4
0,01	1,12	2,18	**217,70**	Q96S94	**Cyclin-L2**	CCNL2
0,02	1,11	2,16	**216,43**	O95232	**Luc7-like protein 3**	LUC7L3
0,02	1,11	2,16	**215,88**	Q53GQ0	**Estradiol 17-beta-dehydrogenase 12**	HSD17B12
0,01	1,11	2,16	**215,80**	P49720	**Proteasome subunit beta type-3**	PSMB3
0,00	1,10	2,15	**214,58**	)7;Q5QNW6;Q93079;Q9	**Histone H2B type 1-K;Histone H2B type F-S;Histone H2B type 1-D;Histone H2B type**	HIST1H2BK;H2BFS;
0,03	1,10	2,14	**214,03**	Q5VZF2	**Muscleblind-like protein 2**	MBNL2
0,00	1,10	2,14	**213,90**	Q96D46	**60S ribosomal export protein NMD3**	NMD3

0,00	1,10	2,14	**213,79**	Q9NX70	**Mediator of RNA polymerase II transcription subunit 29**	MED29
0,02	1,09	2,14	**213,56**	Q9BTD8	**RNA-binding protein 42**	RBM42
0,03	1,08	2,12	**211,61**	O14925;Q5SRD1	**Mitochondrial import inner membrane translocase subunit Tim23;Putative mitocho**	TIMM23;TIMM23B
0,02	1,08	2,12	**211,57**	P10451;CON__P31096	**Osteopontin**	SPP1
0,00	1,07	2,11	**210,65**	Q9NR56	**Muscleblind-like protein 1**	MBNL1
0,00	1,07	2,10	**210,36**	Q9H981	**Actin-related protein 8**	ACTR8
0,00	1,07	2,10	**210,00**	Q9H0G5	**Nuclear speckle splicing regulatory protein 1**	NSRP1
0,01	1,06	2,09	**208,93**	Q06587	**E3 ubiquitin-protein ligase RING1**	RING1
0,00	1,06	2,08	**207,97**	Q6P4R8	**Nuclear factor related to kappa-B-binding protein**	NFRKB
0,00	1,06	2,08	**207,97**	P06454	**Prothymosin alpha;Thymosin alpha-1**	PTMA
0,00	1,05	2,08	**207,73**	P51825	**AF4/FMR2 family member 1**	AFF1
0,02	1,05	2,06	**206,40**	Q13889	**General transcription factor IIH subunit 3**	GTF2H3
0,00	1,04	2,06	**206,12**	Q15532	**Protein SSXT**	SS18
0,00	1,04	2,06	**205,86**	Q9UKI8	**Serine/threonine-protein kinase tousled-like 1**	TLK1
0,01	1,04	2,05	**205,15**	Q7Z3B3	**KAT8 regulatory NSL complex subunit 1**	KANSL1
0,02	1,03	2,04	**203,80**	Q01469;A8MUU1	**Fatty acid-binding protein, epidermal**	FABP5
0,00	1,03	2,04	**203,70**	O95359	**Transforming acidic coiled-coil-containing protein 2**	TACC2
0,01	1,03	2,04	**203,59**	Q9NVR2	**Integrator complex subunit 10**	INTS10
0,01	1,02	2,02	**202,24**	Q9H0B6	**Kinesin light chain 2**	KLC2
0,00	1,01	2,02	**201,91**	P05549;Q92481	**Transcription factor AP-2-alpha;Transcription factor AP-2-beta**	TFAP2A;TFAP2B
0,00	1,01	2,02	**201,64**	Q8WUQ7	**Uncharacterized protein C19orf29**	C19orf29
0,05	1,01	2,02	**201,52**	Q008S8	**Epithelial cell-transforming sequence 2 oncogene-like**	ECT2L
0,04	1,01	2,01	**201,21**	Q9BRD0	**BUD13 homolog**	BUD13
0,00	1,00	2,01	**200,60**	Q96JM7	**Lethal(3)malignant brain tumor-like protein 3**	L3MBTL3
0,00	1,00	2,00	**200,44**	Q06330	**Recombining binding protein suppressor of hairless**	RBPJ
0,01	1,00	2,00	**200,34**	Q01658	**Protein Dr1**	DR1
0,02	1,00	2,00	**199,86**	P05067	**Amyloid beta A4 protein;N-APP;Soluble APP-alpha;Soluble APP-beta;C99;Beta-amyl**	APP
0,05	1,00	2,00	**199,53**	Q32P28	**Prolyl 3-hydroxylase 1**	LEPRE1
0,05	1,00	1,99	**199,40**	Q5T749	**Keratinocyte proline-rich protein**	KPRP
0,02	1,00	1,99	**199,34**	Q9P260	**LisH domain and HEAT repeat-containing protein KIAA1468**	KIAA1468
0,00	0,99	1,99	**199,11**	O60885	**Bromodomain-containing protein 4**	BRD4
0,04	0,99	1,99	**198,97**	Q53EL6	**Programmed cell death protein 4**	PDCD4
0,00	0,99	1,99	**198,80**	Q9BVJ6;Q5TAP6	**U3 small nucleolar RNA-associated protein 14 homolog A**	UTP14A

0,01	0,99	1,98	**198,17**	P26583	**High mobility group protein B2**	HMGB2
0,04	0,98	1,98	**197,63**	Q76FK4	**Nucleolar protein 8**	NOL8
0,01	0,98	1,98	**197,61**	P08651	**Nuclear factor 1 C-type**	NFIC
0,00	0,98	1,97	**196,58**	Q9UPQ0	**LIM and calponin homology domains-containing protein 1**	LIMCH1
0,03	0,97	1,96	**196,30**	Q86VW0	**SEC14 domain and spectrin repeat-containing protein 1**	SESTD1
0,00	0,97	1,95	**195,35**	Q9UPQ3;Q96P47	**Arf-GAP with GTPase, ANK repeat and PH domain-containing protein 1**	AGAP1
0,02	0,97	1,95	**195,21**	P35222	**Catenin beta-1**	CTNNB1
0,04	0,96	1,95	**195,17**	Q75N03	**E3 ubiquitin-protein ligase Hakai**	CBLL1
0,02	0,96	1,95	**195,08**	P80723	**Brain acid soluble protein 1**	BASP1
0,00	0,96	1,95	**194,71**	Q02809	**Procollagen-lysine,2-oxoglutarate 5-dioxygenase 1**	PLOD1
0,00	0,96	1,94	**194,18**	Q674X7	**Kazrin**	KAZN
0,00	0,96	1,94	**194,11**	Q99961;Q99962	**Endophilin-A2**	SH3GL1
0,00	0,95	1,93	**193,46**	P46934	**E3 ubiquitin-protein ligase NEDD4**	NEDD4
0,00	0,95	1,93	**193,28**	Q03112	**MDS1 and EVI1 complex locus protein EVI1**	MECOM
0,02	0,95	1,93	**192,96**	Q8N3C0	**Activating signal cointegrator 1 complex subunit 3**	ASCC3
0,00	0,95	1,93	**192,52**	B1AK53	**Espin**	ESPN
0,00	0,94	1,91	**191,37**	P13674	**Prolyl 4-hydroxylase subunit alpha-1**	P4HA1
0,00	0,94	1,91	**191,30**	Q5TGY3	**AT-hook DNA-binding motif-containing protein 1**	AHDC1
0,00	0,94	1,91	**191,28**	Q16352;P41219;P12036	**Vimentin**	VIM
0,05	0,93	1,91	**191,04**	P60953	**Cell division control protein 42 homolog**	CDC42
0,03	0,93	1,91	**191,01**	O15116	**U6 snRNA-associated Sm-like protein LSm1**	LSM1
0,00	0,93	1,91	**190,53**	P35080	**Profilin-2**	PFN2
0,04	0,92	1,90	**189,63**	P06744	**Glucose-6-phosphate isomerase**	GPI
0,05	0,92	1,90	**189,61**	O00165	**HCLS1-associated protein X-1**	HAX1
0,00	0,92	1,90	**189,52**	Q15554	**Telomeric repeat-binding factor 2**	TERF2
0,02	0,92	1,89	**189,47**	O75376	**Nuclear receptor corepressor 1**	NCOR1
0,04	0,92	1,89	**189,08**	Q53F19	**Uncharacterized protein C17orf85**	C17orf85
0,04	0,92	1,89	**189,07**	P35270	**Sepiapterin reductase**	SPR
0,00	0,91	1,88	**188,39**	Q9BXP5	**Serrate RNA effector molecule homolog**	SRRT
0,04	0,91	1,88	**188,12**	O95067	**G2/mitotic-specific cyclin-B2**	CCNB2
0,00	0,91	1,88	**188,03**	Q92541	**RNA polymerase-associated protein RTF1 homolog**	RTF1
0,00	0,91	1,88	**188,01**	Q9HCK8	**Chromodomain-helicase-DNA-binding protein 8**	CHD8
0,02	0,91	1,88	**187,82**	Q9Y6R0	**Numb-like protein**	NUMBL

0,05	0,91	1,88	**187,67**	Q6RFH5	**WD repeat-containing protein 74**	WDR74
0,00	0,91	1,88	**187,55**	P98160	**Basement membrane-specific heparan sulfate proteoglycan core protein;Endorepell**	HSPG2
0,01	0,91	1,88	**187,52**	Q01167	**Forkhead box protein K2**	FOXK2
0,01	0,90	1,87	**187,16**	P67775	**Serine/threonine-protein phosphatase 2A catalytic subunit alpha isoform**	PPP2CA
0,04	0,90	1,87	**186,82**	O14686	**Histone-lysine N-methyltransferase MLL2**	MLL2
0,00	0,90	1,87	**186,79**	P61981	**14-3-3 protein gamma;14-3-3 protein gamma, N-terminally processed**	YWHAG
0,02	0,90	1,86	**186,30**	Q8NFW8	**N-acylneuraminate cytidylyltransferase**	CMAS
0,03	0,89	1,86	**185,91**	Q86VM9	**Zinc finger CCCH domain-containing protein 18**	ZC3H18
0,01	0,89	1,86	**185,90**	Q9UL33	**Trafficking protein particle complex subunit 2-like protein**	TRAPPC2L
0,00	0,89	1,86	**185,89**	P62805	**Histone H4**	HIST1H4A
0,01	0,89	1,86	**185,84**	O95391	**Pre-mRNA-splicing factor SLU7**	SLU7
0,01	0,89	1,86	**185,55**	P29084	**Transcription initiation factor IIE subunit beta**	GTF2E2
0,00	0,89	1,85	**185,43**	Q8TF68	**Zinc finger protein 384**	ZNF384
0,02	0,89	1,85	**185,35**	Q9NVH1	**DnaJ homolog subfamily C member 11**	DNAJC11
0,01	0,89	1,85	**184,84**	Q7Z5L9	**Interferon regulatory factor 2-binding protein 2**	IRF2BP2
0,01	0,88	1,84	**184,28**	Q96DH6	**RNA-binding protein Musashi homolog 2**	MSI2
0,03	0,88	1,84	**184,24**	P48507	**Glutamate--cysteine ligase regulatory subunit**	GCLM
0,00	0,87	1,83	**183,25**	O60828	**Polyglutamine-binding protein 1**	PQBP1
0,03	0,87	1,83	**182,82**	P63096	**Guanine nucleotide-binding protein G(i) subunit alpha-1**	GNAI1
0,05	0,87	1,82	**182,25**	Q7L7X3	**Serine/threonine-protein kinase TAO1**	TAOK1
0,01	0,86	1,82	**181,76**	Q96P16	**Regulation of nuclear pre-mRNA domain-containing protein 1A**	RPRD1A
0,05	0,86	1,82	**181,61**	P22626	**Heterogeneous nuclear ribonucleoproteins A2/B1**	HNRNPA2B1
0,01	0,86	1,81	**181,37**	O43809	**Cleavage and polyadenylation specificity factor subunit 5**	NUDT21
0,00	0,86	1,81	**181,19**	Q13501	**Sequestosome-1**	SQSTM1
0,00	0,86	1,81	**181,12**	P35052	**Glypican-1;Secreted glypican-1**	GPC1
0,00	0,86	1,81	**181,10**	Q16630	**Cleavage and polyadenylation specificity factor subunit 6**	CPSF6
0,02	0,85	1,81	**180,53**	P55268	**Laminin subunit beta-2**	LAMB2
0,04	0,85	1,80	**180,49**	P35680	**Hepatocyte nuclear factor 1-beta**	HNF1B
0,00	0,85	1,80	**180,32**	Q14978	**Nucleolar and coiled-body phosphoprotein 1**	NOLC1
0,02	0,85	1,80	**180,09**	Q3KQU3	**MAP7 domain-containing protein 1**	MAP7D1
0,01	0,85	1,80	**179,73**	Q9UMN6	**Histone-lysine N-methyltransferase MLL4**	WBP7
0,01	0,84	1,79	**179,47**	Q9Y2T2	**AP-3 complex subunit mu-1**	AP3M1
0,05	0,84	1,79	**179,08**	P18827	**Syndecan-1**	SDC1

0,00	0,84	1,79	**178,74**	Q8IY67	**Ribonucleoprotein PTB-binding 1**	RAVER1
0,00	0,84	1,79	**178,57**	P37275	**Zinc finger E-box-binding homeobox 1**	ZEB1
0,01	0,83	1,78	**178,04**	Q9UFF9	**CCR4-NOT transcription complex subunit 8**	CNOT8
0,02	0,83	1,78	**177,97**	Q75QN2	**Integrator complex subunit 8**	INTS8
0,00	0,83	1,78	**177,75**	Q96EV2	**RNA-binding protein 33**	RBM33
0,00	0,83	1,77	**177,38**	O75937	**DnaJ homolog subfamily C member 8**	DNAJC8
0,00	0,82	1,77	**176,53**	P82979	**SAP domain-containing ribonucleoprotein**	SARNP
0,00	0,82	1,77	**176,52**	P33240	**Cleavage stimulation factor subunit 2**	CSTF2
0,00	0,81	1,76	**175,81**	Q6SPF0	**Atherin**	SAMD1
0,03	0,81	1,76	**175,66**	Q99959	**Plakophilin-2**	PKP2
0,00	0,81	1,76	**175,61**	Q9UKV3	**Apoptotic chromatin condensation inducer in the nucleus**	ACIN1
0,00	0,81	1,75	**175,43**	Q5T8P6	**RNA-binding protein 26**	RBM26
0,00	0,81	1,75	**175,32**	P35221;Q9UI47	**Catenin alpha-1**	CTNNA1
0,04	0,81	1,75	**175,21**	P07311	**Acylphosphatase-1**	ACYP1
0,00	0,81	1,75	**175,09**	Q6PJG2	**Uncharacterized protein C14orf43**	C14orf43
0,02	0,81	1,75	**175,07**	P54578	**Ubiquitin carboxyl-terminal hydrolase 14**	USP14
0,01	0,81	1,75	**174,83**	Q6P6C2	**Probable alpha-ketoglutarate-dependent dioxygenase ABH5**	ALKBH5
0,01	0,80	1,74	**174,43**	Q7Z6E9	**E3 ubiquitin-protein ligase RBBP6**	RBBP6
0,02	0,80	1,74	**174,11**	P54274	**Telomeric repeat-binding factor 1**	TERF1
0,00	0,80	1,74	**173,96**	Q92925;Q6STE5	**SWI/SNF-related matrix-associated actin-dependent regulator of chromatin subfami**	SMARCD2
0,00	0,80	1,74	**173,92**	O94880	**PHD finger protein 14**	PHF14
0,01	0,79	1,73	**173,47**	P63165	**Small ubiquitin-related modifier 1**	SUMO1
0,00	0,79	1,73	**173,25**	Q6ZSJ8	**Uncharacterized protein C1orf122**	C1orf122
0,02	0,79	1,73	**173,23**	Q9NYB0	**Telomeric repeat-binding factor 2-interacting protein 1**	TERF2IP
0,01	0,79	1,72	**172,42**	P08237	**6-phosphofructokinase, muscle type**	PFKM
0,01	0,78	1,72	**172,29**	O43396	**Thioredoxin-like protein 1**	TXNL1
0,02	0,78	1,72	**172,23**	Q99569	**Plakophilin-4**	PKP4
0,01	0,77	1,71	**170,95**	O43670	**Zinc finger protein 207**	ZNF207
0,05	0,77	1,71	**170,81**	16777;Q9BTM1;Q96KK5	**Histone H2A type 1;Histone H2A type 1-D;Histone H2A type 2-A;Histone H2A type 2-**	HIST1H2AG;HIST1H
0,03	0,77	1,71	**170,79**	Q15208	**Serine/threonine-protein kinase 38**	STK38
0,02	0,77	1,71	**170,66**	Q9NQG5	**Regulation of nuclear pre-mRNA domain-containing protein 1B**	RPRD1B
0,02	0,77	1,70	**170,33**	O95218	**Zinc finger Ran-binding domain-containing protein 2**	ZRANB2
0,03	0,77	1,70	**170,31**	Q96ES7	**SAGA-associated factor 29 homolog**	CCDC101

0,01	0,77	1,70	**170,14**	P85037	**Forkhead box protein K1**	FOXK1
0,01	0,76	1,70	**169,88**	P27695	**DNA-(apurinic or apyrimidinic site) lyase;DNA-(apurinic or apyrimidinic site) lyase, n**	APEX1
0,00	0,76	1,69	**169,48**	Q15059	**Bromodomain-containing protein 3**	BRD3
0,02	0,76	1,69	**169,40**	Q14011	**Cold-inducible RNA-binding protein**	CIRBP
0,00	0,76	1,69	**169,27**	Q9NVN8	**Guanine nucleotide-binding protein-like 3-like protein**	GNL3L
0,03	0,76	1,69	**169,16**	Q96FK6	**WD repeat-containing protein 89**	WDR89
0,02	0,76	1,69	**168,94**	Q9HBM6	**Transcription initiation factor TFIID subunit 9B**	TAF9B
0,02	0,76	1,69	**168,84**	Q8TB45	**DEP domain-containing mTOR-interacting protein**	DEPTOR
0,00	0,75	1,68	**168,37**	Q96DI7	**U5 small nuclear ribonucleoprotein 40 kDa protein**	SNRNP40
0,04	0,75	1,68	**168,32**	Q6UX04	**Peptidyl-prolyl cis-trans isomerase CWC27 homolog**	CWC27
0,00	0,75	1,68	**168,06**	Q01518	**Adenylyl cyclase-associated protein 1**	CAP1
0,01	0,75	1,68	**167,71**	Q96CB8	**Integrator complex subunit 12**	INTS12
0,02	0,74	1,67	**167,49**	O00291	**Huntingtin-interacting protein 1**	HIP1
0,00	0,74	1,67	**167,41**	3;CON__H-INV:HIT0000	**Keratin, type I cytoskeletal 18**	KRT18
0,00	0,74	1,67	**166,66**	Q96C57	**Uncharacterized protein C12orf43**	C12orf43
0,01	0,74	1,67	**166,61**	Q7Z589	**Protein EMSY**	EMSY
0,02	0,74	1,67	**166,57**	O15347	**High mobility group protein B3**	HMGB3
0,03	0,74	1,66	**166,45**	Q9HCJ3	**Ribonucleoprotein PTB-binding 2**	RAVER2
0,01	0,73	1,66	**166,20**	Q9H8G2	**Caspase activity and apoptosis inhibitor 1**	CAAP1
0,00	0,73	1,66	**166,09**	Q9BZL4	**Protein phosphatase 1 regulatory subunit 12C**	PPP1R12C
0,00	0,73	1,66	**165,92**	P51608	**Methyl-CpG-binding protein 2**	MECP2
0,01	0,73	1,66	**165,83**	P00519	**Tyrosine-protein kinase ABL1**	ABL1
0,00	0,73	1,66	**165,77**	Q12888	**Tumor suppressor p53-binding protein 1**	TP53BP1
0,01	0,73	1,65	**165,37**	O43719	**HIV Tat-specific factor 1**	HTATSF1
0,03	0,72	1,65	**165,23**	P98095	**Fibulin-2**	FBLN2
0,01	0,72	1,65	**165,11**	Q8NEF9	**Serum response factor-binding protein 1**	SRFBP1
0,00	0,72	1,65	**165,00**	Q9NVC6	**Mediator of RNA polymerase II transcription subunit 17**	MED17
0,00	0,71	1,64	**163,97**	P01034	**Cystatin-C**	CST3
0,00	0,71	1,64	**163,69**	P39880	**Homeobox protein cut-like 1**	CUX1
0,03	0,71	1,64	**163,56**	P21127;Q9UQ88	**Cyclin-dependent kinase 11B;Cyclin-dependent kinase 11A**	CDK11B;CDK11A
0,00	0,71	1,63	**163,49**	Q5T5P2	**Sickle tail protein homolog**	SKT
0,00	0,71	1,63	**163,37**	P35580	**Myosin-10**	MYH10
0,01	0,71	1,63	**163,05**	Q9BRX2	**Protein pelota homolog**	PELO

0,02	0,70	1,63	**163,01**	Q15637	**Splicing factor 1**	SF1
0,02	0,70	1,63	**162,58**	Q7Z6R9	**Transcription factor AP-2-delta**	TFAP2D
0,00	0,70	1,62	**162,49**	Q14676	**Mediator of DNA damage checkpoint protein 1**	MDC1
0,00	0,70	1,62	**162,46**	Q99497	**Protein DJ-1**	PARK7
0,01	0,70	1,62	**162,37**	Q8NFC6;Q96IK1	**Biorientation of chromosomes in cell division protein 1-like 1**	BOD1L1
0,00	0,70	1,62	**162,30**	Q9NYF8	**Bcl-2-associated transcription factor 1**	BCLAF1
0,03	0,70	1,62	**162,27**	Q93034	**Cullin-5**	CUL5
0,01	0,70	1,62	**162,25**	Q96T60	**Bifunctional polynucleotide phosphatase/kinase;Polynucleotide 3-phosphatase;Poly**	PNKP
0,02	0,70	1,62	**162,17**	Q93099	**Homogentisate 1,2-dioxygenase**	HGD
0,01	0,70	1,62	**162,05**	Q14151	**Scaffold attachment factor B2**	SAFB2
0,01	0,70	1,62	**161,95**	Q9ULL5	**Proline-rich protein 12**	PRR12
0,01	0,69	1,62	**161,65**	Q92688	**Acidic leucine-rich nuclear phosphoprotein 32 family member B**	ANP32B
0,01	0,69	1,61	**161,06**	Q92785;Q92782	**Zinc finger protein ubi-d4**	DPF2
0,00	0,69	1,61	**160,93**	O15231	**Zinc finger protein 185**	ZNF185
0,00	0,69	1,61	**160,78**	P39748	**Flap endonuclease 1**	FEN1
0,00	0,68	1,61	**160,72**	Q06481	**Amyloid-like protein 2**	APLP2
0,04	0,68	1,61	**160,54**	O75962	**Triple functional domain protein**	TRIO
0,03	0,68	1,60	**160,37**	Q14687	**Genetic suppressor element 1**	GSE1
0,02	0,68	1,60	**160,27**	Q09028	**Histone-binding protein RBBP4**	RBBP4
0,03	0,67	1,60	**159,66**	P27797	**Calreticulin**	CALR
0,00	0,67	1,60	**159,56**	O75928	**E3 SUMO-protein ligase PIAS2**	PIAS2
0,02	0,67	1,59	**159,37**	Q6EEV6;P55854;P61956	**Small ubiquitin-related modifier 4;Small ubiquitin-related modifier 3;Small ubiquitir**	SUMO4;SUMO3;SU
0,00	0,67	1,59	**159,25**	P23193;Q15560	**Transcription elongation factor A protein 1**	TCEA1
0,02	0,67	1,59	**159,24**	Q13185	**Chromobox protein homolog 3**	CBX3
0,00	0,67	1,59	**159,15**	Q9UMS4	**Pre-mRNA-processing factor 19**	PRPF19
0,04	0,67	1,59	**159,12**	Q9NP50	**Protein FAM60A**	FAM60A
0,01	0,67	1,59	**158,88**	P30504	**HLA class I histocompatibility antigen, Cw-4 alpha chain**	HLA-C
0,00	0,66	1,58	**158,40**	Q86WB0	**Nuclear-interacting partner of ALK**	ZC3HC1
0,02	0,66	1,58	**158,22**	Q66PJ3	**ADP-ribosylation factor-like protein 6-interacting protein 4**	ARL6IP4
0,00	0,66	1,58	**158,02**	Q9NXV6	**CDKN2A-interacting protein**	CDKN2AIP
0,01	0,66	1,58	**157,61**	P60174	**Triosephosphate isomerase**	TPI1
0,01	0,66	1,58	**157,50**	Q13217	**DnaJ homolog subfamily C member 3**	DNAJC3
0,02	0,65	1,57	**157,41**	Q15652	**Probable JmjC domain-containing histone demethylation protein 2C**	JMJD1C

0,05	0,65	1,57	**157,40**	Q02086	**Transcription factor Sp2**	SP2
0,00	0,65	1,57	**157,37**	Q05682	**Caldesmon**	CALD1
0,00	0,65	1,57	**157,12**	Q16186	**Proteasomal ubiquitin receptor ADRM1**	ADRM1
0,04	0,65	1,57	**157,08**	Q8NAV1	**Pre-mRNA-splicing factor 38A**	PRPF38A
0,02	0,65	1,57	**156,92**	Q9UHB6	**LIM domain and actin-binding protein 1**	LIMA1
0,00	0,65	1,57	**156,82**	Q9UFW8	**CGG triplet repeat-binding protein 1**	CGGBP1
0,05	0,65	1,57	**156,79**	Q4VC44	**FLYWCH-type zinc finger-containing protein 1**	FLYWCH1
0,00	0,65	1,57	**156,76**	Q9BTC0	**Death-inducer obliterator 1**	DIDO1
0,00	0,65	1,57	**156,63**	P09661	**U2 small nuclear ribonucleoprotein A**	SNRPA1
0,00	0,65	1,57	**156,57**	P33176;O60282;Q1284(	**Kinesin-1 heavy chain**	KIF5B
0,00	0,64	1,56	**156,10**	Q9UHD8	**Septin-9**	40057,00
0,00	0,64	1,56	**156,08**	Q16181	**Septin-7**	39326,00
0,02	0,64	1,56	**155,93**	Q01085	**Nucleolysin TIAR**	TIAL1
0,02	0,64	1,56	**155,71**	P35251	**Replication factor C subunit 1**	RFC1
0,02	0,64	1,56	**155,52**	Q9UKD1	**Glucocorticoid modulatory element-binding protein 2**	GMEB2
0,05	0,64	1,55	**155,39**	Q5VT52	**Regulation of nuclear pre-mRNA domain-containing protein 2**	RPRD2
0,00	0,63	1,55	**155,22**	P49915	**GMP synthase [glutamine-hydrolyzing]**	GMPS
0,00	0,63	1,55	**155,01**	Q15906	**Vacuolar protein sorting-associated protein 72 homolog**	VPS72
0,01	0,63	1,55	**154,93**	P51571	**Translocon-associated protein subunit delta**	SSR4
0,04	0,63	1,55	**154,82**	Q9BXB4;Q9BXB5	**Oxysterol-binding protein-related protein 11**	OSBPL11
0,03	0,63	1,55	**154,77**	Q08945	**FACT complex subunit SSRP1**	SSRP1
0,05	0,63	1,55	**154,77**	P46100	**Transcriptional regulator ATRX**	ATRX
0,00	0,63	1,55	**154,73**	P06753	**Tropomyosin alpha-3 chain**	TPM3
0,00	0,63	1,54	**154,47**	P25440	**Bromodomain-containing protein 2**	BRD2
0,02	0,63	1,54	**154,31**	Q8NDX5	**Polyhomeotic-like protein 3**	PHC3
0,00	0,63	1,54	**154,31**	P48059;Q7Z4I7	**LIM and senescent cell antigen-like-containing domain protein 1;LIM and senescent**	LIMS1;LIMS2
0,01	0,62	1,54	**154,07**	Q99590	**Protein SCAF11**	SCAF11
0,03	0,62	1,54	**153,53**	O95833	**Chloride intracellular channel protein 3**	CLIC3
0,02	0,62	1,54	**153,52**	Q9ULR0	**Pre-mRNA-splicing factor ISY1 homolog**	ISY1
0,00	0,62	1,54	**153,50**	Q9Y277	**Voltage-dependent anion-selective channel protein 3**	VDAC3
0,04	0,61	1,53	**153,06**	Q9H4L4	**Sentrin-specific protease 3**	SENP3
0,04	0,61	1,53	**153,05**	Q96SB3	**Neurabin-2**	PPP1R9B
0,00	0,61	1,53	**152,53**	Q9UHF7	**Zinc finger transcription factor Trps1**	TRPS1

0,00	0,61	1,52	**152,37**	O15230	**Laminin subunit alpha-5**	LAMA5
0,01	0,60	1,52	**152,00**	P20810	**Calpastatin**	CAST
0,04	0,60	1,52	**151,90**	Q6P1N0	**Coiled-coil and C2 domain-containing protein 1A**	CC2D1A
0,02	0,60	1,51	**151,30**	Q9UHG0	**Doublecortin domain-containing protein 2**	DCDC2
0,02	0,60	1,51	**151,09**	P35637	**RNA-binding protein FUS**	FUS
0,00	0,59	1,51	**150,62**	P08572	**Collagen alpha-2(IV) chain;Canstatin**	COL4A2
0,04	0,59	1,50	**150,25**	O15047	**Histone-lysine N-methyltransferase SETD1A**	SETD1A
0,01	0,59	1,50	**150,17**	P00390	**Glutathione reductase, mitochondrial**	GSR
0,01	0,58	1,50	**149,98**	Q9UQR1	**Zinc finger protein 148**	ZNF148
0,01	0,58	1,49	**149,45**	P29372	**DNA-3-methyladenine glycosylase**	MPG
0,00	0,58	1,49	**149,36**	Q9H307	**Pinin**	PNN
0,00	0,58	1,49	**149,15**	Q9NVA2	**Septin-11**	40787,00
0,00	0,57	1,49	**148,97**	Q7Z6I8	**UPF0461 protein C5orf24**	C5orf24
0,01	0,57	1,49	**148,79**	P37837	**Transaldolase**	TALDO1
0,00	0,57	1,49	**148,68**	Q9H0L4	**Cleavage stimulation factor subunit 2 tau variant**	CSTF2T
0,04	0,57	1,48	**148,26**	P46087	**Putative ribosomal RNA methyltransferase NOP2**	NOP2
0,00	0,56	1,48	**147,84**	Q9Y2S0	**DNA-directed RNA polymerases I and III subunit RPAC2**	POLR1D
0,02	0,56	1,48	**147,82**	Q15599	**Na(+)/H(+) exchange regulatory cofactor NHE-RF2**	SLC9A3R2
0,00	0,56	1,48	**147,59**	Q6WCQ1	**Myosin phosphatase Rho-interacting protein**	MPRIP
0,01	0,56	1,47	**147,38**	P11047	**Laminin subunit gamma-1**	LAMC1
0,01	0,56	1,47	**147,34**	Q9UJU6	**Drebrin-like protein**	DBNL
0,01	0,56	1,47	**147,22**	Q93009	**Ubiquitin carboxyl-terminal hydrolase 7**	USP7
0,00	0,56	1,47	**147,17**	P98175	**RNA-binding protein 10**	RBM10
0,00	0,56	1,47	**147,15**	P28799	**Granulins;Acrogranin;Paragranulin;Granulin-1;Granulin-2;Granulin-3;Granulin-4;Gra**	GRN
0,00	0,56	1,47	**147,13**	O43290	**U4/U6.U5 tri-snRNP-associated protein 1**	SART1
0,04	0,55	1,47	**146,76**	Q9H0E3	**Histone deacetylase complex subunit SAP130**	SAP130
0,02	0,55	1,46	**146,48**	Q12959	**Disks large homolog 1**	DLG1
0,00	0,55	1,46	**146,46**	Q7Z3K3	**Pogo transposable element with ZNF domain**	POGZ
0,02	0,55	1,46	**146,08**	Q99547	**M-phase phosphoprotein 6**	MPHOSPH6
0,03	0,55	1,46	**145,91**	Q5T200	**Zinc finger CCCH domain-containing protein 13**	ZC3H13
0,00	0,54	1,46	**145,65**	P45880	**Voltage-dependent anion-selective channel protein 2**	VDAC2
0,00	0,54	1,46	**145,51**	P52907	**F-actin-capping protein subunit alpha-1**	CAPZA1
0,00	0,54	1,45	**145,15**	Q8N7H5	**RNA polymerase II-associated factor 1 homolog**	PAF1

0,00	0,54	1,45	**145,14**	P49750	**YLP motif-containing protein 1**	YLPM1
0,02	0,54	1,45	**144,94**	P43307	**Translocon-associated protein subunit alpha**	SSR1
0,00	0,53	1,45	**144,85**	Q96ST3	**Paired amphipathic helix protein Sin3a**	SIN3A
0,00	0,53	1,45	**144,83**	O43684	**Mitotic checkpoint protein BUB3**	BUB3
0,04	0,53	1,45	**144,68**	Q9UHD9	**Ubiquilin-2**	UBQLN2
0,00	0,53	1,44	**144,36**	Q13363	**C-terminal-binding protein 1**	CTBP1
0,00	0,53	1,44	**144,36**	Q9HC52	**Chromobox protein homolog 8**	CBX8
0,00	0,53	1,44	**144,31**	IM9;P0DMN0;P50225;P50226		
0,01	0,52	1,44	**143,85**	Q12830	**Nucleosome-remodeling factor subunit BPTF**	BPTF
0,01	0,52	1,44	**143,84**	P18621	**60S ribosomal protein L17**	RPL17
0,00	0,52	1,44	**143,76**	Q9Y6X9;Q86VD1	**MORC family CW-type zinc finger protein 2**	MORC2
0,05	0,52	1,44	**143,68**	Q8N4P3	**Guanosine-3,5-bis(diphosphate) 3-pyrophosphohydrolase MESH1**	HDDC3
0,01	0,52	1,43	**142,93**	Q9UQE7	**Structural maintenance of chromosomes protein 3**	SMC3
0,02	0,51	1,43	**142,70**	Q8WVC0	**RNA polymerase-associated protein LEO1**	LEO1
0,00	0,51	1,42	**142,32**	Q9H1B7	**Interferon regulatory factor 2-binding protein-like**	IRF2BPL
0,01	0,51	1,42	**142,14**	O43395	**U4/U6 small nuclear ribonucleoprotein Prp3**	PRPF3
0,00	0,51	1,42	**142,14**	Q8IX12	**Cell division cycle and apoptosis regulator protein 1**	CCAR1
0,02	0,51	1,42	**142,11**	Q08211	**ATP-dependent RNA helicase A**	DHX9
0,00	0,50	1,42	**141,89**	P35579	**Myosin-9**	MYH9
0,05	0,50	1,42	**141,84**	Q8IXM2	**Chromatin complexes subunit BAP18**	BAP18
0,02	0,50	1,42	**141,71**	Q9BZ95	**Histone-lysine N-methyltransferase NSD3**	WHSC1L1
0,00	0,50	1,42	**141,69**	Q9UQ35	**Serine/arginine repetitive matrix protein 2**	SRRM2
0,01	0,49	1,41	**140,79**	Q9NVM9	**Protein asunder homolog**	Asun
0,00	0,49	1,41	**140,78**	P52701	**DNA mismatch repair protein Msh6**	MSH6
0,00	0,49	1,41	**140,59**	P13807	**Glycogen [starch] synthase, muscle**	GYS1
0,04	0,49	1,41	**140,51**	O60292	**Signal-induced proliferation-associated 1-like protein 3**	SIPA1L3
0,00	0,49	1,40	**140,31**	Q86YP4	**Transcriptional repressor p66-alpha**	GATAD2A
0,03	0,49	1,40	**140,27**	Q9BUQ8	**Probable ATP-dependent RNA helicase DDX23**	DDX23
0,01	0,49	1,40	**139,98**	P22234	**Multifunctional protein ADE2;Phosphoribosylaminoimidazole-succinocarboxamide s**	PAICS
0,01	0,48	1,40	**139,92**	O00712	**Nuclear factor 1 B-type**	NFIB
0,05	0,48	1,40	**139,72**	A0AV96;Q9NQ94	**RNA-binding protein 47**	RBM47
0,03	0,48	1,40	**139,64**	P33991;Q9UJA3	**DNA replication licensing factor MCM4**	MCM4
0,01	0,48	1,39	**139,48**	Q12996	**Cleavage stimulation factor subunit 3**	CSTF3

0,00	0,48	1,39	**139,41**	P49756	**RNA-binding protein 25**	RBM25
0,01	0,48	1,39	**139,08**	Q969V3	**Nicalin**	NCLN
0,00	0,47	1,39	**138,98**	O76027	**Annexin A9**	ANXA9
0,03	0,47	1,39	**138,91**	Q9UBL3	**Set1/Ash2 histone methyltransferase complex subunit ASH2**	ASH2L
0,03	0,47	1,39	**138,69**	P35241	**Radixin**	RDX
0,00	0,47	1,39	**138,56**	Q8IVT2	**Uncharacterized protein C19orf21**	C19orf21
0,00	0,47	1,38	**138,42**	42;O15405;O94900;Q96	**TOX high mobility group box family member 4**	TOX4
0,02	0,46	1,38	**137,87**	Q9BRT2	**Mitochondrial nucleoid factor 1**	MNF1
0,01	0,46	1,38	**137,83**	Q9NWH9	**SAFB-like transcription modulator**	SLTM
0,00	0,46	1,38	**137,60**	Q5JWF2;P63092;P38405	**Guanine nucleotide-binding protein G(s) subunit alpha isoforms XLas;Guanine nucle**	GNAS
0,00	0,46	1,37	**137,34**	P18669;Q8N0Y7;P15259	**Phosphoglycerate mutase 1**	PGAM1
0,01	0,46	1,37	**137,32**	Q8N1G4	**Leucine-rich repeat-containing protein 47**	LRRC47
0,00	0,46	1,37	**137,17**	Q9P2E9	**Ribosome-binding protein 1**	RRBP1
0,01	0,45	1,37	**136,58**	P52756	**RNA-binding protein 5**	RBM5
0,00	0,45	1,37	**136,52**	Q12972	**Nuclear inhibitor of protein phosphatase 1;Activator of RNA decay**	PPP1R8
0,03	0,45	1,37	**136,51**	P07237	**Protein disulfide-isomerase**	P4HB
0,01	0,45	1,36	**136,47**	316;P01892;P16190;P16	**HLA class I histocompatibility antigen, A-68 alpha chain;HLA class I histocompatibilit**	HLA-A
0,00	0,45	1,36	**136,43**	Q14683	**Structural maintenance of chromosomes protein 1A**	SMC1A
0,03	0,45	1,36	**136,28**	Q15029	**116 kDa U5 small nuclear ribonucleoprotein component**	EFTUD2
0,00	0,45	1,36	**136,16**	O15294	**UDP-N-acetylglucosamine--peptide N-acetylglucosaminyltransferase 110 kDa subun**	OGT
0,01	0,44	1,36	**136,09**	P14923	**Junction plakoglobin**	JUP
0,01	0,44	1,36	**135,78**	Q14839	**Chromodomain-helicase-DNA-binding protein 4**	CHD4
0,05	0,44	1,36	**135,72**	O43823	**A-kinase anchor protein 8**	AKAP8
0,04	0,44	1,35	**135,39**	Q9UKM9	**RNA-binding protein Raly**	RALY
0,01	0,43	1,35	**134,57**	O75534	**Cold shock domain-containing protein E1**	CSDE1
0,02	0,43	1,34	**134,43**	O15014	**Zinc finger protein 609**	ZNF609
0,00	0,42	1,34	**133,87**	A6NHR9	**Structural maintenance of chromosomes flexible hinge domain-containing protein 1**	SMCHD1
0,00	0,42	1,34	**133,80**	Q01082	**Spectrin beta chain, brain 1**	SPTBN1
0,00	0,41	1,33	**132,78**	P12429	**Annexin A3**	ANXA3
0,02	0,41	1,33	**132,56**	Q13884	**Beta-1-syntrophin**	SNTB1
0,02	0,41	1,32	**132,50**	P15144	**Aminopeptidase N**	ANPEP
0,04	0,41	1,32	**132,49**	Q8N684	**Cleavage and polyadenylation specificity factor subunit 7**	CPSF7
0,02	0,41	1,32	**132,46**	O75940	**Survival of motor neuron-related-splicing factor 30**	SMNDC1

0,01	0,40	1,32	**132,38**	O60437	**Periplakin**	PPL
0,00	0,40	1,32	**132,36**	Q5VUA4	**Zinc finger protein 318**	ZNF318
0,02	0,40	1,32	**132,29**	Q1KMD3	**Heterogeneous nuclear ribonucleoprotein U-like protein 2**	HNRNPUL2
0,00	0,40	1,32	**132,01**	Q9BZZ5	**Apoptosis inhibitor 5**	API5
0,04	0,40	1,32	**131,66**	P63220	**40S ribosomal protein S21**	RPS21
0,00	0,40	1,32	**131,57**	P62879	**Guanine nucleotide-binding protein G(I)/G(S)/G(T) subunit beta-2**	GNB2
0,00	0,39	1,31	**130,94**	P33993	**DNA replication licensing factor MCM7**	MCM7
0,02	0,39	1,31	**130,83**	Q9H6F5	**Coiled-coil domain-containing protein 86**	CCDC86
0,00	0,39	1,31	**130,78**	Q8NBJ4	**Golgi membrane protein 1**	GOLM1
0,02	0,39	1,31	**130,62**	Q9NUU7;Q9UMR2	**ATP-dependent RNA helicase DDX19A;ATP-dependent RNA helicase DDX19B**	DDX19A;DDX19B
0,00	0,38	1,31	**130,54**	P09429;B2RPK0	**High mobility group protein B1;Putative high mobility group protein B1-like 1**	HMGB1;HMGB1P1
0,00	0,38	1,30	**130,49**	Q14980	**Nuclear mitotic apparatus protein 1**	NUMA1
0,04	0,38	1,30	**130,44**	Q5SSJ5	**Heterochromatin protein 1-binding protein 3**	HP1BP3
0,00	0,38	1,30	**130,24**	P11021	**78 kDa glucose-regulated protein**	HSPA5
0,02	0,38	1,30	**129,97**	P23229	**Integrin alpha-6;Integrin alpha-6 heavy chain;Integrin alpha-6 light chain**	ITGA6
0,05	0,38	1,30	**129,71**	Q6NZY4	**Zinc finger CCHC domain-containing protein 8**	ZCCHC8
0,02	0,37	1,30	**129,59**	P47755	**F-actin-capping protein subunit alpha-2**	CAPZA2
0,01	0,37	1,29	**129,49**	Q86UP2	**Kinectin**	KTN1
0,00	0,37	1,29	**129,49**	P12270	**Nucleoprotein TPR**	TPR
0,00	0,37	1,29	**129,27**	Q15149	**Plectin**	PLEC
0,01	0,37	1,29	**129,04**	Q15019	**Septin-2**	37500,00
0,02	0,37	1,29	**128,94**	Q9UJX3	**Anaphase-promoting complex subunit 7**	ANAPC7
0,03	0,37	1,29	**128,84**	P25205	**DNA replication licensing factor MCM3**	MCM3
0,00	0,36	1,29	**128,63**	P46939	**Utrophin**	UTRN
0,00	0,36	1,29	**128,51**	Q9Y230	**RuvB-like 2**	RUVBL2
0,00	0,36	1,28	**128,43**	Q9Y2L1	**Exosome complex exonuclease RRP44**	DIS3
0,03	0,36	1,28	**128,39**	Q9UHR5	**SAP30-binding protein**	SAP30BP
0,01	0,36	1,28	**128,31**	Q13868	**Exosome complex component RRP4**	EXOSC2
0,03	0,36	1,28	**127,98**	P40222	**Alpha-taxilin**	TXLNA
0,03	0,36	1,28	**127,97**	Q10570	**Cleavage and polyadenylation specificity factor subunit 1**	CPSF1
0,03	0,36	1,28	**127,92**	Q9H0S4	**Probable ATP-dependent RNA helicase DDX47**	DDX47
0,03	0,35	1,28	**127,85**	Q5TZA2;Q86T23	**Rootletin**	CROCC
0,03	0,35	1,28	**127,76**	Q15007	**Pre-mRNA-splicing regulator WTAP**	WTAP

0,05	0,35	1,27	**127,25**	439;P13746;P30443;P3(	**HLA class I histocompatibility antigen, A-3 alpha chain;HLA class I histocompatibility**	HLA-A
0,02	0,35	1,27	**127,08**	P21796	**Voltage-dependent anion-selective channel protein 1**	VDAC1
0,02	0,34	1,27	**126,63**	O60220	**Mitochondrial import inner membrane translocase subunit Tim8 A**	TIMM8A
0,02	0,33	1,26	**125,97**	Q86V48	**Leucine zipper protein 1**	LUZP1
0,00	0,33	1,26	**125,95**	Q9NZN4	**EH domain-containing protein 2**	EHD2
0,03	0,33	1,26	**125,85**	Q9BWF3	**RNA-binding protein 4**	RBM4
0,05	0,33	1,26	**125,65**	P09110	**3-ketoacyl-CoA thiolase, peroxisomal**	ACAA1
0,01	0,33	1,26	**125,60**	P43246	**DNA mismatch repair protein Msh2**	MSH2
0,01	0,33	1,26	**125,52**	Q9C0C2	**182 kDa tankyrase-1-binding protein**	TNKS1BP1
0,03	0,32	1,25	**125,11**	P24928	**DNA-directed RNA polymerase II subunit RPB1**	POLR2A
0,04	0,32	1,25	**125,06**	Q16204	**Coiled-coil domain-containing protein 6**	CCDC6
0,00	0,32	1,25	**124,90**	P15924	**Desmoplakin**	DSP
0,05	0,31	1,24	**124,13**	Q9H6S3	**Epidermal growth factor receptor kinase substrate 8-like protein 2**	EPS8L2
0,01	0,30	1,23	**123,44**	Q13813	**Spectrin alpha chain, brain**	SPTAN1
0,02	0,30	1,23	**123,27**	Q16891	**Mitochondrial inner membrane protein**	IMMT
0,00	0,30	1,23	**122,87**	O43491;Q9Y2J2	**Band 4.1-like protein 2**	EPB41L2
0,00	0,30	1,23	**122,71**	Q9Y6W5	**Wiskott-Aldrich syndrome protein family member 2**	WASF2
0,00	0,29	1,22	**121,99**	O94905	**Erlin-2**	ERLIN2
0,00	0,29	1,22	**121,95**	Q07157	**Tight junction protein ZO-1**	TJP1
0,05	0,28	1,21	**121,10**	Q9Y490	**Talin-1**	TLN1
0,04	0,27	1,21	**120,52**	Q8IX01	**SURP and G-patch domain-containing protein 2**	SUGP2
0,04	0,26	1,20	**120,12**	Q86UE4	**Protein LYRIC**	MTDH
0,02	0,26	1,20	**119,80**	Q8N163	**DBIRD complex subunit KIAA1967**	KIAA1967
0,01	0,25	1,19	**119,31**	O00159	**Unconventional myosin-Ic**	MYO1C
0,04	0,25	1,19	**119,16**	P09874	**Poly [ADP-ribose] polymerase 1**	PARP1
0,04	0,24	1,18	**118,17**	O00268;Q92750	**Transcription initiation factor TFIID subunit 4**	TAF4
0,04	0,13	1,10	**109,71**	Q00341	**Vigilin**	HDLBP
**8 h Down**						
0,000	-5,20	0,03	**2,72**	P82933	**28S ribosomal protein S9, mitochondrial**	MRPS9
0,009	-3,73	0,08	**7,52**	P11498	**Pyruvate carboxylase, mitochondrial**	PC
0,000	-3,54	0,09	**8,61**	Q8N5N7	**39S ribosomal protein L50, mitochondrial**	MRPL50
0,003	-3,49	0,09	**8,92**	O00763	**Acetyl-CoA carboxylase 2;Biotin carboxylase**	ACACB
0,001	-3,39	0,10	**9,57**	Q9BUT1	**3-hydroxybutyrate dehydrogenase type 2**	BDH2

0,004	-3,38	0,10	**9,60**	Q13247	**Serine/arginine-rich splicing factor 6**	SRSF6
0,024	-3,29	0,10	**10,21**	Q8NFF5	**FAD synthase;Molybdenum cofactor biosynthesis protein-like region;FAD synthase r**	FLAD1
0,000	-3,28	0,10	**10,29**	Q9H3P7	**Golgi resident protein GCP60**	ACBD3
0,002	-3,17	0,11	**11,12**	Q02952	**A-kinase anchor protein 12**	AKAP12
0,009	-3,15	0,11	**11,23**	Q9Y223	**Bifunctional UDP-N-acetylglucosamine 2-epimerase/N-acetylmannosamine kinase;U**	GNE
0,005	-3,14	0,11	**11,36**	P49585;Q9Y5K3	**Choline-phosphate cytidylyltransferase A;Choline-phosphate cytidylyltransferase B**	PCYT1A;PCYT1B
0,009	-3,13	0,11	**11,44**	P49257	**Protein ERGIC-53**	LMAN1
0,000	-3,11	0,12	**11,56**	O43837	**Isocitrate dehydrogenase [NAD] subunit beta, mitochondrial**	IDH3B
0,003	-2,98	0,13	**12,69**	Q9H0U6	**39S ribosomal protein L18, mitochondrial**	MRPL18
0,004	-2,96	0,13	**12,84**	O95202	**LETM1 and EF-hand domain-containing protein 1, mitochondrial**	LETM1
0,002	-2,94	0,13	**13,01**	Q7Z2W9	**39S ribosomal protein L21, mitochondrial**	MRPL21
0,041	-2,94	0,13	**13,04**	Q8IX01	**SURP and G-patch domain-containing protein 2**	SUGP2
0,010	-2,94	0,13	**13,08**	P21912	**Succinate dehydrogenase [ubiquinone] iron-sulfur subunit, mitochondrial**	SDHB
0,049	-2,91	0,13	**13,28**	P61981	**14-3-3 protein gamma;14-3-3 protein gamma, N-terminally processed**	YWHAG
0,030	-2,85	0,14	**13,91**	Q03252	**Lamin-B2**	LMNB2
0,001	-2,80	0,14	**14,35**	Q9Y2R9	**28S ribosomal protein S7, mitochondrial**	MRPS7
0,019	-2,80	0,14	**14,38**	Q4G0N4	**NAD kinase domain-containing protein 1**	NADKD1
0,002	-2,77	0,15	**14,61**	Q8TAE8	**Growth arrest and DNA damage-inducible proteins-interacting protein 1**	GADD45GIP1
0,005	-2,77	0,15	**14,68**	Q16890	**Tumor protein D53**	TPD52L1
0,031	-2,77	0,15	**14,68**	P53041	**Serine/threonine-protein phosphatase 5**	PPP5C
0,004	-2,75	0,15	**14,88**	Q9P0M9	**39S ribosomal protein L27, mitochondrial**	MRPL27
0,004	-2,75	0,15	**14,89**	O14646	**Chromodomain-helicase-DNA-binding protein 1**	CHD1
0,042	-2,72	0,15	**15,18**	Q04206	**Transcription factor p65**	RELA
0,012	-2,72	0,15	**15,19**	P11177	**Pyruvate dehydrogenase E1 component subunit beta, mitochondrial**	PDHB
0,002	-2,70	0,15	**15,37**	P82914	**28S ribosomal protein S15, mitochondrial**	MRPS15
0,008	-2,68	0,16	**15,59**	Q9NSE4	**Isoleucine--tRNA ligase, mitochondrial**	IARS2
0,026	-2,67	0,16	**15,69**	Q5T653	**39S ribosomal protein L2, mitochondrial**	MRPL2
0,000	-2,65	0,16	**15,93**	Q96JH7	**Deubiquitinating protein VCIP135**	VCPIP1
0,006	-2,61	0,16	**16,35**	Q96FW1	**Ubiquitin thioesterase OTUB1**	OTUB1
0,000	-2,61	0,16	**16,35**	P50579	**Methionine aminopeptidase 2**	METAP2
0,015	-2,61	0,16	**16,43**	Q8WUX9	**Charged multivesicular body protein 7**	CHMP7
0,027	-2,58	0,17	**16,71**	O60493	**Sorting nexin-3**	SNX3
0,024	-2,56	0,17	**17,01**	Q8N5M4	**Tetratricopeptide repeat protein 9C**	TTC9C

0,001	-2,55	0,17	**17,03**	P11532	**Dystrophin**	DMD
0,039	-2,53	0,17	**17,35**	P63151;Q00005;Q9Y2T4	**Serine/threonine-protein phosphatase 2A 55 kDa regulatory subunit B alpha isoform**	PPP2R2A
0,046	-2,51	0,18	**17,53**	Q2TBE0	**CWF19-like protein 2**	CWF19L2
0,001	-2,51	0,18	**17,54**	P51398	**28S ribosomal protein S29, mitochondrial**	DAP3
0,010	-2,50	0,18	**17,65**	Q92783	**Signal transducing adapter molecule 1**	STAM
0,045	-2,49	0,18	**17,76**	O14530	**Thioredoxin domain-containing protein 9**	TXNDC9
0,021	-2,46	0,18	**18,12**	Q9H2G2	**STE20-like serine/threonine-protein kinase**	SLK
0,011	-2,46	0,18	**18,13**	Q9Y3B2	**Exosome complex component CSL4**	EXOSC1
0,004	-2,45	0,18	**18,36**	Q99798	**Aconitate hydratase, mitochondrial**	ACO2
0,002	-2,43	0,19	**18,60**	P31040	**Succinate dehydrogenase [ubiquinone] flavoprotein subunit, mitochondrial**	SDHA
0,011	-2,42	0,19	**18,66**	Q9NR45	**Sialic acid synthase**	NANS
0,016	-2,40	0,19	**18,89**	Q13564	**NEDD8-activating enzyme E1 regulatory subunit**	NAE1
0,015	-2,40	0,19	**18,91**	Q15785	**Mitochondrial import receptor subunit TOM34**	TOMM34
0,007	-2,40	0,19	**18,92**	A0AVT1	**Ubiquitin-like modifier-activating enzyme 6**	UBA6
0,014	-2,39	0,19	**19,11**	Q9Y450	**HBS1-like protein**	HBS1L
0,006	-2,39	0,19	**19,13**	P49321	**Nuclear autoantigenic sperm protein**	NASP
0,001	-2,38	0,19	**19,15**	Q99598	**Translin-associated protein X**	TSNAX
0,040	-2,37	0,19	**19,33**	P49773	**Histidine triad nucleotide-binding protein 1**	HINT1
0,003	-2,36	0,20	**19,52**	Q9Y2R0	**Coiled-coil domain-containing protein 56**	CCDC56
0,043	-2,35	0,20	**19,63**	Q9HDC9	**Adipocyte plasma membrane-associated protein**	APMAP
0,031	-2,33	0,20	**19,89**	P17174	**Aspartate aminotransferase, cytoplasmic**	GOT1
0,006	-2,33	0,20	**19,92**	Q13085	**Acetyl-CoA carboxylase 1;Biotin carboxylase**	ACACA
0,007	-2,32	0,20	**19,97**	P10515	**Dihydrolipoyllysine-residue acetyltransferase component of pyruvate dehydrogenas**	DLAT
0,000	-2,32	0,20	**20,00**	Q13795	**ADP-ribosylation factor-related protein 1**	ARFRP1
0,018	-2,29	0,20	**20,43**	Q9NZT1	**Calmodulin-like protein 5**	CALML5
0,020	-2,29	0,20	**20,44**	Q9UL63	**Muskelin**	MKLN1
0,004	-2,28	0,21	**20,61**	Q96A35	**39S ribosomal protein L24, mitochondrial**	MRPL24
0,007	-2,27	0,21	**20,66**	O95817	**BAG family molecular chaperone regulator 3**	BAG3
0,011	-2,27	0,21	**20,66**	P00387	**NADH-cytochrome b5 reductase 3;NADH-cytochrome b5 reductase 3 membrane-bo**	CYB5R3
0,006	-2,27	0,21	**20,78**	P22059	**Oxysterol-binding protein 1**	OSBP
0,004	-2,26	0,21	**20,82**	Q99707	**Methionine synthase**	MTR
0,011	-2,26	0,21	**20,82**	P31151;Q86SG5	**Protein S100-A7**	S100A7
0,001	-2,26	0,21	**20,93**	P06737	**Glycogen phosphorylase, liver form**	PYGL

0,006	-2,25	0,21	**20,97**	P16144	**Integrin beta-4**	ITGB4
0,011	-2,25	0,21	**20,99**	P60891	**Ribose-phosphate pyrophosphokinase 1**	PRPS1
0,003	-2,25	0,21	**21,07**	Q96GC5	**39S ribosomal protein L48, mitochondrial**	MRPL48
0,023	-2,25	0,21	**21,09**	Q01581	**Hydroxymethylglutaryl-CoA synthase, cytoplasmic**	HMGCS1
0,036	-2,24	0,21	**21,12**	Q8IXM3	**39S ribosomal protein L41, mitochondrial**	MRPL41
0,025	-2,24	0,21	**21,18**	Q9H444	**Charged multivesicular body protein 4b**	CHMP4B
0,017	-2,24	0,21	**21,21**	O15355	**Protein phosphatase 1G**	PPM1G
0,011	-2,23	0,21	**21,26**	Q9UIS9	**Methyl-CpG-binding domain protein 1**	MBD1
0,007	-2,21	0,22	**21,56**	P07954	**Fumarate hydratase, mitochondrial**	FH
0,003	-2,20	0,22	**21,84**	Q8N3F8	**MICAL-like protein 1**	MICALL1
0,030	-2,19	0,22	**21,88**	P01034	**Cystatin-C**	CST3
0,015	-2,18	0,22	**22,05**	Q16851	**UTP--glucose-1-phosphate uridylyltransferase**	UGP2
0,020	-2,18	0,22	**22,14**	O43765	**Small glutamine-rich tetratricopeptide repeat-containing protein alpha**	SGTA
0,008	-2,17	0,22	**22,15**	O43252	**Bifunctional 3-phosphoadenosine 5-phosphosulfate synthase 1;Sulfate adenylyltran**	PAPSS1
0,003	-2,17	0,22	**22,20**	Q9NYF8	**Bcl-2-associated transcription factor 1**	BCLAF1
0,016	-2,17	0,22	**22,21**	P36915	**Guanine nucleotide-binding protein-like 1**	GNL1
0,006	-2,16	0,22	**22,34**	P12268	**Inosine-5-monophosphate dehydrogenase 2**	IMPDH2
0,029	-2,15	0,22	**22,46**	Q8N5A5	**Zinc finger CCCH-type with G patch domain-containing protein**	ZGPAT
0,042	-2,15	0,22	**22,48**	O14964	**Hepatocyte growth factor-regulated tyrosine kinase substrate**	HGS
0,030	-2,13	0,23	**22,82**	Q9BRA2	**Thioredoxin domain-containing protein 17**	TXNDC17
0,017	-2,13	0,23	**22,84**	P30405	**Peptidyl-prolyl cis-trans isomerase F, mitochondrial**	PPIF
0,001	-2,12	0,23	**22,94**	Q8NF50	**Dedicator of cytokinesis protein 8**	DOCK8
0,031	-2,12	0,23	**23,06**	P51148;P20339;P61020	**Ras-related protein Rab-5C;Ras-related protein Rab-5A;Ras-related protein Rab-5B**	RAB5C;RAB5A;RAB
0,038	-2,12	0,23	**23,07**	Q14257	**Reticulocalbin-2**	RCN2
0,029	-2,11	0,23	**23,16**	P22307	**Non-specific lipid-transfer protein**	SCP2
0,016	-2,10	0,23	**23,30**	Q12965;O00160	**Unconventional myosin-Ie**	MYO1E
0,003	-2,10	0,23	**23,38**	Q96DZ1	**Endoplasmic reticulum lectin 1**	ERLEC1
0,031	-2,09	0,24	**23,52**	Q9H2W6	**39S ribosomal protein L46, mitochondrial**	MRPL46
0,009	-2,09	0,24	**23,56**	Q9P2T1	**GMP reductase 2**	GMPR2
0,001	-2,08	0,24	**23,63**	Q96G25	**Mediator of RNA polymerase II transcription subunit 8**	MED8
0,015	-2,07	0,24	**23,75**	Q9UMS0	**NFU1 iron-sulfur cluster scaffold homolog, mitochondrial**	NFU1
0,041	-2,07	0,24	**23,77**	Q5JRX3	**Presequence protease, mitochondrial**	PITRM1
0,016	-2,06	0,24	**23,94**	P14550	**Alcohol dehydrogenase [NADP(+)]**	AKR1A1

0,007	-2,06	0,24	**24,04**	O00748	**Cocaine esterase**	CES2
0,002	-2,05	0,24	**24,12**	Q96EL3	**39S ribosomal protein L53, mitochondrial**	MRPL53
0,027	-2,05	0,24	**24,12**	P49023	**Paxillin**	PXN
0,049	-2,05	0,24	**24,16**	Q9P013	**Protein CWC15 homolog**	CWC15
0,037	-2,05	0,24	**24,19**	Q9HCC0	**Methylcrotonoyl-CoA carboxylase beta chain, mitochondrial**	MCCC2
0,001	-2,05	0,24	**24,23**	O00469	**Procollagen-lysine,2-oxoglutarate 5-dioxygenase 2**	PLOD2
0,042	-2,04	0,24	**24,28**	Q9Y653	**G-protein coupled receptor 56**	GPR56
0,007	-2,04	0,24	**24,29**	Q9UNH7;Q86XE0	**Sorting nexin-6**	SNX6
0,019	-2,04	0,24	**24,30**	Q96BQ5	**Coiled-coil domain-containing protein 127**	CCDC127
0,011	-2,04	0,24	**24,34**	Q9GZT3	**SRA stem-loop-interacting RNA-binding protein, mitochondrial**	SLIRP
0,032	-2,03	0,24	**24,42**	Q8WUM4	**Programmed cell death 6-interacting protein**	PDCD6IP
0,046	-2,03	0,25	**24,50**	Q8IVM0	**Coiled-coil domain-containing protein 50**	CCDC50
0,014	-2,03	0,25	**24,51**	P62993	**Growth factor receptor-bound protein 2**	GRB2
0,026	-2,03	0,25	**24,51**	Q8TEX9	**Importin-4**	IPO4
0,010	-2,01	0,25	**24,86**	Q8ND24	**RING finger protein 214**	RNF214
0,010	-2,01	0,25	**24,88**	Q99733	**Nucleosome assembly protein 1-like 4**	NAP1L4
0,032	-2,00	0,25	**24,91**	O43617	**Trafficking protein particle complex subunit 3**	TRAPPC3
0,044	-2,00	0,25	**24,95**	P62899	**60S ribosomal protein L31**	RPL31
0,044	-2,00	0,25	**25,05**	Q96C86	**m7GpppX diphosphatase**	DCPS
0,043	-1,99	0,25	**25,09**	P60033	**CD81 antigen**	CD81
0,006	-1,98	0,25	**25,32**	P05165	**Propionyl-CoA carboxylase alpha chain, mitochondrial**	PCCA
0,001	-1,98	0,25	**25,35**	Q9Y2Z0	**Suppressor of G2 allele of SKP1 homolog**	SUGT1
0,024	-1,98	0,25	**25,35**	[illegible]W1;P20340;Q9H0N0;Q[illegible]	**Ras-related protein Rab-6B;Ras-related protein Rab-6A**	RAB6B;RAB6A
0,000	-1,97	0,26	**25,60**	Q13214	**Semaphorin-3B**	SEMA3B
0,009	-1,95	0,26	**25,91**	P00568;Q9Y6K8	**Adenylate kinase isoenzyme 1**	AK1
0,041	-1,94	0,26	**26,01**	Q14019	**Coactosin-like protein**	COTL1
0,001	-1,94	0,26	**26,01**	P28838	**Cytosol aminopeptidase**	LAP3
0,046	-1,92	0,26	**26,35**	Q8NBN7	**Retinol dehydrogenase 13**	RDH13
0,018	-1,92	0,26	**26,39**	Q9ULT8	**E3 ubiquitin-protein ligase HECTD1**	HECTD1
0,038	-1,92	0,26	**26,41**	O95336	**6-phosphogluconolactonase**	PGLS
0,028	-1,91	0,27	**26,57**	O95834	**Echinoderm microtubule-associated protein-like 2**	EML2
0,025	-1,91	0,27	**26,59**	P10074	**Zinc finger and BTB domain-containing protein 48**	ZBTB48
0,006	-1,91	0,27	**26,66**	P30085	**UMP-CMP kinase**	CMPK1

0,041	-1,90	0,27	**26,74**	Q8TDH9	**Protein Muted homolog**	MUTED
0,005	-1,90	0,27	**26,77**	O15173	**Membrane-associated progesterone receptor component 2**	PGRMC2
0,002	-1,90	0,27	**26,78**	P49419	**Alpha-aminoadipic semialdehyde dehydrogenase**	ALDH7A1
0,038	-1,89	0,27	**26,89**	Q8TBC4	**NEDD8-activating enzyme E1 catalytic subunit**	UBA3
0,029	-1,89	0,27	**26,91**	Q8WW12	**PEST proteolytic signal-containing nuclear protein**	PCNP
0,016	-1,89	0,27	**26,96**	Q9UHD1	**Cysteine and histidine-rich domain-containing protein 1**	CHORDC1
0,047	-1,88	0,27	**27,09**	P04632;Q96L46	**Calpain small subunit 1**	CAPNS1
0,005	-1,88	0,27	**27,18**	Q5T5X7	**BEN domain-containing protein 3**	BEND3
0,043	-1,88	0,27	**27,25**	Q08257	**Quinone oxidoreductase**	CRYZ
0,031	-1,86	0,27	**27,45**	Q13596	**Sorting nexin-1**	SNX1
0,023	-1,86	0,27	**27,48**	Q96IX5	**Up-regulated during skeletal muscle growth protein 5**	USMG5
0,035	-1,86	0,27	**27,50**	P30740	**Leukocyte elastase inhibitor**	SERPINB1
0,002	-1,86	0,28	**27,57**	Q9H814	**Phosphorylated adapter RNA export protein**	PHAX
0,020	-1,86	0,28	**27,60**	\6NCE7;Q9GZQ8;Q9H49	**Microtubule-associated proteins 1A/1B light chain 3 beta 2;Microtubule-associated**	MAP1LC3B2;MAP1
0,038	-1,86	0,28	**27,63**	P23921	**Ribonucleoside-diphosphate reductase large subunit**	RRM1
0,003	-1,85	0,28	**27,65**	P21281;P15313	**V-type proton ATPase subunit B, brain isoform**	ATP6V1B2
0,012	-1,85	0,28	**27,71**	P29084	**Transcription initiation factor IIE subunit beta**	GTF2E2
0,001	-1,85	0,28	**27,77**	Q13409	**Cytoplasmic dynein 1 intermediate chain 2**	DYNC1I2
0,004	-1,84	0,28	**27,85**	P27487	**Dipeptidyl peptidase 4;Dipeptidyl peptidase 4 membrane form;Dipeptidyl peptidase**	DPP4
0,016	-1,83	0,28	**28,09**	Q8NFU5	**Inositol polyphosphate multikinase**	IPMK
0,016	-1,83	0,28	**28,14**	Q14116	**Interleukin-18**	IL18
0,014	-1,83	0,28	**28,17**	P82650	**28S ribosomal protein S22, mitochondrial**	MRPS22
0,046	-1,82	0,28	**28,23**	Q5RKV6	**Exosome complex component MTR3**	EXOSC6
0,027	-1,82	0,28	**28,28**	Q969S3	**Zinc finger protein 622**	ZNF622
0,021	-1,82	0,28	**28,33**	O00116	**Alkyldihydroxyacetonephosphate synthase, peroxisomal**	AGPS
0,036	-1,81	0,29	**28,58**	Q96L73	**Histone-lysine N-methyltransferase, H3 lysine-36 and H4 lysine-20 specific**	NSD1
0,022	-1,80	0,29	**28,71**	Q7Z422	**UPF0485 protein C1orf144**	C1orf144
0,037	-1,80	0,29	**28,74**	Q93062	**RNA-binding protein with multiple splicing**	RBPMS
0,020	-1,80	0,29	**28,76**	Q9P032	**NADH dehydrogenase [ubiquinone] 1 alpha subcomplex assembly factor 4**	NDUFAF4
0,007	-1,79	0,29	**28,90**	Q99614	**Tetratricopeptide repeat protein 1**	TTC1
0,031	-1,78	0,29	**29,11**	Q08752	**Peptidyl-prolyl cis-trans isomerase D**	PPID
0,016	-1,78	0,29	**29,14**	O15460	**Prolyl 4-hydroxylase subunit alpha-2**	P4HA2
0,010	-1,78	0,29	**29,19**	Q9BZE1	**39S ribosomal protein L37, mitochondrial**	MRPL37

0,009	-1,77	0,29	**29,24**	O75947	**ATP synthase subunit d, mitochondrial**	ATP5H
0,018	-1,77	0,29	**29,28**	Q13938	**Calcyphosin**	CAPS
0,035	-1,77	0,29	**29,30**	P22681	**E3 ubiquitin-protein ligase CBL**	CBL
0,014	-1,77	0,29	**29,32**	O76031	**ATP-dependent Clp protease ATP-binding subunit clpX-like, mitochondrial**	CLPX
0,002	-1,77	0,29	**29,34**	Q13084	**39S ribosomal protein L28, mitochondrial**	MRPL28
0,002	-1,77	0,29	**29,36**	P82673	**28S ribosomal protein S35, mitochondrial**	MRPS35
0,010	-1,77	0,29	**29,37**	Q96CU9	**FAD-dependent oxidoreductase domain-containing protein 1**	FOXRED1
0,014	-1,77	0,29	**29,38**	Q12962	**Transcription initiation factor TFIID subunit 10**	TAF10
0,005	-1,77	0,29	**29,38**	P62699	**Protein yippee-like 5**	YPEL5
0,029	-1,77	0,29	**29,41**	P57105	**Synaptojanin-2-binding protein**	SYNJ2BP
0,003	-1,76	0,29	**29,49**	Q16762	**Thiosulfate sulfurtransferase**	TST
0,038	-1,76	0,30	**29,57**	Q8NBF2	**NHL repeat-containing protein 2**	NHLRC2
0,029	-1,76	0,30	**29,59**	Q9Y266	**Nuclear migration protein nudC**	NUDC
0,000	-1,74	0,30	**29,85**	Q8NBJ4	**Golgi membrane protein 1**	GOLM1
0,045	-1,74	0,30	**29,85**	P14735	**Insulin-degrading enzyme**	IDE
0,048	-1,74	0,30	**29,93**	P20839	**Inosine-5-monophosphate dehydrogenase 1**	IMPDH1
0,049	-1,74	0,30	**29,97**	P38117	**Electron transfer flavoprotein subunit beta**	ETFB
0,033	-1,73	0,30	**30,08**	Q9Y5P6	**Mannose-1-phosphate guanyltransferase beta**	GMPPB
0,013	-1,73	0,30	**30,22**	O14907	**Tax1-binding protein 3**	TAX1BP3
0,044	-1,73	0,30	**30,24**	Q9UBQ0	**Vacuolar protein sorting-associated protein 29**	VPS29
0,039	-1,72	0,30	**30,25**	P53367	**Arfaptin-1**	ARFIP1
0,000	-1,72	0,30	**30,35**	Q8N983	**39S ribosomal protein L43, mitochondrial**	MRPL43
0,046	-1,72	0,30	**30,40**	Q96DB5	**Regulator of microtubule dynamics protein 1**	FAM82B
0,011	-1,72	0,30	**30,44**	Q96GM8	**Target of EGR1 protein 1**	TOE1
0,000	-1,71	0,31	**30,54**	P16989	**DNA-binding protein A**	CSDA
0,030	-1,71	0,31	**30,58**	Q8IXQ6	**Poly [ADP-ribose] polymerase 9**	PARP9
0,041	-1,70	0,31	**30,68**	Q9HD33	**39S ribosomal protein L47, mitochondrial**	MRPL47
0,001	-1,70	0,31	**30,72**	Q9NX40	**OCIA domain-containing protein 1**	OCIAD1
0,038	-1,70	0,31	**30,79**	O00154	**Cytosolic acyl coenzyme A thioester hydrolase**	ACOT7
0,026	-1,70	0,31	**30,80**	P78417	**Glutathione S-transferase omega-1**	GSTO1
0,013	-1,70	0,31	**30,83**	Q96S19	**UPF0585 protein C16orf13**	C16orf13
0,036	-1,69	0,31	**30,89**	Q15388	**Mitochondrial import receptor subunit TOM20 homolog**	TOMM20
0,036	-1,69	0,31	**30,90**	Q9BYT8	**Neurolysin, mitochondrial**	NLN

0,028	-1,69	0,31	**30,94**	P00966	**Argininosuccinate synthase**	ASS1
0,003	-1,69	0,31	**30,97**	P48047	**ATP synthase subunit O, mitochondrial**	ATP5O
0,007	-1,69	0,31	**30,98**	P46379	**Large proline-rich protein BAG6**	BAG6
0,001	-1,69	0,31	**31,10**	Q92890	**Ubiquitin fusion degradation protein 1 homolog**	UFD1L
0,010	-1,68	0,31	**31,12**	O15067	**Phosphoribosylformylglycinamidine synthase**	PFAS
0,047	-1,68	0,31	**31,27**	Q92526	**T-complex protein 1 subunit zeta-2**	CCT6B
0,015	-1,68	0,31	**31,29**	P30533	**Alpha-2-macroglobulin receptor-associated protein**	LRPAP1
0,001	-1,67	0,31	**31,39**	Q96DV4	**39S ribosomal protein L38, mitochondrial**	MRPL38
0,035	-1,67	0,31	**31,40**	P58546	**Myotrophin**	MTPN
0,029	-1,67	0,31	**31,43**	Q14376	**UDP-glucose 4-epimerase**	GALE
0,027	-1,67	0,32	**31,53**	Q8N183	**Mimitin, mitochondrial**	NDUFAF2
0,003	-1,66	0,32	**31,55**	P28072	**Proteasome subunit beta type-6**	PSMB6
0,025	-1,66	0,32	**31,61**	Q9BXR0	**Queuine tRNA-ribosyltransferase**	QTRT1
0,042	-1,66	0,32	**31,61**	Q96HE7;Q86YB8	**ERO1-like protein alpha**	ERO1L
0,023	-1,66	0,32	**31,63**	P31431	**Syndecan-4**	SDC4
0,005	-1,66	0,32	**31,66**	P21980	**Protein-glutamine gamma-glutamyltransferase 2**	TGM2
0,019	-1,65	0,32	**31,77**	Q9BT30	**Probable alpha-ketoglutarate-dependent dioxygenase ABH7**	ALKBH7
0,010	-1,65	0,32	**31,82**	P06132	**Uroporphyrinogen decarboxylase**	UROD
0,008	-1,65	0,32	**31,83**	Q14197	**Peptidyl-tRNA hydrolase ICT1, mitochondrial**	ICT1
0,030	-1,65	0,32	**31,90**	Q9H8S9	**MOB kinase activator 1A**	MOB1A
0,022	-1,65	0,32	**31,90**	Q14697	**Neutral alpha-glucosidase AB**	GANAB
0,021	-1,65	0,32	**31,92**	Q16204	**Coiled-coil domain-containing protein 6**	CCDC6
0,045	-1,65	0,32	**31,94**	P49356	**Protein farnesyltransferase subunit beta**	FNTB
0,019	-1,64	0,32	**32,00**	P27708;P31327	**CAD protein;Glutamine-dependent carbamoyl-phosphate synthase;Aspartate carba**	CAD
0,009	-1,64	0,32	**32,13**	Q86VX2	**COMM domain-containing protein 7**	COMMD7
0,018	-1,64	0,32	**32,15**	Q02818	**Nucleobindin-1**	NUCB1
0,024	-1,63	0,32	**32,25**	Q7Z6Z7	**E3 ubiquitin-protein ligase HUWE1**	HUWE1
0,001	-1,63	0,32	**32,31**	O94979	**Protein transport protein Sec31A**	SEC31A
0,042	-1,63	0,32	**32,40**	P55327	**Tumor protein D52**	TPD52
0,021	-1,62	0,32	**32,43**	O75348;O95670	**V-type proton ATPase subunit G 1;V-type proton ATPase subunit G 2**	ATP6V1G1;ATP6V1
0,034	-1,62	0,33	**32,54**	P41250	**Glycine--tRNA ligase**	GARS
0,013	-1,61	0,33	**32,69**	P49327	**Fatty acid synthase;[Acyl-carrier-protein] S-acetyltransferase;[Acyl-carrier-protein] S**	FASN
0,011	-1,61	0,33	**32,78**	P06730	**Eukaryotic translation initiation factor 4E**	EIF4E

0,016	-1,61	0,33	**32,79**	Q9H074	**Polyadenylate-binding protein-interacting protein 1**	PAIP1
0,012	-1,61	0,33	**32,82**	Q15370	**Transcription elongation factor B polypeptide 2**	TCEB2
0,027	-1,60	0,33	**32,96**	Q9UEW8	**STE20/SPS1-related proline-alanine-rich protein kinase**	STK39
0,016	-1,60	0,33	**32,99**	Q15293	**Reticulocalbin-1**	RCN1
0,000	-1,59	0,33	**33,25**	O75410	**Transforming acidic coiled-coil-containing protein 1**	TACC1
0,016	-1,59	0,33	**33,31**	P46199	**Translation initiation factor IF-2, mitochondrial**	MTIF2
0,040	-1,59	0,33	**33,33**	O15400	**Syntaxin-7**	STX7
0,020	-1,58	0,33	**33,37**	Q5TFE4	**5-nucleotidase domain-containing protein 1**	NT5DC1
0,009	-1,58	0,33	**33,43**	Q01518	**Adenylyl cyclase-associated protein 1**	CAP1
0,006	-1,58	0,33	**33,46**	Q9Y676	**28S ribosomal protein S18b, mitochondrial**	MRPS18B
0,019	-1,57	0,34	**33,58**	Q9GZU8	**Protein FAM192A**	FAM192A
0,039	-1,57	0,34	**33,63**	Q9BY89	**Uncharacterized protein KIAA1671**	KIAA1671
0,034	-1,57	0,34	**33,66**	Q9HCU4	**Cadherin EGF LAG seven-pass G-type receptor 2**	CELSR2
0,011	-1,57	0,34	**33,68**	P62834	**Ras-related protein Rap-1A**	RAP1A
0,001	-1,56	0,34	**33,80**	O14745	**Na(+)/H(+) exchange regulatory cofactor NHE-RF1**	SLC9A3R1
0,010	-1,56	0,34	**33,84**	Q9P2R7	**Succinyl-CoA ligase [ADP-forming] subunit beta, mitochondrial**	SUCLA2
0,005	-1,56	0,34	**33,92**	Q9BXJ9;Q6N069	**N-alpha-acetyltransferase 15, NatA auxiliary subunit**	NAA15
0,019	-1,56	0,34	**33,97**	Q6VY07	**Phosphofurin acidic cluster sorting protein 1**	PACS1
0,006	-1,56	0,34	**34,00**	O00264	**Membrane-associated progesterone receptor component 1**	PGRMC1
0,031	-1,56	0,34	**34,02**	P25705	**ATP synthase subunit alpha, mitochondrial**	ATP5A1
0,025	-1,55	0,34	**34,08**	Q12800	**Alpha-globin transcription factor CP2**	TFCP2
0,049	-1,55	0,34	**34,26**	Q2TAY7	**WD40 repeat-containing protein SMU1**	SMU1
0,000	-1,54	0,34	**34,33**	P08572	**Collagen alpha-2(IV) chain;Canstatin**	COL4A2
0,037	-1,54	0,34	**34,35**	O94906	**Pre-mRNA-processing factor 6**	PRPF6
0,000	-1,54	0,34	**34,45**	Q16270	**Insulin-like growth factor-binding protein 7**	IGFBP7
0,005	-1,54	0,34	**34,47**	Q9BVQ7	**Spermatogenesis-associated protein 5-like protein 1**	SPATA5L1
0,015	-1,54	0,34	**34,49**	Q13510	**Acid ceramidase;Acid ceramidase subunit alpha;Acid ceramidase subunit beta**	ASAH1
0,021	-1,53	0,35	**34,52**	O95865	**N(G),N(G)-dimethylarginine dimethylaminohydrolase 2**	DDAH2
0,044	-1,53	0,35	**34,59**	Q96M27	**Protein PRRC1**	PRRC1
0,017	-1,53	0,35	**34,64**	O95453	**Poly(A)-specific ribonuclease PARN**	PARN
0,001	-1,53	0,35	**34,71**	Q8NBJ7	**Sulfatase-modifying factor 2**	SUMF2
0,005	-1,53	0,35	**34,72**	Q9UNZ2	**NSFL1 cofactor p47**	NSFL1C
0,004	-1,53	0,35	**34,74**	P09972	**Fructose-bisphosphate aldolase C**	ALDOC

0,012	-1,52	0,35	**34,82**	Q14696	**LDLR chaperone MESD**	MESDC2
0,026	-1,52	0,35	**34,90**	P04083	**Annexin A1**	ANXA1
0,012	-1,52	0,35	**34,94**	P53990	**IST1 homolog**	IST1
0,034	-1,52	0,35	**34,97**	P36957	**Dihydrolipoyllysine-residue succinyltransferase component of 2-oxoglutarate dehyd**	DLST
0,021	-1,50	0,35	**35,33**	Q13442	**28 kDa heat- and acid-stable phosphoprotein**	PDAP1
0,029	-1,50	0,35	**35,44**	O60927	**Protein phosphatase 1 regulatory subunit 11**	PPP1R11
0,041	-1,50	0,35	**35,46**	Q16134	**Electron transfer flavoprotein-ubiquinone oxidoreductase, mitochondrial**	ETFDH
0,025	-1,49	0,36	**35,60**	Q92599	**Septin-8**	Sep.08
0,023	-1,49	0,36	**35,63**	P18206	**Vinculin**	VCL
0,009	-1,48	0,36	**35,80**	Q9Y6Y8	**SEC23-interacting protein**	SEC23IP
0,008	-1,48	0,36	**35,83**	Q8IXI1	**Mitochondrial Rho GTPase 2**	RHOT2
0,045	-1,47	0,36	**35,98**	O43414	**ERI1 exoribonuclease 3**	ERI3
0,035	-1,47	0,36	**36,06**	Q14108	**Lysosome membrane protein 2**	SCARB2
0,012	-1,47	0,36	**36,13**	P08758	**Annexin A5**	ANXA5
0,002	-1,47	0,36	**36,14**	Q96L92;O60759	**Sorting nexin-27**	SNX27
0,001	-1,46	0,36	**36,28**	Q5RI15	**Cytochrome c oxidase protein 20 homolog**	COX20
0,033	-1,45	0,37	**36,62**	P11413	**Glucose-6-phosphate 1-dehydrogenase**	G6PD
0,024	-1,45	0,37	**36,63**	Q96AQ6	**Pre-B-cell leukemia transcription factor-interacting protein 1**	PBXIP1
0,038	-1,44	0,37	**36,80**	Q9NRS6	**Sorting nexin-15**	SNX15
0,045	-1,44	0,37	**36,84**	Q96PZ0	**Pseudouridylate synthase 7 homolog**	PUS7
0,047	-1,44	0,37	**36,95**	Q9UJ70	**N-acetyl-D-glucosamine kinase**	NAGK
0,018	-1,43	0,37	**37,08**	Q32MZ4	**Leucine-rich repeat flightless-interacting protein 1**	LRRFIP1
0,033	-1,42	0,37	**37,26**	Q96EY7	**Pentatricopeptide repeat-containing protein 3, mitochondrial**	PTCD3
0,017	-1,42	0,37	**37,34**	Q9BZX2;Q9HA47	**Uridine-cytidine kinase 2;Uridine-cytidine kinase 1**	UCK2;UCK1
0,006	-1,42	0,37	**37,38**	P23588	**Eukaryotic translation initiation factor 4B**	EIF4B
0,035	-1,41	0,38	**37,53**	Q4G0F5	**Vacuolar protein sorting-associated protein 26B**	VPS26B
0,045	-1,41	0,38	**37,56**	Q9NQT4	**Exosome complex component RRP46**	EXOSC5
0,000	-1,41	0,38	**37,59**	P05067	**Amyloid beta A4 protein;N-APP;Soluble APP-alpha;Soluble APP-beta;C99;Beta-amyl**	APP
0,017	-1,41	0,38	**37,61**	P10644;P31321	**cAMP-dependent protein kinase type I-alpha regulatory subunit**	PRKAR1A
0,019	-1,41	0,38	**37,76**	P29353	**SHC-transforming protein 1**	SHC1
0,045	-1,40	0,38	**37,78**	P42126	**Enoyl-CoA delta isomerase 1, mitochondrial**	ECI1
0,015	-1,40	0,38	**37,85**	O60749	**Sorting nexin-2**	SNX2
0,034	-1,40	0,38	**37,91**	Q16864	**V-type proton ATPase subunit F**	ATP6V1F

0,002	-1,40	0,38	**37,98**	.Q2;Q5SNT6;Q9Y4E1;Q5	**WASH complex subunit FAM21A;WASH complex subunit FAM21B;WASH complex su**	FAM21A;FAM21B;
0,008	-1,40	0,38	**38,02**	O60307	**Microtubule-associated serine/threonine-protein kinase 3**	MAST3
0,011	-1,39	0,38	**38,08**	Q9Y496;Q9P2E2	**Kinesin-like protein KIF3A**	KIF3A
0,017	-1,39	0,38	**38,15**	O95573	**Long-chain-fatty-acid--CoA ligase 3**	ACSL3
0,021	-1,39	0,38	**38,15**	P49588	**Alanine--tRNA ligase, cytoplasmic**	AARS
0,002	-1,39	0,38	**38,25**	P28331	**NADH-ubiquinone oxidoreductase 75 kDa subunit, mitochondrial**	NDUFS1
0,050	-1,38	0,38	**38,45**	Q9NVE7	**Pantothenate kinase 4**	PANK4
0,015	-1,38	0,38	**38,47**	P13667	**Protein disulfide-isomerase A4**	PDIA4
0,001	-1,37	0,39	**38,64**	P22830	**Ferrochelatase, mitochondrial**	FECH
0,001	-1,37	0,39	**38,79**	Q9H6E4	**Coiled-coil domain-containing protein 134**	CCDC134
0,033	-1,36	0,39	**38,84**	Q6DKJ4	**Nucleoredoxin**	NXN
0,013	-1,36	0,39	**38,92**	Q9NVD7;Q9HBI1	**Alpha-parvin**	PARVA
0,025	-1,36	0,39	**39,08**	Q06710	**Paired box protein Pax-8**	PAX8
0,017	-1,35	0,39	**39,10**	P40306	**Proteasome subunit beta type-10**	PSMB10
0,019	-1,35	0,39	**39,17**	Q9HB07	**UPF0160 protein MYG1, mitochondrial**	C12orf10
0,042	-1,35	0,39	**39,17**	Q02127	**Dihydroorotate dehydrogenase (quinone), mitochondrial**	DHODH
0,046	-1,35	0,39	**39,23**	Q96AT9;Q2QD12	**Ribulose-phosphate 3-epimerase**	RPE
0,028	-1,35	0,39	**39,32**	Q9BVG4	**UPF0368 protein Cxorf26**	CXorf26
0,046	-1,34	0,39	**39,43**	P52926	**High mobility group protein HMGI-C**	HMGA2
0,023	-1,34	0,40	**39,51**	Q56VL3	**OCIA domain-containing protein 2**	OCIAD2
0,031	-1,33	0,40	**39,64**	P0C0S5;Q71UI9;Q8IUE6	**Histone H2A.Z;Histone H2A.V**	H2AFZ;H2AFV
0,002	-1,33	0,40	**39,68**	Q96KG9	**N-terminal kinase-like protein**	SCYL1
0,001	-1,33	0,40	**39,72**	P62633	**Cellular nucleic acid-binding protein**	CNBP
0,045	-1,33	0,40	**39,75**	Q05209	**Tyrosine-protein phosphatase non-receptor type 12**	PTPN12
0,048	-1,33	0,40	**39,78**	P14314	**Glucosidase 2 subunit beta**	PRKCSH
0,034	-1,33	0,40	**39,89**	Q9BRF8	**Calcineurin-like phosphoesterase domain-containing protein 1**	CPPED1
0,003	-1,32	0,40	**39,97**	Q99615	**DnaJ homolog subfamily C member 7**	DNAJC7
0,001	-1,32	0,40	**40,01**	O75534	**Cold shock domain-containing protein E1**	CSDE1
0,008	-1,32	0,40	**40,14**	P12429	**Annexin A3**	ANXA3
0,037	-1,31	0,40	**40,34**	Q8IXQ4	**Uncharacterized protein KIAA1704**	KIAA1704
0,000	-1,31	0,40	**40,43**	Q92896	**Golgi apparatus protein 1**	GLG1
0,000	-1,30	0,41	**40,56**	O75569	**Interferon-inducible double stranded RNA-dependent protein kinase activator A**	PRKRA
0,002	-1,30	0,41	**40,57**	P49407	**Beta-arrestin-1**	ARRB1

0,002	-1,30	0,41	**40,57**	P85037	**Forkhead box protein K1**	FOXK1
0,033	-1,30	0,41	**40,62**	Q9NYL2	**Mitogen-activated protein kinase kinase kinase MLT**	MLTK
0,000	-1,30	0,41	**40,70**	P62877	**E3 ubiquitin-protein ligase RBX1**	RBX1
0,004	-1,30	0,41	**40,74**	Q9Y617	**Phosphoserine aminotransferase**	PSAT1
0,000	-1,30	0,41	**40,75**	P18754	**Regulator of chromosome condensation**	RCC1
0,000	-1,30	0,41	**40,75**	Q13740	**CD166 antigen**	ALCAM
0,029	-1,29	0,41	**40,86**	Q86WQ0	**Nuclear receptor 2C2-associated protein**	NR2C2AP
0,049	-1,29	0,41	**40,87**	Q9BYD3	**39S ribosomal protein L4, mitochondrial**	MRPL4
0,000	-1,29	0,41	**40,94**	P52565	**Rho GDP-dissociation inhibitor 1**	ARHGDIA
0,029	-1,29	0,41	**40,94**	Q96RE7	**Nucleus accumbens-associated protein 1**	NACC1
0,002	-1,28	0,41	**41,22**	P12081	**Histidine--tRNA ligase, cytoplasmic**	HARS
0,017	-1,28	0,41	**41,23**	Q9Y2H0	**Disks large-associated protein 4**	DLGAP4
0,032	-1,28	0,41	**41,29**	P21964	**Catechol O-methyltransferase**	COMT
0,000	-1,27	0,41	**41,46**	Q8NBJ5	**Procollagen galactosyltransferase 1**	GLT25D1
0,009	-1,26	0,42	**41,72**	P20073	**Annexin A7**	ANXA7
0,016	-1,26	0,42	**41,74**	Q96EK6	**Glucosamine 6-phosphate N-acetyltransferase**	GNPNAT1
0,035	-1,26	0,42	**41,77**	P14635	**G2/mitotic-specific cyclin-B1**	CCNB1
0,020	-1,26	0,42	**41,90**	P21283	**V-type proton ATPase subunit C 1**	ATP6V1C1
0,009	-1,25	0,42	**41,91**	Q13753	**Laminin subunit gamma-2**	LAMC2
0,015	-1,25	0,42	**41,96**	P05455	**Lupus La protein**	SSB
0,006	-1,25	0,42	**42,00**	Q9Y291	**28S ribosomal protein S33, mitochondrial**	MRPS33
0,000	-1,25	0,42	**42,05**	Q9Y3A5	**Ribosome maturation protein SBDS**	SBDS
0,003	-1,25	0,42	**42,14**	P24752	**Acetyl-CoA acetyltransferase, mitochondrial**	ACAT1
0,019	-1,25	0,42	**42,15**	O60925	**Prefoldin subunit 1**	PFDN1
0,004	-1,24	0,42	**42,21**	P18827	**Syndecan-1**	SDC1
0,024	-1,24	0,42	**42,23**	P50502;Q8NFI4;Q8IZP2	**Hsc70-interacting protein;Putative protein FAM10A5;Putative protein FAM10A4**	ST13;ST13P5;ST13
0,011	-1,24	0,42	**42,32**	Q9NZR1	**Tropomodulin-2**	TMOD2
0,019	-1,24	0,42	**42,41**	Q99436	**Proteasome subunit beta type-7**	PSMB7
0,039	-1,23	0,43	**42,64**	O43865	**Putative adenosylhomocysteinase 2**	AHCYL1
0,031	-1,23	0,43	**42,64**	Q9HB21	**Pleckstrin homology domain-containing family A member 1**	PLEKHA1
0,034	-1,23	0,43	**42,74**	Q13951	**Core-binding factor subunit beta**	CBFB
0,030	-1,22	0,43	**42,83**	Q86VW0	**SEC14 domain and spectrin repeat-containing protein 1**	SESTD1
0,010	-1,22	0,43	**42,94**	P06396	**Gelsolin**	GSN

0,000	-1,21	0,43	**43,09**	P11717	**Cation-independent mannose-6-phosphate receptor**	IGF2R
0,003	-1,21	0,43	**43,10**	P26639	**Threonine--tRNA ligase, cytoplasmic**	TARS
0,029	-1,21	0,43	**43,10**	O75794	**Cell division cycle protein 123 homolog**	CDC123
0,020	-1,21	0,43	**43,14**	O75616	**GTPase Era, mitochondrial**	ERAL1
0,007	-1,21	0,43	**43,15**	P18859	**ATP synthase-coupling factor 6, mitochondrial**	ATP5J
0,000	-1,21	0,43	**43,18**	Q9Y3C8	**Ubiquitin-fold modifier-conjugating enzyme 1**	UFC1
0,004	-1,20	0,43	**43,41**	Q9UM11	**Fizzy-related protein homolog**	FZR1
0,004	-1,20	0,44	**43,60**	P28482	**Mitogen-activated protein kinase 1**	MAPK1
0,000	-1,20	0,44	**43,64**	Q13501	**Sequestosome-1**	SQSTM1
0,010	-1,19	0,44	**43,68**	Q99536	**Synaptic vesicle membrane protein VAT-1 homolog**	VAT1
0,003	-1,19	0,44	**43,68**	P19404	**NADH dehydrogenase [ubiquinone] flavoprotein 2, mitochondrial**	NDUFV2
0,027	-1,19	0,44	**43,70**	Q15758	**Neutral amino acid transporter B(0)**	SLC1A5
0,006	-1,19	0,44	**43,72**	Q9Y2W1	**Thyroid hormone receptor-associated protein 3**	THRAP3
0,018	-1,19	0,44	**43,83**	Q14137	**Ribosome biogenesis protein BOP1**	BOP1
0,017	-1,19	0,44	**43,86**	Q12765	**Secernin-1**	SCRN1
0,007	-1,18	0,44	**44,20**	P11234	**Ras-related protein Ral-B**	RALB
0,003	-1,17	0,44	**44,29**	P55010	**Eukaryotic translation initiation factor 5**	EIF5
0,012	-1,17	0,45	**44,52**	P35080	**Profilin-2**	PFN2
0,038	-1,17	0,45	**44,52**	Q01995	**Transgelin**	TAGLN
0,044	-1,17	0,45	**44,54**	Q8WWK9	**Cytoskeleton-associated protein 2**	CKAP2
0,001	-1,17	0,45	**44,59**	P10155	**60 kDa SS-A/Ro ribonucleoprotein**	TROVE2
0,000	-1,16	0,45	**44,65**	O75822	**Eukaryotic translation initiation factor 3 subunit J**	EIF3J
0,002	-1,16	0,45	**44,68**	Q9Y5J7	**Mitochondrial import inner membrane translocase subunit Tim9**	TIMM9
0,001	-1,16	0,45	**44,69**	O43169	**Cytochrome b5 type B**	CYB5B
0,002	-1,16	0,45	**44,70**	P67809	**Nuclease-sensitive element-binding protein 1**	YBX1
0,030	-1,16	0,45	**44,75**	O75448	**Mediator of RNA polymerase II transcription subunit 24**	MED24
0,035	-1,16	0,45	**44,88**	P32119	**Peroxiredoxin-2**	PRDX2
0,023	-1,15	0,45	**44,99**	P07195	**L-lactate dehydrogenase B chain**	LDHB
0,015	-1,15	0,45	**45,00**	Q8NFH3	**Nucleoporin Nup43**	NUP43
0,005	-1,15	0,45	**45,07**	Q04917	**14-3-3 protein eta**	YWHAH
0,013	-1,15	0,45	**45,17**	Q8N1G4	**Leucine-rich repeat-containing protein 47**	LRRC47
0,010	-1,14	0,45	**45,35**	P27797	**Calreticulin**	CALR
0,038	-1,14	0,45	**45,37**	A2A3N6;Q99755	**Putative PIP5K1A and PSMD4-like protein;Phosphatidylinositol 4-phosphate 5-kinas**	PIPSL;PIP5K1A

0,011	-1,14	0,45	**45,42**	P10606	**Cytochrome c oxidase subunit 5B, mitochondrial**	COX5B
0,006	-1,14	0,46	**45,53**	P61758	**Prefoldin subunit 3**	VBP1
0,006	-1,13	0,46	**45,55**	Q9BRP8	**Partner of Y14 and mago**	WIBG
0,040	-1,13	0,46	**45,58**	Q12972	**Nuclear inhibitor of protein phosphatase 1;Activator of RNA decay**	PPP1R8
0,001	-1,13	0,46	**45,71**	O43583	**Density-regulated protein**	DENR
0,050	-1,13	0,46	**45,83**	Q96FN4	**Copine-2**	CPNE2
0,000	-1,12	0,46	**45,94**	Q9H078	**Caseinolytic peptidase B protein homolog**	CLPB
0,045	-1,12	0,46	**45,99**	P27361	**Mitogen-activated protein kinase 3**	MAPK3
0,008	-1,12	0,46	**46,02**	Q9UPP1	**Histone lysine demethylase PHF8**	PHF8
0,007	-1,12	0,46	**46,10**	P53992	**Protein transport protein Sec24C**	SEC24C
0,032	-1,12	0,46	**46,11**	P30101	**Protein disulfide-isomerase A3**	PDIA3
0,031	-1,12	0,46	**46,15**	P52209	**6-phosphogluconate dehydrogenase, decarboxylating**	PGD
0,000	-1,11	0,46	**46,20**	O75347	**Tubulin-specific chaperone A**	TBCA
0,038	-1,11	0,46	**46,29**	Q9UJA5	**tRNA (adenine(58)-N(1))-methyltransferase non-catalytic subunit TRM6**	TRMT6
0,016	-1,10	0,47	**46,52**	O60568	**Procollagen-lysine,2-oxoglutarate 5-dioxygenase 3**	PLOD3
0,004	-1,10	0,47	**46,54**	P22695	**Cytochrome b-c1 complex subunit 2, mitochondrial**	UQCRC2
0,011	-1,10	0,47	**46,55**	Q9GZT9	**Egl nine homolog 1**	EGLN1
0,009	-1,10	0,47	**46,61**	Q9BZI7	**Regulator of nonsense transcripts 3B**	UPF3B
0,021	-1,10	0,47	**46,64**	Q9C0E2	**Exportin-4**	XPO4
0,027	-1,10	0,47	**46,76**	P61088	**Ubiquitin-conjugating enzyme E2 N**	UBE2N
0,004	-1,09	0,47	**47,13**	Q08J23	**tRNA (cytosine(34)-C(5))-methyltransferase**	NSUN2
0,046	-1,09	0,47	**47,13**	P37837	**Transaldolase**	TALDO1
0,039	-1,09	0,47	**47,14**	Q8NBS9	**Thioredoxin domain-containing protein 5**	TXNDC5
0,010	-1,08	0,47	**47,28**	Q9H6S3	**Epidermal growth factor receptor kinase substrate 8-like protein 2**	EPS8L2
0,005	-1,08	0,47	**47,29**	P23526	**Adenosylhomocysteinase**	AHCY
0,034	-1,08	0,47	**47,34**	Q9BRT2	**Mitochondrial nucleoid factor 1**	MNF1
0,021	-1,08	0,47	**47,36**	P61604	**10 kDa heat shock protein, mitochondrial**	HSPE1
0,014	-1,08	0,47	**47,37**	Q96AC1	**Fermitin family homolog 2**	FERMT2
0,009	-1,08	0,47	**47,39**	P34932	**Heat shock 70 kDa protein 4**	HSPA4
0,003	-1,07	0,48	**47,53**	Q7Z4V5	**Hepatoma-derived growth factor-related protein 2**	HDGFRP2
0,003	-1,07	0,48	**47,54**	O95292	**Vesicle-associated membrane protein-associated protein B/C**	VAPB
0,009	-1,07	0,48	**47,68**	P49821	**NADH dehydrogenase [ubiquinone] flavoprotein 1, mitochondrial**	NDUFV1
0,005	-1,07	0,48	**47,78**	Q9Y3F4	**Serine-threonine kinase receptor-associated protein**	STRAP

0,018	-1,06	0,48	**47,94**	Q06587	**E3 ubiquitin-protein ligase RING1**	RING1
0,047	-1,06	0,48	**47,96**	Q5F1R6	**DnaJ homolog subfamily C member 21**	DNAJC21
0,019	-1,06	0,48	**48,02**	P41227;Q9BSU3	**N-alpha-acetyltransferase 10**	NAA10
0,001	-1,06	0,48	**48,04**	P07858	**Cathepsin B;Cathepsin B light chain;Cathepsin B heavy chain**	CTSB
0,003	-1,06	0,48	**48,13**	P50895	**Basal cell adhesion molecule**	BCAM
0,031	-1,05	0,48	**48,18**	Q6NZ67;Q6P582	**Mitotic-spindle organizing protein 2B;Mitotic-spindle organizing protein 2A**	MZT2B;MZT2A
0,032	-1,05	0,48	**48,23**	Q9P015	**39S ribosomal protein L15, mitochondrial**	MRPL15
0,000	-1,04	0,48	**48,47**	Q8TAT6	**Nuclear protein localization protein 4 homolog**	NPLOC4
0,002	-1,04	0,48	**48,48**	P60520	**Gamma-aminobutyric acid receptor-associated protein-like 2**	GABARAPL2
0,016	-1,04	0,49	**48,52**	Q9BRX2	**Protein pelota homolog**	PELO
0,000	-1,04	0,49	**48,54**	Q14677	**Clathrin interactor 1**	CLINT1
0,000	-1,04	0,49	**48,63**	O60524	**Nuclear export mediator factor NEMF**	NEMF
0,046	-1,04	0,49	**48,69**	Q9UL46	**Proteasome activator complex subunit 2**	PSME2
0,003	-1,04	0,49	**48,73**	P30519	**Heme oxygenase 2**	HMOX2
0,000	-1,03	0,49	**48,92**	P18669;Q8N0Y7;P1525[illegible]	**Phosphoglycerate mutase 1**	PGAM1
0,012	-1,03	0,49	**48,95**	[illegible]26;Q15303;P80192;Q5[illegible]	**Receptor tyrosine-protein kinase erbB-2**	ERBB2
0,004	-1,03	0,49	**49,09**	P23193;Q15560	**Transcription elongation factor A protein 1**	TCEA1
0,002	-1,03	0,49	**49,11**	P55072	**Transitional endoplasmic reticulum ATPase**	VCP
0,001	-1,02	0,49	**49,15**	P61201	**COP9 signalosome complex subunit 2**	COPS2
0,020	-1,02	0,49	**49,16**	Q9Y3D9	**28S ribosomal protein S23, mitochondrial**	MRPS23
0,047	-1,02	0,49	**49,18**	Q9BYD1	**39S ribosomal protein L13, mitochondrial**	MRPL13
0,032	-1,02	0,49	**49,21**	P22314	**Ubiquitin-like modifier-activating enzyme 1**	UBA1
0,007	-1,02	0,49	**49,23**	P61221	**ATP-binding cassette sub-family E member 1**	ABCE1
0,003	-1,02	0,49	**49,23**	Q92598	**Heat shock protein 105 kDa**	HSPH1
0,001	-1,02	0,49	**49,24**	Q9Y5S9	**RNA-binding protein 8A**	RBM8A
0,041	-1,02	0,49	**49,27**	Q15907;P62491	**Ras-related protein Rab-11B;Ras-related protein Rab-11A**	RAB11B;RAB11A
0,013	-1,02	0,49	**49,33**	Q96AE4	**Far upstream element-binding protein 1**	FUBP1
0,004	-1,02	0,49	**49,42**	Q15942	**Zyxin**	ZYX
0,018	-1,01	0,49	**49,49**	P27695	**DNA-(apurinic or apyrimidinic site) lyase;DNA-(apurinic or apyrimidinic site) lyase, n[illegible]**	APEX1
0,024	-1,01	0,50	**49,52**	Q13769	**THO complex subunit 5 homolog**	THOC5
0,035	-1,01	0,50	**49,54**	P07900;Q14568;Q58FG[illegible]	**Heat shock protein HSP 90-alpha**	HSP90AA1
0,030	-1,01	0,50	**49,57**	O43676	**NADH dehydrogenase [ubiquinone] 1 beta subcomplex subunit 3**	NDUFB3
0,005	-1,01	0,50	**49,64**	Q8N3E9	**1-phosphatidylinositol 4,5-bisphosphate phosphodiesterase delta-3**	PLCD3

0,012	-1,01	0,50	**49,73**	Q9UFC0	**Leucine-rich repeat and WD repeat-containing protein 1**	LRWD1
0,041	-1,01	0,50	**49,78**	P08238;Q58FF7	**Heat shock protein HSP 90-beta;Putative heat shock protein HSP 90-beta-3**	HSP90AB1;HSP90A
0,031	-1,00	0,50	**49,93**	Q96JP5	**E3 ubiquitin-protein ligase ZFP91**	ZFP91
0,032	-1,00	0,50	**49,95**	P09493	**Tropomyosin alpha-1 chain**	TPM1
0,022	-1,00	0,50	**49,96**	Q06203	**Amidophosphoribosyltransferase**	PPAT
0,023	-1,00	0,50	**49,99**	Q9UL15	**BAG family molecular chaperone regulator 5**	BAG5
0,008	-1,00	0,50	**50,04**	Q9UI30	**tRNA methyltransferase 112 homolog**	TRMT112
0,047	-1,00	0,50	**50,12**	P25490	**Transcriptional repressor protein YY1**	YY1
0,009	-0,99	0,50	**50,19**	P46937	**Yorkie homolog**	YAP1
0,040	-0,99	0,50	**50,19**	P48163	**NADP-dependent malic enzyme**	ME1
0,017	-0,99	0,51	**50,51**	Q9UQ80	**Proliferation-associated protein 2G4**	PA2G4
0,002	-0,98	0,51	**50,55**	P18031	**Tyrosine-protein phosphatase non-receptor type 1**	PTPN1
0,022	-0,98	0,51	**50,59**	Q9BS26	**Endoplasmic reticulum resident protein 44**	ERP44
0,013	-0,98	0,51	**50,75**	Q01970	**1-phosphatidylinositol 4,5-bisphosphate phosphodiesterase beta-3**	PLCB3
0,021	-0,98	0,51	**50,82**	P11277	**Spectrin beta chain, erythrocyte**	SPTB
0,013	-0,97	0,51	**50,98**	P20810	**Calpastatin**	CAST
0,019	-0,97	0,51	**51,00**	Q15185	**Prostaglandin E synthase 3**	PTGES3
0,026	-0,97	0,51	**51,05**	O95340	**Bifunctional 3-phosphoadenosine 5-phosphosulfate synthase 2;Sulfate adenylyltran**	PAPSS2
0,027	-0,97	0,51	**51,05**	P54577	**Tyrosine--tRNA ligase, cytoplasmic**	YARS
0,003	-0,97	0,51	**51,07**	O14818;Q8TAA3	**Proteasome subunit alpha type-7**	PSMA7
0,023	-0,97	0,51	**51,10**	P22102	**Trifunctional purine biosynthetic protein adenosine-3;Phosphoribosylamine--glycine**	GART
0,017	-0,97	0,51	**51,16**	Q99873;Q9NR22	**Protein arginine N-methyltransferase 1**	PRMT1
0,000	-0,96	0,51	**51,26**	Q07020	**60S ribosomal protein L18**	RPL18
0,000	-0,96	0,51	**51,31**	P48426	**Phosphatidylinositol 5-phosphate 4-kinase type-2 alpha**	PIP4K2A
0,006	-0,96	0,51	**51,33**	Q01105;P0DME0	**Protein SET**	SET
0,002	-0,96	0,51	**51,35**	Q12986	**Transcriptional repressor NF-X1**	NFX1
0,000	-0,96	0,51	**51,42**	O15230	**Laminin subunit alpha-5**	LAMA5
0,031	-0,96	0,52	**51,53**	Q9H019	**Protein FAM54B**	FAM54B
0,008	-0,95	0,52	**51,64**	Q02809	**Procollagen-lysine,2-oxoglutarate 5-dioxygenase 1**	PLOD1
0,037	-0,95	0,52	**51,67**	Q8IYA6	**Cytoskeleton-associated protein 2-like**	CKAP2L
0,039	-0,95	0,52	**51,92**	P18124	**60S ribosomal protein L7**	RPL7
0,005	-0,94	0,52	**52,04**	P16403	**Histone H1.2**	HIST1H1C
0,004	-0,94	0,52	**52,06**	O75312	**Zinc finger protein ZPR1**	ZNF259

0,036	-0,94	0,52	**52,09**	P42566	**Epidermal growth factor receptor substrate 15**	EPS15
0,009	-0,94	0,52	**52,14**	Q9ULX3	**RNA-binding protein NOB1**	NOB1
0,039	-0,94	0,52	**52,18**	P09525	**Annexin A4**	ANXA4
0,048	-0,94	0,52	**52,20**	P00450	**Ceruloplasmin**	CP
0,024	-0,93	0,52	**52,33**	Q9UHV9	**Prefoldin subunit 2**	PFDN2
0,002	-0,93	0,52	**52,37**	P13611	**Versican core protein**	VCAN
0,033	-0,93	0,52	**52,41**	O14653	**Golgi SNAP receptor complex member 2**	GOSR2
0,021	-0,93	0,52	**52,45**	P23381	**Tryptophan--tRNA ligase, cytoplasmic;T1-TrpRS;T2-TrpRS**	WARS
0,030	-0,93	0,53	**52,59**	Q9C035	**Tripartite motif-containing protein 5**	TRIM5
0,036	-0,93	0,53	**52,59**	Q15545	**Transcription initiation factor TFIID subunit 7**	TAF7
0,021	-0,93	0,53	**52,63**	O43447	**Peptidyl-prolyl cis-trans isomerase H**	PPIH
0,031	-0,93	0,53	**52,63**	Q9NZ09	**Ubiquitin-associated protein 1**	UBAP1
0,002	-0,92	0,53	**52,73**	Q13185	**Chromobox protein homolog 3**	CBX3
0,003	-0,92	0,53	**52,88**	P60866	**40S ribosomal protein S20**	RPS20
0,029	-0,92	0,53	**52,91**	Q12931	**Heat shock protein 75 kDa, mitochondrial**	TRAP1
0,031	-0,91	0,53	**53,06**	P29762	**Cellular retinoic acid-binding protein 1**	CRABP1
0,016	-0,91	0,53	**53,08**	O43148	**mRNA cap guanine-N7 methyltransferase**	RNMT
0,027	-0,91	0,53	**53,33**	Q9H2U2	**Inorganic pyrophosphatase 2, mitochondrial**	PPA2
0,013	-0,91	0,53	**53,35**	P15586	**N-acetylglucosamine-6-sulfatase**	GNS
0,007	-0,90	0,54	**53,62**	P62263	**40S ribosomal protein S14**	RPS14
0,030	-0,89	0,54	**53,97**	P20618	**Proteasome subunit beta type-1**	PSMB1
0,003	-0,89	0,54	**54,04**	P52943	**Cysteine-rich protein 2**	CRIP2
0,001	-0,88	0,54	**54,16**	Q9H0B6	**Kinesin light chain 2**	KLC2
0,022	-0,88	0,54	**54,19**	P26885	**Peptidyl-prolyl cis-trans isomerase FKBP2**	FKBP2
0,012	-0,88	0,54	**54,22**	P13693;Q56UQ5	**Translationally-controlled tumor protein**	TPT1
0,003	-0,88	0,54	**54,24**	P40222	**Alpha-taxilin**	TXLNA
0,009	-0,88	0,54	**54,31**	Q9UNF0	**Protein kinase C and casein kinase substrate in neurons protein 2**	PACSIN2
0,004	-0,88	0,54	**54,40**	Q9H7E9	**UPF0488 protein C8orf33**	C8orf33
0,044	-0,88	0,54	**54,40**	P35222	**Catenin beta-1**	CTNNB1
0,004	-0,88	0,54	**54,40**	P23229	**Integrin alpha-6;Integrin alpha-6 heavy chain;Integrin alpha-6 light chain**	ITGA6
0,029	-0,88	0,54	**54,41**	Q9H910	**Hematological and neurological expressed 1-like protein**	HN1L
0,013	-0,88	0,54	**54,43**	O15212	**Prefoldin subunit 6**	PFDN6
0,042	-0,88	0,54	**54,44**	Q13217	**DnaJ homolog subfamily C member 3**	DNAJC3

0,008	-0,88	0,55	**54,52**	P41567;O60739	**Eukaryotic translation initiation factor 1;Eukaryotic translation initiation factor 1b**	EIF1;EIF1B
0,003	-0,87	0,55	**54,56**	P35613	**Basigin**	BSG
0,033	-0,87	0,55	**54,61**	Q9BWU0	**Kanadaptin**	SLC4A1AP
0,048	-0,87	0,55	**54,76**	O43278	**Kunitz-type protease inhibitor 1**	SPINT1
0,047	-0,87	0,55	**54,78**	P28065	**Proteasome subunit beta type-9**	PSMB9
0,002	-0,87	0,55	**54,84**	P07741	**Adenine phosphoribosyltransferase**	APRT
0,000	-0,87	0,55	**54,85**	Q9H8Y8	**Golgi reassembly-stacking protein 2**	GORASP2
0,046	-0,87	0,55	**54,88**	P61289	**Proteasome activator complex subunit 3**	PSME3
0,013	-0,86	0,55	**54,95**	Q9UBS4	**DnaJ homolog subfamily B member 11**	DNAJB11
0,008	-0,86	0,55	**55,02**	P06454	**Prothymosin alpha;Thymosin alpha-1**	PTMA
0,047	-0,86	0,55	**55,09**	O75781	**Paralemmin-1**	PALM
0,043	-0,86	0,55	**55,11**	Q9Y490	**Talin-1**	TLN1
0,037	-0,86	0,55	**55,16**	Q92905	**COP9 signalosome complex subunit 5**	COPS5
0,048	-0,85	0,55	**55,31**	Q8TDM6	**Disks large homolog 5**	DLG5
0,027	-0,85	0,55	**55,32**	Q71F56	**Mediator of RNA polymerase II transcription subunit 13-like**	MED13L
0,006	-0,85	0,55	**55,34**	P07602	**Proactivator polypeptide;Saposin-A;Saposin-B-Val;Saposin-B;Saposin-C;Saposin-D**	PSAP
0,000	-0,85	0,55	**55,42**	P52815	**39S ribosomal protein L12, mitochondrial**	MRPL12
0,008	-0,85	0,55	**55,43**	P17987	**T-complex protein 1 subunit alpha**	TCP1
0,045	-0,85	0,56	**55,52**	P80723	**Brain acid soluble protein 1**	BASP1
0,036	-0,85	0,56	**55,56**	Q9BXV9	**Uncharacterized protein C14orf142**	C14orf142
0,010	-0,85	0,56	**55,61**	Q9BV57	**1,2-dihydroxy-3-keto-5-methylthiopentene dioxygenase**	ADI1
0,030	-0,84	0,56	**55,74**	P15144	**Aminopeptidase N**	ANPEP
0,013	-0,84	0,56	**55,85**	P28062	**Proteasome subunit beta type-8**	PSMB8
0,028	-0,84	0,56	**55,95**	E9PAV3;Q13765	**Nascent polypeptide-associated complex subunit alpha**	NACA
0,008	-0,83	0,56	**56,14**	P82912	**28S ribosomal protein S11, mitochondrial**	MRPS11
0,002	-0,83	0,56	**56,16**	P62424	**60S ribosomal protein L7a**	RPL7A
0,019	-0,83	0,56	**56,42**	Q9Y237	**Peptidyl-prolyl cis-trans isomerase NIMA-interacting 4**	PIN4
0,011	-0,82	0,56	**56,46**	P31930	**Cytochrome b-c1 complex subunit 1, mitochondrial**	UQCRC1
0,019	-0,82	0,57	**56,50**	P00813	**Adenosine deaminase**	ADA
0,018	-0,82	0,57	**56,56**	Q9H0C8	**Integrin-linked kinase-associated serine/threonine phosphatase 2C**	ILKAP
0,029	-0,82	0,57	**56,64**	Q14318	**Peptidyl-prolyl cis-trans isomerase FKBP8**	FKBP8
0,012	-0,82	0,57	**56,65**	Q9NRY4	**Rho GTPase-activating protein 35**	ARHGAP35
0,003	-0,82	0,57	**56,67**	Q99714	**3-hydroxyacyl-CoA dehydrogenase type-2**	HSD17B10

0,011	-0,81	0,57	**56,85**	P26358	**DNA (cytosine-5)-methyltransferase 1**	DNMT1
0,009	-0,81	0,57	**56,86**	P14406	**Cytochrome c oxidase subunit 7A2, mitochondrial**	COX7A2
0,004	-0,81	0,57	**56,89**	Q7Z2T5	**TRMT1-like protein**	TRMT1L
0,003	-0,81	0,57	**56,89**	Q9UKY7	**Protein CDV3 homolog**	CDV3
0,001	-0,81	0,57	**56,92**	Q92665	**28S ribosomal protein S31, mitochondrial**	MRPS31
0,018	-0,81	0,57	**57,00**	P13674	**Prolyl 4-hydroxylase subunit alpha-1**	P4HA1
0,029	-0,81	0,57	**57,12**	P40227	**T-complex protein 1 subunit zeta**	CCT6A
0,027	-0,81	0,57	**57,17**	P42766	**60S ribosomal protein L35**	RPL35
0,003	-0,80	0,57	**57,33**	O43402	**Neighbor of COX4**	COX4NB
0,038	-0,80	0,57	**57,37**	P13639	**Elongation factor 2**	EEF2
0,021	-0,80	0,58	**57,63**	P10599	**Thioredoxin**	TXN
0,044	-0,79	0,58	**57,64**	Q99497	**Protein DJ-1**	PARK7
0,019	-0,79	0,58	**57,67**	Q9UK41	**Vacuolar protein sorting-associated protein 28 homolog**	VPS28
0,001	-0,79	0,58	**57,72**	P49755	**Transmembrane emp24 domain-containing protein 10**	TMED10
0,005	-0,79	0,58	**57,80**	O43709	**Uncharacterized methyltransferase WBSCR22**	WBSCR22
0,039	-0,79	0,58	**57,81**	P10768	**S-formylglutathione hydrolase**	ESD
0,003	-0,79	0,58	**57,89**	P62750	**60S ribosomal protein L23a**	RPL23A
0,008	-0,79	0,58	**57,94**	P63208	**S-phase kinase-associated protein 1**	SKP1
0,001	-0,78	0,58	**58,21**	P60842	**Eukaryotic initiation factor 4A-I**	EIF4A1
0,000	-0,78	0,58	**58,23**	P30050	**60S ribosomal protein L12**	RPL12
0,032	-0,78	0,58	**58,30**	P51665	**26S proteasome non-ATPase regulatory subunit 7**	PSMD7
0,040	-0,78	0,58	**58,37**	P30041	**Peroxiredoxin-6**	PRDX6
0,045	-0,78	0,58	**58,40**	P27348	**14-3-3 protein theta**	YWHAQ
0,025	-0,77	0,58	**58,45**	Q15075	**Early endosome antigen 1**	EEA1
0,022	-0,77	0,58	**58,47**	Q9BXS6	**Nucleolar and spindle-associated protein 1**	NUSAP1
0,012	-0,77	0,58	**58,49**	Q9H4M9	**EH domain-containing protein 1**	EHD1
0,038	-0,77	0,59	**58,61**	Q15006	**Tetratricopeptide repeat protein 35**	TTC35
0,000	-0,77	0,59	**58,61**	O00468	**Agrin**	AGRN
0,040	-0,77	0,59	**58,65**	Q96QK1	**Vacuolar protein sorting-associated protein 35**	VPS35
0,002	-0,77	0,59	**58,69**	Q9UBK7;Q9UNT1	**Rab-like protein 2A;Rab-like protein 2B**	RABL2A;RABL2B
0,009	-0,77	0,59	**58,72**	P25789	**Proteasome subunit alpha type-4**	PSMA4
0,003	-0,77	0,59	**58,84**	Q02878	**60S ribosomal protein L6**	RPL6
0,001	-0,76	0,59	**59,01**	Q04837	**Single-stranded DNA-binding protein, mitochondrial**	SSBP1

0,006	-0,76	0,59	**59,14**	P61254;Q9UNX3	**60S ribosomal protein L26;60S ribosomal protein L26-like 1**	RPL26;RPL26L1
0,015	-0,76	0,59	**59,23**	P51649	**Succinate-semialdehyde dehydrogenase, mitochondrial**	ALDH5A1
0,036	-0,76	0,59	**59,25**	O95630	**STAM-binding protein**	STAMBP
0,036	-0,75	0,59	**59,34**	P46940	**Ras GTPase-activating-like protein IQGAP1**	IQGAP1
0,014	-0,75	0,59	**59,38**	Q96HC4	**PDZ and LIM domain protein 5**	PDLIM5
0,005	-0,75	0,59	**59,46**	Q6PKG0;Q659C4	**La-related protein 1**	LARP1
0,009	-0,75	0,59	**59,47**	P54819	**Adenylate kinase 2, mitochondrial**	AK2
0,006	-0,75	0,60	**59,53**	P00441	**Superoxide dismutase [Cu-Zn]**	SOD1
0,005	-0,75	0,60	**59,54**	Q5HYJ3	**Protein FAM76B**	FAM76B
0,000	-0,75	0,60	**59,58**	Q14240	**Eukaryotic initiation factor 4A-II**	EIF4A2
0,036	-0,75	0,60	**59,64**	P50990	**T-complex protein 1 subunit theta**	CCT8
0,011	-0,75	0,60	**59,65**	P51858	**Hepatoma-derived growth factor**	HDGF
0,014	-0,74	0,60	**59,84**	Q9GZN8	**UPF0687 protein C20orf27**	C20orf27
0,004	-0,74	0,60	**59,96**	O00170	**AH receptor-interacting protein**	AIP
0,029	-0,74	0,60	**59,97**	Q13642	**Four and a half LIM domains protein 1**	FHL1
0,047	-0,74	0,60	**60,00**	Q9NX24	**H/ACA ribonucleoprotein complex subunit 2**	NHP2
0,016	-0,74	0,60	**60,05**	P35606	**Coatomer subunit beta**	COPB2
0,012	-0,73	0,60	**60,11**	P07339	**Cathepsin D;Cathepsin D light chain;Cathepsin D heavy chain**	CTSD
0,033	-0,73	0,60	**60,14**	P62249	**40S ribosomal protein S16**	RPS16
0,018	-0,73	0,60	**60,16**	O75390	**Citrate synthase, mitochondrial**	CS
0,023	-0,73	0,60	**60,24**	Q8WX93	**Palladin**	PALLD
0,005	-0,73	0,60	**60,36**	P46781	**40S ribosomal protein S9**	RPS9
0,041	-0,73	0,60	**60,43**	Q92804	**TATA-binding protein-associated factor 2N**	TAF15
0,007	-0,72	0,61	**60,50**	P62753	**40S ribosomal protein S6**	RPS6
0,002	-0,72	0,61	**60,55**	O94760	**N(G),N(G)-dimethylarginine dimethylaminohydrolase 1**	DDAH1
0,006	-0,72	0,61	**60,56**	Q06481	**Amyloid-like protein 2**	APLP2
0,000	-0,72	0,61	**60,61**	P40429;Q6NVV1	**60S ribosomal protein L13a;Putative 60S ribosomal protein L13a-like MGC87657**	RPL13A
0,010	-0,72	0,61	**60,63**	P62851	**40S ribosomal protein S25**	RPS25
0,043	-0,72	0,61	**60,72**	O00461	**Golgi integral membrane protein 4**	GOLIM4
0,004	-0,72	0,61	**60,77**	Q15436	**Protein transport protein Sec23A**	SEC23A
0,042	-0,72	0,61	**60,90**	P61247	**40S ribosomal protein S3a**	RPS3A
0,035	-0,72	0,61	**60,90**	P10619	**Lysosomal protective protein;Lysosomal protective protein 32 kDa chain;Lysosomal**	CTSA
0,015	-0,71	0,61	**60,93**	Q8TCS8	**Polyribonucleotide nucleotidyltransferase 1, mitochondrial**	PNPT1

0,039	-0,71	0,61	**60,96**	O75935	**Dynactin subunit 3**	DCTN3
0,038	-0,71	0,61	**60,98**	Q15056	**Eukaryotic translation initiation factor 4H**	EIF4H
0,001	-0,71	0,61	**61,02**	Q70UQ0	**Inhibitor of nuclear factor kappa-B kinase-interacting protein**	IKBIP
0,014	-0,71	0,61	**61,08**	P61224;A6NIZ1	**Ras-related protein Rap-1b;Ras-related protein Rap-1b-like protein**	RAP1B
0,046	-0,71	0,61	**61,22**	Q16718	**NADH dehydrogenase [ubiquinone] 1 alpha subcomplex subunit 5**	NDUFA5
0,035	-0,71	0,61	**61,26**	Q9UGR2	**Zinc finger CCCH domain-containing protein 7B**	ZC3H7B
0,028	-0,71	0,61	**61,34**	Q8NC51	**Plasminogen activator inhibitor 1 RNA-binding protein**	SERBP1
0,050	-0,70	0,61	**61,38**	O95571	**Protein ETHE1, mitochondrial**	ETHE1
0,011	-0,70	0,61	**61,49**	P08708;P0CW22	**40S ribosomal protein S17;40S ribosomal protein S17-like**	RPS17;RPS17L
0,009	-0,70	0,62	**61,51**	P62495	**Eukaryotic peptide chain release factor subunit 1**	ETF1
0,045	-0,70	0,62	**61,54**	Q16831	**Uridine phosphorylase 1**	UPP1
0,015	-0,70	0,62	**61,56**	P48643	**T-complex protein 1 subunit epsilon**	CCT5
0,033	-0,70	0,62	**61,62**	P28066	**Proteasome subunit alpha type-5**	PSMA5
0,010	-0,69	0,62	**61,79**	Q9GZZ1	**N-alpha-acetyltransferase 50**	NAA50
0,015	-0,69	0,62	**61,79**	P02545	**Prelamin-A/C;Lamin-A/C**	LMNA
0,003	-0,69	0,62	**62,06**	P41091;Q2VIR3	**Eukaryotic translation initiation factor 2 subunit 3;Putative eukaryotic translation in**	EIF2S3;EIF2S3L
0,036	-0,69	0,62	**62,07**	O60271;Q9UPT6	**C-Jun-amino-terminal kinase-interacting protein 4**	SPAG9
0,019	-0,69	0,62	**62,13**	P35269	**General transcription factor IIF subunit 1**	GTF2F1
0,015	-0,68	0,62	**62,32**	P54578	**Ubiquitin carboxyl-terminal hydrolase 14**	USP14
0,014	-0,68	0,62	**62,39**	Q9H6T3	**RNA polymerase II-associated protein 3**	RPAP3
0,015	-0,68	0,62	**62,45**	P62277	**40S ribosomal protein S13**	RPS13
0,012	-0,68	0,63	**62,54**	Q9BSD7	**Cancer-related nucleoside-triphosphatase**	NTPCR
0,005	-0,67	0,63	**62,89**	P36776	**Lon protease homolog, mitochondrial**	LONP1
0,045	-0,67	0,63	**63,02**	Q15599	**Na(+)/H(+) exchange regulatory cofactor NHE-RF2**	SLC9A3R2
0,031	-0,66	0,63	**63,11**	P55769	**NHP2-like protein 1**	NHP2L1
0,010	-0,66	0,63	**63,16**	Q12797	**Aspartyl/asparaginyl beta-hydroxylase**	ASPH
0,001	-0,66	0,63	**63,20**	Q8ND56	**Protein LSM14 homolog A**	LSM14A
0,012	-0,66	0,63	**63,28**	P23396	**40S ribosomal protein S3**	RPS3
0,027	-0,66	0,63	**63,34**	O95816	**BAG family molecular chaperone regulator 2**	BAG2
0,024	-0,66	0,63	**63,37**	P10636	**Microtubule-associated protein tau**	MAPT
0,008	-0,66	0,63	**63,44**	Q3L8U1	**Chromodomain-helicase-DNA-binding protein 9**	CHD9
0,023	-0,66	0,63	**63,47**	P53350	**Serine/threonine-protein kinase PLK1**	PLK1
0,032	-0,65	0,64	**63,51**	Q16775	**Hydroxyacylglutathione hydrolase, mitochondrial**	HAGH

0,000	-0,65	0,64	**63,53**	Q9Y520	**Protein PRRC2C**	PRRC2C
0,005	-0,65	0,64	**63,78**	Q99832	**T-complex protein 1 subunit eta**	CCT7
0,045	-0,65	0,64	**63,83**	O60763	**General vesicular transport factor p115**	USO1
0,007	-0,65	0,64	**63,90**	O60841	**Eukaryotic translation initiation factor 5B**	EIF5B
0,046	-0,64	0,64	**63,95**	Q9BRJ6	**Uncharacterized protein C7orf50**	C7orf50
0,019	-0,64	0,64	**64,08**	P08243	**Asparagine synthetase [glutamine-hydrolyzing]**	ASNS
0,042	-0,64	0,64	**64,16**	Q14558;O60256	**Phosphoribosyl pyrophosphate synthase-associated protein 1**	PRPSAP1
0,028	-0,64	0,64	**64,18**	P56537	**Eukaryotic translation initiation factor 6**	EIF6
0,004	-0,64	0,64	**64,26**	P11279	**Lysosome-associated membrane glycoprotein 1**	LAMP1
0,002	-0,64	0,64	**64,29**	Q5T6F2	**Ubiquitin-associated protein 2**	UBAP2
0,012	-0,64	0,64	**64,31**	P11047	**Laminin subunit gamma-1**	LAMC1
0,010	-0,64	0,64	**64,32**	P46821	**Microtubule-associated protein 1B;MAP1 light chain LC1**	MAP1B
0,015	-0,64	0,64	**64,38**	Q93009	**Ubiquitin carboxyl-terminal hydrolase 7**	USP7
0,009	-0,63	0,64	**64,40**	P49590	**Probable histidine--tRNA ligase, mitochondrial**	HARS2
0,044	-0,63	0,65	**64,52**	P52594	**Arf-GAP domain and FG repeat-containing protein 1**	AGFG1
0,003	-0,63	0,65	**64,67**	Q96CT7	**Coiled-coil domain-containing protein 124**	CCDC124
0,025	-0,63	0,65	**64,69**	Q9UFW8	**CGG triplet repeat-binding protein 1**	CGGBP1
0,012	-0,62	0,65	**64,85**	Q96P16	**Regulation of nuclear pre-mRNA domain-containing protein 1A**	RPRD1A
0,041	-0,62	0,65	**64,85**	Q96K21	**Zinc finger FYVE domain-containing protein 19**	ZFYVE19
0,002	-0,62	0,65	**64,93**	P05198	**Eukaryotic translation initiation factor 2 subunit 1**	EIF2S1
0,008	-0,62	0,65	**64,98**	Q8IV08	**Phospholipase D3**	PLD3
0,001	-0,62	0,65	**65,02**	P04075	**Fructose-bisphosphate aldolase A**	ALDOA
0,043	-0,62	0,65	**65,08**	O94925	**Glutaminase kidney isoform, mitochondrial**	GLS
0,009	-0,62	0,65	**65,10**	Q13287	**N-myc-interactor**	NMI
0,034	-0,62	0,65	**65,16**	Q12841	**Follistatin-related protein 1**	FSTL1
0,005	-0,62	0,65	**65,21**	Q9NP81	**Serine--tRNA ligase, mitochondrial**	SARS2
0,004	-0,62	0,65	**65,23**	P18077	**60S ribosomal protein L35a**	RPL35A
0,018	-0,61	0,65	**65,31**	979;P0CG48;P0CG47;P6	**Ubiquitin-40S ribosomal protein S27a;Ubiquitin;40S ribosomal protein S27a;Polyubi**	RPS27A;UBC;UBB;
0,007	-0,61	0,66	**65,70**	131;Q9UBL6;O95741;Q9	**Copine-3**	CPNE3
0,017	-0,61	0,66	**65,73**	Q15637	**Splicing factor 1**	SF1
0,046	-0,60	0,66	**65,77**	O96000	**NADH dehydrogenase [ubiquinone] 1 beta subcomplex subunit 10**	NDUFB10
0,019	-0,60	0,66	**65,96**	P49915	**GMP synthase [glutamine-hydrolyzing]**	GMPS
0,006	-0,60	0,66	**65,96**	Q15008	**26S proteasome non-ATPase regulatory subunit 6**	PSMD6

0,023	-0,60	0,66	**65,97**	Q9NVA2	**Septin-11**	Sep.11
0,005	-0,60	0,66	**66,01**	Q6RFH5	**WD repeat-containing protein 74**	WDR74
0,019	-0,60	0,66	**66,01**	P50914	**60S ribosomal protein L14**	RPL14
0,009	-0,60	0,66	**66,16**	O94992	**Protein HEXIM1**	HEXIM1
0,023	-0,59	0,66	**66,40**	O75489	**NADH dehydrogenase [ubiquinone] iron-sulfur protein 3, mitochondrial**	NDUFS3
0,046	-0,59	0,66	**66,46**	Q9H223	**EH domain-containing protein 4**	EHD4
0,009	-0,59	0,67	**66,57**	P55145	**Mesencephalic astrocyte-derived neurotrophic factor**	MANF
0,017	-0,58	0,67	**67,12**	P50991	**T-complex protein 1 subunit delta**	CCT4
0,044	-0,57	0,67	**67,16**	Q9NVT9	**Armadillo repeat-containing protein 1**	ARMC1
0,017	-0,57	0,67	**67,18**	Q8N6T3	**ADP-ribosylation factor GTPase-activating protein 1**	ARFGAP1
0,022	-0,57	0,67	**67,19**	Q9NP97;Q8TF09	**Dynein light chain roadblock-type 1;Dynein light chain roadblock-type 2**	DYNLRB1;DYNLRB2
0,020	-0,57	0,67	**67,31**	P78371	**T-complex protein 1 subunit beta**	CCT2
0,013	-0,57	0,67	**67,39**	O14950;P19105;P24844	**Myosin regulatory light chain 12B;Myosin regulatory light chain 12A;Myosin regulat**	MYL12B;MYL12A;M
0,005	-0,57	0,67	**67,41**	P63173	**60S ribosomal protein L38**	RPL38
0,003	-0,57	0,67	**67,45**	Q9H0F6	**Sharpin**	SHARPIN
0,020	-0,57	0,67	**67,49**	Q08378	**Golgin subfamily A member 3**	GOLGA3
0,000	-0,56	0,68	**67,68**	P35579	**Myosin-9**	MYH9
0,026	-0,56	0,68	**67,81**	P11216	**Glycogen phosphorylase, brain form**	PYGB
0,023	-0,56	0,68	**67,83**	P48739	**Phosphatidylinositol transfer protein beta isoform**	PITPNB
0,031	-0,56	0,68	**67,87**	P23528	**Cofilin-1**	CFL1
0,012	-0,56	0,68	**67,97**	P00390	**Glutathione reductase, mitochondrial**	GSR
0,033	-0,56	0,68	**68,01**	Q9NSD9	**Phenylalanine--tRNA ligase beta subunit**	FARSB
0,005	-0,56	0,68	**68,06**	P18615	**Negative elongation factor E**	RDBP
0,019	-0,56	0,68	**68,06**	P04181	**Ornithine aminotransferase, mitochondrial;Ornithine aminotransferase, hepatic for**	OAT
0,013	-0,55	0,68	**68,08**	P62847	**40S ribosomal protein S24**	RPS24
0,048	-0,55	0,68	**68,09**	Q16513	**Serine/threonine-protein kinase N2**	PKN2
0,001	-0,55	0,68	**68,26**	Q9H6F5	**Coiled-coil domain-containing protein 86**	CCDC86
0,042	-0,55	0,68	**68,40**	P62244	**40S ribosomal protein S15a**	RPS15A
0,014	-0,55	0,68	**68,41**	P49411	**Elongation factor Tu, mitochondrial**	TUFM
0,019	-0,55	0,68	**68,48**	P30622	**CAP-Gly domain-containing linker protein 1**	CLIP1
0,001	-0,54	0,69	**68,60**	P13861;P31323	**cAMP-dependent protein kinase type II-alpha regulatory subunit**	PRKAR2A
0,000	-0,54	0,69	**68,71**	Q9BY44	**Eukaryotic translation initiation factor 2A**	EIF2A
0,036	-0,54	0,69	**68,71**	P38646	**Stress-70 protein, mitochondrial**	HSPA9

0,024	-0,54	0,69	**68,77**	P05166	**Propionyl-CoA carboxylase beta chain, mitochondrial**	PCCB
0,012	-0,54	0,69	**68,79**	Q96JB5	**CDK5 regulatory subunit-associated protein 3**	CDK5RAP3
0,045	-0,54	0,69	**68,88**	P15289	**Arylsulfatase A;Arylsulfatase A component B;Arylsulfatase A component C**	ARSA
0,025	-0,53	0,69	**69,19**	Q8N684	**Cleavage and polyadenylation specificity factor subunit 7**	CPSF7
0,030	-0,53	0,69	**69,30**	P11142	**Heat shock cognate 71 kDa protein**	HSPA8
0,009	-0,53	0,69	**69,32**	O60220	**Mitochondrial import inner membrane translocase subunit Tim8 A**	TIMM8A
0,028	-0,53	0,69	**69,33**	P36578	**60S ribosomal protein L4**	RPL4
0,031	-0,53	0,69	**69,41**	P54652	**Heat shock-related 70 kDa protein 2**	HSPA2
0,030	-0,53	0,69	**69,42**	O75976	**Carboxypeptidase D**	CPD
0,031	-0,53	0,69	**69,48**	P30048	**Thioredoxin-dependent peroxide reductase, mitochondrial**	PRDX3
0,017	-0,53	0,69	**69,49**	P46926	**Glucosamine-6-phosphate isomerase 1**	GNPDA1
0,042	-0,52	0,70	**69,53**	Q9BUP3	**Oxidoreductase HTATIP2**	HTATIP2
0,016	-0,52	0,70	**69,60**	P48730	**Casein kinase I isoform delta**	CSNK1D
0,049	-0,52	0,70	**69,60**	Q6QNY0	**Biogenesis of lysosome-related organelles complex 1 subunit 3**	BLOC1S3
0,031	-0,52	0,70	**69,87**	Q96C01	**Protein FAM136A**	FAM136A
0,008	-0,52	0,70	**69,94**	P46776	**60S ribosomal protein L27a**	RPL27A
0,008	-0,51	0,70	**70,14**	Q7L2E3	**Putative ATP-dependent RNA helicase DHX30**	DHX30
0,027	-0,51	0,70	**70,20**	Q9UN81		ORF1
0,038	-0,51	0,70	**70,22**	Q3KQU3	**MAP7 domain-containing protein 1**	MAP7D1
0,001	-0,51	0,70	**70,25**	O95793	**Double-stranded RNA-binding protein Staufen homolog 1**	STAU1
0,007	-0,51	0,70	**70,36**	P48634	**Protein PRRC2A**	PRRC2A
0,031	-0,51	0,70	**70,38**	P48444	**Coatomer subunit delta**	ARCN1
0,044	-0,51	0,70	**70,40**	Q8N6H7	**ADP-ribosylation factor GTPase-activating protein 2**	ARFGAP2
0,037	-0,51	0,70	**70,41**	P42704	**Leucine-rich PPR motif-containing protein, mitochondrial**	LRPPRC
0,001	-0,51	0,70	**70,43**	P22392	**Nucleoside diphosphate kinase B**	NME2
0,010	-0,50	0,71	**70,64**	Q9NWH9	**SAFB-like transcription modulator**	SLTM
0,029	-0,50	0,71	**70,65**	Q9P0R6	**GSK3-beta interaction protein**	GSKIP
0,033	-0,50	0,71	**70,89**	Q5TZA2;Q86T23	**Rootletin**	CROCC
0,005	-0,49	0,71	**71,01**	P51608	**Methyl-CpG-binding protein 2**	MECP2
0,016	-0,49	0,71	**71,11**	P33316	**Deoxyuridine 5-triphosphate nucleotidohydrolase, mitochondrial**	DUT
0,019	-0,48	0,72	**71,75**	P55084	**Trifunctional enzyme subunit beta, mitochondrial;3-ketoacyl-CoA thiolase**	HADHB
0,036	-0,47	0,72	**72,15**	Q8WUQ7	**Uncharacterized protein C19orf29**	C19orf29
0,033	-0,47	0,72	**72,37**	P32969	**60S ribosomal protein L9**	RPL9

0,036	-0,47	0,72	**72,41**	O00233	**26S proteasome non-ATPase regulatory subunit 9**	PSMD9
0,028	-0,46	0,73	**72,51**	O00425	**Insulin-like growth factor 2 mRNA-binding protein 3**	IGF2BP3
0,014	-0,46	0,73	**72,71**	Q14258	**E3 ubiquitin/ISG15 ligase TRIM25**	TRIM25
0,036	-0,46	0,73	**72,77**	P08621	**U1 small nuclear ribonucleoprotein 70 kDa**	SNRNP70
0,002	-0,46	0,73	**72,79**	P60660	**Myosin light polypeptide 6**	MYL6
0,027	-0,46	0,73	**72,83**	P31937	**3-hydroxyisobutyrate dehydrogenase, mitochondrial**	HIBADH
0,038	-0,46	0,73	**72,85**	Q4J6C6	**Prolyl endopeptidase-like**	PREPL
0,006	-0,45	0,73	**73,19**	Q96FV2	**Secernin-2**	SCRN2
0,006	-0,45	0,73	**73,29**	Q9NSK0	**Kinesin light chain 4**	KLC4
0,046	-0,45	0,73	**73,36**	P31153;Q00266	**S-adenosylmethionine synthase isoform type-2**	MAT2A
0,013	-0,45	0,73	**73,37**	Q9Y6M1	**Insulin-like growth factor 2 mRNA-binding protein 2**	IGF2BP2
0,027	-0,44	0,74	**73,54**	Q9BW91	**ADP-ribose pyrophosphatase, mitochondrial**	NUDT9
0,020	-0,44	0,74	**73,74**	Q14157	**Ubiquitin-associated protein 2-like**	UBAP2L
0,030	-0,43	0,74	**74,28**	P12955	**Xaa-Pro dipeptidase**	PEPD
0,045	-0,43	0,74	**74,44**	P28799	**Granulins;Acrogranin;Paragranulin;Granulin-1;Granulin-2;Granulin-3;Granulin-4;Gra**	GRN
0,024	-0,43	0,74	**74,47**	Q96G03	**Phosphoglucomutase-2**	PGM2
0,040	-0,42	0,75	**74,66**	Q92688	**Acidic leucine-rich nuclear phosphoprotein 32 family member B**	ANP32B
0,017	-0,41	0,75	**75,19**	Q00341	**Vigilin**	HDLBP
0,031	-0,41	0,75	**75,44**	Q53H82	**Beta-lactamase-like protein 2**	LACTB2
0,034	-0,40	0,76	**75,63**	Q02543	**60S ribosomal protein L18a**	RPL18A
0,039	-0,40	0,76	**75,91**	Q16555;Q14194	**Dihydropyrimidinase-related protein 2**	DPYSL2
0,005	-0,39	0,76	**76,24**	Q14151	**Scaffold attachment factor B2**	SAFB2
0,033	-0,39	0,76	**76,37**	P62913	**60S ribosomal protein L11**	RPL11
0,029	-0,39	0,76	**76,39**	Q15369	**Transcription elongation factor B polypeptide 1**	TCEB1
0,031	-0,39	0,76	**76,43**	P35241	**Radixin**	RDX
0,002	-0,39	0,76	**76,45**	Q9Y2T3	**Guanine deaminase**	GDA
0,036	-0,39	0,77	**76,55**	P27816	**Microtubule-associated protein 4**	MAP4
0,048	-0,38	0,77	**76,81**	P20042	**Eukaryotic translation initiation factor 2 subunit 2**	EIF2S2
0,011	-0,37	0,77	**77,22**	O43491;Q9Y2J2	**Band 4.1-like protein 2**	EPB41L2
0,035	-0,37	0,77	**77,26**	P12814;Q08043	**Alpha-actinin-1**	ACTN1
0,023	-0,35	0,78	**78,41**	O15347	**High mobility group protein B3**	HMGB3
0,008	-0,35	0,78	**78,44**	P61353	**60S ribosomal protein L27**	RPL27
0,035	-0,34	0,79	**78,81**	P13798	**Acylamino-acid-releasing enzyme**	APEH

0,017	-0,34	0,79	**78,82**	P40939	**Trifunctional enzyme subunit alpha, mitochondrial;Long-chain enoyl-CoA hydratase;**	HADHA
0,049	-0,34	0,79	**79,05**	P49770	**Translation initiation factor eIF-2B subunit beta**	EIF2B2
0,044	-0,34	0,79	**79,13**	Q8IVF2	**Protein AHNAK2**	AHNAK2
0,042	-0,33	0,79	**79,39**	Q9Y3I0	**tRNA-splicing ligase RtcB homolog**	C22orf28
0,048	-0,33	0,79	**79,47**	P49189	**4-trimethylaminobutyraldehyde dehydrogenase**	ALDH9A1
0,041	-0,33	0,80	**79,55**	Q8IW45	**ATP-dependent (S)-NAD(P)H-hydrate dehydratase**	CARKD
0,042	-0,33	0,80	**79,73**	Q9NTK5	**Obg-like ATPase 1**	OLA1
0,025	-0,32	0,80	**79,85**	Q7Z2W4	**Zinc finger CCCH-type antiviral protein 1**	ZC3HAV1
0,015	-0,32	0,80	**79,89**	O95359	**Transforming acidic coiled-coil-containing protein 2**	TACC2
0,041	-0,32	0,80	**80,35**	P55081	**Microfibrillar-associated protein 1**	MFAP1
0,037	-0,30	0,81	**81,25**	Q9NZB2;Q5T035	**Constitutive coactivator of PPAR-gamma-like protein 1**	FAM120A
0,008	-0,29	0,82	**81,68**	P51114	**Fragile X mental retardation syndrome-related protein 1**	FXR1
0,011	-0,28	0,82	**82,23**	Q14764	**Major vault protein**	MVP
0,013	-0,27	0,83	**82,74**	P26196	**Probable ATP-dependent RNA helicase DDX6**	DDX6
0,049	-0,26	0,83	**83,50**	Q9Y5P4	**Collagen type IV alpha-3-binding protein**	COL4A3BP
0,008	-0,26	0,84	**83,72**	Q86UE4	**Protein LYRIC**	MTDH
0,010	-0,24	0,85	**84,66**	P98170	**E3 ubiquitin-protein ligase XIAP**	XIAP
0,017	-0,24	0,85	**84,73**	Q9NQR4	**Omega-amidase NIT2**	NIT2
**24 h Down**						
0,000	-4,76	0,04	**3,70**	P61513	**60S ribosomal protein L37a**	RPL37A
0,000	-4,64	0,04	**4,01**	P82933	**28S ribosomal protein S9, mitochondrial**	MRPS9
0,003	-4,12	0,06	**5,77**	P47985	**Cytochrome b-c1 complex subunit Rieske, mitochondrial;Cytochrome b-c1 complex**	UQCRFS1
0,038	-3,73	0,08	**7,52**	Q9Y4Y9	**U6 snRNA-associated Sm-like protein LSm5**	LSM5
0,001	-3,73	0,08	**7,53**	Q9UNS2	**COP9 signalosome complex subunit 3**	COPS3
0,003	-3,60	0,08	**8,26**	Q27J81	**Inverted formin-2**	INF2
0,000	-3,55	0,09	**8,51**	P17066;P48741	**Heat shock 70 kDa protein 6;Putative heat shock 70 kDa protein 7**	HSPA6;HSPA7
0,005	-3,49	0,09	**8,90**	P14927	**Cytochrome b-c1 complex subunit 7**	UQCRB
0,013	-3,48	0,09	**8,98**	O96000	**NADH dehydrogenase [ubiquinone] 1 beta subcomplex subunit 10**	NDUFB10
0,002	-3,43	0,09	**9,28**	Q12849	**G-rich sequence factor 1**	GRSF1
0,036	-3,39	0,10	**9,56**	Q15366	**Poly(rC)-binding protein 2**	PCBP2
0,018	-3,38	0,10	**9,64**	P47813;O14602	**Eukaryotic translation initiation factor 1A, X-chromosomal;Eukaryotic translation ini**	EIF1AX;EIF1AY
0,000	-3,37	0,10	**9,65**	O43181	**NADH dehydrogenase [ubiquinone] iron-sulfur protein 4, mitochondrial**	NDUFS4
0,003	-3,31	0,10	**10,09**	P14406	**Cytochrome c oxidase subunit 7A2, mitochondrial**	COX7A2

0,000	-3,30	0,10	**10,17**	P53350	**Serine/threonine-protein kinase PLK1**	PLK1
0,002	-3,27	0,10	**10,35**	Q9UBQ5	**Eukaryotic translation initiation factor 3 subunit K**	EIF3K
0,004	-3,27	0,10	**10,38**	Q9Y6M9	**NADH dehydrogenase [ubiquinone] 1 beta subcomplex subunit 9**	NDUFB9
0,020	-3,24	0,11	**10,55**	P53618	**Coatomer subunit beta**	COPB1
0,004	-3,18	0,11	**11,02**	P57105	**Synaptojanin-2-binding protein**	SYNJ2BP
0,021	-3,18	0,11	**11,07**	Q96FJ2	**Dynein light chain 2, cytoplasmic**	DYNLL2
0,020	-3,11	0,12	**11,58**	Q9Y262	**Eukaryotic translation initiation factor 3 subunit L**	EIF3L
0,000	-3,05	0,12	**12,11**	Q02241	**Kinesin-like protein KIF23**	KIF23
0,000	-3,02	0,12	**12,35**	Q9BZE1	**39S ribosomal protein L37, mitochondrial**	MRPL37
0,000	-3,00	0,13	**12,51**	Q9H0W8	**Protein SMG9**	SMG9
0,000	-2,97	0,13	**12,74**	Q96DC8	**Enoyl-CoA hydratase domain-containing protein 3, mitochondrial**	ECHDC3
0,003	-2,95	0,13	**12,91**	Q96T51	**RUN and FYVE domain-containing protein 1**	RUFY1
0,013	-2,94	0,13	**13,00**	P60228	**Eukaryotic translation initiation factor 3 subunit E**	EIF3E
0,040	-2,93	0,13	**13,16**	P15559	**NAD(P)H dehydrogenase [quinone] 1**	NQO1
0,042	-2,90	0,13	**13,44**	P09211	**Glutathione S-transferase P**	GSTP1
0,009	-2,88	0,14	**13,58**	O95470	**Sphingosine-1-phosphate lyase 1**	SGPL1
0,015	-2,85	0,14	**13,84**	O00410;O60518	**Importin-5**	IPO5
0,001	-2,84	0,14	**13,95**	Q9P015	**39S ribosomal protein L15, mitochondrial**	MRPL15
0,032	-2,80	0,14	**14,37**	P08754	**Guanine nucleotide-binding protein G(k) subunit alpha**	GNAI3
0,007	-2,75	0,15	**14,87**	Q9Y2U8	**Inner nuclear membrane protein Man1**	LEMD3
0,006	-2,75	0,15	**14,92**	P04844	**Dolichyl-diphosphooligosaccharide--protein glycosyltransferase subunit 2**	RPN2
0,011	-2,73	0,15	**15,07**	Q9H8Y8	**Golgi reassembly-stacking protein 2**	GORASP2
0,040	-2,72	0,15	**15,19**	Q9NV56	**MRG-binding protein**	MRGBP
0,000	-2,72	0,15	**15,20**	Q9NQ50	**39S ribosomal protein L40, mitochondrial**	MRPL40
0,011	-2,66	0,16	**15,79**	Q9UPV0	**Centrosomal protein of 164 kDa**	CEP164
0,003	-2,66	0,16	**15,80**	Q8WV24	**Pleckstrin homology-like domain family A member 1**	PHLDA1
0,009	-2,65	0,16	**15,90**	Q02978	**Mitochondrial 2-oxoglutarate/malate carrier protein**	SLC25A11
0,015	-2,65	0,16	**15,91**	Q9BWM7	**Sideroflexin-3**	SFXN3
0,002	-2,65	0,16	**15,93**	Q9H1I8	**Activating signal cointegrator 1 complex subunit 2**	ASCC2
0,017	-2,65	0,16	**15,96**	O00116	**Alkyldihydroxyacetonephosphate synthase, peroxisomal**	AGPS
0,000	-2,64	0,16	**16,00**	Q9H4L7	**SWI/SNF-related matrix-associated actin-dependent regulator of chromatin subfami**	SMARCAD1
0,018	-2,64	0,16	**16,01**	P49821	**NADH dehydrogenase [ubiquinone] flavoprotein 1, mitochondrial**	NDUFV1
0,001	-2,64	0,16	**16,06**	P61923	**Coatomer subunit zeta-1**	COPZ1

0,035	-2,64	0,16	**16,08**	Q14241	**Transcription elongation factor B polypeptide 3**	TCEB3
0,014	-2,63	0,16	**16,16**	O75251	**NADH dehydrogenase [ubiquinone] iron-sulfur protein 7, mitochondrial**	NDUFS7
0,045	-2,60	0,17	**16,52**	P10619	**Lysosomal protective protein;Lysosomal protective protein 32 kDa chain;Lysosomal**	CTSA
0,014	-2,58	0,17	**16,75**	Q9H479	**Fructosamine-3-kinase**	FN3K
0,003	-2,57	0,17	**16,81**	P62861	**40S ribosomal protein S30**	FAU
0,033	-2,57	0,17	**16,83**	O43324	**Eukaryotic translation elongation factor 1 epsilon-1**	EEF1E1
0,008	-2,57	0,17	**16,84**	Q9HD33	**39S ribosomal protein L47, mitochondrial**	MRPL47
0,000	-2,56	0,17	**16,96**	P62266	**40S ribosomal protein S23**	RPS23
0,009	-2,55	0,17	**17,07**	Q9BQG0	**Myb-binding protein 1A**	MYBBP1A
0,013	-2,54	0,17	**17,14**	O15379	**Histone deacetylase 3**	HDAC3
0,000	-2,53	0,17	**17,29**	Q9Y448	**Small kinetochore-associated protein**	SKAP
0,007	-2,51	0,18	**17,52**	P14854	**Cytochrome c oxidase subunit 6B1**	COX6B1
0,032	-2,51	0,18	**17,59**	P30048	**Thioredoxin-dependent peroxide reductase, mitochondrial**	PRDX3
0,002	-2,49	0,18	**17,78**	Q9P0J0	**NADH dehydrogenase [ubiquinone] 1 alpha subcomplex subunit 13**	NDUFA13
0,001	-2,49	0,18	**17,79**	P51398	**28S ribosomal protein S29, mitochondrial**	DAP3
0,020	-2,49	0,18	**17,86**	Q9UJX2	**Cell division cycle protein 23 homolog**	CDC23
0,003	-2,49	0,18	**17,86**	Q8IY17	**Neuropathy target esterase**	PNPLA6
0,032	-2,48	0,18	**17,91**	Q9BUF5	**Tubulin beta-6 chain**	TUBB6
0,032	-2,48	0,18	**17,96**	Q9UI09	**NADH dehydrogenase [ubiquinone] 1 alpha subcomplex subunit 12**	NDUFA12
0,013	-2,47	0,18	**18,01**	P36543	**V-type proton ATPase subunit E 1**	ATP6V1E1
0,001	-2,45	0,18	**18,33**	O00743	**Serine/threonine-protein phosphatase 6 catalytic subunit**	PPP6C
0,024	-2,44	0,18	**18,38**	Q8TEM1	**Nuclear pore membrane glycoprotein 210**	NUP210
0,007	-2,44	0,18	**18,48**	Q12769	**Nuclear pore complex protein Nup160**	NUP160
0,030	-2,43	0,19	**18,50**	P16455	**Methylated-DNA--protein-cysteine methyltransferase**	MGMT
0,001	-2,43	0,19	**18,52**	Q9Y6W3	**Calpain-7**	CAPN7
0,041	-2,43	0,19	**18,57**	Q9GZT3	**SRA stem-loop-interacting RNA-binding protein, mitochondrial**	SLIRP
0,002	-2,43	0,19	**18,60**	O00148	**ATP-dependent RNA helicase DDX39A**	DDX39A
0,015	-2,42	0,19	**18,66**	Q10567	**AP-1 complex subunit beta-1**	AP1B1
0,034	-2,42	0,19	**18,73**	O00425	**Insulin-like growth factor 2 mRNA-binding protein 3**	IGF2BP3
0,023	-2,42	0,19	**18,73**	Q8NDA2	**Hemicentin-2**	HMCN2
0,025	-2,37	0,19	**19,39**	Q9BVK6	**Transmembrane emp24 domain-containing protein 9**	TMED9
0,003	-2,35	0,20	**19,58**	P15407	**Fos-related antigen 1**	FOSL1
0,039	-2,34	0,20	**19,76**	P16070	**CD44 antigen**	CD44

0,032	-2,33	0,20	**19,89**	Q9HCE1	**Putative helicase MOV-10**	MOV10
0,025	-2,33	0,20	**19,89**	P56556	**NADH dehydrogenase [ubiquinone] 1 alpha subcomplex subunit 6**	NDUFA6
0,001	-2,32	0,20	**20,03**	Q8TB72	**Pumilio homolog 2**	PUM2
0,003	-2,32	0,20	**20,06**	P27635;Q96L21	**60S ribosomal protein L10;60S ribosomal protein L10-like**	RPL10;RPL10L
0,026	-2,32	0,20	**20,06**	P55209	**Nucleosome assembly protein 1-like 1**	NAP1L1
0,004	-2,31	0,20	**20,21**	Q9BUR5	**Apolipoprotein O**	APOO
0,000	-2,29	0,20	**20,38**	P14635	**G2/mitotic-specific cyclin-B1**	CCNB1
0,025	-2,28	0,21	**20,58**	Q96N67	**Dedicator of cytokinesis protein 7**	DOCK7
0,036	-2,28	0,21	**20,59**	Q07021	**Complement component 1 Q subcomponent-binding protein, mitochondrial**	C1QBP
0,010	-2,26	0,21	**20,91**	P60510	**Serine/threonine-protein phosphatase 4 catalytic subunit**	PPP4C
0,042	-2,22	0,22	**21,51**	P29992;O95837	**Guanine nucleotide-binding protein subunit alpha-11**	GNA11
0,015	-2,21	0,22	**21,56**	Q9BXS5;Q9Y6Q5	**AP-1 complex subunit mu-1**	AP1M1
0,011	-2,20	0,22	**21,77**	Q5UIP0	**Telomere-associated protein RIF1**	RIF1
0,003	-2,19	0,22	**21,88**	P08240	**Signal recognition particle receptor subunit alpha**	SRPR
0,043	-2,19	0,22	**21,92**	Q9Y2Z4	**Tyrosine--tRNA ligase, mitochondrial**	YARS2
0,017	-2,19	0,22	**21,94**	O75884	**Putative hydrolase RBBP9**	RBBP9
0,045	-2,19	0,22	**21,95**	Q9NU22	**Midasin**	MDN1
0,001	-2,18	0,22	**22,09**	O75489	**NADH dehydrogenase [ubiquinone] iron-sulfur protein 3, mitochondrial**	NDUFS3
0,034	-2,17	0,22	**22,19**	O95292	**Vesicle-associated membrane protein-associated protein B/C**	VAPB
0,036	-2,17	0,22	**22,23**	Q969H8	**UPF0556 protein C19orf10**	C19orf10
0,002	-2,17	0,22	**22,24**	O75616	**GTPase Era, mitochondrial**	ERAL1
0,014	-2,16	0,22	**22,35**	Q9NZM5	**Glioma tumor suppressor candidate region gene 2 protein**	GLTSCR2
0,013	-2,15	0,23	**22,53**	Q6P2E9	**Enhancer of mRNA-decapping protein 4**	EDC4
0,014	-2,14	0,23	**22,61**	P78362	**SRSF protein kinase 2;SRSF protein kinase 2 N-terminal;SRSF protein kinase 2 C-term**	SRPK2
0,023	-2,14	0,23	**22,62**	Q13885;Q9BVA1	**Tubulin beta-2A chain;Tubulin beta-2B chain**	TUBB2A;TUBB2B
0,025	-2,14	0,23	**22,67**	P02649	**Apolipoprotein E**	APOE
0,004	-2,14	0,23	**22,76**	P39656	**Dolichyl-diphosphooligosaccharide--protein glycosyltransferase 48 kDa subunit**	DDOST
0,004	-2,13	0,23	**22,82**	P53007	**Tricarboxylate transport protein, mitochondrial**	SLC25A1
0,006	-2,12	0,23	**22,95**	Q9NPA8	**Enhancer of yellow 2 transcription factor homolog**	ENY2
0,017	-2,11	0,23	**23,15**	Q93052	**Lipoma-preferred partner**	LPP
0,025	-2,09	0,23	**23,44**	Q9Y3C8	**Ubiquitin-fold modifier-conjugating enzyme 1**	UFC1
0,006	-2,09	0,24	**23,52**	Q9C0F1	**Centrosomal protein of 44 kDa**	CEP44
0,044	-2,09	0,24	**23,53**	O00217	**NADH dehydrogenase [ubiquinone] iron-sulfur protein 8, mitochondrial**	NDUFS8

0,001	-2,09	0,24	**23,54**	Q7Z2W9	**39S ribosomal protein L21, mitochondrial**	MRPL21
0,015	-2,09	0,24	**23,57**	Q9H9B4	**Sideroflexin-1**	SFXN1
0,035	-2,07	0,24	**23,78**	Q9Y6A4	**UPF0468 protein C16orf80**	C16orf80
0,015	-2,07	0,24	**23,82**	O14967	**Calmegin**	CLGN
0,001	-2,07	0,24	**23,85**	Q14644	**Ras GTPase-activating protein 3**	RASA3
0,015	-2,07	0,24	**23,88**	Q9NYK5	**39S ribosomal protein L39, mitochondrial**	MRPL39
0,023	-2,06	0,24	**24,02**	O75694	**Nuclear pore complex protein Nup155**	NUP155
0,000	-2,06	0,24	**24,02**	Q8IVH8	**Mitogen-activated protein kinase kinase kinase kinase 3**	MAP4K3
0,007	-2,05	0,24	**24,09**	Q9Y6K5	**2-5-oligoadenylate synthase 3**	OAS3
0,037	-2,05	0,24	**24,12**	P63313	**Thymosin beta-10**	TMSB10
0,035	-2,05	0,24	**24,20**	Q9Y2R9	**28S ribosomal protein S7, mitochondrial**	MRPS7
0,038	-2,04	0,24	**24,35**	Q8IUR7	**Armadillo repeat-containing protein 8**	ARMC8
0,006	-2,02	0,25	**24,57**	Q9NVV4	**Poly(A) RNA polymerase, mitochondrial**	MTPAP
0,000	-2,02	0,25	**24,62**	P21912	**Succinate dehydrogenase [ubiquinone] iron-sulfur subunit, mitochondrial**	SDHB
0,018	-2,02	0,25	**24,67**	Q92882	**Osteoclast-stimulating factor 1**	OSTF1
0,045	-2,02	0,25	**24,68**	Q04917	**14-3-3 protein eta**	YWHAH
0,014	-2,02	0,25	**24,69**	O95373	**Importin-7**	IPO7
0,003	-2,01	0,25	**24,77**	P46778	**60S ribosomal protein L21**	RPL21
0,028	-2,01	0,25	**24,78**	O14617	**AP-3 complex subunit delta-1**	AP3D1
0,042	-2,01	0,25	**24,81**	Q96HS1	**Serine/threonine-protein phosphatase PGAM5, mitochondrial**	PGAM5
0,008	-2,01	0,25	**24,89**	P62857	**40S ribosomal protein S28**	RPS28
0,001	-2,00	0,25	**25,02**	Q9UJS0	**Calcium-binding mitochondrial carrier protein Aralar2**	SLC25A13
0,023	-1,99	0,25	**25,14**	Q8IXI2	**Mitochondrial Rho GTPase 1**	RHOT1
0,014	-1,99	0,25	**25,15**	Q5JPE7;P69849;Q15155	**Nodal modulator 2;Nodal modulator 3;Nodal modulator 1**	NOMO2;NOMO3;N
0,023	-1,99	0,25	**25,18**	P12235	**ADP/ATP translocase 1**	SLC25A4
0,017	-1,98	0,25	**25,26**	Q9Y6X3	**MAU2 chromatid cohesion factor homolog**	MAU2
0,016	-1,98	0,25	**25,31**	Q02833	**Ras association domain-containing protein 7**	RASSF7
0,007	-1,98	0,25	**25,34**	Q9H7D7	**WD repeat-containing protein 26**	WDR26
0,022	-1,98	0,25	**25,41**	Q9Y3T9	**Nucleolar complex protein 2 homolog**	NOC2L
0,041	-1,97	0,25	**25,48**	Q5VYK3	**Proteasome-associated protein ECM29 homolog**	ECM29
0,002	-1,97	0,26	**25,51**	P04843	**Dolichyl-diphosphooligosaccharide--protein glycosyltransferase subunit 1**	RPN1
0,014	-1,97	0,26	**25,58**	Q5VTL8	**Pre-mRNA-splicing factor 38B**	PRPF38B
0,007	-1,97	0,26	**25,59**	Q9BYD1	**39S ribosomal protein L13, mitochondrial**	MRPL13

0,012	-1,97	0,26	**25,61**	Q9Y3B7	**39S ribosomal protein L11, mitochondrial**	MRPL11
0,046	-1,95	0,26	**25,82**	Q15418	**Ribosomal protein S6 kinase alpha-1**	RPS6KA1
0,015	-1,95	0,26	**25,82**	P48444	**Coatomer subunit delta**	ARCN1
0,015	-1,95	0,26	**25,84**	O14579	**Coatomer subunit epsilon**	COPE
0,020	-1,95	0,26	**25,92**	Q9BV57	**1,2-dihydroxy-3-keto-5-methylthiopentene dioxygenase**	ADI1
0,008	-1,95	0,26	**25,92**	Q86WA8	**Lon protease homolog 2, peroxisomal**	LONP2
0,006	-1,94	0,26	**26,07**	O43169	**Cytochrome b5 type B**	CYB5B
0,019	-1,94	0,26	**26,07**	Q9UIV1	**CCR4-NOT transcription complex subunit 7**	CNOT7
0,005	-1,94	0,26	**26,09**	Q9BRK5	**45 kDa calcium-binding protein**	SDF4
0,001	-1,94	0,26	**26,10**	Q99623	**Prohibitin-2**	PHB2
0,018	-1,93	0,26	**26,17**	P82912	**28S ribosomal protein S11, mitochondrial**	MRPS11
0,006	-1,93	0,26	**26,26**	Q8NE01	**Metal transporter CNNM3**	CNNM3
0,017	-1,93	0,26	**26,28**	Q9UHI6	**Probable ATP-dependent RNA helicase DDX20**	DDX20
0,001	-1,93	0,26	**26,28**	P06493	**Cyclin-dependent kinase 1**	CDK1
0,003	-1,93	0,26	**26,29**	P04350;A6NNZ2	**Tubulin beta-4A chain**	TUBB4A
0,027	-1,91	0,27	**26,59**	Q9Y2D4	**Exocyst complex component 6B**	EXOC6B
0,012	-1,91	0,27	**26,65**	P17568	**NADH dehydrogenase [ubiquinone] 1 beta subcomplex subunit 7**	NDUFB7
0,018	-1,91	0,27	**26,66**	Q86Y39	**NADH dehydrogenase [ubiquinone] 1 alpha subcomplex subunit 11**	NDUFA11
0,001	-1,91	0,27	**26,67**	Q13753	**Laminin subunit gamma-2**	LAMC2
0,024	-1,90	0,27	**26,81**	P07305	**Histone H1.0**	H1F0
0,036	-1,90	0,27	**26,88**	O43747	**AP-1 complex subunit gamma-1**	AP1G1
0,028	-1,89	0,27	**26,97**	Q9Y5Q9	**General transcription factor 3C polypeptide 3**	GTF3C3
0,002	-1,89	0,27	**26,97**	O75223	**Gamma-glutamylcyclotransferase**	GGCT
0,000	-1,89	0,27	**27,01**	Q10471	**Polypeptide N-acetylgalactosaminyltransferase 2;Polypeptide N-acetylgalactosamin**	GALNT2
0,009	-1,89	0,27	**27,02**	P62888	**60S ribosomal protein L30**	RPL30
0,019	-1,87	0,27	**27,35**	Q96GC5	**39S ribosomal protein L48, mitochondrial**	MRPL48
0,032	-1,86	0,28	**27,53**	Q13155	**Aminoacyl tRNA synthase complex-interacting multifunctional protein 2**	AIMP2
0,024	-1,85	0,28	**27,70**	Q66K14	**TBC1 domain family member 9B**	TBC1D9B
0,000	-1,85	0,28	**27,71**	Q9Y6A5	**Transforming acidic coiled-coil-containing protein 3**	TACC3
0,033	-1,84	0,28	**27,88**	Q9H0U6	**39S ribosomal protein L18, mitochondrial**	MRPL18
0,008	-1,84	0,28	**27,97**	Q13405	**39S ribosomal protein L49, mitochondrial**	MRPL49
0,048	-1,83	0,28	**28,04**	Q6P2Q9	**Pre-mRNA-processing-splicing factor 8**	PRPF8
0,024	-1,83	0,28	**28,06**	Q7Z7A4	**PX domain-containing protein kinase-like protein**	PXK

0,024	-1,83	0,28	**28,18**	Q9Y6C9	**Mitochondrial carrier homolog 2**	MTCH2
0,032	-1,81	0,28	**28,48**	Q9Y3A3	**MOB-like protein phocein**	MOB4
0,004	-1,80	0,29	**28,66**	Q9NVU0	**DNA-directed RNA polymerase III subunit RPC5**	POLR3E
0,000	-1,80	0,29	**28,68**	P08574	**Cytochrome c1, heme protein, mitochondrial**	CYC1
0,022	-1,80	0,29	**28,76**	O95365	**Zinc finger and BTB domain-containing protein 7A**	ZBTB7A
0,022	-1,79	0,29	**28,85**	Q69YH5	**Cell division cycle-associated protein 2**	CDCA2
0,002	-1,79	0,29	**28,87**	Q9Y606	**tRNA pseudouridine synthase A, mitochondrial**	PUS1
0,005	-1,79	0,29	**28,99**	Q3ZCM7	**Tubulin beta-8 chain**	TUBB8
0,013	-1,78	0,29	**29,12**	P11182	**Lipoamide acyltransferase component of branched-chain alpha-keto acid dehydroge**	DBT
0,011	-1,78	0,29	**29,22**	O43709	**Uncharacterized methyltransferase WBSCR22**	WBSCR22
0,047	-1,76	0,30	**29,51**	Q6IAA8	**Ragulator complex protein LAMTOR1**	LAMTOR1
0,018	-1,76	0,30	**29,52**	P13716	**Delta-aminolevulinic acid dehydratase**	ALAD
0,022	-1,76	0,30	**29,59**	Q9NRY5	**Protein FAM114A2**	FAM114A2
0,029	-1,76	0,30	**29,62**	Q9GZQ3	**COMM domain-containing protein 5**	COMMD5
0,037	-1,75	0,30	**29,67**	O75381	**Peroxisomal membrane protein PEX14**	PEX14
0,025	-1,75	0,30	**29,75**	P34896	**Serine hydroxymethyltransferase, cytosolic**	SHMT1
0,034	-1,75	0,30	**29,75**	O60504	**Vinexin**	SORBS3
0,003	-1,75	0,30	**29,80**	Q9NXV2	**BTB/POZ domain-containing protein KCTD5**	KCTD5
0,001	-1,74	0,30	**29,85**	O75380	**NADH dehydrogenase [ubiquinone] iron-sulfur protein 6, mitochondrial**	NDUFS6
0,006	-1,74	0,30	**29,93**	Q92692	**Poliovirus receptor-related protein 2**	PVRL2
0,001	-1,73	0,30	**30,05**	P62269	**40S ribosomal protein S18**	RPS18
0,011	-1,73	0,30	**30,07**	Q9NPI6	**mRNA-decapping enzyme 1A**	DCP1A
0,046	-1,72	0,30	**30,40**	Q86VP6	**Cullin-associated NEDD8-dissociated protein 1**	CAND1
0,001	-1,71	0,30	**30,47**	P12236	**ADP/ATP translocase 3**	SLC25A6
0,000	-1,71	0,31	**30,57**	P19404	**NADH dehydrogenase [ubiquinone] flavoprotein 2, mitochondrial**	NDUFV2
0,007	-1,69	0,31	**30,94**	P28331	**NADH-ubiquinone oxidoreductase 75 kDa subunit, mitochondrial**	NDUFS1
0,007	-1,69	0,31	**31,01**	P62244	**40S ribosomal protein S15a**	RPS15A
0,017	-1,69	0,31	**31,02**	Q7Z460	**CLIP-associating protein 1**	CLASP1
0,033	-1,69	0,31	**31,09**	Q8IWJ2	**GRIP and coiled-coil domain-containing protein 2**	GCC2
0,048	-1,68	0,31	**31,16**	Q9BY49	**Peroxisomal trans-2-enoyl-CoA reductase**	PECR
0,012	-1,68	0,31	**31,23**	P36404	**ADP-ribosylation factor-like protein 2**	ARL2
0,030	-1,68	0,31	**31,26**	Q9BYD3	**39S ribosomal protein L4, mitochondrial**	MRPL4
0,046	-1,67	0,31	**31,42**	Q13426	**DNA repair protein XRCC4**	XRCC4

0,004	-1,67	0,32	**31,51**	Q9BX10	**GTP-binding protein 2**	GTPBP2
0,039	-1,66	0,32	**31,65**	Q9BZE9	**Tether containing UBX domain for GLUT4**	ASPSCR1
0,003	-1,66	0,32	**31,68**	Q99447	**Ethanolamine-phosphate cytidylyltransferase**	PCYT2
0,007	-1,65	0,32	**31,90**	P26641	**Elongation factor 1-gamma**	EEF1G
0,003	-1,65	0,32	**31,94**	Q14165	**Malectin**	MLEC
0,021	-1,63	0,32	**32,24**	Q9BQE3	**Tubulin alpha-1C chain**	TUBA1C
0,038	-1,63	0,32	**32,30**	Q9BRJ2	**39S ribosomal protein L45, mitochondrial**	MRPL45
0,004	-1,63	0,32	**32,34**	P32969	**60S ribosomal protein L9**	RPL9
0,001	-1,63	0,32	**32,41**	O60763	**General vesicular transport factor p115**	USO1
0,006	-1,62	0,32	**32,47**	Q92542	**Nicastrin**	NCSTN
0,026	-1,61	0,33	**32,76**	Q9NVH0	**Exonuclease 3-5 domain-containing protein 2**	EXD2
0,000	-1,60	0,33	**32,96**	Q9UF33;P54753;Q1537	**Ephrin type-A receptor 2**	EPHA2
0,046	-1,59	0,33	**33,17**	Q14451	**Growth factor receptor-bound protein 7**	GRB7
0,013	-1,59	0,33	**33,33**	O14965	**Aurora kinase A**	AURKA
0,004	-1,59	0,33	**33,33**	O43920	**NADH dehydrogenase [ubiquinone] iron-sulfur protein 5**	NDUFS5
0,025	-1,58	0,33	**33,40**	Q00577	**Transcriptional activator protein Pur-alpha**	PURA
0,029	-1,58	0,33	**33,45**	O75976	**Carboxypeptidase D**	CPD
0,027	-1,58	0,33	**33,47**	Q9NR50	**Translation initiation factor eIF-2B subunit gamma**	EIF2B3
0,005	-1,58	0,34	**33,53**	Q8N9B5	**Junction-mediating and -regulatory protein**	JMY
0,037	-1,58	0,34	**33,54**	Q96Q15;Q6P435	**Serine/threonine-protein kinase SMG1**	SMG1
0,031	-1,57	0,34	**33,60**	Q4KMP7	**TBC1 domain family member 10B**	TBC1D10B
0,042	-1,57	0,34	**33,69**	Q6P9B9	**Integrator complex subunit 5**	INTS5
0,024	-1,56	0,34	**33,84**	P42345	**Serine/threonine-protein kinase mTOR**	MTOR
0,001	-1,56	0,34	**33,85**	P62906	**60S ribosomal protein L10a**	RPL10A
0,012	-1,56	0,34	**33,88**	Q9NRS6	**Sorting nexin-15**	SNX15
0,009	-1,56	0,34	**33,91**	P05388;Q8NHW5	**60S acidic ribosomal protein P0;60S acidic ribosomal protein P0-like**	RPLP0;RPLP0P6
0,036	-1,56	0,34	**33,96**	P15170;Q8IYD1	**Eukaryotic peptide chain release factor GTP-binding subunit ERF3A**	GSPT1
0,009	-1,55	0,34	**34,07**	Q9BQC6	**Ribosomal protein 63, mitochondrial**	MRP63
0,022	-1,55	0,34	**34,16**	Q9Y291	**28S ribosomal protein S33, mitochondrial**	MRPS33
0,007	-1,54	0,34	**34,34**	O15067	**Phosphoribosylformylglycinamidine synthase**	PFAS
0,004	-1,54	0,34	**34,47**	P41252	**Isoleucine--tRNA ligase, cytoplasmic**	IARS
0,020	-1,53	0,35	**34,52**	O60294	**Leucine carboxyl methyltransferase 2**	LCMT2
0,042	-1,53	0,35	**34,63**	P31040	**Succinate dehydrogenase [ubiquinone] flavoprotein subunit, mitochondrial**	SDHA

0,001	-1,53	0,35	**34,74**	P35232	**Prohibitin**	PHB
0,041	-1,53	0,35	**34,74**	Q96IX5	**Up-regulated during skeletal muscle growth protein 5**	USMG5
0,014	-1,52	0,35	**34,77**	Q99714	**3-hydroxyacyl-CoA dehydrogenase type-2**	HSD17B10
0,011	-1,52	0,35	**34,88**	Q14008	**Cytoskeleton-associated protein 5**	CKAP5
0,015	-1,52	0,35	**34,92**	Q13445	**Transmembrane emp24 domain-containing protein 1**	TMED1
0,006	-1,52	0,35	**34,92**	P11940;Q9H361;Q96DU[illegible]	**Polyadenylate-binding protein 1;Polyadenylate-binding protein 3**	PABPC1;PABPC3
0,039	-1,51	0,35	**35,00**	Q8N183	**Mimitin, mitochondrial**	NDUFAF2
0,040	-1,51	0,35	**35,04**	O95782	**AP-2 complex subunit alpha-1**	AP2A1
0,001	-1,51	0,35	**35,05**	Q7L2H7	**Eukaryotic translation initiation factor 3 subunit M**	EIF3M
0,011	-1,51	0,35	**35,13**	Q14197	**Peptidyl-tRNA hydrolase ICT1, mitochondrial**	ICT1
0,008	-1,50	0,35	**35,24**	P61313	**60S ribosomal protein L15**	RPL15
0,039	-1,49	0,35	**35,49**	P68036	**Ubiquitin-conjugating enzyme E2 L3**	UBE2L3
0,008	-1,49	0,36	**35,60**	Q96JB2	**Conserved oligomeric Golgi complex subunit 3**	COG3
0,017	-1,49	0,36	**35,66**	P27824	**Calnexin**	CANX
0,007	-1,49	0,36	**35,69**	Q5VSL9;Q9ULQ0	**Protein FAM40A**	FAM40A
0,014	-1,49	0,36	**35,71**	Q92783	**Signal transducing adapter molecule 1**	STAM
0,002	-1,48	0,36	**35,80**	P52815	**39S ribosomal protein L12, mitochondrial**	MRPL12
0,008	-1,48	0,36	**35,84**	Q14789	**Golgin subfamily B member 1**	GOLGB1
0,002	-1,46	0,36	**36,27**	Q8WVB6	**Chromosome transmission fidelity protein 18 homolog**	CHTF18
0,002	-1,46	0,36	**36,29**	P63173	**60S ribosomal protein L38**	RPL38
0,026	-1,46	0,36	**36,36**	P61962	**DDB1- and CUL4-associated factor 7**	DCAF7
0,026	-1,46	0,36	**36,39**	Q16836	**Hydroxyacyl-coenzyme A dehydrogenase, mitochondrial**	HADH
0,029	-1,46	0,36	**36,47**	P49327	**Fatty acid synthase;[Acyl-carrier-protein] S-acetyltransferase;[Acyl-carrier-protein] [illegible]**	FASN
0,026	-1,45	0,37	**36,53**	P46199	**Translation initiation factor IF-2, mitochondrial**	MTIF2
0,000	-1,45	0,37	**36,64**	O43678	**NADH dehydrogenase [ubiquinone] 1 alpha subcomplex subunit 2**	NDUFA2
0,001	-1,44	0,37	**36,77**	Q06203	**Amidophosphoribosyltransferase**	PPAT
0,004	-1,44	0,37	**36,78**	P10606	**Cytochrome c oxidase subunit 5B, mitochondrial**	COX5B
0,000	-1,44	0,37	**36,81**	Q99988	**Growth/differentiation factor 15**	GDF15
0,011	-1,44	0,37	**36,82**	P61421	**V-type proton ATPase subunit d 1**	ATP6V0D1
0,001	-1,43	0,37	**37,08**	Q8NBJ7	**Sulfatase-modifying factor 2**	SUMF2
0,010	-1,43	0,37	**37,15**	O00151	**PDZ and LIM domain protein 1**	PDLIM1
0,030	-1,43	0,37	**37,21**	O00748	**Cocaine esterase**	CES2
0,019	-1,43	0,37	**37,23**	Q14204	**Cytoplasmic dynein 1 heavy chain 1**	DYNC1H1

0,002	-1,42	0,37	**37,46**	P62701;P22090	**40S ribosomal protein S4, X isoform**	RPS4X
0,012	-1,41	0,38	**37,57**	P25098;P35626	**Beta-adrenergic receptor kinase 1;Beta-adrenergic receptor kinase 2**	ADRBK1;ADRBK2
0,026	-1,41	0,38	**37,60**	P46013	**Antigen KI-67**	MKI67
0,028	-1,41	0,38	**37,62**	Q8NHV4	**Protein NEDD1**	NEDD1
0,023	-1,40	0,38	**37,84**	Q14807	**Kinesin-like protein KIF22**	KIF22
0,001	-1,40	0,38	**37,88**	Q96HP0	**Dedicator of cytokinesis protein 6**	DOCK6
0,017	-1,40	0,38	**37,94**	Q9NRX2	**39S ribosomal protein L17, mitochondrial**	MRPL17
0,003	-1,40	0,38	**38,01**	Q9ULX9	**Transcription factor MafF**	MAFF
0,037	-1,40	0,38	**38,02**	Q14558;O60256	**Phosphoribosyl pyrophosphate synthase-associated protein 1**	PRPSAP1
0,002	-1,39	0,38	**38,21**	Q7Z6K5	**UPF0552 protein C15orf38**	C15orf38
0,044	-1,38	0,38	**38,29**	P36405	**ADP-ribosylation factor-like protein 3**	ARL3
0,004	-1,38	0,38	**38,33**	P53621	**Coatomer subunit alpha;Xenin;Proxenin**	COPA
0,032	-1,38	0,38	**38,34**	Q15477	**Helicase SKI2W**	SKIV2L
0,000	-1,38	0,38	**38,37**	Q9Y3U8	**60S ribosomal protein L36**	RPL36
0,043	-1,38	0,38	**38,41**	P13804	**Electron transfer flavoprotein subunit alpha, mitochondrial**	ETFA
0,005	-1,38	0,38	**38,46**	P54136	**Arginine--tRNA ligase, cytoplasmic**	RARS
0,025	-1,38	0,39	**38,51**	P62166	**Neuronal calcium sensor 1**	NCS1
0,021	-1,37	0,39	**38,65**	Q9P0L0	**Vesicle-associated membrane protein-associated protein A**	VAPA
0,043	-1,37	0,39	**38,67**	Q6ZNW5	**GDP-D-glucose phosphorylase C15orf58**	C15orf58
0,015	-1,37	0,39	**38,69**	Q15056	**Eukaryotic translation initiation factor 4H**	EIF4H
0,017	-1,37	0,39	**38,77**	Q13084	**39S ribosomal protein L28, mitochondrial**	MRPL28
0,041	-1,37	0,39	**38,81**	Q9UJ41	**Rab5 GDP/GTP exchange factor**	RABGEF1
0,002	-1,36	0,39	**38,83**	Q9ULF5	**Zinc transporter ZIP10**	SLC39A10
0,012	-1,35	0,39	**39,10**	P55884	**Eukaryotic translation initiation factor 3 subunit B**	EIF3B
0,003	-1,35	0,39	**39,12**	P18077	**60S ribosomal protein L35a**	RPL35A
0,036	-1,35	0,39	**39,23**	Q3MHD2	**Protein LSM12 homolog**	LSM12
0,025	-1,35	0,39	**39,28**	Q9UHV7	**Mediator of RNA polymerase II transcription subunit 13**	MED13
0,030	-1,35	0,39	**39,29**	Q8WTW3	**Conserved oligomeric Golgi complex subunit 1**	COG1
0,001	-1,35	0,39	**39,30**	Q8WX92	**Negative elongation factor B**	COBRA1
0,014	-1,35	0,39	**39,33**	Q8WVX9	**Fatty acyl-CoA reductase 1**	FAR1
0,014	-1,34	0,39	**39,42**	Q96EE3	**Nucleoporin SEH1**	SEH1L
0,004	-1,34	0,40	**39,59**	O60502	**Bifunctional protein NCOAT;Protein O-GlcNAcase;Histone acetyltransferase**	MGEA5
0,006	-1,33	0,40	**39,64**	O14618	**Copper chaperone for superoxide dismutase**	CCS

0,039	-1,33	0,40	**39,81**	Q9Y3E2	**BolA-like protein 1**	BOLA1
0,016	-1,33	0,40	**39,85**	P07947	**Tyrosine-protein kinase Yes**	YES1
0,023	-1,33	0,40	**39,88**	P00387	**NADH-cytochrome b5 reductase 3;NADH-cytochrome b5 reductase 3 membrane-bo**	CYB5R3
0,045	-1,33	0,40	**39,91**	Q6ZMI0	**Protein phosphatase 1 regulatory subunit 21**	PPP1R21
0,030	-1,32	0,40	**39,92**	O14802	**DNA-directed RNA polymerase III subunit RPC1**	POLR3A
0,002	-1,32	0,40	**39,92**	P25398	**40S ribosomal protein S12**	RPS12
0,034	-1,32	0,40	**39,97**	Q9UPN4	**5-azacytidine-induced protein 1**	AZI1
0,045	-1,32	0,40	**40,09**	Q8IZL8	**Proline-, glutamic acid- and leucine-rich protein 1**	PELP1
0,038	-1,32	0,40	**40,16**	P49459	**Ubiquitin-conjugating enzyme E2 A**	UBE2A
0,000	-1,32	0,40	**40,17**	Q12797	**Aspartyl/asparaginyl beta-hydroxylase**	ASPH
0,047	-1,32	0,40	**40,18**	Q712K3	**Ubiquitin-conjugating enzyme E2 R2**	UBE2R2
0,002	-1,31	0,40	**40,24**	Q6NUK1	**Calcium-binding mitochondrial carrier protein SCaMC-1**	SLC25A24
0,024	-1,31	0,40	**40,43**	Q9BYN8	**28S ribosomal protein S26, mitochondrial**	MRPS26
0,030	-1,31	0,40	**40,46**	P18074	**TFIIH basal transcription factor complex helicase XPD subunit**	ERCC2
0,021	-1,31	0,40	**40,47**	Q86TB9	**Protein PAT1 homolog 1**	PATL1
0,006	-1,30	0,40	**40,49**	Q01780	**Exosome component 10**	EXOSC10
0,011	-1,30	0,41	**40,51**	Q9BVG4	**UPF0368 protein Cxorf26**	CXorf26
0,004	-1,30	0,41	**40,62**	P78344	**Eukaryotic translation initiation factor 4 gamma 2**	EIF4G2
0,001	-1,29	0,41	**40,83**	Q99729	**Heterogeneous nuclear ribonucleoprotein A/B**	HNRNPAB
0,000	-1,29	0,41	**40,99**	P22695	**Cytochrome b-c1 complex subunit 2, mitochondrial**	UQCRC2
0,018	-1,29	0,41	**41,02**	Q9Y4B6	**Protein VPRBP**	VPRBP
0,035	-1,28	0,41	**41,08**	Q6P158	**Putative ATP-dependent RNA helicase DHX57**	DHX57
0,034	-1,28	0,41	**41,14**	Q9BVL2	**Nucleoporin p58/p45**	NUPL1
0,002	-1,28	0,41	**41,23**	Q14847	**LIM and SH3 domain protein 1**	LASP1
0,030	-1,27	0,41	**41,43**	Q6UWZ7	**BRCA1-A complex subunit Abraxas**	FAM175A
0,045	-1,27	0,41	**41,48**	P62942	**Peptidyl-prolyl cis-trans isomerase FKBP1A**	FKBP1A
0,001	-1,27	0,42	**41,53**	P51970	**NADH dehydrogenase [ubiquinone] 1 alpha subcomplex subunit 8**	NDUFA8
0,049	-1,27	0,42	**41,55**	Q96CU9	**FAD-dependent oxidoreductase domain-containing protein 1**	FOXRED1
0,041	-1,27	0,42	**41,58**	P36873	**Serine/threonine-protein phosphatase PP1-gamma catalytic subunit**	PPP1CC
0,015	-1,25	0,42	**42,01**	Q99653	**Calcium-binding protein p22**	CHP
0,022	-1,25	0,42	**42,15**	O94973	**AP-2 complex subunit alpha-2**	AP2A2
0,000	-1,24	0,42	**42,26**	P40429;Q6NVV1	**60S ribosomal protein L13a;Putative 60S ribosomal protein L13a-like MGC87657**	RPL13A
0,033	-1,24	0,42	**42,30**	Q2TB90	**Putative hexokinase HKDC1**	HKDC1

0,035	-1,24	0,42	**42,40**	O95453	**Poly(A)-specific ribonuclease PARN**	PARN
0,029	-1,23	0,43	**42,53**	Q9H267	**Vacuolar protein sorting-associated protein 33B**	VPS33B
0,019	-1,23	0,43	**42,57**	Q8NFH3	**Nucleoporin Nup43**	NUP43
0,035	-1,23	0,43	**42,64**	O15372	**Eukaryotic translation initiation factor 3 subunit H**	EIF3H
0,014	-1,22	0,43	**42,88**	Q9NRG0	**Chromatin accessibility complex protein 1**	CHRAC1
0,030	-1,22	0,43	**42,91**	Q8NE86	**Calcium uniporter protein, mitochondrial**	MCU
0,035	-1,22	0,43	**43,04**	O60825;Q16875;P1611	**6-phosphofructo-2-kinase/fructose-2,6-bisphosphatase 2;6-phosphofructo-2-kinase;**	PFKFB2
0,022	-1,21	0,43	**43,12**	Q9BT30	**Probable alpha-ketoglutarate-dependent dioxygenase ABH7**	ALKBH7
0,043	-1,21	0,43	**43,17**	O43824	**Putative GTP-binding protein 6**	GTPBP6
0,013	-1,21	0,43	**43,24**	Q9HD26	**Golgi-associated PDZ and coiled-coil motif-containing protein**	GOPC
0,026	-1,21	0,43	**43,25**	Q7Z4Q2	**HEAT repeat-containing protein 3**	HEATR3
0,030	-1,21	0,43	**43,27**	Q96HQ2	**CDKN2AIP N-terminal-like protein**	CDKN2AIPNL
0,001	-1,21	0,43	**43,29**	P13073	**Cytochrome c oxidase subunit 4 isoform 1, mitochondrial**	COX4I1
0,001	-1,20	0,43	**43,46**	Q8NBJ5	**Procollagen galactosyltransferase 1**	GLT25D1
0,018	-1,19	0,44	**43,77**	Q16539	**Mitogen-activated protein kinase 14**	MAPK14
0,006	-1,19	0,44	**43,81**	P28288	**ATP-binding cassette sub-family D member 3**	ABCD3
0,022	-1,19	0,44	**43,85**	Q9Y678	**Coatomer subunit gamma-1**	COPG1
0,002	-1,19	0,44	**43,93**	Q92974	**Rho guanine nucleotide exchange factor 2**	ARHGEF2
0,035	-1,18	0,44	**43,98**	Q92797	**Symplekin**	SYMPK
0,009	-1,18	0,44	**44,25**	Q16698	**2,4-dienoyl-CoA reductase, mitochondrial**	DECR1
0,038	-1,18	0,44	**44,25**	Q8IVM0	**Coiled-coil domain-containing protein 50**	CCDC50
0,001	-1,17	0,44	**44,41**	Q8TDN6	**Ribosome biogenesis protein BRX1 homolog**	BRIX1
0,021	-1,16	0,45	**44,61**	Q00610;P53675	**Clathrin heavy chain 1**	CLTC
0,015	-1,16	0,45	**44,67**	O75175	**CCR4-NOT transcription complex subunit 3**	CNOT3
0,014	-1,16	0,45	**44,69**	Q56VL3	**OCIA domain-containing protein 2**	OCIAD2
0,014	-1,15	0,45	**44,91**	Q7L9L4	**MOB kinase activator 1B**	MOB1B
0,032	-1,15	0,45	**44,92**	Q9Y3D0	**Mitotic spindle-associated MMXD complex subunit MIP18**	FAM96B
0,000	-1,15	0,45	**44,99**	P11717	**Cation-independent mannose-6-phosphate receptor**	IGF2R
0,025	-1,15	0,45	**45,04**	Q9Y676	**28S ribosomal protein S18b, mitochondrial**	MRPS18B
0,042	-1,15	0,45	**45,16**	Q9UGN5	**Poly [ADP-ribose] polymerase 2**	PARP2
0,016	-1,15	0,45	**45,16**	Q8N766	**Uncharacterized protein KIAA0090**	KIAA0090
0,048	-1,15	0,45	**45,18**	P45877	**Peptidyl-prolyl cis-trans isomerase C**	PPIC
0,021	-1,14	0,45	**45,24**	Q99728	**BRCA1-associated RING domain protein 1**	BARD1

0,000	-1,14	0,45	**45,26**	Q5JTW2	**Centrosomal protein of 78 kDa**	CEP78
0,018	-1,14	0,45	**45,27**	Q9Y6E0	**Serine/threonine-protein kinase 24;Serine/threonine-protein kinase 24 36 kDa subu**	STK24
0,001	-1,14	0,45	**45,31**	Q9UMX5	**Neudesin**	NENF
0,018	-1,14	0,45	**45,31**	O00178	**GTP-binding protein 1**	GTPBP1
0,000	-1,14	0,46	**45,52**	Q9H0H5	**Rac GTPase-activating protein 1**	RACGAP1
0,002	-1,13	0,46	**45,60**	P20618	**Proteasome subunit beta type-1**	PSMB1
0,005	-1,13	0,46	**45,65**	Q96J01	**THO complex subunit 3**	THOC3
0,000	-1,13	0,46	**45,65**	Q12882	**Dihydropyrimidine dehydrogenase [NADP(+)]**	DPYD
0,031	-1,13	0,46	**45,72**	Q9H8V3	**Protein ECT2**	ECT2
0,049	-1,13	0,46	**45,78**	Q8NBN7	**Retinol dehydrogenase 13**	RDH13
0,001	-1,13	0,46	**45,84**	P31930	**Cytochrome b-c1 complex subunit 1, mitochondrial**	UQCRC1
0,027	-1,12	0,46	**45,95**	Q12789	**General transcription factor 3C polypeptide 1**	GTF3C1
0,025	-1,11	0,46	**46,38**	P63000;P15153;P60763	**Ras-related C3 botulinum toxin substrate 1;Ras-related C3 botulinum toxin substrat**	RAC1;RAC2;RAC3
0,001	-1,11	0,46	**46,40**	Q96II8	**Leucine-rich repeat and calponin homology domain-containing protein 3**	LRCH3
0,005	-1,10	0,47	**46,60**	P12814;Q08043	**Alpha-actinin-1**	ACTN1
0,047	-1,10	0,47	**46,65**	P28065	**Proteasome subunit beta type-9**	PSMB9
0,006	-1,10	0,47	**46,70**	P26640	**Valine--tRNA ligase**	VARS
0,039	-1,10	0,47	**46,71**	Q99584	**Protein S100-A13**	S100A13
0,026	-1,10	0,47	**46,80**	O00303	**Eukaryotic translation initiation factor 3 subunit F**	EIF3F
0,001	-1,09	0,47	**46,89**	Q08378	**Golgin subfamily A member 3**	GOLGA3
0,001	-1,09	0,47	**46,93**	B5ME19;Q99613	**Eukaryotic translation initiation factor 3 subunit C**	EIF3CL;EIF3C
0,005	-1,08	0,47	**47,18**	O00487	**26S proteasome non-ATPase regulatory subunit 14**	PSMD14
0,012	-1,08	0,47	**47,26**	Q9BTT6	**Leucine-rich repeat-containing protein 1**	LRRC1
0,007	-1,08	0,47	**47,34**	P35613	**Basigin**	BSG
0,036	-1,08	0,47	**47,37**	P09651;Q32P51	**Heterogeneous nuclear ribonucleoprotein A1;Heterogeneous nuclear ribonucleopro**	HNRNPA1;HNRNPA
0,001	-1,08	0,47	**47,38**	P07437	**Tubulin beta chain**	TUBB
0,019	-1,08	0,47	**47,43**	P68104;Q5VTE0	**Elongation factor 1-alpha 1;Putative elongation factor 1-alpha-like 3**	EEF1A1;EEF1A1P5
0,000	-1,07	0,48	**47,51**	Q6PIU2	**Neutral cholesterol ester hydrolase 1**	NCEH1
0,042	-1,06	0,48	**47,85**	Q8WWH5	**Probable tRNA pseudouridine synthase 1**	TRUB1
0,017	-1,06	0,48	**47,92**	Q9BSH4	**Translational activator of cytochrome c oxidase 1**	TACO1
0,018	-1,06	0,48	**47,97**	Q9UBP6	**tRNA (guanine-N(7)-)-methyltransferase**	METTL1
0,008	-1,06	0,48	**47,98**	Q9Y333	**U6 snRNA-associated Sm-like protein LSm2**	LSM2
0,014	-1,06	0,48	**48,10**	P00441	**Superoxide dismutase [Cu-Zn]**	SOD1

0,028	-1,06	0,48	**48,13**	P55010	**Eukaryotic translation initiation factor 5**	EIF5
0,014	-1,05	0,48	**48,14**	O96008	**Mitochondrial import receptor subunit TOM40 homolog**	TOMM40
0,038	-1,05	0,48	**48,20**	P78346	**Ribonuclease P protein subunit p30**	RPP30
0,048	-1,05	0,48	**48,35**	Q9BRR8	**G patch domain-containing protein 1**	GPATCH1
0,029	-1,05	0,48	**48,39**	Q96IZ7	**Serine/Arginine-related protein 53**	RSRC1
0,007	-1,05	0,48	**48,41**	Q9BRJ7	**Protein syndesmos**	NUDT16L1
0,002	-1,04	0,49	**48,51**	P83731	**60S ribosomal protein L24**	RPL24
0,009	-1,04	0,49	**48,65**	O14907	**Tax1-binding protein 3**	TAX1BP3
0,035	-1,04	0,49	**48,76**	Q8WVM8	**Sec1 family domain-containing protein 1**	SCFD1
0,014	-1,03	0,49	**48,83**	Q10469	**Alpha-1,6-mannosyl-glycoprotein 2-beta-N-acetylglucosaminyltransferase**	MGAT2
0,009	-1,03	0,49	**48,92**	Q96CS3	**FAS-associated factor 2**	FAF2
0,044	-1,03	0,49	**48,94**	O15020	**Spectrin beta chain, brain 2**	SPTBN2
0,011	-1,02	0,49	**49,22**	Q8WXX5	**DnaJ homolog subfamily C member 9**	DNAJC9
0,040	-1,02	0,49	**49,30**	Q9UJW0	**Dynactin subunit 4**	DCTN4
0,020	-1,02	0,49	**49,34**	Q8WYQ5	**Microprocessor complex subunit DGCR8**	DGCR8
0,001	-1,02	0,49	**49,35**	O76003	**Glutaredoxin-3**	GLRX3
0,015	-1,02	0,49	**49,38**	Q9Y5J7	**Mitochondrial import inner membrane translocase subunit Tim9**	TIMM9
0,023	-1,02	0,49	**49,42**	Q09161	**Nuclear cap-binding protein subunit 1**	NCBP1
0,010	-1,02	0,49	**49,45**	Q9ULC3	**Ras-related protein Rab-23**	RAB23
0,001	-1,02	0,49	**49,46**	O95881	**Thioredoxin domain-containing protein 12**	TXNDC12
0,015	-1,01	0,50	**49,52**	Q7L5Y9	**Macrophage erythroblast attacher**	MAEA
0,012	-1,01	0,50	**49,63**	Q96CN7	**Isochorismatase domain-containing protein 1**	ISOC1
0,021	-1,01	0,50	**49,80**	Q01968	**Inositol polyphosphate 5-phosphatase OCRL-1**	OCRL
0,009	-1,00	0,50	**49,93**	P57740	**Nuclear pore complex protein Nup107**	NUP107
0,001	-1,00	0,50	**49,96**	P49755	**Transmembrane emp24 domain-containing protein 10**	TMED10
0,020	-1,00	0,50	**50,05**	P26373	**60S ribosomal protein L13**	RPL13
0,009	-1,00	0,50	**50,09**	Q13496	**Myotubularin**	MTM1
0,005	-1,00	0,50	**50,11**	Q15813	**Tubulin-specific chaperone E**	TBCE
0,022	-1,00	0,50	**50,15**	Q92900	**Regulator of nonsense transcripts 1**	UPF1
0,041	-0,99	0,50	**50,21**	Q96G01	**Protein bicaudal D homolog 1**	BICD1
0,017	-0,99	0,50	**50,24**	P50452	**Serpin B8**	SERPINB8
0,005	-0,99	0,50	**50,45**	Q5TA45	**Integrator complex subunit 11**	CPSF3L
0,002	-0,99	0,50	**50,47**	Q9Y4W6;Q01484	**AFG3-like protein 2**	AFG3L2

0,024	-0,99	0,50	**50,49**	Q6ZNB6	**NF-X1-type zinc finger protein NFXL1**	NFXL1
0,007	-0,99	0,51	**50,51**	Q9UNN5	**FAS-associated factor 1**	FAF1
0,015	-0,98	0,51	**50,58**	Q6IA69	**Glutamine-dependent NAD(+) synthetase**	NADSYN1
0,032	-0,98	0,51	**50,66**	Q96SB4;Q9UPE1	**SRSF protein kinase 1**	SRPK1
0,032	-0,98	0,51	**50,68**	Q71SY5	**Mediator of RNA polymerase II transcription subunit 25**	MED25
0,002	-0,98	0,51	**50,71**	Q9BRT3	**Migration and invasion enhancer 1**	MIEN1
0,014	-0,98	0,51	**50,76**	O95248	**Myotubularin-related protein 5**	SBF1
0,013	-0,98	0,51	**50,77**	P52948	**Nuclear pore complex protein Nup98-Nup96;Nuclear pore complex protein Nup98;N**	NUP98
0,011	-0,98	0,51	**50,79**	Q9NRF9	**DNA polymerase epsilon subunit 3**	POLE3
0,005	-0,98	0,51	**50,84**	P08865	**40S ribosomal protein SA**	RPSA
0,002	-0,97	0,51	**50,88**	P49790	**Nuclear pore complex protein Nup153**	NUP153
0,023	-0,97	0,51	**50,89**	Q7L2J0	**7SK snRNA methylphosphate capping enzyme**	MEPCE
0,000	-0,97	0,51	**51,06**	P62241	**40S ribosomal protein S8**	RPS8
0,035	-0,97	0,51	**51,18**	Q6IN84	**rRNA methyltransferase 1, mitochondrial**	MRM1
0,000	-0,97	0,51	**51,19**	Q02543	**60S ribosomal protein L18a**	RPL18A
0,023	-0,96	0,51	**51,30**	Q15003	**Condensin complex subunit 2**	NCAPH
0,029	-0,96	0,51	**51,37**	Q5C9Z4	**Nucleolar MIF4G domain-containing protein 1**	NOM1
0,001	-0,96	0,51	**51,39**	Q9H9T3	**Elongator complex protein 3**	ELP3
0,000	-0,96	0,51	**51,40**	P22830	**Ferrochelatase, mitochondrial**	FECH
0,000	-0,96	0,51	**51,41**	P62280	**40S ribosomal protein S11**	RPS11
0,037	-0,96	0,51	**51,42**	P30419;O60551	**Glycylpeptide N-tetradecanoyltransferase 1**	NMT1
0,000	-0,95	0,52	**51,59**	Q92793	**CREB-binding protein**	CREBBP
0,002	-0,95	0,52	**51,70**	Q13310;P0CB38	**Polyadenylate-binding protein 4**	PABPC4
0,001	-0,95	0,52	**51,80**	P61457	**Pterin-4-alpha-carbinolamine dehydratase**	PCBD1
0,017	-0,94	0,52	**52,07**	O43813	**LanC-like protein 1**	LANCL1
0,046	-0,94	0,52	**52,10**	Q9UDT6	**CAP-Gly domain-containing linker protein 2**	CLIP2
0,000	-0,94	0,52	**52,22**	Q8N1F7	**Nuclear pore complex protein Nup93**	NUP93
0,042	-0,94	0,52	**52,22**	P11388	**DNA topoisomerase 2-alpha**	TOP2A
0,021	-0,94	0,52	**52,28**	Q15154	**Pericentriolar material 1 protein**	PCM1
0,006	-0,93	0,52	**52,34**	Q92615	**La-related protein 4B**	LARP4B
0,029	-0,93	0,52	**52,38**	Q13268	**Dehydrogenase/reductase SDR family member 2**	DHRS2
0,020	-0,93	0,52	**52,41**	P63261	**Actin, cytoplasmic 2;Actin, cytoplasmic 2, N-terminally processed**	ACTG1
0,000	-0,93	0,52	**52,43**	Q9NZB2;Q5T035	**Constitutive coactivator of PPAR-gamma-like protein 1**	FAM120A

0,010	-0,93	0,53	**52,54**	Q7Z4H3	**HD domain-containing protein 2**	HDDC2
0,011	-0,93	0,53	**52,58**	Q9BXR0	**Queuine tRNA-ribosyltransferase**	QTRT1
0,017	-0,93	0,53	**52,62**	P05141	**ADP/ATP translocase 2**	SLC25A5
0,015	-0,92	0,53	**52,73**	O14787	**Transportin-2**	TNPO2
0,019	-0,92	0,53	**52,76**	Q9BPX5	**Actin-related protein 2/3 complex subunit 5-like protein**	ARPC5L
0,028	-0,92	0,53	**52,78**	Q9NRF8	**CTP synthase 2**	CTPS2
0,000	-0,92	0,53	**52,84**	O43353	**Receptor-interacting serine/threonine-protein kinase 2**	RIPK2
0,021	-0,92	0,53	**52,87**	Q9NV96;Q3MIR4	**Cell cycle control protein 50A;Cell cycle control protein 50B**	TMEM30A;TMEM3
0,026	-0,92	0,53	**52,95**	Q13188	**Serine/threonine-protein kinase 3;Serine/threonine-protein kinase 3 36kDa subunit**	STK3
0,026	-0,92	0,53	**52,98**	Q14152	**Eukaryotic translation initiation factor 3 subunit A**	EIF3A
0,002	-0,91	0,53	**53,10**	P46821	**Microtubule-associated protein 1B;MAP1 light chain LC1**	MAP1B
0,027	-0,91	0,53	**53,13**	Q9NTJ3	**Structural maintenance of chromosomes protein 4**	SMC4
0,037	-0,91	0,53	**53,36**	Q53H12	**Acylglycerol kinase, mitochondrial**	AGK
0,024	-0,90	0,53	**53,47**	P51452	**Dual specificity protein phosphatase 3**	DUSP3
0,002	-0,90	0,54	**53,63**	Q7Z4W1	**L-xylulose reductase**	DCXR
0,001	-0,90	0,54	**53,77**	P62829	**60S ribosomal protein L23**	RPL23
0,000	-0,89	0,54	**53,80**	Q92896	**Golgi apparatus protein 1**	GLG1
0,000	-0,89	0,54	**53,83**	P17812	**CTP synthase 1**	CTPS
0,003	-0,89	0,54	**53,90**	Q99661	**Kinesin-like protein KIF2C**	KIF2C
0,002	-0,89	0,54	**53,93**	Q13751	**Laminin subunit beta-3**	LAMB3
0,002	-0,89	0,54	**54,14**	Q9Y4P3	**Transducin beta-like protein 2**	TBL2
0,041	-0,88	0,54	**54,17**	P56962	**Syntaxin-17**	STX17
0,018	-0,88	0,54	**54,33**	Q15645	**Pachytene checkpoint protein 2 homolog**	TRIP13
0,003	-0,88	0,54	**54,48**	P17980	**26S protease regulatory subunit 6A**	PSMC3
0,001	-0,88	0,55	**54,50**	P49736	**DNA replication licensing factor MCM2**	MCM2
0,005	-0,88	0,55	**54,50**	Q13895	**Bystin**	BYSL
0,039	-0,87	0,55	**54,55**	P17858	**6-phosphofructokinase, liver type**	PFKL
0,000	-0,87	0,55	**54,64**	Q8IYA6	**Cytoskeleton-associated protein 2-like**	CKAP2L
0,030	-0,87	0,55	**54,70**	O95479	**GDH/6PGL endoplasmic bifunctional protein;Glucose 1-dehydrogenase;6-phosphogl**	H6PD
0,012	-0,87	0,55	**54,73**	Q14669	**Probable E3 ubiquitin-protein ligase TRIP12**	TRIP12
0,011	-0,87	0,55	**54,74**	P31946	**14-3-3 protein beta/alpha;14-3-3 protein beta/alpha, N-terminally processed**	YWHAB
0,008	-0,87	0,55	**54,88**	Q99439	**Calponin-2**	CNN2
0,014	-0,86	0,55	**54,97**	P43155	**Carnitine O-acetyltransferase**	CRAT

0,041	-0,86	0,55	**54,98**	Q16740	**Putative ATP-dependent Clp protease proteolytic subunit, mitochondrial**	CLPP
0,030	-0,86	0,55	**54,99**	Q5TDH0	**Protein DDI1 homolog 2**	DDI2
0,023	-0,86	0,55	**55,02**	Q9H9A6	**Leucine-rich repeat-containing protein 40**	LRRC40
0,000	-0,86	0,55	**55,07**	P42704	**Leucine-rich PPR motif-containing protein, mitochondrial**	LRPPRC
0,050	-0,86	0,55	**55,11**	Q9NWU2	**Protein C20orf11**	C20orf11
0,047	-0,86	0,55	**55,13**	Q9NZC9	**SWI/SNF-related matrix-associated actin-dependent regulator of chromatin subfami**	SMARCAL1
0,029	-0,86	0,55	**55,14**	Q6IBS0	**Twinfilin-2**	TWF2
0,020	-0,86	0,55	**55,14**	Q9H3P7	**Golgi resident protein GCP60**	ACBD3
0,006	-0,86	0,55	**55,24**	P23919	**Thymidylate kinase**	DTYMK
0,042	-0,86	0,55	**55,26**	Q7Z392	**Trafficking protein particle complex subunit 11**	TRAPPC11
0,017	-0,85	0,55	**55,35**	Q9UMY4	**Sorting nexin-12**	SNX12
0,035	-0,85	0,56	**55,54**	P56385	**ATP synthase subunit e, mitochondrial**	ATP5I
0,002	-0,85	0,56	**55,56**	P24534	**Elongation factor 1-beta**	EEF1B2
0,000	-0,85	0,56	**55,65**	O95834	**Echinoderm microtubule-associated protein-like 2**	EML2
0,020	-0,84	0,56	**55,74**	Q8WWM7	**Ataxin-2-like protein**	ATXN2L
0,001	-0,84	0,56	**55,76**	P08243	**Asparagine synthetase [glutamine-hydrolyzing]**	ASNS
0,002	-0,84	0,56	**55,94**	P54886	**Delta-1-pyrroline-5-carboxylate synthase;Glutamate 5-kinase;Gamma-glutamyl pho**	ALDH18A1
0,016	-0,84	0,56	**55,99**	Q9GZN8	**UPF0687 protein C20orf27**	C20orf27
0,007	-0,84	0,56	**56,00**	Q8IWZ3	**Ankyrin repeat and KH domain-containing protein 1**	ANKHD1
0,002	-0,84	0,56	**56,01**	O43716	**Glutamyl-tRNA(Gln) amidotransferase subunit C, mitochondrial**	GATC
0,009	-0,83	0,56	**56,09**	P23396	**40S ribosomal protein S3**	RPS3
0,033	-0,83	0,56	**56,13**	O75717	**WD repeat and HMG-box DNA-binding protein 1**	WDHD1
0,005	-0,83	0,56	**56,15**	Q7Z2W4	**Zinc finger CCCH-type antiviral protein 1**	ZC3HAV1
0,016	-0,83	0,56	**56,21**	P84098	**60S ribosomal protein L19**	RPL19
0,006	-0,83	0,56	**56,25**	Q05397;Q14289	**Focal adhesion kinase 1**	PTK2
0,037	-0,83	0,56	**56,28**	Q9P1U0	**DNA-directed RNA polymerase I subunit RPA12**	ZNRD1
0,032	-0,83	0,56	**56,30**	Q13308	**Inactive tyrosine-protein kinase 7**	PTK7
0,015	-0,83	0,56	**56,33**	O43707	**Alpha-actinin-4**	ACTN4
0,024	-0,83	0,56	**56,35**	O75879	**Glutamyl-tRNA(Gln) amidotransferase subunit B, mitochondrial**	PET112
0,000	-0,83	0,56	**56,42**	P30519	**Heme oxygenase 2**	HMOX2
0,014	-0,83	0,56	**56,42**	P28676	**Grancalcin**	GCA
0,019	-0,82	0,57	**56,61**	O94906	**Pre-mRNA-processing factor 6**	PRPF6
0,034	-0,82	0,57	**56,62**	Q96S44	**TP53-regulating kinase**	TP53RK

0,022	-0,82	0,57	**56,65**	P04150	**Glucocorticoid receptor**	NR3C1
0,021	-0,82	0,57	**56,69**	A6NFE2	**Uncharacterized protein C12orf70**	C12orf70
0,021	-0,82	0,57	**56,72**	P23258;Q9NRH3	**Tubulin gamma-1 chain;Tubulin gamma-2 chain**	TUBG1;TUBG2
0,016	-0,82	0,57	**56,75**	O95182	**NADH dehydrogenase [ubiquinone] 1 alpha subcomplex subunit 7**	NDUFA7
0,003	-0,82	0,57	**56,77**	Q9NR28	**Diablo homolog, mitochondrial**	DIABLO
0,039	-0,82	0,57	**56,78**	O60678	**Protein arginine N-methyltransferase 3**	PRMT3
0,040	-0,82	0,57	**56,80**	Q14318	**Peptidyl-prolyl cis-trans isomerase FKBP8**	FKBP8
0,044	-0,82	0,57	**56,81**	P48163	**NADP-dependent malic enzyme**	ME1
0,048	-0,81	0,57	**56,85**	Q9NWU5	**39S ribosomal protein L22, mitochondrial**	MRPL22
0,008	-0,81	0,57	**56,85**	O00499	**Myc box-dependent-interacting protein 1**	BIN1
0,001	-0,81	0,57	**56,98**	O95571	**Protein ETHE1, mitochondrial**	ETHE1
0,021	-0,81	0,57	**57,08**	Q9NVS2	**28S ribosomal protein S18a, mitochondrial**	MRPS18A
0,007	-0,81	0,57	**57,23**	O95817	**BAG family molecular chaperone regulator 3**	BAG3
0,025	-0,80	0,57	**57,28**	P43487	**Ran-specific GTPase-activating protein**	RANBP1
0,022	-0,80	0,58	**57,61**	Q96MW1	**Coiled-coil domain-containing protein 43**	CCDC43
0,017	-0,80	0,58	**57,63**	P67936	**Tropomyosin alpha-4 chain**	TPM4
0,014	-0,79	0,58	**57,72**	P04792	**Heat shock protein beta-1**	HSPB1
0,003	-0,79	0,58	**57,86**	P12004	**Proliferating cell nuclear antigen**	PCNA
0,026	-0,79	0,58	**57,90**	O14737	**Programmed cell death protein 5**	PDCD5
0,025	-0,79	0,58	**57,91**	Q96FW1	**Ubiquitin thioesterase OTUB1**	OTUB1
0,018	-0,79	0,58	**57,95**	Q6IN85	**Serine/threonine-protein phosphatase 4 regulatory subunit 3A**	SMEK1
0,046	-0,79	0,58	**58,00**	P09038	**Fibroblast growth factor 2**	FGF2
0,009	-0,79	0,58	**58,02**	P63244	**Guanine nucleotide-binding protein subunit beta-2-like 1**	GNB2L1
0,006	-0,79	0,58	**58,03**	P61011	**Signal recognition particle 54 kDa protein**	SRP54
0,038	-0,78	0,58	**58,05**	P08238;Q58FF7	**Heat shock protein HSP 90-beta;Putative heat shock protein HSP 90-beta-3**	HSP90AB1;HSP90A
0,031	-0,78	0,58	**58,14**	Q13347	**Eukaryotic translation initiation factor 3 subunit I**	EIF3I
0,031	-0,78	0,58	**58,31**	Q9HAV4	**Exportin-5**	XPO5
0,050	-0,78	0,58	**58,36**	O15371	**Eukaryotic translation initiation factor 3 subunit D**	EIF3D
0,016	-0,78	0,58	**58,40**	Q16134	**Electron transfer flavoprotein-ubiquinone oxidoreductase, mitochondrial**	ETFDH
0,014	-0,78	0,58	**58,43**	P19623	**Spermidine synthase**	SRM
0,001	-0,77	0,58	**58,49**	O60664	**Perilipin-3**	PLIN3
0,036	-0,77	0,59	**58,52**	P50502;Q8NFI4;Q8IZP2	**Hsc70-interacting protein;Putative protein FAM10A5;Putative protein FAM10A4**	ST13;ST13P5;ST13I
0,049	-0,77	0,59	**58,60**	Q6KC79	**Nipped-B-like protein**	NIPBL

0,003	-0,77	0,59	**58,79**	Q9H5N1	**Rab GTPase-binding effector protein 2**	RABEP2
0,001	-0,76	0,59	**58,92**	P60842	**Eukaryotic initiation factor 4A-I**	EIF4A1
0,019	-0,76	0,59	**59,07**	Q9NTX5	**Ethylmalonyl-CoA decarboxylase**	ECHDC1
0,016	-0,76	0,59	**59,12**	Q02127	**Dihydroorotate dehydrogenase (quinone), mitochondrial**	DHODH
0,007	-0,76	0,59	**59,19**	P00374;Q86XF0	**Dihydrofolate reductase**	DHFR
0,018	-0,75	0,59	**59,29**	Q13442	**28 kDa heat- and acid-stable phosphoprotein**	PDAP1
0,003	-0,75	0,59	**59,34**	O43175	**D-3-phosphoglycerate dehydrogenase**	PHGDH
0,013	-0,75	0,59	**59,35**	Q14331;Q9BZ01	**Protein FRG1;Protein FRG1B**	FRG1;FRG1B
0,019	-0,75	0,59	**59,44**	O94874	**E3 UFM1-protein ligase 1**	UFL1
0,000	-0,75	0,60	**59,60**	Q99798	**Aconitate hydratase, mitochondrial**	ACO2
0,002	-0,75	0,60	**59,61**	Q96EY1	**DnaJ homolog subfamily A member 3, mitochondrial**	DNAJA3
0,025	-0,75	0,60	**59,63**	P61081	**NEDD8-conjugating enzyme Ubc12**	UBE2M
0,013	-0,74	0,60	**59,78**	Q8WW59	**SPRY domain-containing protein 4**	SPRYD4
0,000	-0,74	0,60	**59,87**	Q07065	**Cytoskeleton-associated protein 4**	CKAP4
0,039	-0,74	0,60	**59,99**	O43237	**Cytoplasmic dynein 1 light intermediate chain 2**	DYNC1LI2
0,009	-0,74	0,60	**59,99**	Q8N9N7	**Leucine-rich repeat-containing protein 57**	LRRC57
0,003	-0,74	0,60	**60,02**	Q14258	**E3 ubiquitin/ISG15 ligase TRIM25**	TRIM25
0,001	-0,74	0,60	**60,05**	Q9NYU2;Q9NYU1	**UDP-glucose:glycoprotein glucosyltransferase 1**	UGGT1
0,024	-0,73	0,60	**60,12**	Q86X55	**Histone-arginine methyltransferase CARM1**	CARM1
0,003	-0,73	0,60	**60,13**	O95347	**Structural maintenance of chromosomes protein 2**	SMC2
0,000	-0,73	0,60	**60,21**	Q6L8Q7	**2,5-phosphodiesterase 12**	PDE12
0,044	-0,73	0,60	**60,25**	Q9BY43	**Charged multivesicular body protein 4a**	CHMP4A
0,048	-0,73	0,60	**60,25**	Q9NPA0	**UPF0480 protein C15orf24**	C15orf24
0,013	-0,73	0,60	**60,28**	O60925	**Prefoldin subunit 1**	PFDN1
0,003	-0,73	0,60	**60,29**	Q8TAE6	**Protein phosphatase 1 regulatory subunit 14C**	PPP1R14C
0,003	-0,73	0,60	**60,33**	P51553	**Isocitrate dehydrogenase [NAD] subunit gamma, mitochondrial**	IDH3G
0,047	-0,73	0,60	**60,38**	Q5VW32	**BRO1 domain-containing protein BROX**	BROX
0,006	-0,73	0,60	**60,48**	P11142	**Heat shock cognate 71 kDa protein**	HSPA8
0,001	-0,72	0,61	**60,53**	Q96S99	**Pleckstrin homology domain-containing family F member 1**	PLEKHF1
0,026	-0,72	0,61	**60,69**	P63104	**14-3-3 protein zeta/delta**	YWHAZ
0,050	-0,72	0,61	**60,74**	Q8TE77	**Protein phosphatase Slingshot homolog 3**	SSH3
0,036	-0,72	0,61	**60,84**	Q96AG4	**Leucine-rich repeat-containing protein 59**	LRRC59
0,019	-0,71	0,61	**60,92**	P07741	**Adenine phosphoribosyltransferase**	APRT

0,005	-0,71	0,61	**60,95**	P46926	**Glucosamine-6-phosphate isomerase 1**	GNPDA1
0,004	-0,71	0,61	**61,02**	Q71RC2	**La-related protein 4**	LARP4
0,005	-0,71	0,61	**61,06**	Q8IYI6	**Exocyst complex component 8**	EXOC8
0,034	-0,71	0,61	**61,12**	P46783;Q9NQ39	**40S ribosomal protein S10**	RPS10
0,024	-0,71	0,61	**61,17**	P30044	**Peroxiredoxin-5, mitochondrial**	PRDX5
0,011	-0,71	0,61	**61,22**	Q2NL82	**Pre-rRNA-processing protein TSR1 homolog**	TSR1
0,035	-0,70	0,61	**61,43**	Q96A33	**Coiled-coil domain-containing protein 47**	CCDC47
0,003	-0,70	0,61	**61,47**	Q04637	**Eukaryotic translation initiation factor 4 gamma 1**	EIF4G1
0,003	-0,70	0,62	**61,69**	O43815	**Striatin**	STRN
0,026	-0,69	0,62	**61,78**	Q16881	**Thioredoxin reductase 1, cytoplasmic**	TXNRD1
0,003	-0,69	0,62	**61,79**	Q9NR45	**Sialic acid synthase**	NANS
0,023	-0,69	0,62	**61,82**	P62277	**40S ribosomal protein S13**	RPS13
0,001	-0,69	0,62	**61,85**	Q9BTW9	**Tubulin-specific chaperone D**	TBCD
0,005	-0,69	0,62	**61,85**	Q9NQT8	**Kinesin-like protein KIF13B**	KIF13B
0,031	-0,69	0,62	**61,86**	O95071	**E3 ubiquitin-protein ligase UBR5**	UBR5
0,019	-0,69	0,62	**61,93**	P19838	**Nuclear factor NF-kappa-B p105 subunit;Nuclear factor NF-kappa-B p50 subunit**	NFKB1
0,008	-0,69	0,62	**61,94**	Q8TBX8	**Phosphatidylinositol 5-phosphate 4-kinase type-2 gamma**	PIP4K2C
0,045	-0,69	0,62	**62,00**	Q9NTI5	**Sister chromatid cohesion protein PDS5 homolog B**	PDS5B
0,048	-0,69	0,62	**62,06**	P26196	**Probable ATP-dependent RNA helicase DDX6**	DDX6
0,026	-0,69	0,62	**62,15**	P55735	**Protein SEC13 homolog**	SEC13
0,029	-0,68	0,62	**62,25**	P50416	**Carnitine O-palmitoyltransferase 1, liver isoform**	CPT1A
0,045	-0,68	0,62	**62,31**	Q9NZN8	**CCR4-NOT transcription complex subunit 2**	CNOT2
0,040	-0,68	0,62	**62,32**	Q9Y2R0	**Coiled-coil domain-containing protein 56**	CCDC56
0,006	-0,68	0,62	**62,35**	Q9BUH6	**Uncharacterized protein C9orf142**	C9orf142
0,000	-0,68	0,62	**62,40**	Q8NBS9	**Thioredoxin domain-containing protein 5**	TXNDC5
0,003	-0,68	0,62	**62,49**	O60524	**Nuclear export mediator factor NEMF**	NEMF
0,000	-0,68	0,63	**62,51**	P19174	**1-phosphatidylinositol 4,5-bisphosphate phosphodiesterase gamma-1**	PLCG1
0,001	-0,68	0,63	**62,51**	Q8IWB7	**WD repeat and FYVE domain-containing protein 1**	WDFY1
0,012	-0,68	0,63	**62,55**	Q9NSD9	**Phenylalanine--tRNA ligase beta subunit**	FARSB
0,001	-0,67	0,63	**62,72**	P46060	**Ran GTPase-activating protein 1**	RANGAP1
0,034	-0,67	0,63	**62,78**	Q96A35	**39S ribosomal protein L24, mitochondrial**	MRPL24
0,005	-0,67	0,63	**62,87**	P62263	**40S ribosomal protein S14**	RPS14
0,028	-0,67	0,63	**62,87**	Q13951	**Core-binding factor subunit beta**	CBFB

0,001	-0,67	0,63	**62,89**	P46109	**Crk-like protein**	CRKL
0,023	-0,67	0,63	**62,90**	P25815	**Protein S100-P**	S100P
0,038	-0,67	0,63	**63,01**	Q8TEQ6	**Gem-associated protein 5**	GEMIN5
0,042	-0,66	0,63	**63,09**	Q9NTZ6	**RNA-binding protein 12**	RBM12
0,021	-0,66	0,63	**63,21**	Q9UKK9	**ADP-sugar pyrophosphatase**	NUDT5
0,007	-0,66	0,63	**63,34**	P52565	**Rho GDP-dissociation inhibitor 1**	ARHGDIA
0,013	-0,66	0,63	**63,39**	Q9Y496;Q9P2E2	**Kinesin-like protein KIF3A**	KIF3A
0,034	-0,66	0,63	**63,45**	A0FGR8	**Extended synaptotagmin-2**	ESYT2
0,038	-0,65	0,64	**63,51**	Q9P0M9	**39S ribosomal protein L27, mitochondrial**	MRPL27
0,009	-0,65	0,64	**63,52**	Q9NR19	**Acetyl-coenzyme A synthetase, cytoplasmic**	ACSS2
0,012	-0,65	0,64	**63,64**	Q04323	**UBX domain-containing protein 1**	UBXN1
0,000	-0,65	0,64	**63,67**	P21980	**Protein-glutamine gamma-glutamyltransferase 2**	TGM2
0,002	-0,65	0,64	**63,77**	Q9H3N1	**Thioredoxin-related transmembrane protein 1**	TMX1
0,002	-0,65	0,64	**63,79**	P12081	**Histidine--tRNA ligase, cytoplasmic**	HARS
0,041	-0,65	0,64	**63,86**	P60520	**Gamma-aminobutyric acid receptor-associated protein-like 2**	GABARAPL2
0,013	-0,65	0,64	**63,88**	P53004	**Biliverdin reductase A**	BLVRA
0,000	-0,64	0,64	**63,95**	P30050	**60S ribosomal protein L12**	RPL12
0,022	-0,64	0,64	**63,96**	Q6PI48	**Aspartate--tRNA ligase, mitochondrial**	DARS2
0,003	-0,64	0,64	**64,13**	Q13162	**Peroxiredoxin-4**	PRDX4
0,028	-0,64	0,64	**64,28**	Q9UIJ7	**GTP:AMP phosphotransferase, mitochondrial**	AK3
0,016	-0,63	0,64	**64,42**	Q16774	**Guanylate kinase**	GUK1
0,007	-0,63	0,64	**64,44**	Q8N6T3	**ADP-ribosylation factor GTPase-activating protein 1**	ARFGAP1
0,025	-0,63	0,64	**64,46**	Q96LD4	**Tripartite motif-containing protein 47**	TRIM47
0,020	-0,63	0,65	**64,57**	Q13564	**NEDD8-activating enzyme E1 regulatory subunit**	NAE1
0,018	-0,63	0,65	**64,59**	O43681	**ATPase ASNA1**	ASNA1
0,001	-0,63	0,65	**64,64**	O14964	**Hepatocyte growth factor-regulated tyrosine kinase substrate**	HGS
0,030	-0,63	0,65	**64,70**	Q8N0X7	**Spartin**	SPG20
0,044	-0,63	0,65	**64,75**	Q14116	**Interleukin-18**	IL18
0,004	-0,63	0,65	**64,77**	P30040	**Endoplasmic reticulum resident protein 29**	ERP29
0,013	-0,63	0,65	**64,83**	P12268	**Inosine-5-monophosphate dehydrogenase 2**	IMPDH2
0,007	-0,62	0,65	**64,89**	O60610	**Protein diaphanous homolog 1**	DIAPH1
0,005	-0,62	0,65	**64,90**	Q96FZ7	**Charged multivesicular body protein 6**	CHMP6
0,029	-0,62	0,65	**64,92**	Q9GZS1	**DNA-directed RNA polymerase I subunit RPA49**	POLR1E

0,023	-0,62	0,65	**65,20**	Q14694	**Ubiquitin carboxyl-terminal hydrolase 10**	USP10
0,000	-0,62	0,65	**65,21**	P52292	**Importin subunit alpha-2**	KPNA2
0,005	-0,62	0,65	**65,25**	Q8NHU6	**Tudor domain-containing protein 7**	TDRD7
0,018	-0,62	0,65	**65,25**	P55072	**Transitional endoplasmic reticulum ATPase**	VCP
0,004	-0,61	0,65	**65,38**	P43304	**Glycerol-3-phosphate dehydrogenase, mitochondrial**	GPD2
0,034	-0,61	0,66	**65,51**	Q9UHD2	**Serine/threonine-protein kinase TBK1**	TBK1
0,029	-0,61	0,66	**65,53**	P49591	**Serine--tRNA ligase, cytoplasmic**	SARS
0,009	-0,61	0,66	**65,68**	P01130	**Low-density lipoprotein receptor**	LDLR
0,025	-0,61	0,66	**65,69**	P28072	**Proteasome subunit beta type-6**	PSMB6
0,015	-0,61	0,66	**65,71**	Q7Z3T8	**Zinc finger FYVE domain-containing protein 16**	ZFYVE16
0,030	-0,60	0,66	**65,78**	Q7L5N1	**COP9 signalosome complex subunit 6**	COPS6
0,004	-0,60	0,66	**65,81**	O43252	**Bifunctional 3-phosphoadenosine 5-phosphosulfate synthase 1;Sulfate adenylyltran**	PAPSS1
0,033	-0,60	0,66	**65,83**	Q9GZP4	**PITH domain-containing protein 1**	PITHD1
0,018	-0,60	0,66	**65,86**	Q96G46	**tRNA-dihydrouridine(47) synthase [NAD(P)(+)]-like**	DUS3L
0,013	-0,60	0,66	**65,87**	O75390	**Citrate synthase, mitochondrial**	CS
0,025	-0,60	0,66	**65,95**	O95163	**Elongator complex protein 1**	IKBKAP
0,012	-0,60	0,66	**65,96**	Q9UHY7	**Enolase-phosphatase E1**	ENOPH1
0,006	-0,60	0,66	**66,01**	P61221	**ATP-binding cassette sub-family E member 1**	ABCE1
0,002	-0,60	0,66	**66,04**	O14950;P19105;P24844	**Myosin regulatory light chain 12B;Myosin regulatory light chain 12A;Myosin regulat**	MYL12B;MYL12A;M
0,002	-0,60	0,66	**66,05**	Q8IYB7	**DIS3-like exonuclease 2**	DIS3L2
0,027	-0,60	0,66	**66,17**	Q8WUX9	**Charged multivesicular body protein 7**	CHMP7
0,001	-0,59	0,66	**66,21**	Q9BUL8	**Programmed cell death protein 10**	PDCD10
0,040	-0,59	0,66	**66,23**	O95707	**Ribonuclease P protein subunit p29**	POP4
0,002	-0,59	0,66	**66,24**	Q86X76	**Nitrilase homolog 1**	NIT1
0,004	-0,59	0,66	**66,25**	P51665	**26S proteasome non-ATPase regulatory subunit 7**	PSMD7
0,045	-0,59	0,66	**66,26**	O60443	**Non-syndromic hearing impairment protein 5**	DFNA5
0,000	-0,59	0,66	**66,38**	P29692	**Elongation factor 1-delta**	EEF1D
0,024	-0,59	0,66	**66,42**	Q9BYX2	**TBC1 domain family member 2A**	TBC1D2
0,036	-0,59	0,66	**66,43**	P15880	**40S ribosomal protein S2**	RPS2
0,012	-0,59	0,66	**66,48**	Q9UQ80	**Proliferation-associated protein 2G4**	PA2G4
0,046	-0,59	0,66	**66,49**	Q92973	**Transportin-1**	TNPO1
0,000	-0,59	0,67	**66,53**	Q16775	**Hydroxyacylglutathione hydrolase, mitochondrial**	HAGH
0,001	-0,59	0,67	**66,60**	Q6P179	**Endoplasmic reticulum aminopeptidase 2**	ERAP2

0,003	-0,59	0,67	**66,63**	O00629	**Importin subunit alpha-4**	KPNA4
0,015	-0,58	0,67	**66,76**	Q9UPN3	**Microtubule-actin cross-linking factor 1, isoforms 1/2/3/5**	MACF1
0,016	-0,58	0,67	**66,76**	Q9NQP4	**Prefoldin subunit 4**	PFDN4
0,000	-0,58	0,67	**66,78**	Q14195	**Dihydropyrimidinase-related protein 3**	DPYSL3
0,002	-0,58	0,67	**66,79**	P62495	**Eukaryotic peptide chain release factor subunit 1**	ETF1
0,033	-0,58	0,67	**66,80**	P78318	**Immunoglobulin-binding protein 1**	IGBP1
0,002	-0,58	0,67	**66,85**	P11086	**Phenylethanolamine N-methyltransferase**	PNMT
0,013	-0,58	0,67	**66,94**	P50991	**T-complex protein 1 subunit delta**	CCT4
0,024	-0,58	0,67	**66,95**	P52943	**Cysteine-rich protein 2**	CRIP2
0,000	-0,58	0,67	**66,97**	Q9ULW0	**Targeting protein for Xklp2**	TPX2
0,020	-0,58	0,67	**67,00**	P78417	**Glutathione S-transferase omega-1**	GSTO1
0,015	-0,58	0,67	**67,00**	P62847	**40S ribosomal protein S24**	RPS24
0,000	-0,58	0,67	**67,05**	O43837	**Isocitrate dehydrogenase [NAD] subunit beta, mitochondrial**	IDH3B
0,000	-0,58	0,67	**67,06**	P17174	**Aspartate aminotransferase, cytoplasmic**	GOT1
0,024	-0,58	0,67	**67,09**	Q9H6E4	**Coiled-coil domain-containing protein 134**	CCDC134
0,045	-0,58	0,67	**67,13**	P59998	**Actin-related protein 2/3 complex subunit 4**	ARPC4
0,015	-0,57	0,67	**67,17**	Q9NSK0	**Kinesin light chain 4**	KLC4
0,002	-0,57	0,67	**67,21**	P82650	**28S ribosomal protein S22, mitochondrial**	MRPS22
0,006	-0,57	0,67	**67,21**	P06756	**Integrin alpha-V;Integrin alpha-V heavy chain;Integrin alpha-V light chain**	ITGAV
0,009	-0,57	0,67	**67,28**	P18206	**Vinculin**	VCL
0,000	-0,57	0,67	**67,29**	P49368	**T-complex protein 1 subunit gamma**	CCT3
0,000	-0,57	0,67	**67,32**	P54819	**Adenylate kinase 2, mitochondrial**	AK2
0,006	-0,57	0,67	**67,35**	Q12802	**A-kinase anchor protein 13**	AKAP13
0,032	-0,57	0,67	**67,37**	Q96A49	**Synapse-associated protein 1**	SYAP1
0,004	-0,57	0,67	**67,39**	P00492	**Hypoxanthine-guanine phosphoribosyltransferase**	HPRT1
0,004	-0,57	0,67	**67,40**	Q9NX02	**NACHT, LRR and PYD domains-containing protein 2**	NLRP2
0,006	-0,57	0,67	**67,49**	P49796	**Regulator of G-protein signaling 3**	RGS3
0,001	-0,57	0,68	**67,56**	Q9NP97;Q8TF09	**Dynein light chain roadblock-type 1;Dynein light chain roadblock-type 2**	DYNLRB1;DYNLRB2
0,019	-0,56	0,68	**67,64**	O15382	**Branched-chain-amino-acid aminotransferase, mitochondrial**	BCAT2
0,002	-0,56	0,68	**67,66**	Q9Y266	**Nuclear migration protein nudC**	NUDC
0,002	-0,56	0,68	**67,66**	P46781	**40S ribosomal protein S9**	RPS9
0,039	-0,56	0,68	**67,72**	Q96PQ0	**VPS10 domain-containing receptor SorCS2**	SORCS2
0,014	-0,56	0,68	**67,73**	Q9UBW8	**COP9 signalosome complex subunit 7a**	COPS7A

0,006	-0,56	0,68	**67,81**	Q9NP81	**Serine--tRNA ligase, mitochondrial**	SARS2
0,022	-0,56	0,68	**67,88**	P39019	**40S ribosomal protein S19**	RPS19
0,009	-0,56	0,68	**67,91**	Q8TEX9	**Importin-4**	IPO4
0,014	-0,56	0,68	**67,91**	P10599	**Thioredoxin**	TXN
0,034	-0,56	0,68	**67,94**	O96033	**Molybdopterin synthase sulfur carrier subunit**	MOCS2
0,001	-0,56	0,68	**67,98**	Q15417	**Calponin-3**	CNN3
0,039	-0,56	0,68	**68,01**	P40261	**Nicotinamide N-methyltransferase**	NNMT
0,015	-0,56	0,68	**68,04**	P49454	**Centromere protein F**	CENPF
0,035	-0,55	0,68	**68,26**	P31937	**3-hydroxyisobutyrate dehydrogenase, mitochondrial**	HIBADH
0,010	-0,55	0,68	**68,29**	P49753;Q86TX2	**Acyl-coenzyme A thioesterase 2, mitochondrial;Acyl-coenzyme A thioesterase 1**	ACOT2;ACOT1
0,016	-0,55	0,68	**68,44**	Q02218;Q9ULD0	**2-oxoglutarate dehydrogenase, mitochondrial**	OGDH
0,011	-0,55	0,68	**68,44**	Q66K74	**Microtubule-associated protein 1S;MAP1S heavy chain;MAP1S light chain**	MAP1S
0,015	-0,55	0,68	**68,45**	Q9Y6G9	**Cytoplasmic dynein 1 light intermediate chain 1**	DYNC1LI1
0,014	-0,55	0,68	**68,45**	P42126	**Enoyl-CoA delta isomerase 1, mitochondrial**	ECI1
0,003	-0,55	0,69	**68,51**	Q9BZQ8	**Protein Niban**	FAM129A
0,005	-0,54	0,69	**68,57**	P16949;Q93045	**Stathmin**	STMN1
0,029	-0,54	0,69	**68,60**	O15357	**Phosphatidylinositol 3,4,5-trisphosphate 5-phosphatase 2**	INPPL1
0,001	-0,54	0,69	**68,65**	Q9NR46	**Endophilin-B2**	SH3GLB2
0,010	-0,54	0,69	**68,65**	P42166	**Lamina-associated polypeptide 2, isoform alpha;Thymopoietin;Thymopentin**	TMPO
0,002	-0,54	0,69	**68,66**	P05165	**Propionyl-CoA carboxylase alpha chain, mitochondrial**	PCCA
0,028	-0,54	0,69	**68,85**	Q13642	**Four and a half LIM domains protein 1**	FHL1
0,032	-0,54	0,69	**68,86**	P61247	**40S ribosomal protein S3a**	RPS3A
0,023	-0,54	0,69	**68,92**	O60271;Q9UPT6	**C-Jun-amino-terminal kinase-interacting protein 4**	SPAG9
0,002	-0,54	0,69	**68,94**	Q16851	**UTP--glucose-1-phosphate uridylyltransferase**	UGP2
0,012	-0,54	0,69	**69,01**	Q12768	**WASH complex subunit strumpellin**	KIAA0196
0,028	-0,53	0,69	**69,09**	Q13425;Q13424	**Beta-2-syntrophin**	SNTB2
0,002	-0,53	0,69	**69,09**	Q16555;Q14194	**Dihydropyrimidinase-related protein 2**	DPYSL2
0,000	-0,53	0,69	**69,15**	P78371	**T-complex protein 1 subunit beta**	CCT2
0,014	-0,53	0,69	**69,26**	Q9BT78	**COP9 signalosome complex subunit 4**	COPS4
0,029	-0,53	0,69	**69,28**	Q07020	**60S ribosomal protein L18**	RPL18
0,003	-0,53	0,69	**69,31**	O95865	**N(G),N(G)-dimethylarginine dimethylaminohydrolase 2**	DDAH2
0,003	-0,53	0,69	**69,32**	P62158	**Calmodulin**	CALM1
0,001	-0,53	0,69	**69,40**	P63241;Q6IS14	**Eukaryotic translation initiation factor 5A-1;Eukaryotic translation initiation factor 5**	EIF5A;EIF5AL1

0,032	-0,52	0,70	**69,58**	Q9Y446	**Plakophilin-3**	PKP3
0,004	-0,52	0,70	**69,63**	Q53FA7	**Quinone oxidoreductase PIG3**	TP53I3
0,012	-0,52	0,70	**69,69**	P14902	**Indoleamine 2,3-dioxygenase 1**	IDO1
0,004	-0,52	0,70	**69,71**	P05387	**60S acidic ribosomal protein P2**	RPLP2
0,049	-0,52	0,70	**69,75**	P46937	**Yorkie homolog**	YAP1
0,000	-0,52	0,70	**69,79**	Q99832	**T-complex protein 1 subunit eta**	CCT7
0,029	-0,52	0,70	**69,85**	O00154	**Cytosolic acyl coenzyme A thioester hydrolase**	ACOT7
0,001	-0,52	0,70	**69,89**	P14314	**Glucosidase 2 subunit beta**	PRKCSH
0,007	-0,52	0,70	**69,92**	Q96L92;O60759	**Sorting nexin-27**	SNX27
0,022	-0,52	0,70	**69,98**	P78330	**Phosphoserine phosphatase**	PSPH
0,008	-0,51	0,70	**70,13**	P40925	**Malate dehydrogenase, cytoplasmic**	MDH1
0,001	-0,51	0,70	**70,14**	Q15075	**Early endosome antigen 1**	EEA1
0,042	-0,51	0,70	**70,22**	Q06323	**Proteasome activator complex subunit 1**	PSME1
0,013	-0,51	0,70	**70,23**	P30085	**UMP-CMP kinase**	CMPK1
0,005	-0,51	0,70	**70,27**	O95573	**Long-chain-fatty-acid--CoA ligase 3**	ACSL3
0,017	-0,51	0,70	**70,29**	Q9HDC9	**Adipocyte plasma membrane-associated protein**	APMAP
0,000	-0,51	0,70	**70,31**	Q12904	**Aminoacyl tRNA synthase complex-interacting multifunctional protein 1;Endothelial**	AIMP1
0,006	-0,51	0,70	**70,33**	Q9UMS0	**NFU1 iron-sulfur cluster scaffold homolog, mitochondrial**	NFU1
0,028	-0,51	0,70	**70,34**	Q9BS26	**Endoplasmic reticulum resident protein 44**	ERP44
0,042	-0,51	0,70	**70,35**	P49902	**Cytosolic purine 5-nucleotidase**	NT5C2
0,004	-0,51	0,70	**70,37**	O95757	**Heat shock 70 kDa protein 4L**	HSPA4L
0,027	-0,51	0,70	**70,41**	Q96HN2	**Putative adenosylhomocysteinase 3**	AHCYL2
0,039	-0,51	0,70	**70,46**	P25685	**DnaJ homolog subfamily B member 1**	DNAJB1
0,001	-0,50	0,70	**70,47**	P11586	**C-1-tetrahydrofolate synthase, cytoplasmic;Methylenetetrahydrofolate dehydrogen**	MTHFD1
0,000	-0,50	0,70	**70,48**	Q96AE4	**Far upstream element-binding protein 1**	FUBP1
0,007	-0,50	0,71	**70,50**	Q7Z2Z2	**Elongation factor Tu GTP-binding domain-containing protein 1**	EFTUD1
0,006	-0,50	0,71	**70,51**	P23528	**Cofilin-1**	CFL1
0,020	-0,50	0,71	**70,60**	P16885	**1-phosphatidylinositol 4,5-bisphosphate phosphodiesterase gamma-2**	PLCG2
0,006	-0,50	0,71	**70,62**	P22392	**Nucleoside diphosphate kinase B**	NME2
0,037	-0,50	0,71	**70,71**	O43583	**Density-regulated protein**	DENR
0,007	-0,50	0,71	**70,72**	Q9NTK5	**Obg-like ATPase 1**	OLA1
0,024	-0,50	0,71	**70,75**	Q05086	**Ubiquitin-protein ligase E3A**	UBE3A
0,015	-0,50	0,71	**70,76**	O14980	**Exportin-1**	XPO1

0,002	-0,50	0,71	**70,78**	Q15054	**DNA polymerase delta subunit 3**	POLD3
0,001	-0,50	0,71	**70,80**	Q9UL15	**BAG family molecular chaperone regulator 5**	BAG5
0,042	-0,50	0,71	**70,81**	Q00653	**Nuclear factor NF-kappa-B p100 subunit;Nuclear factor NF-kappa-B p52 subunit**	NFKB2
0,003	-0,50	0,71	**70,82**	P33992	**DNA replication licensing factor MCM5**	MCM5
0,042	-0,50	0,71	**70,86**	Q96Q11	**CCA tRNA nucleotidyltransferase 1, mitochondrial**	TRNT1
0,011	-0,50	0,71	**70,89**	P23588	**Eukaryotic translation initiation factor 4B**	EIF4B
0,007	-0,50	0,71	**70,90**	P28482	**Mitogen-activated protein kinase 1**	MAPK1
0,022	-0,49	0,71	**70,97**	Q9UD71	**Protein phosphatase 1 regulatory subunit 1B**	PPP1R1B
0,000	-0,49	0,71	**70,98**	Q7Z417	**Nuclear fragile X mental retardation-interacting protein 2**	NUFIP2
0,020	-0,49	0,71	**71,03**	Q9UIA9	**Exportin-7**	XPO7
0,001	-0,49	0,71	**71,10**	Q5JRX3	**Presequence protease, mitochondrial**	PITRM1
0,002	-0,49	0,71	**71,12**	Q86VS8	**Protein Hook homolog 3**	HOOK3
0,003	-0,49	0,71	**71,16**	Q13596	**Sorting nexin-1**	SNX1
0,019	-0,49	0,71	**71,21**	Q96QK1	**Vacuolar protein sorting-associated protein 35**	VPS35
0,028	-0,49	0,71	**71,21**	P33316	**Deoxyuridine 5-triphosphate nucleotidohydrolase, mitochondrial**	DUT
0,009	-0,49	0,71	**71,22**	P50990	**T-complex protein 1 subunit theta**	CCT8
0,003	-0,49	0,71	**71,24**	›51003;Q9BWT3;Q9NRJ!	**Poly(A) polymerase alpha**	PAPOLA
0,030	-0,49	0,71	**71,26**	Q9NRY4	**Rho GTPase-activating protein 35**	ARHGAP35
0,047	-0,49	0,71	**71,28**	Q53H82	**Beta-lactamase-like protein 2**	LACTB2
0,036	-0,49	0,71	**71,33**	Q15435	**Protein phosphatase 1 regulatory subunit 7**	PPP1R7
0,028	-0,49	0,71	**71,34**	Q96CW1	**AP-2 complex subunit mu**	AP2M1
0,006	-0,49	0,71	**71,34**	Q9UHV9	**Prefoldin subunit 2**	PFDN2
0,005	-0,49	0,71	**71,35**	P22314	**Ubiquitin-like modifier-activating enzyme 1**	UBA1
0,001	-0,49	0,71	**71,39**	Q3LXA3	**Bifunctional ATP-dependent dihydroxyacetone kinase/FAD-AMP lyase (cyclizing);AT**	DAK
0,001	-0,49	0,71	**71,43**	A0MZ66	**Shootin-1**	KIAA1598
0,034	-0,49	0,71	**71,43**	P15311	**Ezrin**	EZR
0,040	-0,49	0,71	**71,44**	Q9UBU9	**Nuclear RNA export factor 1**	NXF1
0,035	-0,48	0,71	**71,49**	P00568;Q9Y6K8	**Adenylate kinase isoenzyme 1**	AK1
0,002	-0,48	0,72	**71,52**	Q9UNZ2	**NSFL1 cofactor p47**	NSFL1C
0,019	-0,48	0,72	**71,58**	Q9BQA1	**Methylosome protein 50**	WDR77
0,007	-0,48	0,72	**71,59**	P25705	**ATP synthase subunit alpha, mitochondrial**	ATP5A1
0,004	-0,48	0,72	**71,68**	Q14697	**Neutral alpha-glucosidase AB**	GANAB
0,011	-0,48	0,72	**71,75**	P07858	**Cathepsin B;Cathepsin B light chain;Cathepsin B heavy chain**	CTSB

0,009	-0,48	0,72	**71,77**	Q04760	**Lactoylglutathione lyase**	GLO1
0,004	-0,48	0,72	**71,86**	Q99747	**Gamma-soluble NSF attachment protein**	NAPG
0,010	-0,48	0,72	**71,88**	Q9BXJ9;Q6N069	**N-alpha-acetyltransferase 15, NatA auxiliary subunit**	NAA15
0,008	-0,48	0,72	**71,88**	Q9BV86	**Alpha N-terminal protein methyltransferase 1A**	METTL11A
0,039	-0,47	0,72	**71,96**	Q15021	**Condensin complex subunit 1**	NCAPD2
0,008	-0,47	0,72	**72,03**	P50213	**Isocitrate dehydrogenase [NAD] subunit alpha, mitochondrial**	IDH3A
0,005	-0,47	0,72	**72,11**	P07900;Q14568;Q58FG(	**Heat shock protein HSP 90-alpha**	HSP90AA1
0,042	-0,47	0,72	**72,16**	Q9NUQ8	**ATP-binding cassette sub-family F member 3**	ABCF3
0,049	-0,47	0,72	**72,26**	P40818	**Ubiquitin carboxyl-terminal hydrolase 8**	USP8
0,019	-0,47	0,72	**72,27**	P28838	**Cytosol aminopeptidase**	LAP3
0,036	-0,47	0,72	**72,28**	P62937;F5H284;Q9Y536	**Peptidyl-prolyl cis-trans isomerase A**	PPIA
0,044	-0,47	0,72	**72,29**	P08758	**Annexin A5**	ANXA5
0,005	-0,47	0,72	**72,36**	O60318	**80 kDa MCM3-associated protein**	MCM3AP
0,030	-0,47	0,72	**72,36**	O75153	**Protein KIAA0664**	KIAA0664
0,034	-0,47	0,72	**72,38**	O95295	**SNARE-associated protein Snapin**	SNAPIN
0,005	-0,46	0,72	**72,49**	P55263	**Adenosine kinase**	ADK
0,001	-0,46	0,73	**72,52**	Q99460	**26S proteasome non-ATPase regulatory subunit 1**	PSMD1
0,005	-0,46	0,73	**72,62**	Q15042	**Rab3 GTPase-activating protein catalytic subunit**	RAB3GAP1
0,002	-0,46	0,73	**72,64**	P50995	**Annexin A11**	ANXA11
0,024	-0,46	0,73	**72,69**	Q99733	**Nucleosome assembly protein 1-like 4**	NAP1L4
0,006	-0,46	0,73	**72,69**	Q96JJ7	**Protein disulfide-isomerase TMX3**	TMX3
0,003	-0,46	0,73	**72,70**	P40123	**Adenylyl cyclase-associated protein 2**	CAP2
0,013	-0,46	0,73	**72,71**	Q15084	**Protein disulfide-isomerase A6**	PDIA6
0,002	-0,46	0,73	**72,75**	Q13085	**Acetyl-CoA carboxylase 1;Biotin carboxylase**	ACACA
0,007	-0,46	0,73	**72,79**	P23526	**Adenosylhomocysteinase**	AHCY
0,034	-0,46	0,73	**72,87**	Q9BRA2	**Thioredoxin domain-containing protein 17**	TXNDC17
0,003	-0,46	0,73	**72,88**	Q99614	**Tetratricopeptide repeat protein 1**	TTC1
0,008	-0,45	0,73	**72,96**	Q6DKJ4	**Nucleoredoxin**	NXN
0,022	-0,45	0,73	**73,01**	P48426	**Phosphatidylinositol 5-phosphate 4-kinase type-2 alpha**	PIP4K2A
0,027	-0,45	0,73	**73,02**	Q99426	**Tubulin-folding cofactor B**	TBCB
0,038	-0,45	0,73	**73,12**	P38117	**Electron transfer flavoprotein subunit beta**	ETFB
0,022	-0,45	0,73	**73,14**	O95831	**Apoptosis-inducing factor 1, mitochondrial**	AIFM1
0,021	-0,45	0,73	**73,16**	Q9Y371	**Endophilin-B1**	SH3GLB1

0,000	-0,45	0,73	**73,21**	Q8TCS8	**Polyribonucleotide nucleotidyltransferase 1, mitochondrial**	PNPT1
0,001	-0,45	0,73	**73,32**	Q92616	**Translational activator GCN1**	GCN1L1
0,007	-0,45	0,73	**73,33**	Q9H6T3	**RNA polymerase II-associated protein 3**	RPAP3
0,005	-0,44	0,73	**73,47**	P30041	**Peroxiredoxin-6**	PRDX6
0,013	-0,44	0,74	**73,53**	Q9Y3A5	**Ribosome maturation protein SBDS**	SBDS
0,000	-0,44	0,74	**73,54**	Q9UPN7	**Serine/threonine-protein phosphatase 6 regulatory subunit 1**	PPP6R1
0,020	-0,44	0,74	**73,56**	Q9HAV7	**GrpE protein homolog 1, mitochondrial**	GRPEL1
0,027	-0,44	0,74	**73,64**	Q9HB71	**Calcyclin-binding protein**	CACYBP
0,000	-0,44	0,74	**73,65**	Q06124	**Tyrosine-protein phosphatase non-receptor type 11**	PTPN11
0,005	-0,44	0,74	**73,69**	P42224	**Signal transducer and activator of transcription 1-alpha/beta**	STAT1
0,003	-0,44	0,74	**73,81**	P43034	**Platelet-activating factor acetylhydrolase IB subunit alpha**	PAFAH1B1
0,045	-0,44	0,74	**73,86**	Q9UPT5	**Exocyst complex component 7**	EXOC7
0,016	-0,44	0,74	**73,89**	Q6UB35	**Monofunctional C1-tetrahydrofolate synthase, mitochondrial**	MTHFD1L
0,045	-0,43	0,74	**73,97**	O00273	**DNA fragmentation factor subunit alpha**	DFFA
0,005	-0,43	0,74	**73,98**	O00764	**Pyridoxal kinase**	PDXK
0,043	-0,43	0,74	**73,99**	O94826	**Mitochondrial import receptor subunit TOM70**	TOMM70A
0,000	-0,43	0,74	**73,99**	P62826	**GTP-binding nuclear protein Ran**	RAN
0,007	-0,43	0,74	**74,01**	Q9UK59	**Lariat debranching enzyme**	DBR1
0,043	-0,43	0,74	**74,03**	Q9UBQ0	**Vacuolar protein sorting-associated protein 29**	VPS29
0,048	-0,43	0,74	**74,17**	P30626	**Sorcin**	SRI
0,015	-0,43	0,74	**74,18**	P41250	**Glycine--tRNA ligase**	GARS
0,033	-0,43	0,74	**74,18**	P50453	**Serpin B9**	SERPINB9
0,008	-0,43	0,74	**74,19**	P49321	**Nuclear autoantigenic sperm protein**	NASP
0,016	-0,43	0,74	**74,22**	Q9UKY7	**Protein CDV3 homolog**	CDV3
0,028	-0,43	0,74	**74,24**	Q16401	**26S proteasome non-ATPase regulatory subunit 5**	PSMD5
0,036	-0,43	0,74	**74,27**	O95816	**BAG family molecular chaperone regulator 2**	BAG2
0,012	-0,43	0,74	**74,30**	O43399	**Tumor protein D54**	TPD52L2
0,000	-0,43	0,74	**74,32**	Q8WUM4	**Programmed cell death 6-interacting protein**	PDCD6IP
0,026	-0,43	0,74	**74,33**	O00541	**Pescadillo homolog**	PES1
0,005	-0,43	0,74	**74,35**	O95202	**LETM1 and EF-hand domain-containing protein 1, mitochondrial**	LETM1
0,039	-0,43	0,74	**74,36**	P55036	**26S proteasome non-ATPase regulatory subunit 4**	PSMD4
0,011	-0,43	0,74	**74,36**	Q9H936;Q9H1K4	**Mitochondrial glutamate carrier 1**	SLC25A22
0,037	-0,43	0,74	**74,38**	P32321	**Deoxycytidylate deaminase**	DCTD

0,004	-0,43	0,74	**74,39**	Q01581	**Hydroxymethylglutaryl-CoA synthase, cytoplasmic**	HMGCS1
0,003	-0,42	0,74	**74,50**	O00330	**Pyruvate dehydrogenase protein X component, mitochondrial**	PDHX
0,002	-0,42	0,75	**74,55**	P31153;Q00266	**S-adenosylmethionine synthase isoform type-2**	MAT2A
0,020	-0,42	0,75	**74,60**	P84090	**Enhancer of rudimentary homolog**	ERH
0,015	-0,42	0,75	**74,62**	P30740	**Leukocyte elastase inhibitor**	SERPINB1
0,039	-0,42	0,75	**74,63**	P21291	**Cysteine and glycine-rich protein 1**	CSRP1
0,033	-0,42	0,75	**74,68**	Q9Y223	**Bifunctional UDP-N-acetylglucosamine 2-epimerase/N-acetylmannosamine kinase;U**	GNE
0,023	-0,42	0,75	**74,69**	P23921	**Ribonucleoside-diphosphate reductase large subunit**	RRM1
0,017	-0,42	0,75	**74,71**	P38646	**Stress-70 protein, mitochondrial**	HSPA9
0,005	-0,42	0,75	**74,91**	Q8TD19	**Serine/threonine-protein kinase Nek9**	NEK9
0,049	-0,42	0,75	**74,95**	Q8NFQ8	**Torsin-1A-interacting protein 2**	TOR1AIP2
0,000	-0,42	0,75	**74,99**	Q8IVF2	**Protein AHNAK2**	AHNAK2
0,002	-0,41	0,75	**75,01**	Q9UM54	**Unconventional myosin-VI**	MYO6
0,026	-0,41	0,75	**75,06**	P18031	**Tyrosine-protein phosphatase non-receptor type 1**	PTPN1
0,008	-0,41	0,75	**75,07**	O95487	**Protein transport protein Sec24B**	SEC24B
0,005	-0,41	0,75	**75,08**	Q13616	**Cullin-1**	CUL1
0,031	-0,41	0,75	**75,12**	Q9H9P8	**L-2-hydroxyglutarate dehydrogenase, mitochondrial**	L2HGDH
0,050	-0,41	0,75	**75,14**	P11233	**Ras-related protein Ral-A**	RALA
0,020	-0,41	0,75	**75,14**	Q96EL3	**39S ribosomal protein L53, mitochondrial**	MRPL53
0,016	-0,41	0,75	**75,18**	Q96JB5	**CDK5 regulatory subunit-associated protein 3**	CDK5RAP3
0,007	-0,41	0,75	**75,27**	Q13464	**Rho-associated protein kinase 1**	ROCK1
0,001	-0,41	0,75	**75,29**	Q13177	**Serine/threonine-protein kinase PAK 2;PAK-2p27;PAK-2p34**	PAK2
0,042	-0,41	0,75	**75,29**	P46459	**Vesicle-fusing ATPase**	NSF
0,021	-0,41	0,75	**75,32**	Q9P2J5	**Leucine--tRNA ligase, cytoplasmic**	LARS
0,050	-0,41	0,75	**75,32**	O75828	**Carbonyl reductase [NADPH] 3**	CBR3
0,002	-0,41	0,75	**75,34**	P13489	**Ribonuclease inhibitor**	RNH1
0,000	-0,41	0,75	**75,36**	P20073	**Annexin A7**	ANXA7
0,006	-0,41	0,75	**75,41**	O00429	**Dynamin-1-like protein**	DNM1L
0,030	-0,41	0,76	**75,51**	Q13057	**Bifunctional coenzyme A synthase;Phosphopantetheine adenylyltransferase;Dephos**	COASY
0,024	-0,41	0,76	**75,52**	P61224;A6NIZ1	**Ras-related protein Rap-1b;Ras-related protein Rap-1b-like protein**	RAP1B
0,032	-0,40	0,76	**75,73**	Q15942	**Zyxin**	ZYX
0,035	-0,40	0,76	**75,83**	P10768	**S-formylglutathione hydrolase**	ESD
0,004	-0,40	0,76	**75,88**	Q08257	**Quinone oxidoreductase**	CRYZ

0,001	-0,40	0,76	**75,96**	Q13045	**Protein flightless-1 homolog**	FLII
0,006	-0,39	0,76	**76,09**	P60866	**40S ribosomal protein S20**	RPS20
0,007	-0,39	0,76	**76,17**	P40763	**Signal transducer and activator of transcription 3**	STAT3
0,017	-0,39	0,76	**76,21**	O15212	**Prefoldin subunit 6**	PFDN6
0,006	-0,39	0,76	**76,23**	Q12931	**Heat shock protein 75 kDa, mitochondrial**	TRAP1
0,021	-0,39	0,76	**76,28**	P46940	**Ras GTPase-activating-like protein IQGAP1**	IQGAP1
0,003	-0,39	0,76	**76,28**	P30622	**CAP-Gly domain-containing linker protein 1**	CLIP1
0,024	-0,39	0,76	**76,39**	P14550	**Alcohol dehydrogenase [NADP(+)]**	AKR1A1
0,001	-0,39	0,76	**76,40**	O43847	**Nardilysin**	NRD1
0,009	-0,39	0,76	**76,41**	P13010	**X-ray repair cross-complementing protein 5**	XRCC5
0,012	-0,39	0,76	**76,44**	Q92598	**Heat shock protein 105 kDa**	HSPH1
0,042	-0,39	0,76	**76,45**	O75569	**Interferon-inducible double stranded RNA-dependent protein kinase activator A**	PRKRA
0,002	-0,39	0,76	**76,48**	P49589	**Cysteine--tRNA ligase, cytoplasmic**	CARS
0,005	-0,39	0,76	**76,49**	P58546	**Myotrophin**	MTPN
0,001	-0,39	0,77	**76,50**	Q9UBC2	**Epidermal growth factor receptor substrate 15-like 1**	EPS15L1
0,002	-0,39	0,77	**76,50**	O75822	**Eukaryotic translation initiation factor 3 subunit J**	EIF3J
0,031	-0,39	0,77	**76,51**	P24666	**Low molecular weight phosphotyrosine protein phosphatase**	ACP1
0,010	-0,38	0,77	**76,67**	Q9NZL9	**Methionine adenosyltransferase 2 subunit beta**	MAT2B
0,020	-0,38	0,77	**76,67**	P05556	**Integrin beta-1**	ITGB1
0,018	-0,38	0,77	**76,69**	P67809	**Nuclease-sensitive element-binding protein 1**	YBX1
0,038	-0,38	0,77	**76,73**	O75439	**Mitochondrial-processing peptidase subunit beta**	PMPCB
0,001	-0,37	0,77	**77,21**	P31939	**Bifunctional purine biosynthesis protein PURH;Phosphoribosylaminoimidazolecarbo**	ATIC
0,034	-0,37	0,77	**77,24**	P50570;Q9UQ16;Q0519	**Dynamin-2**	DNM2
0,004	-0,37	0,77	**77,34**	Q4G0N4	**NAD kinase domain-containing protein 1**	NADKD1
0,002	-0,37	0,77	**77,34**	P07355;A6NMY6	**Annexin A2;Putative annexin A2-like protein**	ANXA2;ANXA2P2
0,018	-0,37	0,77	**77,37**	P34897	**Serine hydroxymethyltransferase, mitochondrial**	SHMT2
0,007	-0,37	0,77	**77,45**	Q7L523;Q5VZM2	**Ras-related GTP-binding protein A;Ras-related GTP-binding protein B**	RRAGA;RRAGB
0,004	-0,37	0,77	**77,46**	P60660	**Myosin light polypeptide 6**	MYL6
0,038	-0,37	0,78	**77,54**	P12956	**X-ray repair cross-complementing protein 6**	XRCC6
0,047	-0,37	0,78	**77,54**	P54652	**Heat shock-related 70 kDa protein 2**	HSPA2
0,030	-0,37	0,78	**77,64**	Q9Y4E8	**Ubiquitin carboxyl-terminal hydrolase 15**	USP15
0,002	-0,36	0,78	**77,77**	Q9H2G2	**STE20-like serine/threonine-protein kinase**	SLK
0,000	-0,36	0,78	**77,79**	P56537	**Eukaryotic translation initiation factor 6**	EIF6

0,019	-0,36	0,78	**77,81**	P50454	**Serpin H1**	SERPINH1
0,026	-0,36	0,78	**77,93**	P13639	**Elongation factor 2**	EEF2
0,030	-0,36	0,78	**77,96**	O43143	**Putative pre-mRNA-splicing factor ATP-dependent RNA helicase DHX15**	DHX15
0,016	-0,36	0,78	**78,00**	Q09666	**Neuroblast differentiation-associated protein AHNAK**	AHNAK
0,028	-0,36	0,78	**78,00**	Q6FI81	**Anamorsin**	CIAPIN1
0,001	-0,36	0,78	**78,01**	P04632;Q96L46	**Calpain small subunit 1**	CAPNS1
0,032	-0,35	0,78	**78,24**	O94979	**Protein transport protein Sec31A**	SEC31A
0,032	-0,35	0,78	**78,47**	Q9UKK3	**Poly [ADP-ribose] polymerase 4**	PARP4
0,024	-0,35	0,78	**78,47**	P34932	**Heat shock 70 kDa protein 4**	HSPA4
0,025	-0,35	0,79	**78,50**	Q12965;O00160	**Unconventional myosin-Ie**	MYO1E
0,012	-0,35	0,79	**78,55**	O14745	**Na(+)/H(+) exchange regulatory cofactor NHE-RF1**	SLC9A3R1
0,038	-0,35	0,79	**78,66**	P08107	**Heat shock 70 kDa protein 1A/1B**	HSPA1A
0,023	-0,34	0,79	**78,77**	P07737;CON__P02584	**Profilin-1**	PFN1
0,005	-0,34	0,79	**78,80**	131;Q9UBL6;O95741;Q9	**Copine-3**	CPNE3
0,041	-0,34	0,79	**78,85**	P36957	**Dihydrolipoyllysine-residue succinyltransferase component of 2-oxoglutarate dehyd**	DLST
0,007	-0,34	0,79	**78,86**	Q9NY33	**Dipeptidyl peptidase 3**	DPP3
0,017	-0,34	0,79	**78,89**	P29401	**Transketolase**	TKT
0,004	-0,34	0,79	**78,95**	Q9Y2Z0	**Suppressor of G2 allele of SKP1 homolog**	SUGT1
0,015	-0,34	0,79	**79,17**	P23381	**Tryptophan--tRNA ligase, cytoplasmic;T1-TrpRS;T2-TrpRS**	WARS
0,005	-0,34	0,79	**79,17**	O60749	**Sorting nexin-2**	SNX2
0,042	-0,34	0,79	**79,25**	P55327	**Tumor protein D52**	TPD52
0,021	-0,34	0,79	**79,25**	O94903	**Proline synthase co-transcribed bacterial homolog protein**	PROSC
0,039	-0,33	0,79	**79,29**	O00231	**26S proteasome non-ATPase regulatory subunit 11**	PSMD11
0,010	-0,33	0,79	**79,30**	Q9Y617	**Phosphoserine aminotransferase**	PSAT1
0,000	-0,33	0,79	**79,32**	P54577	**Tyrosine--tRNA ligase, cytoplasmic**	YARS
0,035	-0,33	0,79	**79,39**	O75351	**Vacuolar protein sorting-associated protein 4B**	VPS4B
0,019	-0,33	0,79	**79,41**	Q9NPQ8	**Synembryn-A**	RIC8A
0,002	-0,33	0,79	**79,42**	P13611	**Versican core protein**	VCAN
0,016	-0,33	0,79	**79,44**	Q6PL18	**ATPase family AAA domain-containing protein 2**	ATAD2
0,013	-0,33	0,80	**79,51**	O15270	**Serine palmitoyltransferase 2**	SPTLC2
0,038	-0,33	0,80	**79,56**	P53597	**Succinyl-CoA ligase [ADP/GDP-forming] subunit alpha, mitochondrial**	SUCLG1
0,006	-0,33	0,80	**79,57**	Q13586	**Stromal interaction molecule 1**	STIM1
0,003	-0,33	0,80	**79,60**	Q06787	**Fragile X mental retardation protein 1**	FMR1

0,042	-0,33	0,80	**79,69**	P43897	**Elongation factor Ts, mitochondrial**	TSFM
0,016	-0,33	0,80	**79,71**	P49137;P51817;O43930	**MAP kinase-activated protein kinase 3;MAP kinase-activated protein kinase 2**	MAPKAPK3;MAPKA
0,037	-0,33	0,80	**79,72**	P12277	**Creatine kinase B-type**	CKB
0,024	-0,33	0,80	**79,78**	Q9H4A4	**Aminopeptidase B**	RNPEP
0,016	-0,32	0,80	**79,88**	Q96JH7	**Deubiquitinating protein VCIP135**	VCPIP1
0,004	-0,32	0,80	**79,91**	P53992	**Protein transport protein Sec24C**	SEC24C
0,000	-0,32	0,80	**79,91**	P17987	**T-complex protein 1 subunit alpha**	TCP1
0,020	-0,32	0,80	**79,99**	P13797;P13796	**Plastin-3**	PLS3
0,004	-0,32	0,80	**80,00**	O75116	**Rho-associated protein kinase 2**	ROCK2
0,029	-0,32	0,80	**80,03**	O14818;Q8TAA3	**Proteasome subunit alpha type-7**	PSMA7
0,028	-0,32	0,80	**80,08**	P22059	**Oxysterol-binding protein 1**	OSBP
0,036	-0,32	0,80	**80,16**	Q63ZY3;Q14678	**KN motif and ankyrin repeat domain-containing protein 2**	KANK2
0,045	-0,32	0,80	**80,16**	P45984;P53779;P45983	**Mitogen-activated protein kinase 9**	MAPK9
0,033	-0,32	0,80	**80,31**	Q16513	**Serine/threonine-protein kinase N2**	PKN2
0,023	-0,31	0,80	**80,41**	Q9H2M9	**Rab3 GTPase-activating protein non-catalytic subunit**	RAB3GAP2
0,009	-0,31	0,80	**80,42**	P04083	**Annexin A1**	ANXA1
0,000	-0,31	0,80	**80,44**	O43765	**Small glutamine-rich tetratricopeptide repeat-containing protein alpha**	SGTA
0,014	-0,31	0,81	**80,53**	P10809	**60 kDa heat shock protein, mitochondrial**	HSPD1
0,016	-0,31	0,81	**80,61**	P52888	**Thimet oligopeptidase**	THOP1
0,002	-0,31	0,81	**80,61**	P04040	**Catalase**	CAT
0,025	-0,31	0,81	**80,64**	Q9Y5K5	**Ubiquitin carboxyl-terminal hydrolase isozyme L5**	UCHL5
0,048	-0,31	0,81	**80,68**	Q9BSJ8	**Extended synaptotagmin-1**	ESYT1
0,050	-0,31	0,81	**80,80**	P07902	**Galactose-1-phosphate uridylyltransferase**	GALT
0,033	-0,31	0,81	**80,83**	O15027	**Protein transport protein Sec16A**	SEC16A
0,027	-0,31	0,81	**80,89**	P27708;P31327	**CAD protein;Glutamine-dependent carbamoyl-phosphate synthase;Aspartate carba**	CAD
0,043	-0,31	0,81	**80,94**	P17655	**Calpain-2 catalytic subunit**	CAPN2
0,030	-0,30	0,81	**80,98**	Q9Y376	**Calcium-binding protein 39**	CAB39
0,016	-0,30	0,81	**81,14**	P40227	**T-complex protein 1 subunit zeta**	CCT6A
0,035	-0,30	0,81	**81,20**	Q14738	**Serine/threonine-protein phosphatase 2A 56 kDa regulatory subunit delta isoform**	PPP2R5D
0,006	-0,30	0,81	**81,22**	O43852	**Calumenin**	CALU
0,022	-0,30	0,81	**81,29**	P35250	**Replication factor C subunit 2**	RFC2
0,008	-0,30	0,81	**81,30**	P26038	**Moesin**	MSN
0,048	-0,30	0,81	**81,34**	P42566	**Epidermal growth factor receptor substrate 15**	EPS15

0,005	-0,30	0,81	**81,36**	P52758	**Ribonuclease UK114**	HRSP12
0,009	-0,30	0,81	**81,44**	Q15181	**Inorganic pyrophosphatase**	PPA1
0,003	-0,30	0,81	**81,46**	Q96C90	**Protein phosphatase 1 regulatory subunit 14B**	PPP1R14B
0,023	-0,30	0,81	**81,50**	P29144	**Tripeptidyl-peptidase 2**	TPP2
0,016	-0,29	0,82	**81,53**	P49257	**Protein ERGIC-53**	LMAN1
0,000	-0,29	0,82	**81,57**	P11498	**Pyruvate carboxylase, mitochondrial**	PC
0,036	-0,29	0,82	**81,57**	O00592	**Podocalyxin**	PODXL
0,015	-0,29	0,82	**81,61**	Q5T4S7	**E3 ubiquitin-protein ligase UBR4**	UBR4
0,015	-0,29	0,82	**81,77**	P55145	**Mesencephalic astrocyte-derived neurotrophic factor**	MANF
0,040	-0,29	0,82	**81,83**	Q13131	**5-AMP-activated protein kinase catalytic subunit alpha-1**	PRKAA1
0,030	-0,29	0,82	**81,84**	Q9BYT8	**Neurolysin, mitochondrial**	NLN
0,001	-0,29	0,82	**81,88**	Q13907	**Isopentenyl-diphosphate Delta-isomerase 1**	IDI1
0,028	-0,28	0,82	**82,21**	P11216	**Glycogen phosphorylase, brain form**	PYGB
0,014	-0,28	0,82	**82,29**	P49748	**Very long-chain specific acyl-CoA dehydrogenase, mitochondrial**	ACADVL
0,004	-0,28	0,82	**82,36**	Q9HC35	**Echinoderm microtubule-associated protein-like 4**	EML4
0,001	-0,28	0,82	**82,37**	Q9UL46	**Proteasome activator complex subunit 2**	PSME2
0,018	-0,28	0,82	**82,39**	P62873	**Guanine nucleotide-binding protein G(I)/G(S)/G(T) subunit beta-1**	GNB1
0,002	-0,28	0,82	**82,46**	Q13200	**26S proteasome non-ATPase regulatory subunit 2**	PSMD2
0,039	-0,28	0,82	**82,47**	Q9H2U2	**Inorganic pyrophosphatase 2, mitochondrial**	PPA2
0,016	-0,27	0,83	**82,68**	P48556	**26S proteasome non-ATPase regulatory subunit 8**	PSMD8
0,005	-0,27	0,83	**82,70**	Q07960	**Rho GTPase-activating protein 1**	ARHGAP1
0,011	-0,27	0,83	**82,76**	O75886	**Signal transducing adapter molecule 2**	STAM2
0,041	-0,27	0,83	**82,78**	P08134;P62745	**Rho-related GTP-binding protein RhoC**	RHOC
0,045	-0,27	0,83	**82,94**	P60981	**Destrin**	DSTN
0,012	-0,27	0,83	**82,99**	Q9HCC0	**Methylcrotonoyl-CoA carboxylase beta chain, mitochondrial**	MCCC2
0,035	-0,27	0,83	**83,03**	O60841	**Eukaryotic translation initiation factor 5B**	EIF5B
0,025	-0,27	0,83	**83,12**	Q9UHG3	**Prenylcysteine oxidase 1**	PCYOX1
0,003	-0,26	0,83	**83,41**	A0AVT1	**Ubiquitin-like modifier-activating enzyme 6**	UBA6
0,010	-0,26	0,83	**83,44**	Q13938	**Calcyphosin**	CAPS
0,045	-0,26	0,83	**83,45**	P48643	**T-complex protein 1 subunit epsilon**	CCT5
0,041	-0,26	0,84	**83,75**	P56134	**ATP synthase subunit f, mitochondrial**	ATP5J2
0,046	-0,25	0,84	**83,99**	P23284	**Peptidyl-prolyl cis-trans isomerase B**	PPIB
0,006	-0,24	0,84	**84,39**	O60716	**Catenin delta-1**	CTNND1

0,021	-0,24	0,84	**84,41**	979;P0CG48;P0CG47;P62	**Ubiquitin-40S ribosomal protein S27a;Ubiquitin;40S ribosomal protein S27a;Polyubi**	RPS27A;UBC;UBB;U
0,003	-0,24	0,85	**84,82**	P41091;Q2VIR3	**Eukaryotic translation initiation factor 2 subunit 3;Putative eukaryotic translation in**	EIF2S3;EIF2S3L
0,006	-0,24	0,85	**84,86**	P36776	**Lon protease homolog, mitochondrial**	LONP1
0,001	-0,24	0,85	**84,88**	P21333	**Filamin-A**	FLNA
0,032	-0,24	0,85	**84,95**	Q12792	**Twinfilin-1**	TWF1
0,049	-0,23	0,85	**85,12**	P53990	**IST1 homolog**	IST1
0,035	-0,23	0,85	**85,41**	Q8TAT6	**Nuclear protein localization protein 4 homolog**	NPLOC4
0,014	-0,23	0,85	**85,41**	P49411	**Elongation factor Tu, mitochondrial**	TUFM
0,002	-0,23	0,85	**85,42**	P33121;Q9ULC5	**Long-chain-fatty-acid--CoA ligase 1**	ACSL1
0,041	-0,23	0,86	**85,56**	P41240	**Tyrosine-protein kinase CSK**	CSK
0,039	-0,22	0,86	**85,56**	Q9UNM6	**26S proteasome non-ATPase regulatory subunit 13**	PSMD13
0,001	-0,22	0,86	**86,02**	O75179	**Ankyrin repeat domain-containing protein 17**	ANKRD17
0,036	-0,20	0,87	**86,83**	P16930	**Fumarylacetoacetase**	FAH
0,038	-0,20	0,87	**87,13**	P09382	**Galectin-1**	LGALS1
0,019	-0,19	0,87	**87,38**	Q14444	**Caprin-1**	CAPRIN1
0,018	-0,19	0,87	**87,41**	Q9Y5K6	**CD2-associated protein**	CD2AP
0,045	-0,19	0,88	**87,55**	Q96C19;Q9BUP0	**EF-hand domain-containing protein D2**	EFHD2
0,018	-0,18	0,88	**88,37**	Q99615	**DnaJ homolog subfamily C member 7**	DNAJC7
0,020	-0,17	0,89	**88,65**	P55786;A6NEC2	**Puromycin-sensitive aminopeptidase**	NPEPPS
0,050	-0,16	0,89	**89,23**	P51808	**Dynein light chain Tctex-type 3**	DYNLT3

Color Code

8h Up
24h Up
8h Down
24h Down

Suppemental Table S2b: Shotgun proteomic analysis of OVCAR3 cells exposed for 8h and 24h to 40µM G28UCM. All proteins identified by MS/MS.

p-Value	x	2^x	% of Control	Protein IDs	Protein Names	Gene names
**8 h Up**						
0,017	5,75	53,86	**5386,33**	P33527	**Multidrug resistance-associated protein 1**	ABCC1
0,000	4,73	26,50	**2650,38**	Q9HBL0	**Tensin-1**	TNS1
0,005	4,48	22,36	**2236,48**	O00479	**High mobility group nucleosome-binding domain-containing protein 4**	HMGN4
0,014	4,13	17,51	**1750,87**	Q96S55	**ATPase WRNIP1**	WRNIP1
0,014	4,11	17,26	**1725,54**	Q9UDY4	**DnaJ homolog subfamily B member 4**	DNAJB4
0,000	3,99	15,89	**1588,95**	Q9UMS0	**NFU1 iron-sulfur cluster scaffold homolog, mitochondrial**	NFU1
0,020	3,86	14,55	**1455,00**	Q6PL18	**ATPase family AAA domain-containing protein 2**	ATAD2
0,006	3,78	13,71	**1371,02**	O00584	**Ribonuclease T2**	RNASET2
0,017	3,77	13,65	**1365,35**	Q5VWQ8	**Disabled homolog 2-interacting protein**	DAB2IP
0,000	3,76	13,52	**1351,57**	P11166	**Solute carrier family 2, facilitated glucose transporter member 1**	SLC2A1
0,032	3,62	12,33	**1233,50**	O75448	**Mediator of RNA polymerase II transcription subunit 24**	MED24
0,034	3,57	11,88	**1187,62**	Q96DH6	**RNA-binding protein Musashi homolog 2**	MSI2
0,001	3,57	11,84	**1183,64**	Q9GZT9	**Egl nine homolog 1**	EGLN1
0,002	3,54	11,63	**1163,18**	Q16665	**Hypoxia-inducible factor 1-alpha**	HIF1A
0,019	3,52	11,51	**1150,86**	Q8TEA8	**D-tyrosyl-tRNA(Tyr) deacylase 1**	DTD1
0,005	3,49	11,27	**1126,52**	Q9BZV1	**UBX domain-containing protein 6**	UBXN6
0,005	3,48	11,16	**1115,79**	Q13423	**NAD(P) transhydrogenase, mitochondrial**	NNT
0,001	3,48	11,16	**1115,79**	P17693	**HLA class I histocompatibility antigen, alpha chain G**	HLA-G
0,027	3,42	10,67	**1067,47**	Q70UQ0	**Inhibitor of nuclear factor kappa-B kinase-interacting protein**	IKBIP
0,026	3,35	10,21	**1021,05**	P05976;P08590	**Myosin light chain 1/3, skeletal muscle isoform;Myosin light chain 3**	MYL1;MYL3
0,012	3,33	10,06	**1005,61**	Q6ZSR9	**Uncharacterized protein FLJ45252**	
0,035	3,30	9,88	**987,62**	O95208	**Epsin-2**	EPN2
0,000	3,30	9,84	**984,44**	Q8NCH0	**Carbohydrate sulfotransferase 14**	CHST14
0,004	3,27	9,65	**964,65**	Q3YBR2	**Transforming growth factor beta regulator 1**	TBRG1
0,020	3,23	9,41	**941,18**	Q15434	**RNA-binding motif, single-stranded-interacting protein 2**	RBMS2
0,006	3,21	9,23	**922,97**	Q14493	**Histone RNA hairpin-binding protein**	SLBP
0,012	3,14	8,82	**881,52**	P45985	**Dual specificity mitogen-activated protein kinase kinase 4**	MAP2K4

0,003	3,12	8,69	**868,70**	Q96CU9	**FAD-dependent oxidoreductase domain-containing protein 1**	FOXRED1
0,002	3,10	8,59	**858,80**	Q99996	**A-kinase anchor protein 9**	AKAP9
0,000	3,06	8,34	**834,36**	Q9UPQ3	**Arf-GAP with GTPase, ANK repeat and PH domain-containing protein 1**	AGAP1
0,027	3,04	8,25	**824,52**	O14907	**Tax1-binding protein 3**	TAX1BP3
0,000	3,03	8,17	**816,82**	O76041	**Nebulette**	NEBL
0,005	2,99	7,96	**795,52**	Q9UKM9	**RNA-binding protein Raly**	RALY
0,024	2,89	7,41	**741,34**	P53803	**DNA-directed RNA polymerases I, II, and III subunit RPABC4**	POLR2K
0,030	2,84	7,15	**715,20**	Q5VZF2	**Muscleblind-like protein 2**	MBNL2
0,005	2,82	7,06	**706,16**	Q8IWB7	**WD repeat and FYVE domain-containing protein 1**	WDFY1
0,001	2,81	7,01	**701,28**	Q9H936	**Mitochondrial glutamate carrier 1**	SLC25A22
0,029	2,79	6,90	**689,75**	Q9C0D4	**Zinc finger protein 518B**	ZNF518B
0,012	2,73	6,63	**662,74**	O14656	**Torsin-1A**	TOR1A
0,019	2,66	6,32	**632,03**	Q9BRQ6	**MICOS complex subunit MIC25**	CHCHD6
0,001	2,65	6,28	**627,67**	P06703	**Protein S100-A6**	S100A6
0,040	2,63	6,19	**619,03**	Q4G0P3	**Hydrocephalus-inducing protein homolog**	HYDIN
0,014	2,57	5,94	**594,06**	P06400	**Retinoblastoma-associated protein**	RB1
0,005	2,57	5,94	**593,64**	P17050	**Alpha-N-acetylgalactosaminidase**	NAGA
0,037	2,55	5,86	**586,07**	Q9BXW7	**Cat eye syndrome critical region protein 5**	CECR5
0,003	2,55	5,86	**585,63**	Q9UEU0	**Vesicle transport through interaction with t-SNAREs homolog 1B**	VTI1B
0,008	2,51	5,68	**567,74**	Q9P0U4	**CXXC-type zinc finger protein 1**	CXXC1
0,044	2,50	5,67	**566,75**	Q15652	**Probable JmjC domain-containing histone demethylation protein 2C**	JMJD1C
0,039	2,49	5,62	**562,28**	P49356	**Protein farnesyltransferase subunit beta**	FNTB
0,006	2,48	5,60	**559,76**	Q9BRT6	**Protein LLP homolog**	LLPH
0,004	2,48	5,58	**557,90**	Q9BRZ2	**E3 ubiquitin-protein ligase TRIM56**	TRIM56
0,002	2,47	5,56	**555,79**	P08621	**U1 small nuclear ribonucleoprotein 70 kDa**	SNRNP70
0,016	2,47	5,52	**552,24**	Q86WX3	**Active regulator of SIRT1**	RPS19BP1
0,000	2,45	5,46	**546,42**	O95498	**Vascular non-inflammatory molecule 2**	VNN2
0,049	2,43	5,37	**537,07**	Q9Y295	**Developmentally-regulated GTP-binding protein 1**	DRG1
0,041	2,42	5,35	**535,17**	Q9P2K3	**REST corepressor 3**	RCOR3
0,016	2,41	5,33	**532,70**	P42345	**Serine/threonine-protein kinase mTOR**	MTOR
0,011	2,41	5,31	**531,47**	Q9Y421	**Protein FAM32A**	FAM32A
0,039	2,41	5,30	**530,11**	O00411	**DNA-directed RNA polymerase, mitochondrial**	POLRMT
0,038	2,40	5,28	**527,80**	Q53HC9	**Protein TSSC1**	TSSC1

0,026	2,38	5,21	**520,54**	Q99439	**Calponin-2**	CNN2
0,016	2,37	5,17	**516,94**	Q13796	**Protein Shroom2**	SHROOM2
0,038	2,37	5,17	**516,94**	Q4J6C6	**Prolyl endopeptidase-like**	PREPL
0,046	2,36	5,13	**513,08**	P09758	**Tumor-associated calcium signal transducer 2**	TACSTD2
0,050	2,30	4,94	**493,99**	Q9H3P2	**Negative elongation factor A**	NELFA
0,047	2,30	4,92	**492,46**	Q9UDT6	**CAP-Gly domain-containing linker protein 2**	CLIP2
0,029	2,30	4,92	**491,64**	Q9NY61	**Protein AATF**	AATF
0,003	2,29	4,89	**489,06**	Q5W111	**SPRY domain-containing protein 7**	SPRYD7
0,001	2,27	4,83	**483,47**	Q14676	**Mediator of DNA damage checkpoint protein 1**	MDC1
0,010	2,27	4,82	**482,32**	O75164	**Lysine-specific demethylase 4A**	KDM4A
0,019	2,26	4,79	**478,99**	P49593	**Protein phosphatase 1F**	PPM1F
0,030	2,24	4,72	**471,74**	Q9BYD1	**39S ribosomal protein L13, mitochondrial**	MRPL13
0,016	2,24	4,71	**471,22**	P04114	**Apolipoprotein B-100;Apolipoprotein B-48**	APOB
0,024	2,22	4,67	**466,68**	Q71F56	**Mediator of RNA polymerase II transcription subunit 13-like**	MED13L
0,016	2,18	4,54	**454,21**	Q9UJX4	**Anaphase-promoting complex subunit 5**	ANAPC5
0,009	2,18	4,53	**453,15**	Q4V328	**GRIP1-associated protein 1**	GRIPAP1
0,028	2,17	4,50	**450,02**	Q8N392	**Rho GTPase-activating protein 18**	ARHGAP18
0,017	2,15	4,44	**443,83**	P27540	**Aryl hydrocarbon receptor nuclear translocator**	ARNT
0,038	2,13	4,39	**439,23**	Q5RKV6	**Exosome complex component MTR3**	EXOSC6
0,009	2,12	4,34	**433,58**	Q96B26	**Exosome complex component RRP43**	EXOSC8
0,016	2,11	4,32	**431,69**	P42695	**Condensin-2 complex subunit D3**	NCAPD3
0,012	2,09	4,26	**425,75**	Q8WWQ0	**PH-interacting protein**	PHIP
0,008	2,09	4,24	**424,47**	Q9Y4P3	**Transducin beta-like protein 2**	TBL2
0,033	2,08	4,24	**423,82**	Q96S94	**Cyclin-L2**	CCNL2
0,013	2,08	4,22	**422,21**	Q96PK6	**RNA-binding protein 14**	RBM14
0,032	2,06	4,17	**416,63**	P17676	**CCAAT/enhancer-binding protein beta**	CEBPB
0,002	2,02	4,07	**406,53**	Q9UHR4	**Brain-specific angiogenesis inhibitor 1-associated protein 2-like protein 1**	BAIAP2L1
0,005	2,00	4,00	**400,29**	Q6P2Q9	**Pre-mRNA-processing-splicing factor 8**	PRPF8
0,005	1,98	3,96	**395,80**	P60602	**Reactive oxygen species modulator 1**	ROMO1
0,001	1,98	3,96	**395,57**	Q9BUQ8	**Probable ATP-dependent RNA helicase DDX23**	DDX23
0,032	1,98	3,95	**395,13**	Q9BQG0	**Myb-binding protein 1A**	MYBBP1A
0,025	1,98	3,94	**394,49**	Q13889	**General transcription factor IIH subunit 3**	GTF2H3
0,024	1,96	3,89	**389,06**	Q6MZP7	**Protein lin-54 homolog**	LIN54

0,010	1,95	3,87	**387,11**	Q9Y2D4	**Exocyst complex component 6B**	EXOC6B
0,009	1,95	3,86	**386,21**	Q16537	**Serine/threonine-protein phosphatase 2A 56 kDa regulatory subunit epsilon isoform**	PPP2R5E
0,002	1,93	3,81	**381,44**	P48637	**Glutathione synthetase**	GSS
0,019	1,92	3,78	**378,42**	Q86YS3	**Rab11 family-interacting protein 4**	RAB11FIP4
0,010	1,89	3,71	**370,57**	P27986	**Phosphatidylinositol 3-kinase regulatory subunit alpha**	PIK3R1
0,007	1,87	3,66	**365,53**	O00423	**Echinoderm microtubule-associated protein-like 1**	EML1
0,013	1,84	3,58	**358,01**	O00418	**Eukaryotic elongation factor 2 kinase**	EEF2K
0,025	1,83	3,54	**354,43**	Q9Y6R0	**Numb-like protein**	NUMBL
0,005	1,82	3,54	**353,66**	Q9H0S4	**Probable ATP-dependent RNA helicase DDX47**	DDX47
0,031	1,81	3,51	**350,88**	Q86W42	**THO complex subunit 6 homolog**	THOC6
0,028	1,81	3,51	**350,64**	Q92600	**Cell differentiation protein RCD1 homolog**	RQCD1
0,024	1,81	3,49	**349,43**	O95479	**GDH/6PGL endoplasmic bifunctional protein;Glucose 1-dehydrogenase;6-phosphogl**	H6PD
0,021	1,80	3,48	**348,22**	Q14119	**Vascular endothelial zinc finger 1**	VEZF1
0,013	1,80	3,48	**348,06**	O76021	**Ribosomal L1 domain-containing protein 1**	RSL1D1
0,007	1,79	3,45	**345,02**	P98175	**RNA-binding protein 10**	RBM10
0,001	1,78	3,44	**343,97**	Q13557	**Calcium/calmodulin-dependent protein kinase type II subunit delta**	CAMK2D
0,008	1,78	3,43	**343,50**	Q96L91	**E1A-binding protein p400**	EP400
0,042	1,78	3,43	**343,23**	P14902	**Indoleamine 2,3-dioxygenase 1**	IDO1
0,006	1,77	3,41	**341,37**	Q9NRS6	**Sorting nexin-15**	SNX15
0,003	1,77	3,41	**340,55**	Q8IXT5	**RNA-binding protein 12B**	RBM12B
0,005	1,74	3,34	**333,99**	Q9NR30	**Nucleolar RNA helicase 2**	DDX21
0,003	1,73	3,32	**331,59**	Q8NCN5	**Pyruvate dehydrogenase phosphatase regulatory subunit, mitochondrial**	PDPR
0,040	1,73	3,31	**331,28**	P82912	**28S ribosomal protein S11, mitochondrial**	MRPS11
0,032	1,73	3,31	**330,77**	Q8IY17	**Neuropathy target esterase**	PNPLA6
0,001	1,72	3,29	**328,91**	O95197	**Reticulon-3**	RTN3
0,002	1,71	3,28	**328,27**	Q01415	**N-acetylgalactosamine kinase**	GALK2
0,009	1,70	3,25	**324,90**	Q14203	**Dynactin subunit 1**	DCTN1
0,020	1,70	3,24	**323,90**	Q6ZW49	**PAX-interacting protein 1**	PAXIP1
0,007	1,69	3,23	**323,27**	Q9NXV2	**BTB/POZ domain-containing protein KCTD5**	KCTD5
0,028	1,69	3,23	**322,81**	Q9BVK6	**Transmembrane emp24 domain-containing protein 9**	TMED9
0,018	1,69	3,23	**322,66**	Q92597	**Protein NDRG1**	NDRG1
0,016	1,68	3,21	**321,46**	Q96SY0	**von Willebrand factor A domain-containing protein 9**	VWA9
0,005	1,68	3,21	**321,03**	Q13596	**Sorting nexin-1**	SNX1

0,040	1,68	3,20	**320,26**	Q9NY27	**Serine/threonine-protein phosphatase 4 regulatory subunit 2**	PPP4R2
0,000	1,65	3,14	**313,69**	Q7RTV0	**PHD finger-like domain-containing protein 5A**	PHF5A
0,035	1,65	3,13	**313,02**	P09234	**U1 small nuclear ribonucleoprotein C**	SNRPC
0,001	1,64	3,11	**311,40**	P49756	**RNA-binding protein 25**	RBM25
0,021	1,64	3,11	**311,13**	Q9NPE3	**H/ACA ribonucleoprotein complex subunit 3**	NOP10
0,011	1,63	3,11	**310,58**	Q92820	**Gamma-glutamyl hydrolase**	GGH
0,045	1,63	3,09	**309,14**	Q14683	**Structural maintenance of chromosomes protein 1A**	SMC1A
0,018	1,62	3,07	**307,38**	Q9BVQ7	**Spermatogenesis-associated protein 5-like protein 1**	SPATA5L1
0,012	1,59	3,00	**300,39**	Q9Y570	**Protein phosphatase methylesterase 1**	PPME1
0,022	1,58	3,00	**299,96**	Q8IX12	**Cell division cycle and apoptosis regulator protein 1**	CCAR1
0,028	1,58	2,99	**299,29**	Q9NZJ6	**Hexaprenyldihydroxybenzoate methyltransferase, mitochondrial**	COQ3
0,041	1,56	2,94	**293,88**	Q5ZPR3	**CD276 antigen**	CD276
0,028	1,55	2,93	**292,82**	Q9Y5Z9	**UbiA prenyltransferase domain-containing protein 1**	UBIAD1
0,002	1,54	2,91	**291,03**	Q96LD4	**Tripartite motif-containing protein 47**	TRIM47
0,000	1,52	2,87	**287,17**	Q9NVR2	**Integrator complex subunit 10**	INTS10
0,030	1,51	2,85	**284,68**	Q96FV9	**THO complex subunit 1**	THOC1
0,041	1,50	2,83	**282,84**	Q7Z460	**CLIP-associating protein 1**	CLASP1
0,034	1,50	2,82	**282,16**	Q9H2W6	**39S ribosomal protein L46, mitochondrial**	MRPL46
0,015	1,49	2,81	**280,89**	Q8N442	**Translation factor GUF1, mitochondrial**	GUF1
0,040	1,48	2,79	**279,21**	Q9BZF9	**Uveal autoantigen with coiled-coil domains and ankyrin repeats**	UACA
0,039	1,48	2,79	**278,95**	P21926	**CD9 antigen**	CD9
0,021	1,48	2,78	**278,42**	P45954	**Short/branched chain specific acyl-CoA dehydrogenase, mitochondrial**	ACADSB
0,041	1,47	2,77	**277,02**	O15382	**Branched-chain-amino-acid aminotransferase, mitochondrial**	BCAT2
0,027	1,46	2,75	**275,31**	Q9BRA0	**N-alpha-acetyltransferase 38, NatC auxiliary subunit**	NAA38
0,039	1,46	2,75	**275,11**	P22307	**Non-specific lipid-transfer protein**	SCP2
0,009	1,46	2,75	**275,11**	P30622	**CAP-Gly domain-containing linker protein 1**	CLIP1
0,004	1,46	2,75	**274,62**	Q9H2J4	**Phosducin-like protein 3**	PDCL3
0,005	1,46	2,74	**274,50**	Q8TE58	**A disintegrin and metalloproteinase with thrombospondin motifs 15**	ADAMTS15
0,043	1,45	2,74	**274,01**	Q96PV6	**Leukocyte receptor cluster member 8**	LENG8
0,003	1,45	2,73	**273,46**	Q96KR1	**Zinc finger RNA-binding protein**	ZFR
0,012	1,45	2,73	**273,21**	Q9UGI8	**Testin**	TES
0,011	1,44	2,72	**271,86**	Q15014	**Mortality factor 4-like protein 2**	MORF4L2
0,013	1,44	2,71	**270,61**	P53582	**Methionine aminopeptidase 1**	METAP1

0,002	1,43	2,70	**269,99**	Q9BY77	**Polymerase delta-interacting protein 3**	POLDIP3
0,027	1,43	2,69	**269,45**	P54619	**5-AMP-activated protein kinase subunit gamma-1**	PRKAG1
0,033	1,43	2,69	**269,45**	Q9NYK5	**39S ribosomal protein L39, mitochondrial**	MRPL39
0,017	1,42	2,68	**267,59**	Q9C0D5	**Protein TANC1**	TANC1
0,001	1,42	2,67	**267,01**	Q9UNX4	**WD repeat-containing protein 3**	WDR3
0,024	1,40	2,64	**263,82**	P14866	**Heterogeneous nuclear ribonucleoprotein L**	HNRNPL
0,024	1,40	2,63	**263,24**	Q8N983	**39S ribosomal protein L43, mitochondrial**	MRPL43
0,025	1,38	2,60	**260,20**	P48553	**Trafficking protein particle complex subunit 10**	TRAPPC10
0,015	1,38	2,60	**260,15**	P55265	**Double-stranded RNA-specific adenosine deaminase**	ADAR
0,043	1,37	2,58	**258,47**	O95171	**Sciellin**	SCEL
0,001	1,37	2,58	**257,99**	Q99459	**Cell division cycle 5-like protein**	CDC5L
0,044	1,35	2,55	**255,11**	Q14BN4	**Sarcolemmal membrane-associated protein**	SLMAP
0,043	1,35	2,55	**255,05**	Q8TEM1	**Nuclear pore membrane glycoprotein 210**	NUP210
0,004	1,35	2,55	**254,96**	Q8WXF1	**Paraspeckle component 1**	PSPC1
0,008	1,35	2,55	**254,91**	O00401	**Neural Wiskott-Aldrich syndrome protein**	WASL
0,024	1,34	2,53	**253,15**	P29279	**Connective tissue growth factor**	CTGF
0,006	1,33	2,51	**250,76**	Q13627;Q9Y463	**Dual specificity tyrosine-phosphorylation-regulated kinase 1A;Dual specificity tyrosi**	DYRK1A;DYRK1B
0,021	1,32	2,50	**250,40**	P12270	**Nucleoprotein TPR**	TPR
0,002	1,32	2,50	**250,37**	Q9H0B6	**Kinesin light chain 2**	KLC2
0,007	1,32	2,50	**249,67**	Q4VC44	**FLYWCH-type zinc finger-containing protein 1**	FLYWCH1
0,003	1,30	2,46	**246,23**	P17275	**Transcription factor jun-B**	JUNB
0,049	1,30	2,46	**246,23**	Q9Y244	**Proteasome maturation protein**	POMP
0,003	1,30	2,46	**245,50**	P51149	**Ras-related protein Rab-7a**	RAB7A
0,026	1,29	2,45	**244,84**	Q15233	**Non-POU domain-containing octamer-binding protein**	NONO
0,002	1,29	2,45	**244,53**	Q03468	**DNA excision repair protein ERCC-6**	ERCC6
0,024	1,28	2,42	**242,21**	Q96T76	**MMS19 nucleotide excision repair protein homolog**	MMS19
0,017	1,27	2,42	**241,75**	Q9BX40	**Protein LSM14 homolog B**	LSM14B
0,043	1,27	2,41	**240,74**	Q9Y3C4	**EKC/KEOPS complex subunit TPRKB**	TPRKB
0,037	1,26	2,40	**240,08**	Q00169	**Phosphatidylinositol transfer protein alpha isoform**	PITPNA
0,011	1,25	2,38	**238,43**	Q9UIC8	**Leucine carboxyl methyltransferase 1**	LCMT1
0,020	1,24	2,36	**236,43**	P20700	**Lamin-B1**	LMNB1
0,035	1,24	2,36	**236,20**	P30838	**Aldehyde dehydrogenase, dimeric NADP-preferring**	ALDH3A1
0,044	1,23	2,35	**234,57**	O60610	**Protein diaphanous homolog 1**	DIAPH1

0,020	1,23	2,34	**234,40**	Q9NQW6	**Actin-binding protein anillin**	ANLN
0,031	1,23	2,34	**234,03**	P49354	**Protein farnesyltransferase/geranylgeranyltransferase type-1 subunit alpha**	FNTA
0,045	1,22	2,33	**233,04**	P15170;Q8IYD1	**Eukaryotic peptide chain release factor GTP-binding subunit ERF3A;Eukaryotic pepti**	GSPT1;GSPT2
0,048	1,22	2,33	**232,55**	Q15397	**Pumilio domain-containing protein KIAA0020**	KIAA0020
0,000	1,22	2,32	**232,35**	P14406	**Cytochrome c oxidase subunit 7A2, mitochondrial**	COX7A2
0,030	1,21	2,32	**231,78**	P46100	**Transcriptional regulator ATRX**	ATRX
0,040	1,21	2,31	**231,31**	A6NKD9	**Coiled-coil domain-containing protein 85C**	CCDC85C
0,047	1,20	2,30	**229,68**	P11137	**Microtubule-associated protein 2**	MAP2
0,047	1,20	2,29	**229,47**	P51610	**Host cell factor 1;HCF N-terminal chain 1;HCF N-terminal chain 2;HCF N-terminal cha**	HCFC1
0,034	1,19	2,29	**228,93**	P52732	**Kinesin-like protein KIF11**	KIF11
0,015	1,19	2,28	**228,15**	Q15785	**Mitochondrial import receptor subunit TOM34**	TOMM34
0,003	1,19	2,28	**227,85**	P01130	**Low-density lipoprotein receptor**	LDLR
0,024	1,18	2,27	**226,58**	P48735	**Isocitrate dehydrogenase [NADP], mitochondrial**	IDH2
0,023	1,18	2,27	**226,58**	P09429	**High mobility group protein B1**	HMGB1
0,006	1,16	2,23	**223,43**	O75663	**TIP41-like protein**	TIPRL
0,005	1,15	2,22	**222,09**	Q8N556	**Actin filament-associated protein 1**	AFAP1
0,015	1,15	2,22	**221,91**	Q7Z2Z2	**Elongation factor Tu GTP-binding domain-containing protein 1**	EFTUD1
0,048	1,15	2,22	**221,52**	Q15459	**Splicing factor 3A subunit 1**	SF3A1
0,009	1,15	2,21	**221,36**	O75643	**U5 small nuclear ribonucleoprotein 200 kDa helicase**	SNRNP200
0,035	1,14	2,21	**220,76**	O00567	**Nucleolar protein 56**	NOP56
0,012	1,14	2,21	**220,63**	Q01105;P0DME0	**Protein SET;Protein SETSIP**	SET;SETSIP
0,035	1,14	2,20	**220,43**	Q96EY4	**Translation machinery-associated protein 16**	TMA16
0,003	1,14	2,20	**219,87**	Q969G3	**SWI/SNF-related matrix-associated actin-dependent regulator of chromatin subfami**	SMARCE1
0,003	1,13	2,19	**219,29**	P49916	**DNA ligase 3**	LIG3
0,037	1,13	2,19	**219,28**	P32321	**Deoxycytidylate deaminase**	DCTD
0,044	1,13	2,19	**219,19**	Q92466	**DNA damage-binding protein 2**	DDB2
0,027	1,13	2,19	**219,09**	Q8WUX9	**Charged multivesicular body protein 7**	CHMP7
0,031	1,12	2,17	**217,35**	P26583	**High mobility group protein B2**	HMGB2
0,023	1,11	2,16	**216,32**	Q03701	**CCAAT/enhancer-binding protein zeta**	CEBPZ
0,004	1,11	2,16	**215,85**	Q04206	**Transcription factor p65**	RELA
0,032	1,10	2,14	**214,16**	O94864	**STAGA complex 65 subunit gamma**	SUPT7L
0,001	1,10	2,14	**214,04**	P04637	**Cellular tumor antigen p53**	TP53
0,033	1,09	2,12	**212,30**	O75150	**E3 ubiquitin-protein ligase BRE1B**	RNF40

0,009	1,08	2,11	**211,02**	O15357	**Phosphatidylinositol 3,4,5-trisphosphate 5-phosphatase 2**	INPPL1
0,001	1,08	2,11	**210,70**	O60341	**Lysine-specific histone demethylase 1A**	KDM1A
0,020	1,07	2,10	**209,94**	O95182	**NADH dehydrogenase [ubiquinone] 1 alpha subcomplex subunit 7**	NDUFA7
0,004	1,07	2,09	**209,49**	Q92609	**TBC1 domain family member 5**	TBC1D5
0,028	1,05	2,07	**207,23**	Q13506	**NGFI-A-binding protein 1**	NAB1
0,035	1,05	2,07	**207,05**	Q86Y39	**NADH dehydrogenase [ubiquinone] 1 alpha subcomplex subunit 11**	NDUFA11
0,023	1,04	2,06	**206,07**	Q9Y2X3	**Nucleolar protein 58**	NOP58
0,028	1,04	2,06	**205,68**	O75674	**TOM1-like protein 1**	TOM1L1
0,011	1,03	2,05	**204,79**	Q86X55	**Histone-arginine methyltransferase CARM1**	CARM1
0,025	1,03	2,05	**204,58**	Q9BPX3	**Condensin complex subunit 3**	NCAPG
0,011	1,03	2,04	**203,79**	O76071	**Probable cytosolic iron-sulfur protein assembly protein CIAO1**	CIAO1
0,007	1,02	2,03	**203,49**	Q9H2G2	**STE20-like serine/threonine-protein kinase**	SLK
0,031	1,02	2,03	**202,79**	Q92615	**La-related protein 4B**	LARP4B
0,010	1,02	2,03	**202,79**	Q9NW82	**WD repeat-containing protein 70**	WDR70
0,037	1,01	2,02	**202,02**	Q6GMV3	**Putative peptidyl-tRNA hydrolase PTRHD1**	PTRHD1
0,001	0,99	1,99	**198,88**	P35658	**Nuclear pore complex protein Nup214**	NUP214
0,025	0,99	1,99	**198,69**	Q9H3S7	**Tyrosine-protein phosphatase non-receptor type 23**	PTPN23
0,045	0,99	1,99	**198,62**	P26006	**Integrin alpha-3;Integrin alpha-3 heavy chain;Integrin alpha-3 light chain**	ITGA3
0,024	0,98	1,97	**197,25**	Q9Y3Y2	**Chromatin target of PRMT1 protein**	CHTOP
0,038	0,98	1,97	**197,12**	P31949	**Protein S100-A11;Protein S100-A11, N-terminally processed**	S100A11
0,035	0,98	1,97	**196,70**	Q9BW27	**Nuclear pore complex protein Nup85**	NUP85
0,007	0,97	1,96	**196,46**	Q9NUB1	**Acetyl-coenzyme A synthetase 2-like, mitochondrial**	ACSS1
0,008	0,97	1,96	**196,21**	P07910	**Heterogeneous nuclear ribonucleoproteins C1/C2**	HNRNPC
0,012	0,97	1,96	**195,87**	Q96BZ8	**Leukocyte receptor cluster member 1**	LENG1
0,001	0,97	1,96	**195,74**	Q9Y5X1	**Sorting nexin-9**	SNX9
0,030	0,97	1,96	**195,73**	Q9UKN8	**General transcription factor 3C polypeptide 4**	GTF3C4
0,008	0,97	1,95	**195,26**	Q16890	**Tumor protein D53**	TPD52L1
0,032	0,96	1,95	**195,10**	Q9H098	**Protein FAM107B**	FAM107B
0,024	0,96	1,94	**194,47**	Q9NVH0	**Exonuclease 3-5 domain-containing protein 2**	EXD2
0,038	0,95	1,93	**193,19**	P61009	**Signal peptidase complex subunit 3**	SPCS3
0,007	0,95	1,93	**192,97**	Q9H0C8	**Integrin-linked kinase-associated serine/threonine phosphatase 2C**	ILKAP
0,029	0,95	1,93	**192,84**	Q9HAV7	**GrpE protein homolog 1, mitochondrial**	GRPEL1
0,009	0,93	1,91	**190,64**	Q6PJT7	**Zinc finger CCCH domain-containing protein 14**	ZC3H14

0,007	0,92	1,89	**189,21**	Q9BY42	**Protein RTF2 homolog**	RTFDC1
0,022	0,92	1,89	**188,94**	O75494	**Serine/arginine-rich splicing factor 10**	SRSF10
0,014	0,92	1,89	**188,56**	O95602	**DNA-directed RNA polymerase I subunit RPA1**	POLR1A
0,003	0,90	1,87	**186,61**	P25205	**DNA replication licensing factor MCM3**	MCM3
0,043	0,90	1,87	**186,61**	P32929	**Cystathionine gamma-lyase**	CTH
0,021	0,90	1,86	**186,46**	Q9NR12	**PDZ and LIM domain protein 7**	PDLIM7
0,045	0,89	1,86	**185,74**	Q96L92	**Sorting nexin-27**	SNX27
0,023	0,89	1,85	**185,21**	O15231	**Zinc finger protein 185**	ZNF185
0,023	0,89	1,85	**184,82**	O43291	**Kunitz-type protease inhibitor 2**	SPINT2
0,008	0,88	1,84	**184,36**	Q96MW1	**Coiled-coil domain-containing protein 43**	CCDC43
0,039	0,88	1,84	**183,99**	Q12874	**Splicing factor 3A subunit 3**	SF3A3
0,007	0,88	1,84	**183,86**	Q9Y224	**UPF0568 protein C14orf166**	C14orf166
0,023	0,87	1,83	**183,38**	Q92575	**UBX domain-containing protein 4**	UBXN4
0,043	0,86	1,82	**181,69**	Q9Y305	**Acyl-coenzyme A thioesterase 9, mitochondrial**	ACOT9
0,026	0,86	1,81	**181,42**	Q7L014	**Probable ATP-dependent RNA helicase DDX46**	DDX46
0,000	0,86	1,81	**181,29**	P52789	**Hexokinase-2**	HK2
0,024	0,85	1,80	**179,91**	Q5VZE5	**N-alpha-acetyltransferase 35, NatC auxiliary subunit**	NAA35
0,032	0,84	1,79	**179,50**	Q13509	**Tubulin beta-3 chain**	TUBB3
0,039	0,84	1,79	**179,17**	Q96RU3	**Formin-binding protein 1**	FNBP1
0,014	0,84	1,79	**179,01**	P40938	**Replication factor C subunit 3**	RFC3
0,001	0,84	1,78	**178,47**	Q9H553	**Alpha-1,3/1,6-mannosyltransferase ALG2**	ALG2
0,050	0,83	1,78	**177,80**	Q9UQ35	**Serine/arginine repetitive matrix protein 2**	SRRM2
0,025	0,82	1,77	**177,12**	P52272	**Heterogeneous nuclear ribonucleoprotein M**	HNRNPM
0,014	0,82	1,76	**176,13**	Q8IWW6	**Rho GTPase-activating protein 12**	ARHGAP12
0,023	0,81	1,76	**175,62**	Q9H7Z7	**Prostaglandin E synthase 2;Prostaglandin E synthase 2 truncated form**	PTGES2
0,021	0,81	1,75	**175,35**	Q96GK7	**Fumarylacetoacetate hydrolase domain-containing protein 2A**	FAHD2A
0,020	0,81	1,75	**175,32**	Q9BTE7	**DCN1-like protein 5**	DCUN1D5
0,592	0,81	1,75	**175,07**	O00767	**Acyl-CoA desaturase**	SCD
0,020	0,81	1,75	**175,03**	P38159	**RNA-binding motif protein, X chromosome;RNA-binding motif protein, X chromosor**	RBMX
0,011	0,81	1,75	**174,88**	O95470	**Sphingosine-1-phosphate lyase 1**	SGPL1
0,002	0,80	1,75	**174,67**	Q8NI27	**THO complex subunit 2**	THOC2
0,018	0,80	1,74	**174,11**	Q14204	**Cytoplasmic dynein 1 heavy chain 1**	DYNC1H1
0,002	0,80	1,74	**173,85**	Q06203	**Amidophosphoribosyltransferase**	PPAT

0,022	0,79	1,73	**173,10**	P30740	**Leukocyte elastase inhibitor**	SERPINB1
0,028	0,78	1,72	**172,29**	O60568	**Procollagen-lysine,2-oxoglutarate 5-dioxygenase 3**	PLOD3
0,018	0,78	1,72	**171,81**	P35914	**Hydroxymethylglutaryl-CoA lyase, mitochondrial**	HMGCL
0,010	0,78	1,72	**171,71**	Q13561	**Dynactin subunit 2**	DCTN2
0,035	0,78	1,71	**171,49**	O15020	**Spectrin beta chain, non-erythrocytic 2**	SPTBN2
0,016	0,78	1,71	**171,31**	Q96A49	**Synapse-associated protein 1**	SYAP1
0,025	0,78	1,71	**171,20**	P63010	**AP-2 complex subunit beta**	AP2B1
0,022	0,77	1,71	**170,55**	P05783	**Keratin, type I cytoskeletal 18**	KRT18
0,002	0,77	1,70	**170,12**	Q5T4S7	**E3 ubiquitin-protein ligase UBR4**	UBR4
0,038	0,77	1,70	**170,00**	Q9H967	**WD repeat-containing protein 76**	WDR76
0,043	0,76	1,69	**169,24**	P55196	**Afadin**	MLLT4
0,026	0,76	1,69	**169,18**	Q12996	**Cleavage stimulation factor subunit 3**	CSTF3
0,043	0,74	1,67	**167,02**	Q9NS86	**LanC-like protein 2**	LANCL2
0,006	0,74	1,67	**167,02**	P33991	**DNA replication licensing factor MCM4**	MCM4
0,024	0,74	1,67	**167,02**	Q04323	**UBX domain-containing protein 1**	UBXN1
0,049	0,73	1,66	**166,25**	Q9UIJ7	**GTP:AMP phosphotransferase AK3, mitochondrial**	AK3
0,010	0,73	1,66	**166,08**	Q99536	**Synaptic vesicle membrane protein VAT-1 homolog**	VAT1
0,013	0,73	1,66	**165,97**	Q05682	**Caldesmon**	CALD1
0,007	0,73	1,65	**165,34**	Q9Y3A5	**Ribosome maturation protein SBDS**	SBDS
0,018	0,72	1,65	**164,68**	Q147X3	**N-alpha-acetyltransferase 30**	NAA30
0,022	0,72	1,64	**164,40**	P36915	**Guanine nucleotide-binding protein-like 1**	GNL1
0,005	0,71	1,64	**163,72**	Q15042	**Rab3 GTPase-activating protein catalytic subunit**	RAB3GAP1
0,032	0,70	1,63	**162,51**	P35270	**Sepiapterin reductase**	SPR
0,010	0,70	1,62	**162,27**	P43243	**Matrin-3**	MATR3
0,029	0,70	1,62	**162,07**	Q9UBQ0	**Vacuolar protein sorting-associated protein 29**	VPS29
0,011	0,69	1,62	**161,78**	P49757	**Protein numb homolog**	NUMB
0,022	0,68	1,60	**160,39**	P29218	**Inositol monophosphatase 1**	IMPA1
0,038	0,68	1,60	**160,21**	P51812	**Ribosomal protein S6 kinase alpha-3**	RPS6KA3
0,010	0,68	1,60	**159,93**	O00442	**RNA 3-terminal phosphate cyclase**	RTCA
0,007	0,67	1,59	**158,91**	O00330	**Pyruvate dehydrogenase protein X component, mitochondrial**	PDHX
0,048	0,67	1,59	**158,57**	Q9BRT3	**Migration and invasion enhancer 1**	MIEN1
0,012	0,66	1,58	**158,47**	Q14980	**Nuclear mitotic apparatus protein 1**	NUMA1
0,014	0,66	1,58	**158,13**	P00441	**Superoxide dismutase [Cu-Zn]**	SOD1

0,001	0,66	1,58	**158,01**	Q14566	**DNA replication licensing factor MCM6**	MCM6
0,000	0,66	1,58	**157,77**	P29317	**Ephrin type-A receptor 2**	EPHA2
0,005	0,66	1,58	**157,61**	O15031	**Plexin-B2**	PLXNB2
0,009	0,66	1,58	**157,59**	P22061	**Protein-L-isoaspartate(D-aspartate) O-methyltransferase**	PCMT1
0,045	0,65	1,57	**156,92**	Q9BS26	**Endoplasmic reticulum resident protein 44**	ERP44
0,003	0,65	1,57	**156,69**	Q01813	**ATP-dependent 6-phosphofructokinase, platelet type**	PFKP
0,029	0,64	1,56	**155,83**	Q9H1B7	**Interferon regulatory factor 2-binding protein-like**	IRF2BPL
0,048	0,64	1,56	**155,83**	Q99627	**COP9 signalosome complex subunit 8**	COPS8
0,004	0,63	1,55	**154,95**	Q9BRX2	**Protein pelota homolog**	PELO
0,003	0,63	1,55	**154,72**	P50452	**Serpin B8**	SERPINB8
0,037	0,63	1,54	**154,29**	Q9Y4Z0	**U6 snRNA-associated Sm-like protein LSm4**	LSM4
0,033	0,62	1,54	**153,69**	Q09666	**Neuroblast differentiation-associated protein AHNAK**	AHNAK
0,005	0,62	1,53	**153,48**	P82979	**SAP domain-containing ribonucleoprotein**	SARNP
0,045	0,61	1,53	**153,12**	O95817	**BAG family molecular chaperone regulator 3**	BAG3
0,025	0,61	1,53	**152,68**	Q9Y6Q5	**AP-1 complex subunit mu-2**	AP1M2
0,029	0,61	1,53	**152,60**	Q96AT9	**Ribulose-phosphate 3-epimerase**	RPE
0,003	0,61	1,52	**152,16**	Q13136	**Liprin-alpha-1**	PPFIA1
0,007	0,59	1,51	**150,52**	P49736	**DNA replication licensing factor MCM2**	MCM2
0,036	0,59	1,50	**150,41**	Q6FI81	**Anamorsin**	CIAPIN1
0,039	0,58	1,50	**149,72**	Q9NW64	**Pre-mRNA-splicing factor RBM22**	RBM22
0,027	0,58	1,49	**149,48**	Q8WZA0	**Protein LZIC**	LZIC
0,021	0,58	1,49	**149,48**	Q9BT78	**COP9 signalosome complex subunit 4**	COPS4
0,047	0,58	1,49	**149,37**	Q9NQR4	**Omega-amidase NIT2**	NIT2
0,035	0,58	1,49	**149,24**	P24821	**Tenascin**	TNC
0,038	0,57	1,49	**148,79**	P22033	**Methylmalonyl-CoA mutase, mitochondrial**	MUT
0,047	0,57	1,49	**148,59**	Q9P000	**COMM domain-containing protein 9**	COMMD9
0,046	0,56	1,48	**147,80**	P41229	**Lysine-specific demethylase 5C**	KDM5C
0,039	0,56	1,47	**147,43**	Q7Z3J2	**UPF0505 protein C16orf62**	C16orf62
0,042	0,56	1,47	**147,35**	O95163	**Elongator complex protein 1**	IKBKAP
0,004	0,56	1,47	**147,00**	Q7Z2E3	**Aprataxin**	APTX
0,010	0,55	1,47	**146,71**	Q9ULV0	**Unconventional myosin-Vb**	MYO5B
0,022	0,55	1,46	**146,49**	Q9Y6C9	**Mitochondrial carrier homolog 2**	MTCH2
0,023	0,55	1,46	**146,41**	Q8IY18	**Structural maintenance of chromosomes protein 5**	SMC5

0,018	0,55	1,46	**146,13**	Q08211	**ATP-dependent RNA helicase A**	DHX9
0,020	0,54	1,46	**145,74**	P43304	**Glycerol-3-phosphate dehydrogenase, mitochondrial**	GPD2
0,008	0,54	1,46	**145,54**	P18065	**Insulin-like growth factor-binding protein 2**	IGFBP2
0,034	0,54	1,45	**145,46**	O15126	**Secretory carrier-associated membrane protein 1**	SCAMP1
0,021	0,54	1,45	**145,40**	Q14847	**LIM and SH3 domain protein 1**	LASP1
0,009	0,54	1,45	**145,40**	P33993	**DNA replication licensing factor MCM7**	MCM7
0,000	0,54	1,45	**145,08**	P52306	**Rap1 GTPase-GDP dissociation stimulator 1**	RAP1GDS1
0,011	0,53	1,44	**144,07**	Q13011	**Delta(3,5)-Delta(2,4)-dienoyl-CoA isomerase, mitochondrial**	ECH1
0,030	0,53	1,44	**144,02**	Q9H8S9	**MOB kinase activator 1A**	MOB1A
0,020	0,52	1,44	**143,63**	P21980	**Protein-glutamine gamma-glutamyltransferase 2**	TGM2
0,004	0,52	1,43	**143,40**	P28066	**Proteasome subunit alpha type-5**	PSMA5
0,023	0,51	1,43	**142,68**	Q99426	**Tubulin-folding cofactor B**	TBCB
0,023	0,51	1,43	**142,61**	P02545	**Prelamin-A/C;Lamin-A/C**	LMNA
0,019	0,51	1,42	**142,41**	Q99714	**3-hydroxyacyl-CoA dehydrogenase type-2**	HSD17B10
0,000	0,51	1,42	**142,41**	P40818	**Ubiquitin carboxyl-terminal hydrolase 8**	USP8
0,031	0,51	1,42	**142,41**	P17535	**Transcription factor jun-D**	JUND
0,006	0,49	1,41	**140,66**	P07919	**Cytochrome b-c1 complex subunit 6, mitochondrial**	UQCRH
0,043	0,48	1,40	**139,91**	P39060	**Collagen alpha-1(XVIII) chain;Endostatin**	COL18A1
0,048	0,48	1,40	**139,54**	Q16658	**Fascin**	FSCN1
0,014	0,48	1,39	**139,43**	P17844	**Probable ATP-dependent RNA helicase DDX5**	DDX5
0,007	0,48	1,39	**139,39**	P20674	**Cytochrome c oxidase subunit 5A, mitochondrial**	COX5A
0,030	0,48	1,39	**139,29**	Q9P0J1	**[Pyruvate dehydrogenase [acetyl-transferring]]-phosphatase 1, mitochondrial**	PDP1
0,020	0,48	1,39	**139,13**	Q5TA45	**Integrator complex subunit 11**	CPSF3L
0,025	0,47	1,39	**138,93**	P20585	**DNA mismatch repair protein Msh3**	MSH3
0,037	0,47	1,39	**138,77**	Q13363	**C-terminal-binding protein 1**	CTBP1
0,019	0,46	1,37	**137,38**	P33240	**Cleavage stimulation factor subunit 2**	CSTF2
0,038	0,45	1,37	**136,60**	Q02833	**Ras association domain-containing protein 7**	RASSF7
0,047	0,45	1,36	**136,30**	O43795	**Unconventional myosin-Ib**	MYO1B
0,021	0,44	1,36	**136,06**	P49711	**Transcriptional repressor CTCF**	CTCF
0,020	0,44	1,36	**135,95**	Q6NXS1;P41236	**Protein phosphatase inhibitor 2-like protein 3;Protein phosphatase inhibitor 2**	PPP1R2P3;PPP1R2
0,045	0,44	1,36	**135,66**	O75531	**Barrier-to-autointegration factor;Barrier-to-autointegration factor, N-terminally pro**	BANF1
0,037	0,44	1,36	**135,63**	Q9UKL0	**REST corepressor 1**	RCOR1
0,003	0,43	1,35	**134,84**	Q9H7E9	**UPF0488 protein C8orf33**	C8orf33

0,000	0,43	1,35	**134,72**	Q15554	**Telomeric repeat-binding factor 2**	TERF2
0,002	0,43	1,34	**134,35**	P38919	**Eukaryotic initiation factor 4A-III;Eukaryotic initiation factor 4A-III, N-terminally pro**	EIF4A3
0,011	0,42	1,34	**133,79**	P34897	**Serine hydroxymethyltransferase, mitochondrial**	SHMT2
0,041	0,42	1,34	**133,79**	Q9UNZ2	**NSFL1 cofactor p47**	NSFL1C
0,027	0,42	1,34	**133,54**	Q9BUR5	**Apolipoprotein O**	APOO
0,046	0,41	1,33	**133,18**	Q9NUQ7	**Ufm1-specific protease 2**	UFSP2
0,014	0,41	1,33	**133,10**	Q93009	**Ubiquitin carboxyl-terminal hydrolase 7**	USP7
0,041	0,41	1,33	**132,77**	P06733	**Alpha-enolase**	ENO1
0,046	0,41	1,33	**132,60**	Q15424	**Scaffold attachment factor B1**	SAFB
0,014	0,40	1,32	**132,35**	O43447	**Peptidyl-prolyl cis-trans isomerase H**	PPIH
0,008	0,40	1,32	**131,91**	Q8TDP1	**Ribonuclease H2 subunit C**	RNASEH2C
0,027	0,40	1,32	**131,79**	Q9Y2W2	**WW domain-binding protein 11**	WBP11
0,029	0,39	1,31	**131,12**	O43719	**HIV Tat-specific factor 1**	HTATSF1
0,009	0,39	1,31	**130,76**	P52758	**Ribonuclease UK114**	HRSP12
0,045	0,38	1,30	**130,41**	Q9UBI6	**Guanine nucleotide-binding protein G(I)/G(S)/G(O) subunit gamma-12**	GNG12
0,033	0,38	1,30	**130,30**	Q15061	**WD repeat-containing protein 43**	WDR43
0,037	0,38	1,30	**130,12**	Q9NRX1	**RNA-binding protein PNO1**	PNO1
0,047	0,38	1,30	**129,86**	Q92696	**Geranylgeranyl transferase type-2 subunit alpha**	RABGGTA
0,010	0,37	1,29	**129,36**	Q9H6S3	**Epidermal growth factor receptor kinase substrate 8-like protein 2**	EPS8L2
0,017	0,37	1,29	**129,32**	Q9UI12	**V-type proton ATPase subunit H**	ATP6V1H
0,035	0,37	1,29	**129,28**	P19474	**E3 ubiquitin-protein ligase TRIM21**	TRIM21
0,007	0,37	1,29	**129,24**	P55072	**Transitional endoplasmic reticulum ATPase**	VCP
0,005	0,37	1,29	**129,12**	Q86W92	**Liprin-beta-1**	PPFIBP1
0,015	0,37	1,29	**129,06**	Q86X76	**Nitrilase homolog 1**	NIT1
0,044	0,36	1,29	**128,77**	Q8WX93	**Palladin**	PALLD
0,049	0,36	1,28	**128,16**	P07902	**Galactose-1-phosphate uridylyltransferase**	GALT
0,031	0,36	1,28	**128,13**	Q7Z2W4	**Zinc finger CCCH-type antiviral protein 1**	ZC3HAV1
0,048	0,36	1,28	**127,91**	Q15149	**Plectin**	PLEC
0,016	0,35	1,28	**127,81**	P08865	**40S ribosomal protein SA**	RPSA
0,038	0,35	1,27	**127,30**	P29144	**Tripeptidyl-peptidase 2**	TPP2
0,029	0,34	1,27	**127,01**	Q9H3U1	**Protein unc-45 homolog A**	UNC45A
0,001	0,34	1,27	**126,76**	P08195	**4F2 cell-surface antigen heavy chain**	SLC3A2
0,047	0,34	1,26	**126,33**	Q9UJS0	**Calcium-binding mitochondrial carrier protein Aralar2**	SLC25A13

0,008	0,34	1,26	**126,26**	Q9Y237	**Peptidyl-prolyl cis-trans isomerase NIMA-interacting 4**	PIN4
0,022	0,34	1,26	**126,24**	Q15427	**Splicing factor 3B subunit 4**	SF3B4
0,017	0,33	1,26	**125,59**	P46379	**Large proline-rich protein BAG6**	BAG6
0,042	0,33	1,25	**125,46**	P04181	**Ornithine aminotransferase, mitochondrial;Ornithine aminotransferase, hepatic for**	OAT
0,030	0,32	1,25	**125,05**	Q9Y617	**Phosphoserine aminotransferase**	PSAT1
0,009	0,32	1,25	**124,66**	Q9Y6E2	**Basic leucine zipper and W2 domain-containing protein 2**	BZW2
0,029	0,32	1,24	**124,46**	Q9NP92	**28S ribosomal protein S30, mitochondrial**	MRPS30
0,001	0,31	1,24	**124,40**	P20810	**Calpastatin**	CAST
0,000	0,31	1,24	**124,31**	Q86VP6	**Cullin-associated NEDD8-dissociated protein 1**	CAND1
0,024	0,31	1,24	**124,16**	P62269	**40S ribosomal protein S18**	RPS18
0,031	0,31	1,24	**124,07**	Q9C0C2	**182 kDa tankyrase-1-binding protein**	TNKS1BP1
0,038	0,31	1,24	**124,00**	P39687	**Acidic leucine-rich nuclear phosphoprotein 32 family member A**	ANP32A
0,005	0,31	1,24	**123,97**	P35998	**26S protease regulatory subunit 7**	PSMC2
0,031	0,31	1,24	**123,94**	Q13838	**Spliceosome RNA helicase DDX39B**	DDX39B
0,030	0,30	1,23	**123,25**	Q92538	**Golgi-specific brefeldin A-resistance guanine nucleotide exchange factor 1**	GBF1
0,006	0,30	1,23	**122,80**	P23396	**40S ribosomal protein S3**	RPS3
0,045	0,29	1,23	**122,53**	P60174	**Triosephosphate isomerase**	TPI1
0,015	0,29	1,22	**122,45**	Q9NX63	**MICOS complex subunit MIC19**	CHCHD3
0,026	0,29	1,22	**122,26**	P24752	**Acetyl-CoA acetyltransferase, mitochondrial**	ACAT1
0,018	0,29	1,22	**122,26**	Q9NV31	**U3 small nucleolar ribonucleoprotein protein IMP3**	IMP3
0,012	0,29	1,22	**122,26**	Q9Y5K5	**Ubiquitin carboxyl-terminal hydrolase isozyme L5**	UCHL5
0,045	0,29	1,22	**122,19**	P62873	**Guanine nucleotide-binding protein G(I)/G(S)/G(T) subunit beta-1**	GNB1
0,001	0,28	1,22	**121,79**	Q7Z4L5	**Tetratricopeptide repeat protein 21B**	TTC21B
0,010	0,28	1,22	**121,79**	P32119	**Peroxiredoxin-2**	PRDX2
0,039	0,28	1,22	**121,66**	P40926	**Malate dehydrogenase, mitochondrial**	MDH2
0,017	0,28	1,21	**121,11**	Q9NP81	**Serine--tRNA ligase, mitochondrial**	SARS2
0,010	0,27	1,21	**120,86**	P54577	**Tyrosine--tRNA ligase, cytoplasmic;Tyrosine--tRNA ligase, cytoplasmic, N-terminally**	YARS
0,022	0,27	1,21	**120,62**	P16278	**Beta-galactosidase**	GLB1
0,038	0,27	1,21	**120,58**	Q8N163	**Cell cycle and apoptosis regulator protein 2**	CCAR2
0,048	0,27	1,21	**120,58**	P34932	**Heat shock 70 kDa protein 4**	HSPA4
0,050	0,27	1,21	**120,58**	P62195	**26S protease regulatory subunit 8**	PSMC5
0,041	0,27	1,20	**120,45**	Q9H3P7	**Golgi resident protein GCP60**	ACBD3
0,014	0,26	1,20	**120,04**	P00505	**Aspartate aminotransferase, mitochondrial**	GOT2

0,011	0,26	1,20	**119,89**	Q96QK1	**Vacuolar protein sorting-associated protein 35**	VPS35
0,027	0,26	1,20	**119,84**	P12081	**Histidine--tRNA ligase, cytoplasmic**	HARS
0,024	0,26	1,20	**119,75**	Q9P258	**Protein RCC2**	RCC2
0,006	0,26	1,20	**119,57**	O95373	**Importin-7**	IPO7
0,048	0,25	1,19	**118,93**	P69849;Q5JPE7;Q15155	**Nodal modulator 3;Nodal modulator 2;Nodal modulator 1**	MO3;NOMO2;NOM
0,042	0,25	1,19	**118,92**	O95391	**Pre-mRNA-splicing factor SLU7**	SLU7
0,021	0,25	1,19	**118,92**	P18085	**ADP-ribosylation factor 4**	ARF4
0,006	0,25	1,19	**118,55**	Q9Y6M1	**Insulin-like growth factor 2 mRNA-binding protein 2**	IGF2BP2
0,007	0,24	1,18	**118,13**	Q8N6H7	**ADP-ribosylation factor GTPase-activating protein 2**	ARFGAP2
0,017	0,24	1,18	**117,96**	Q92973	**Transportin-1**	TNPO1
0,012	0,23	1,17	**117,28**	Q15717	**ELAV-like protein 1**	ELAVL1
0,050	0,22	1,16	**116,47**	O14818	**Proteasome subunit alpha type-7**	PSMA7
0,039	0,22	1,16	**116,43**	P63167	**Dynein light chain 1, cytoplasmic**	DYNLL1
0,007	0,22	1,16	**116,34**	P62241	**40S ribosomal protein S8**	RPS8
0,045	0,21	1,15	**115,42**	P16949	**Stathmin**	STMN1
0,024	0,21	1,15	**115,36**	Q14258	**E3 ubiquitin/ISG15 ligase TRIM25**	TRIM25
0,041	0,20	1,15	**114,87**	Q8WUF8	**Protein FAM172A**	FAM172A
0,013	0,19	1,14	**114,19**	Q6P1N0	**Coiled-coil and C2 domain-containing protein 1A**	CC2D1A
0,001	0,19	1,14	**114,09**	P05455	**Lupus La protein**	SSB
0,030	0,17	1,12	**112,37**	Q13131	**5-AMP-activated protein kinase catalytic subunit alpha-1**	PRKAA1
0,033	0,16	1,12	**111,73**	Q15020	**Squamous cell carcinoma antigen recognized by T-cells 3**	SART3
0,031	0,15	1,11	**110,67**	O76094	**Signal recognition particle subunit SRP72**	SRP72
**24h Up**						
0,011	6,44	86,82	**8682,27**	Q8N3F8	**MICAL-like protein 1**	MICALL1
0,043	5,94	61,39	**6139,29**	Q8IYS1	**Peptidase M20 domain-containing protein 2**	PM20D2
0,013	4,60	24,26	**2426,07**	P61769	**Beta-2-microglobulin;Beta-2-microglobulin form pI 5.3**	B2M
0,001	4,35	20,39	**2039,30**	Q92466	**DNA damage-binding protein 2**	DDB2
0,006	4,06	16,68	**1667,95**	O43237	**Cytoplasmic dynein 1 light intermediate chain 2**	DYNC1LI2
0,007	3,99	15,89	**1588,95**	Q9BYC8	**39S ribosomal protein L32, mitochondrial**	MRPL32
0,006	3,89	14,80	**1480,03**	P05090	**Apolipoprotein D**	APOD
0,009	3,78	13,74	**1373,70**	Q9HC36	**RNA methyltransferase-like protein 1**	RNMTL1
0,041	3,70	13,00	**1299,60**	O60678	**Protein arginine N-methyltransferase 3**	PRMT3

0,030	3,61	12,21	**1221,01**	Q9Y484	**WD repeat domain phosphoinositide-interacting protein 4**	WDR45
0,002	3,49	11,24	**1123,56**	Q9BU02	**Thiamine-triphosphatase**	THTPA
0,000	3,37	10,34	**1033,88**	Q8IXM3	**39S ribosomal protein L41, mitochondrial**	MRPL41
0,004	3,36	10,27	**1026,74**	Q8N983	**39S ribosomal protein L43, mitochondrial**	MRPL43
0,003	3,31	9,92	**991,77**	Q99569	**Plakophilin-4**	PKP4
0,037	3,23	9,38	**938,27**	Q9H019	**Mitochondrial fission regulator 1-like**	MTFR1L
0,000	3,15	8,88	**887,66**	Q8TAE8	**Growth arrest and DNA damage-inducible proteins-interacting protein 1**	GADD45GIP1
0,020	3,11	8,66	**866,08**	Q9Y483	**Metal-response element-binding transcription factor 2**	MTF2
0,033	3,01	8,07	**807,05**	P40261	**Nicotinamide N-methyltransferase**	NNMT
0,008	2,98	7,91	**791,17**	Q86UE8	**Serine/threonine-protein kinase tousled-like 2**	TLK2
0,011	2,94	7,67	**767,41**	O60762	**Dolichol-phosphate mannosyltransferase subunit 1**	DPM1
0,021	2,89	7,41	**741,27**	P11086	**Phenylethanolamine N-methyltransferase**	PNMT
0,046	2,85	7,23	**722,72**	P16403	**Histone H1.2**	HIST1H1C
0,033	2,82	7,06	**706,13**	Q96EK6	**Glucosamine 6-phosphate N-acetyltransferase**	GNPNAT1
0,037	2,79	6,92	**691,63**	P49406	**39S ribosomal protein L19, mitochondrial**	MRPL19
0,000	2,76	6,78	**677,83**	Q9Y2S6	**Translation machinery-associated protein 7**	TMA7
0,021	2,76	6,77	**677,40**	Q9H0U6	**39S ribosomal protein L18, mitochondrial**	MRPL18
0,023	2,72	6,57	**656,59**	Q01831	**DNA repair protein complementing XP-C cells**	XPC
0,000	2,71	6,54	**654,32**	Q96GC5	**39S ribosomal protein L48, mitochondrial**	MRPL48
0,043	2,68	6,43	**642,63**	O60888	**Protein CutA**	CUTA
0,044	2,66	6,31	**630,63**	Q9H3L0	**Methylmalonic aciduria and homocystinuria type D protein, mitochondrial**	MMADHC
0,000	2,62	6,15	**614,75**	Q0JRZ9	**FCH domain only protein 2**	FCHO2
0,021	2,61	6,11	**610,50**	Q9ULX9	**Transcription factor MafF**	MAFF
0,000	2,60	6,05	**604,54**	Q96JM3	**Chromosome alignment-maintaining phosphoprotein 1**	CHAMP1
0,021	2,54	5,82	**581,59**	Q9UNE7	**E3 ubiquitin-protein ligase CHIP**	STUB1
0,028	2,53	5,78	**577,57**	Q9P032	**NADH dehydrogenase [ubiquinone] 1 alpha subcomplex assembly factor 4**	NDUFAF4
0,003	2,52	5,74	**573,58**	Q9P015	**39S ribosomal protein L15, mitochondrial**	MRPL15
0,027	2,49	5,62	**561,78**	Q9NQ50	**39S ribosomal protein L40, mitochondrial**	MRPL40
0,001	2,47	5,52	**552,15**	Q3ZCW2	**Galectin-related protein**	LGALSL
0,038	2,46	5,52	**551,97**	Q15363	**Transmembrane emp24 domain-containing protein 2**	TMED2
0,000	2,44	5,43	**542,64**	P49321	**Nuclear autoantigenic sperm protein**	NASP
0,016	2,39	5,24	**524,16**	Q92552	**28S ribosomal protein S27, mitochondrial**	MRPS27
0,010	2,38	5,21	**520,50**	P06400	**Retinoblastoma-associated protein**	RB1

0,027	2,38	5,20	**520,09**	Q9H2F5	**Enhancer of polycomb homolog 1**	EPC1
0,011	2,36	5,13	**513,37**	Q13451	**Peptidyl-prolyl cis-trans isomerase FKBP5**	FKBP5
0,031	2,36	5,13	**513,37**	Q9H5N1	**Rab GTPase-binding effector protein 2**	RABEP2
0,011	2,33	5,03	**502,81**	Q2TAL8	**Glutamine-rich protein 1**	QRICH1
0,003	2,31	4,96	**495,88**	Q13409	**Cytoplasmic dynein 1 intermediate chain 2**	DYNC1I2
0,020	2,29	4,89	**489,06**	Q5T653	**39S ribosomal protein L2, mitochondrial**	MRPL2
0,003	2,28	4,86	**485,68**	Q9H974	**Queuine tRNA-ribosyltransferase subunit QTRTD1**	QTRTD1
0,028	2,28	4,86	**485,68**	Q9H3N1	**Thioredoxin-related transmembrane protein 1**	TMX1
0,000	2,28	4,86	**485,68**	Q9GZP4	**PITH domain-containing protein 1**	PITHD1
0,000	2,26	4,79	**478,99**	Q14061	**Cytochrome c oxidase copper chaperone**	COX17
0,024	2,25	4,77	**476,55**	Q8WUD4	**Coiled-coil domain-containing protein 12**	CCDC12
0,000	2,21	4,63	**462,68**	Q9BZE9	**Tether containing UBX domain for GLUT4**	ASPSCR1
0,037	2,18	4,53	**453,19**	Q13825	**Methylglutaconyl-CoA hydratase, mitochondrial**	AUH
0,043	2,18	4,52	**452,16**	Q676U5	**Autophagy-related protein 16-1**	ATG16L1
0,002	2,14	4,41	**440,67**	Q96Q89	**Kinesin-like protein KIF20B**	KIF20B
0,010	2,13	4,38	**437,72**	Q96EL3	**39S ribosomal protein L53, mitochondrial**	MRPL53
0,019	2,12	4,35	**434,69**	Q3YBR2	**Transforming growth factor beta regulator 1**	TBRG1
0,003	2,12	4,35	**434,69**	P46199	**Translation initiation factor IF-2, mitochondrial**	MTIF2
0,048	2,11	4,32	**431,69**	Q96FV2	**Secernin-2**	SCRN2
0,018	2,06	4,17	**416,99**	Q96PP8	**Guanylate-binding protein 5**	GBP5
0,040	2,04	4,11	**411,25**	P08670	**Vimentin**	VIM
0,006	2,03	4,08	**408,40**	Q96G01	**Protein bicaudal D homolog 1**	BICD1
0,014	2,02	4,06	**406,13**	Q96N67	**Dedicator of cytokinesis protein 7**	DOCK7
0,026	2,02	4,06	**405,58**	O60504	**Vinexin**	SORBS3
0,000	2,01	4,03	**402,78**	P30622	**CAP-Gly domain-containing linker protein 1**	CLIP1
0,046	1,99	3,97	**397,24**	Q9BQA1	**Methylosome protein 50**	WDR77
0,034	1,96	3,89	**389,06**	P80303	**Nucleobindin-2;Nesfatin-1**	NUCB2
0,048	1,95	3,86	**385,60**	Q9H2Y7	**Zinc finger protein 106**	ZNF106
0,013	1,93	3,81	**381,06**	Q96MW1	**Coiled-coil domain-containing protein 43**	CCDC43
0,013	1,92	3,78	**377,53**	Q9P246	**Stromal interaction molecule 2**	STIM2
0,015	1,90	3,73	**373,21**	Q9H2W6	**39S ribosomal protein L46, mitochondrial**	MRPL46
0,030	1,89	3,71	**370,64**	P52735	**Guanine nucleotide exchange factor VAV2**	VAV2
0,005	1,87	3,66	**366,46**	Q9NR30	**Nucleolar RNA helicase 2**	DDX21

0,012	1,87	3,66	**365,53**	P05166	**Propionyl-CoA carboxylase beta chain, mitochondrial**	PCCB
0,037	1,86	3,63	**362,84**	Q8N668	**COMM domain-containing protein 1**	COMMD1
0,040	1,85	3,60	**359,57**	P32780	**General transcription factor IIH subunit 1**	GTF2H1
0,006	1,83	3,55	**355,20**	P84098	**60S ribosomal protein L19**	RPL19
0,002	1,82	3,53	**353,08**	O75164	**Lysine-specific demethylase 4A**	KDM4A
0,005	1,81	3,51	**350,64**	Q7L2J0	**7SK snRNA methylphosphate capping enzyme**	MEPCE
0,036	1,81	3,51	**350,64**	Q8NDH3	**Probable aminopeptidase NPEPL1**	NPEPL1
0,007	1,80	3,48	**348,22**	Q9BYD1	**39S ribosomal protein L13, mitochondrial**	MRPL13
0,001	1,80	3,48	**348,22**	O75935	**Dynactin subunit 3**	DCTN3
0,020	1,79	3,47	**346,82**	P62072	**Mitochondrial import inner membrane translocase subunit Tim10**	TIMM10
0,034	1,79	3,47	**346,66**	Q9BRQ6	**MICOS complex subunit MIC25**	CHCHD6
0,001	1,78	3,43	**343,43**	Q13084	**39S ribosomal protein L28, mitochondrial**	MRPL28
0,001	1,74	3,34	**334,04**	Q5JTZ9	**Alanine--tRNA ligase, mitochondrial**	AARS2
0,037	1,72	3,30	**329,72**	P38935	**DNA-binding protein SMUBP-2**	IGHMBP2
0,011	1,71	3,27	**326,90**	P98179	**Putative RNA-binding protein 3**	RBM3
0,005	1,70	3,25	**324,90**	Q9NRV9	**Heme-binding protein 1**	HEBP1
0,012	1,67	3,19	**319,10**	Q96SY0	**von Willebrand factor A domain-containing protein 9**	VWA9
0,008	1,67	3,18	**318,21**	Q9NWU5	**39S ribosomal protein L22, mitochondrial**	MRPL22
0,031	1,66	3,16	**316,49**	Q9BVK6	**Transmembrane emp24 domain-containing protein 9**	TMED9
0,005	1,66	3,15	**315,22**	Q9H967	**WD repeat-containing protein 76**	WDR76
0,038	1,62	3,08	**308,33**	O60244	**Mediator of RNA polymerase II transcription subunit 14**	MED14
0,040	1,62	3,08	**308,25**	Q13541	**Eukaryotic translation initiation factor 4E-binding protein 1**	EIF4EBP1
0,006	1,62	3,07	**307,38**	Q9NYK5	**39S ribosomal protein L39, mitochondrial**	MRPL39
0,001	1,61	3,05	**305,25**	Q9ULA0	**Aspartyl aminopeptidase**	DNPEP
0,015	1,60	3,03	**303,47**	Q8WYP5	**Protein ELYS**	AHCTF1
0,001	1,60	3,03	**303,14**	Q93034	**Cullin-5**	CUL5
0,044	1,59	3,01	**300,69**	Q9P260	**LisH domain and HEAT repeat-containing protein KIAA1468**	KIAA1468
0,024	1,57	2,96	**296,18**	Q92766	**Ras-responsive element-binding protein 1**	RREB1
0,035	1,56	2,95	**294,85**	O95865	**N(G),N(G)-dimethylarginine dimethylaminohydrolase 2**	DDAH2
0,000	1,55	2,93	**292,82**	P34932	**Heat shock 70 kDa protein 4**	HSPA4
0,026	1,53	2,89	**288,79**	O00559	**Receptor-binding cancer antigen expressed on SiSo cells**	EBAG9
0,028	1,53	2,89	**288,79**	Q8IVH4	**Methylmalonic aciduria type A protein, mitochondrial**	MMAA
0,009	1,52	2,87	**286,50**	P20700	**Lamin-B1**	LMNB1

0,001	1,50	2,83	**282,84**	Q14203	**Dynactin subunit 1**	DCTN1
0,000	1,49	2,81	**280,89**	Q13561	**Dynactin subunit 2**	DCTN2
0,001	1,48	2,79	**278,95**	P38646	**Stress-70 protein, mitochondrial**	HSPA9
0,046	1,47	2,77	**277,02**	Q96A35	**39S ribosomal protein L24, mitochondrial**	MRPL24
0,017	1,47	2,77	**277,02**	Q16352	**Alpha-internexin**	INA
0,006	1,46	2,75	**275,11**	Q9GZL7	**Ribosome biogenesis protein WDR12**	WDR12
0,035	1,44	2,71	**271,32**	O75832	**26S proteasome non-ATPase regulatory subunit 10**	PSMD10
0,043	1,44	2,71	**271,24**	P61960	**Ubiquitin-fold modifier 1**	UFM1
0,010	1,43	2,69	**269,45**	P12268	**Inosine-5-monophosphate dehydrogenase 2**	IMPDH2
0,000	1,42	2,68	**267,59**	Q12765	**Secernin-1**	SCRN1
0,028	1,42	2,68	**267,59**	Q8NBZ0	**INO80 complex subunit E**	INO80E
0,012	1,40	2,65	**264,55**	O00515	**Ladinin-1**	LAD1
0,019	1,40	2,64	**264,36**	P61916	**Epididymal secretory protein E1**	NPC2
0,001	1,40	2,64	**263,90**	Q9BYD2	**39S ribosomal protein L9, mitochondrial**	MRPL9
0,002	1,37	2,58	**258,47**	Q9Y6G9	**Cytoplasmic dynein 1 light intermediate chain 1**	DYNC1LI1
0,004	1,37	2,58	**258,07**	Q9UKV3	**Apoptotic chromatin condensation inducer in the nucleus**	ACIN1
0,003	1,35	2,55	**255,24**	Q9Y305	**Acyl-coenzyme A thioesterase 9, mitochondrial**	ACOT9
0,001	1,35	2,55	**254,91**	P10768	**S-formylglutathione hydrolase**	ESD
0,010	1,34	2,53	**253,15**	Q9Y2S7	**Polymerase delta-interacting protein 2**	POLDIP2
0,000	1,33	2,51	**251,40**	P08727	**Keratin, type I cytoskeletal 19**	KRT19
0,001	1,32	2,50	**249,67**	O14744	**Protein arginine N-methyltransferase 5;Protein arginine N-methyltransferase 5, N-te**	PRMT5
0,042	1,32	2,50	**249,67**	P25789	**Proteasome subunit alpha type-4**	PSMA4
0,021	1,32	2,50	**249,67**	P49585;Q9Y5K3	**Choline-phosphate cytidylyltransferase A;Choline-phosphate cytidylyltransferase B**	PCYT1A;PCYT1B
0,001	1,31	2,48	**247,94**	O95171	**Sciellin**	SCEL
0,011	1,30	2,47	**246,70**	Q8NDT2	**Putative RNA-binding protein 15B**	RBM15B
0,036	1,30	2,46	**246,23**	Q9UL15	**BAG family molecular chaperone regulator 5**	BAG5
0,030	1,29	2,45	**244,53**	Q96EY8	**Cob(I)yrinic acid a,c-diamide adenosyltransferase, mitochondrial**	MMAB
0,015	1,28	2,43	**242,84**	Q9UJW0	**Dynactin subunit 4**	DCTN4
0,008	1,28	2,43	**242,84**	P11586	**C-1-tetrahydrofolate synthase, cytoplasmic;Methylenetetrahydrofolate dehydrogen**	MTHFD1
0,009	1,27	2,41	**241,16**	P51784	**Ubiquitin carboxyl-terminal hydrolase 11**	USP11
0,002	1,26	2,40	**240,07**	Q9H3H3	**UPF0696 protein C11orf68**	C11orf68
0,000	1,25	2,38	**237,84**	Q09666	**Neuroblast differentiation-associated protein AHNAK**	AHNAK
0,010	1,25	2,38	**237,84**	Q14137	**Ribosome biogenesis protein BOP1**	BOP1

0,004	1,24	2,36	**236,20**	Q96JH7	**Deubiquitinating protein VCIP135**	VCPIP1
0,001	1,24	2,36	**236,20**	O75323	**Protein NipSnap homolog 2**	GBAS
0,033	1,23	2,35	**235,20**	Q99729	**Heterogeneous nuclear ribonucleoprotein A/B**	HNRNPAB
0,001	1,23	2,35	**234,57**	Q06323	**Proteasome activator complex subunit 1**	PSME1
0,001	1,23	2,34	**234,25**	Q969Q0;P83881	**60S ribosomal protein L36a-like;60S ribosomal protein L36a**	RPL36AL;RPL36A
0,000	1,22	2,33	**232,95**	P33991	**DNA replication licensing factor MCM4**	MCM4
0,001	1,21	2,31	**231,34**	P02794	**Ferritin heavy chain;Ferritin heavy chain, N-terminally processed**	FTH1
0,000	1,20	2,30	**229,91**	P52789	**Hexokinase-2**	HK2
0,007	1,19	2,28	**228,28**	O76021	**Ribosomal L1 domain-containing protein 1**	RSL1D1
0,001	1,18	2,27	**226,58**	Q9NVN8	**Guanine nucleotide-binding protein-like 3-like protein**	GNL3L
0,035	1,18	2,27	**226,58**	Q9H3P7	**Golgi resident protein GCP60**	ACBD3
0,038	1,18	2,27	**226,58**	O00743	**Serine/threonine-protein phosphatase 6 catalytic subunit;Serine/threonine-protein**	PPP6C
0,008	1,17	2,25	**225,01**	Q9BYD6	**39S ribosomal protein L1, mitochondrial**	MRPL1
0,001	1,16	2,23	**223,46**	Q04726	**Transducin-like enhancer protein 3**	TLE3
0,000	1,16	2,23	**223,46**	Q15020	**Squamous cell carcinoma antigen recognized by T-cells 3**	SART3
0,007	1,16	2,23	**223,46**	Q13938	**Calcyphosin**	CAPS
0,028	1,14	2,21	**221,03**	P48163	**NADP-dependent malic enzyme**	ME1
0,000	1,14	2,20	**220,38**	Q14566	**DNA replication licensing factor MCM6**	MCM6
0,006	1,14	2,20	**220,38**	Q8TE68	**Epidermal growth factor receptor kinase substrate 8-like protein 1**	EPS8L1
0,018	1,13	2,20	**219,58**	P06703	**Protein S100-A6**	S100A6
0,006	1,13	2,19	**218,86**	P61201	**COP9 signalosome complex subunit 2**	COPS2
0,000	1,13	2,19	**218,86**	O60437	**Periplakin**	PPL
0,007	1,12	2,17	**217,37**	Q96C57	**Uncharacterized protein C12orf43**	C12orf43
0,021	1,12	2,17	**217,35**	Q9ULJ3	**Zinc finger and BTB domain-containing protein 21**	ZBTB21
0,041	1,12	2,17	**217,35**	Q9Y5V0	**Zinc finger protein 706**	ZNF706
0,046	1,12	2,17	**217,35**	Q16555	**Dihydropyrimidinase-related protein 2**	DPYSL2
0,015	1,12	2,17	**217,35**	P05455	**Lupus La protein**	SSB
0,001	1,11	2,16	**215,85**	P82914	**28S ribosomal protein S15, mitochondrial**	MRPS15
0,000	1,11	2,16	**215,85**	Q15149	**Plectin**	PLEC
0,024	1,11	2,16	**215,85**	Q99627	**COP9 signalosome complex subunit 8**	COPS8
0,024	1,11	2,16	**215,85**	Q92890	**Ubiquitin fusion degradation protein 1 homolog**	UFD1L
0,034	1,11	2,15	**215,46**	Q7Z6B7	**SLIT-ROBO Rho GTPase-activating protein 1**	SRGAP1
0,003	1,10	2,14	**214,35**	Q9Y5U2	**Protein TSSC4**	TSSC4

0,017	1,09	2,13	**212,87**	P47985	**Cytochrome b-c1 complex subunit Rieske, mitochondrial;Cytochrome b-c1 complex**	UQCRFS1
0,035	1,09	2,13	**212,87**	P56747	**Claudin-6**	CLDN6
0,050	1,09	2,13	**212,87**	Q15785	**Mitochondrial import receptor subunit TOM34**	TOMM34
0,002	1,09	2,13	**212,87**	P52815	**39S ribosomal protein L12, mitochondrial**	MRPL12
0,000	1,09	2,13	**212,87**	P55072	**Transitional endoplasmic reticulum ATPase**	VCP
0,026	1,09	2,13	**212,87**	O75525	**KH domain-containing, RNA-binding, signal transduction-associated protein 3**	KHDRBS3
0,000	1,09	2,13	**212,87**	O14530	**Thioredoxin domain-containing protein 9**	TXNDC9
0,000	1,09	2,13	**212,87**	Q9Y3C1	**Nucleolar protein 16**	NOP16
0,047	1,09	2,13	**212,87**	Q9Y6M9	**NADH dehydrogenase [ubiquinone] 1 beta subcomplex subunit 9**	NDUFB9
0,000	1,08	2,11	**211,40**	P25205	**DNA replication licensing factor MCM3**	MCM3
0,003	1,07	2,10	**209,94**	Q96D46	**60S ribosomal export protein NMD3**	NMD3
0,000	1,07	2,10	**209,94**	Q92817	**Envoplakin**	EVPL
0,000	1,06	2,08	**208,49**	Q9HCS7	**Pre-mRNA-splicing factor SYF1**	XAB2
0,020	1,06	2,08	**208,49**	Q7LBC6	**Lysine-specific demethylase 3B**	KDM3B
0,012	1,06	2,08	**207,94**	P46821	**Microtubule-associated protein 1B;MAP1B heavy chain;MAP1 light chain LC1**	MAP1B
0,011	1,05	2,07	**207,05**	Q8TD16	**Protein bicaudal D homolog 2**	BICD2
0,013	1,04	2,06	**205,62**	Q9NRX2	**39S ribosomal protein L17, mitochondrial**	MRPL17
0,007	1,04	2,06	**205,62**	Q9P253	**Vacuolar protein sorting-associated protein 18 homolog**	VPS18
0,002	1,04	2,06	**205,62**	Q9Y2Z0	**Suppressor of G2 allele of SKP1 homolog**	SUGT1
0,030	1,04	2,06	**205,62**	Q16851	**UTP--glucose-1-phosphate uridylyltransferase**	UGP2
0,008	1,04	2,06	**205,62**	Q8TAT6	**Nuclear protein localization protein 4 homolog**	NPLOC4
0,000	1,04	2,06	**205,62**	Q92598	**Heat shock protein 105 kDa**	HSPH1
0,000	1,03	2,04	**204,20**	Q02539	**Histone H1.1**	HIST1H1A
0,017	1,03	2,04	**204,20**	Q16401	**26S proteasome non-ATPase regulatory subunit 5**	PSMD5
0,002	1,03	2,04	**204,20**	P43034	**Platelet-activating factor acetylhydrolase IB subunit alpha**	PAFAH1B1
0,004	1,02	2,03	**202,79**	P09001	**39S ribosomal protein L3, mitochondrial**	MRPL3
0,022	1,01	2,01	**201,39**	P10155	**60 kDa SS-A/Ro ribonucleoprotein**	TROVE2
0,016	1,01	2,01	**201,39**	Q00577	**Transcriptional activator protein Pur-alpha**	PURA
0,034	1,01	2,01	**201,39**	Q9NQV6	**PR domain zinc finger protein 10**	PRDM10
0,019	1,00	2,00	**200,33**	Q9H5X1	**MIP18 family protein FAM96A**	FAM96A
0,010	1,00	2,00	**199,69**	O43716	**Glutamyl-tRNA(Gln) amidotransferase subunit C, mitochondrial**	GATC
0,001	0,99	1,99	**198,62**	Q99700	**Ataxin-2**	ATXN2
0,002	0,99	1,99	**198,62**	Q7L5N1	**COP9 signalosome complex subunit 6**	COPS6

0,009	0,98	1,98	**197,52**	Q9H269	**Vacuolar protein sorting-associated protein 16 homolog**	VPS16
0,001	0,98	1,97	**197,48**	P32929	**Cystathionine gamma-lyase**	CTH
0,003	0,98	1,97	**197,25**	P49411	**Elongation factor Tu, mitochondrial**	TUFM
0,000	0,98	1,97	**197,25**	P33992	**DNA replication licensing factor MCM5**	MCM5
0,000	0,98	1,97	**197,25**	P07900	**Heat shock protein HSP 90-alpha**	HSP90AA1
0,003	0,97	1,96	**195,88**	P05783	**Keratin, type I cytoskeletal 18**	KRT18
0,002	0,97	1,96	**195,88**	Q9UL40	**Zinc finger protein 346**	ZNF346
0,010	0,97	1,95	**195,32**	Q9NS69	**Mitochondrial import receptor subunit TOM22 homolog**	TOMM22
0,005	0,96	1,95	**194,95**	Q16629	**Serine/arginine-rich splicing factor 7**	SRSF7
0,044	0,96	1,95	**194,53**	Q96RP9	**Elongation factor G, mitochondrial**	GFM1
0,049	0,96	1,95	**194,53**	Q92989	**Polyribonucleotide 5-hydroxyl-kinase Clp1**	CLP1
0,017	0,95	1,93	**193,19**	Q5VZK9	**Leucine-rich repeat-containing protein 16A**	LRRC16A
0,040	0,95	1,93	**193,19**	Q02790	**Peptidyl-prolyl cis-trans isomerase FKBP4;Peptidyl-prolyl cis-trans isomerase FKBP4,**	FKBP4
0,031	0,95	1,93	**193,19**	Q8NCH0	**Carbohydrate sulfotransferase 14**	CHST14
0,007	0,94	1,92	**191,85**	Q16881	**Thioredoxin reductase 1, cytoplasmic**	TXNRD1
0,008	0,94	1,92	**191,85**	Q14204	**Cytoplasmic dynein 1 heavy chain 1**	DYNC1H1
0,022	0,94	1,92	**191,85**	Q9Y3B7	**39S ribosomal protein L11, mitochondrial**	MRPL11
0,006	0,93	1,91	**190,53**	P41250	**Glycine--tRNA ligase**	GARS
0,014	0,93	1,91	**190,53**	O14745	**Na(+)/H(+) exchange regulatory cofactor NHE-RF1**	SLC9A3R1
0,012	0,93	1,91	**190,53**	P50750	**Cyclin-dependent kinase 9**	CDK9
0,047	0,93	1,91	**190,53**	Q9NW08	**DNA-directed RNA polymerase III subunit RPC2**	POLR3B
0,044	0,93	1,91	**190,53**	Q969S3	**Zinc finger protein 622**	ZNF622
0,026	0,92	1,90	**189,67**	P33764	**Protein S100-A3**	S100A3
0,013	0,92	1,89	**189,21**	Q8TBX8	**Phosphatidylinositol 5-phosphate 4-kinase type-2 gamma**	PIP4K2C
0,001	0,91	1,88	**187,90**	Q9BT78	**COP9 signalosome complex subunit 4**	COPS4
0,018	0,91	1,88	**187,90**	P51608	**Methyl-CpG-binding protein 2**	MECP2
0,034	0,91	1,88	**187,90**	P25787	**Proteasome subunit alpha type-2**	PSMA2
0,007	0,91	1,88	**187,90**	Q9HCY8	**Protein S100-A14**	S100A14
0,004	0,90	1,87	**186,61**	Q14197	**Peptidyl-tRNA hydrolase ICT1, mitochondrial**	ICT1
0,046	0,90	1,87	**186,58**	Q5SW79	**Centrosomal protein of 170 kDa**	CEP170
0,001	0,89	1,86	**185,57**	Q9Y2X3	**Nucleolar protein 58**	NOP58
0,000	0,87	1,83	**182,93**	P39060	**Collagen alpha-1(XVIII) chain;Endostatin**	COL18A1
0,035	0,87	1,83	**182,86**	Q9UNH6	**Sorting nexin-7**	SNX7

0,030	0,87	1,83	**182,77**	Q9H9B4	**Sideroflexin-1**	SFXN1
0,001	0,87	1,83	**182,77**	P17066;P48741	**Heat shock 70 kDa protein 6;Putative heat shock 70 kDa protein 7**	HSPA6;HSPA7
0,029	0,86	1,82	**181,50**	P08754	**Guanine nucleotide-binding protein G(k) subunit alpha**	GNAI3
0,039	0,85	1,80	**180,25**	Q86TI2	**Dipeptidyl peptidase 9**	DPP9
0,000	0,85	1,80	**180,25**	P33993	**DNA replication licensing factor MCM7**	MCM7
0,004	0,84	1,79	**179,01**	Q93009	**Ubiquitin carboxyl-terminal hydrolase 7**	USP7
0,043	0,83	1,78	**177,91**	Q9H307	**Pinin**	PNN
0,001	0,83	1,78	**177,77**	P13639	**Elongation factor 2**	EEF2
0,029	0,83	1,78	**177,77**	P50213	**Isocitrate dehydrogenase [NAD] subunit alpha, mitochondrial**	IDH3A
0,000	0,83	1,78	**177,77**	P31948	**Stress-induced-phosphoprotein 1**	STIP1
0,002	0,83	1,78	**177,77**	P25786	**Proteasome subunit alpha type-1**	PSMA1
0,004	0,83	1,78	**177,77**	Q9UBW8	**COP9 signalosome complex subunit 7a**	COPS7A
0,001	0,82	1,77	**176,54**	P15927	**Replication protein A 32 kDa subunit**	RPA2
0,000	0,82	1,77	**176,54**	P49736	**DNA replication licensing factor MCM2**	MCM2
0,003	0,82	1,77	**176,54**	P28066	**Proteasome subunit alpha type-5**	PSMA5
0,002	0,82	1,77	**176,54**	Q9HCC0	**Methylcrotonoyl-CoA carboxylase beta chain, mitochondrial**	MCCC2
0,037	0,81	1,76	**175,93**	Q8IYB8	**ATP-dependent RNA helicase SUPV3L1, mitochondrial**	SUPV3L1
0,022	0,81	1,75	**175,42**	O94906	**Pre-mRNA-processing factor 6**	PRPF6
0,025	0,81	1,75	**175,18**	Q9Y2U5;Q99759	**Mitogen-activated protein kinase kinase kinase 2;Mitogen-activated protein kinase**	MAP3K2;MAP3K3
0,024	0,81	1,75	**175,00**	Q07955	**Serine/arginine-rich splicing factor 1**	SRSF1
0,003	0,80	1,74	**174,11**	O94992	**Protein HEXIM1**	HEXIM1
0,018	0,80	1,74	**173,95**	P09651;Q32P51	**Heterogeneous nuclear ribonucleoprotein A1;Heterogeneous nuclear ribonucleopro**	VRNPA1;HNRNPA1I
0,006	0,80	1,74	**173,83**	P49589	**Cysteine--tRNA ligase, cytoplasmic**	CARS
0,030	0,80	1,74	**173,61**	P38159	**RNA-binding motif protein, X chromosome;RNA-binding motif protein, X chromosor**	RBMX
0,005	0,79	1,73	**172,91**	Q9Y4C8	**Probable RNA-binding protein 19**	RBM19
0,038	0,78	1,72	**171,95**	Q5SNT6;Q641Q2;Q9Y4E	**WASH complex subunit FAM21B;WASH complex subunit FAM21A;WASH complex su**	121B;FAM21A;FAM
0,029	0,78	1,72	**171,89**	P04279	**Semenogelin-1;Alpha-inhibin-92;Alpha-inhibin-31;Seminal basic protein**	SEMG1
0,003	0,78	1,72	**171,77**	Q13247	**Serine/arginine-rich splicing factor 6**	SRSF6
0,045	0,78	1,72	**171,71**	P82673	**28S ribosomal protein S35, mitochondrial**	MRPS35
0,010	0,78	1,72	**171,71**	Q9UKD1	**Glucocorticoid modulatory element-binding protein 2**	GMEB2
0,000	0,78	1,72	**171,71**	P11142	**Heat shock cognate 71 kDa protein**	HSPA8
0,009	0,77	1,71	**170,53**	Q9Y5K5	**Ubiquitin carboxyl-terminal hydrolase isozyme L5**	UCHL5
0,040	0,76	1,69	**169,42**	Q96A26	**Protein FAM162A**	FAM162A

0,003	0,76	1,69	**169,35**	P62333	**26S protease regulatory subunit 10B**	PSMC6
0,000	0,76	1,69	**169,35**	P50502;Q8NFI4;Q8IZP2	**Hsc70-interacting protein;Putative protein FAM10A5;Putative protein FAM10A4**	T13;ST13P5;ST13P
0,018	0,76	1,69	**169,35**	Q8N2M8	**CLK4-associating serine/arginine rich protein**	CLASRP
0,000	0,76	1,69	**169,35**	P51149	**Ras-related protein Rab-7a**	RAB7A
0,026	0,76	1,69	**169,29**	Q96JC1	**Vam6/Vps39-like protein**	VPS39
0,009	0,75	1,68	**168,18**	P40227	**T-complex protein 1 subunit zeta**	CCT6A
0,027	0,75	1,68	**168,18**	Q9Y676	**28S ribosomal protein S18b, mitochondrial**	MRPS18B
0,043	0,75	1,68	**168,18**	Q8WZA0	**Protein LZIC**	LZIC
0,030	0,75	1,68	**168,18**	O43681	**ATPase ASNA1**	ASNA1
0,045	0,75	1,68	**168,18**	Q9NX24	**H/ACA ribonucleoprotein complex subunit 2**	NHP2
0,007	0,73	1,66	**165,86**	Q9UL46	**Proteasome activator complex subunit 2**	PSME2
0,017	0,73	1,66	**165,86**	P60510	**Serine/threonine-protein phosphatase 4 catalytic subunit**	PPP4C
0,011	0,73	1,66	**165,86**	Q13426	**DNA repair protein XRCC4**	XRCC4
0,006	0,73	1,66	**165,86**	O43395	**U4/U6 small nuclear ribonucleoprotein Prp3**	PRPF3
0,001	0,73	1,66	**165,72**	P08195	**4F2 cell-surface antigen heavy chain**	SLC3A2
0,015	0,72	1,65	**164,72**	P29590	**Protein PML**	PML
0,017	0,72	1,65	**164,72**	P12532	**Creatine kinase U-type, mitochondrial**	CKMT1A
0,046	0,71	1,64	**163,85**	P48723	**Heat shock 70 kDa protein 13**	HSPA13
0,000	0,71	1,64	**163,58**	P20618	**Proteasome subunit beta type-1**	PSMB1
0,001	0,70	1,62	**162,45**	P25788	**Proteasome subunit alpha type-3**	PSMA3
0,002	0,70	1,62	**162,45**	P49721	**Proteasome subunit beta type-2**	PSMB2
0,038	0,69	1,61	**161,33**	Q13619	**Cullin-4A**	CUL4A
0,004	0,69	1,61	**161,33**	Q9Y2R9	**28S ribosomal protein S7, mitochondrial**	MRPS7
0,037	0,68	1,60	**160,21**	Q00059	**Transcription factor A, mitochondrial**	TFAM
0,025	0,68	1,60	**160,21**	P62316	**Small nuclear ribonucleoprotein Sm D2**	SNRPD2
0,022	0,68	1,60	**160,21**	Q07021	**Complement component 1 Q subcomponent-binding protein, mitochondrial**	C1QBP
0,001	0,68	1,60	**160,21**	Q9NW13	**RNA-binding protein 28**	RBM28
0,000	0,68	1,60	**160,21**	P28070	**Proteasome subunit beta type-4**	PSMB4
0,026	0,68	1,60	**160,21**	P15408	**Fos-related antigen 2**	FOSL2
0,002	0,67	1,59	**159,11**	O14818	**Proteasome subunit alpha type-7**	PSMA7
0,040	0,67	1,59	**159,11**	O43719	**HIV Tat-specific factor 1**	HTATSF1
0,011	0,66	1,58	**158,07**	Q14116	**Interleukin-18**	IL18
0,000	0,66	1,58	**158,01**	P27694	**Replication protein A 70 kDa DNA-binding subunit;Replication protein A 70 kDa DN**	RPA1

0,028	0,66	1,58	**158,01**	Q9NRA8	**Eukaryotic translation initiation factor 4E transporter**	EIF4ENIF1
0,002	0,66	1,58	**158,01**	Q99436	**Proteasome subunit beta type-7**	PSMB7
0,000	0,66	1,58	**158,01**	P08107	**Heat shock 70 kDa protein 1A/1B**	HSPA1A
0,041	0,66	1,58	**158,01**	Q9Y399	**28S ribosomal protein S2, mitochondrial**	MRPS2
0,037	0,65	1,57	**157,38**	P07910	**Heterogeneous nuclear ribonucleoproteins C1/C2**	HNRNPC
0,289	0,65	1,57	**157,24**	O00767	**Acyl-CoA desaturase**	SCD
0,012	0,63	1,55	**154,76**	O95456	**Proteasome assembly chaperone 1**	PSMG1
0,002	0,63	1,55	**154,76**	O15355	**Protein phosphatase 1G**	PPM1G
0,034	0,63	1,55	**154,76**	P62877	**E3 ubiquitin-protein ligase RBX1;E3 ubiquitin-protein ligase RBX1, N-terminally proc**	RBX1
0,016	0,63	1,55	**154,76**	Q02040	**A-kinase anchor protein 17A**	AKAP17A
0,037	0,62	1,54	**153,69**	Q8NF91	**Nesprin-1**	SYNE1
0,002	0,62	1,54	**153,69**	P62191	**26S protease regulatory subunit 4**	PSMC1
0,014	0,61	1,53	**152,63**	P07919	**Cytochrome b-c1 complex subunit 6, mitochondrial**	UQCRH
0,010	0,60	1,52	**151,57**	P28072	**Proteasome subunit beta type-6**	PSMB6
0,001	0,60	1,52	**151,57**	P17980	**26S protease regulatory subunit 6A**	PSMC3
0,003	0,60	1,52	**151,57**	P17987	**T-complex protein 1 subunit alpha**	TCP1
0,027	0,60	1,51	**151,36**	Q9BZJ0	**Crooked neck-like protein 1**	CRNKL1
0,034	0,59	1,51	**150,88**	Q05BQ5	**MBT domain-containing protein 1**	MBTD1
0,014	0,59	1,51	**150,83**	O95834	**Echinoderm microtubule-associated protein-like 2**	EML2
0,002	0,59	1,51	**150,52**	O75381	**Peroxisomal membrane protein PEX14**	PEX14
0,000	0,59	1,51	**150,52**	P35998	**26S protease regulatory subunit 7**	PSMC2
0,043	0,58	1,49	**149,48**	P25685	**DnaJ homolog subfamily B member 1**	DNAJB1
0,033	0,58	1,49	**149,48**	P42704	**Leucine-rich PPR motif-containing protein, mitochondrial**	LRPPRC
0,005	0,56	1,48	**147,61**	P29317	**Ephrin type-A receptor 2**	EPHA2
0,010	0,56	1,47	**147,43**	O00232	**26S proteasome non-ATPase regulatory subunit 12**	PSMD12
0,006	0,56	1,47	**147,43**	P55036	**26S proteasome non-ATPase regulatory subunit 4**	PSMD4
0,015	0,56	1,47	**147,04**	P67936	**Tropomyosin alpha-4 chain**	TPM4
0,041	0,55	1,46	**146,41**	O00170	**AH receptor-interacting protein**	AIP
0,016	0,55	1,46	**146,41**	P28074	**Proteasome subunit beta type-5**	PSMB5
0,000	0,54	1,45	**145,40**	P13995	**Bifunctional methylenetetrahydrofolate dehydrogenase/cyclohydrolase, mitochond**	MTHFD2
0,016	0,54	1,45	**145,40**	Q04837	**Single-stranded DNA-binding protein, mitochondrial**	SSBP1
0,013	0,54	1,45	**145,40**	P68400;Q8NEV1	**Casein kinase II subunit alpha;Casein kinase II subunit alpha 3**	CSNK2A1;CSNK2A3
0,003	0,53	1,44	**144,39**	P49368	**T-complex protein 1 subunit gamma**	CCT3

0,035	0,53	1,44	**144,39**	Q8N556	**Actin filament-associated protein 1**	AFAP1
0,012	0,53	1,44	**144,39**	Q9H6R0	**Putative ATP-dependent RNA helicase DHX33**	DHX33
0,012	0,53	1,44	**144,39**	Q9UL63	**Muskelin**	MKLN1
0,010	0,53	1,44	**144,39**	Q9UBP9	**PTB domain-containing engulfment adapter protein 1**	GULP1
0,019	0,52	1,43	**143,40**	P08621	**U1 small nuclear ribonucleoprotein 70 kDa**	SNRNP70
0,003	0,52	1,43	**143,40**	P31942	**Heterogeneous nuclear ribonucleoprotein H3**	HNRNPH3
0,029	0,51	1,42	**142,41**	Q9Y6X4	**Soluble lamin-associated protein of 75 kDa**	FAM169A
0,003	0,51	1,42	**142,41**	Q9UJZ1	**Stomatin-like protein 2, mitochondrial**	STOML2
0,036	0,50	1,41	**141,42**	O95232	**Luc7-like protein 3**	LUC7L3
0,000	0,50	1,41	**141,42**	Q13263	**Transcription intermediary factor 1-beta**	TRIM28
0,016	0,50	1,41	**141,42**	Q15424	**Scaffold attachment factor B1**	SAFB
0,003	0,50	1,41	**141,42**	Q99733	**Nucleosome assembly protein 1-like 4**	NAP1L4
0,000	0,50	1,41	**141,42**	Q8N163	**Cell cycle and apoptosis regulator protein 2**	CCAR2
0,004	0,50	1,41	**141,42**	P53396	**ATP-citrate synthase**	ACLY
0,018	0,50	1,41	**141,42**	P52907	**F-actin-capping protein subunit alpha-1**	CAPZA1
0,001	0,49	1,40	**140,44**	P56537	**Eukaryotic translation initiation factor 6**	EIF6
0,022	0,49	1,40	**140,44**	Q13217	**DnaJ homolog subfamily C member 3**	DNAJC3
0,002	0,49	1,40	**140,44**	P78332	**RNA-binding protein 6**	RBM6
0,019	0,49	1,40	**140,44**	Q96S82	**Ubiquitin-like protein 7**	UBL7
0,017	0,49	1,40	**140,44**	P50552	**Vasodilator-stimulated phosphoprotein**	VASP
0,018	0,49	1,40	**140,44**	P28062	**Proteasome subunit beta type-8**	PSMB8
0,021	0,48	1,40	**139,93**	Q96L91	**E1A-binding protein p400**	EP400
0,004	0,48	1,39	**139,47**	Q9NP79	**Vacuolar protein sorting-associated protein VTA1 homolog**	VTA1
0,005	0,47	1,39	**138,51**	P07355;A6NMY6	**Annexin A2;Putative annexin A2-like protein**	ANXA2;ANXA2P2
0,034	0,47	1,39	**138,51**	Q96JY6	**PDZ and LIM domain protein 2**	PDLIM2
0,031	0,47	1,39	**138,51**	Q9Y221	**60S ribosome subunit biogenesis protein NIP7 homolog**	NIP7
0,000	0,47	1,39	**138,51**	Q15008	**26S proteasome non-ATPase regulatory subunit 6**	PSMD6
0,007	0,47	1,39	**138,51**	Q9UHB6	**LIM domain and actin-binding protein 1**	LIMA1
0,042	0,47	1,39	**138,51**	Q6PI48	**Aspartate--tRNA ligase, mitochondrial**	DARS2
0,001	0,47	1,39	**138,51**	P51665	**26S proteasome non-ATPase regulatory subunit 7**	PSMD7
0,027	0,47	1,39	**138,51**	Q96GQ7	**Probable ATP-dependent RNA helicase DDX27**	DDX27
0,028	0,47	1,38	**138,31**	Q66K74	**Microtubule-associated protein 1S;MAP1S heavy chain;MAP1S light chain**	MAP1S
0,004	0,46	1,38	**137,55**	Q15717	**ELAV-like protein 1**	ELAVL1

0,045	0,46	1,38	**137,55**	Q9UKK9	**ADP-sugar pyrophosphatase**	NUDT5
0,048	0,45	1,37	**136,62**	Q13769	**THO complex subunit 5 homolog**	THOC5
0,015	0,45	1,37	**136,60**	P08579	**U2 small nuclear ribonucleoprotein B**	SNRPB2
0,041	0,45	1,37	**136,60**	Q8IUD2	**ELKS/Rab6-interacting/CAST family member 1**	ERC1
0,010	0,45	1,37	**136,60**	Q9BWU0	**Kanadaptin**	SLC4A1AP
0,035	0,44	1,36	**135,91**	P02545	**Prelamin-A/C;Lamin-A/C**	LMNA
0,000	0,44	1,36	**135,66**	P62195	**26S protease regulatory subunit 8**	PSMC5
0,020	0,44	1,36	**135,66**	P00367	**Glutamate dehydrogenase 1, mitochondrial**	GLUD1
0,029	0,44	1,36	**135,66**	Q16527	**Cysteine and glycine-rich protein 2**	CSRP2
0,013	0,44	1,36	**135,66**	P48643	**T-complex protein 1 subunit epsilon**	CCT5
0,006	0,44	1,36	**135,66**	Q99460	**26S proteasome non-ATPase regulatory subunit 1**	PSMD1
0,001	0,44	1,36	**135,66**	Q4G0J3	**La-related protein 7**	LARP7
0,019	0,43	1,35	**134,79**	P05387	**60S acidic ribosomal protein P2**	RPLP2
0,010	0,43	1,35	**134,72**	O43818	**U3 small nucleolar RNA-interacting protein 2**	RRP9
0,034	0,43	1,35	**134,72**	P08238	**Heat shock protein HSP 90-beta**	HSP90AB1
0,025	0,43	1,35	**134,72**	Q13098	**COP9 signalosome complex subunit 1**	GPS1
0,013	0,41	1,33	**133,07**	Q15366	**Poly(rC)-binding protein 2**	PCBP2
0,011	0,41	1,33	**132,87**	O43765	**Small glutamine-rich tetratricopeptide repeat-containing protein alpha**	SGTA
0,002	0,40	1,32	**132,04**	Q13242	**Serine/arginine-rich splicing factor 9**	SRSF9
0,041	0,39	1,31	**131,04**	Q9UNP9	**Peptidyl-prolyl cis-trans isomerase E**	PPIE
0,010	0,39	1,31	**131,04**	P51991	**Heterogeneous nuclear ribonucleoprotein A3**	HNRNPA3
0,001	0,39	1,31	**131,04**	O43823	**A-kinase anchor protein 8**	AKAP8
0,000	0,39	1,31	**131,04**	P48556	**26S proteasome non-ATPase regulatory subunit 8**	PSMD8
0,019	0,39	1,31	**131,04**	P52298	**Nuclear cap-binding protein subunit 2**	NCBP2
0,007	0,38	1,30	**130,13**	O60716	**Catenin delta-1**	CTNND1
0,001	0,38	1,30	**130,13**	P22626	**Heterogeneous nuclear ribonucleoproteins A2/B1**	HNRNPA2B1
0,015	0,38	1,30	**130,13**	Q96IR2;A2RRD8	**Zinc finger protein 845;Zinc finger protein 320**	ZNF845;ZNF320
0,007	0,37	1,29	**129,24**	P50990	**T-complex protein 1 subunit theta**	CCT8
0,023	0,37	1,29	**129,20**	Q9BYG3	**MKI67 FHA domain-interacting nucleolar phosphoprotein**	NIFK
0,038	0,36	1,29	**128,74**	Q9UBS4	**DnaJ homolog subfamily B member 11**	DNAJB11
0,023	0,36	1,28	**128,42**	Q01813	**ATP-dependent 6-phosphofructokinase, platelet type**	PFKP
0,004	0,36	1,28	**128,34**	Q9H0L4	**Cleavage stimulation factor subunit 2 tau variant**	CSTF2T
0,006	0,36	1,28	**128,34**	Q9NRL3	**Striatin-4**	STRN4

0,006	0,36	1,28	**128,34**	O00231	**26S proteasome non-ATPase regulatory subunit 11**	PSMD11
0,023	0,35	1,27	**127,46**	Q13951	**Core-binding factor subunit beta**	CBFB
0,020	0,35	1,27	**127,46**	Q9UHX1	**Poly(U)-binding-splicing factor PUF60**	PUF60
0,001	0,35	1,27	**127,46**	P15924	**Desmoplakin**	DSP
0,046	0,34	1,27	**126,58**	Q13952	**Nuclear transcription factor Y subunit gamma**	NFYC
0,005	0,34	1,27	**126,58**	Q99832	**T-complex protein 1 subunit eta**	CCT7
0,027	0,34	1,27	**126,58**	P49750	**YLP motif-containing protein 1**	YLPM1
0,023	0,33	1,26	**125,70**	Q14690	**Protein RRP5 homolog**	PDCD11
0,007	0,33	1,26	**125,70**	P43686	**26S protease regulatory subunit 6B**	PSMC4
0,022	0,33	1,26	**125,70**	Q9NZL9	**Methionine adenosyltransferase 2 subunit beta**	MAT2B
0,006	0,33	1,26	**125,70**	Q01130	**Serine/arginine-rich splicing factor 2**	SRSF2
0,026	0,32	1,25	**124,83**	P35579	**Myosin-9**	MYH9
0,030	0,32	1,25	**124,83**	P20810	**Calpastatin**	CAST
0,034	0,32	1,25	**124,83**	O75880	**Protein SCO1 homolog, mitochondrial**	SCO1
0,015	0,31	1,24	**123,97**	P67870	**Casein kinase II subunit beta**	CSNK2B
0,026	0,31	1,24	**123,97**	Q8IX12	**Cell division cycle and apoptosis regulator protein 1**	CCAR1
0,002	0,31	1,24	**123,66**	O43707	**Alpha-actinin-4**	ACTN4
0,032	0,30	1,23	**123,33**	P19338	**Nucleolin**	NCL
0,040	0,30	1,23	**123,32**	P00558	**Phosphoglycerate kinase 1**	PGK1
0,028	0,30	1,23	**123,11**	P46087	**Probable 28S rRNA (cytosine(4447)-C(5))-methyltransferase**	NOP2
0,030	0,29	1,22	**122,26**	Q9Y6M1	**Insulin-like growth factor 2 mRNA-binding protein 2**	IGF2BP2
0,048	0,29	1,22	**122,26**	O15160	**DNA-directed RNA polymerases I and III subunit RPAC1**	POLR1C
0,008	0,28	1,21	**121,42**	Q8IZ69	**tRNA (uracil-5-)-methyltransferase homolog A**	TRMT2A
0,033	0,28	1,21	**121,42**	Q9UQE7	**Structural maintenance of chromosomes protein 3**	SMC3
0,033	0,28	1,21	**121,06**	P15311	**Ezrin**	EZR
0,007	0,27	1,21	**120,63**	P11021	**78 kDa glucose-regulated protein**	HSPA5
0,013	0,27	1,21	**120,58**	P09874	**Poly [ADP-ribose] polymerase 1**	PARP1
0,016	0,27	1,20	**120,27**	Q13442	**28 kDa heat- and acid-stable phosphoprotein**	PDAP1
0,001	0,26	1,20	**119,75**	Q12906	**Interleukin enhancer-binding factor 3**	ILF3
0,033	0,25	1,19	**118,92**	O43242	**26S proteasome non-ATPase regulatory subunit 3**	PSMD3
0,030	0,25	1,19	**118,90**	P23528	**Cofilin-1**	CFL1
0,027	0,24	1,18	**118,46**	P23284	**Peptidyl-prolyl cis-trans isomerase B**	PPIB
0,043	0,24	1,18	**118,30**	Q9Y617	**Phosphoserine aminotransferase**	PSAT1

0,006	0,24	1,18	**117,74**	Q8N1G4	**Leucine-rich repeat-containing protein 47**	LRRC47
0,029	0,24	1,18	**117,73**	O76094	**Signal recognition particle subunit SRP72**	SRP72
0,012	0,23	1,17	**117,03**	P04083	**Annexin A1**	ANXA1
0,042	0,23	1,17	**117,00**	Q9BRP8	**Partner of Y14 and mago**	WIBG
0,038	0,22	1,17	**116,75**	Q14103	**Heterogeneous nuclear ribonucleoprotein D0**	HNRNPD
0,042	0,22	1,17	**116,67**	P06753	**Tropomyosin alpha-3 chain**	TPM3
0,002	0,22	1,16	**116,47**	P35580	**Myosin-10**	MYH10
0,002	0,22	1,16	**116,47**	Q15029	**116 kDa U5 small nuclear ribonucleoprotein component**	EFTUD2
0,030	0,21	1,16	**115,83**	P62241	**40S ribosomal protein S8**	RPS8
0,005	0,21	1,16	**115,67**	P55769	**NHP2-like protein 1;NHP2-like protein 1, N-terminally processed**	NHP2L1
0,013	0,19	1,14	**113,77**	O60307	**Microtubule-associated serine/threonine-protein kinase 3**	MAST3
0,033	0,18	1,13	**113,29**	Q6VN20	**Ran-binding protein 10**	RANBP10
0,040	0,17	1,12	**112,31**	P36578	**60S ribosomal protein L4**	RPL4
0,039	0,16	1,12	**111,94**	P62750	**60S ribosomal protein L23a**	RPL23A
0,012	0,12	1,09	**108,87**	P61088	**Ubiquitin-conjugating enzyme E2 N**	UBE2N
**8 h Down**						
0,026	-0,16	0,90	**89,59**	P48739	**Phosphatidylinositol transfer protein beta isoform**	PITPNB
0,030	-0,17	0,89	**88,91**	P35221	**Catenin alpha-1**	CTNNA1
0,033	-0,18	0,88	**88,27**	P46060	**Ran GTPase-activating protein 1**	RANGAP1
0,018	-0,18	0,88	**88,17**	P11142	**Heat shock cognate 71 kDa protein**	HSPA8
0,010	-0,19	0,88	**87,66**	P11021	**78 kDa glucose-regulated protein**	HSPA5
0,012	-0,20	0,87	**87,21**	P08238	**Heat shock protein HSP 90-beta**	HSP90AB1
0,028	-0,20	0,87	**87,06**	O43143	**Putative pre-mRNA-splicing factor ATP-dependent RNA helicase DHX15**	DHX15
0,024	-0,20	0,87	**86,85**	P49411	**Elongation factor Tu, mitochondrial**	TUFM
0,023	-0,21	0,86	**86,46**	P46940	**Ras GTPase-activating-like protein IQGAP1**	IQGAP1
0,039	-0,21	0,86	**86,23**	P15311	**Ezrin**	EZR
0,001	-0,22	0,86	**85,86**	P11234	**Ras-related protein Ral-B**	RALB
0,024	-0,22	0,86	**85,70**	P08574	**Cytochrome c1, heme protein, mitochondrial**	CYC1
0,021	-0,24	0,85	**84,67**	Q9NRG0	**Chromatin accessibility complex protein 1**	CHRAC1
0,018	-0,27	0,83	**83,18**	P61353	**60S ribosomal protein L27**	RPL27
0,013	-0,27	0,83	**83,02**	P35580	**Myosin-10**	MYH10
0,049	-0,27	0,83	**82,90**	P78371	**T-complex protein 1 subunit beta**	CCT2
0,045	-0,27	0,83	**82,82**	P28482	**Mitogen-activated protein kinase 1**	MAPK1

0,033	-0,28	0,82	**82,42**	P63220	**40S ribosomal protein S21**	RPS21
0,048	-0,29	0,82	**82,01**	P18669	**Phosphoglycerate mutase 1**	PGAM1
0,045	-0,29	0,82	**81,79**	Q16630	**Cleavage and polyadenylation specificity factor subunit 6**	CPSF6
0,031	-0,29	0,82	**81,79**	P40429;Q6NVV1	**60S ribosomal protein L13a;Putative 60S ribosomal protein L13a protein RPL13AP3**	RPL13A;RPL13AP3
0,033	-0,29	0,82	**81,79**	P48444	**Coatomer subunit delta**	ARCN1
0,014	-0,29	0,82	**81,77**	Q8IXI1	**Mitochondrial Rho GTPase 2**	RHOT2
0,019	-0,29	0,82	**81,60**	Q15019	**Septin-2**	37500,000
0,007	-0,29	0,82	**81,54**	P35222	**Catenin beta-1**	CTNNB1
0,016	-0,30	0,81	**81,37**	O00231	**26S proteasome non-ATPase regulatory subunit 11**	PSMD11
0,026	-0,30	0,81	**81,23**	P42677	**40S ribosomal protein S27**	RPS27
0,033	-0,30	0,81	**81,18**	O96019	**Actin-like protein 6A**	ACTL6A
0,013	-0,31	0,81	**80,66**	O43809	**Cleavage and polyadenylation specificity factor subunit 5**	NUDT21
0,043	-0,31	0,81	**80,66**	Q9H307	**Pinin**	PNN
0,050	-0,31	0,81	**80,66**	P54578	**Ubiquitin carboxyl-terminal hydrolase 14**	USP14
0,013	-0,31	0,81	**80,55**	P25786	**Proteasome subunit alpha type-1**	PSMA1
0,041	-0,32	0,80	**80,35**	P62993	**Growth factor receptor-bound protein 2**	GRB2
0,022	-0,33	0,79	**79,37**	P62333	**26S protease regulatory subunit 10B**	PSMC6
0,011	-0,33	0,79	**79,34**	O76031	**ATP-dependent Clp protease ATP-binding subunit clpX-like, mitochondrial**	CLPX
0,038	-0,34	0,79	**79,00**	Q86XP3	**ATP-dependent RNA helicase DDX42**	DDX42
0,000	-0,35	0,78	**78,46**	P49327	**Fatty acid synthase;[Acyl-carrier-protein] S-acetyltransferase;[Acyl-carrier-protein] S**	FASN
0,025	-0,37	0,77	**77,38**	Q52LJ0	**Protein FAM98B**	FAM98B
0,041	-0,37	0,77	**77,25**	Q9Y371	**Endophilin-B1**	SH3GLB1
0,028	-0,38	0,77	**76,91**	P82094	**TATA element modulatory factor**	TMF1
0,041	-0,38	0,77	**76,84**	O00303	**Eukaryotic translation initiation factor 3 subunit F**	EIF3F
0,008	-0,39	0,76	**76,31**	Q96SI9	**Spermatid perinuclear RNA-binding protein**	STRBP
0,033	-0,39	0,76	**76,31**	O94906	**Pre-mRNA-processing factor 6**	PRPF6
0,019	-0,39	0,76	**76,31**	Q9Y3C1	**Nucleolar protein 16**	NOP16
0,006	-0,39	0,76	**76,22**	P16422	**Epithelial cell adhesion molecule**	EPCAM
0,002	-0,40	0,76	**75,79**	P30154	**Serine/threonine-protein phosphatase 2A 65 kDa regulatory subunit A beta isoform**	PPP2R1B
0,042	-0,41	0,75	**75,26**	Q7L576	**Cytoplasmic FMR1-interacting protein 1**	CYFIP1
0,050	-0,42	0,75	**74,74**	Q9NP79	**Vacuolar protein sorting-associated protein VTA1 homolog**	VTA1
0,000	-0,43	0,74	**74,24**	P57764	**Gasdermin-D**	GSDMD
0,012	-0,43	0,74	**74,23**	A6NHQ2	**rRNA/tRNA 2-O-methyltransferase fibrillarin-like protein 1**	FBLL1

0,020	-0,44	0,74	**73,96**	P11802	**Cyclin-dependent kinase 4**	CDK4
0,022	-0,44	0,74	**73,84**	Q8N7H5	**RNA polymerase II-associated factor 1 homolog**	PAF1
0,050	-0,44	0,74	**73,76**	P53007	**Tricarboxylate transport protein, mitochondrial**	SLC25A1
0,002	-0,44	0,74	**73,67**	Q13867	**Bleomycin hydrolase**	BLMH
0,047	-0,45	0,73	**73,20**	Q8NC51	**Plasminogen activator inhibitor 1 RNA-binding protein**	SERBP1
0,045	-0,46	0,73	**72,80**	Q01844	**RNA-binding protein EWS**	EWSR1
0,014	-0,47	0,72	**72,20**	O15550;O14607	**Lysine-specific demethylase 6A;Histone demethylase UTY**	KDM6A;UTY
0,000	-0,47	0,72	**72,12**	Q14165	**Malectin**	MLEC
0,028	-0,48	0,72	**71,92**	Q9BY43	**Charged multivesicular body protein 4a**	CHMP4A
0,004	-0,49	0,71	**71,05**	O95861	**3(2),5-bisphosphate nucleotidase 1**	BPNT1
0,005	-0,50	0,71	**70,86**	Q13098	**COP9 signalosome complex subunit 1**	GPS1
0,013	-0,50	0,71	**70,71**	P26358	**DNA (cytosine-5)-methyltransferase 1**	DNMT1
0,028	-0,50	0,71	**70,71**	P49792	**E3 SUMO-protein ligase RanBP2**	RANBP2
0,036	-0,50	0,71	**70,71**	P63165	**Small ubiquitin-related modifier 1**	SUMO1
0,045	-0,52	0,70	**69,74**	O95433	**Activator of 90 kDa heat shock protein ATPase homolog 1**	AHSA1
0,007	-0,53	0,69	**69,36**	Q12888	**Tumor suppressor p53-binding protein 1**	TP53BP1
0,019	-0,53	0,69	**69,26**	O75822	**Eukaryotic translation initiation factor 3 subunit J**	EIF3J
0,019	-0,53	0,69	**69,26**	P43246	**DNA mismatch repair protein Msh2**	MSH2
0,041	-0,54	0,69	**68,72**	Q86Y56	**HEAT repeat-containing protein 2**	HEATR2
0,015	-0,55	0,68	**68,19**	O43847	**Nardilysin**	NRD1
0,030	-0,56	0,68	**67,83**	Q9P2T1	**GMP reductase 2**	GMPR2
0,029	-0,56	0,68	**67,83**	Q9Y388	**RNA-binding motif protein, X-linked 2**	RBMX2
0,023	-0,57	0,67	**67,36**	Q9H981	**Actin-related protein 8**	ACTR8
0,041	-0,57	0,67	**67,36**	Q9H6T3	**RNA polymerase II-associated protein 3**	RPAP3
0,004	-0,57	0,67	**67,32**	P63241;Q6IS14	**Eukaryotic translation initiation factor 5A-1;Eukaryotic translation initiation factor 5**	EIF5A;EIF5AL1
0,043	-0,57	0,67	**67,25**	P31153	**S-adenosylmethionine synthase isoform type-2**	MAT2A
0,039	-0,57	0,67	**67,19**	Q96BP3	**Peptidylprolyl isomerase domain and WD repeat-containing protein 1**	PPWD1
0,026	-0,57	0,67	**67,19**	O94992	**Protein HEXIM1**	HEXIM1
0,036	-0,58	0,67	**66,90**	O14653	**Golgi SNAP receptor complex member 2**	GOSR2
0,022	-0,59	0,67	**66,63**	Q6P1N9	**Putative deoxyribonuclease TATDN1**	TATDN1
0,047	-0,59	0,67	**66,61**	P13591	**Neural cell adhesion molecule 1**	NCAM1
0,028	-0,59	0,67	**66,53**	P19404	**NADH dehydrogenase [ubiquinone] flavoprotein 2, mitochondrial**	NDUFV2
0,045	-0,59	0,66	**66,43**	Q92830	**Histone acetyltransferase KAT2A**	KAT2A

0,009	-0,59	0,66	**66,31**	P24386	**Rab proteins geranylgeranyltransferase component A 1**	CHM
0,025	-0,61	0,66	**65,70**	Q9UFN0	**Protein NipSnap homolog 3A**	NIPSNAP3A
0,041	-0,61	0,66	**65,52**	Q9UNQ2	**Probable dimethyladenosine transferase**	DIMT1
0,039	-0,62	0,65	**65,26**	P09496	**Clathrin light chain A**	CLTA
0,012	-0,62	0,65	**65,07**	Q7Z589	**Protein EMSY**	EMSY
0,042	-0,63	0,65	**64,62**	Q14764	**Major vault protein**	MVP
0,000	-0,63	0,65	**64,53**	P07686	**Beta-hexosaminidase subunit beta;Beta-hexosaminidase subunit beta chain B;Beta-**	HEXB
0,027	-0,63	0,64	**64,42**	Q13501	**Sequestosome-1**	SQSTM1
0,028	-0,64	0,64	**64,17**	O75347	**Tubulin-specific chaperone A**	TBCA
0,013	-0,65	0,64	**63,85**	Q9Y5K8	**V-type proton ATPase subunit D**	ATP6V1D
0,018	-0,65	0,64	**63,73**	O60307	**Microtubule-associated serine/threonine-protein kinase 3**	MAST3
0,003	-0,67	0,63	**62,85**	Q9HCG8	**Pre-mRNA-splicing factor CWC22 homolog**	CWC22
0,043	-0,67	0,63	**62,85**	Q99567	**Nuclear pore complex protein Nup88**	NUP88
0,042	-0,67	0,63	**62,85**	Q13442	**28 kDa heat- and acid-stable phosphoprotein**	PDAP1
0,002	-0,67	0,63	**62,74**	Q96P16	**Regulation of nuclear pre-mRNA domain-containing protein 1A**	RPRD1A
0,028	-0,68	0,63	**62,58**	Q9H7P9	**Pleckstrin homology domain-containing family G member 2**	PLEKHG2
0,007	-0,68	0,62	**62,42**	Q49AG3	**Zinc finger BED domain-containing protein 5**	ZBED5
0,022	-0,68	0,62	**62,42**	O94880	**PHD finger protein 14**	PHF14
0,005	-0,68	0,62	**62,42**	O95490	**Latrophilin-2**	LPHN2
0,000	-0,68	0,62	**62,39**	P49585;Q9Y5K3	**Choline-phosphate cytidylyltransferase A;Choline-phosphate cytidylyltransferase B**	PCYT1A;PCYT1B
0,011	-0,69	0,62	**61,99**	P46013	**Antigen KI-67**	MKI67
0,006	-0,69	0,62	**61,99**	P12004	**Proliferating cell nuclear antigen**	PCNA
0,030	-0,69	0,62	**61,82**	O14893	**Gem-associated protein 2**	GEMIN2
0,012	-0,70	0,62	**61,64**	Q9BSV6	**tRNA-splicing endonuclease subunit Sen34**	TSEN34
0,031	-0,70	0,62	**61,56**	Q08J23	**tRNA (cytosine(34)-C(5))-methyltransferase**	NSUN2
0,007	-0,71	0,61	**61,13**	Q9Y5J1	**U3 small nucleolar RNA-associated protein 18 homolog**	UTP18
0,002	-0,71	0,61	**61,13**	P50502;Q8NFI4;Q8IZP2	**Hsc70-interacting protein;Putative protein FAM10A5;Putative protein FAM10A4**	T13;ST13P5;ST13P
0,011	-0,71	0,61	**61,05**	P08047	**Transcription factor Sp1**	SP1
0,009	-0,72	0,61	**60,90**	Q9NQX6	**Zinc finger protein 331**	ZNF331
0,017	-0,73	0,60	**60,36**	O96008	**Mitochondrial import receptor subunit TOM40 homolog**	TOMM40
0,050	-0,73	0,60	**60,35**	Q01995	**Transgelin**	TAGLN
0,003	-0,73	0,60	**60,33**	Q96Q11	**CCA tRNA nucleotidyltransferase 1, mitochondrial**	TRNT1
0,049	-0,73	0,60	**60,29**	P53992	**Protein transport protein Sec24C**	SEC24C

0,033	-0,73	0,60	**60,29**	P08048;P17010	**Zinc finger Y-chromosomal protein;Zinc finger X-chromosomal protein**	ZFY;ZFX
0,026	-0,73	0,60	**60,16**	P10155	**60 kDa SS-A/Ro ribonucleoprotein**	TROVE2
0,042	-0,74	0,60	**60,04**	Q96HW7	**Integrator complex subunit 4**	INTS4
0,030	-0,74	0,60	**59,87**	Q9H078	**Caseinolytic peptidase B protein homolog**	CLPB
0,023	-0,74	0,60	**59,87**	Q01804	**OTU domain-containing protein 4**	OTUD4
0,044	-0,74	0,60	**59,87**	Q92614	**Unconventional myosin-XVIIIa**	MYO18A
0,010	-0,74	0,60	**59,81**	O00217	**NADH dehydrogenase [ubiquinone] iron-sulfur protein 8, mitochondrial**	NDUFS8
0,015	-0,74	0,60	**59,72**	Q16539	**Mitogen-activated protein kinase 14**	MAPK14
0,018	-0,75	0,59	**59,44**	P23458	**Tyrosine-protein kinase JAK1**	JAK1
0,041	-0,76	0,59	**59,05**	Q6ISB3	**Grainyhead-like protein 2 homolog**	GRHL2
0,050	-0,76	0,59	**59,05**	Q96EZ8	**Microspherule protein 1**	MCRS1
0,003	-0,78	0,58	**58,40**	Q13617	**Cullin-2**	CUL2
0,028	-0,78	0,58	**58,27**	Q9UG63	**ATP-binding cassette sub-family F member 2**	ABCF2
0,027	-0,78	0,58	**58,24**	Q9BZJ0	**Crooked neck-like protein 1**	CRNKL1
0,011	-0,79	0,58	**57,86**	Q9Y2S0	**DNA-directed RNA polymerases I and III subunit RPAC2**	POLR1D
0,009	-0,79	0,58	**57,83**	Q14192	**Four and a half LIM domains protein 2**	FHL2
0,020	-0,79	0,58	**57,83**	Q9UHV7	**Mediator of RNA polymerase II transcription subunit 13**	MED13
0,021	-0,79	0,58	**57,78**	Q15382	**GTP-binding protein Rheb**	RHEB
0,027	-0,80	0,57	**57,35**	Q13144	**Translation initiation factor eIF-2B subunit epsilon**	EIF2B5
0,025	-0,81	0,57	**57,20**	Q96PE2	**Rho guanine nucleotide exchange factor 17**	ARHGEF17
0,036	-0,81	0,57	**57,16**	P43307	**Translocon-associated protein subunit alpha**	SSR1
0,037	-0,81	0,57	**57,04**	Q9Y2A7	**Nck-associated protein 1**	NCKAP1
0,031	-0,81	0,57	**57,04**	Q16775	**Hydroxyacylglutathione hydrolase, mitochondrial**	HAGH
0,007	-0,82	0,57	**56,64**	P41227	**N-alpha-acetyltransferase 10**	NAA10
0,028	-0,82	0,57	**56,61**	O95166;Q9H0R8	**Gamma-aminobutyric acid receptor-associated protein;Gamma-aminobutyric acid r**	ABARAP;GABARAPL
0,009	-0,82	0,57	**56,56**	Q53GQ0	**Estradiol 17-beta-dehydrogenase 12**	HSD17B12
0,040	-0,83	0,56	**56,41**	Q92817	**Envoplakin**	EVPL
0,036	-0,83	0,56	**56,25**	A1L020	**RNA-binding protein MEX3A**	MEX3A
0,024	-0,83	0,56	**56,25**	P53621	**Coatomer subunit alpha;Xenin;Proxenin**	COPA
0,001	-0,84	0,56	**55,86**	P51858	**Hepatoma-derived growth factor**	HDGF
0,017	-0,85	0,55	**55,50**	Q9UBQ7	**Glyoxylate reductase/hydroxypyruvate reductase**	GRHPR
0,007	-0,85	0,55	**55,48**	P52948	**Nuclear pore complex protein Nup98-Nup96;Nuclear pore complex protein Nup98;N**	NUP98
0,037	-0,85	0,55	**55,46**	Q6P1L8	**39S ribosomal protein L14, mitochondrial**	MRPL14

0,034	-0,85	0,55	**55,32**	Q14241	**Transcription elongation factor B polypeptide 3**	TCEB3
0,008	-0,86	0,55	**55,19**	Q14134	**Tripartite motif-containing protein 29**	TRIM29
0,042	-0,86	0,55	**55,10**	P21283	**V-type proton ATPase subunit C 1**	ATP6V1C1
0,018	-0,86	0,55	**55,09**	Q8NEL9	**Phospholipase DDHD1**	DDHD1
0,022	-0,88	0,54	**54,34**	Q96CW1	**AP-2 complex subunit mu**	AP2M1
0,001	-0,88	0,54	**54,34**	Q8IYB3	**Serine/arginine repetitive matrix protein 1**	SRRM1
0,035	-0,88	0,54	**54,34**	Q8NEM2	**SHC SH2 domain-binding protein 1**	SHCBP1
0,022	-0,89	0,54	**53,96**	Q6UXN9	**WD repeat-containing protein 82**	WDR82
0,025	-0,89	0,54	**53,96**	Q9H0W8	**Protein SMG9**	SMG9
0,019	-0,89	0,54	**53,96**	P12236	**ADP/ATP translocase 3;ADP/ATP translocase 3, N-terminally processed**	SLC25A6
0,039	-0,89	0,54	**53,96**	P55010	**Eukaryotic translation initiation factor 5**	EIF5
0,038	-0,89	0,54	**53,96**	Q8IV08	**Phospholipase D3**	PLD3
0,032	-0,89	0,54	**53,79**	Q9P015	**39S ribosomal protein L15, mitochondrial**	MRPL15
0,013	-0,90	0,54	**53,71**	Q9Y4B6	**Protein VPRBP**	VPRBP
0,037	-0,90	0,54	**53,57**	Q9Y2W1	**Thyroid hormone receptor-associated protein 3**	THRAP3
0,050	-0,91	0,53	**53,22**	P50416	**Carnitine O-palmitoyltransferase 1, liver isoform**	CPT1A
0,009	-0,91	0,53	**53,04**	Q969T7	**7-methylguanosine phosphate-specific 5-nucleotidase**	NT5C3B
0,018	-0,92	0,53	**53,03**	Q8TDH9	**Biogenesis of lysosome-related organelles complex 1 subunit 5**	BLOC1S5
0,001	-0,92	0,53	**52,99**	Q5VTR2	**E3 ubiquitin-protein ligase BRE1A**	RNF20
0,037	-0,92	0,53	**52,80**	Q9H993	**UPF0364 protein C6orf211**	C6orf211
0,010	-0,94	0,52	**52,29**	Q06210	**Glutamine--fructose-6-phosphate aminotransferase [isomerizing] 1**	GFPT1
0,010	-0,94	0,52	**52,12**	Q9H6W3	**Bifunctional lysine-specific demethylase and histidyl-hydroxylase NO66**	NO66
0,029	-0,94	0,52	**52,12**	P69905	**Hemoglobin subunit alpha**	HBA1
0,021	-0,94	0,52	**51,99**	P16219	**Short-chain specific acyl-CoA dehydrogenase, mitochondrial**	ACADS
0,034	-0,95	0,52	**51,76**	Q96KQ7	**Histone-lysine N-methyltransferase EHMT2**	EHMT2
0,023	-0,96	0,51	**51,48**	Q9H0E9	**Bromodomain-containing protein 8**	BRD8
0,042	-0,96	0,51	**51,41**	Q7Z7C8	**Transcription initiation factor TFIID subunit 8**	TAF8
0,006	-0,96	0,51	**51,41**	Q92576	**PHD finger protein 3**	PHF3
0,033	-0,96	0,51	**51,41**	Q9H4L4	**Sentrin-specific protease 3**	SENP3
0,035	-0,97	0,51	**51,05**	Q9NPI6	**mRNA-decapping enzyme 1A**	DCP1A
0,035	-0,97	0,51	**51,05**	P38606	**V-type proton ATPase catalytic subunit A**	ATP6V1A
0,029	-0,98	0,51	**50,70**	P19224	**UDP-glucuronosyltransferase 1-6**	UGT1A6
0,045	-0,99	0,50	**50,35**	O14967	**Calmegin**	CLGN

0,009	-0,99	0,50	**50,35**	Q92563	**Testican-2**	SPOCK2
0,011	-0,99	0,50	**50,35**	P06756	**Integrin alpha-V;Integrin alpha-V heavy chain;Integrin alpha-V light chain**	ITGAV
0,027	-0,99	0,50	**50,28**	Q92667	**A-kinase anchor protein 1, mitochondrial**	AKAP1
0,023	-1,00	0,50	**50,08**	Q8NDH3	**Probable aminopeptidase NPEPL1**	NPEPL1
0,016	-1,01	0,50	**49,67**	Q9NVS9	**Pyridoxine-5-phosphate oxidase**	PNPO
0,010	-1,01	0,50	**49,65**	Q9UBW7	**Zinc finger MYM-type protein 2**	ZMYM2
0,042	-1,01	0,50	**49,65**	Q8WXE1	**ATR-interacting protein**	ATRIP
0,026	-1,01	0,50	**49,65**	Q9NTZ6	**RNA-binding protein 12**	RBM12
0,031	-1,02	0,49	**49,31**	Q14669	**E3 ubiquitin-protein ligase TRIP12**	TRIP12
0,002	-1,02	0,49	**49,31**	Q9Y580	**RNA-binding protein 7**	RBM7
0,048	-1,03	0,49	**48,97**	Q8IUC4	**Rhophilin-2**	RHPN2
0,046	-1,03	0,49	**48,97**	Q15811	**Intersectin-1**	ITSN1
0,026	-1,04	0,49	**48,67**	Q96GA3	**Protein LTV1 homolog**	LTV1
0,025	-1,04	0,49	**48,63**	Q6P1J9	**Parafibromin**	CDC73
0,041	-1,04	0,49	**48,63**	Q15004	**PCNA-associated factor**	KIAA0101
0,044	-1,04	0,49	**48,63**	P35241	**Radixin**	RDX
0,029	-1,05	0,48	**48,38**	Q92544	**Transmembrane 9 superfamily member 4**	TM9SF4
0,030	-1,05	0,48	**48,33**	O95394	**Phosphoacetylglucosamine mutase**	PGM3
0,035	-1,05	0,48	**48,30**	Q9H8V3	**Protein ECT2**	ECT2
0,008	-1,05	0,48	**48,30**	Q8WUM0	**Nuclear pore complex protein Nup133**	NUP133
0,043	-1,06	0,48	**47,96**	P11086	**Phenylethanolamine N-methyltransferase**	PNMT
0,000	-1,07	0,48	**47,80**	Q9NTJ3	**Structural maintenance of chromosomes protein 4**	SMC4
0,039	-1,07	0,48	**47,63**	O95453	**Poly(A)-specific ribonuclease PARN**	PARN
0,021	-1,09	0,47	**46,98**	O75367	**Core histone macro-H2A.1**	H2AFY
0,022	-1,09	0,47	**46,98**	Q9NZN4	**EH domain-containing protein 2**	EHD2
0,000	-1,09	0,47	**46,89**	Q96C86	**m7GpppX diphosphatase**	DCPS
0,049	-1,10	0,47	**46,66**	Q9H1E3	**Nuclear ubiquitous casein and cyclin-dependent kinase substrate 1**	NUCKS1
0,034	-1,10	0,47	**46,66**	O43768	**Alpha-endosulfine**	ENSA
0,045	-1,10	0,47	**46,55**	Q69YN4	**Protein virilizer homolog**	KIAA1429
0,042	-1,11	0,46	**46,47**	P27448	**MAP/microtubule affinity-regulating kinase 3**	MARK3
0,034	-1,12	0,46	**46,10**	Q8WVX9	**Fatty acyl-CoA reductase 1**	FAR1
0,021	-1,12	0,46	**46,08**	Q8N1G2	**Cap-specific mRNA (nucleoside-2-O-)-methyltransferase 1**	CMTR1
0,014	-1,12	0,46	**46,01**	O60318	**Germinal-center associated nuclear protein**	MCM3AP

0,012	-1,13	0,46	**45,55**	Q9UKK6	**NTF2-related export protein 1**	NXT1
0,007	-1,14	0,45	**45,38**	Q13162	**Peroxiredoxin-4**	PRDX4
0,005	-1,14	0,45	**45,38**	9BWW4;P81877;Q9BW(	**Single-stranded DNA-binding protein 3;Single-stranded DNA-binding protein 2;Singl**	SSBP3;SSBP2;SSBP4
0,006	-1,16	0,45	**44,75**	P30504	**HLA class I histocompatibility antigen, Cw-4 alpha chain**	HLA-C
0,007	-1,16	0,45	**44,75**	Q5VZL5	**Zinc finger MYM-type protein 4**	ZMYM4
0,046	-1,16	0,45	**44,60**	P46736	**Lys-63-specific deubiquitinase BRCC36**	BRCC3
0,013	-1,17	0,44	**44,32**	Q9Y6H1;Q5T1J5	**Coiled-coil-helix-coiled-coil-helix domain-containing protein 2, mitochondrial;Putati**	CHCHD2;CHCHD2P9
0,017	-1,18	0,44	**44,26**	O15042	**U2 snRNP-associated SURP motif-containing protein**	U2SURP
0,031	-1,18	0,44	**44,14**	Q9HCM1	**Uncharacterized protein KIAA1551**	KIAA1551
0,002	-1,19	0,44	**43,83**	Q53H12	**Acylglycerol kinase, mitochondrial**	AGK
0,049	-1,19	0,44	**43,83**	Q8N5C6	**S1 RNA-binding domain-containing protein 1**	SRBD1
0,012	-1,19	0,44	**43,73**	Q9NTX5	**Ethylmalonyl-CoA decarboxylase**	ECHDC1
0,004	-1,20	0,44	**43,66**	Q7L3B6	**Hsp90 co-chaperone Cdc37-like 1**	CDC37L1
0,022	-1,20	0,44	**43,60**	P31350	**Ribonucleoside-diphosphate reductase subunit M2**	RRM2
0,006	-1,20	0,44	**43,53**	Q13503	**Mediator of RNA polymerase II transcription subunit 21**	MED21
0,043	-1,21	0,43	**43,23**	Q9BRD0	**BUD13 homolog**	BUD13
0,050	-1,22	0,43	**42,98**	Q6PK04	**Coiled-coil domain-containing protein 137**	CCDC137
0,036	-1,22	0,43	**42,93**	Q8TF74	**WAS/WASL-interacting protein family member 2**	WIPF2
0,001	-1,22	0,43	**42,93**	Q15545	**Transcription initiation factor TFIID subunit 7**	TAF7
0,049	-1,22	0,43	**42,90**	Q9Y5L0	**Transportin-3**	TNPO3
0,038	-1,22	0,43	**42,90**	P55210	**Caspase-7;Caspase-7 subunit p20;Caspase-7 subunit p11**	CASP7
0,007	-1,23	0,43	**42,63**	O95071	**E3 ubiquitin-protein ligase UBR5**	UBR5
0,006	-1,25	0,42	**42,04**	P29375	**Lysine-specific demethylase 5A**	KDM5A
0,036	-1,25	0,42	**42,04**	O15460	**Prolyl 4-hydroxylase subunit alpha-2**	P4HA2
0,049	-1,26	0,42	**41,75**	Q9BWD1	**Acetyl-CoA acetyltransferase, cytosolic**	ACAT2
0,050	-1,27	0,41	**41,47**	Q9BZQ8	**Protein Niban**	FAM129A
0,050	-1,27	0,41	**41,47**	P23921	**Ribonucleoside-diphosphate reductase large subunit**	RRM1
0,002	-1,27	0,41	**41,47**	Q9BQ39	**ATP-dependent RNA helicase DDX50**	DDX50
0,009	-1,28	0,41	**41,21**	O94855	**Protein transport protein Sec24D**	SEC24D
0,048	-1,28	0,41	**41,18**	P51572	**B-cell receptor-associated protein 31**	BCAP31
0,038	-1,29	0,41	**40,90**	P84090	**Enhancer of rudimentary homolog**	ERH
0,029	-1,29	0,41	**40,77**	Q9H267	**Vacuolar protein sorting-associated protein 33B**	VPS33B
0,007	-1,30	0,41	**40,73**	O15347	**High mobility group protein B3**	HMGB3

0,034	-1,30	0,41	**40,61**	Q92796	**Disks large homolog 3**	DLG3
0,009	-1,30	0,41	**40,61**	P50402	**Emerin**	EMD
0,014	-1,30	0,41	**40,61**	Q8TEX9	**Importin-4**	IPO4
0,046	-1,30	0,41	**40,61**	Q9Y2R5	**28S ribosomal protein S17, mitochondrial**	MRPS17
0,047	-1,31	0,40	**40,33**	Q12797	**Aspartyl/asparaginyl beta-hydroxylase**	ASPH
0,043	-1,33	0,40	**39,78**	Q9NXX6	**Non-structural maintenance of chromosomes element 4 homolog A**	NSMCE4A
0,049	-1,33	0,40	**39,78**	Q9BWM7	**Sideroflexin-3**	SFXN3
0,007	-1,34	0,40	**39,50**	Q9Y4F1	**FERM, RhoGEF and pleckstrin domain-containing protein 1**	FARP1
0,032	-1,34	0,40	**39,50**	Q5VWG9	**Transcription initiation factor TFIID subunit 3**	TAF3
0,048	-1,35	0,39	**39,24**	Q96E29	**Transcription termination factor 3, mitochondrial**	MTERF3
0,037	-1,35	0,39	**39,12**	Q9BZ23	**Pantothenate kinase 2, mitochondrial**	PANK2
0,028	-1,36	0,39	**38,96**	Q8IWA0	**WD repeat-containing protein 75**	WDR75
0,039	-1,36	0,39	**38,96**	Q9HCS7	**Pre-mRNA-splicing factor SYF1**	XAB2
0,004	-1,36	0,39	**38,96**	P40692	**DNA mismatch repair protein Mlh1**	MLH1
0,003	-1,37	0,39	**38,75**	Q9NZ45	**CDGSH iron-sulfur domain-containing protein 1**	CISD1
0,000	-1,37	0,39	**38,75**	Q9H488	**GDP-fucose protein O-fucosyltransferase 1**	POFUT1
0,009	-1,37	0,39	**38,72**	Q9NX08	**COMM domain-containing protein 8**	COMMD8
0,024	-1,37	0,39	**38,69**	O14980	**Exportin-1**	XPO1
0,015	-1,38	0,38	**38,42**	Q69YH5	**Cell division cycle-associated protein 2**	CDCA2
0,049	-1,38	0,38	**38,42**	439;P13746;P30455;P30	**HLA class I histocompatibility antigen, A-3 alpha chain;HLA class I histocompatibility**	HLA-A
0,034	-1,38	0,38	**38,41**	Q9Y314	**Nitric oxide synthase-interacting protein**	NOSIP
0,004	-1,39	0,38	**38,24**	Q9HD26	**Golgi-associated PDZ and coiled-coil motif-containing protein**	GOPC
0,001	-1,39	0,38	**38,16**	Q16706	**Alpha-mannosidase 2**	MAN2A1
0,021	-1,39	0,38	**38,16**	P52209	**6-phosphogluconate dehydrogenase, decarboxylating**	PGD
0,030	-1,40	0,38	**38,02**	P12830	**Cadherin-1;E-Cad/CTF1;E-Cad/CTF2;E-Cad/CTF3**	CDH1
0,010	-1,40	0,38	**37,89**	Q12849	**G-rich sequence factor 1**	GRSF1
0,004	-1,40	0,38	**37,82**	Q8IY67	**Ribonucleoprotein PTB-binding 1**	RAVER1
0,017	-1,41	0,38	**37,63**	Q96T58	**Msx2-interacting protein**	SPEN
0,045	-1,41	0,38	**37,63**	O75436	**Vacuolar protein sorting-associated protein 26A**	VPS26A
0,038	-1,42	0,37	**37,49**	Q6UN15	**Pre-mRNA 3-end-processing factor FIP1**	FIP1L1
0,001	-1,43	0,37	**37,11**	P54886	**Delta-1-pyrroline-5-carboxylate synthase;Glutamate 5-kinase;Gamma-glutamyl pho**	ALDH18A1
0,016	-1,44	0,37	**36,86**	Q92616	**Translational activator GCN1**	GCN1L1
0,026	-1,45	0,37	**36,60**	O60306	**Intron-binding protein aquarius**	AQR

0,047	-1,45	0,37	**36,58**	O15047	**Histone-lysine N-methyltransferase SETD1A**	SETD1A
0,025	-1,46	0,36	**36,35**	Q9BQ69	**O-acetyl-ADP-ribose deacetylase MACROD1**	MACROD1
0,049	-1,46	0,36	**36,35**	O00170	**AH receptor-interacting protein**	AIP
0,047	-1,46	0,36	**36,35**	Q96MX6	**WD repeat-containing protein 92**	WDR92
0,006	-1,46	0,36	**36,34**	Q13505	**Metaxin-1**	MTX1
0,002	-1,48	0,36	**35,85**	Q68CP9	**AT-rich interactive domain-containing protein 2**	ARID2
0,030	-1,49	0,36	**35,61**	Q8TCZ2	**CD99 antigen-like protein 2**	CD99L2
0,028	-1,49	0,36	**35,60**	P62166	**Neuronal calcium sensor 1**	NCS1
0,040	-1,49	0,36	**35,60**	P09001	**39S ribosomal protein L3, mitochondrial**	MRPL3
0,035	-1,49	0,36	**35,60**	P19387	**DNA-directed RNA polymerase II subunit RPB3**	POLR2C
0,006	-1,50	0,35	**35,36**	Q5T200	**Zinc finger CCCH domain-containing protein 13**	ZC3H13
0,029	-1,51	0,35	**35,11**	P22234	**Multifunctional protein ADE2;Phosphoribosylaminoimidazole-succinocarboxamide s**	PAICS
0,023	-1,51	0,35	**35,11**	Q15528	**Mediator of RNA polymerase II transcription subunit 22**	MED22
0,009	-1,51	0,35	**35,11**	O15027	**Protein transport protein Sec16A**	SEC16A
0,049	-1,52	0,35	**34,87**	Q6VMQ6	**Activating transcription factor 7-interacting protein 1**	ATF7IP
0,010	-1,52	0,35	**34,87**	Q12769	**Nuclear pore complex protein Nup160**	NUP160
0,030	-1,52	0,35	**34,87**	P48634	**Protein PRRC2A**	PRRC2A
0,033	-1,53	0,35	**34,51**	Q9H9Y6	**DNA-directed RNA polymerase I subunit RPA2**	POLR1B
0,005	-1,54	0,34	**34,39**	Q9BXS6	**Nucleolar and spindle-associated protein 1**	NUSAP1
0,003	-1,55	0,34	**34,23**	O94762	**ATP-dependent DNA helicase Q5**	RECQL5
0,048	-1,55	0,34	**34,19**	Q9H6I2	**Transcription factor SOX-17**	SOX17
0,015	-1,55	0,34	**34,15**	Q8NEJ9	**Neuroguidin**	NGDN
0,039	-1,55	0,34	**34,15**	P38432	**Coilin**	COIL
0,003	-1,57	0,34	**33,68**	Q9NYV4	**Cyclin-dependent kinase 12**	CDK12
0,001	-1,58	0,33	**33,47**	P40261	**Nicotinamide N-methyltransferase**	NNMT
0,007	-1,60	0,33	**32,99**	Q9UM13	**Anaphase-promoting complex subunit 10**	ANAPC10
0,038	-1,61	0,33	**32,84**	Q9P1Y5	**Calmodulin-regulated spectrin-associated protein 3**	CAMSAP3
0,039	-1,62	0,33	**32,56**	Q14738	**Serine/threonine-protein phosphatase 2A 56 kDa regulatory subunit delta isoform**	PPP2R5D
0,025	-1,62	0,33	**32,53**	O75534	**Cold shock domain-containing protein E1**	CSDE1
0,013	-1,63	0,32	**32,31**	Q9HCN4	**GPN-loop GTPase 1**	GPN1
0,012	-1,64	0,32	**32,09**	Q8N2Z9	**Centromere protein S**	APITD1
0,046	-1,64	0,32	**32,09**	P36776	**Lon protease homolog, mitochondrial**	LONP1
0,027	-1,64	0,32	**32,09**	P49591	**Serine--tRNA ligase, cytoplasmic**	SARS

0,033	-1,65	0,32	**31,91**	P0CG12	**Chromosome transmission fidelity protein 8 homolog isoform 2**	CHTF8
0,048	-1,65	0,32	**31,86**	Q9P2R6	**Arginine-glutamic acid dipeptide repeats protein**	RERE
0,042	-1,66	0,32	**31,64**	Q2NL82	**Pre-rRNA-processing protein TSR1 homolog**	TSR1
0,025	-1,66	0,32	**31,64**	Q9UGP8	**Translocation protein SEC63 homolog**	SEC63
0,017	-1,66	0,32	**31,64**	P33897	**ATP-binding cassette sub-family D member 1**	ABCD1
0,041	-1,67	0,32	**31,53**	O43414	**ERI1 exoribonuclease 3**	ERI3
0,008	-1,67	0,31	**31,43**	Q9Y2T2	**AP-3 complex subunit mu-1**	AP3M1
0,022	-1,67	0,31	**31,43**	P16152	**Carbonyl reductase [NADPH] 1**	CBR1
0,031	-1,68	0,31	**31,21**	Q96Q05	**Trafficking protein particle complex subunit 9**	TRAPPC9
0,012	-1,68	0,31	**31,21**	Q13084	**39S ribosomal protein L28, mitochondrial**	MRPL28
0,022	-1,69	0,31	**30,99**	Q8N5N7	**39S ribosomal protein L50, mitochondrial**	MRPL50
0,047	-1,72	0,30	**30,35**	P37837	**Transaldolase**	TALDO1
0,008	-1,73	0,30	**30,22**	P31749	**RAC-alpha serine/threonine-protein kinase**	AKT1
0,033	-1,73	0,30	**30,15**	P14927	**Cytochrome b-c1 complex subunit 7**	UQCRB
0,027	-1,74	0,30	**29,94**	P35573	**Glycogen debranching enzyme;4-alpha-glucanotransferase;Amylo-alpha-1,6-glucosi**	AGL
0,014	-1,74	0,30	**29,87**	Q16864	**V-type proton ATPase subunit F**	ATP6V1F
0,040	-1,76	0,30	**29,52**	P48730	**Casein kinase I isoform delta**	CSNK1D
0,012	-1,76	0,30	**29,52**	P62491;Q15907	**Ras-related protein Rab-11A;Ras-related protein Rab-11B**	RAB11A;RAB11B
0,017	-1,77	0,29	**29,42**	Q9NZD8	**Maspardin**	SPG21
0,015	-1,77	0,29	**29,38**	Q9UJX6	**Anaphase-promoting complex subunit 2**	ANAPC2
0,032	-1,77	0,29	**29,37**	P62070	**Ras-related protein R-Ras2**	RRAS2
0,017	-1,79	0,29	**28,92**	P30626	**Sorcin**	SRI
0,004	-1,80	0,29	**28,72**	Q9H9A5	**CCR4-NOT transcription complex subunit 10**	CNOT10
0,018	-1,81	0,29	**28,52**	Q8WUB8	**PHD finger protein 10**	PHF10
0,004	-1,81	0,29	**28,52**	A8MW92	**PHD finger protein 20-like protein 1**	PHF20L1
0,006	-1,81	0,29	**28,52**	O60293	**Zinc finger C3H1 domain-containing protein**	ZFC3H1
0,018	-1,81	0,28	**28,45**	Q14331;Q9BZ01	**Protein FRG1;Protein FRG1B**	FRG1;FRG1B
0,001	-1,82	0,28	**28,32**	Q969Z3;Q5VT66	**Mitochondrial amidoxime reducing component 2;Mitochondrial amidoxime-reducin**	MARC2;MARC1
0,028	-1,83	0,28	**28,11**	Q13535	**Serine/threonine-protein kinase ATR**	ATR
0,016	-1,84	0,28	**27,86**	O75928	**E3 SUMO-protein ligase PIAS2**	PIAS2
0,021	-1,85	0,28	**27,74**	Q9Y399	**28S ribosomal protein S2, mitochondrial**	MRPS2
0,024	-1,89	0,27	**27,01**	Q5R3I4	**Tetratricopeptide repeat protein 38**	TTC38
0,046	-1,91	0,27	**26,61**	Q08722	**Leukocyte surface antigen CD47**	CD47

0,005	-1,91	0,27	**26,56**	Q9UGM6	**Tryptophan--tRNA ligase, mitochondrial**	WARS2
0,007	-1,94	0,26	**26,06**	O95155	**Ubiquitin conjugation factor E4 B**	UBE4B
0,028	-1,94	0,26	**26,06**	Q02978	**Mitochondrial 2-oxoglutarate/malate carrier protein**	SLC25A11
0,038	-1,94	0,26	**26,06**	Q8NI36	**WD repeat-containing protein 36**	WDR36
0,021	-1,94	0,26	**26,06**	Q86XZ4	**Spermatogenesis-associated serine-rich protein 2**	SPATS2
0,007	-1,95	0,26	**25,94**	O95989	**Diphosphoinositol polyphosphate phosphohydrolase 1**	NUDT3
0,006	-1,96	0,26	**25,70**	Q9NY93	**Probable ATP-dependent RNA helicase DDX56**	DDX56
0,041	-1,97	0,26	**25,53**	P55060	**Exportin-2**	CSE1L
0,039	-1,99	0,25	**25,17**	P31939	**Bifunctional purine biosynthesis protein PURH;Phosphoribosylaminoimidazolecarbo**	ATIC
0,016	-1,99	0,25	**25,16**	P38935	**DNA-binding protein SMUBP-2**	IGHMBP2
0,039	-2,04	0,24	**24,32**	O95235	**Kinesin-like protein KIF20A**	KIF20A
0,004	-2,06	0,24	**23,97**	P98088	**Mucin-5AC**	MUC5AC
0,000	-2,08	0,24	**23,71**	Q8NF37	**Lysophosphatidylcholine acyltransferase 1**	LPCAT1
0,005	-2,08	0,24	**23,65**	P55011	**Solute carrier family 12 member 2**	SLC12A2
0,031	-2,09	0,24	**23,57**	Q9NZJ9	**Diphosphoinositol polyphosphate phosphohydrolase 2**	NUDT4
0,039	-2,10	0,23	**23,30**	Q06481	**Amyloid-like protein 2**	APLP2
0,038	-2,12	0,23	**23,00**	O75616	**GTPase Era, mitochondrial**	ERAL1
0,030	-2,13	0,23	**22,88**	P42898	**Methylenetetrahydrofolate reductase**	MTHFR
0,001	-2,13	0,23	**22,80**	Q16352	**Alpha-internexin**	INA
0,043	-2,13	0,23	**22,78**	P01891	**HLA class I histocompatibility antigen, A-68 alpha chain**	HLA-A
0,027	-2,14	0,23	**22,69**	P16402	**Histone H1.3**	HIST1H1D
0,011	-2,15	0,23	**22,51**	Q6P179	**Endoplasmic reticulum aminopeptidase 2**	ERAP2
0,002	-2,17	0,22	**22,22**	Q9H5H4	**Zinc finger protein 768**	ZNF768
0,021	-2,19	0,22	**21,92**	P49406	**39S ribosomal protein L19, mitochondrial**	MRPL19
0,038	-2,20	0,22	**21,76**	Q9Y2X0	**Mediator of RNA polymerase II transcription subunit 16**	MED16
0,000	-2,20	0,22	**21,76**	Q9Y6W5	**Wiskott-Aldrich syndrome protein family member 2**	WASF2
0,028	-2,20	0,22	**21,76**	Q9NRY4	**Rho GTPase-activating protein 35**	ARHGAP35
0,016	-2,22	0,21	**21,48**	Q96B36	**Proline-rich AKT1 substrate 1**	AKT1S1
0,044	-2,22	0,21	**21,40**	Q6NXR4	**TELO2-interacting protein 2**	TTI2
0,015	-2,23	0,21	**21,34**	P11171	**Protein 4.1**	EPB41
0,016	-2,23	0,21	**21,32**	Q12824	**SWI/SNF-related matrix-associated actin-dependent regulator of chromatin subfami**	SMARCB1
0,021	-2,24	0,21	**21,17**	Q9NYJ1	**Cytochrome c oxidase assembly factor 4 homolog, mitochondrial**	COA4
0,017	-2,25	0,21	**21,02**	P13807	**Glycogen [starch] synthase, muscle**	GYS1

0,047	-2,28	0,21	**20,66**	Q8TB45	**DEP domain-containing mTOR-interacting protein**	DEPTOR
0,038	-2,28	0,21	**20,59**	Q9BYG5	**Partitioning defective 6 homolog beta**	PARD6B
0,016	-2,28	0,21	**20,59**	Q5SVZ6	**Zinc finger MYM-type protein 1**	ZMYM1
0,032	-2,28	0,21	**20,59**	Q92665	**28S ribosomal protein S31, mitochondrial**	MRPS31
0,002	-2,28	0,21	**20,59**	Q15054	**DNA polymerase delta subunit 3**	POLD3
0,029	-2,33	0,20	**19,89**	Q9UHG0	**Doublecortin domain-containing protein 2**	DCDC2
0,029	-2,34	0,20	**19,80**	Q9H2Y7	**Zinc finger protein 106**	ZNF106
0,034	-2,35	0,20	**19,61**	Q8ND04	**Protein SMG8**	SMG8
0,018	-2,35	0,20	**19,61**	Q13630	**GDP-L-fucose synthase**	TSTA3
0,023	-2,36	0,19	**19,48**	Q04760	**Lactoylglutathione lyase**	GLO1
0,038	-2,36	0,19	**19,47**	O75962	**Triple functional domain protein**	TRIO
0,001	-2,37	0,19	**19,34**	Q9Y2Q3	**Glutathione S-transferase kappa 1**	GSTK1
0,003	-2,41	0,19	**18,82**	Q9UIV1	**CCR4-NOT transcription complex subunit 7**	CNOT7
0,021	-2,44	0,18	**18,43**	Q96JM3	**Chromosome alignment-maintaining phosphoprotein 1**	CHAMP1
0,023	-2,44	0,18	**18,43**	P01034	**Cystatin-C**	CST3
0,015	-2,45	0,18	**18,30**	Q14527	**Helicase-like transcription factor**	HLTF
0,043	-2,47	0,18	**18,05**	P25208	**Nuclear transcription factor Y subunit beta**	NFYB
0,003	-2,47	0,18	**18,03**	Q96EA4	**Protein Spindly**	SPDL1
0,005	-2,49	0,18	**17,80**	P07339	**Cathepsin D;Cathepsin D light chain;Cathepsin D heavy chain**	CTSD
0,001	-2,50	0,18	**17,73**	Q9Y3A4	**Ribosomal RNA-processing protein 7 homolog A**	RRP7A
0,008	-2,50	0,18	**17,68**	P10746	**Uroporphyrinogen-III synthase**	UROS
0,048	-2,50	0,18	**17,68**	O95298;E9PQ53	**NADH dehydrogenase [ubiquinone] 1 subunit C2;NADH dehydrogenase [ubiquinone**	UFC2;NDUFC2-KCTD
0,011	-2,51	0,18	**17,56**	O94973	**AP-2 complex subunit alpha-2**	AP2A2
0,045	-2,51	0,18	**17,56**	Q05D32	**CTD small phosphatase-like protein 2**	CTDSPL2
0,005	-2,52	0,17	**17,43**	Q14257	**Reticulocalbin-2**	RCN2
0,027	-2,52	0,17	**17,39**	P16401	**Histone H1.5**	HIST1H1B
0,024	-2,53	0,17	**17,36**	O75362	**Zinc finger protein 217**	ZNF217
0,014	-2,53	0,17	**17,26**	Q8N5A5	**Zinc finger CCCH-type with G patch domain-containing protein**	ZGPAT
0,035	-2,55	0,17	**17,08**	P30042	**ES1 protein homolog, mitochondrial**	C21orf33
0,001	-2,56	0,17	**16,95**	P98179	**Putative RNA-binding protein 3**	RBM3
0,019	-2,57	0,17	**16,84**	Q96KP1	**Exocyst complex component 2**	EXOC2
0,002	-2,66	0,16	**15,82**	Q9NYF8	**Bcl-2-associated transcription factor 1**	BCLAF1
0,041	-2,73	0,15	**15,11**	Q08AD1	**Calmodulin-regulated spectrin-associated protein 2**	CAMSAP2

0,002	-2,73	0,15	**15,07**	Q4FZB7	**Histone-lysine N-methyltransferase SUV420H1**	SUV420H1
0,010	-2,75	0,15	**14,83**	P78346	**Ribonuclease P protein subunit p30**	RPP30
0,002	-2,77	0,15	**14,62**	O94916	**Nuclear factor of activated T-cells 5**	NFAT5
0,000	-2,84	0,14	**13,97**	O43716	**Glutamyl-tRNA(Gln) amidotransferase subunit C, mitochondrial**	GATC
0,032	-2,85	0,14	**13,87**	Q9Y4E5	**Zinc finger protein 451**	ZNF451
0,034	-2,96	0,13	**12,85**	Q13451	**Peptidyl-prolyl cis-trans isomerase FKBP5**	FKBP5
0,010	-2,96	0,13	**12,85**	Q96RQ3	**Methylcrotonoyl-CoA carboxylase subunit alpha, mitochondrial**	MCCC1
0,002	-2,98	0,13	**12,70**	Q15361	**Transcription termination factor 1**	TTF1
0,020	-3,02	0,12	**12,33**	Q06587	**E3 ubiquitin-protein ligase RING1**	RING1
0,000	-3,05	0,12	**12,04**	Q8TD47	**40S ribosomal protein S4, Y isoform 2**	RPS4Y2
0,036	-3,06	0,12	**11,99**	P29034	**Protein S100-A2**	S100A2
0,001	-3,07	0,12	**11,91**	Q96CV9	**Optineurin**	OPTN
0,002	-3,11	0,12	**11,62**	Q96MU7	**YTH domain-containing protein 1**	YTHDC1
0,003	-3,11	0,12	**11,56**	A1X283	**SH3 and PX domain-containing protein 2B**	SH3PXD2B
0,043	-3,22	0,11	**10,77**	Q9BVK2	**Probable dolichyl pyrophosphate Glc1Man9GlcNAc2 alpha-1,3-glucosyltransferase**	ALG8
0,009	-3,24	0,11	**10,58**	Q9NVV4	**Poly(A) RNA polymerase, mitochondrial**	MTPAP
0,026	-3,25	0,11	**10,51**	Q8N5L8	**Ribonuclease P protein subunit p25-like protein**	RPP25L
0,000	-3,27	0,10	**10,37**	Q99798	**Aconitate hydratase, mitochondrial**	ACO2
0,007	-3,29	0,10	**10,24**	Q17RN3	**Protein FAM98C**	FAM98C
0,008	-3,33	0,10	**9,95**	Q969Y2	**tRNA modification GTPase GTPBP3, mitochondrial**	GTPBP3
0,011	-3,35	0,10	**9,82**	Q92766	**Ras-responsive element-binding protein 1**	RREB1
0,032	-3,36	0,10	**9,77**	P07711	**Cathepsin L1;Cathepsin L1 heavy chain;Cathepsin L1 light chain**	CTSL
0,010	-3,36	0,10	**9,74**	Q49MG5	**Microtubule-associated protein 9**	MAP9
0,028	-3,38	0,10	**9,61**	O75843	**AP-1 complex subunit gamma-like 2**	AP1G2
0,048	-3,48	0,09	**8,96**	Q9HCN8	**Stromal cell-derived factor 2-like protein 1**	SDF2L1
0,036	-3,56	0,08	**8,48**	P58107	**Epiplakin**	EPPK1
0,034	-3,66	0,08	**7,90**	Q96TC7	**Regulator of microtubule dynamics protein 3**	RMDN3
0,000	-3,68	0,08	**7,81**	Q8NDT2	**Putative RNA-binding protein 15B**	RBM15B
0,001	-3,72	0,08	**7,59**	O00193	**Small acidic protein**	SMAP
0,017	-3,86	0,07	**6,89**	Q96S44	**TP53-regulating kinase**	TP53RK
0,050	-3,87	0,07	**6,84**	P52434	**DNA-directed RNA polymerases I, II, and III subunit RPABC3**	POLR2H
0,001	-3,91	0,07	**6,65**	Q8TDD1	**ATP-dependent RNA helicase DDX54**	DDX54
0,010	-3,94	0,07	**6,52**	Q9BPX5	**Actin-related protein 2/3 complex subunit 5-like protein**	ARPC5L

0,027	-4,21	0,05	**5,40**	Q70E73	**Ras-associated and pleckstrin homology domains-containing protein 1**	RAPH1
0,014	-4,28	0,05	**5,15**	P63272	**Transcription elongation factor SPT4**	SUPT4H1
0,035	-4,45	0,05	**4,58**	Q5K651	**Sterile alpha motif domain-containing protein 9**	SAMD9
0,000	-4,50	0,04	**4,42**	Q92626	**Peroxidasin homolog**	PXDN
0,003	-4,95	0,03	**3,24**	P30405	**Peptidyl-prolyl cis-trans isomerase F, mitochondrial**	PPIF
**24h Down**						
0,047	-0,12	0,92	**92,02**	Q9Y230	**RuvB-like 2**	RUVBL2
0,024	-0,12	0,92	**92,02**	Q9BY44	**Eukaryotic translation initiation factor 2A;Eukaryotic translation initiation factor 2A,**	EIF2A
0,005	-0,12	0,92	**91,79**	P06746	**DNA polymerase beta**	POLB
0,046	-0,16	0,90	**89,50**	Q9NXV6	**CDKN2A-interacting protein**	CDKN2AIP
0,022	-0,16	0,90	**89,50**	Q9Y265	**RuvB-like 1**	RUVBL1
0,019	-0,16	0,90	**89,50**	P50402	**Emerin**	EMD
0,017	-0,16	0,90	**89,50**	O95400	**CD2 antigen cytoplasmic tail-binding protein 2**	CD2BP2
0,044	-0,17	0,89	**88,93**	P61586	**Transforming protein RhoA**	RHOA
0,029	-0,17	0,89	**88,88**	Q12788	**Transducin beta-like protein 3**	TBL3
0,018	-0,19	0,88	**87,74**	P11940	**Polyadenylate-binding protein 1**	PABPC1
0,024	-0,19	0,88	**87,66**	O43809	**Cleavage and polyadenylation specificity factor subunit 5**	NUDT21
0,022	-0,19	0,87	**87,44**	Q9H2G2	**STE20-like serine/threonine-protein kinase**	SLK
0,001	-0,20	0,87	**87,25**	P45880	**Voltage-dependent anion-selective channel protein 2**	VDAC2
0,047	-0,20	0,87	**87,15**	Q86UP2	**Kinectin**	KTN1
0,019	-0,20	0,87	**87,06**	Q15046	**Lysine--tRNA ligase**	KARS
0,041	-0,20	0,87	**87,06**	Q7L014	**Probable ATP-dependent RNA helicase DDX46**	DDX46
0,019	-0,20	0,87	**87,06**	O14776	**Transcription elongation regulator 1**	TCERG1
0,010	-0,20	0,87	**86,86**	Q9UNM6	**26S proteasome non-ATPase regulatory subunit 13**	PSMD13
0,026	-0,21	0,86	**86,45**	Q92841	**Probable ATP-dependent RNA helicase DDX17**	DDX17
0,015	-0,22	0,86	**85,86**	P13010	**X-ray repair cross-complementing protein 5**	XRCC5
0,010	-0,22	0,86	**85,86**	P62136	**Serine/threonine-protein phosphatase PP1-alpha catalytic subunit**	PPP1CA
0,033	-0,22	0,86	**85,86**	Q16630	**Cleavage and polyadenylation specificity factor subunit 6**	CPSF6
0,015	-0,23	0,85	**85,26**	Q9UHD8	**Septin-9**	40057,000
0,024	-0,23	0,85	**85,26**	Q15287	**RNA-binding protein with serine-rich domain 1**	RNPS1
0,027	-0,24	0,85	**84,85**	P04843	**Dolichyl-diphosphooligosaccharide--protein glycosyltransferase subunit 1**	RPN1
0,001	-0,24	0,85	**84,71**	Q9NVT9	**Armadillo repeat-containing protein 1**	ARMC1
0,009	-0,24	0,85	**84,67**	Q9UQB8	**Brain-specific angiogenesis inhibitor 1-associated protein 2**	BAIAP2

0,001	-0,24	0,85	**84,67**	Q5T280	**Uncharacterized protein C9orf114**	C9orf114
0,029	-0,25	0,84	**83,91**	Q15365	**Poly(rC)-binding protein 1**	PCBP1
0,014	-0,26	0,84	**83,70**	P26640	**Valine--tRNA ligase**	VARS
0,033	-0,26	0,84	**83,51**	Q13573	**SNW domain-containing protein 1**	SNW1
0,025	-0,27	0,83	**83,17**	P22102	**Trifunctional purine biosynthetic protein adenosine-3;Phosphoribosylamine--glycine**	GART
0,024	-0,28	0,83	**82,63**	Q9BZK7	**F-box-like/WD repeat-containing protein TBL1XR1**	TBL1XR1
0,043	-0,28	0,83	**82,52**	P28482	**Mitogen-activated protein kinase 1**	MAPK1
0,033	-0,28	0,82	**82,48**	Q86XP3	**ATP-dependent RNA helicase DDX42**	DDX42
0,024	-0,28	0,82	**82,45**	P12931	**Proto-oncogene tyrosine-protein kinase Src**	SRC
0,009	-0,28	0,82	**82,36**	Q92820	**Gamma-glutamyl hydrolase**	GGH
0,042	-0,29	0,82	**82,04**	P48444	**Coatomer subunit delta**	ARCN1
0,011	-0,29	0,82	**82,02**	Q9P2E9	**Ribosome-binding protein 1**	RRBP1
0,007	-0,29	0,82	**81,79**	P41223	**Protein BUD31 homolog**	BUD31
0,002	-0,30	0,81	**81,23**	Q96QK1	**Vacuolar protein sorting-associated protein 35**	VPS35
0,036	-0,30	0,81	**81,23**	P61978	**Heterogeneous nuclear ribonucleoprotein K**	HNRNPK
0,006	-0,30	0,81	**81,04**	P54136	**Arginine--tRNA ligase, cytoplasmic**	RARS
0,039	-0,31	0,81	**80,66**	Q8TEQ6	**Gem-associated protein 5**	GEMIN5
0,004	-0,31	0,81	**80,66**	P35637	**RNA-binding protein FUS**	FUS
0,012	-0,31	0,81	**80,63**	Q9Y5S2	**Serine/threonine-protein kinase MRCK beta**	CDC42BPB
0,015	-0,31	0,81	**80,60**	Q00610	**Clathrin heavy chain 1**	CLTC
0,049	-0,31	0,80	**80,45**	Q9UMX0	**Ubiquilin-1**	UBQLN1
0,030	-0,32	0,80	**80,37**	P26196	**Probable ATP-dependent RNA helicase DDX6**	DDX6
0,014	-0,32	0,80	**80,11**	P78347	**General transcription factor II-I**	GTF2I
0,024	-0,32	0,80	**80,11**	P49792	**E3 SUMO-protein ligase RanBP2**	RANBP2
0,038	-0,32	0,80	**80,11**	Q13503	**Mediator of RNA polymerase II transcription subunit 21**	MED21
0,015	-0,33	0,80	**79,72**	P49327	**Fatty acid synthase;[Acyl-carrier-protein] S-acetyltransferase;[Acyl-carrier-protein] S**	FASN
0,014	-0,33	0,80	**79,55**	P63279	**SUMO-conjugating enzyme UBC9**	UBE2I
0,049	-0,33	0,80	**79,55**	P09497	**Clathrin light chain B**	CLTB
0,031	-0,33	0,80	**79,55**	Q9Y2W2	**WW domain-binding protein 11**	WBP11
0,039	-0,33	0,80	**79,51**	Q5T6F2	**Ubiquitin-associated protein 2**	UBAP2
0,011	-0,33	0,79	**79,39**	Q9NZB2	**Constitutive coactivator of PPAR-gamma-like protein 1**	FAM120A
0,046	-0,34	0,79	**79,28**	P61970	**Nuclear transport factor 2**	NUTF2
0,025	-0,34	0,79	**78,99**	O00203	**AP-3 complex subunit beta-1**	AP3B1

0,044	-0,35	0,79	**78,60**	P42166	**Lamina-associated polypeptide 2, isoform alpha;Thymopoietin;Thymopentin**	TMPO
0,016	-0,35	0,79	**78,60**	Q9UI10	**Translation initiation factor eIF-2B subunit delta**	EIF2B4
0,004	-0,35	0,79	**78,51**	Q99613;B5ME19	**Eukaryotic translation initiation factor 3 subunit C;Eukaryotic translation initiation f**	EIF3C;EIF3CL
0,011	-0,35	0,78	**78,46**	O00571;O15523	**ATP-dependent RNA helicase DDX3X;ATP-dependent RNA helicase DDX3Y**	DDX3X;DDX3Y
0,002	-0,35	0,78	**78,34**	Q9UMS4	**Pre-mRNA-processing factor 19**	PRPF19
0,029	-0,35	0,78	**78,27**	Q8WX93	**Palladin**	PALLD
0,004	-0,35	0,78	**78,27**	P46060	**Ran GTPase-activating protein 1**	RANGAP1
0,042	-0,35	0,78	**78,19**	P00918	**Carbonic anhydrase 2**	CA2
0,000	-0,35	0,78	**78,19**	P38432	**Coilin**	COIL
0,039	-0,36	0,78	**77,92**	P52926	**High mobility group protein HMGI-C**	HMGA2
0,039	-0,36	0,78	**77,92**	Q13685	**Angio-associated migratory cell protein**	AAMP
0,010	-0,36	0,78	**77,92**	O00148	**ATP-dependent RNA helicase DDX39A**	DDX39A
0,038	-0,36	0,78	**77,92**	O75083	**WD repeat-containing protein 1**	WDR1
0,023	-0,36	0,78	**77,90**	O43684	**Mitotic checkpoint protein BUB3**	BUB3
0,019	-0,36	0,78	**77,85**	Q9P031	**Thyroid transcription factor 1-associated protein 26**	CCDC59
0,021	-0,36	0,78	**77,84**	Q99873	**Protein arginine N-methyltransferase 1**	PRMT1
0,046	-0,37	0,78	**77,54**	Q8TD47	**40S ribosomal protein S4, Y isoform 2**	RPS4Y2
0,013	-0,37	0,77	**77,38**	P61247	**40S ribosomal protein S3a**	RPS3A
0,031	-0,37	0,77	**77,35**	P52597	**Heterogeneous nuclear ribonucleoprotein F;Heterogeneous nuclear ribonucleoprote**	HNRNPF
0,015	-0,37	0,77	**77,22**	Q00341	**Vigilin**	HDLBP
0,046	-0,37	0,77	**77,16**	P35222	**Catenin beta-1**	CTNNB1
0,020	-0,38	0,77	**77,08**	Q14152	**Eukaryotic translation initiation factor 3 subunit A**	EIF3A
0,014	-0,38	0,77	**76,87**	P48047	**ATP synthase subunit O, mitochondrial**	ATP5O
0,017	-0,38	0,77	**76,84**	Q9BY77	**Polymerase delta-interacting protein 3**	POLDIP3
0,048	-0,38	0,77	**76,84**	Q7Z3B4	**Nucleoporin p54**	NUP54
0,023	-0,38	0,77	**76,84**	Q4LE39	**AT-rich interactive domain-containing protein 4B**	ARID4B
0,034	-0,38	0,77	**76,68**	Q9Y5A9	**YTH domain-containing family protein 2**	YTHDF2
0,009	-0,38	0,77	**76,64**	O15031	**Plexin-B2**	PLXNB2
0,026	-0,39	0,77	**76,55**	Q9H4A4	**Aminopeptidase B**	RNPEP
0,021	-0,39	0,76	**76,48**	P56192	**Methionine--tRNA ligase, cytoplasmic**	MARS
0,016	-0,39	0,76	**76,31**	Q9HCN4	**GPN-loop GTPase 1**	GPN1
0,023	-0,39	0,76	**76,31**	Q8N9M1	**Uncharacterized protein C19orf47**	C19orf47
0,030	-0,39	0,76	**76,31**	Q14839	**Chromodomain-helicase-DNA-binding protein 4**	CHD4

0,049	-0,40	0,76	**75,99**	P04181	**Ornithine aminotransferase, mitochondrial;Ornithine aminotransferase, hepatic for**	OAT
0,031	-0,40	0,76	**75,82**	P61163	**Alpha-centractin**	ACTR1A
0,028	-0,40	0,76	**75,82**	P19388	**DNA-directed RNA polymerases I, II, and III subunit RPABC1**	POLR2E
0,041	-0,40	0,76	**75,79**	Q13347	**Eukaryotic translation initiation factor 3 subunit I**	EIF3I
0,013	-0,40	0,76	**75,79**	Q9P0U4	**CXXC-type zinc finger protein 1**	CXXC1
0,000	-0,40	0,76	**75,79**	Q9ULL5	**Proline-rich protein 12**	PRR12
0,030	-0,40	0,76	**75,76**	Q92506	**Estradiol 17-beta-dehydrogenase 8**	HSD17B8
0,015	-0,40	0,76	**75,55**	P60842	**Eukaryotic initiation factor 4A-I**	EIF4A1
0,027	-0,40	0,76	**75,55**	Q96S99	**Pleckstrin homology domain-containing family F member 1**	PLEKHF1
0,000	-0,41	0,75	**75,43**	Q96GX2	**Putative ataxin-7-like protein 3B**	ATXN7L3B
0,030	-0,41	0,75	**75,34**	Q92541	**RNA polymerase-associated protein RTF1 homolog**	RTF1
0,008	-0,41	0,75	**75,29**	Q9H6S0	**Probable ATP-dependent RNA helicase YTHDC2**	YTHDC2
0,009	-0,41	0,75	**75,26**	P35611	**Alpha-adducin**	ADD1
0,003	-0,41	0,75	**75,17**	P07602	**Prosaposin;Saposin-A;Saposin-B-Val;Saposin-B;Saposin-C;Saposin-D**	PSAP
0,049	-0,41	0,75	**75,11**	P04899	**Guanine nucleotide-binding protein G(i) subunit alpha-2**	GNAI2
0,001	-0,41	0,75	**75,04**	Q9UDY2	**Tight junction protein ZO-2**	TJP2
0,011	-0,42	0,75	**74,74**	Q9BZH6	**WD repeat-containing protein 11**	WDR11
0,035	-0,42	0,75	**74,72**	Q99615	**DnaJ homolog subfamily C member 7**	DNAJC7
0,017	-0,42	0,75	**74,67**	P46379	**Large proline-rich protein BAG6**	BAG6
0,045	-0,42	0,75	**74,61**	Q9P265	**Disco-interacting protein 2 homolog B**	DIP2B
0,033	-0,43	0,74	**74,44**	Q9UJS0	**Calcium-binding mitochondrial carrier protein Aralar2**	SLC25A13
0,031	-0,43	0,74	**74,34**	Q9H6S3	**Epidermal growth factor receptor kinase substrate 8-like protein 2**	EPS8L2
0,015	-0,43	0,74	**74,23**	Q15084	**Protein disulfide-isomerase A6**	PDIA6
0,016	-0,43	0,74	**74,23**	P35249	**Replication factor C subunit 4**	RFC4
0,038	-0,43	0,74	**74,23**	P30050	**60S ribosomal protein L12**	RPL12
0,011	-0,44	0,74	**73,71**	P62913	**60S ribosomal protein L11**	RPL11
0,002	-0,44	0,74	**73,71**	P63173	**60S ribosomal protein L38**	RPL38
0,044	-0,44	0,74	**73,71**	Q02543	**60S ribosomal protein L18a**	RPL18A
0,014	-0,44	0,74	**73,59**	P60900	**Proteasome subunit alpha type-6**	PSMA6
0,040	-0,45	0,73	**73,36**	O95470	**Sphingosine-1-phosphate lyase 1**	SGPL1
0,034	-0,45	0,73	**73,29**	Q6VY07	**Phosphofurin acidic cluster sorting protein 1**	PACS1
0,049	-0,45	0,73	**73,28**	O60869	**Endothelial differentiation-related factor 1**	EDF1
0,010	-0,45	0,73	**73,20**	P42285	**Superkiller viralicidic activity 2-like 2**	SKIV2L2

0,033	-0,45	0,73	**73,20**	Q13523	**Serine/threonine-protein kinase PRP4 homolog**	PRPF4B
0,011	-0,45	0,73	**73,17**	Q15031	**Probable leucine--tRNA ligase, mitochondrial**	LARS2
0,028	-0,45	0,73	**73,08**	P61011	**Signal recognition particle 54 kDa protein**	SRP54
0,008	-0,45	0,73	**73,00**	P52948	**Nuclear pore complex protein Nup98-Nup96;Nuclear pore complex protein Nup98;N**	NUP98
0,030	-0,46	0,73	**72,94**	Q9NSK7	**Protein C19orf12**	C19orf12
0,049	-0,46	0,73	**72,87**	P17931	**Galectin-3**	LGALS3
0,003	-0,46	0,73	**72,70**	Q8WWQ0	**PH-interacting protein**	PHIP
0,013	-0,46	0,73	**72,70**	Q92522	**Histone H1x**	H1FX
0,031	-0,46	0,73	**72,70**	Q9ULW0	**Targeting protein for Xklp2**	TPX2
0,033	-0,46	0,73	**72,59**	O95793	**Double-stranded RNA-binding protein Staufen homolog 1**	STAU1
0,050	-0,46	0,73	**72,54**	Q9Y4W2	**Ribosomal biogenesis protein LAS1L**	LAS1L
0,004	-0,46	0,72	**72,49**	O43852	**Calumenin**	CALU
0,032	-0,47	0,72	**72,25**	Q9BQE3	**Tubulin alpha-1C chain**	TUBA1C
0,010	-0,47	0,72	**72,22**	Q92973	**Transportin-1**	TNPO1
0,001	-0,47	0,72	**72,20**	Q7Z6R9	**Transcription factor AP-2-delta**	TFAP2D
0,014	-0,47	0,72	**72,20**	Q00839	**Heterogeneous nuclear ribonucleoprotein U**	HNRNPU
0,006	-0,47	0,72	**72,20**	P62158	**Calmodulin**	CALM1
0,026	-0,47	0,72	**72,20**	P55084	**Trifunctional enzyme subunit beta, mitochondrial;3-ketoacyl-CoA thiolase**	HADHB
0,029	-0,47	0,72	**71,99**	P56545	**C-terminal-binding protein 2**	CTBP2
0,014	-0,47	0,72	**71,96**	Q01844	**RNA-binding protein EWS**	EWSR1
0,010	-0,48	0,72	**71,74**	Q8N1F7	**Nuclear pore complex protein Nup93**	NUP93
0,019	-0,48	0,72	**71,70**	P41567;O60739	**Eukaryotic translation initiation factor 1;Eukaryotic translation initiation factor 1b**	EIF1;EIF1B
0,029	-0,48	0,72	**71,70**	P57772	**Selenocysteine-specific elongation factor**	EEFSEC
0,004	-0,48	0,72	**71,70**	P62888	**60S ribosomal protein L30**	RPL30
0,049	-0,48	0,71	**71,47**	O75663	**TIP41-like protein**	TIPRL
0,009	-0,49	0,71	**71,31**	Q9H6T3	**RNA polymerase II-associated protein 3**	RPAP3
0,036	-0,49	0,71	**71,20**	Q9NUD5	**Zinc finger CCHC domain-containing protein 3**	ZCCHC3
0,006	-0,49	0,71	**71,20**	P32969	**60S ribosomal protein L9**	RPL9
0,037	-0,49	0,71	**71,20**	Q10570	**Cleavage and polyadenylation specificity factor subunit 1**	CPSF1
0,016	-0,49	0,71	**71,20**	Q86V48	**Leucine zipper protein 1**	LUZP1
0,021	-0,50	0,71	**70,82**	Q92905	**COP9 signalosome complex subunit 5**	COPS5
0,030	-0,50	0,71	**70,71**	Q6EEV6;P61956;P55854	**Small ubiquitin-related modifier 4;Small ubiquitin-related modifier 2;Small ubiquitir**	MO4;SUMO2;SUM
0,028	-0,50	0,71	**70,71**	P39023	**60S ribosomal protein L3**	RPL3

0,030	-0,50	0,71	**70,71**	Q5JVS0	**Intracellular hyaluronan-binding protein 4**	HABP4
0,025	-0,50	0,70	**70,47**	Q6EEV4	**DNA-directed RNA polymerase II subunit GRINL1A, isoforms 4/5**	POLR2M
0,006	-0,51	0,70	**70,42**	Q9Y3D8	**Adenylate kinase isoenzyme 6**	AK6
0,018	-0,51	0,70	**70,33**	Q86VP6	**Cullin-associated NEDD8-dissociated protein 1**	CAND1
0,001	-0,51	0,70	**70,28**	Q9UHD1	**Cysteine and histidine-rich domain-containing protein 1**	CHORDC1
0,016	-0,51	0,70	**70,26**	A0FGR8	**Extended synaptotagmin-2**	ESYT2
0,001	-0,51	0,70	**70,22**	Q2TAK8	**PWWP domain-containing protein MUM1**	MUM1
0,005	-0,51	0,70	**70,22**	Q9UPT8	**Zinc finger CCCH domain-containing protein 4**	ZC3H4
0,015	-0,51	0,70	**70,22**	P11233	**Ras-related protein Ral-A**	RALA
0,009	-0,51	0,70	**70,22**	P47897	**Glutamine--tRNA ligase**	QARS
0,027	-0,51	0,70	**70,20**	P07737	**Profilin-1**	PFN1
0,022	-0,52	0,70	**69,85**	O95373	**Importin-7**	IPO7
0,038	-0,52	0,70	**69,78**	O15371	**Eukaryotic translation initiation factor 3 subunit D**	EIF3D
0,005	-0,52	0,70	**69,74**	Q9UHV9	**Prefoldin subunit 2**	PFDN2
0,009	-0,52	0,70	**69,74**	Q8WVK2	**U4/U6.U5 small nuclear ribonucleoprotein 27 kDa protein**	SNRNP27
0,027	-0,52	0,70	**69,74**	Q96BK5	**PIN2/TERF1-interacting telomerase inhibitor 1**	PINX1
0,045	-0,53	0,69	**69,47**	Q9BPX6	**Calcium uptake protein 1, mitochondrial**	MICU1
0,007	-0,53	0,69	**69,33**	Q99798	**Aconitate hydratase, mitochondrial**	ACO2
0,003	-0,53	0,69	**69,26**	Q8WUZ0	**B-cell CLL/lymphoma 7 protein family member C**	BCL7C
0,017	-0,53	0,69	**69,26**	Q9UN86	**Ras GTPase-activating protein-binding protein 2**	G3BP2
0,036	-0,53	0,69	**69,26**	P24534	**Elongation factor 1-beta**	EEF1B2
0,021	-0,53	0,69	**69,20**	Q76FK4	**Nucleolar protein 8**	NOL8
0,009	-0,53	0,69	**69,11**	Q12888	**Tumor suppressor p53-binding protein 1**	TP53BP1
0,032	-0,53	0,69	**69,05**	Q9UHD9	**Ubiquilin-2**	UBQLN2
0,023	-0,53	0,69	**69,04**	P54819	**Adenylate kinase 2, mitochondrial;Adenylate kinase 2, mitochondrial, N-terminally**	AK2
0,040	-0,54	0,69	**68,87**	O00116	**Alkyldihydroxyacetonephosphate synthase, peroxisomal**	AGPS
0,000	-0,54	0,69	**68,84**	P35221	**Catenin alpha-1**	CTNNA1
0,003	-0,54	0,69	**68,78**	Q13308	**Inactive tyrosine-protein kinase 7**	PTK7
0,040	-0,54	0,69	**68,78**	P07437	**Tubulin beta chain**	TUBB
0,014	-0,54	0,69	**68,78**	Q15637	**Splicing factor 1**	SF1
0,005	-0,54	0,69	**68,78**	A6NHR9	**Structural maintenance of chromosomes flexible hinge domain-containing protein 1**	SMCHD1
0,002	-0,54	0,69	**68,78**	Q9UQ80	**Proliferation-associated protein 2G4**	PA2G4
0,039	-0,54	0,69	**68,78**	P25490	**Transcriptional repressor protein YY1**	YY1

0,028	-0,54	0,69	**68,78**	P28370	**Probable global transcription activator SNF2L1**	SMARCA1
0,046	-0,54	0,69	**68,72**	P39656	**Dolichyl-diphosphooligosaccharide--protein glycosyltransferase 48 kDa subunit**	DDOST
0,026	-0,54	0,69	**68,59**	Q9NP77	**RNA polymerase II subunit A C-terminal domain phosphatase SSU72**	SSU72
0,030	-0,55	0,68	**68,43**	O75616	**GTPase Era, mitochondrial**	ERAL1
0,036	-0,55	0,68	**68,42**	Q96TA1	**Niban-like protein 1**	FAM129B
0,021	-0,55	0,68	**68,41**	P21266	**Glutathione S-transferase Mu 3**	GSTM3
0,022	-0,55	0,68	**68,30**	Q8TAQ2	**SWI/SNF complex subunit SMARCC2**	SMARCC2
0,046	-0,55	0,68	**68,30**	Q00403	**Transcription initiation factor IIB**	GTF2B
0,001	-0,55	0,68	**68,30**	Q5TA45	**Integrator complex subunit 11**	CPSF3L
0,014	-0,55	0,68	**68,30**	Q7Z3B3	**KAT8 regulatory NSL complex subunit 1**	KANSL1
0,003	-0,55	0,68	**68,26**	P68371	**Tubulin beta-4B chain**	TUBB4B
0,045	-0,55	0,68	**68,23**	P00491	**Purine nucleoside phosphorylase**	PNP
0,004	-0,56	0,68	**67,88**	Q5T1M5	**FK506-binding protein 15**	FKBP15
0,041	-0,56	0,68	**67,83**	Q9BVC4	**Target of rapamycin complex subunit LST8**	MLST8
0,012	-0,56	0,68	**67,83**	Q14157	**Ubiquitin-associated protein 2-like**	UBAP2L
0,014	-0,56	0,68	**67,83**	O15164	**Transcription intermediary factor 1-alpha**	TRIM24
0,003	-0,56	0,68	**67,83**	Q9BXB4	**Oxysterol-binding protein-related protein 11**	OSBPL11
0,017	-0,56	0,68	**67,83**	Q14694	**Ubiquitin carboxyl-terminal hydrolase 10**	USP10
0,007	-0,56	0,68	**67,83**	Q15906	**Vacuolar protein sorting-associated protein 72 homolog**	VPS72
0,012	-0,56	0,68	**67,80**	Q9H3G5	**Probable serine carboxypeptidase CPVL**	CPVL
0,033	-0,56	0,68	**67,61**	P08047	**Transcription factor Sp1**	SP1
0,004	-0,56	0,68	**67,60**	Q9Y277	**Voltage-dependent anion-selective channel protein 3**	VDAC3
0,011	-0,57	0,67	**67,49**	P40855	**Peroxisomal biogenesis factor 19**	PEX19
0,014	-0,57	0,67	**67,46**	Q9HB07	**UPF0160 protein MYG1, mitochondrial**	C12orf10
0,005	-0,57	0,67	**67,36**	Q96S94	**Cyclin-L2**	CCNL2
0,045	-0,57	0,67	**67,36**	Q06124	**Tyrosine-protein phosphatase non-receptor type 11**	PTPN11
0,023	-0,57	0,67	**67,36**	O15372	**Eukaryotic translation initiation factor 3 subunit H**	EIF3H
0,022	-0,57	0,67	**67,25**	P15144	**Aminopeptidase N**	ANPEP
0,019	-0,58	0,67	**67,12**	Q01085	**Nucleolysin TIAR**	TIAL1
0,011	-0,58	0,67	**67,00**	Q7Z434	**Mitochondrial antiviral-signaling protein**	MAVS
0,010	-0,58	0,67	**66,93**	Q14696	**LDLR chaperone MESD**	MESDC2
0,012	-0,58	0,67	**66,92**	Q15628	**Tumor necrosis factor receptor type 1-associated DEATH domain protein**	TRADD
0,020	-0,58	0,67	**66,90**	P63244	**Guanine nucleotide-binding protein subunit beta-2-like 1;Guanine nucleotide-bindir**	GNB2L1

0,007	-0,58	0,67	**66,90**	Q6IQ23	**Pleckstrin homology domain-containing family A member 7**	PLEKHA7
0,013	-0,58	0,67	**66,90**	Q96GK7	**Fumarylacetoacetate hydrolase domain-containing protein 2A**	FAHD2A
0,015	-0,58	0,67	**66,90**	P62424	**60S ribosomal protein L7a**	RPL7A
0,005	-0,58	0,67	**66,90**	Q9GZS1	**DNA-directed RNA polymerase I subunit RPA49**	POLR1E
0,043	-0,59	0,67	**66,54**	Q14671	**Pumilio homolog 1**	PUM1
0,042	-0,59	0,66	**66,43**	P22033	**Methylmalonyl-CoA mutase, mitochondrial**	MUT
0,032	-0,59	0,66	**66,43**	O60220	**Mitochondrial import inner membrane translocase subunit Tim8 A**	TIMM8A
0,021	-0,59	0,66	**66,43**	P08134	**Rho-related GTP-binding protein RhoC**	RHOC
0,002	-0,59	0,66	**66,43**	Q12792	**Twinfilin-1**	TWF1
0,048	-0,59	0,66	**66,43**	Q9Y6X9	**MORC family CW-type zinc finger protein 2**	MORC2
0,002	-0,59	0,66	**66,43**	P20290	**Transcription factor BTF3**	BTF3
0,011	-0,59	0,66	**66,43**	P61254	**60S ribosomal protein L26**	RPL26
0,034	-0,59	0,66	**66,27**	Q9NQR4	**Omega-amidase NIT2**	NIT2
0,050	-0,59	0,66	**66,21**	P61224;A6NIZ1	**Ras-related protein Rap-1b;Ras-related protein Rap-1b-like protein**	RAP1B
0,047	-0,60	0,66	**66,14**	Q9Y508	**E3 ubiquitin-protein ligase RNF114**	RNF114
0,009	-0,60	0,66	**65,98**	Q8WUM0	**Nuclear pore complex protein Nup133**	NUP133
0,017	-0,60	0,66	**65,98**	O15042	**U2 snRNP-associated SURP motif-containing protein**	U2SURP
0,014	-0,60	0,66	**65,98**	Q6P3W7	**SCY1-like protein 2**	SCYL2
0,026	-0,60	0,66	**65,98**	P98175	**RNA-binding protein 10**	RBM10
0,030	-0,60	0,66	**65,98**	Q7L4I2	**Arginine/serine-rich coiled-coil protein 2**	RSRC2
0,026	-0,60	0,66	**65,85**	Q9BWF3	**RNA-binding protein 4**	RBM4
0,047	-0,61	0,66	**65,63**	P35914	**Hydroxymethylglutaryl-CoA lyase, mitochondrial**	HMGCL
0,004	-0,61	0,66	**65,52**	P50995	**Annexin A11**	ANXA11
0,014	-0,61	0,66	**65,52**	Q4VCS5	**Angiomotin**	AMOT
0,032	-0,61	0,66	**65,52**	Q7Z4W1	**L-xylulose reductase**	DCXR
0,011	-0,61	0,66	**65,52**	P07195	**L-lactate dehydrogenase B chain**	LDHB
0,047	-0,61	0,66	**65,52**	Q15436	**Protein transport protein Sec23A**	SEC23A
0,036	-0,61	0,65	**65,39**	P10599	**Thioredoxin**	TXN
0,049	-0,61	0,65	**65,32**	P11047	**Laminin subunit gamma-1**	LAMC1
0,021	-0,62	0,65	**65,18**	Q9NVS9	**Pyridoxine-5-phosphate oxidase**	PNPO
0,029	-0,62	0,65	**65,18**	Q92878	**DNA repair protein RAD50**	RAD50
0,012	-0,62	0,65	**65,07**	P18077	**60S ribosomal protein L35a**	RPL35A
0,000	-0,62	0,65	**65,07**	P17844	**Probable ATP-dependent RNA helicase DDX5**	DDX5

0,048	-0,62	0,65	**65,07**	Q9BYG5	**Partitioning defective 6 homolog beta**	PARD6B
0,045	-0,62	0,65	**65,07**	979;P62987;P0CG47;P0(	**Ubiquitin-40S ribosomal protein S27a;Ubiquitin;40S ribosomal protein S27a;Ubiquit**	S27A;UBA52;UBB;U
0,004	-0,62	0,65	**65,07**	P62753	**40S ribosomal protein S6**	RPS6
0,014	-0,62	0,65	**65,07**	Q5T200	**Zinc finger CCCH domain-containing protein 13**	ZC3H13
0,008	-0,62	0,65	**65,00**	P05141	**ADP/ATP translocase 2;ADP/ATP translocase 2, N-terminally processed**	SLC25A5
0,049	-0,62	0,65	**64,91**	Q9UBF2	**Coatomer subunit gamma-2**	COPG2
0,035	-0,63	0,65	**64,72**	O96019	**Actin-like protein 6A**	ACTL6A
0,014	-0,63	0,65	**64,64**	Q99805	**Transmembrane 9 superfamily member 2**	TM9SF2
0,027	-0,63	0,65	**64,62**	P57740	**Nuclear pore complex protein Nup107**	NUP107
0,004	-0,63	0,65	**64,53**	Q16763	**Ubiquitin-conjugating enzyme E2 S**	UBE2S
0,038	-0,63	0,64	**64,44**	Q53H96	**Pyrroline-5-carboxylate reductase 3**	PYCRL
0,050	-0,63	0,64	**64,40**	Q7Z2W9	**39S ribosomal protein L21, mitochondrial**	MRPL21
0,007	-0,64	0,64	**64,22**	Q96RU2	**Ubiquitin carboxyl-terminal hydrolase 28**	USP28
0,031	-0,64	0,64	**64,17**	P26373	**60S ribosomal protein L13**	RPL13
0,000	-0,64	0,64	**64,17**	P68104;Q5VTE0	**Elongation factor 1-alpha 1;Putative elongation factor 1-alpha-like 3**	EEF1A1;EEF1A1P5
0,007	-0,64	0,64	**64,17**	P08865	**40S ribosomal protein SA**	RPSA
0,018	-0,64	0,64	**64,17**	P62906	**60S ribosomal protein L10a**	RPL10A
0,027	-0,64	0,64	**63,99**	O75179	**Ankyrin repeat domain-containing protein 17**	ANKRD17
0,035	-0,64	0,64	**63,95**	Q96S59	**Ran-binding protein 9**	RANBP9
0,004	-0,65	0,64	**63,82**	Q56VL3	**OCIA domain-containing protein 2**	OCIAD2
0,050	-0,65	0,64	**63,79**	P20585	**DNA mismatch repair protein Msh3**	MSH3
0,007	-0,65	0,64	**63,78**	O00391	**Sulfhydryl oxidase 1**	QSOX1
0,042	-0,65	0,64	**63,75**	P14923	**Junction plakoglobin**	JUP
0,025	-0,65	0,64	**63,73**	Q9BX68	**Histidine triad nucleotide-binding protein 2, mitochondrial**	HINT2
0,001	-0,65	0,64	**63,73**	Q9NWH9	**SAFB-like transcription modulator**	SLTM
0,007	-0,65	0,64	**63,73**	Q8NI77	**Kinesin-like protein KIF18A**	KIF18A
0,006	-0,65	0,64	**63,73**	P62851	**40S ribosomal protein S25**	RPS25
0,023	-0,66	0,63	**63,46**	P32970	**CD70 antigen**	CD70
0,015	-0,66	0,63	**63,29**	P62847	**40S ribosomal protein S24**	RPS24
0,012	-0,66	0,63	**63,29**	P69849;Q5JPE7;Q15155	**Nodal modulator 3;Nodal modulator 2;Nodal modulator 1**	MO3;NOMO2;NOM
0,008	-0,66	0,63	**63,29**	Q9BZZ5	**Apoptosis inhibitor 5**	API5
0,004	-0,66	0,63	**63,29**	Q96C36	**Pyrroline-5-carboxylate reductase 2**	PYCR2
0,019	-0,66	0,63	**63,29**	O75436	**Vacuolar protein sorting-associated protein 26A**	VPS26A

0,041	-0,66	0,63	**63,29**	P06396	**Gelsolin**	GSN
0,037	-0,66	0,63	**63,29**	Q04637	**Eukaryotic translation initiation factor 4 gamma 1**	EIF4G1
0,015	-0,66	0,63	**63,29**	Q13765;E9PAV3	**Nascent polypeptide-associated complex subunit alpha;Nascent polypeptide-associa**	NACA
0,022	-0,66	0,63	**63,29**	Q02880	**DNA topoisomerase 2-beta**	TOP2B
0,000	-0,66	0,63	**63,24**	P35606	**Coatomer subunit beta**	COPB2
0,025	-0,66	0,63	**63,21**	Q16204	**Coiled-coil domain-containing protein 6**	CCDC6
0,017	-0,67	0,63	**63,03**	Q14160	**Protein scribble homolog**	SCRIB
0,007	-0,67	0,63	**63,02**	Q9H8W4	**Pleckstrin homology domain-containing family F member 2**	PLEKHF2
0,021	-0,67	0,63	**63,02**	Q9UKK3	**Poly [ADP-ribose] polymerase 4**	PARP4
0,014	-0,67	0,63	**62,96**	P10398	**Serine/threonine-protein kinase A-Raf**	ARAF
0,007	-0,67	0,63	**62,94**	Q9NYV4	**Cyclin-dependent kinase 12**	CDK12
0,028	-0,67	0,63	**62,93**	P51571	**Translocon-associated protein subunit delta**	SSR4
0,006	-0,67	0,63	**62,91**	Q9Y6X8	**Zinc fingers and homeoboxes protein 2**	ZHX2
0,024	-0,67	0,63	**62,85**	O75151	**Lysine-specific demethylase PHF2**	PHF2
0,026	-0,67	0,63	**62,85**	P27708	**CAD protein;Glutamine-dependent carbamoyl-phosphate synthase;Aspartate carba**	CAD
0,007	-0,67	0,63	**62,78**	Q13200	**26S proteasome non-ATPase regulatory subunit 2**	PSMD2
0,036	-0,67	0,63	**62,76**	P15170;Q8IYD1	**Eukaryotic peptide chain release factor GTP-binding subunit ERF3A;Eukaryotic pepti**	GSPT1;GSPT2
0,009	-0,67	0,63	**62,76**	Q15643	**Thyroid receptor-interacting protein 11**	TRIP11
0,026	-0,68	0,62	**62,42**	P51858	**Hepatoma-derived growth factor**	HDGF
0,024	-0,68	0,62	**62,42**	Q96FW1	**Ubiquitin thioesterase OTUB1**	OTUB1
0,013	-0,68	0,62	**62,42**	P78527	**DNA-dependent protein kinase catalytic subunit**	PRKDC
0,014	-0,68	0,62	**62,42**	P07942	**Laminin subunit beta-1**	LAMB1
0,023	-0,68	0,62	**62,42**	P40429;Q6NVV1	**60S ribosomal protein L13a;Putative 60S ribosomal protein L13a protein RPL13AP3**	RPL13A;RPL13AP3
0,005	-0,68	0,62	**62,42**	P46783;Q9NQ39	**40S ribosomal protein S10;Putative 40S ribosomal protein S10-like**	RPS10;RPS10P5
0,003	-0,68	0,62	**62,42**	P42677	**40S ribosomal protein S27**	RPS27
0,034	-0,68	0,62	**62,42**	O95785	**Protein Wiz**	WIZ
0,021	-0,68	0,62	**62,36**	Q15654	**Thyroid receptor-interacting protein 6**	TRIP6
0,017	-0,69	0,62	**62,14**	Q13616	**Cullin-1**	CUL1
0,001	-0,69	0,62	**62,10**	A0AV96	**RNA-binding protein 47**	RBM47
0,020	-0,69	0,62	**61,99**	Q9BXS5	**AP-1 complex subunit mu-1**	AP1M1
0,026	-0,69	0,62	**61,99**	O75718	**Cartilage-associated protein**	CRTAP
0,005	-0,69	0,62	**61,99**	O94776	**Metastasis-associated protein MTA2**	MTA2
0,032	-0,69	0,62	**61,99**	Q8NDX5	**Polyhomeotic-like protein 3**	PHC3

0,049	-0,69	0,62	**61,99**	P53350	**Serine/threonine-protein kinase PLK1**	PLK1
0,012	-0,70	0,62	**61,70**	Q86WR0	**Coiled-coil domain-containing protein 25**	CCDC25
0,005	-0,70	0,62	**61,56**	P52594	**Arf-GAP domain and FG repeat-containing protein 1**	AGFG1
0,009	-0,70	0,62	**61,56**	Q9BZ95	**Histone-lysine N-methyltransferase NSD3**	WHSC1L1
0,019	-0,70	0,62	**61,56**	Q14004	**Cyclin-dependent kinase 13**	CDK13
0,008	-0,70	0,62	**61,56**	O00178	**GTP-binding protein 1**	GTPBP1
0,036	-0,70	0,62	**61,56**	P62699	**Protein yippee-like 5**	YPEL5
0,018	-0,70	0,62	**61,55**	P55011	**Solute carrier family 12 member 2**	SLC12A2
0,021	-0,70	0,61	**61,37**	P54105	**Methylosome subunit pICln**	CLNS1A
0,015	-0,71	0,61	**61,26**	P17706	**Tyrosine-protein phosphatase non-receptor type 2**	PTPN2
0,017	-0,71	0,61	**61,15**	Q8N6H7	**ADP-ribosylation factor GTPase-activating protein 2**	ARFGAP2
0,028	-0,71	0,61	**61,13**	Q99584	**Protein S100-A13**	S100A13
0,029	-0,71	0,61	**61,13**	Q9BYE7	**Polycomb group RING finger protein 6**	PCGF6
0,023	-0,71	0,61	**61,11**	O43920	**NADH dehydrogenase [ubiquinone] iron-sulfur protein 5**	NDUFS5
0,043	-0,71	0,61	**61,10**	P46778	**60S ribosomal protein L21**	RPL21
0,018	-0,71	0,61	**61,01**	Q99570	**Phosphoinositide 3-kinase regulatory subunit 4**	PIK3R4
0,049	-0,71	0,61	**60,96**	Q00765	**Receptor expression-enhancing protein 5**	REEP5
0,049	-0,72	0,61	**60,89**	P53985	**Monocarboxylate transporter 1**	SLC16A1
0,021	-0,72	0,61	**60,74**	Q16762	**Thiosulfate sulfurtransferase**	TST
0,017	-0,72	0,61	**60,72**	Q9NPQ8	**Synembryn-A**	RIC8A
0,030	-0,72	0,61	**60,71**	Q5TGY3	**AT-hook DNA-binding motif-containing protein 1**	AHDC1
0,002	-0,72	0,61	**60,71**	Q92900	**Regulator of nonsense transcripts 1**	UPF1
0,004	-0,72	0,61	**60,71**	Q9P0K7	**Ankycorbin**	RAI14
0,034	-0,72	0,61	**60,71**	Q8NFF5	**FAD synthase;Molybdenum cofactor biosynthesis protein-like region;FAD synthase r**	FLAD1
0,000	-0,72	0,61	**60,71**	P23258;Q9NRH3	**Tubulin gamma-1 chain;Tubulin gamma-2 chain**	TUBG1;TUBG2
0,029	-0,72	0,61	**60,71**	Q8NFC6	**Biorientation of chromosomes in cell division protein 1-like 1**	BOD1L1
0,008	-0,72	0,61	**60,59**	Q96CW1	**AP-2 complex subunit mu**	AP2M1
0,004	-0,72	0,61	**60,54**	P43246	**DNA mismatch repair protein Msh2**	MSH2
0,010	-0,73	0,60	**60,46**	P00374	**Dihydrofolate reductase**	DHFR
0,018	-0,73	0,60	**60,43**	P53007	**Tricarboxylate transport protein, mitochondrial**	SLC25A1
0,041	-0,73	0,60	**60,43**	Q9NRN7	**L-aminoadipate-semialdehyde dehydrogenase-phosphopantetheinyl transferase**	AASDHPPT
0,018	-0,73	0,60	**60,34**	Q3ZCQ8	**Mitochondrial import inner membrane translocase subunit TIM50**	TIMM50
0,044	-0,73	0,60	**60,33**	Q7Z6K5	**Arpin**	ARPIN

0,002	-0,73	0,60	**60,29**	P27816	**Microtubule-associated protein 4**	MAP4
0,004	-0,73	0,60	**60,29**	Q9NRR4	**Ribonuclease 3**	DROSHA
0,012	-0,73	0,60	**60,29**	Q96DA6	**Mitochondrial import inner membrane translocase subunit TIM14**	DNAJC19
0,044	-0,73	0,60	**60,29**	Q8NCN4	**E3 ubiquitin-protein ligase RNF169**	RNF169
0,036	-0,73	0,60	**60,29**	Q96SI9	**Spermatid perinuclear RNA-binding protein**	STRBP
0,004	-0,73	0,60	**60,29**	P40424	**Pre-B-cell leukemia transcription factor 1**	PBX1
0,004	-0,73	0,60	**60,27**	Q9H0U4;Q92928;P6282	**Ras-related protein Rab-1B;Putative Ras-related protein Rab-1C;Ras-related protein**	AB1B;RAB1C;RAB1
0,013	-0,74	0,60	**60,06**	P49770	**Translation initiation factor eIF-2B subunit beta**	EIF2B2
0,003	-0,74	0,60	**60,04**	P60228	**Eukaryotic translation initiation factor 3 subunit E**	EIF3E
0,010	-0,74	0,60	**59,87**	Q14807	**Kinesin-like protein KIF22**	KIF22
0,022	-0,74	0,60	**59,87**	P18124	**60S ribosomal protein L7**	RPL7
0,013	-0,74	0,60	**59,87**	Q8N5C6	**S1 RNA-binding domain-containing protein 1**	SRBD1
0,017	-0,74	0,60	**59,87**	P61353	**60S ribosomal protein L27**	RPL27
0,046	-0,74	0,60	**59,87**	Q14676	**Mediator of DNA damage checkpoint protein 1**	MDC1
0,009	-0,74	0,60	**59,87**	P0CW22;P08708	**40S ribosomal protein S17-like;40S ribosomal protein S17**	RPS17L;RPS17
0,003	-0,75	0,60	**59,54**	Q8WVV9	**Heterogeneous nuclear ribonucleoprotein L-like**	HNRNPLL
0,049	-0,75	0,60	**59,51**	Q99471	**Prefoldin subunit 5**	PFDN5
0,048	-0,75	0,59	**59,46**	O14964	**Hepatocyte growth factor-regulated tyrosine kinase substrate**	HGS
0,033	-0,75	0,59	**59,46**	Q9BQ04	**RNA-binding protein 4B**	RBM4B
0,001	-0,75	0,59	**59,46**	P25398	**40S ribosomal protein S12**	RPS12
0,002	-0,75	0,59	**59,46**	P23396	**40S ribosomal protein S3**	RPS3
0,006	-0,75	0,59	**59,46**	P46776	**60S ribosomal protein L27a**	RPL27A
0,014	-0,75	0,59	**59,46**	Q92896	**Golgi apparatus protein 1**	GLG1
0,020	-0,75	0,59	**59,43**	P27448	**MAP/microtubule affinity-regulating kinase 3**	MARK3
0,034	-0,75	0,59	**59,43**	P51970	**NADH dehydrogenase [ubiquinone] 1 alpha subcomplex subunit 8**	NDUFA8
0,005	-0,75	0,59	**59,40**	P11717	**Cation-independent mannose-6-phosphate receptor**	IGF2R
0,017	-0,75	0,59	**59,31**	Q9UBQ5	**Eukaryotic translation initiation factor 3 subunit K**	EIF3K
0,001	-0,76	0,59	**59,19**	Q9Y5J9	**Mitochondrial import inner membrane translocase subunit Tim8 B**	TIMM8B
0,000	-0,76	0,59	**59,18**	Q16531	**DNA damage-binding protein 1**	DDB1
0,034	-0,76	0,59	**59,14**	Q9BTC8	**Metastasis-associated protein MTA3**	MTA3
0,009	-0,76	0,59	**59,13**	Q96N66	**Lysophospholipid acyltransferase 7**	MBOAT7
0,029	-0,76	0,59	**59,11**	Q96RU3	**Formin-binding protein 1**	FNBP1
0,047	-0,76	0,59	**59,05**	Q9UJV9	**Probable ATP-dependent RNA helicase DDX41**	DDX41

0,001	-0,76	0,59	**59,05**	P62249	**40S ribosomal protein S16**	RPS16
0,035	-0,76	0,59	**58,96**	Q9HB40	**Retinoid-inducible serine carboxypeptidase**	SCPEP1
0,006	-0,76	0,59	**58,94**	P08559	**Pyruvate dehydrogenase E1 component subunit alpha, somatic form, mitochondrial**	PDHA1
0,049	-0,77	0,59	**58,75**	Q8WXE0	**Caskin-2**	CASKIN2
0,010	-0,77	0,59	**58,64**	Q92922	**SWI/SNF complex subunit SMARCC1**	SMARCC1
0,002	-0,77	0,59	**58,64**	Q9UKJ3	**G patch domain-containing protein 8**	GPATCH8
0,001	-0,77	0,59	**58,64**	P46013	**Antigen KI-67**	MKI67
0,002	-0,77	0,59	**58,64**	Q14444	**Caprin-1**	CAPRIN1
0,009	-0,77	0,59	**58,64**	Q14258	**E3 ubiquitin/ISG15 ligase TRIM25**	TRIM25
0,034	-0,77	0,59	**58,64**	Q14134	**Tripartite motif-containing protein 29**	TRIM29
0,007	-0,77	0,59	**58,64**	P62487	**DNA-directed RNA polymerase II subunit RPB7**	POLR2G
0,012	-0,77	0,59	**58,64**	Q96EV2	**RNA-binding protein 33**	RBM33
0,022	-0,77	0,58	**58,50**	Q9C0C9	**E2/E3 hybrid ubiquitin-protein ligase UBE2O**	UBE2O
0,012	-0,78	0,58	**58,38**	Q9UIJ7	**GTP:AMP phosphotransferase AK3, mitochondrial**	AK3
0,041	-0,78	0,58	**58,36**	Q8IWB7	**WD repeat and FYVE domain-containing protein 1**	WDFY1
0,007	-0,78	0,58	**58,24**	P62701	**40S ribosomal protein S4, X isoform**	RPS4X
0,027	-0,78	0,58	**58,24**	Q9H6I2	**Transcription factor SOX-17**	SOX17
0,036	-0,78	0,58	**58,15**	Q9HCE1	**Putative helicase MOV-10**	MOV10
0,035	-0,78	0,58	**58,07**	Q9NZ01	**Very-long-chain enoyl-CoA reductase**	TECR
0,036	-0,79	0,58	**57,90**	P61313	**60S ribosomal protein L15**	RPL15
0,033	-0,79	0,58	**57,83**	P26641	**Elongation factor 1-gamma**	EEF1G
0,005	-0,79	0,58	**57,83**	Q9Y5Q8	**General transcription factor 3C polypeptide 5**	GTF3C5
0,049	-0,79	0,58	**57,83**	Q9H0E9	**Bromodomain-containing protein 8**	BRD8
0,010	-0,79	0,58	**57,83**	P50570	**Dynamin-2**	DNM2
0,016	-0,79	0,58	**57,83**	Q9NVC6	**Mediator of RNA polymerase II transcription subunit 17**	MED17
0,050	-0,79	0,58	**57,83**	Q8WXI9	**Transcriptional repressor p66-beta**	GATAD2B
0,002	-0,79	0,58	**57,79**	P07686	**Beta-hexosaminidase subunit beta;Beta-hexosaminidase subunit beta chain B;Beta-**	HEXB
0,008	-0,79	0,58	**57,72**	Q9NPL8	**Complex I assembly factor TIMMDC1, mitochondrial**	TIMMDC1
0,000	-0,80	0,58	**57,57**	Q9Y6E2	**Basic leucine zipper and W2 domain-containing protein 2**	BZW2
0,005	-0,80	0,58	**57,54**	P32456	**Interferon-induced guanylate-binding protein 2**	GBP2
0,016	-0,80	0,57	**57,43**	Q6I9Y2	**THO complex subunit 7 homolog**	THOC7
0,016	-0,80	0,57	**57,43**	P57088	**Transmembrane protein 33**	TMEM33
0,020	-0,80	0,57	**57,43**	P63313	**Thymosin beta-10**	TMSB10

0,008	-0,80	0,57	**57,35**	Q9Y3T9	**Nucleolar complex protein 2 homolog**	NOC2L
0,006	-0,80	0,57	**57,28**	Q9UK41	**Vacuolar protein sorting-associated protein 28 homolog**	VPS28
0,042	-0,81	0,57	**57,07**	Q6P1N0	**Coiled-coil and C2 domain-containing protein 1A**	CC2D1A
0,004	-0,81	0,57	**57,04**	Q9H0E3	**Histone deacetylase complex subunit SAP130**	SAP130
0,011	-0,81	0,57	**57,04**	Q9GZZ1	**N-alpha-acetyltransferase 50**	NAA50
0,003	-0,81	0,57	**57,04**	Q9BSC4	**Nucleolar protein 10**	NOL10
0,020	-0,81	0,57	**57,02**	O95602	**DNA-directed RNA polymerase I subunit RPA1**	POLR1A
0,048	-0,81	0,57	**56,94**	Q9BVL2	**Nucleoporin p58/p45**	NUPL1
0,001	-0,81	0,57	**56,92**	Q9HCU4	**Cadherin EGF LAG seven-pass G-type receptor 2**	CELSR2
0,019	-0,82	0,57	**56,64**	Q9BV38	**WD repeat-containing protein 18**	WDR18
0,001	-0,82	0,57	**56,64**	Q07157	**Tight junction protein ZO-1**	TJP1
0,013	-0,82	0,57	**56,64**	Q9NP50	**Protein FAM60A**	FAM60A
0,000	-0,82	0,57	**56,64**	Q8TAF3	**WD repeat-containing protein 48**	WDR48
0,002	-0,82	0,57	**56,64**	O15294	**UDP-N-acetylglucosamine--peptide N-acetylglucosaminyltransferase 110 kDa subun**	OGT
0,001	-0,82	0,57	**56,64**	Q7KZF4	**Staphylococcal nuclease domain-containing protein 1**	SND1
0,014	-0,82	0,57	**56,64**	P62244	**40S ribosomal protein S15a**	RPS15A
0,010	-0,82	0,57	**56,64**	Q15428	**Splicing factor 3A subunit 2**	SF3A2
0,018	-0,82	0,57	**56,64**	Q8ND04	**Protein SMG8**	SMG8
0,000	-0,82	0,57	**56,64**	O94832	**Unconventional myosin-Id**	MYO1D
0,021	-0,82	0,57	**56,64**	P20073	**Annexin A7**	ANXA7
0,035	-0,82	0,57	**56,63**	Q9UPT5	**Exocyst complex component 7**	EXOC7
0,043	-0,82	0,57	**56,60**	P31937	**3-hydroxyisobutyrate dehydrogenase, mitochondrial**	HIBADH
0,014	-0,82	0,57	**56,55**	Q5VYK3	**Proteasome-associated protein ECM29 homolog**	ECM29
0,000	-0,82	0,56	**56,46**	P0C1Z6	**TCF3 fusion partner**	TFPT
0,035	-0,83	0,56	**56,41**	Q7KZ85	**Transcription elongation factor SPT6**	SUPT6H
0,029	-0,83	0,56	**56,34**	O14639	**Actin-binding LIM protein 1**	ABLIM1
0,027	-0,83	0,56	**56,33**	P82933	**28S ribosomal protein S9, mitochondrial**	MRPS9
0,041	-0,83	0,56	**56,30**	Q70UQ0	**Inhibitor of nuclear factor kappa-B kinase-interacting protein**	IKBIP
0,033	-0,83	0,56	**56,29**	P20719	**Homeobox protein Hox-A5**	HOXA5
0,008	-0,83	0,56	**56,25**	O75351	**Vacuolar protein sorting-associated protein 4B**	VPS4B
0,045	-0,83	0,56	**56,25**	Q8WUA2	**Peptidyl-prolyl cis-trans isomerase-like 4**	PPIL4
0,007	-0,83	0,56	**56,25**	Q8IYI6	**Exocyst complex component 8**	EXOC8
0,021	-0,83	0,56	**56,22**	P84090	**Enhancer of rudimentary homolog**	ERH

0,004	-0,83	0,56	**56,12**	Q9UKX7	**Nuclear pore complex protein Nup50**	NUP50
0,030	-0,83	0,56	**56,12**	Q969Z3;Q5VT66	**Mitochondrial amidoxime reducing component 2;Mitochondrial amidoxime-reducin**	MARC2;MARC1
0,002	-0,84	0,56	**55,86**	P46108	**Adapter molecule crk**	CRK
0,042	-0,84	0,56	**55,86**	P78406	**mRNA export factor**	RAE1
0,034	-0,84	0,56	**55,86**	O75400	**Pre-mRNA-processing factor 40 homolog A**	PRPF40A
0,012	-0,84	0,56	**55,83**	O00159	**Unconventional myosin-Ic**	MYO1C
0,013	-0,84	0,56	**55,80**	Q13443	**Disintegrin and metalloproteinase domain-containing protein 9**	ADAM9
0,034	-0,85	0,56	**55,58**	P35269	**General transcription factor IIF subunit 1**	GTF2F1
0,006	-0,85	0,56	**55,51**	P17655	**Calpain-2 catalytic subunit**	CAPN2
0,026	-0,85	0,55	**55,48**	Q71F56	**Mediator of RNA polymerase II transcription subunit 13-like**	MED13L
0,034	-0,85	0,55	**55,48**	Q9ULL0	**Uncharacterized protein KIAA1210**	KIAA1210
0,030	-0,85	0,55	**55,36**	Q63ZY3	**KN motif and ankyrin repeat domain-containing protein 2**	KANK2
0,002	-0,85	0,55	**55,32**	P55268	**Laminin subunit beta-2**	LAMB2
0,031	-0,86	0,55	**55,28**	Q8ND56	**Protein LSM14 homolog A**	LSM14A
0,008	-0,86	0,55	**55,22**	P07384	**Calpain-1 catalytic subunit**	CAPN1
0,041	-0,86	0,55	**55,17**	P54725	**UV excision repair protein RAD23 homolog A**	RAD23A
0,013	-0,86	0,55	**55,14**	Q5T4S7	**E3 ubiquitin-protein ligase UBR4**	UBR4
0,006	-0,86	0,55	**55,10**	P39019	**40S ribosomal protein S19**	RPS19
0,010	-0,86	0,55	**55,10**	Q5JTV8	**Torsin-1A-interacting protein 1**	TOR1AIP1
0,000	-0,86	0,55	**55,10**	Q92614	**Unconventional myosin-XVIIIa**	MYO18A
0,010	-0,86	0,55	**55,10**	Q86W42	**THO complex subunit 6 homolog**	THOC6
0,019	-0,86	0,55	**55,00**	P31689	**DnaJ homolog subfamily A member 1**	DNAJA1
0,030	-0,87	0,55	**54,79**	Q16890	**Tumor protein D53**	TPD52L1
0,041	-0,87	0,55	**54,71**	Q9Y4K4	**Mitogen-activated protein kinase kinase kinase kinase 5**	MAP4K5
0,002	-0,87	0,55	**54,71**	Q96FV9	**THO complex subunit 1**	THOC1
0,016	-0,87	0,55	**54,71**	Q9BSJ2	**Gamma-tubulin complex component 2**	TUBGCP2
0,001	-0,87	0,55	**54,71**	A8MW92	**PHD finger protein 20-like protein 1**	PHF20L1
0,004	-0,87	0,55	**54,69**	Q13907	**Isopentenyl-diphosphate Delta-isomerase 1**	IDI1
0,002	-0,87	0,55	**54,62**	Q05048	**Cleavage stimulation factor subunit 1**	CSTF1
0,016	-0,88	0,54	**54,49**	Q5JTJ3	**Cytochrome c oxidase assembly factor 6 homolog**	COA6
0,028	-0,88	0,54	**54,41**	Q9Y3B4	**Splicing factor 3B subunit 6**	SF3B6
0,003	-0,88	0,54	**54,34**	P30154	**Serine/threonine-protein phosphatase 2A 65 kDa regulatory subunit A beta isoform**	PPP2R1B
0,004	-0,88	0,54	**54,34**	P46781	**40S ribosomal protein S9**	RPS9

0,002	-0,88	0,54	**54,34**	Q9H0H5	**Rac GTPase-activating protein 1**	RACGAP1
0,008	-0,88	0,54	**54,34**	Q9P206	**Uncharacterized protein KIAA1522**	KIAA1522
0,009	-0,88	0,54	**54,34**	Q96KQ7	**Histone-lysine N-methyltransferase EHMT2**	EHMT2
0,020	-0,88	0,54	**54,34**	Q92879	**CUGBP Elav-like family member 1**	CELF1
0,038	-0,88	0,54	**54,25**	Q96EP5	**DAZ-associated protein 1**	DAZAP1
0,007	-0,88	0,54	**54,22**	P21964	**Catechol O-methyltransferase**	COMT
0,037	-0,88	0,54	**54,19**	Q96J02	**E3 ubiquitin-protein ligase Itchy homolog**	ITCH
0,043	-0,89	0,54	**54,14**	Q96EB1	**Elongator complex protein 4**	ELP4
0,023	-0,89	0,54	**54,05**	Q9Y5Z9	**UbiA prenyltransferase domain-containing protein 1**	UBIAD1
0,015	-0,89	0,54	**54,03**	Q9UK76	**Hematological and neurological expressed 1 protein;Hematological and neurologica**	HN1
0,038	-0,89	0,54	**53,96**	Q92793	**CREB-binding protein**	CREBBP
0,050	-0,89	0,54	**53,96**	P46100	**Transcriptional regulator ATRX**	ATRX
0,021	-0,89	0,54	**53,90**	Q9HB71	**Calcyclin-binding protein**	CACYBP
0,007	-0,89	0,54	**53,84**	P82675	**28S ribosomal protein S5, mitochondrial**	MRPS5
0,019	-0,90	0,54	**53,59**	Q9NRG0	**Chromatin accessibility complex protein 1**	CHRAC1
0,004	-0,90	0,54	**53,59**	P11388	**DNA topoisomerase 2-alpha**	TOP2A
0,002	-0,90	0,54	**53,59**	P12004	**Proliferating cell nuclear antigen**	PCNA
0,032	-0,90	0,54	**53,59**	P30085	**UMP-CMP kinase**	CMPK1
0,023	-0,90	0,54	**53,57**	Q96ER3	**Protein SAAL1**	SAAL1
0,042	-0,90	0,54	**53,57**	P32004	**Neural cell adhesion molecule L1**	L1CAM
0,003	-0,90	0,54	**53,50**	O94964	**Protein SOGA1;N-terminal form;C-terminal 80 kDa form**	SOGA1
0,027	-0,91	0,53	**53,37**	P46063	**ATP-dependent DNA helicase Q1**	RECQL
0,003	-0,91	0,53	**53,37**	Q5T440	**Putative transferase CAF17, mitochondrial**	IBA57
0,015	-0,91	0,53	**53,32**	Q9HD15	**Steroid receptor RNA activator 1**	SRA1
0,011	-0,91	0,53	**53,22**	P15880	**40S ribosomal protein S2**	RPS2
0,002	-0,91	0,53	**53,22**	P17096	**High mobility group protein HMG-I/HMG-Y**	HMGA1
0,036	-0,91	0,53	**53,22**	P62834	**Ras-related protein Rap-1A**	RAP1A
0,003	-0,91	0,53	**53,16**	Q13438	**Protein OS-9**	OS9
0,031	-0,92	0,53	**53,00**	P50416	**Carnitine O-palmitoyltransferase 1, liver isoform**	CPT1A
0,000	-0,92	0,53	**52,97**	O15230	**Laminin subunit alpha-5**	LAMA5
0,003	-0,92	0,53	**52,90**	O75534	**Cold shock domain-containing protein E1**	CSDE1
0,000	-0,92	0,53	**52,85**	P62633	**Cellular nucleic acid-binding protein**	CNBP
0,050	-0,92	0,53	**52,77**	Q15003	**Condensin complex subunit 2**	NCAPH

0,007	-0,93	0,53	**52,52**	Q9BSJ8	**Extended synaptotagmin-1**	ESYT1
0,036	-0,93	0,52	**52,49**	P55081	**Microfibrillar-associated protein 1**	MFAP1
0,007	-0,93	0,52	**52,49**	P09012	**U1 small nuclear ribonucleoprotein A**	SNRPA
0,007	-0,93	0,52	**52,49**	Q9NV88	**Integrator complex subunit 9**	INTS9
0,035	-0,93	0,52	**52,38**	P31153	**S-adenosylmethionine synthase isoform type-2**	MAT2A
0,004	-0,94	0,52	**52,24**	Q9Y2U8	**Inner nuclear membrane protein Man1**	LEMD3
0,005	-0,94	0,52	**52,18**	Q9NRR5	**Ubiquilin-4**	UBQLN4
0,022	-0,94	0,52	**52,16**	O95163	**Elongator complex protein 1**	IKBKAP
0,034	-0,94	0,52	**52,12**	Q6PGP7	**Tetratricopeptide repeat protein 37**	TTC37
0,018	-0,94	0,52	**52,12**	Q9BW27	**Nuclear pore complex protein Nup85**	NUP85
0,047	-0,94	0,52	**52,12**	O43660	**Pleiotropic regulator 1**	PLRG1
0,012	-0,94	0,52	**52,12**	Q9BQ61	**Uncharacterized protein C19orf43**	C19orf43
0,015	-0,94	0,52	**52,12**	P14324	**Farnesyl pyrophosphate synthase**	FDPS
0,009	-0,94	0,52	**52,03**	Q969U7	**Proteasome assembly chaperone 2**	PSMG2
0,015	-0,94	0,52	**51,94**	Q9H936	**Mitochondrial glutamate carrier 1**	SLC25A22
0,039	-0,95	0,52	**51,92**	P61962	**DDB1- and CUL4-associated factor 7**	DCAF7
0,010	-0,95	0,52	**51,86**	Q6IN85	**Serine/threonine-protein phosphatase 4 regulatory subunit 3A**	SMEK1
0,019	-0,95	0,52	**51,77**	P61619;Q9H9S3	**Protein transport protein Sec61 subunit alpha isoform 1;Protein transport protein S**	SEC61A1;SEC61A2
0,007	-0,95	0,52	**51,76**	Q96J01	**THO complex subunit 3**	THOC3
0,003	-0,95	0,52	**51,76**	Q9UGU0	**Transcription factor 20**	TCF20
0,005	-0,96	0,52	**51,53**	Q9Y282	**Endoplasmic reticulum-Golgi intermediate compartment protein 3**	ERGIC3
0,002	-0,96	0,52	**51,50**	Q6NXG1	**Epithelial splicing regulatory protein 1**	ESRP1
0,031	-0,96	0,51	**51,50**	Q8WVC0	**RNA polymerase-associated protein LEO1**	LEO1
0,011	-0,96	0,51	**51,41**	Q9H2M9	**Rab3 GTPase-activating protein non-catalytic subunit**	RAB3GAP2
0,005	-0,96	0,51	**51,41**	Q9UBL3	**Set1/Ash2 histone methyltransferase complex subunit ASH2**	ASH2L
0,022	-0,96	0,51	**51,41**	Q8NHQ9	**ATP-dependent RNA helicase DDX55**	DDX55
0,001	-0,96	0,51	**51,41**	Q8TEA8	**D-tyrosyl-tRNA(Tyr) deacylase 1**	DTD1
0,003	-0,96	0,51	**51,41**	Q9C0F1	**Centrosomal protein of 44 kDa**	CEP44
0,001	-0,96	0,51	**51,41**	P62495	**Eukaryotic peptide chain release factor subunit 1**	ETF1
0,002	-0,96	0,51	**51,41**	Q9NVM9	**Protein asunder homolog**	ASUN
0,000	-0,96	0,51	**51,41**	Q8NC51	**Plasminogen activator inhibitor 1 RNA-binding protein**	SERBP1
0,038	-0,96	0,51	**51,40**	P42224	**Signal transducer and activator of transcription 1-alpha/beta**	STAT1
0,036	-0,96	0,51	**51,37**	Q96K76	**Ubiquitin carboxyl-terminal hydrolase 47**	USP47

0,007	-0,97	0,51	**51,17**	P28288	**ATP-binding cassette sub-family D member 3**	ABCD3
0,003	-0,97	0,51	**51,13**	Q9BZX2	**Uridine-cytidine kinase 2**	UCK2
0,027	-0,97	0,51	**51,11**	Q9UNS2	**COP9 signalosome complex subunit 3**	COPS3
0,011	-0,97	0,51	**51,05**	Q96JM2	**Zinc finger protein 462**	ZNF462
0,030	-0,97	0,51	**51,05**	P21399	**Cytoplasmic aconitate hydratase**	ACO1
0,001	-0,97	0,51	**51,05**	P40926	**Malate dehydrogenase, mitochondrial**	MDH2
0,042	-0,97	0,51	**51,05**	Q00325	**Phosphate carrier protein, mitochondrial**	SLC25A3
0,005	-0,97	0,51	**51,05**	P62280	**40S ribosomal protein S11**	RPS11
0,020	-0,97	0,51	**51,03**	O43813	**LanC-like protein 1**	LANCL1
0,005	-0,97	0,51	**51,01**	P48730	**Casein kinase I isoform delta**	CSNK1D
0,040	-0,97	0,51	**50,97**	Q8IWS0	**PHD finger protein 6**	PHF6
0,026	-0,98	0,51	**50,83**	P05556	**Integrin beta-1**	ITGB1
0,019	-0,98	0,51	**50,82**	Q14008	**Cytoskeleton-associated protein 5**	CKAP5
0,043	-0,98	0,51	**50,70**	Q8TDD1	**ATP-dependent RNA helicase DDX54**	DDX54
0,024	-0,98	0,51	**50,70**	P63092;Q5JWF2	**Guanine nucleotide-binding protein G(s) subunit alpha isoforms short;Guanine nucl**	GNAS
0,007	-0,98	0,51	**50,56**	O15162	**Phospholipid scramblase 1**	PLSCR1
0,013	-0,98	0,51	**50,54**	Q13206	**Probable ATP-dependent RNA helicase DDX10**	DDX10
0,020	-0,99	0,51	**50,52**	Q27J81	**Inverted formin-2**	INF2
0,006	-0,99	0,51	**50,51**	Q14318	**Peptidyl-prolyl cis-trans isomerase FKBP8**	FKBP8
0,001	-0,99	0,50	**50,35**	Q9BQ39	**ATP-dependent RNA helicase DDX50**	DDX50
0,048	-0,99	0,50	**50,35**	Q00535	**Cyclin-dependent-like kinase 5**	CDK5
0,001	-0,99	0,50	**50,35**	Q13257	**Mitotic spindle assembly checkpoint protein MAD2A**	MAD2L1
0,004	-0,99	0,50	**50,35**	Q13330	**Metastasis-associated protein MTA1**	MTA1
0,001	-0,99	0,50	**50,33**	O15357	**Phosphatidylinositol 3,4,5-trisphosphate 5-phosphatase 2**	INPPL1
0,010	-0,99	0,50	**50,25**	Q7Z6Z7	**E3 ubiquitin-protein ligase HUWE1**	HUWE1
0,007	-0,99	0,50	**50,20**	P33121	**Long-chain-fatty-acid--CoA ligase 1**	ACSL1
0,026	-1,00	0,50	**50,00**	Q96GM8	**Target of EGR1 protein 1**	TOE1
0,002	-1,00	0,50	**50,00**	Q9H444	**Charged multivesicular body protein 4b**	CHMP4B
0,000	-1,00	0,50	**50,00**	P26599	**Polypyrimidine tract-binding protein 1**	PTBP1
0,004	-1,00	0,50	**50,00**	Q9H910	**Hematological and neurological expressed 1-like protein**	HN1L
0,023	-1,01	0,50	**49,80**	Q9NUY8	**TBC1 domain family member 23**	TBC1D23
0,043	-1,01	0,50	**49,73**	Q9UBI6	**Guanine nucleotide-binding protein G(I)/G(S)/G(O) subunit gamma-12**	GNG12
0,014	-1,01	0,50	**49,71**	Q9Y6Q5	**AP-1 complex subunit mu-2**	AP1M2

0,009	-1,01	0,50	**49,66**	Q9HBL0	**Tensin-1**	TNS1
0,010	-1,01	0,50	**49,65**	Q99986	**Serine/threonine-protein kinase VRK1**	VRK1
0,028	-1,01	0,50	**49,65**	O43639	**Cytoplasmic protein NCK2**	NCK2
0,003	-1,01	0,50	**49,65**	O96028	**Histone-lysine N-methyltransferase NSD2**	WHSC1
0,041	-1,01	0,50	**49,65**	Q14191	**Werner syndrome ATP-dependent helicase**	WRN
0,002	-1,01	0,50	**49,65**	Q8TEX9	**Importin-4**	IPO4
0,003	-1,01	0,50	**49,63**	Q14108	**Lysosome membrane protein 2**	SCARB2
0,007	-1,01	0,50	**49,62**	Q9Y520	**Protein PRRC2C**	PRRC2C
0,025	-1,02	0,49	**49,44**	Q9H6W3	**Bifunctional lysine-specific demethylase and histidyl-hydroxylase NO66**	NO66
0,008	-1,02	0,49	**49,43**	P04844	**Dolichyl-diphosphooligosaccharide--protein glycosyltransferase subunit 2**	RPN2
0,003	-1,02	0,49	**49,31**	Q8NFW8	**N-acylneuraminate cytidylyltransferase**	CMAS
0,002	-1,02	0,49	**49,31**	Q8NDF8	**Non-canonical poly(A) RNA polymerase PAPD5**	PAPD5
0,006	-1,02	0,49	**49,31**	Q13283	**Ras GTPase-activating protein-binding protein 1**	G3BP1
0,003	-1,02	0,49	**49,31**	Q96AG4	**Leucine-rich repeat-containing protein 59**	LRRC59
0,026	-1,02	0,49	**49,31**	O15514	**DNA-directed RNA polymerase II subunit RPB4**	POLR2D
0,030	-1,02	0,49	**49,31**	O00471	**Exocyst complex component 5**	EXOC5
0,011	-1,02	0,49	**49,31**	Q96M96	**FYVE, RhoGEF and PH domain-containing protein 4**	FGD4
0,014	-1,02	0,49	**49,31**	Q9H8S9	**MOB kinase activator 1A**	MOB1A
0,003	-1,02	0,49	**49,31**	Q13506	**NGFI-A-binding protein 1**	NAB1
0,002	-1,02	0,49	**49,28**	Q99828	**Calcium and integrin-binding protein 1**	CIB1
0,035	-1,02	0,49	**49,26**	Q96HY6	**DDRGK domain-containing protein 1**	DDRGK1
0,018	-1,02	0,49	**49,22**	9BWW4;P81877;Q9BW(	**Single-stranded DNA-binding protein 3;Single-stranded DNA-binding protein 2;Singl**	SSBP3;SSBP2;SSBP4
0,010	-1,02	0,49	**49,16**	Q9NXW2	**DnaJ homolog subfamily B member 12**	DNAJB12
0,016	-1,03	0,49	**49,07**	Q9H5V8	**CUB domain-containing protein 1**	CDCP1
0,015	-1,03	0,49	**49,05**	Q92563	**Testican-2**	SPOCK2
0,020	-1,03	0,49	**48,98**	Q9H3U1	**Protein unc-45 homolog A**	UNC45A
0,039	-1,03	0,49	**48,97**	O75909	**Cyclin-K**	CCNK
0,019	-1,03	0,49	**48,97**	P12235	**ADP/ATP translocase 1**	SLC25A4
0,025	-1,03	0,49	**48,97**	Q7Z4L5	**Tetratricopeptide repeat protein 21B**	TTC21B
0,001	-1,03	0,49	**48,97**	Q7Z5J4	**Retinoic acid-induced protein 1**	RAI1
0,010	-1,03	0,49	**48,97**	Q7Z2T5	**TRMT1-like protein**	TRMT1L
0,008	-1,03	0,49	**48,93**	Q9UFC0	**Leucine-rich repeat and WD repeat-containing protein 1**	LRWD1
0,030	-1,04	0,49	**48,65**	Q7L2E3	**Putative ATP-dependent RNA helicase DHX30**	DHX30

0,018	-1,04	0,49	**48,65**	Q460N5	**Poly [ADP-ribose] polymerase 14**	PARP14
0,012	-1,04	0,49	**48,63**	Q9UJX3	**Anaphase-promoting complex subunit 7**	ANAPC7
0,009	-1,04	0,49	**48,63**	Q5JVF3	**PCI domain-containing protein 2**	PCID2
0,001	-1,04	0,49	**48,63**	P23588	**Eukaryotic translation initiation factor 4B**	EIF4B
0,045	-1,04	0,49	**48,57**	Q15477	**Helicase SKI2W**	SKIV2L
0,010	-1,04	0,49	**48,52**	Q9UIQ6	**Leucyl-cystinyl aminopeptidase;Leucyl-cystinyl aminopeptidase, pregnancy serum fo**	LNPEP
0,000	-1,05	0,48	**48,39**	O60232	**Sjoegren syndrome/scleroderma autoantigen 1**	SSSCA1
0,036	-1,05	0,48	**48,30**	Q14697	**Neutral alpha-glucosidase AB**	GANAB
0,011	-1,05	0,48	**48,30**	Q9HCK8	**Chromodomain-helicase-DNA-binding protein 8**	CHD8
0,013	-1,05	0,48	**48,21**	P63165	**Small ubiquitin-related modifier 1**	SUMO1
0,010	-1,05	0,48	**48,20**	P26358	**DNA (cytosine-5)-methyltransferase 1**	DNMT1
0,007	-1,06	0,48	**48,11**	Q8NBJ7	**Sulfatase-modifying factor 2**	SUMF2
0,013	-1,06	0,48	**48,07**	P06756	**Integrin alpha-V;Integrin alpha-V heavy chain;Integrin alpha-V light chain**	ITGAV
0,014	-1,06	0,48	**48,04**	P40222	**Alpha-taxilin**	TXLNA
0,047	-1,06	0,48	**47,87**	Q6XQN6	**Nicotinate phosphoribosyltransferase**	NAPRT
0,047	-1,07	0,48	**47,75**	P46976	**Glycogenin-1**	GYG1
0,016	-1,07	0,48	**47,71**	O75781	**Paralemmin-1**	PALM
0,006	-1,07	0,48	**47,64**	O43847	**Nardilysin**	NRD1
0,013	-1,07	0,48	**47,63**	Q75QN2	**Integrator complex subunit 8**	INTS8
0,022	-1,07	0,48	**47,63**	O43929	**Origin recognition complex subunit 4**	ORC4
0,018	-1,07	0,48	**47,63**	O95239	**Chromosome-associated kinesin KIF4A**	KIF4A
0,018	-1,07	0,48	**47,63**	Q9Y2T2	**AP-3 complex subunit mu-1**	AP3M1
0,001	-1,07	0,48	**47,63**	Q14966	**Zinc finger protein 638**	ZNF638
0,010	-1,07	0,48	**47,63**	O15013	**Rho guanine nucleotide exchange factor 10**	ARHGEF10
0,025	-1,07	0,48	**47,62**	O15258	**Protein RER1**	RER1
0,007	-1,07	0,48	**47,52**	O60610	**Protein diaphanous homolog 1**	DIAPH1
0,007	-1,07	0,48	**47,52**	O75175	**CCR4-NOT transcription complex subunit 3**	CNOT3
0,038	-1,07	0,48	**47,50**	O43676	**NADH dehydrogenase [ubiquinone] 1 beta subcomplex subunit 3**	NDUFB3
0,000	-1,08	0,47	**47,36**	Q8IZH2	**5-3 exoribonuclease 1**	XRN1
0,026	-1,08	0,47	**47,31**	Q13153	**Serine/threonine-protein kinase PAK 1**	PAK1
0,001	-1,08	0,47	**47,30**	P33176	**Kinesin-1 heavy chain**	KIF5B
0,005	-1,08	0,47	**47,30**	P62277	**40S ribosomal protein S13**	RPS13
0,037	-1,08	0,47	**47,30**	Q9H270	**Vacuolar protein sorting-associated protein 11 homolog**	VPS11

0,010	-1,09	0,47	**47,10**	Q9UKU6	**Thyrotropin-releasing hormone-degrading ectoenzyme**	TRHDE
0,008	-1,09	0,47	**47,06**	Q02818	**Nucleobindin-1**	NUCB1
0,022	-1,09	0,47	**47,03**	Q96T23	**Remodeling and spacing factor 1**	RSF1
0,039	-1,09	0,47	**46,98**	Q99719	**Septin-5**	38596,000
0,002	-1,09	0,47	**46,98**	Q96DE0	**U8 snoRNA-decapping enzyme**	NUDT16
0,001	-1,09	0,47	**46,98**	Q96BZ8	**Leukocyte receptor cluster member 1**	LENG1
0,011	-1,10	0,47	**46,77**	Q9H0D6	**5-3 exoribonuclease 2**	XRN2
0,008	-1,10	0,47	**46,66**	Q86Y56	**HEAT repeat-containing protein 2**	HEATR2
0,008	-1,10	0,47	**46,65**	O00429	**Dynamin-1-like protein**	DNM1L
0,032	-1,10	0,47	**46,65**	P13693	**Translationally-controlled tumor protein**	TPT1
0,000	-1,10	0,47	**46,65**	Q6UXN9	**WD repeat-containing protein 82**	WDR82
0,012	-1,10	0,47	**46,60**	P04818	**Thymidylate synthase**	TYMS
0,015	-1,11	0,46	**46,40**	P62714	**Serine/threonine-protein phosphatase 2A catalytic subunit beta isoform**	PPP2CB
0,023	-1,11	0,46	**46,37**	Q9UKF6	**Cleavage and polyadenylation specificity factor subunit 3**	CPSF3
0,041	-1,11	0,46	**46,37**	P11274	**Breakpoint cluster region protein**	BCR
0,005	-1,11	0,46	**46,33**	Q07020	**60S ribosomal protein L18**	RPL18
0,015	-1,11	0,46	**46,33**	P09936	**Ubiquitin carboxyl-terminal hydrolase isozyme L1**	UCHL1
0,007	-1,11	0,46	**46,33**	P41227	**N-alpha-acetyltransferase 10**	NAA10
0,004	-1,11	0,46	**46,33**	Q8NEM2	**SHC SH2 domain-binding protein 1**	SHCBP1
0,009	-1,12	0,46	**46,01**	Q16698	**2,4-dienoyl-CoA reductase, mitochondrial**	DECR1
0,029	-1,12	0,46	**46,01**	Q6ZYL4	**General transcription factor IIH subunit 5**	GTF2H5
0,007	-1,12	0,46	**46,01**	Q9P2I0	**Cleavage and polyadenylation specificity factor subunit 2**	CPSF2
0,022	-1,12	0,46	**46,01**	P62266	**40S ribosomal protein S23**	RPS23
0,008	-1,12	0,46	**46,01**	Q9UHF7	**Zinc finger transcription factor Trps1**	TRPS1
0,025	-1,12	0,46	**46,01**	P18074	**TFIIH basal transcription factor complex helicase XPD subunit**	ERCC2
0,000	-1,12	0,46	**46,01**	Q9BWU1;P49336	**Cyclin-dependent kinase 19;Cyclin-dependent kinase 8**	CDK19;CDK8
0,002	-1,12	0,46	**46,01**	Q9GZR2	**RNA exonuclease 4**	REXO4
0,017	-1,12	0,46	**45,99**	Q9NYJ1	**Cytochrome c oxidase assembly factor 4 homolog, mitochondrial**	COA4
0,006	-1,13	0,46	**45,73**	O60563	**Cyclin-T1**	CCNT1
0,049	-1,13	0,46	**45,71**	Q7L266	**Isoaspartyl peptidase/L-asparaginase;Isoaspartyl peptidase/L-asparaginase alpha ch**	ASRGL1
0,031	-1,13	0,46	**45,69**	Q8IWZ3	**Ankyrin repeat and KH domain-containing protein 1**	ANKHD1
0,023	-1,14	0,45	**45,38**	Q8NB78	**Lysine-specific histone demethylase 1B**	KDM1B
0,027	-1,14	0,45	**45,38**	Q69YN2	**CWF19-like protein 1**	CWF19L1

0,001	-1,14	0,45	**45,38**	Q9UHV7	**Mediator of RNA polymerase II transcription subunit 13**	MED13
0,003	-1,15	0,45	**45,16**	Q93008	**Probable ubiquitin carboxyl-terminal hydrolase FAF-X**	USP9X
0,036	-1,15	0,45	**45,06**	P49257	**Protein ERGIC-53**	LMAN1
0,012	-1,15	0,45	**45,06**	O96000	**NADH dehydrogenase [ubiquinone] 1 beta subcomplex subunit 10**	NDUFB10
0,021	-1,15	0,45	**45,06**	Q96G46	**tRNA-dihydrouridine(47) synthase [NAD(P)(+)]-like**	DUS3L
0,003	-1,15	0,45	**45,00**	Q7Z5L9	**Interferon regulatory factor 2-binding protein 2**	IRF2BP2
0,014	-1,15	0,45	**44,93**	Q8N3C0	**Activating signal cointegrator 1 complex subunit 3**	ASCC3
0,005	-1,15	0,45	**44,93**	O96013	**Serine/threonine-protein kinase PAK 4**	PAK4
0,010	-1,16	0,45	**44,80**	Q00534	**Cyclin-dependent kinase 6**	CDK6
0,016	-1,16	0,45	**44,75**	Q9H8H2	**Probable ATP-dependent RNA helicase DDX31**	DDX31
0,003	-1,16	0,45	**44,75**	Q8IZP0	**Abl interactor 1**	ABI1
0,045	-1,16	0,45	**44,75**	O43776	**Asparagine--tRNA ligase, cytoplasmic**	NARS
0,001	-1,17	0,44	**44,44**	Q13127	**RE1-silencing transcription factor**	REST
0,045	-1,17	0,44	**44,38**	O14976	**Cyclin-G-associated kinase**	GAK
0,002	-1,18	0,44	**44,23**	P55060	**Exportin-2**	CSE1L
0,046	-1,18	0,44	**44,14**	Q9UJX2	**Cell division cycle protein 23 homolog**	CDC23
0,050	-1,18	0,44	**44,14**	Q9NVH2	**Integrator complex subunit 7**	INTS7
0,004	-1,18	0,44	**44,00**	Q16795	**NADH dehydrogenase [ubiquinone] 1 alpha subcomplex subunit 9, mitochondrial**	NDUFA9
0,011	-1,19	0,44	**43,95**	Q96HW7	**Integrator complex subunit 4**	INTS4
0,043	-1,19	0,44	**43,85**	P55809	**Succinyl-CoA:3-ketoacid coenzyme A transferase 1, mitochondrial**	OXCT1
0,000	-1,19	0,44	**43,83**	Q9BRJ7	**Protein syndesmos**	NUDT16L1
0,031	-1,19	0,44	**43,83**	O76003	**Glutaredoxin-3**	GLRX3
0,018	-1,19	0,44	**43,83**	Q9H0P0	**Cytosolic 5-nucleotidase 3A**	NT5C3A
0,010	-1,19	0,44	**43,83**	P30876	**DNA-directed RNA polymerase II subunit RPB2**	POLR2B
0,038	-1,19	0,44	**43,80**	O95782	**AP-2 complex subunit alpha-1**	AP2A1
0,002	-1,20	0,44	**43,53**	Q96RN5	**Mediator of RNA polymerase II transcription subunit 15**	MED15
0,012	-1,20	0,44	**43,53**	Q674X7	**Kazrin**	KAZN
0,018	-1,20	0,43	**43,45**	O43592	**Exportin-T**	XPOT
0,005	-1,20	0,43	**43,44**	Q8WWY3	**U4/U6 small nuclear ribonucleoprotein Prp31**	PRPF31
0,049	-1,20	0,43	**43,43**	Q12824	**SWI/SNF-related matrix-associated actin-dependent regulator of chromatin subfami**	SMARCB1
0,026	-1,20	0,43	**43,41**	Q9P273	**Teneurin-3**	TENM3
0,025	-1,21	0,43	**43,23**	Q7Z4G1	**COMM domain-containing protein 6**	COMMD6
0,002	-1,21	0,43	**43,23**	Q15398	**Disks large-associated protein 5**	DLGAP5

0,021	-1,21	0,43	**43,23**	P51531	**Probable global transcription activator SNF2L2**	SMARCA2
0,023	-1,21	0,43	**43,09**	Q13501	**Sequestosome-1**	SQSTM1
0,014	-1,21	0,43	**43,08**	Q7L576	**Cytoplasmic FMR1-interacting protein 1**	CYFIP1
0,007	-1,22	0,43	**42,93**	P30260	**Cell division cycle protein 27 homolog**	CDC27
0,006	-1,22	0,43	**42,93**	O60890	**Oligophrenin-1**	OPHN1
0,005	-1,22	0,43	**42,83**	Q86X29	**Lipolysis-stimulated lipoprotein receptor**	LSR
0,003	-1,23	0,43	**42,63**	Q96QC0	**Serine/threonine-protein phosphatase 1 regulatory subunit 10**	PPP1R10
0,014	-1,23	0,43	**42,63**	Q8N201	**Integrator complex subunit 1**	INTS1
0,008	-1,24	0,42	**42,42**	O95155	**Ubiquitin conjugation factor E4 B**	UBE4B
0,049	-1,24	0,42	**42,34**	P42167	**Lamina-associated polypeptide 2, isoforms beta/gamma;Thymopoietin;Thymopenti**	TMPO
0,048	-1,24	0,42	**42,34**	Q9UKS7	**Zinc finger protein Helios**	IKZF2
0,035	-1,24	0,42	**42,34**	O00443	**Phosphatidylinositol 4-phosphate 3-kinase C2 domain-containing subunit alpha**	PIK3C2A
0,008	-1,24	0,42	**42,34**	Q53F19	**Uncharacterized protein C17orf85**	C17orf85
0,013	-1,24	0,42	**42,34**	Q9Y262	**Eukaryotic translation initiation factor 3 subunit L**	EIF3L
0,018	-1,24	0,42	**42,23**	Q8IY81	**pre-rRNA processing protein FTSJ3**	FTSJ3
0,018	-1,25	0,42	**42,14**	Q8IV08	**Phospholipase D3**	PLD3
0,026	-1,25	0,42	**42,07**	Q92538	**Golgi-specific brefeldin A-resistance guanine nucleotide exchange factor 1**	GBF1
0,013	-1,25	0,42	**42,04**	O95861	**3(2),5-bisphosphate nucleotidase 1**	BPNT1
0,017	-1,25	0,42	**42,04**	Q96IZ7	**Serine/Arginine-related protein 53**	RSRC1
0,000	-1,25	0,42	**42,04**	Q14320	**Protein FAM50A**	FAM50A
0,010	-1,26	0,42	**41,77**	P62330	**ADP-ribosylation factor 6**	ARF6
0,043	-1,26	0,42	**41,75**	Q7Z7C8	**Transcription initiation factor TFIID subunit 8**	TAF8
0,003	-1,26	0,42	**41,75**	O60216	**Double-strand-break repair protein rad21 homolog**	RAD21
0,026	-1,26	0,42	**41,72**	P10644	**cAMP-dependent protein kinase type I-alpha regulatory subunit;cAMP-dependent p**	PRKAR1A
0,006	-1,27	0,41	**41,47**	Q00688	**Peptidyl-prolyl cis-trans isomerase FKBP3**	FKBP3
0,043	-1,27	0,41	**41,47**	Q9BVG4	**Protein PBDC1**	PBDC1
0,002	-1,27	0,41	**41,47**	Q01970	**1-phosphatidylinositol 4,5-bisphosphate phosphodiesterase beta-3**	PLCB3
0,006	-1,27	0,41	**41,47**	Q96II8	**Leucine-rich repeat and calponin homology domain-containing protein 3**	LRCH3
0,000	-1,27	0,41	**41,46**	Q96ER9	**Coiled-coil domain-containing protein 51**	CCDC51
0,006	-1,27	0,41	**41,42**	P00533	**Epidermal growth factor receptor**	EGFR
0,016	-1,28	0,41	**41,23**	P04637	**Cellular tumor antigen p53**	TP53
0,049	-1,28	0,41	**41,18**	Q9UHR4	**Brain-specific angiogenesis inhibitor 1-associated protein 2-like protein 1**	BAIAP2L1
0,026	-1,28	0,41	**41,18**	Q99816	**Tumor susceptibility gene 101 protein**	TSG101

0,005	-1,28	0,41	**41,18**	Q86YP4	**Transcriptional repressor p66-alpha**	GATAD2A
0,026	-1,28	0,41	**41,18**	P06493	**Cyclin-dependent kinase 1**	CDK1
0,046	-1,28	0,41	**41,17**	P23634	**Plasma membrane calcium-transporting ATPase 4**	ATP2B4
0,000	-1,28	0,41	**41,09**	O00468	**Agrin;Agrin N-terminal 110 kDa subunit;Agrin C-terminal 110 kDa subunit;Agrin C-te**	AGRN
0,008	-1,28	0,41	**41,07**	Q9BSV6	**tRNA-splicing endonuclease subunit Sen34**	TSEN34
0,015	-1,29	0,41	**40,90**	P12236	**ADP/ATP translocase 3;ADP/ATP translocase 3, N-terminally processed**	SLC25A6
0,001	-1,29	0,41	**40,90**	Q6PJT7	**Zinc finger CCCH domain-containing protein 14**	ZC3H14
0,032	-1,29	0,41	**40,90**	Q9UPN6	**Protein SCAF8**	SCAF8
0,009	-1,29	0,41	**40,90**	A1L020	**RNA-binding protein MEX3A**	MEX3A
0,012	-1,29	0,41	**40,90**	Q9Y5B6	**PAX3- and PAX7-binding protein 1**	PAXBP1
0,012	-1,29	0,41	**40,90**	Q8IX18	**Probable ATP-dependent RNA helicase DHX40**	DHX40
0,007	-1,29	0,41	**40,90**	Q9H8V3	**Protein ECT2**	ECT2
0,011	-1,29	0,41	**40,81**	Q8NFH4	**Nucleoporin Nup37**	NUP37
0,001	-1,30	0,41	**40,61**	P62861	**40S ribosomal protein S30**	FAU
0,006	-1,30	0,41	**40,61**	Q9NZQ3	**NCK-interacting protein with SH3 domain**	NCKIPSD
0,034	-1,30	0,41	**40,55**	O95436	**Sodium-dependent phosphate transport protein 2B**	SLC34A2
0,005	-1,31	0,40	**40,42**	Q99567	**Nuclear pore complex protein Nup88**	NUP88
0,013	-1,31	0,40	**40,33**	Q9Y421	**Protein FAM32A**	FAM32A
0,000	-1,31	0,40	**40,33**	P50238	**Cysteine-rich protein 1**	CRIP1
0,004	-1,31	0,40	**40,33**	Q6IN84	**rRNA methyltransferase 1, mitochondrial**	MRM1
0,001	-1,31	0,40	**40,33**	P30533	**Alpha-2-macroglobulin receptor-associated protein**	LRPAP1
0,003	-1,31	0,40	**40,29**	Q9BW91	**ADP-ribose pyrophosphatase, mitochondrial**	NUDT9
0,019	-1,32	0,40	**40,05**	Q8WXA9	**Splicing regulatory glutamine/lysine-rich protein 1**	SREK1
0,001	-1,32	0,40	**40,05**	Q99728	**BRCA1-associated RING domain protein 1**	BARD1
0,034	-1,32	0,40	**40,05**	Q9ULU4	**Protein kinase C-binding protein 1**	ZMYND8
0,009	-1,32	0,40	**40,05**	Q13144	**Translation initiation factor eIF-2B subunit epsilon**	EIF2B5
0,026	-1,32	0,40	**40,05**	P49459	**Ubiquitin-conjugating enzyme E2 A**	UBE2A
0,002	-1,32	0,40	**39,94**	Q147X3	**N-alpha-acetyltransferase 30**	NAA30
0,031	-1,33	0,40	**39,80**	Q9NYY8	**FAST kinase domain-containing protein 2**	FASTKD2
0,020	-1,33	0,40	**39,78**	Q5SY16	**Polynucleotide 5-hydroxyl-kinase NOL9**	NOL9
0,021	-1,33	0,40	**39,78**	Q9HDC9	**Adipocyte plasma membrane-associated protein**	APMAP
0,015	-1,33	0,40	**39,71**	Q9Y6C9	**Mitochondrial carrier homolog 2**	MTCH2
0,000	-1,33	0,40	**39,66**	Q9H7P9	**Pleckstrin homology domain-containing family G member 2**	PLEKHG2

0,004	-1,34	0,40	**39,60**	Q8IWA0	**WD repeat-containing protein 75**	WDR75
0,016	-1,34	0,40	**39,50**	Q6IA86	**Elongator complex protein 2**	ELP2
0,006	-1,34	0,40	**39,50**	Q13356	**Peptidyl-prolyl cis-trans isomerase-like 2**	PPIL2
0,008	-1,34	0,39	**39,47**	Q13423	**NAD(P) transhydrogenase, mitochondrial**	NNT
0,000	-1,34	0,39	**39,37**	O95140	**Mitofusin-2**	MFN2
0,002	-1,35	0,39	**39,29**	P18065	**Insulin-like growth factor-binding protein 2**	IGFBP2
0,003	-1,35	0,39	**39,23**	Q99496	**E3 ubiquitin-protein ligase RING2**	RNF2
0,028	-1,35	0,39	**39,23**	Q56NI9	**N-acetyltransferase ESCO2**	ESCO2
0,002	-1,35	0,39	**39,23**	Q9P270	**SLAIN motif-containing protein 2**	SLAIN2
0,000	-1,36	0,39	**39,02**	Q13191	**E3 ubiquitin-protein ligase CBL-B**	CBLB
0,032	-1,36	0,39	**38,96**	Q86Y91	**Kinesin-like protein KIF18B**	KIF18B
0,012	-1,36	0,39	**38,96**	Q15648	**Mediator of RNA polymerase II transcription subunit 1**	MED1
0,034	-1,36	0,39	**38,96**	P54132	**Bloom syndrome protein**	BLM
0,007	-1,36	0,39	**38,83**	O15347	**High mobility group protein B3**	HMGB3
0,019	-1,37	0,39	**38,69**	Q8NFU3	**Thiosulfate sulfurtransferase/rhodanese-like domain-containing protein 1**	TSTD1
0,004	-1,37	0,39	**38,69**	P12081	**Histidine--tRNA ligase, cytoplasmic**	HARS
0,015	-1,37	0,39	**38,65**	Q9H1B7	**Interferon regulatory factor 2-binding protein-like**	IRF2BPL
0,039	-1,38	0,39	**38,52**	Q6WCQ1	**Myosin phosphatase Rho-interacting protein**	MPRIP
0,001	-1,38	0,38	**38,42**	Q5UIP0	**Telomere-associated protein RIF1**	RIF1
0,048	-1,38	0,38	**38,31**	P11117	**Lysosomal acid phosphatase**	ACP2
0,037	-1,39	0,38	**38,23**	Q9ULT8	**E3 ubiquitin-protein ligase HECTD1**	HECTD1
0,006	-1,39	0,38	**38,20**	O75717	**WD repeat and HMG-box DNA-binding protein 1**	WDHD1
0,015	-1,39	0,38	**38,16**	Q14508	**WAP four-disulfide core domain protein 2**	WFDC2
0,001	-1,39	0,38	**38,16**	Q86U86	**Protein polybromo-1**	PBRM1
0,012	-1,39	0,38	**38,16**	P62854;Q5JNZ5	**40S ribosomal protein S26;Putative 40S ribosomal protein S26-like 1**	RPS26;RPS26P11
0,023	-1,39	0,38	**38,14**	P05067	**Amyloid beta A4 protein;N-APP;Soluble APP-alpha;Soluble APP-beta;C99;Beta-amyl**	APP
0,005	-1,39	0,38	**38,14**	Q01581	**Hydroxymethylglutaryl-CoA synthase, cytoplasmic**	HMGCS1
0,037	-1,39	0,38	**38,14**	O75382	**Tripartite motif-containing protein 3**	TRIM3
0,001	-1,40	0,38	**37,99**	P02649	**Apolipoprotein E**	APOE
0,005	-1,40	0,38	**37,89**	Q9UBW7	**Zinc finger MYM-type protein 2**	ZMYM2
0,002	-1,40	0,38	**37,89**	O00567	**Nucleolar protein 56**	NOP56
0,001	-1,40	0,38	**37,89**	Q96BP3	**Peptidylprolyl isomerase domain and WD repeat-containing protein 1**	PPWD1
0,041	-1,40	0,38	**37,89**	Q5VSL9	**Striatin-interacting protein 1**	STRIP1

0,018	-1,40	0,38	**37,83**	Q14527	**Helicase-like transcription factor**	HLTF
0,003	-1,41	0,38	**37,68**	P23458	**Tyrosine-protein kinase JAK1**	JAK1
0,011	-1,41	0,38	**37,68**	O14893	**Gem-associated protein 2**	GEMIN2
0,026	-1,41	0,38	**37,67**	Q9BQ52	**Zinc phosphodiesterase ELAC protein 2**	ELAC2
0,011	-1,41	0,38	**37,63**	Q9H501	**ESF1 homolog**	ESF1
0,003	-1,41	0,38	**37,63**	Q96SB4	**SRSF protein kinase 1**	SRPK1
0,021	-1,41	0,38	**37,63**	Q96AQ6	**Pre-B-cell leukemia transcription factor-interacting protein 1**	PBXIP1
0,004	-1,41	0,38	**37,63**	P63098	**Calcineurin subunit B type 1**	PPP3R1
0,013	-1,41	0,38	**37,63**	O15047	**Histone-lysine N-methyltransferase SETD1A**	SETD1A
0,002	-1,41	0,38	**37,58**	Q8WXI7	**Mucin-16**	MUC16
0,002	-1,41	0,38	**37,56**	Q9Y6H1;Q5T1J5	**Coiled-coil-helix-coiled-coil-helix domain-containing protein 2, mitochondrial;Putati**	CHCHD2;CHCHD2P9
0,002	-1,42	0,37	**37,37**	Q8NCW5	**NAD(P)H-hydrate epimerase**	APOA1BP
0,001	-1,42	0,37	**37,37**	Q68D10	**Protein SPT2 homolog**	SPTY2D1
0,019	-1,42	0,37	**37,31**	Q9BY42	**Protein RTF2 homolog**	RTFDC1
0,002	-1,42	0,37	**37,31**	Q9NP92	**28S ribosomal protein S30, mitochondrial**	MRPS30
0,008	-1,43	0,37	**37,13**	P40763	**Signal transducer and activator of transcription 3**	STAT3
0,008	-1,43	0,37	**37,11**	Q5VUA4	**Zinc finger protein 318**	ZNF318
0,022	-1,43	0,37	**37,11**	Q9NPI1	**Bromodomain-containing protein 7**	BRD7
0,043	-1,44	0,37	**36,86**	Q99543	**DnaJ homolog subfamily C member 2;DnaJ homolog subfamily C member 2, N-termi**	DNAJC2
0,005	-1,44	0,37	**36,86**	P46939	**Utrophin**	UTRN
0,000	-1,44	0,37	**36,85**	Q99538	**Legumain**	LGMN
0,040	-1,44	0,37	**36,79**	Q9Y6I4	**Ubiquitin carboxyl-terminal hydrolase 3**	USP3
0,008	-1,45	0,37	**36,60**	P36776	**Lon protease homolog, mitochondrial**	LONP1
0,022	-1,45	0,37	**36,60**	P51648	**Fatty aldehyde dehydrogenase**	ALDH3A2
0,002	-1,45	0,37	**36,60**	Q03252	**Lamin-B2**	LMNB2
0,000	-1,45	0,37	**36,60**	P48729	**Casein kinase I isoform alpha**	CSNK1A1
0,005	-1,45	0,37	**36,60**	Q03112	**MDS1 and EVI1 complex locus protein EVI1**	MECOM
0,033	-1,45	0,37	**36,60**	Q9UER7	**Death domain-associated protein 6**	DAXX
0,016	-1,45	0,37	**36,59**	Q96P70	**Importin-9**	IPO9
0,007	-1,46	0,36	**36,35**	Q5VZL5	**Zinc finger MYM-type protein 4**	ZMYM4
0,024	-1,46	0,36	**36,35**	P19525	**Interferon-induced, double-stranded RNA-activated protein kinase**	EIF2AK2
0,014	-1,46	0,36	**36,31**	Q96EE3	**Nucleoporin SEH1**	SEH1L
0,024	-1,46	0,36	**36,23**	P11802	**Cyclin-dependent kinase 4**	CDK4

0,030	-1,47	0,36	**36,16**	Q96BN8	**Ubiquitin thioesterase otulin**	OTULIN
0,044	-1,47	0,36	**36,10**	O95503	**Chromobox protein homolog 6**	CBX6
0,027	-1,47	0,36	**36,10**	Q13405	**39S ribosomal protein L49, mitochondrial**	MRPL49
0,007	-1,47	0,36	**36,09**	P49821	**NADH dehydrogenase [ubiquinone] flavoprotein 1, mitochondrial**	NDUFV1
0,024	-1,47	0,36	**36,02**	Q15819	**Ubiquitin-conjugating enzyme E2 variant 2**	UBE2V2
0,045	-1,47	0,36	**35,99**	P05023	**Sodium/potassium-transporting ATPase subunit alpha-1**	ATP1A1
0,001	-1,48	0,36	**35,92**	O43278	**Kunitz-type protease inhibitor 1**	SPINT1
0,037	-1,48	0,36	**35,85**	Q9NX46	**Poly(ADP-ribose) glycohydrolase ARH3**	ADPRHL2
0,031	-1,48	0,36	**35,85**	Q15269	**Periodic tryptophan protein 2 homolog**	PWP2
0,003	-1,48	0,36	**35,83**	Q9H792	**Pseudopodium-enriched atypical kinase 1**	PEAK1
0,002	-1,48	0,36	**35,75**	P63151;Q66LE6	**Serine/threonine-protein phosphatase 2A 55 kDa regulatory subunit B alpha isoforn**	PPP2R2A;PPP2R2D
0,002	-1,49	0,36	**35,64**	Q9H3S7	**Tyrosine-protein phosphatase non-receptor type 23**	PTPN23
0,024	-1,49	0,36	**35,63**	Q9BTW9	**Tubulin-specific chaperone D**	TBCD
0,002	-1,49	0,36	**35,60**	O14578	**Citron Rho-interacting kinase**	CIT
0,023	-1,49	0,36	**35,60**	Q14669	**E3 ubiquitin-protein ligase TRIP12**	TRIP12
0,002	-1,49	0,36	**35,60**	Q99661	**Kinesin-like protein KIF2C**	KIF2C
0,032	-1,49	0,36	**35,60**	Q96CN7	**Isochorismatase domain-containing protein 1**	ISOC1
0,033	-1,50	0,35	**35,45**	P63000;P60763;P15153	**Ras-related C3 botulinum toxin substrate 1;Ras-related C3 botulinum toxin substrat**	RAC1;RAC3;RAC2
0,001	-1,50	0,35	**35,36**	P46977	**Dolichyl-diphosphooligosaccharide--protein glycosyltransferase subunit STT3A**	STT3A
0,033	-1,50	0,35	**35,36**	Q9UBR2	**Cathepsin Z**	CTSZ
0,001	-1,50	0,35	**35,36**	Q9Y6W5	**Wiskott-Aldrich syndrome protein family member 2**	WASF2
0,015	-1,50	0,35	**35,28**	Q9HCJ3	**Ribonucleoprotein PTB-binding 2**	RAVER2
0,014	-1,50	0,35	**35,25**	P53680	**AP-2 complex subunit sigma**	AP2S1
0,001	-1,51	0,35	**35,11**	Q9C0J8	**pre-mRNA 3 end processing protein WDR33**	WDR33
0,007	-1,51	0,35	**35,06**	P57076	**UPF0769 protein C21orf59**	C21orf59
0,017	-1,52	0,35	**34,99**	P18615	**Negative elongation factor E**	NELFE
0,006	-1,52	0,35	**34,75**	Q96EI5	**Transcription elongation factor A protein-like 4**	TCEAL4
0,028	-1,53	0,35	**34,73**	Q6NZY4	**Zinc finger CCHC domain-containing protein 8**	ZCCHC8
0,022	-1,53	0,35	**34,63**	O60763	**General vesicular transport factor p115**	USO1
0,047	-1,53	0,35	**34,63**	Q8N3U4	**Cohesin subunit SA-2**	STAG2
0,002	-1,54	0,34	**34,47**	Q9NPD8	**Ubiquitin-conjugating enzyme E2 T**	UBE2T
0,002	-1,54	0,34	**34,39**	O15027	**Protein transport protein Sec16A**	SEC16A
0,025	-1,54	0,34	**34,39**	Q70E73	**Ras-associated and pleckstrin homology domains-containing protein 1**	RAPH1

0,001	-1,54	0,34	**34,32**	Q5VT25	**Serine/threonine-protein kinase MRCK alpha**	CDC42BPA
0,031	-1,55	0,34	**34,27**	P23229	**Integrin alpha-6;Integrin alpha-6 heavy chain;Integrin alpha-6 light chain;Processed**	ITGA6
0,008	-1,55	0,34	**34,15**	P06744	**Glucose-6-phosphate isomerase**	GPI
0,019	-1,55	0,34	**34,14**	Q16678	**Cytochrome P450 1B1**	CYP1B1
0,021	-1,55	0,34	**34,11**	O15213	**WD repeat-containing protein 46**	WDR46
0,025	-1,56	0,34	**33,83**	O14617	**AP-3 complex subunit delta-1**	AP3D1
0,018	-1,58	0,33	**33,45**	Q9NQZ2	**Something about silencing protein 10**	UTP3
0,010	-1,58	0,33	**33,45**	P55786	**Puromycin-sensitive aminopeptidase**	NPEPPS
0,003	-1,58	0,33	**33,45**	Q96B54	**Zinc finger protein 428**	ZNF428
0,002	-1,58	0,33	**33,38**	Q6P1L8	**39S ribosomal protein L14, mitochondrial**	MRPL14
0,012	-1,58	0,33	**33,37**	O95166;Q9H0R8	**Gamma-aminobutyric acid receptor-associated protein;Gamma-aminobutyric acid r**	ABARAP;GABARAPL
0,014	-1,59	0,33	**33,32**	P28331	**NADH-ubiquinone oxidoreductase 75 kDa subunit, mitochondrial**	NDUFS1
0,010	-1,59	0,33	**33,22**	Q13724	**Mannosyl-oligosaccharide glucosidase**	MOGS
0,005	-1,59	0,33	**33,22**	P21741	**Midkine**	MDK
0,028	-1,59	0,33	**33,22**	Q7Z5K2	**Wings apart-like protein homolog**	WAPAL
0,013	-1,59	0,33	**33,22**	P49757	**Protein numb homolog**	NUMB
0,002	-1,59	0,33	**33,19**	Q14739	**Lamin-B receptor**	LBR
0,039	-1,60	0,33	**32,99**	Q92947	**Glutaryl-CoA dehydrogenase, mitochondrial**	GCDH
0,004	-1,60	0,33	**32,99**	Q9UNX4	**WD repeat-containing protein 3**	WDR3
0,040	-1,60	0,33	**32,99**	Q6NV74	**Uncharacterized protein KIAA1211-like**	KIAA1211L
0,015	-1,60	0,33	**32,96**	Q06203	**Amidophosphoribosyltransferase**	PPAT
0,043	-1,60	0,33	**32,95**	P18084	**Integrin beta-5**	ITGB5
0,001	-1,60	0,33	**32,91**	Q9NTJ3	**Structural maintenance of chromosomes protein 4**	SMC4
0,019	-1,61	0,33	**32,76**	Q12955	**Ankyrin-3**	ANK3
0,013	-1,61	0,33	**32,76**	P36404	**ADP-ribosylation factor-like protein 2**	ARL2
0,004	-1,61	0,33	**32,76**	P40937	**Replication factor C subunit 5**	RFC5
0,016	-1,61	0,33	**32,76**	Q92618	**Zinc finger protein 516**	ZNF516
0,001	-1,62	0,33	**32,53**	Q6PL18	**ATPase family AAA domain-containing protein 2**	ATAD2
0,002	-1,62	0,33	**32,53**	Q69YH5	**Cell division cycle-associated protein 2**	CDCA2
0,014	-1,63	0,32	**32,41**	O43760	**Synaptogyrin-2**	SYNGR2
0,000	-1,63	0,32	**32,40**	O43252	**Bifunctional 3-phosphoadenosine 5-phosphosulfate synthase 1;Sulfate adenylyltran**	PAPSS1
0,002	-1,63	0,32	**32,24**	P50851	**Lipopolysaccharide-responsive and beige-like anchor protein**	LRBA
0,025	-1,64	0,32	**32,09**	O75182	**Paired amphipathic helix protein Sin3b**	SIN3B

0,021	-1,64	0,32	**32,09**	Q8WXH0	**Nesprin-2**	SYNE2
0,005	-1,64	0,32	**32,09**	Q8N1G0	**Zinc finger protein 687**	ZNF687
0,003	-1,65	0,32	**31,86**	Q8N726	**Cyclin-dependent kinase inhibitor 2A, isoform 4**	CDKN2A
0,046	-1,65	0,32	**31,86**	Q14BN4	**Sarcolemmal membrane-associated protein**	SLMAP
0,005	-1,65	0,32	**31,86**	Q9Y5J1	**U3 small nucleolar RNA-associated protein 18 homolog**	UTP18
0,032	-1,65	0,32	**31,78**	Q86WA8	**Lon protease homolog 2, peroxisomal**	LONP2
0,028	-1,66	0,32	**31,68**	O14786	**Neuropilin-1**	NRP1
0,000	-1,66	0,32	**31,60**	P31350	**Ribonucleoside-diphosphate reductase subunit M2**	RRM2
0,032	-1,67	0,31	**31,43**	Q13535	**Serine/threonine-protein kinase ATR**	ATR
0,008	-1,68	0,31	**31,22**	Q9Y6D9	**Mitotic spindle assembly checkpoint protein MAD1**	MAD1L1
0,000	-1,68	0,31	**31,21**	Q9NYF8	**Bcl-2-associated transcription factor 1**	BCLAF1
0,003	-1,68	0,31	**31,21**	Q06710	**Paired box protein Pax-8**	PAX8
0,007	-1,69	0,31	**31,03**	A5YKK6	**CCR4-NOT transcription complex subunit 1**	CNOT1
0,035	-1,69	0,31	**30,99**	Q13017	**Rho GTPase-activating protein 5**	ARHGAP5
0,002	-1,69	0,31	**30,99**	P49848	**Transcription initiation factor TFIID subunit 6**	TAF6
0,041	-1,70	0,31	**30,86**	P84103	**Serine/arginine-rich splicing factor 3**	SRSF3
0,002	-1,70	0,31	**30,78**	P30419	**Glycylpeptide N-tetradecanoyltransferase 1**	NMT1
0,002	-1,71	0,30	**30,48**	O95347	**Structural maintenance of chromosomes protein 2**	SMC2
0,015	-1,72	0,30	**30,37**	O75438	**NADH dehydrogenase [ubiquinone] 1 beta subcomplex subunit 1**	NDUFB1
0,040	-1,72	0,30	**30,36**	Q9Y5L0	**Transportin-3**	TNPO3
0,007	-1,72	0,30	**30,35**	Q49AG3	**Zinc finger BED domain-containing protein 5**	ZBED5
0,034	-1,72	0,30	**30,35**	Q93074	**Mediator of RNA polymerase II transcription subunit 12**	MED12
0,021	-1,72	0,30	**30,33**	P61024	**Cyclin-dependent kinases regulatory subunit 1**	CKS1B
0,002	-1,72	0,30	**30,29**	Q9BZE1	**39S ribosomal protein L37, mitochondrial**	MRPL37
0,045	-1,73	0,30	**30,25**	P60953	**Cell division control protein 42 homolog**	CDC42
0,013	-1,73	0,30	**30,24**	Q9Y2D4	**Exocyst complex component 6B**	EXOC6B
0,004	-1,73	0,30	**30,22**	Q9NR31	**GTP-binding protein SAR1a**	SAR1A
0,002	-1,73	0,30	**30,15**	Q14692	**Ribosome biogenesis protein BMS1 homolog**	BMS1
0,008	-1,73	0,30	**30,15**	Q96GM5	**SWI/SNF-related matrix-associated actin-dependent regulator of chromatin subfami**	SMARCD1
0,010	-1,73	0,30	**30,08**	Q9BV57	**1,2-dihydroxy-3-keto-5-methylthiopentene dioxygenase**	ADI1
0,030	-1,73	0,30	**30,06**	O14907	**Tax1-binding protein 3**	TAX1BP3
0,001	-1,74	0,30	**30,01**	Q9NX47	**E3 ubiquitin-protein ligase MARCH5**	MARCH5
0,006	-1,74	0,30	**29,94**	O60934	**Nibrin**	NBN

0,025	-1,74	0,30	**29,94**	Q96K17	**Transcription factor BTF3 homolog 4**	BTF3L4
0,009	-1,74	0,30	**29,94**	O75147	**Obscurin-like protein 1**	OBSL1
0,037	-1,75	0,30	**29,73**	Q07866	**Kinesin light chain 1**	KLC1
0,013	-1,76	0,30	**29,60**	Q8IWR0	**Zinc finger CCCH domain-containing protein 7A**	ZC3H7A
0,009	-1,76	0,30	**29,60**	Q96EX3	**WD repeat-containing protein 34**	WDR34
0,046	-1,76	0,30	**29,52**	Q6P9B9	**Integrator complex subunit 5**	INTS5
0,041	-1,77	0,29	**29,33**	Q9H0U9	**Testis-specific Y-encoded-like protein 1**	TSPYL1
0,032	-1,77	0,29	**29,32**	Q6P597	**Kinesin light chain 3**	KLC3
0,044	-1,77	0,29	**29,32**	Q9NU22	**Midasin**	MDN1
0,001	-1,77	0,29	**29,32**	P57721	**Poly(rC)-binding protein 3**	PCBP3
0,022	-1,78	0,29	**29,12**	Q71RC2	**La-related protein 4**	LARP4
0,007	-1,78	0,29	**29,12**	P14635	**G2/mitotic-specific cyclin-B1**	CCNB1
0,026	-1,79	0,29	**28,95**	Q15291	**Retinoblastoma-binding protein 5**	RBBP5
0,000	-1,79	0,29	**28,86**	Q9Y448	**Small kinetochore-associated protein**	KNSTRN
0,007	-1,80	0,29	**28,72**	Q9Y4A5	**Transformation/transcription domain-associated protein**	TRRAP
0,006	-1,80	0,29	**28,72**	P18887	**DNA repair protein XRCC1**	XRCC1
0,018	-1,80	0,29	**28,72**	O60293	**Zinc finger C3H1 domain-containing protein**	ZFC3H1
0,009	-1,80	0,29	**28,72**	Q15750	**TGF-beta-activated kinase 1 and MAP3K7-binding protein 1**	TAB1
0,046	-1,81	0,29	**28,52**	O60568	**Procollagen-lysine,2-oxoglutarate 5-dioxygenase 3**	PLOD3
0,013	-1,83	0,28	**28,17**	Q5VT52	**Regulation of nuclear pre-mRNA domain-containing protein 2**	RPRD2
0,047	-1,84	0,28	**28,02**	Q8N9B5	**Junction-mediating and -regulatory protein**	JMY
0,041	-1,84	0,28	**27,97**	O75410	**Transforming acidic coiled-coil-containing protein 1**	TACC1
0,006	-1,84	0,28	**27,89**	P23921	**Ribonucleoside-diphosphate reductase large subunit**	RRM1
0,041	-1,85	0,28	**27,74**	Q92547	**DNA topoisomerase 2-binding protein 1**	TOPBP1
0,015	-1,86	0,28	**27,59**	Q8N0X7	**Spartin**	SPG20
0,010	-1,86	0,28	**27,55**	Q5PRF9	**Protein Smaug homolog 2**	SAMD4B
0,039	-1,86	0,28	**27,55**	A3KN83	**Protein strawberry notch homolog 1**	SBNO1
0,001	-1,86	0,28	**27,55**	Q5VYS8	**Terminal uridylyltransferase 7**	ZCCHC6
0,021	-1,87	0,27	**27,40**	P55285	**Cadherin-6**	CDH6
0,032	-1,87	0,27	**27,27**	O15260	**Surfeit locus protein 4**	SURF4
0,002	-1,88	0,27	**27,23**	Q9H9T3	**Elongator complex protein 3**	ELP3
0,041	-1,88	0,27	**27,17**	Q9H977	**WD repeat-containing protein 54**	WDR54
0,004	-1,88	0,27	**27,17**	Q9P2M7	**Cingulin**	CGN

0,004	-1,88	0,27	**27,17**	Q9HBE1	**POZ-, AT hook-, and zinc finger-containing protein 1**	PATZ1
0,000	-1,88	0,27	**27,14**	Q9BVK2	**Probable dolichyl pyrophosphate Glc1Man9GlcNAc2 alpha-1,3-glucosyltransferase**	ALG8
0,028	-1,89	0,27	**27,04**	Q9Y5Y6	**Suppressor of tumorigenicity 14 protein**	ST14
0,030	-1,89	0,27	**26,99**	Q5R3I4	**Tetratricopeptide repeat protein 38**	TTC38
0,002	-1,89	0,27	**26,98**	B1AK53	**Espin**	ESPN
0,005	-1,89	0,27	**26,98**	Q96PE2	**Rho guanine nucleotide exchange factor 17**	ARHGEF17
0,001	-1,90	0,27	**26,87**	Q8N7H5	**RNA polymerase II-associated factor 1 homolog**	PAF1
0,012	-1,90	0,27	**26,79**	P78563	**Double-stranded RNA-specific editase 1**	ADARB1
0,027	-1,91	0,27	**26,61**	Q6ZSR9	**Uncharacterized protein FLJ45252**	
0,026	-1,91	0,27	**26,61**	Q14202	**Zinc finger MYM-type protein 3**	ZMYM3
0,001	-1,92	0,26	**26,45**	O95298;E9PQ53	**NADH dehydrogenase [ubiquinone] 1 subunit C2;NADH dehydrogenase [ubiquinone**	UFC2;NDUFC2-KCTI
0,009	-1,92	0,26	**26,44**	P57678	**Gem-associated protein 4**	GEMIN4
0,024	-1,92	0,26	**26,43**	Q15811	**Intersectin-1**	ITSN1
0,003	-1,92	0,26	**26,43**	Q68E01	**Integrator complex subunit 3**	INTS3
0,006	-1,93	0,26	**26,26**	P24928	**DNA-directed RNA polymerase II subunit RPB1**	POLR2A
0,040	-1,93	0,26	**26,24**	P19404	**NADH dehydrogenase [ubiquinone] flavoprotein 2, mitochondrial**	NDUFV2
0,002	-1,94	0,26	**26,06**	P18583	**Protein SON**	SON
0,031	-1,95	0,26	**25,96**	Q96KB5	**Lymphokine-activated killer T-cell-originated protein kinase**	PBK
0,011	-1,95	0,26	**25,88**	Q13867	**Bleomycin hydrolase**	BLMH
0,019	-1,96	0,26	**25,70**	Q6ZNB6	**NF-X1-type zinc finger protein NFXL1**	NFXL1
0,002	-1,97	0,26	**25,53**	P82663	**28S ribosomal protein S25, mitochondrial**	MRPS25
0,001	-1,97	0,26	**25,53**	Q9BRA0	**N-alpha-acetyltransferase 38, NatC auxiliary subunit**	NAA38
0,016	-1,97	0,26	**25,53**	P13473	**Lysosome-associated membrane glycoprotein 2**	LAMP2
0,020	-1,98	0,25	**25,40**	P04626	**Receptor tyrosine-protein kinase erbB-2**	ERBB2
0,004	-1,98	0,25	**25,28**	P35240	**Merlin**	NF2
0,003	-1,99	0,25	**25,23**	P35573	**Glycogen debranching enzyme;4-alpha-glucanotransferase;Amylo-alpha-1,6-glucosi**	AGL
0,014	-1,99	0,25	**25,17**	O14646	**Chromodomain-helicase-DNA-binding protein 1**	CHD1
0,007	-1,99	0,25	**25,10**	Q6UWZ7	**BRCA1-A complex subunit Abraxas**	FAM175A
0,045	-2,00	0,25	**25,00**	Q3L8U1	**Chromodomain-helicase-DNA-binding protein 9**	CHD9
0,007	-2,00	0,25	**25,00**	P29372	**DNA-3-methyladenine glycosylase**	MPG
0,006	-2,00	0,25	**25,00**	Q9HCB6	**Spondin-1**	SPON1
0,030	-2,01	0,25	**24,90**	Q9GZN8	**UPF0687 protein C20orf27**	C20orf27
0,005	-2,01	0,25	**24,83**	O00268	**Transcription initiation factor TFIID subunit 4**	TAF4

0,000	-2,01	0,25	**24,83**	Q9NSD9	**Phenylalanine--tRNA ligase beta subunit**	FARSB
0,035	-2,02	0,25	**24,74**	O15397	**Importin-8**	IPO8
0,000	-2,02	0,25	**24,66**	Q99715	**Collagen alpha-1(XII) chain**	COL12A1
0,000	-2,02	0,25	**24,66**	P51946	**Cyclin-H**	CCNH
0,023	-2,02	0,25	**24,66**	P53992	**Protein transport protein Sec24C**	SEC24C
0,040	-2,02	0,25	**24,57**	Q9UPN9	**E3 ubiquitin-protein ligase TRIM33**	TRIM33
0,019	-2,04	0,24	**24,32**	Q4FZB7	**Histone-lysine N-methyltransferase SUV420H1**	SUV420H1
0,013	-2,05	0,24	**24,22**	Q8IXQ6	**Poly [ADP-ribose] polymerase 9**	PARP9
0,036	-2,05	0,24	**24,14**	Q9NUQ6	**SPATS2-like protein**	SPATS2L
0,003	-2,06	0,24	**23,91**	O00762	**Ubiquitin-conjugating enzyme E2 C**	UBE2C
0,017	-2,07	0,24	**23,80**	Q13509	**Tubulin beta-3 chain**	TUBB3
0,021	-2,08	0,24	**23,73**	P13591	**Neural cell adhesion molecule 1**	NCAM1
0,000	-2,08	0,24	**23,72**	O95573	**Long-chain-fatty-acid--CoA ligase 3**	ACSL3
0,002	-2,08	0,24	**23,65**	P35251	**Replication factor C subunit 1**	RFC1
0,013	-2,08	0,24	**23,65**	Q9NUQ3	**Gamma-taxilin**	TXLNG
0,006	-2,08	0,24	**23,65**	Q9BXJ9	**N-alpha-acetyltransferase 15, NatA auxiliary subunit**	NAA15
0,018	-2,08	0,24	**23,65**	O43768	**Alpha-endosulfine**	ENSA
0,020	-2,09	0,23	**23,49**	Q9NRL2	**Bromodomain adjacent to zinc finger domain protein 1A**	BAZ1A
0,013	-2,09	0,23	**23,43**	Q9UJX6	**Anaphase-promoting complex subunit 2**	ANAPC2
0,030	-2,10	0,23	**23,33**	Q8TBC4	**NEDD8-activating enzyme E1 catalytic subunit**	UBA3
0,025	-2,10	0,23	**23,33**	Q8NBT2	**Kinetochore protein Spc24**	SPC24
0,006	-2,12	0,23	**23,00**	Q9UL03	**Integrator complex subunit 6**	INTS6
0,025	-2,12	0,23	**22,95**	P54198	**Protein HIRA**	HIRA
0,015	-2,13	0,23	**22,85**	P98095	**Fibulin-2**	FBLN2
0,020	-2,13	0,23	**22,85**	Q9UBC2	**Epidermal growth factor receptor substrate 15-like 1**	EPS15L1
0,004	-2,13	0,23	**22,85**	P30040	**Endoplasmic reticulum resident protein 29**	ERP29
0,004	-2,13	0,23	**22,85**	Q9UBX3	**Mitochondrial dicarboxylate carrier**	SLC25A10
0,031	-2,15	0,23	**22,55**	Q9UQR0	**Sex comb on midleg-like protein 2**	SCML2
0,005	-2,15	0,23	**22,53**	Q9C0J9	**Class E basic helix-loop-helix protein 41**	BHLHE41
0,001	-2,15	0,22	**22,47**	Q12800	**Alpha-globin transcription factor CP2**	TFCP2
0,007	-2,16	0,22	**22,38**	Q12830	**Nucleosome-remodeling factor subunit BPTF**	BPTF
0,000	-2,17	0,22	**22,26**	O43291	**Kunitz-type protease inhibitor 2**	SPINT2
0,004	-2,18	0,22	**22,07**	Q8NI27	**THO complex subunit 2**	THOC2

0,000	-2,19	0,22	**21,92**	Q8NEM7	**Transcription factor SPT20 homolog**	SUPT20H
0,023	-2,20	0,22	**21,72**	Q58FF6	**Putative heat shock protein HSP 90-beta 4**	HSP90AB4P
0,031	-2,22	0,21	**21,46**	Q9UHY1	**Nuclear receptor-binding protein**	NRBP1
0,046	-2,22	0,21	**21,40**	Q5QJE6	**Deoxynucleotidyltransferase terminal-interacting protein 2**	DNTTIP2
0,000	-2,23	0,21	**21,32**	Q9Y3D3	**28S ribosomal protein S16, mitochondrial**	MRPS16
0,013	-2,23	0,21	**21,32**	Q9Y3A2	**Probable U3 small nucleolar RNA-associated protein 11**	UTP11L
0,038	-2,24	0,21	**21,22**	P29375	**Lysine-specific demethylase 5A**	KDM5A
0,005	-2,24	0,21	**21,17**	P33897	**ATP-binding cassette sub-family D member 1**	ABCD1
0,008	-2,24	0,21	**21,17**	Q99622	**Protein C10**	C12orf57
0,006	-2,24	0,21	**21,14**	O95167	**NADH dehydrogenase [ubiquinone] 1 alpha subcomplex subunit 3**	NDUFA3
0,029	-2,24	0,21	**21,11**	P80188	**Neutrophil gelatinase-associated lipocalin**	LCN2
0,021	-2,25	0,21	**21,00**	Q2M389	**WASH complex subunit 7**	KIAA1033
0,020	-2,25	0,21	**20,96**	P16144	**Integrin beta-4**	ITGB4
0,045	-2,26	0,21	**20,90**	Q8TCT9	**Minor histocompatibility antigen H13**	HM13
0,008	-2,26	0,21	**20,88**	Q9NV70	**Exocyst complex component 1**	EXOC1
0,011	-2,27	0,21	**20,75**	O14980	**Exportin-1**	XPO1
0,008	-2,27	0,21	**20,73**	Q92797	**Symplekin**	SYMPK
0,017	-2,28	0,21	**20,59**	Q8N6M0	**OTU domain-containing protein 6B**	OTUD6B
0,000	-2,28	0,21	**20,59**	P36954	**DNA-directed RNA polymerase II subunit RPB9**	POLR2I
0,023	-2,29	0,20	**20,45**	P52434	**DNA-directed RNA polymerases I, II, and III subunit RPABC3**	POLR2H
0,022	-2,30	0,20	**20,33**	Q96Q15	**Serine/threonine-protein kinase SMG1**	SMG1
0,021	-2,30	0,20	**20,32**	Q562R1	**Beta-actin-like protein 2**	ACTBL2
0,020	-2,31	0,20	**20,17**	O75362	**Zinc finger protein 217**	ZNF217
0,025	-2,31	0,20	**20,17**	Q8NI36	**WD repeat-containing protein 36**	WDR36
0,011	-2,33	0,20	**19,89**	Q9Y4C2	**Protein FAM115A**	FAM115A
0,017	-2,33	0,20	**19,88**	Q9UBD5	**Origin recognition complex subunit 3**	ORC3
0,005	-2,35	0,20	**19,61**	Q66PJ3	**ADP-ribosylation factor-like protein 6-interacting protein 4**	ARL6IP4
0,006	-2,35	0,20	**19,58**	P22087	**rRNA 2-O-methyltransferase fibrillarin**	FBL
0,000	-2,36	0,19	**19,48**	Q92576	**PHD finger protein 3**	PHF3
0,000	-2,36	0,19	**19,48**	Q9Y285	**Phenylalanine--tRNA ligase alpha subunit**	FARSA
0,006	-2,37	0,19	**19,36**	P22681	**E3 ubiquitin-protein ligase CBL**	CBL
0,039	-2,38	0,19	**19,21**	A1X283	**SH3 and PX domain-containing protein 2B**	SH3PXD2B
0,035	-2,38	0,19	**19,17**	Q8TB45	**DEP domain-containing mTOR-interacting protein**	DEPTOR

0,049	-2,39	0,19	**19,08**	Q8N680	**Zinc finger and BTB domain-containing protein 2**	ZBTB2
0,023	-2,40	0,19	**18,95**	Q9NVF7	**F-box only protein 28**	FBXO28
0,028	-2,43	0,19	**18,58**	Q6RFH5	**WD repeat-containing protein 74**	WDR74
0,027	-2,43	0,19	**18,52**	P01876;P01877	**Ig alpha-1 chain C region;Ig alpha-2 chain C region**	IGHA1;IGHA2
0,013	-2,44	0,18	**18,43**	Q9Y295	**Developmentally-regulated GTP-binding protein 1**	DRG1
0,003	-2,44	0,18	**18,38**	Q8WVX9	**Fatty acyl-CoA reductase 1**	FAR1
0,001	-2,45	0,18	**18,27**	P30504	**HLA class I histocompatibility antigen, Cw-4 alpha chain**	HLA-C
0,016	-2,46	0,18	**18,23**	O95159	**Zinc finger protein-like 1**	ZFPL1
0,023	-2,46	0,18	**18,17**	Q92917	**G patch domain and KOW motifs-containing protein**	GPKOW
0,000	-2,48	0,18	**17,97**	Q9UHG0	**Doublecortin domain-containing protein 2**	DCDC2
0,028	-2,48	0,18	**17,97**	P16615	**Sarcoplasmic/endoplasmic reticulum calcium ATPase 2**	ATP2A2
0,028	-2,48	0,18	**17,92**	Q6DKI1	**60S ribosomal protein L7-like 1**	RPL7L1
0,001	-2,49	0,18	**17,80**	Q15047	**Histone-lysine N-methyltransferase SETDB1**	SETDB1
0,044	-2,51	0,18	**17,56**	Q02978	**Mitochondrial 2-oxoglutarate/malate carrier protein**	SLC25A11
0,046	-2,51	0,18	**17,56**	P04150	**Glucocorticoid receptor**	NR3C1
0,000	-2,52	0,17	**17,43**	Q9UIV1	**CCR4-NOT transcription complex subunit 7**	CNOT7
0,001	-2,52	0,17	**17,43**	Q15542	**Transcription initiation factor TFIID subunit 5**	TAF5
0,000	-2,52	0,17	**17,41**	O14672	**Disintegrin and metalloproteinase domain-containing protein 10**	ADAM10
0,001	-2,53	0,17	**17,31**	P35250	**Replication factor C subunit 2**	RFC2
0,000	-2,56	0,17	**16,93**	Q9Y580	**RNA-binding protein 7**	RBM7
0,002	-2,57	0,17	**16,90**	Q15021	**Condensin complex subunit 1**	NCAPD2
0,007	-2,57	0,17	**16,79**	Q9H944	**Mediator of RNA polymerase II transcription subunit 20**	MED20
0,007	-2,58	0,17	**16,77**	P10909	**Clusterin;Clusterin beta chain;Clusterin alpha chain**	CLU
0,005	-2,58	0,17	**16,72**	Q9UI12	**V-type proton ATPase subunit H**	ATP6V1H
0,001	-2,59	0,17	**16,61**	O75153	**Clustered mitochondria protein homolog**	CLUH
0,010	-2,59	0,17	**16,61**	Q14644	**Ras GTPase-activating protein 3**	RASA3
0,002	-2,60	0,16	**16,50**	Q86YS3	**Rab11 family-interacting protein 4**	RAB11FIP4
0,002	-2,60	0,16	**16,49**	Q6KC79	**Nipped-B-like protein**	NIPBL
0,000	-2,60	0,16	**16,45**	Q14687	**Genetic suppressor element 1**	GSE1
0,044	-2,63	0,16	**16,15**	Q9H204	**Mediator of RNA polymerase II transcription subunit 28**	MED28
0,027	-2,63	0,16	**16,15**	Q9UPP1	**Histone lysine demethylase PHF8**	PHF8
0,014	-2,63	0,16	**16,15**	Q92616	**Translational activator GCN1**	GCN1L1
0,003	-2,64	0,16	**16,06**	Q8TCG1	**Protein CIP2A**	KIAA1524

0,015	-2,64	0,16	**16,05**	P62273	**40S ribosomal protein S29**	RPS29
0,041	-2,64	0,16	**16,00**	O15551	**Claudin-3**	CLDN3
0,026	-2,65	0,16	**15,93**	Q9BQ70	**Transcription factor 25**	TCF25
0,001	-2,66	0,16	**15,82**	Q9BTY7;P0CB43	**Protein HGH1 homolog**	HGH1
0,020	-2,66	0,16	**15,78**	P01034	**Cystatin-C**	CST3
0,000	-2,70	0,15	**15,39**	Q9HCM1	**Uncharacterized protein KIAA1551**	KIAA1551
0,035	-2,72	0,15	**15,19**	P06865	**Beta-hexosaminidase subunit alpha**	HEXA
0,048	-2,72	0,15	**15,18**	Q92794	**Histone acetyltransferase KAT6A**	KAT6A
0,000	-2,73	0,15	**15,07**	Q68CP9	**AT-rich interactive domain-containing protein 2**	ARID2
0,002	-2,73	0,15	**15,07**	Q9NV06	**DDB1- and CUL4-associated factor 13**	DCAF13
0,001	-2,73	0,15	**15,07**	P14373	**Zinc finger protein RFP**	TRIM27
0,016	-2,74	0,15	**14,97**	Q9NQW7	**Xaa-Pro aminopeptidase 1**	XPNPEP1
0,000	-2,75	0,15	**14,89**	Q53EL6	**Programmed cell death protein 4**	PDCD4
0,046	-2,77	0,15	**14,69**	Q8ND76	**Cyclin-Y**	CCNY
0,015	-2,79	0,14	**14,46**	Q8IYA6	**Cytoskeleton-associated protein 2-like**	CKAP2L
0,003	-2,80	0,14	**14,32**	P0CG12	**Chromosome transmission fidelity protein 8 homolog isoform 2**	CHTF8
0,002	-2,81	0,14	**14,24**	Q9Y320	**Thioredoxin-related transmembrane protein 2**	TMX2
0,024	-2,81	0,14	**14,21**	Q9P0P0	**E3 ubiquitin-protein ligase RNF181**	RNF181
0,035	-2,82	0,14	**14,16**	P0CAP2	**DNA-directed RNA polymerase II subunit GRINL1A**	POLR2M
0,035	-2,82	0,14	**14,16**	Q9NYH9	**U3 small nucleolar RNA-associated protein 6 homolog**	UTP6
0,000	-2,84	0,14	**13,97**	Q92610	**Zinc finger protein 592**	ZNF592
0,002	-2,86	0,14	**13,77**	Q52LW3	**Rho GTPase-activating protein 29**	ARHGAP29
0,035	-2,87	0,14	**13,68**	Q69YN4	**Protein virilizer homolog**	KIAA1429
0,047	-2,88	0,14	**13,58**	P28340	**DNA polymerase delta catalytic subunit**	POLD1
0,021	-2,88	0,14	**13,58**	Q14686	**Nuclear receptor coactivator 6**	NCOA6
0,042	-2,89	0,14	**13,52**	Q9Y653	**G-protein coupled receptor 56;GPR56 N-terminal fragment;GPR56 C-terminal fragm**	GPR56
0,029	-2,89	0,13	**13,49**	P04114	**Apolipoprotein B-100;Apolipoprotein B-48**	APOB
0,013	-2,91	0,13	**13,30**	Q96GN5	**Cell division cycle-associated 7-like protein**	CDCA7L
0,011	-2,92	0,13	**13,21**	P50613	**Cyclin-dependent kinase 7**	CDK7
0,003	-2,92	0,13	**13,17**	P29083	**General transcription factor IIE subunit 1**	GTF2E1
0,026	-2,95	0,13	**12,94**	P40938	**Replication factor C subunit 3**	RFC3
0,005	-2,96	0,13	**12,85**	P98160	**Basement membrane-specific heparan sulfate proteoglycan core protein;Endorepell**	HSPG2
0,021	-2,96	0,13	**12,85**	Q9H2U1	**ATP-dependent RNA helicase DHX36**	DHX36

0,024	-2,96	0,13	**12,85**	P09211	**Glutathione S-transferase P**	GSTP1
0,002	-2,98	0,13	**12,67**	P49454	**Centromere protein F**	CENPF
0,013	-3,00	0,13	**12,52**	Q7Z392	**Trafficking protein particle complex subunit 11**	TRAPPC11
0,000	-3,00	0,13	**12,50**	P54886	**Delta-1-pyrroline-5-carboxylate synthase;Glutamate 5-kinase;Gamma-glutamyl pho**	ALDH18A1
0,024	-3,01	0,12	**12,41**	ː13554;Q13555;Q9UQM	**Calcium/calmodulin-dependent protein kinase type II subunit beta;Calcium/calmod**	IK2B;CAMK2G;CAM
0,001	-3,01	0,12	**12,41**	Q9BZI7	**Regulator of nonsense transcripts 3B**	UPF3B
0,012	-3,03	0,12	**12,26**	Q03426	**Mevalonate kinase**	MVK
0,000	-3,03	0,12	**12,24**	Q9BXY0	**Protein MAK16 homolog**	MAK16
0,009	-3,06	0,12	**12,02**	Q9C037	**Tripartite motif-containing protein 4**	TRIM4
0,013	-3,07	0,12	**11,91**	O14503	**Class E basic helix-loop-helix protein 40**	BHLHE40
0,005	-3,09	0,12	**11,74**	Q16513	**Serine/threonine-protein kinase N2**	PKN2
0,016	-3,12	0,12	**11,50**	P49902	**Cytosolic purine 5-nucleotidase**	NT5C2
0,002	-3,12	0,12	**11,50**	Q9NQW6	**Actin-binding protein anillin**	ANLN
0,048	-3,12	0,11	**11,50**	O94819	**Kelch repeat and BTB domain-containing protein 11**	KBTBD11
0,003	-3,13	0,11	**11,42**	Q15545	**Transcription initiation factor TFIID subunit 7**	TAF7
0,006	-3,14	0,11	**11,34**	Q6VMQ6	**Activating transcription factor 7-interacting protein 1**	ATF7IP
0,034	-3,15	0,11	**11,24**	Q9Y4P1	**Cysteine protease ATG4B**	ATG4B
0,036	-3,16	0,11	**11,19**	Q4U2R6	**39S ribosomal protein L51, mitochondrial**	MRPL51
0,016	-3,20	0,11	**10,88**	Q8N5L8	**Ribonuclease P protein subunit p25-like protein**	RPP25L
0,002	-3,20	0,11	**10,88**	Q96BD5	**PHD finger protein 21A**	PHF21A
0,004	-3,22	0,11	**10,70**	O75882	**Attractin**	ATRN
0,024	-3,23	0,11	**10,68**	Q9H0A8	**COMM domain-containing protein 4**	COMMD4
0,031	-3,23	0,11	**10,66**	Q99470	**Stromal cell-derived factor 2**	SDF2
0,000	-3,28	0,10	**10,29**	P16333	**Cytoplasmic protein NCK1**	NCK1
0,030	-3,28	0,10	**10,29**	Q96QE3	**ATPase family AAA domain-containing protein 5**	ATAD5
0,031	-3,30	0,10	**10,15**	Q15054	**DNA polymerase delta subunit 3**	POLD3
0,010	-3,31	0,10	**10,08**	Q9HD26	**Golgi-associated PDZ and coiled-coil motif-containing protein**	GOPC
0,024	-3,41	0,09	**9,41**	P17535	**Transcription factor jun-D**	JUND
0,013	-3,46	0,09	**9,09**	Q9UNQ2	**Probable dimethyladenosine transferase**	DIMT1
0,001	-3,47	0,09	**9,02**	Q9BZF9	**Uveal autoantigen with coiled-coil domains and ankyrin repeats**	UACA
0,016	-3,56	0,08	**8,48**	Q9Y2P8	**RNA 3-terminal phosphate cyclase-like protein**	RCL1
0,041	-3,62	0,08	**8,12**	Q15434	**RNA-binding motif, single-stranded-interacting protein 2**	RBMS2
0,006	-3,65	0,08	**7,97**	Q49MG5	**Microtubule-associated protein 9**	MAP9

0,000	-3,72	0,08	**7,59**	Q9NXX6	**Non-structural maintenance of chromosomes element 4 homolog A**	NSMCE4A
0,002	-3,73	0,08	**7,55**	Q96AX1	**Vacuolar protein sorting-associated protein 33A**	VPS33A
0,010	-3,73	0,08	**7,54**	Q9BXR0	**Queuine tRNA-ribosyltransferase**	QTRT1
0,020	-3,73	0,08	**7,54**	Q9Y2Z4	**Tyrosine--tRNA ligase, mitochondrial**	YARS2
0,001	-3,74	0,07	**7,47**	O15075	**Serine/threonine-protein kinase DCLK1**	DCLK1
0,000	-3,85	0,07	**6,93**	Q9BXS6	**Nucleolar and spindle-associated protein 1**	NUSAP1
0,015	-4,02	0,06	**6,17**	Q9Y2Q5	**Ragulator complex protein LAMTOR2**	LAMTOR2
0,000	-4,03	0,06	**6,12**	Q9Y2W1	**Thyroid hormone receptor-associated protein 3**	THRAP3
0,002	-4,07	0,06	**5,95**	P78549	**Endonuclease III-like protein 1**	NTHL1
0,023	-4,15	0,06	**5,62**	Q0VGL1	**Ragulator complex protein LAMTOR4;Ragulator complex protein LAMTOR4, N-termi**	LAMTOR4
0,000	-4,30	0,05	**5,07**	O14773	**Tripeptidyl-peptidase 1**	TPP1
0,018	-4,48	0,04	**4,48**	O14965	**Aurora kinase A**	AURKA
0,000	-4,72	0,04	**3,79**	O95235	**Kinesin-like protein KIF20A**	KIF20A
0,001	-5,21	0,03	**2,70**	Q9NRZ9	**Lymphoid-specific helicase**	HELLS
0,000	-5,55	0,02	**2,13**	Q5XUX1	**F-box/WD repeat-containing protein 9**	FBXW9
0,000	-6,23	0,01	**1,33**	Q9P2K8	**Eukaryotic translation initiation factor 2-alpha kinase 4**	EIF2AK4
0,000	-6,25	0,01	**1,31**	P62875	**DNA-directed RNA polymerases I, II, and III subunit RPABC5**	POLR2L

Color Code

8h Up
24h Up
8h Down
24h Down

Supplemental Table S3: DAVID-assisted shotgun proteomic analysis of key cell processes in SKOV3 and OVCAR3 cells exposed to 40µM G28UCM using BioCarta and KEGG databases.

		SKOV3/OVCAR3		SKOV3		OVCAR3	
**UniProt-ID**	**Protein Name**	**Matching Score**		**Expression**		**Expression**	
		**8h**	**24h**	**8h**	**24h**	**8h**	**24h**
FATTY ACID METABOLISM AND BETA OXIDATION							
P24752	acetyl-CoA acetyltransferase 1(ACAT1)						
Q9BWD1	acetyl-CoA acetyltransferase 2(ACAT2)						
P09110	acetyl-CoA acyltransferase 1(ACAA1)						
Q13085	acetyl-CoA carboxylase alpha(ACACA)		1,00				
P16219	acyl-CoA dehydrogenase, C-2 to C-3 short chain(ACADS)						
P45954	acyl-CoA dehydrogenase, short/branched chain(ACADSB)						
P49748	acyl-CoA dehydrogenase, very long chain(ACADVL)						
P33121	acyl-CoA synthetase long-chain family member 1(ACSL1)		1,00				
O95573	acyl-CoA synthetase long-chain family member 3(ACSL3)		1,00				
P49419	aldehyde dehydrogenase 7 family member A1(ALDH7A1)						
P49189	aldehyde dehydrogenase 9 family member A1(ALDH9A1)						
P50416	carnitine palmitoyltransferase 1A(CPT1A)		1,00				
P42126	enoyl-CoA delta isomerase 1(ECI1)						
P49327	fatty acid synthase(FASN)	1,00	1,00				
P40939	hydroxyacyl-CoA dehydrogenase (trifunctional protein), alpha subunit(HADHA)						
P55084	hydroxyacyl-CoA dehydrogenase (trifunctional protein), beta subunit(HADHB)						
Q16836	hydroxyacyl-CoA dehydrogenase(HADH)						
Q53GQ0	hydroxysteroid 17-beta dehydrogenase 12(HSD17B12), very-long-chain 3-oxoacyl-CoA reductase		1,00				
Q9BY49	peroxisomal trans-2-enoyl-CoA reductase(PECR)						
O00767	stearoyl-CoA desaturase(SCD)	1,00	1,00				
	**Mean SKOV3/OVCAR3 Matching Score for fatty acid metabolism and beta oxidation**	**0,10**	**0,35**				
CENTRAL CARBON METABOLISM							
General carbon metabolism							
Q6NVY1	3-hydroxyisobutyryl-CoA hydrolase(HIBCH)						
O95336	6-phosphogluconolactonase(PGLS)						
P24752	acetyl-CoA acetyltransferase 1(ACAT1)						
Q9BWD1	acetyl-CoA acetyltransferase 2(ACAT2)						
Q99798	aconitase 2(ACO2)	1,00	1,00				
P16219	acyl-CoA dehydrogenase, C-2 to C-3 short chain(ACADS)						
Q9NUB1	acyl-CoA synthetase short-chain family member 1(ACSS1)						
Q9NR19	acyl-CoA synthetase short-chain family member 2(ACSS2)						
P04075	aldolase, fructose-bisphosphate A(ALDOA)						
P09972	aldolase, fructose-bisphosphate C(ALDOC)						
P04040	catalase(CAT)						
O75390	citrate synthase(CS)						
P10515	dihydrolipoamide S-acetyltransferase(DLAT)						

P36957	dihydrolipoamide S-succinyltransferase(DLST)						
P13929	enolase 3(ENO3)						
P10768	esterase D(ESD)						
P07954	fumarate hydratase(FH)						
P11413	glucose-6-phosphate dehydrogenase(G6PD)						
P06744	glucose-6-phosphate isomerase(GPI)						
P17174	glutamic-oxaloacetic transaminase 1(GOT1)						
P00505	glutamic-oxaloacetic transaminase 2(GOT2)						
P52789	hexokinase 2(HK2)	1,00					
Q2TB90	hexokinase domain containing 1(HKDC1)						
O95479	hexose-6-phosphate dehydrogenase/glucose 1-dehydrogenase(H6PD)						
P40939	hydroxyacyl-CoA dehydrogenase (trifunctional protein), alpha subunit(HADHA)						
P50213	isocitrate dehydrogenase 3 (NAD(+)) alpha(IDH3A)						
O43837	isocitrate dehydrogenase 3 (NAD(+)) beta(IDH3B)						
P51553	isocitrate dehydrogenase 3 (NAD(+)) gamma(IDH3G)						
P40925	malate dehydrogenase 1(MDH1)						
P40926	malate dehydrogenase 2(MDH2)						
P48163	malic enzyme 1(ME1)						
Q02218	oxoglutarate dehydrogenase(OGDH)						
P17858	phosphofructokinase, liver type(PFKL)						
P08237	phosphofructokinase, muscle(PFKM)						
Q01813	phosphofructokinase, platelet(PFKP)						
P52209	phosphogluconate dehydrogenase(PGD)	1,00					
O43175	phosphoglycerate dehydrogenase(PHGDH)						
P18669	phosphoglycerate mutase 1(PGAM1)	1,00					
P60891	phosphoribosyl pyrophosphate synthetase 1(PRPS1)						
Q9Y617	phosphoserine aminotransferase 1(PSAT1)						
P78330	phosphoserine phosphatase(PSPH)						
P05165	propionyl-CoA carboxylase alpha subunit(PCCA)						
P05166	propionyl-CoA carboxylase beta subunit(PCCB)						
P11498	pyruvate carboxylase(PC)						
P11177	pyruvate dehydrogenase (lipoamide) beta(PDHB)						
Q96AT9	ribulose-5-phosphate-3-epimerase(RPE)						
P34896	serine hydroxymethyltransferase 1(SHMT1)						
P34897	serine hydroxymethyltransferase 2(SHMT2)						
P31040	succinate dehydrogenase complex flavoprotein subunit A(SDHA)						
P21912	succinate dehydrogenase complex iron sulfur subunit B(SDHB)						
Q9P2R7	succinate-CoA ligase ADP-forming beta subunit(SUCLA2)						
P53597	succinate-CoA ligase alpha subunit(SUCLG1)						
P37837	transaldolase 1(TALDO1)	1,00					
P29401	transketolase(TKT)						
Q3LXA3	triokinase and FMN cyclase(TKFC)						
P60174	triosephosphate isomerase 1(TPI1)						
	Mean SKOV3/OVCAR3 Matching Score for general carbon metabolism	0,09	0,02				
Glycolysis							

Q9NR19	acyl-CoA synthetase short-chain family member 2(ACSS2)						
P30838	aldehyde dehydrogenase 3 family member A1(ALDH3A1)						
P49419	aldehyde dehydrogenase 7 family member A1(ALDH7A1)						
P49189	aldehyde dehydrogenase 9 family member A1(ALDH9A1)	1,00					
P14550	aldo-keto reductase family 1 member A1(AKR1A1)						
P04075	aldolase, fructose-bisphosphate A(ALDOA)						
P09972	aldolase, fructose-bisphosphate C(ALDOC)						
P10515	dihydrolipoamide S-acetyltransferase(DLAT)						
P13929	enolase 3(ENO3)						
P06744	glucose-6-phosphate isomerase(GPI)						
P52789	hexokinase 2(HK2)	1,00					
Q2TB90	hexokinase domain containing 1(HKDC1)						
P00338	lactate dehydrogenase A(LDHA)						
P07195	lactate dehydrogenase B(LDHB)						
P17858	phosphofructokinase, liver type(PFKL)						
P08237	phosphofructokinase, muscle(PFKM)						
Q96G03	phosphoglucomutase 2(PGM2)						
P18669	phosphoglycerate mutase 1(PGAM1)	1,00					
P11177	pyruvate dehydrogenase (lipoamide) beta(PDHB)						
P60174	triosephosphate isomerase 1(TPI1)						
	Mean SKOV3/OVCAR3 Matching Score for glycolysis	0,15	0,00				
Pentose phosphate pathway							
O95336	6-phosphogluconolactonase(PGLS)						
P04075	aldolase, fructose-bisphosphate A(ALDOA)						
P09972	aldolase, fructose-bisphosphate C(ALDOC)						
P11413	glucose-6-phosphate dehydrogenase(G6PD)						
P06744	glucose-6-phosphate isomerase(GPI)						
O95479	hexose-6-phosphate dehydrogenase (H6PD)						
P17858	phosphofructokinase, liver type(PFKL)						
P08237	phosphofructokinase, muscle(PFKM)						
Q96G03	phosphoglucomutase 2(PGM2)						
P52209	phosphogluconate dehydrogenase(PGD)	1,00					
P60891	phosphoribosyl pyrophosphate synthetase 1(PRPS1)						
Q96AT9	ribulose-5-phosphate-3-epimerase(RPE)						
P37837	transaldolase 1(TALDO1)	1,00					
P29401	transketolase(TKT)						
	Mean SKOV3/OVCAR3 Matching Score for pentose phosphate pathway	0,14	0,00				
TCA cycle							
Q99798	aconitase 2(ACO2)	1,00	1,00				
P53396	ATP citrate lyase(ACLY)						
O75390	citrate synthase(CS)						
P10515	dihydrolipoamide S-acetyltransferase(DLAT)						
P36957	dihydrolipoamide S-succinyltransferase(DLST)						
P07954	fumarate hydratase(FH)						
P50213	isocitrate dehydrogenase 3 (NAD(+)) alpha(IDH3A)						

O43837	isocitrate dehydrogenase 3 (NAD(+)) beta(IDH3B)						
P51553	isocitrate dehydrogenase 3 (NAD(+)) gamma(IDH3G)						
P40925	malate dehydrogenase 1(MDH1)						
Q02218	oxoglutarate dehydrogenase(OGDH)						
P11498	pyruvate carboxylase(PC)						
P11177	pyruvate dehydrogenase (lipoamide) beta(PDHB)						
P31040	succinate dehydrogenase complex flavoprotein subunit A(SDHA)						
P21912	succinate dehydrogenase complex iron sulfur subunit B(SDHB)						
Q9P2R7	succinate-CoA ligase ADP-forming beta subunit(SUCLA2)						
P53597	succinate-CoA ligase alpha subunit(SUCLG1)						
	Mean SKOV3/OVCAR3 Matching Score for TCA cycle	0,06	0,06				
	**Mean SKOV3/OVCAR3 Matching Score for central carbon metabolism**	**0,10**	**0,02**				
OXIDATIVE PHOSPHORYLATION AND ELECTRON TRANSPORT							
P25705	ATP synthase, H+ transporting, mitochondrial F1 complex, alpha subunit 1 (ATP5A1)						
P48047	ATP synthase, H+ transporting, mitochondrial F1 complex, O subunit(ATP5O)						
O75947	ATP synthase, H+ transporting, mitochondrial Fo complex subunit D(ATP5H)						
P56385	ATP synthase, H+ transporting, mitochondrial Fo complex subunit E(ATP5I)						
P56134	ATP synthase, H+ transporting, mitochondrial Fo complex subunit F2(ATP5J2)						
P18859	ATP synthase, H+ transporting, mitochondrial Fo complex subunit F6(ATP5J)						
P61421	ATPase H+ transporting V0 subunit d1(ATP6V0D1)						
P38606	ATPase H+ transporting V1 subunit A(ATP6V1A)						
P21281	ATPase H+ transporting V1 subunit B2(ATP6V1B2)	1,00					
P21283	ATPase H+ transporting V1 subunit C1(ATP6V1C1)	1,00					
Q9Y5K8	ATPase H+ transporting V1 subunit D(ATP6V1D)						
P36543	ATPase H+ transporting V1 subunit E1(ATP6V1E1)						
Q16864	ATPase H+ transporting V1 subunit F(ATP6V1F)	1,00					
O75348	ATPase H+ transporting V1 subunit G1(ATP6V1G1)						
Q9UI12	ATPase H+ transporting V1 subunit H(ATP6V1H)						
P13073	cytochrome c oxidase subunit 4I1(COX4I1)						
P20674	cytochrome c oxidase subunit 5A(COX5A)						
P10606	cytochrome c oxidase subunit 5B(COX5B)						
P14854	cytochrome c oxidase subunit 6B1(COX6B1)						
P14406	cytochrome c oxidase subunit 7A2(COX7A2)						
P08574	cytochrome c1(CYC1)						
P43304	glycerol-3-phosphate dehydrogenase 2(GPD2)						
P28331	NADH:ubiquinone oxidoreductase core subunit S1(NDUFS1)		1,00				
O75489	NADH:ubiquinone oxidoreductase core subunit S3(NDUFS3)						
O75251	NADH:ubiquinone oxidoreductase core subunit S7(NDUFS7)						
O00217	NADH:ubiquinone oxidoreductase core subunit S8(NDUFS8)						
P49821	NADH:ubiquinone oxidoreductase core subunit V1(NDUFV1)		1,00				
P19404	NADH:ubiquinone oxidoreductase core subunit V2(NDUFV2)	1,00	1,00				
Q86Y39	NADH:ubiquinone oxidoreductase subunit A11(NDUFA11)						
Q9UI09	NADH:ubiquinone oxidoreductase subunit A12(NDUFA12)						

Q9P0J0	NADH:ubiquinone oxidoreductase subunit A13(NDUFA13)						
O43678	NADH:ubiquinone oxidoreductase subunit A2(NDUFA2)						
Q16718	NADH:ubiquinone oxidoreductase subunit A5(NDUFA5)						
P56556	NADH:ubiquinone oxidoreductase subunit A6(NDUFA6)						
O95182	NADH:ubiquinone oxidoreductase subunit A7(NDUFA7)						
P51970	NADH:ubiquinone oxidoreductase subunit A8(NDUFA8)		1,00				
O96000	NADH:ubiquinone oxidoreductase subunit B10(NDUFB10)		1,00				
O43676	NADH:ubiquinone oxidoreductase subunit B3(NDUFB3)						
P17568	NADH:ubiquinone oxidoreductase subunit B7(NDUFB7)						
Q9Y6M9	NADH:ubiquinone oxidoreductase subunit B9(NDUFB9)						
O95298	NADH:ubiquinone oxidoreductase subunit C2(NDUFC2)						
O43181	NADH:ubiquinone oxidoreductase subunit S4(NDUFS4)						
O43920	NADH:ubiquinone oxidoreductase subunit S5(NDUFS5)		1,00				
O75380	NADH:ubiquinone oxidoreductase subunit S6(NDUFS6)						
E9PQ53	NDUFC2-KCTD14 readthrough(NDUFC2-KCTD14)						
Q9H008	phospholysine phosphohistidine inorganic pyrophosphate phosphatase(LHPP)						
Q15181	pyrophosphatase (inorganic) 1(PPA1)						
Q9H2U2	pyrophosphatase (inorganic) 2(PPA2)						
P12235	solute carrier family 25 member 4(SLC25A4)		1,00				
P12236	solute carrier family 25 member 6(SLC25A6)		1,00				
P31040	succinate dehydrogenase complex flavoprotein subunit A(SDHA)						
P21912	succinate dehydrogenase complex iron sulfur subunit B(SDHB)						
P14927	ubiquinol-cytochrome c reductase binding protein(UQCRB)						
P31930	ubiquinol-cytochrome c reductase core protein I(UQCRC1)						
P22695	ubiquinol-cytochrome c reductase core protein II(UQCRC2)						
P07919	ubiquinol-cytochrome c reductase hinge protein(UQCRH)						
P47985	ubiquinol-cytochrome c reductase, Rieske iron-sulfur polypeptide 1(UQCRFS1)						
	**Mean SKOV3/OVCAR3 Matching Score for oxidative phosphorylation and electron transport**	**0,07**	**0,14**				
PROTEIN EXPRESSION							
Basal transcription factors							
P18074	ERCC excision repair 2, TFIIH core complex helicase subunit(ERCC2)		1,00				
P19447	ERCC excision repair 3, TFIIH core complex helicase subunit(ERCC3)						
Q00403	general transcription factor IIB(GTF2B)						
P29083	general transcription factor IIE subunit 1(GTF2E1)						
P29084	general transcription factor IIE subunit 2(GTF2E2)						
P35269	general transcription factor IIF subunit 1(GTF2F1)						
P32780	general transcription factor IIH subunit 1(GTF2H1)						
Q13888	general transcription factor IIH subunit 2(GTF2H2)						
Q13889	general transcription factor IIH subunit 3(GTF2H3)						
Q6ZYL4	general transcription factor IIH subunit 5(GTF2H5)						
P78347	general transcription factor IIi(GTF2I)						
P51948	MNAT1, CDK activating kinase assembly factor(MNAT1)						
Q12962	TATA-box binding protein associated factor 10(TAF10)						
Q92804	TATA-box binding protein associated factor 15(TAF15)						

O00268	TATA-box binding protein associated factor 4(TAF4)						
Q15542	TATA-box binding protein associated factor 5(TAF5)						
P49848	TATA-box binding protein associated factor 6(TAF6)						
Q15545	TATA-box binding protein associated factor 7(TAF7)	1,00					
Q7Z7C8	TATA-box binding protein associated factor 8(TAF8)						
Q9HBM6	TATA-box binding protein associated factor 9b(TAF9B)						
	Mean SKOV3/OVCAR3 Matching Score for basal transcription factors	0,05	0,05				
Aminoacyl-tRNA biosynthesis							
Q5JTZ9	alanyl-tRNA synthetase 2, mitochondrial(AARS2)						
P49588	alanyl-tRNA synthetase(AARS)						
P54136	arginyl-tRNA synthetase(RARS)		1,00				
Q6PI48	aspartyl-tRNA synthetase 2, mitochondrial(DARS2)						
P49589	cysteinyl-tRNA synthetase(CARS)						
O75879	glutamyl-tRNA amidotransferase subunit B(GATB)						
O43716	glutamyl-tRNA amidotransferase subunit C(GATC)						
P41250	glycyl-tRNA synthetase(GARS)						
P49590	histidyl-tRNA synthetase 2, mitochondrial(HARS2)						
P12081	histidyl-tRNA synthetase(HARS)		1,00				
Q9NSE4	isoleucyl-tRNA synthetase 2, mitochondrial(IARS2)						
P41252	isoleucyl-tRNA synthetase(IARS)		1,00				
Q9P2J5	leucyl-tRNA synthetase(LARS)						
Q15046	lysyl-tRNA synthetase(KARS)						
P56192	methionyl-tRNA synthetase(MARS)						
Q9Y285	phenylalanyl-tRNA synthetase alpha subunit(FARSA)						
Q9NSD9	phenylalanyl-tRNA synthetase beta subunit(FARSB)		1,00				
Q9NP81	seryl-tRNA synthetase 2, mitochondrial(SARS2)						
P49591	seryl-tRNA synthetase(SARS)						
P26639	threonyl-tRNA synthetase(TARS)						
P23381	tryptophanyl-tRNA synthetase(WARS)						
Q9Y2Z4	tyrosyl-tRNA synthetase 2(YARS2)		1,00				
P54577	tyrosyl-tRNA synthetase(YARS)						
P26640	valyl-tRNA synthetase(VARS)		1,00				
	Mean SKOV3/OVCAR3 Matching Score for aminoacyl-tRNA biosynthesis	0,00	0,25				
Protein translation							
P13639	eukaryotic translation elongation factor 2(EEF2)						
P41567	eukaryotic translation initiation factor 1(EIF1)						
P47813	eukaryotic translation initiation factor 1A, X-linked(EIF1AX)						
P05198	eukaryotic translation initiation factor 2 subunit alpha(EIF2S1)						
P20042	eukaryotic translation initiation factor 2 subunit beta(EIF2S2)						
P41091	eukaryotic translation initiation factor 2 subunit gamma(EIF2S3)						
Q14152	eukaryotic translation initiation factor 3 subunit A(EIF3A)		1,00				
Q04637	eukaryotic translation initiation factor 4 gamma 1(EIF4G1)		1,00				
P78344	eukaryotic translation initiation factor 4 gamma 2(EIF4G2)						
O43432	eukaryotic translation initiation factor 4 gamma 3(EIF4G3)						
P60842	eukaryotic translation initiation factor 4A1(EIF4A1)		1,00				

Q14240	eukaryotic translation initiation factor 4A2(EIF4A2)	1,00					
P06730	eukaryotic translation initiation factor 4E(EIF4E)						
P55010	eukaryotic translation initiation factor 5(EIF5)	1,00					
P56537	eukaryotic translation initiation factor 6(EIF6)						
	Mean SKOV3/OVCAR3 Matching Score for protein translation	0,13	0,20				
Ribosome							
Q7Z2Z2	elongation factor like GTPase 1(EFL1)						
P56537	eukaryotic translation initiation factor 6(EIF6)						
O14980	exportin 1(XPO1)		1,00				
P62861	FAU, ubiquitin like and ribosomal protein S30 fusion(FAU)		1,00				
Q9NVN8	G protein nucleolar 3 like(GNL3L)		1,00				
Q9NU22	midasin AAA ATPase 1(MDN1)		1,00				
Q9BYD6	mitochondrial ribosomal protein L1(MRPL1)						
Q9Y3B7	mitochondrial ribosomal protein L11(MRPL11)		1,00				
P52815	mitochondrial ribosomal protein L12(MRPL12)		1,00				
Q9BYD1	mitochondrial ribosomal protein L13(MRPL13)		1,00				
Q9P015	mitochondrial ribosomal protein L15(MRPL15)	1,00					
Q9NRX2	mitochondrial ribosomal protein L17(MRPL17)						
Q9H0U6	mitochondrial ribosomal protein L18(MRPL18)	1,00					
Q5T653	mitochondrial ribosomal protein L2(MRPL2)						
Q7Z2W9	mitochondrial ribosomal protein L21(MRPL21)		1,00				
Q9NWU5	mitochondrial ribosomal protein L22(MRPL22)		1,00				
Q96A35	mitochondrial ribosomal protein L24(MRPL24)						
Q9P0M9	mitochondrial ribosomal protein L27(MRPL27)						
Q13084	mitochondrial ribosomal protein L28(MRPL28)	1,00					
Q9BYD3	mitochondrial ribosomal protein L4(MRPL4)	1,00					
P82912	mitochondrial ribosomal protein S11(MRPS11)						
Q9BYD1	mitochondrial ribosomal protein L13(MRPL13)						
Q6P1L8	mitochondrial ribosomal protein L14(MRPL14)						
P82914	mitochondrial ribosomal protein S15(MRPS15)						
P49406	mitochondrial ribosomal protein L19(MRPL19)						
P09001	mitochondrial ribosomal protein L3(MRPL3)						
Q9BYC8	mitochondrial ribosomal protein L32(MRPL32)						
Q9BYD2	mitochondrial ribosomal protein L9(MRPL9)						
Q9Y2R5	mitochondrial ribosomal protein S17(MRPS17)						
Q9NVS2	mitochondrial ribosomal protein S18A(MRPS18A)						
Q9Y399	mitochondrial ribosomal protein S2(MRPS2)						
Q9Y2R9	mitochondrial ribosomal protein S7(MRPS7)						
P82933	mitochondrial ribosomal protein S9(MRPS9)	1,00					
Q9H0A0	N-acetyltransferase 10(NAT10)						
Q9NX24	NHP2 ribonucleoprotein(NHP2)						
Q9ULX3	NIN1/PSMD8 binding protein 1 homolog(NOB1)						
Q96D46	NMD3 ribosome export adaptor(NMD3)		1,00				
Q9UBU9	nuclear RNA export factor 1(NXF1)						
O95707	POP4 homolog, ribonuclease P/MRP subunit(POP4)						

P62826	RAN, member RAS oncogene family(RAN)						
P78346	ribonuclease P/MRP subunit p30(RPP30)						
P27635	ribosomal protein L10(RPL10)						
P62906	ribosomal protein L10a(RPL10A)		1,00				
P62913	ribosomal protein L11(RPL11)						
P30050	ribosomal protein L12(RPL12)		1,00				
P26373	ribosomal protein L13(RPL13)		1,00				
P40429	ribosomal protein L13a(RPL13A)	1,00	1,00				
P50914	ribosomal protein L14(RPL14)						
P61313	ribosomal protein L15(RPL15)		1,00				
P18621	ribosomal protein L17(RPL17)						
Q07020	ribosomal protein L18(RPL18)		1,00				
Q02543	ribosomal protein L18a(RPL18A)		1,00				
P84098	ribosomal protein L19(RPL19)						
P46778	ribosomal protein L21(RPL21)		1,00				
P62829	ribosomal protein L23(RPL23)		1,00				
P62750	ribosomal protein L23a(RPL23A)						
P83731	ribosomal protein L24(RPL24)		1,00				
P61254	ribosomal protein L26(RPL26)						
P61353	ribosomal protein L27(RPL27)	1,00					
P46776	ribosomal protein L27a(RPL27A)						
P62888	ribosomal protein L30(RPL30)		1,00				
P62899	ribosomal protein L31(RPL31)						
P42766	ribosomal protein L35(RPL35)						
P18077	ribosomal protein L35a(RPL35A)						
Q9Y3U8	ribosomal protein L36(RPL36)		1,00				
P61513	ribosomal protein L37a(RPL37A)						
P63173	ribosomal protein L38(RPL38)		1,00				
P36578	ribosomal protein L4(RPL4)						
Q02878	ribosomal protein L6(RPL6)						
P18124	ribosomal protein L7(RPL7)	1,00					
P62424	ribosomal protein L7a(RPL7A)						
P32969	ribosomal protein L9(RPL9)		1,00				
P05388	ribosomal protein lateral stalk subunit P0(RPLP0)						
P05386	ribosomal protein lateral stalk subunit P1(RPLP1)						
P05387	ribosomal protein lateral stalk subunit P2(RPLP2)						
P46783	ribosomal protein S10(RPS10)		1,00				
P62280	ribosomal protein S11(RPS11)		1,00				
P25398	ribosomal protein S12(RPS12)		1,00				
P62277	ribosomal protein S13(RPS13)		1,00				
P62263	ribosomal protein S14(RPS14)						
P62244	ribosomal protein S15a(RPS15A)		1,00				
P62249	ribosomal protein S16(RPS16)						
P08708	ribosomal protein S17(RPS17)						
P62269	ribosomal protein S18(RPS18)						

P39019	ribosomal protein S19(RPS19)		1,00				
P15880	ribosomal protein S2(RPS2)		1,00				
P60866	ribosomal protein S20(RPS20)		1,00				
P63220	ribosomal protein S21(RPS21)		1,00				
P62266	ribosomal protein S23(RPS23)		1,00				
P62847	ribosomal protein S24(RPS24)		1,00				
P62851	ribosomal protein S25(RPS25)						
P42677	ribosomal protein S27(RPS27)						
P62979	ribosomal protein S27a(RPS27A)		1,00				
P62857	ribosomal protein S28(RPS28)		1,00				
P23396	ribosomal protein S3(RPS3)		1,00				
P61247	ribosomal protein S3A(RPS3A)		1,00				
P62701	ribosomal protein S4, X-linked(RPS4X)		1,00				
Q8TD47	ribosomal protein S4, Y-linked 2(RPS4Y2)						
P46782	ribosomal protein S5(RPS5)						
P62753	ribosomal protein S6(RPS6)						
P62241	ribosomal protein S8(RPS8)		1,00				
P46781	ribosomal protein S9(RPS9)		1,00				
P08865	ribosomal protein SA(RPSA)		1,00				
Q9NW13	RNA binding motif protein 28(RBM28)						
Q9Y3B8	RNA exonuclease 2(REXO2)						
Q9Y3A5	SBDS ribosome assembly guanine nucleotide exchange factor(SBDS)						
P55769	SNU13 homolog, small nuclear ribonucleoprotein (U4/U6.U5)(SNU13)						
Q13428	treacle ribosome biogenesis factor 1(TCOF1)		1,00				
P62987	ubiquitin A-52 residue ribosomal protein fusion product 1(UBA52)						
Q9BVJ6	UTP14A small subunit processome component(UTP14A)						
Q9NYH9	UTP6, small subunit processome component(UTP6)						
	Mean SKOV3/OVCAR3 Matching Score for ribosome	0,07	0,40				
Proteasome							
P62191	proteasome 26S subunit, ATPase 1(PSMC1)						
P35998	proteasome 26S subunit, ATPase 2(PSMC2)						
P17980	proteasome 26S subunit, ATPase 3(PSMC3)						
P62195	proteasome 26S subunit, ATPase 5(PSMC5)						
P62333	proteasome 26S subunit, ATPase 6(PSMC6)						
Q99460	proteasome 26S subunit, non-ATPase 1(PSMD1)						
O00231	proteasome 26S subunit, non-ATPase 11(PSMD11)						
O00232	proteasome 26S subunit, non-ATPase 12(PSMD12)						
Q9UNM6	proteasome 26S subunit, non-ATPase 13(PSMD13)		1,00				
O00487	proteasome 26S subunit, non-ATPase 14(PSMD14)						
Q13200	proteasome 26S subunit, non-ATPase 2(PSMD2)		1,00				
P55036	proteasome 26S subunit, non-ATPase 4(PSMD4)						
Q15008	proteasome 26S subunit, non-ATPase 6(PSMD6)						
P51665	proteasome 26S subunit, non-ATPase 7(PSMD7)						
P48556	proteasome 26S subunit, non-ATPase 8(PSMD8)						
Q06323	proteasome activator subunit 1(PSME1)						

Q9UL46	proteasome activator subunit 2(PSME2)						
P61289	proteasome activator subunit 3(PSME3)						
Q9Y244	proteasome maturation protein(POMP)						
P25786	proteasome subunit alpha 1(PSMA1)						
P25789	proteasome subunit alpha 4(PSMA4)						
P28066	proteasome subunit alpha 5(PSMA5)						
P60900	proteasome subunit alpha 6(PSMA6)						
O14818	proteasome subunit alpha 7(PSMA7)						
P20618	proteasome subunit beta 1(PSMB1)						
P40306	proteasome subunit beta 10(PSMB10)						
P49720	proteasome subunit beta 3(PSMB3)						
P28072	proteasome subunit beta 6(PSMB6)						
Q99436	proteasome subunit beta 7(PSMB7)						
P28062	proteasome subunit beta 8(PSMB8)						
P28065	proteasome subunit beta 9(PSMB9)						
	Mean SKOV3/OVCAR3 Matching Score for proteasome	0,00	0,06				
	**Mean SKOV3/OVCAR3 Matching Score for protein expression**	**0,05**	**0,28**				
KINASE SIGNALING							
Receptor tyrosine kinase and MAPK signaling							
Q13085	acetyl-CoA carboxylase alpha(ACACA)		1,00				
O00763	acetyl-CoA carboxylase beta(ACACB)						
Q9UQM7	calcium/calmodulin dependent protein kinase II alpha(CAMK2A)						
Q13554	calcium/calmodulin dependent protein kinase II beta(CAMK2B)						
Q13555	calcium/calmodulin dependent protein kinase II gamma(CAMK2G)						
P62158	calmodulin 1(CALM1)		1,00				
Q9NZT1	calmodulin like 5(CALML5)						
P22681	Cbl proto-oncogene(CBL)						
O75131	copine 3(CPNE3)						
P46109	CRK like proto-oncogene, adaptor protein(CRKL)						
P00533	epidermal growth factor receptor(EGFR)						
P04626	erb-b2 receptor tyrosine kinase 2(ERBB2)						
P06730	eukaryotic translation initiation factor 4E(EIF4E)						
Q13541	eukaryotic translation initiation factor 4E binding protein 1(EIF4EBP1)						
Q9UPT5	exocyst complex component 7(EXOC7)		1,00				
P49327	fatty acid synthase(FASN)	1,00	1,00				
P62873	G protein subunit beta 1(GNB1)						
P13807	glycogen synthase 1(GYS1)						
Q5JWF2	GNAS complex locus(GNAS)		1,00				
P62993	growth factor receptor bound protein 2(GRB2)	1,00	1,00				
P52789	hexokinase 2(HK2)	1,00					
Q2TB90	hexokinase domain containing 1(HKDC1)						
O15357	inositol polyphosphate phosphatase like 1(INPPL1)						
P05556	integrin subunit beta 1(ITGB1)		1,00				
P42345	mechanistic target of rapamycin(MTOR)		1,00				

P28482	mitogen-activated protein kinase 1(MAPK1) = ERK-2	1,00	1,00
Q16539	mitogen-activated protein kinase 14(MAPK14)		
P27361	mitogen-activated protein kinase 3(MAPK3) = ERK-1		
P45984	mitogen-activated protein kinase 9(MAPK9)		
Q02750	mitogen-activated protein kinase kinase 1(MAP2K1) = MEK-1		
P49023	paxillin(PXN)		
P42338	phosphatidylinositol-4,5-bisphosphate 3-kinase catalytic subunit beta(PIK3CB)		
P27986	phosphoinositide-3-kinase regulatory subunit 1(PIK3R1)		
P19174	phospholipase C gamma 1(PLCG1)		
P06737	phosphorylase, glycogen, liver(PYGL)		
P11216	phosphorylase, glycogen; brain(PYGB)		
Q13131	protein kinase AMP-activated catalytic subunit alpha 1(PRKAA1)		
P10644	protein kinase cAMP-dependent type I regulatory subunit alpha(PRKAR1A)		
P13861	protein kinase cAMP-dependent type II regulatory subunit alpha(PRKAR2A)		
P62136	protein phosphatase 1 catalytic subunit alpha(PPP1CA)		
P62140	protein phosphatase 1 catalytic subunit beta(PPP1CB)		
P36873	protein phosphatase 1 catalytic subunit gamma(PPP1CC)		
P67775	protein phosphatase 2 catalytic subunit alpha(PPP2CA)		
Q05397	protein tyrosine kinase 2(PTK2) = FAK1		
P18031	protein tyrosine phosphatase, non-receptor type 1(PTPN1)		
Q06124	protein tyrosine phosphatase, non-receptor type 11(PTPN11) = SHP-2		1,00
P62834	RAP1A, member of RAS oncogene family(RAP1A)		
P61224	RAP1B, member of RAS oncogene family(RAP1B)		1,00
P63244	receptor for activated C kinase 1(RACK1)		1,00
Q15418	ribosomal protein S6 kinase A1(RPS6KA1)		
P62753	ribosomal protein S6(RPS6)		
Q13501	sequestosome 1(SQSTM1)	1,00	
P29353	SHC adaptor protein 1(SHC1)		
P40763	signal transducer and activator of transcription 3(STAT3)		1,00
P12931	SRC proto-oncogene, non-receptor tyrosine kinase(SRC)		
O75312	ZPR1 zinc finger(ZPR1)		
	Mean SKOV3/OVCAR3 Matching Score for receptor tyrosine kinase and MAPK signaling	0,09	0,23
mTOR signaling			
P31749	AKT serine/threonine kinase 1(AKT1)		
Q96B36	AKT1 substrate 1(AKT1S1)		
Q14152	eukaryotic translation initiation factor 3 subunit A(EIF3A)		1,00
Q04637	eukaryotic translation initiation factor 4 gamma 1(EIF4G1)		1,00
P78344	eukaryotic translation initiation factor 4 gamma 2(EIF4G2)		
O43432	eukaryotic translation initiation factor 4 gamma 3(EIF4G3)		
P60842	eukaryotic translation initiation factor 4A1(EIF4A1)		1,00
Q14240	eukaryotic translation initiation factor 4A2(EIF4A2)		
P23588	eukaryotic translation initiation factor 4B(EIF4B)	1,00	1,00
P06730	eukaryotic translation initiation factor 4E(EIF4E)		
P62942	FK506 binding protein 1A(FKBP1A)		
P42345	mechanistic target of rapamycin(MTOR)		1,00

P27986	phosphoinositide-3-kinase regulatory subunit 1(PIK3R1)						
P67775	protein phosphatase 2 catalytic subunit alpha(PPP2CA)						
Q15382	Ras homolog enriched in brain(RHEB)						
P62753	ribosomal protein S6(RPS6)						
P51812	ribosomal protein S6 kinase A3(RPS6KA3)						
	Mean SKOV3/OVCAR3 Matching Score for mTOR signaling	0,06	0,29				
Cell cycle							
P00519	ABL proto-oncogene 1, non-receptor tyrosine kinase(ABL1)						
Q9UM13	anaphase promoting complex subunit 10(ANAPC10)						
Q9UJX6	anaphase promoting complex subunit 2(ANAPC2)						
Q9UJX4	anaphase promoting complex subunit 5(ANAPC5)						
Q9UJX3	anaphase promoting complex subunit 7(ANAPC7)						
Q13535	ATR serine/threonine kinase(ATR)						
O43684	BUB3, mitotic checkpoint protein(BUB3)						
Q9UJX2	cell division cycle 23(CDC23)		1,00				
P30260	cell division cycle 27(CDC27)						
Q92793	CREB binding protein(CREBBP)						
Q13616	cullin 1(CUL1)		1,00				
P14635	cyclin B1(CCNB1)		1,00				
O95067	cyclin B2(CCNB2)						
P51946	cyclin H(CCNH)						
P06493	cyclin dependent kinase 1(CDK1)		1,00				
P11802	cyclin dependent kinase 4(CDK4)						
Q00534	cyclin dependent kinase 6(CDK6)						
P50613	cyclin dependent kinase 7(CDK7)						
Q16254	E2F transcription factor 4(E2F4)						
Q9UM11	fizzy/cell division cycle 20 related 1(FZR1)						
Q13547	histone deacetylase 1(HDAC1)						
Q92769	histone deacetylase 2(HDAC2)						
P49736	minichromosome maintenance complex component 2(MCM2)						
P25205	minichromosome maintenance complex component 3(MCM3)		1,00				
P33991	minichromosome maintenance complex component 4(MCM4)	1,00	1,00				
P33992	minichromosome maintenance complex component 5(MCM5)						
Q14566	minichromosome maintenance complex component 6(MCM6)						
P33993	minichromosome maintenance complex component 7(MCM7)		1,00				
P53350	polo like kinase 1(PLK1)		1,00				
P12004	proliferating cell nuclear antigen(PCNA)		1,00				
P06400	RB transcriptional corepressor 1(RB1)						
P62877	ring-box 1(RBX1)						
P63208	S-phase kinase associated protein 1(SKP1)						
Q14683	structural maintenance of chromosomes 1A(SMC1A)						
Q9UQE7	structural maintenance of chromosomes 3(SMC3)		1,00				
P04637	tumor protein p53(TP53)						
P31946	tyrosine 3-monooxygenase/tryptophan 5-monooxygenase activation protein beta(YWHAB)						
Q04917	tyrosine 3-monooxygenase/tryptophan 5-monooxygenase activation protein eta(YWHAH)						

P61981	tyrosine 3-monooxygenase/tryptophan 5-monooxygenase activation protein gamma(YWHAG)		
P27348	tyrosine 3-monooxygenase/tryptophan 5-monooxygenase activation protein theta(YWHAQ)		
P63104	tyrosine 3-monooxygenase/tryptophan 5-monooxygenase activation protein zeta(YWHAZ)		
	Mean SKOV3/OVCAR3 Matching Score for cell cycle	0,02	0,24
	**Mean SKOV3/OVCAR3 Matching Score for kinase signaling**	**0,06**	**0,25**
MEMBRANE INTEGRITY, MOLECULAR UPTAKE/TRANSPORT			
Intracellular protein transport pathways			
O43707	actinin alpha 4(ACTN4)		
Q9BXS5	adaptor related protein complex 1 mu 1 subunit(AP1M1)		1,00
Q8N6T3	ADP ribosylation factor GTPase activating protein 1(ARFGAP1)		
Q8N6H7	ADP ribosylation factor GTPase activating protein 2(ARFGAP2)		
O43823	A-kinase anchoring protein 8(AKAP8)		1,00
P48444	archain 1(ARCN1)	1,00	1,00
Q9UPQ3	ArfGAP with GTPase domain, ankyrin repeat and PH domain 1(AGAP1)		
P49407	arrestin beta 1(ARRB1)		
P49454	centromere protein F(CENPF)		1,00
Q92526	chaperonin containing TCP1 subunit 6B(CCT6B)		
Q96FZ7	charged multivesicular body protein 6(CHMP6)		
Q8WUX9	charged multivesicular body protein 7(CHMP7)		
O14579	coatomer protein complex subunit epsilon(COPE)		1,00
P61923	coatomer protein complex subunit zeta 1(COPZ1)		
Q8WTW3	component of oligomeric golgi complex 1(COG1)		
Q9NUU7	DEAD-box helicase 19A(DDX19A)		
Q9NPA8	ENY2, transcription and export complex 2 subunit(ENY2)		
P42566	epidermal growth factor receptor pathway substrate 15(EPS15)		
Q9H8V3	epithelial cell transforming 2(ECT2)		1,00
Q9Y2D4	exocyst complex component 6B(EXOC6B)		
Q9UPT5	exocyst complex component 7(EXOC7)		1,00
Q8IYI6	exocyst complex component 8(EXOC8)		1,00
Q641Q2	family with sequence similarity 21 member A(FAM21A)		
P60520	GABA type A receptor associated protein like 2(GABARAPL2)		
Q9HD26	golgi associated PDZ and coiled-coil motif containing(GOPC)		1,00
Q86VS8	hook microtubule tethering protein 3(HOOK3)		
Q8TEX9	importin 4(IPO4)	1,00	1,00
P53990	IST1, ESCRT-III associated factor(IST1)		
Q12768	KIAA0196(KIAA0196)		
Q86UP2	kinectin 1(KTN1)		
Q8NI77	kinesin family member 18A(KIF18A)		
P49257	lectin, mannose binding 1(LMAN1)		1,00
Q14764	major vault protein(MVP)	1,00	
Q5JRA6	MIA family member 3, ER export factor(MIA3)		
P35579	myosin heavy chain 9(MYH9)		1,00
Q13496	myotubularin 1(MTM1)		
E9PAV3	nascent polypeptide-associated complex alpha subunit(NACA)		

Q9NVZ3	NECAP endocytosis associated 2(NECAP2)						
Q96D46	NMD3 ribosome export adaptor(NMD3)		1,00				
P49321	nuclear autoantigenic sperm protein(NASP)						
Q12769	nucleoporin 160(NUP160)		1,00				
Q8TEM1	nucleoporin 210(NUP210)						
Q8NFH3	nucleoporin 43(NUP43)	1,00	1,00				
Q08752	peptidylprolyl isomerase D(PPID)						
O00562	phosphatidylinositol transfer protein membrane associated 1(PITPNM1)						
Q9H814	phosphorylated adaptor for RNA export(PHAX)						
Q8WUM4	programmed cell death 6 interacting protein(PDCD6IP)						
Q6WKZ4	RAB11 family interacting protein 1(RAB11FIP1)						
P62491	RAB11A, member RAS oncogene family(RAB11A)						
Q15907	RAB11B, member RAS oncogene family(RAB11B)						
Q9ULC3	RAB23, member RAS oncogene family(RAB23)						
P51148	RAB5C, member RAS oncogene family(RAB5C)						
Q9NRW1	RAB6B, member RAS oncogene family(RAB6B)						
P51149	RAB7A, member RAS oncogene family(RAB7A)						
Q9H5N1	rabaptin, RAB GTPase binding effector protein 2(RABEP2)						
P62834	RAP1A, member of RAS oncogene family(RAP1A)						
Q9P2E9	ribosome binding protein 1(RRBP1)						
Q96T51	RUN and FYVE domain containing 1(RUFY1)						
Q8WVM8	sec1 family domain containing 1(SCFD1)						
O15027	SEC16 homolog A, endoplasmic reticulum export factor(SEC16A)		1,00				
O94979	SEC31 homolog A, COPII coat complex component(SEC31A)						
Q96EE3	SEH1 like nucleoporin(SEH1L)		1,00				
Q9UMY4	sorting nexin 12(SNX12)						
O60493	sorting nexin 3(SNX3)						
Q99598	translin associated factor X(TSNAX)						
Q9Y5J7	translocase of inner mitochondrial membrane 9(TIMM9)						
Q13445	transmembrane p24 trafficking protein 1(TMED1)						
Q9BVK6	transmembrane p24 trafficking protein 9(TMED9)						
Q9NZ09	ubiquitin associated protein 1(UBAP1)						
O75351	vacuolar protein sorting 4 homolog B(VPS4B)		1,00				
O75436	VPS26, retromer complex component A(VPS26A)						
Q9UBQ0	VPS29, retromer complex component(VPS29)						
Q9H267	VPS33B, late endosome and lysosome associated(VPS33B)						
Q96QK1	VPS35, retromer complex component(VPS35)						
O43264	zw10 kinetochore protein(ZW10)						
	Mean SKOV3/OVCAR3 Matching Score for intracellular protein transport pathways	0,05	0,24				
Solute carrier family proteins							
P12235	ADP/ATP translocase 1 (SLC25A4)		1,00				
P05141	ADP/ATP translocase 2 (SLC25A5)		1,00				
P12236	ADP/ATP translocase 3 (SLC25A6)		1,00				
Q9UJS0	Calcium-binding mitochondrial carrier protein Aralar2 (SLC25A13)		1,00				
Q6NUK1	Calcium-binding mitochondrial carrier protein SCaMC-1 (SLC25A24)						

Q9BWU0	Kanadaptin (SLC4A1AP)		
Q02978	Mitochondrial 2-oxoglutarate/malate carrier protein (SLC25A11)		1,00
Q9H936	Mitochondrial glutamate carrier 1 (SLC25A22)		1,00
O14745	Na(+)/H(+) exchange regulatory cofactor NHE-RF1 (SLC9A3R1)		
Q15599	Na(+)/H(+) exchange regulatory cofactor NHE-RF2 (SLC9A3R2)		
Q15758	Neutral amino acid transporter B(0) (SLC1A5)		
P55011	Solute carrier family 12 member 2		
P11166	solute carrier family 2 member 1(SLC2A1)		
Q9Y226	Solute carrier family 22 member 13 (SLC22A13)		
P53007	Tricarboxylate transport protein, mitochondrial (SLC25A1)		1,00
Q9ULF5	Zinc transporter ZIP10 (SLC39A10)		
	Mean SKOV3/OVCAR3 Matching Score for solute carrier family proteins	0,00	0,44
Transport vesicles/endosomal transport			
Q12904	aminoacyl tRNA synthetase complex interacting multifunctional protein 1(AIMP1)		
P48444	archain 1(ARCN1)	1,00	1,00
Q6QNY0	biogenesis of lysosomal organelles complex 1 subunit 3(BLOC1S3)		
Q8TDH9	biogenesis of lysosomal organelles complex 1 subunit 5(BLOC1S5)		
Q99653	calcineurin like EF-hand protein 1(CHP1)		
Q9BY43	charged multivesicular body protein 4A(CHMP4A)		
Q9H444	charged multivesicular body protein 4B(CHMP4B)		
Q96FZ7	charged multivesicular body protein 6(CHMP6)		
Q8WUX9	charged multivesicular body protein 7(CHMP7)		
P53621	coatomer protein complex subunit alpha(COPA)		
P53618	coatomer protein complex subunit beta 1(COPB1)		
P35606	coatomer protein complex subunit beta 2(COPB2)		1,00
O14579	coatomer protein complex subunit epsilon(COPE)		
Q9Y678	coatomer protein complex subunit gamma 1(COPG1)		
P61923	coatomer protein complex subunit zeta 1(COPZ1)		
P30040	endoplasmic reticulum protein 29(ERP29)		1,00
O00461	golgi integral membrane protein 4(GOLIM4)		
O14964	hepatocyte growth factor-regulated tyrosine kinase substrate(HGS)		1,00
P11717	insulin like growth factor 2 receptor(IGF2R)		1,00
Q12768	KIAA0196(KIAA0196)		
O60664	perilipin 3(PLIN3)		
P62979	ribosomal protein S27a(RPS27A)		1,00
Q13501	sequestosome 1(SQSTM1)	1,00	
O75886	signal transducing adaptor molecule 2(STAM2)		
Q92783	signal transducing adaptor molecule(STAM)		
Q8WV41	sorting nexin 33(SNX33)		
Q96L92	sorting nexin family member 27(SNX27)		
P49755	transmembrane p24 trafficking protein 10(TMED10)		
Q9BVK6	transmembrane p24 trafficking protein 9(TMED9)		
O60763	USO1 vesicle transport factor(USO1)		
O75351	vacuolar protein sorting 4 homolog B(VPS4B)		1,00
Q9UK41	VPS28, ESCRT-I subunit(VPS28)		

Q7Z3T8	zinc finger FYVE-type containing 16(ZFYVE16)						
	Mean SKOV3/OVCAR3 Matching Score for transport vesicles/endosomal transport	0,06	0,21				
Nuclear pore complex							
P57740	nucleoporin 107(NUP107)		1,00				
P49790	nucleoporin 153(NUP153)						
O75694	nucleoporin 155(NUP155)						
Q12769	nucleoporin 160(NUP160)		1,00				
Q8TEM1	nucleoporin 210(NUP210)						
P35658	nucleoporin 214(NUP214)						
Q8NFH5	nucleoporin 35(NUP35)						
Q8NFH3	nucleoporin 43(NUP43)		1,00				
Q9BVL2	nucleoporin 58(NUP58)		1,00				
Q8N1F7	nucleoporin 93(NUP93)		1,00				
P52948	nucleoporin 98(NUP98)		1,00				
Q96EE3	SEH1 like nucleoporin(SEH1L)		1,00				
P12270	translocated promoter region, nuclear basket protein(TPR)						
	Mean SKOV3/OVCAR3 Matching Score for nuclear pore complex	0,00	0,54				
	**Mean SKOV3/OVCAR3 Matching Score for membrane integrity, molecular uptake/transport**	**0,04**	**0,28**				
STRESS PATHWAYS							
HIF signaling							
P31749	AKT serine/threonine kinase 1(AKT1)						
P27540	aryl hydrocarbon receptor nuclear translocator(ARNT)						
Q92793	CREB binding protein(CREBBP)		1,00				
Q9GZT9	egl-9 family hypoxia inducible factor 1(EGLN1)						
P13929	enolase 3(ENO3)						
P04626	erb-b2 receptor tyrosine kinase 2(ERBB2)						
P06730	eukaryotic translation initiation factor 4E(EIF4E)						
P52789	hexokinase 2(HK2)	1,00					
Q2TB90	hexokinase domain containing 1(HKDC1)						
Q16665	hypoxia inducible factor 1 alpha subunit(HIF1A)						
P42345	mechanistic target of rapamycin(MTOR)		1,00				
P28482	mitogen-activated protein kinase 1(MAPK1) = ERK-2	1,00	1,00				
P27361	mitogen-activated protein kinase 3(MAPK3) = ERK-1						
Q02750	mitogen-activated protein kinase kinase 1(MAP2K1) = MEK-1						
P19838	nuclear factor kappa B subunit 1(NFKB1)						
P42338	phosphatidylinositol-4,5-bisphosphate 3-kinase catalytic subunit beta(PIK3CB)						
P27986	phosphoinositide-3-kinase regulatory subunit 1(PIK3R1)						
P19174	phospholipase C gamma 1(PLCG1)						
P16885	phospholipase C gamma 2(PLCG2)						
P11177	pyruvate dehydrogenase (lipoamide) beta(PDHB)						
Q04206	RELA proto-oncogene, NF-kB subunit(RELA)						
P62753	ribosomal protein S6(RPS6)		1,00				
P62877	ring-box 1(RBX1)						
P40763	signal transducer and activator of transcription 3(STAT3)		1,00				

Q15369	transcription elongation factor B subunit 1(TCEB1)						
Q15370	transcription elongation factor B subunit 2(TCEB2)						
	Mean SKOV3/OVCAR3 Matching Score for HIF signaling	0,08	0,19				
Processing ER							
O95816	BCL2 associated athanogene 2(BAG2)						
P27824	calnexin(CANX)						
P17655	calpain 2(CAPN2)		1,00				
P27797	calreticulin(CALR)						
Q13616	cullin 1(CUL1)		1,00				
Q07065	cytoskeleton associated protein 4(CKAP4)						
P31689	DnaJ heat shock protein family (Hsp40) member A1(DNAJA1)						
P25685	DnaJ heat shock protein family (Hsp40) member B1(DNAJB1)						
Q9UBS4	DnaJ heat shock protein family (Hsp40) member B11(DNAJB11)						
Q9NXW2	DnaJ heat shock protein family (Hsp40) member B12(DNAJB12)						
Q13217	DnaJ heat shock protein family (Hsp40) member C3(DNAJC3)						
P39656	dolichyl-diphosphooligosaccharide--protein glycosyltransferase non-catalytic subunit(DDOST)		1,00				
Q96DZ1	endoplasmic reticulum lectin 1(ERLEC1)						
Q96HE7	endoplasmic reticulum oxidoreductase 1 alpha(ERO1A)						
P30040	endoplasmic reticulum protein 29(ERP29)		1,00				
P05198	eukaryotic translation initiation factor 2 subunit alpha(EIF2S1)						
Q14697	glucosidase II alpha subunit(GANAB)						
P07900	heat shock protein 90 alpha family class A member 1(HSP90AA1)						
P08238	heat shock protein 90 alpha family class B member 1(HSP90AB1)	1,00					
P08107	heat shock protein family A (Hsp70) member 1A(HSPA1A)						
P54652	heat shock protein family A (Hsp70) member 2(HSPA2)						
O95757	heat shock protein family A (Hsp70) member 4 like(HSPA4L)						
P11021	heat shock protein family A (Hsp70) member 5(HSPA5)		1,00				
P17066	heat shock protein family A (Hsp70) member 6(HSPA6)						
P11142	heat shock protein family A (Hsp70) member 8(HSPA8)	1,00	1,00				
Q92598	heat shock protein family H (Hsp110) member 1(HSPH1)						
P49257	lectin, mannose binding 1(LMAN1)		1,00				
Q13724	mannosyl-oligosaccharide glucosidase(MOGS)						
P45984	mitogen-activated protein kinase 9(MAPK9)						
Q8TAT6	NPL4 homolog, ubiquitin recognition factor(NPLOC4)		1,00				
Q9UNZ2	NSFL1 cofactor(NSFL1C)						
P07237	prolyl 4-hydroxylase subunit beta(P4HB)						
P30101	protein disulfide isomerase family A member 3(PDIA3)						
P13667	protein disulfide isomerase family A member 4(PDIA4)						
Q15084	protein disulfide isomerase family A member 6(PDIA6)		1,00				
P14314	protein kinase C substrate 80K-H(PRKCSH)						
P54727	RAD23 homolog B, nucleotide excision repair protein(RAD23B)						
P04843	ribophorin I(RPN1)		1,00				
P04844	ribophorin II(RPN2)		1,00				
Q9P2E9	ribosome binding protein 1(RRBP1)						
P62877	ring-box 1(RBX1)						

P55735	SEC13 homolog, nuclear pore and COPII coat complex component(SEC13)						
Q15436	Sec23 homolog A, coat complex II component(SEC23A)						
O95487	SEC24 homolog B, COPII coat complex component(SEC24B)						
P53992	SEC24 homolog C, COPII coat complex component(SEC24C)	1,00	1,00				
O94979	SEC31 homolog A, COPII coat complex component(SEC31A)						
Q9UGP8	SEC63 homolog, protein translocation regulator(SEC63)	1,00					
P43307	signal sequence receptor subunit 1(SSR1)						
P51571	signal sequence receptor subunit 4(SSR4)		1,00				
P63208	S-phase kinase associated protein 1(SKP1)						
Q8NBS9	thioredoxin domain containing 5(TXNDC5)						
Q9UHD9	ubiquilin 2(UBQLN2)						
Q92890	ubiquitin fusion degradation 1 like (yeast)(UFD1L)						
O95155	ubiquitination factor E4B(UBE4B)						
Q9BZV1	UBX domain protein 6(UBXN6)						
Q9NYU2	UDP-glucose glycoprotein glucosyltransferase 1(UGGT1)						
P55072	valosin containing protein(VCP)						
	Mean SKOV3/OVCAR3 Matching Score for processing ER	0,07	0,23				
	**Mean SKOV3/OVCAR3 Matching Score for stress pathways**	**0,07**	**0,22**				
APOPTOSIS							
O95831	Apoptosis-inducing factor 1, mitochondrial(AIFM1)						
Q9UKV3	Apoptotic chromatin condensation inducer in the nucleus(ACIN1)		1,00				
Q9H8G2	Caspase activity and apoptosis inhibitor 1(CAAP1)						
Q8IX12	Cell division cycle and apoptosis regulator protein 1(CCAR1)		1,00				
Q9UER7	Death domain-associated protein 6(DAAX)						
P53355	Death-associated protein kinase 1(DAPK1)		1,00				
Q9BTC0	Death-inducer obliterator 1(DIDO1)						
Q8WUM4	Programmed cell death 6-interacting protein(PDCD6IP)						
Q9BUL8	Programmed cell death protein 10(PDCD10)		1,00				
Q53EL6	Programmed cell death protein 4(PDCD4)						
O14737	Programmed cell death protein 5(PDCD5)		1,00				
	**Mean SKOV3/OVCAR3 Matching Score for apoptosis**	**0,00**	**0,45**				
	**Legend**						
	Significantly ($p < 0.05$) downregulated proteins: Blue cell						
	Significantly ($p < 0.05$) upregulated proteins: Red cell						
	**For Individual Proteins**						
	SKOV3/OVCAR3 Matching Score:						
	1,00: uniform regulation at the same time in both cell lines.						
	0,00: no uniform regulation in both cell lines at the specific time.						
	**For Key Cell Processes or Sub-Processes**						
	Mean SKOV3/OVCAR3 Matching Score:						
	Mean of 'Individual Protein SKOV3/OVCAR3 Matching Scores'						

Supplemental Table S4a: Summary of all down- (< 50 % of Control) and up-regulated (> 150 % of Control) phosphoproteins as determined by antibody microarray kinomic analysis in SKOV3 cells exposed for 24h to 40µM G28UCM.

Target Name	Full Target Protein Name	P-Site	UniProt #	% of Untreated Control
**I. Down-regulated Phosphoproteins (< 50% of Control)**				
GFAP	Glial fibrillary acidic protein	S8	P14136	3
Csk	C-terminus of Src tyrosine kinase	Y184	P41240	3
ATF2 (CRE-BP1)	Cyclic AMP-dependent transcription factor ATF-2	S112	P15336	6
CFL1	Cofilin 1	S3	P23528	6
B-Myb (MYBL2)	Myb-related protein B	T487	P10244	7
CK2a1 (CSNK2A1)	Casein protein-serine kinase 2 alpha/ alpha prime	T360+S362	P68400	8
ENFB2	Ephrin-B2	Y316	P52799	8
CDK5	Cyclin-dependent protein-serine kinase 5	S159	Q00535	12
UBF	Nucleolar transcription factor 1	S484	P17480	15
CFL1	Cofilin 1	S3	P23528	16
CrkL	Crk-like protein	Y251	P46109	16
CaMKK1 (CaMKK)	Calcium/calmodulin-dependent protein-serine kinase kinase	S74	Q8N5S9	19
Cbl	E3 ubiquitin-protein ligase CBL	Y700	P22681	25
HDAC4/5/9	Histone deacetylase 4	S246/259/220	P56524	27
Huntingtin	Huntington's disease protein	S419	P42858	28
eEF2K	Eukaryotic elongation factor 2 kinase	S366	O00418	28
CTNNB1	Catenin (cadherin-associated protein) beta 1	Y333	P35222	28
H2AFX	Histone H2A.X	S139	P16104	29
CTNNB1	Catenin (cadherin-associated protein) beta 1	S33+S37	P35222	30
SNCA (a-Synuclein)	Alpha-synuclein	S129	P37840	31
CDK1 (CDC2)	Cyclin-dependent protein-serine kinase 1	T161	P06493	32
DDR1	Epithelial discoidin domain-containing receptor 1	Y796+Y797	Q08345	32
PLCE1	1-phosphatidylinositol-4,5-bisphosphate phosphodiesterase epsilon-1	S1096+T1100	Q9P212	33
Jun (c-Jun)	Jun proto-oncogene-encoded AP1 transcription factor	T91+T93	P05412	34
CLK1	Dual specificity protein kinase CLK1	S337	P49759	34
DYRK1A	Dual specificity tyrosine-phosphorylation-regulated kinase 1A	Y321	Q13627	35
MEK1 (MKK1, MAP2K1)	MAPK/ERK protein-serine kinase 1 (MKK1)	S222	Q02750	35
CSF1R (Fms)	Macrophage colony-stimulating factor 1 receptor	Y809	P07333	36
CTNNA1	Catenin alpha-1	S641	P35221	36
Abl (Abl1)	Abelson murine leukemia viral oncogene homologue 1	Y413	P00519	36
EGFR (ErbB1)	Epidermal growth factor receptor-tyrosine kinase	Y1110	P00533	37
MDM2	E3 ubiquitin-protein ligase double minute 2 protein	S166	Q00987	37
Caveolin 1	Caveolin 1	Y14	Q03135	38

Gab1	GRB2-associated-binding protein 1	Y627	Q13480	38
ROCK2 (ROKa)	Rho-associated protein kinase 2	Y722	O75116	39
EGFR (ErbB1)	Epidermal growth factor receptor-tyrosine kinase	Y1172	P00533	39
Connexin 43	Gap junction alpha-1 protein	S368	P17302	40
NMDAR2B (GRIN2B)	Glutamate [NMDA] receptor subunit epsilon-2	Y1474	Q13224	40
VIM	Vimentin	S34	P08670	41
ERK1 (MAPK3)	Extracellular regulated protein-serine kinase 1 (p44 MAP kinase)	Y204	P27361	42
DLK (ZPK)	Mitogen-activated protein kinase kinase kinase 12	S269	Q12852	43
CSF1R (Fms)	Macrophage colony-stimulating factor 1 receptor	Y699	P07333	43
COX2	Prostaglandin G/H synthase 2	Y446	P35354	44
HePTP (PTPN7)	Tyrosine-protein phosphatase non-receptor type 7	S44	P35236	47
Cbl	E3 ubiquitin-protein ligase CBL	Y674	P22681	47
CTNNB1	Catenin (cadherin-associated protein) beta 1	S33	P35222	48
ERK1 (MAPK3)	Extracellular regulated protein-serine kinase 1 (p44 MAP kinase)	Y204	P27361	48
SIK (SNF1LK)	Salt-inducible serine/threonine-protein kinase SIK1	T182	P57059	49
CDK5	Cyclin-dependent protein-serine kinase 5	Y15	Q00535	49
HDAC5	Histone deacetylase 5	S498	Q9UQL6	49
Dynamin I	Dynamin-1	S795	Q05193	49
**II. Up-regulated Phosphoproteins (> 150% of Control)**				
p53 (TP53)	Cellular tumor antigen p53	S392	P04637	152
Fyn	Fyn proto-oncogene-encoded protein-tyrosine kinase	T12	P06241	152
PCYT1A (CTPCT; CCTA)	CTP: phosphocholine cytidylyltransferase isoform A	Y359+S362	P49585	152
Src	Src oncogene-encoded protein-tyrosine kinase	Y530	P12931	153
FAK (PTK2)	Focal adhesion protein-tyrosine kinase	Y397	Q05397	153
NFKB1 (NFkB-p105)	Nuclear factor NF-kappa-B p105 subunit	S932	P19838	154
SMC1A	Structural maintenance of chromosomes protein 1A	S957	Q14683	155
SMARCB1	SWI/SNF-rel. matrix-assoc. actin-dep. Regul. of chromatin subf.B memb.1	T134	Q12824	160
SLK	STE20-like serine/threonine-protein kinase; CTCL tumour antigen se20-9	S189	Q9H2G2	160
PKD1 (PRKCM, PKCm, PRKD1)	Protein-serine kinase C mu (Protein kinase D)	S910	Q15139	160
PDGFRb	Platelet-derived growth factor receptor kinase beta	Y716	P09619	162
Syk	Spleen protein-tyrosine kinase	Y352	P43405	162
PKCt (PRKCQ)	Protein-serine kinase C theta	T538	Q04759	164
PKCg (PRKCG)	Protein-serine kinase C gamma	T655	P05129	165
Src	Src oncogene-encoded protein-tyrosine kinase	Y419	P12931	168
PTEN	Protein phosphatase and tensin homolog deleted on chromosome 10	S380+T382+T383	P60484	168
PKCz (PRKCZ)	Protein-serine kinase C zeta	T410	Q05513	170
TrkB (NTRK2)	BNDF/NT3/4/5 receptor- tyrosine kinase	Y702	Q16620	171
Src	Src oncogene-encoded protein-tyrosine kinase	Y419	P12931	172
Cip (WAF1; p21) p21(WAF1/CIP1)	Cyclin-dependent kinase inhibitor 1	T145	P38936	172
Syk	Spleen protein-tyrosine kinase	Y525+Y526	P43405	173
TRIM28 (TIF1B)	Transcription intermediary factor 1-beta	Y517	Q13263	174

PKD1 (PRKCM, PKCm, PRKD1)	Protein-serine kinase C mu (Protein kinase D)	S738+S742	Q15139	176
p53 (TP53)	Cellular tumor antigen p53	S6	P04637	180
ILK1 (ILK)	Integrin-linked protein-serine kinase-1	Y351	Q13418	180
THRAP3	Thyroid hormone receptor-associated protein 3	S253	Q9Y2W1	181
SRF	Serum response factor	S224	P11831	183
PKCd (PRKCD)	Protein-serine kinase C delta	S645	Q05655	185
Lck	Lymphocyte-specific protein-tyrosine kinase	Y394	P06239	195
p53 (TP53)	Cellular tumor antigen p53	T155	P04637	200
p70S6K (S6Ka, RPS6KB1)	Ribosomal protein S6 kinase beta-1; Ribosomal protein S6 kinase 1	S434	P23443	206
hnRNP-K	Heterogeneous nuclear ribonucleoprotein K	S302	P61978	208
p53 (TP53)	Cellular tumor antigen p53	T55	P04637	212
PKCa (PRKCA)	Protein-serine kinase C alpha	Y195	P17252	214
Tau	Microtubule-associated protein tau	S713	P10636	222
Caveolin 2	Caveolin 2	S36	P51636	239
PKCd (PRKCD)	Protein-serine kinase C delta	Y334	Q05655	242
MEK1 (MKK1, MAP2K1)	MAPK/ERK protein-serine kinase 1 (MKK1)	S222	Q02750	243
ASK1 (MAP3K5)	Apoptosis signal regulating protein-serine kinase 1	S966	Q99683	243
Adducin a/g (ADD3)	Adducin-alpha/gamma	S726	Q9UEY8	278
Cip (WAF1; p21) p21(WAF1/CIP1)	Cyclin-dependent kinase inhibitor 1	S146	P38936	279
ErbB4 (HER4)	ErbB4 receptor-tyrosine kinase	Y875	Q15303	286
IkBa	Inhibitor of NF-kappa-B alpha (MAD3)	S32	P25963	293
eIF4E	Eukaryotic translation initiation factor 4 (mRNA cap binding protein)	S209	P06730	310
p53 (TP53)	Cellular tumor antigen p53	S46	P04637	322
PRKAR2A (PKA2RA)	cAMP-dependent protein kinase type II-alpha regulatory subunit	S99	P13861	353
p40-phox	Neutrophil cytosol factor 4	T154	Q15080	418
Smad2	Mothers against decapentaplegic homologue 2	T220	Q15796	529
PKCd (PRKCD)	Protein-serine kinase C delta	S664	Q05655	610
Tau	Microtubule-associated protein tau	T522	P10636	690
RSK1 (RPS6KA1, p90RSK)	Ribosomal S6 protein-serine kinase 1	T348	Q15418	1252

Supplemental Table S4b: Summary of all down- (< 50 % of Control) and up-regulated (> 150 % of Control) phosphoproteins as determined by antibody microarray kinomic analysis in OVCAR3 cells exposed for 24h to 40µM G28UCM.

Target Name	Full Target Protein Name	P-Site	UniProt #	% of Untreated Control
**I. Down-regulated Phosphoproteins (< 50% of Control)**				
eIF4B	Eukaryotic translation initiation factor 4B	S422	P23588	3
JUN	Jun proto-oncogene-encoded AP1 transcription factor (c-Jun)	Y170	P05412	8
Smad1	Mothers against decapentaplegic homologs 1	S465	Q15797	9
MYC	Myc proto-oncogene protein	T58	P01106	10
Tau	Microtubule-associated protein tau	S713	P10636	12
p53	Tumor suppressor protein p53 (antigenNY-CO-13) (TP53)	S392	P04637	15
MEK2	MAPK/ERK protein-serine kinase 2 (MKK2, MAP2K2)	T394	P36507	15
NFkappaB p65	NF-kappa-B p65 nuclear transcription factor (Rel A)	S276	Q04206	16
TRKB	BNDF/NT3/4/5 receptor- tyrosine kinase (NTRK2)	Y706	Q16620	16
CHK2	Checkpoint protein-serine kinase 2 (CHEK2)	T68	O96017	17
LCK	Lymphocyte-specific protein-tyrosine kinase	Y505	P06239	20
FES	Fes/Fps protein-tyrosine kinase	Y713	P07332	22
IRS1	Insulin receptor substrate 1	Y612	P35568	27
JNK1	Jun N-terminus protein-serine kinase (stress-activated protein kinase (SAPK)) 1 (SAPKg, MA	Y185	P45983	29
PKCd	Protein-serine kinase C delta (PRKCD)	S645	Q05655	29
p53	Tumor suppressor protein p53 (antigenNY-CO-13) (TP53)	S37	P04637	30
p53	Tumor suppressor protein p53 (antigenNY-CO-13) (TP53)	S6	P04637	30
IGF1R	Insulin-like growth factor 1 receptor protein-tyrosine kinase	Y1280	P08069	32
A6	Twinfilin-1	Y309	Q12792	32
CLK1	Dual specificity protein kinase CLK1	S337	P49759	32
PAK1	p21-activated kinase 1 (alpha) (serine/threonine-protein kinase PAK 1) (PAKa)	S144/S141/S154	Q13153	33
Gab1	GRB2-associated binder 1	Y627	Q13480	34
JUN	Jun proto-oncogene-encoded AP1 transcription factor (c-Jun)	S243	P05412	37
JAK2	Janus protein-tyrosine kinase 2	Y1007+Y1008	O60674	38
p38d MAPK	Mitogen-activated protein-serine kinase p38 delta (MAPK13)	T180+Y182	O15264	38
MEK1	MAPK/ERK protein-serine kinase 1 (MKK1, MAP2K1)	T386	Q02750	38
ERK1 (MAPK3)	Extracellular regulated protein-serine kinase 1 (p44 MAP kinase)	T202	P27361	41
TRKB	BNDF/NT3/4/5 receptor- tyrosine kinase (NTRK2)	Y516	Q16620	42
Tau	Microtubule-associated protein tau	S516	P10636	42
EGFR	Epidermal growth factor receptor-tyrosine kinase (ErbB1)	Y1197	P00533	43

SMC1	Structural maintenance of chromosomes protein 1A	S957	Q14683	44
JNK 1/2/3	Jun N-terminus protein-serine kinase (stress-activated protein kinase (SAPK)) 1 (SAPKg, MA	T183+Y185	P45983	44
FYN	Fyn proto-oncogene-encoded protein-tyrosine kinase	Y213+Y214	P06241	44
PDGFRa	Platelet-derived growth factor receptor kinase alpha	Y754	P16234	45
p70 S6K	Ribosomal protein S6 kinase beta-1 (RPS6KB1, p70S6Ka)	S447	P23443	45
PAK2	p21-activated kinase 2 (gamma) (serine/threonine-protein kinase PAK 2) (PAKg)	S141	Q13177	45
ZAP70	Zeta-chain (TCR) associated protein-tyrosine kinase, 70 kDa	Y292	P43403	46
Caveolin 1	Caveolin 1 (CAV1)	Y14	Q03135	47
WASP	Wiskott-Aldrich syndrome protein	Y291	P42768	48
CaMK1d	Calcium/calmodulin-dependent protein-serine kinase 1 delta	T180	Q8IU85	48
ENO1	Alpha-enolase	Y44	P06733	48
AKT1 (PKBa)	RAC-alpha serine/threonine-protein kinase	Y315	P31749	48
ALK	Anaplastic lymphoma kinase	Y1507	Q9UM73	48
InsR (IR)	Insulin receptor beta chain	Y1189	P06213	49
4E-BP1	Eukaryotic translation initiation factor 4E binding protein 1 (PHAS1)		Q13541	49
STAT1	Signal transducer and activator of transcription 1 beta	S727	P42224	49
CSF1R	Macrophage colony-stimulating factor 1 receptor (Fms)	Y809	P07333	49
MSK1	Mitogen & stress-activated protein-serine kinase 1 (RPS6KA5)	S376	O75582	49
**II. Up-regulated Phosphoproteins (> 150% of Control)**				
BCR	Breakpoint cluster region protein	Y591	P11274	151
p70 S6K	Ribosomal protein S6 kinase beta-1 (RPS6KB1 p70S6Ka)	T412	P23443	153
Fused	Serine/threonine-protein kinase 36 (STK36)	S159	Q9NRP7	156
SYK	Spleen protein-tyrosine kinase	Y525+Y526	P43405	157
PLK4	Polo-like protein kinase 3 (cytokine- inducible kinase (CNK) (SAK, STK18)	T170	O00444	158
B-Raf (RafB)	RafB proto-oncogene-encoded protein-serine kinase	S729	P15056	158
RSK1	Ribosomal S6 protein-serine kinase 1 (RPS6KA1, p90RSK)	Y220+S221	Q15418	159
RSK1/2	Ribosomal S6 protein-serine kinase 1/2 (RPS6KA1/A3, p90RSK)	S363/S369	Q15418	160
ERK4 (MAPK4)	Extracellular regulated protein-serine kinase 4	S186	P31152	160
GSK3a	Glycogen synthase-serine kinase 3 alpha	T19+S21	P49840	161
FER (TYK3)	Fer (fps/fes related) tyrosine kinase	Y402	P16591	163
RSK1	Ribosomal S6 protein-serine kinase 1 (RPS6KA1, p90RSK)	S380	Q15418	163
FAK (PTK2)	Focal adhesion protein-tyrosine kinase	Y576+Y577	Q05397	164
Rb	Retinoblastoma-associated protein 1	T821	P06400	164
FLT3	Receptor-type tyrosine-protein kinase FLT3 (STK1)	Y842	P36888	164
VEGFR2 (KDR)	Vascular endothelial growth factor receptor-tyrosine kinase 2 (Flk1)	Y1059	P35968	166
RSK1	Ribosomal S6 protein-serine kinase 1 (RPS6KA1, p90RSK)	S221	Q15418	167
Tau	Microtubule-associated protein tau	T522	P10636	168
PRKX	Serine/threonine-protein kinase PRKX	T201+T203	P51817	168

eIF2a	Eukaryotic translation initiation factor 2 alpha	S52	P05198	169
RPS6	40S ribosomal protein S6	S235	P62753	170
RIPK2	Receptor-interacting serine/threonine-protein kinase 2 (RIPK2, RIP2, RICK)	S176	O43353	170
PLCg1	1-phosphatidylinositol-4,5-bisphosphate phosphodiesterase gamma-1	Y783	P19174	170
STAT5A	Signal transducer and activator of transcription 5A	Y694	P42229	171
SIT	Signaling threshold-regulating transmembrane adapter 1	Y90	Q9Y3P8	174
Adducin a/g	Adducin alpha, gamma (ADD 1/3)	S726	Q9UEY8	174
MKK4	MAPK/ERK protein-serine kinase 4 (MEK4, MAP2K4)	S257	P45985	177
Sgk223	Tyrosine-protein kinase SgK223	Y413	Q86YV5	178
EGFR	Epidermal growth factor receptor-tyrosine kinase (ErbB1)	Y1172	P00533	178
Ezrin	Cytovillin 2	T567	P15311	179
CHK2	Checkpoint protein-serine kinase 2 (CHEK2)	T383	O96017	179
PLCg2	1-phosphatidylinositol-4,5-bisphosphate phosphodiesterase gamma-2 (PLC R)	Y753	P16885	184
FKHR	Forkhead box protein O1 (FOXO1A)	S319	Q12778	192
APP	Amyloid beta A4 protein	T743	P05067	196
MOK	MOK protein kinase (RAGE)	Y167	Q9UQ07	196
MYPT1	Myosin phosphatase target 1 (PPP1R12A)	T696	O14974	197
PBK	Lymphokine-activated killer T-cell-originated protein kinase	Y74	Q96KB5	198
PRKACB	cAMP-dependent protein-serine kinase catalytic subunit beta	S339	P22694	202
MOK	MOK protein kinase (RAGE)	T159+Y161	Q9UQ07	202
MSK1	Mitogen & stress-activated protein-serine kinase 1 (RPS6KA5)	S212	O75582	202
PLCg2	1-phosphatidylinositol-4,5-bisphosphate phosphodiesterase gamma-2 (PLC R)	Y759	P16885	205
Catenin b	Catenin (cadherin-associated protein) beta 1 (CTNNB1)	Y333	P35222	215
NEK2	NIMA (never-in-mitosis)-related protein-serine kinase 2	S171	P51955	218
NUAK1	NUAK family SNF1-like kinase 1 (ARK5)/Nuak2	T211	O60285	230
p38a MAPK	Mitogen-activated protein-serine kinase p38 alpha (MAPK14)	T180+Y182	Q16539	237
GSK3a	Glycogen synthase-serine kinase 3 alpha	S278+Y279	P49840	248
p70 S6K	Ribosomal protein S6 kinase beta-1 (RPS6KB1, p70S6Ka)	T252	P23443	254
SIK2 (QIK)	Salt-inducible kinase 2	S358	Q9H0K1	257
SYK	Spleen protein-tyrosine kinase	Y323	P43405	261
NBS1	Nijmegen breakage syndrome protein 1 (NBN, Nibrin)	S343	O60934	285
KHS1	Kinase homologous to SPS1/STE20 (MAP kinase kinase kinase protein-serine kinase 5 (MEK	Y31	Q9Y4K4	333
JAK1	Janus protein-tyrosine kinase 1	Y1034	P23458	385
Rb	Retinoblastoma-associated protein 1	S795	P06400	393
SgK269	Tyrosine-protein kinase SgK269 (PEAK1)	Y635	Q9H792	393
RSK1	Ribosomal S6 protein-serine kinase 1 (RPS6KA1, p90RSK)	S363	Q15418	422
STAT1	Signal transducer and activator of transcription 1 beta	Y701	P42224	490
ErbB3 (HER3)	Tyrosine kinase-type cell surface receptor HER3	Y1328	P21860	538

Supplemental Table S4b

KIT	'Mast/stem cell growth factor receptor Kit	Y721	P10721	607
MET	Hepatocyte growth factor (HGF) receptor-tyrosine kinase	Y1230+Y1234+Y1235	P08581	675
Vimentin	VIM (Vimentin)	S34	P08670	998
ATF2	Activating transcription factor 2 (CRE-BP1)	S112	P15336	1279
EGFR	Epidermal growth factor receptor-tyrosine kinase (ErbB1)	Y1110	P00533	1472
CK2a1	Casein protein-serine kinase 2 alpha/ alpha prime (CSNK2A1)	T360+S362	P68400	2690
